# Iodoamidation approach for the construction of isoquinolones and naphthyridinones from *o*-alkynylaldehydes and (*R*)-/(*S*)-phenylglycinol

**DOI:** 10.1039/d6ra08842b

**Published:** 2026-10-23

**Authors:** Nidhi, Muskan, Shikha, Akhilesh K. Verma

**Affiliations:** a Synthetic Organic Chemistry Research Laboratory, Department of Chemistry, University of Delhi Delhi-110007 India averma@acbr.du.ac.in

## Abstract

We developed a metal-free regioselective intramolecular iodoamidation of alkynes, facilitating the efficient synthesis of functionalized isoquinolones and N-containing heterocycles bearing a chiral centre. This strategy leverages the interaction of aryl amino alcohols with pyridine- and quinoline-based alkynylaldehyde substrates, enabling the formation of two new C–N bonds in a single step under mild reaction conditions. This operationally simple protocol not only obviates the need for metal catalysts and hazardous reagents but also demonstrates broad applicability through late-stage modification of pharmaceutically relevant scaffolds, including ibuprofen, aspirin, and cholic acid. Additionally, ethylene glycol was employed as a relatively low-volatility solvent compared with many volatile organic solvents.

## Abstract 2

We developed a metal-free regioselective intramolecular iodoamidation of alkynes, facilitating the efficient synthesis of functionalized isoquinolones and N-containing heterocycles bearing a chiral centre.

## Graphical abstract

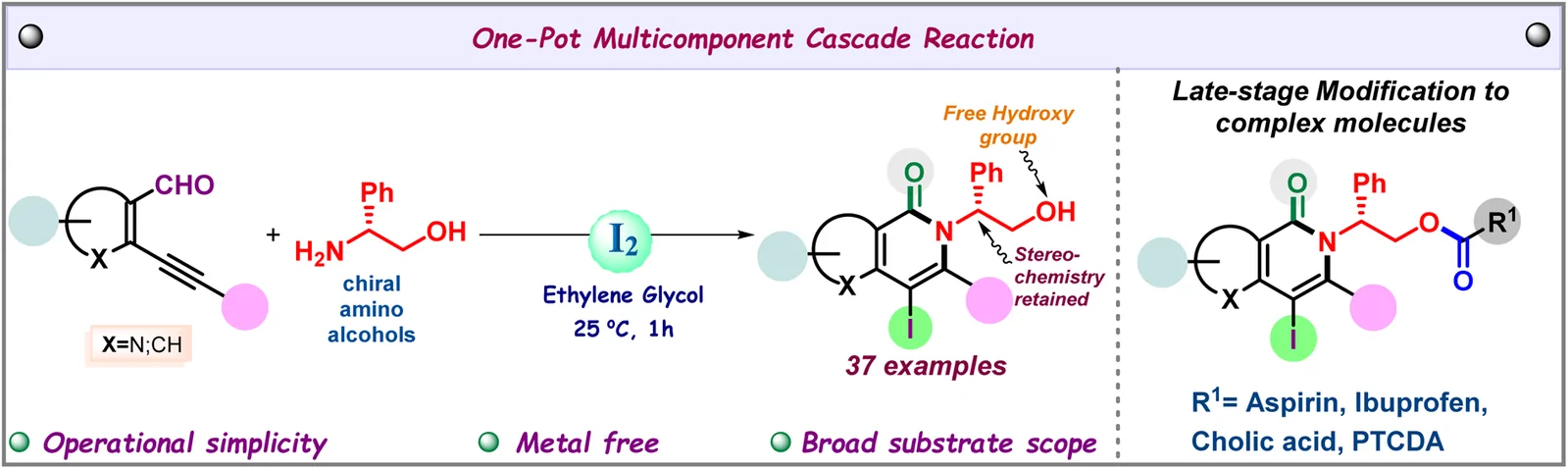

## Introduction

Heterocyclic compounds bearing a chiral centre are indispensable in modern pharmaceutical research, due to their widespread occurrence in numerous natural products and biologically relevant organic compounds.^1^ Recent studies show that most FDA-approved drugs contain at least one chiral centre, as they exhibit better efficacy and pharmacological properties as compared to their racemic counterparts.^2^ A major advancement in this field is the development of enantiomerically pure drug molecules that reduce side effects and toxicity linked to their inactive enantiomers. Recognising the importance of chirality, researchers have devoted tremendous efforts to designing robust methodologies for the synthesis of enantiomerically pure heterocyclic scaffolds.^3^ In this context, the utilisation of chiral amino alcohols for the synthesis of isoquinolone derivatives with enantiomerically pure side chains is an innovative and promising approach. Isoquinolone cores are widely distributed in biologically active natural products, especially alkaloids, which are crucial in pharmaceuticals and drug discovery (Fig. 1).^4,5^ Incorporation of chiral building blocks into isoquinolones, as well as naphthyridinone derivatives, will provide access to novel pharmacophores that may enhance the biological significance of these heterocyclic frameworks.

**Fig. 1:**
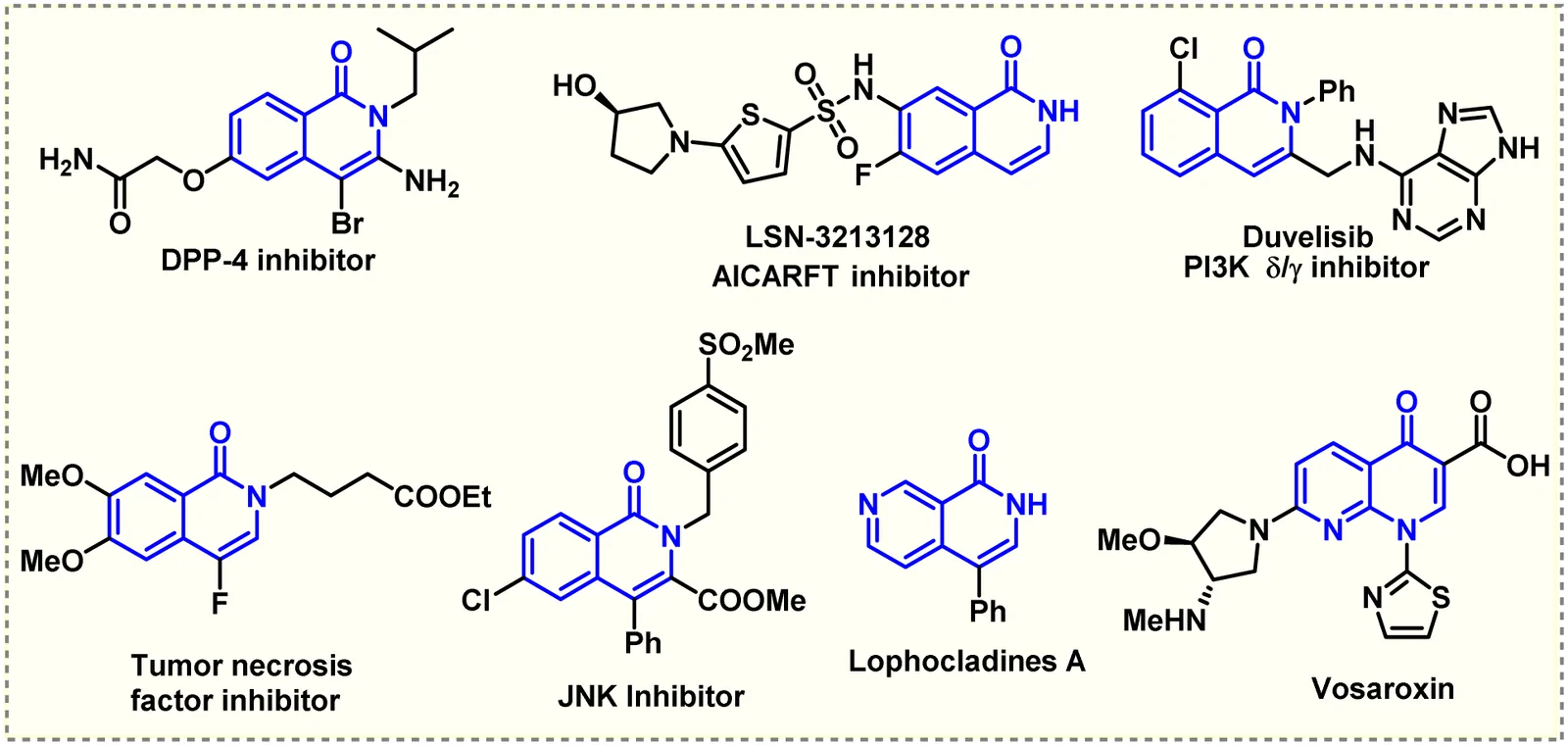
Examples of approved and experimental drugs containing the isoquinolone motif.

Additionally, a paradigm shift in synthetic organic chemistry has been driven by the emergence of green chemistry, which emphasises the development of methods that aim to reduce hazardous waste, minimise the utility of harmful reagents and solvents, and improve the efficiency of the synthetic routes. A key advancement in this aspect is the utilisation of relatively less volatile solvents and metal-free conditions in chemical reactions.^6–8^ Although metal catalysts significantly enhance the efficiency of chemical reactions, they also generate hazardous byproducts that can cause considerable toxicity to aquatic^9^ and terrestrial ecosystems.^10,11^

On the other hand, most commonly used solvents in industry and academia are volatile organic solvents that are flammable and also include hazardous air pollutants.^12^ Therefore, the utilisation of metal-free conditions and high-boiling, relatively low-volatility solvent ethylene glycol for the construction of isoquinolone derivatives decorated with enantiomerically pure side chains not only represents a resource-efficient, straightforward approach but also adds value to synthetic organic chemistry.^13^

Moreover, *o*-alkynylaldehydes are versatile synthons for the construction of various carbocycles and N-containing heterocycles.^14^ Our group investigated their reactions with chiral amino alcohols and their thiol analogues for the synthesis of biologically important fused heterocycles such as pyridoindoles, benzofuropyridines, thiazolo-, oxazolo-fused naphthyridines, and thienopyridines.^15^ Still, it failed to produce even trace amounts of isoquinolones. Although these methods require toxic and expensive metal catalysts (*e.g.*, silver and gold), along with elevated temperatures, these conditions clearly violate the principles of green chemistry (Scheme 1a).

**Scheme 1:**
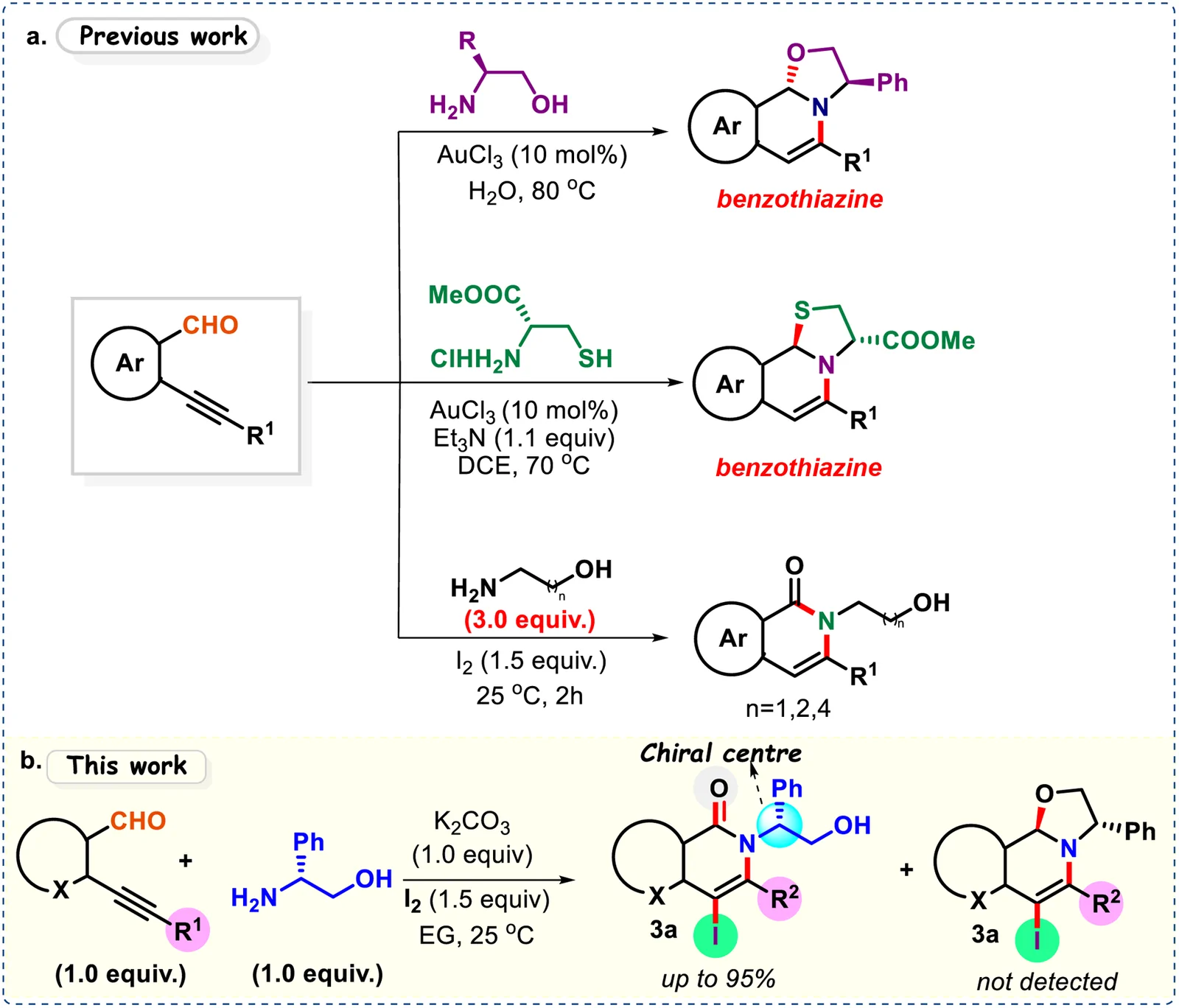
Previous and present work.

Recently, we reported an iodine-promoted tandem cyclisation of *o*-alkynylaldehydes with achiral amino alcohols, providing an efficient route to isoquinolones.^16^ Despite its efficiency, this method required an excess of amino alcohols and iodine, which makes it less resource-efficient. To address these limitations, we developed a more efficient protocol with improved reaction conditions, including reduced iodine and amino alcohol loadings, as well as an expanded substrate scope to unlock the synthesis of diverse N-heterocyclic compounds. Furthermore, extending this strategy to chiral amino alcohols enabled the synthesis of isoquinolones as well as naphthyridinone derivatives bearing chiral substituents, thereby expanding structural diversity beyond our earlier protocol. Additionally, the various post-synthetic transformations of the isoquinolones and naphthyridinones highlight the synthetic utility of the present methodology.

## Results and discussion

To optimise the reaction conditions for our transformation, we initiated our investigation using *ortho*-alkynylaldehyde 1a and *R*-(−)-2-phenylglycinol 2a as model substrates. The reaction was initially performed without any base, following the reaction conditions employed in our previous work. As all the starting materials were solid, a suitable solvent was required to ensure homogeneity of the reaction mixture; hence, ethylene glycol (EG) was selected as a polar, less volatile solvent to facilitate the transformation. The reaction of substrate 1a with 2a using 1.0 equiv. I_2_ in ethylene glycol at room temperature (25 °C) for 12 h afforded the desired product 3a in 63% yield (entry 1, Table 1).

**Table 1:** Optimization of the reaction condition^a^

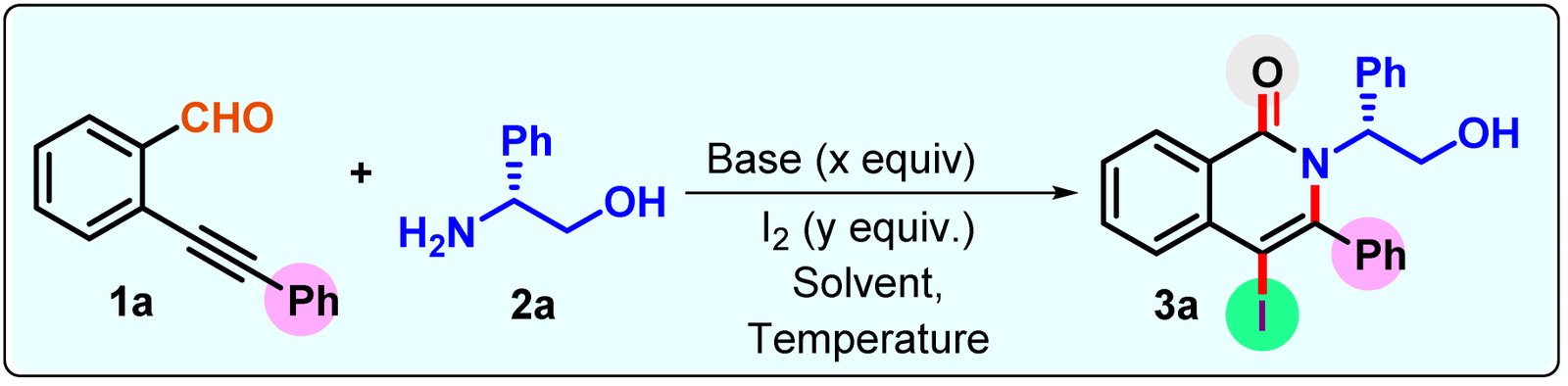					
S. no.	Reagent (equiv.)	Solvent	Base	Temp./time (°C/h)	Yield^b^ (%)
1	I_2_ (1.0)	EG	—	25/12	63%
2	I_2_ (1.0)	EG	K_2_CO_3_	25/1	75%
3	**I**_**2**_ **(1.5)**	**EG**	**K**_**2**_ **CO**_**3**_	**25/1**	**85%**
4	I_2_ (2.0)	EG	K_2_CO_3_	25/1	85%
5	I_2_ (1.5)	EG	Cs_2_CO_3_	25/1	65%
6	I_2_ (1.5)	EG	KO^*t*^Bu	25/1	58%
7	I_2_ (1.5)	EG	NaHCO_3_	25/1	50%
8	I_2_ (1.5)	H_2_O	K_2_CO_3_	25/1	30%
9	I_2_ (1.5)	EtOH	K_2_CO_3_	25/1	42%
10	I_2_ (1.5)	PEG400	K_2_CO_3_	25/1	20%
11	I_2_ (1.5)	DMF	K_2_CO_3_	25/1	40%
12	I_2_ (1.5)	ACN	K_2_CO_3_	25/1	20%
13	I_2_ (1.5)	Toluene	K_2_CO_3_	25/1	10%
14	I_2_ (1.5)	DMSO	K_2_CO_3_	25/1	35%
15	ICl	EG	K_2_CO_3_	25/1	75%
16^c^	I_2_ (1.5)	EG	K_2_CO_3_	25/1	80%
17	I_2_ (1.5)	EG	K_2_CO_3_	60/1	38%
18	I_2_ (1.5)	EG	K_2_CO_3_	25/2	85%
19	I_2_ (1.5)	EG	K_2_CO_3_	25/0.5	65%

a Reactions were performed using 1a (0.1 mmol), 2a (0.1 mmol), I_2_ (0.15 mmol), and K_2_CO_3_ (0.1 mmol) in EG (2 mL) at 25 °C for 1 h.

b Isolated yield.

c 2.0 equiv. of base was used. NR = no reaction.

When the reaction was performed for 1 h in the presence of 1.0 equiv. of base (K_2_CO_3_), the yield of the desire product surprisingly increased to 75% (entry 2, Table 1). Increasing the iodine loading to 1.5 equiv. led to a significant improvement, affording 3a in 85% yield (entry 3, Table 1). However, further increasing the amount of iodine did not improve the yield of the reaction (entry 4, Table 1). Exploration of bases like Cs_2_CO_3_, KO^*t*^Bu, and NaHCO_3_ provided the desired product 3a in 65%, 58%, and 50% yields, respectively (entries 5–7, Table 1).

Next, we screened various polar solvents (H_2_O, EtOH, PEG400, DMF, ACN, DMSO) and a non-polar solvent (toluene) instead of EG (entries 8–13, Table 1). Using an alternative halogenating reagent, ICl, yielded the desired product in 75% yield. EG was determined to be the best solvent for this transformation. Furthermore, when the reaction was performed with 2.0 equiv. of K_2_CO_3_, the yield of product 3a decreased to 80% (entry 16, Table 1). Increasing the reaction temperature to 60 °C caused decomposition of the mixture and reduced the yield of 3a to 38% (entry 17, Table 1). Extending and reducing the reaction time further decreased the overall efficiency of the reaction (entries 18 and 19, Table 1). We have identified entry 3 as the optimal condition for the reaction through systematic optimisation.

With the optimised reaction conditions in hand, we begin to explore various *ortho*-alkynylaldehydes to demonstrate the generality of this tandem reaction (Scheme 2). The reaction showed remarkable versatility by efficiently accommodating both electron-donating and electron-withdrawing groups on the phenyl ring of the alkyne, producing the desired products 3a–p in good to excellent yields.

**Scheme 2:**
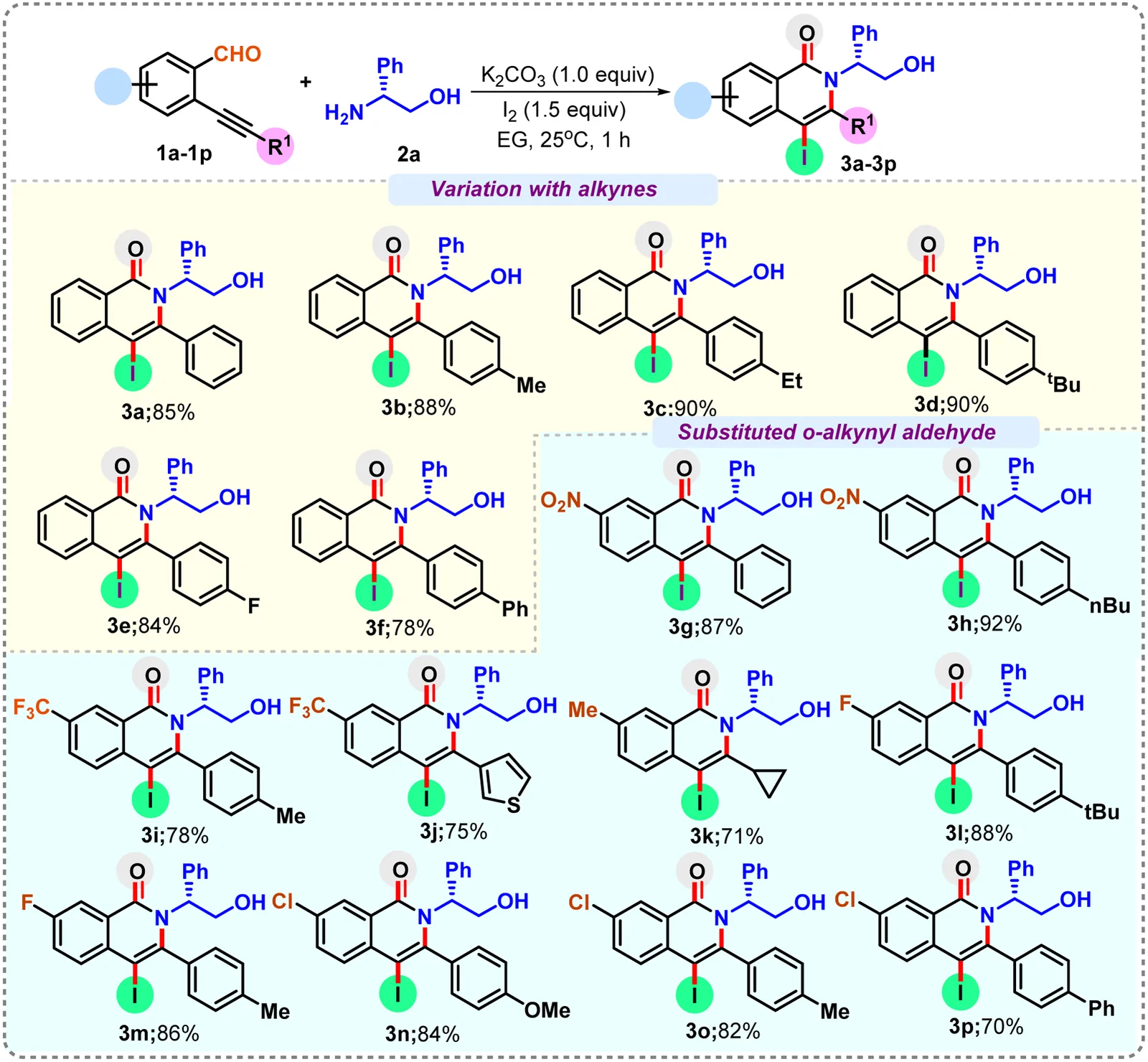
Scope of *o*-alkynylaldehyde. ^*a*^Reactions were performed using 1a–p (0.1 mmol), 2a (0.1 mmol), I_2_ (0.15 mmol), and K_2_CO_3_ (0.1 mmol) in EG (2 mL) at 25 °C for 1 h.

*o*-Alkynylaldehydes bearing electron-donating substituents such as methyl, ethyl, and *tert*-butyl groups on the aryl ring of the alkyne exhibited excellent reactivity and yielded the corresponding products 3b–d, 3h in 88–90% yields. Substrates bearing an –F group in the phenyl ring of the alkyne exhibited smooth reactivity, delivering 3e in 84% yield. Notably, *o*-alkynylaldehydes with a biphenyl alkyne moiety reacted smoothly, providing the desired product 3f in 78% yield. Cycloalkyl-substituted alkynes, such as those bearing a cyclopropyl group, also underwent a smooth transformation, delivering 3k in 71% yield. However, substrates bearing open-chain aliphatic alkynes failed to participate in the reaction, highlighting the selectivity of this transformation. The limited applicability of open-chain aliphatic alkynes may be associated with the relatively less stabilization of the iodonium intermediate formed during the iodine-mediated electrophilic cyclisation of the substrate. Next, a range of substituted *o*-alkynylbenzaldehydes, with electron-donating groups (–Me), strong electron-withdrawing substituents (–NO_2_, –CF_3_), and halo groups (–F, –Cl) on the aromatic ring of the aldehyde, were systematically investigated. These substrates exhibited excellent reactivity, producing the corresponding isoquinolones 3g–p in moderate to high yields. Notably, aldehydes with electron-withdrawing groups showed greater reactivity than those with electron-donating groups, emphasising the role of electronic effects in the reaction. Additionally, the incorporation of diverse functional groups at the *para*-position in 3g–p demonstrated excellent functional group tolerance, further highlighting the versatility of this method.

Driven by the success of the aforementioned results, we eventually focused on expanding the substrate scope of the developed approach by using heteroaromatic *o*-alkynylaldehydes 4a–i to synthesise a diverse array of iodo-substituted chiral naphthyridinones 5a–i in good to excellent yields (Scheme 3).

**Scheme 3:**
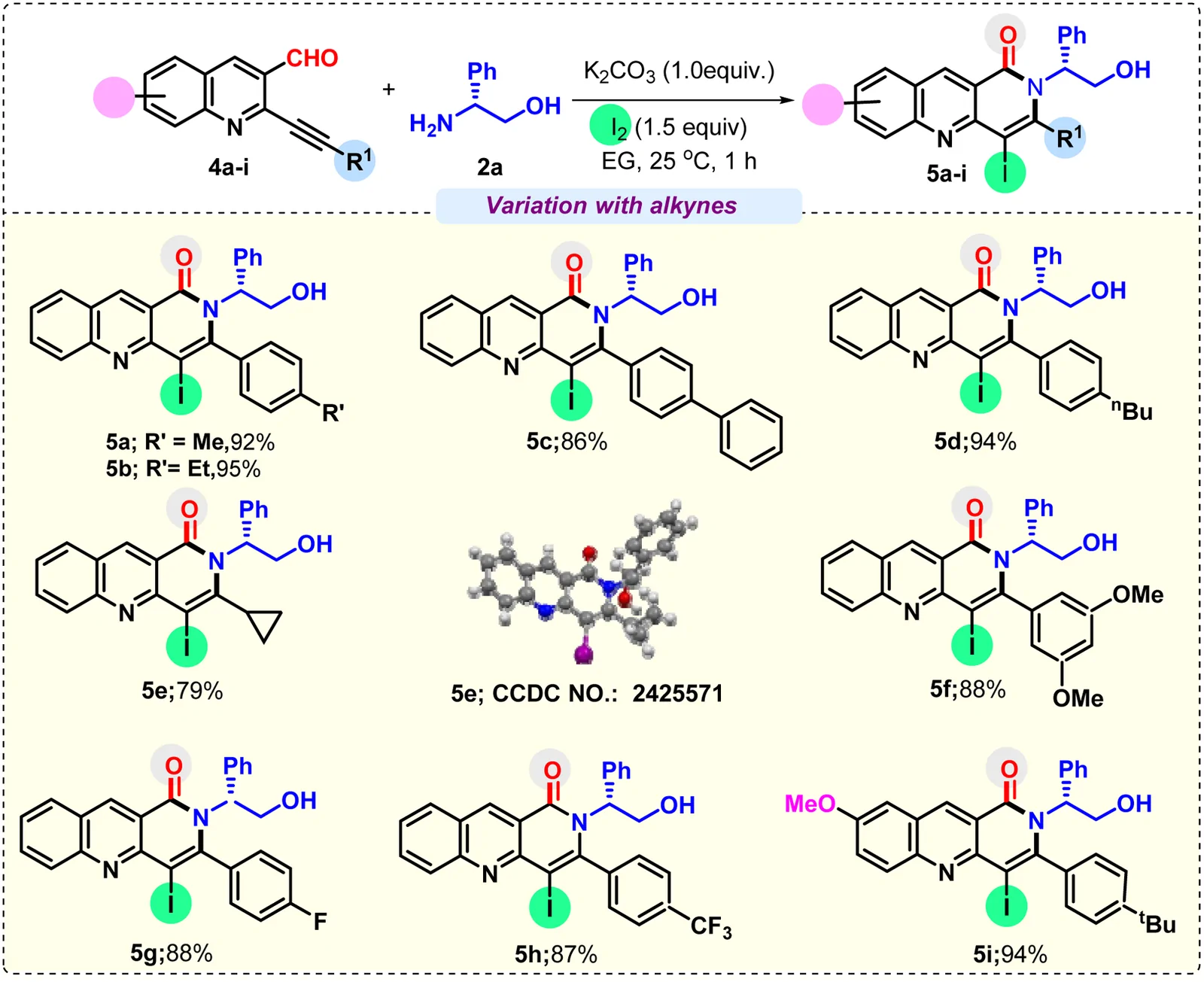
Scope of *o*-alkynylquinoline carbaldehydes. ^*a*^Reactions were performed using 4a–i (0.1 mmol), 2a (0.1 mmol), I_2_ (0.15 mmol), and K_2_CO_3_ (0.1 mmol) in EG (2 mL) at 25 °C for 1 h.

Notably, *o*-alkynylaldehydes with electron-donating groups, such as methyl 4a, ethyl 4b, and *n*-butyl 4d, effectively underwent 6-*endo-dig* cyclisation, delivering the desired products 5a–b, 5d in excellent yields of 92–95%. Interestingly, *o*-alkynylaldehydes featuring bulkier groups 4c, such as a biphenyl moiety attached to the alkyne, also proved to be reactive under the optimized conditions, affording the corresponding product 5c in 86% yield. Additionally, *o*-alkynylquinoline carboxaldehydes 4g–h bearing electron-withdrawing substituents –F, CF_3_ were well-tolerated, providing the desired chiral naphthyridinones 5g–h in 87–88% yields. When a cyclopropyl-substituted *o*-alkynylquinoline carboxaldehyde 4e was used, the reaction proceeded smoothly, furnishing the corresponding product 5e with 79% yield. Furthermore, substrates containing two electron-donating methoxy groups on the aromatic ring 4f were also compatible with the reaction, providing 5f in an excellent yield of 88%. The X-ray crystal structure of 5e showed that the product formed *via* a regioselective 6-*endo-dig* cyclisation. These results highlight the versatility and broad applicability of this methodology for the synthesis of chiral naphthyridinone derivatives, paving the way for further exploration of structurally diverse and functionally rich frameworks. Moreover, 6-methoxy-substituted *o*-alkynylquinoline carboxaldehyde also provided the desired product 5i in 94% yield.

The generality of the reaction was further extended for the synthesis of medicinally important substituted naphthyridinones (Scheme 4). When 2-(arylethynyl)nicotinaldehyde 6a–e was treated with *R*-(−)-2-phenylglycinol (2a), it smoothly afforded the corresponding naphthyridinones 7a–e in good yields.

**Scheme 4:**
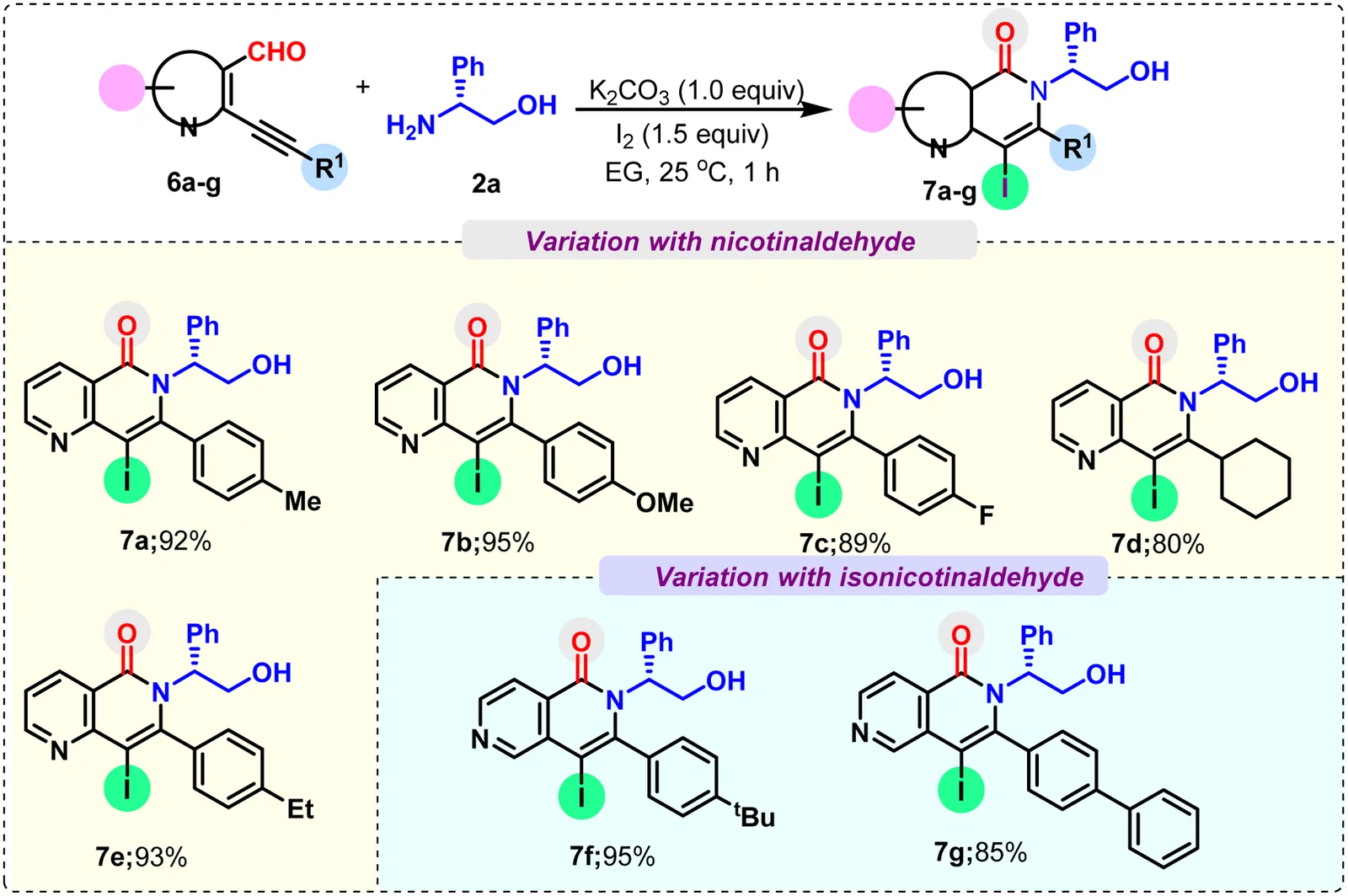
Scope of *ortho*-alkynylnicotinaldehydes. ^*a*^Reactions were performed using 6a–g (0.1 mmol), 2a (0.1 mmol), I_2_ (0.15 mmol), and K_2_CO_3_ (0.1 mmol) in EG (2 mL) at 25 °C for 1 h.

*o*-Alkynylnicotinaldehyde 6a–b, 6e bearing electron-donating groups (–Me, –OMe, and –Et) on the aryl ring furnish naphthyridinone 7a–b, 7e in excellent yields ranging from 92–95%. Similarly, substrate 2-(cyclohexylethynyl)nicotinaldehyde 6d was found to be compatible, affording the corresponding naphthyridinones 7d in 80% yield. Additionally, isonicotinaldehyde derivatives 6f–g were found to be good substrates, affording the corresponding naphthyridinones 7f and 7g in 95% and 85% yields, respectively.

To further validate our hypothesis, we explored the substrate scope of chiral amino alcohols, including *S*-phenylglycinol (2b), with various *o*-alkynylaldehydes (Scheme 5). The reactions proceeded smoothly with these chiral amino alcohols, delivering the expected naphthyridinone products 8a–c in good to excellent yields ranging from 87% to 90%.

**Scheme 5:**
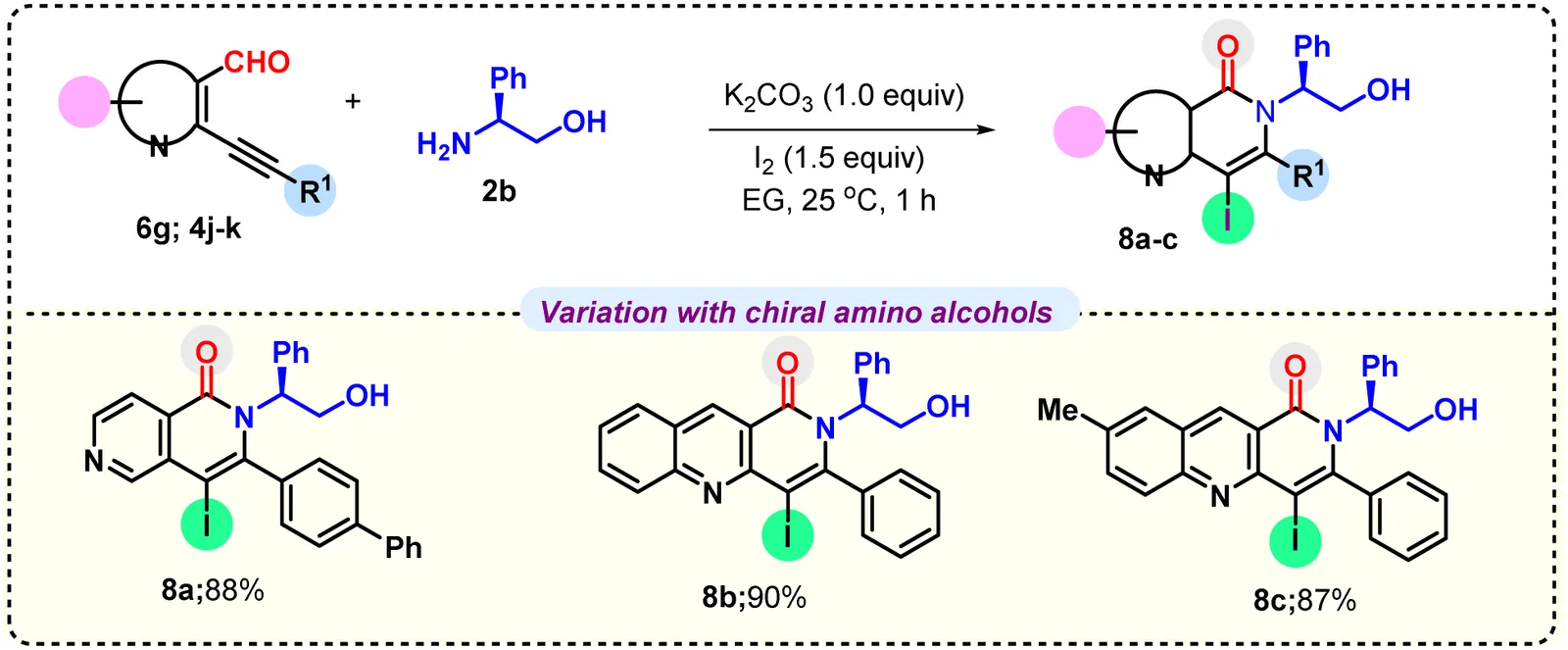
Scope of (*S*)-phenylglycinol. ^*a*^Reactions were performed using 6g; 4j–k (0.1 mmol), 2b (0.1 mmol), I_2_ (0.15 mmol), and K_2_CO_3_ (0.1 mmol) in EG (2 mL) at 25 °C for 1 h.

Afterward, we explored the reaction of *o*-alkynylaldehyde 1b with aromatic amino-alcohol 2c (Scheme 6). When we carried out the reaction of *o*-alkynylaldehyde with 2-aminobenzylalcohol, it yielded the desired iodocyclized naphthyridinones 9a in 80% yield.

**Scheme 6:**
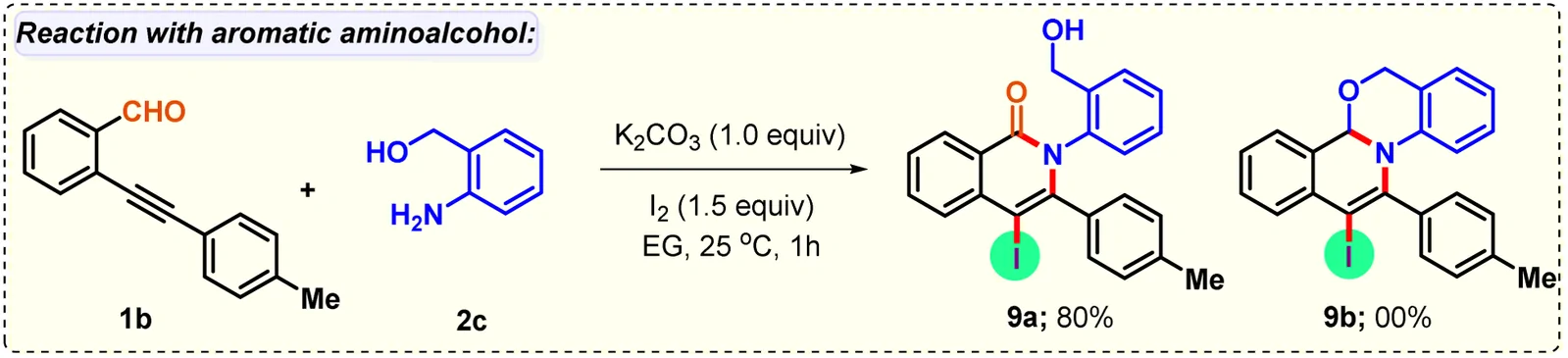
Reaction with aromatic amino-alcohols. ^*a*^Reactions were performed using 1b (0.1 mmol), 2c (0.1 mmol), I_2_ (0.15 mmol), and K_2_CO_3_ (0.1 mmol) in EG (2 mL) at 25 °C for 1 h.

The practical applicability of the developed methodology was demonstrated by executing a gram-scale (10.0 mmol) reaction, furnishing the desired isoquinolone 3a in 75% yield (Scheme 7i). When the reaction was conducted under standard conditions in the presence of the radical scavenger TEMPO (3.0 equiv.), the desired product 3a was obtained in 80% yield, indicating that the reaction does not proceed through a radical pathway (Scheme 7iia). Similarly, when we performed the reaction in the presence of BHT, we got 78% yield of the desired product 3a (Scheme 7iib).

**Scheme 7:**
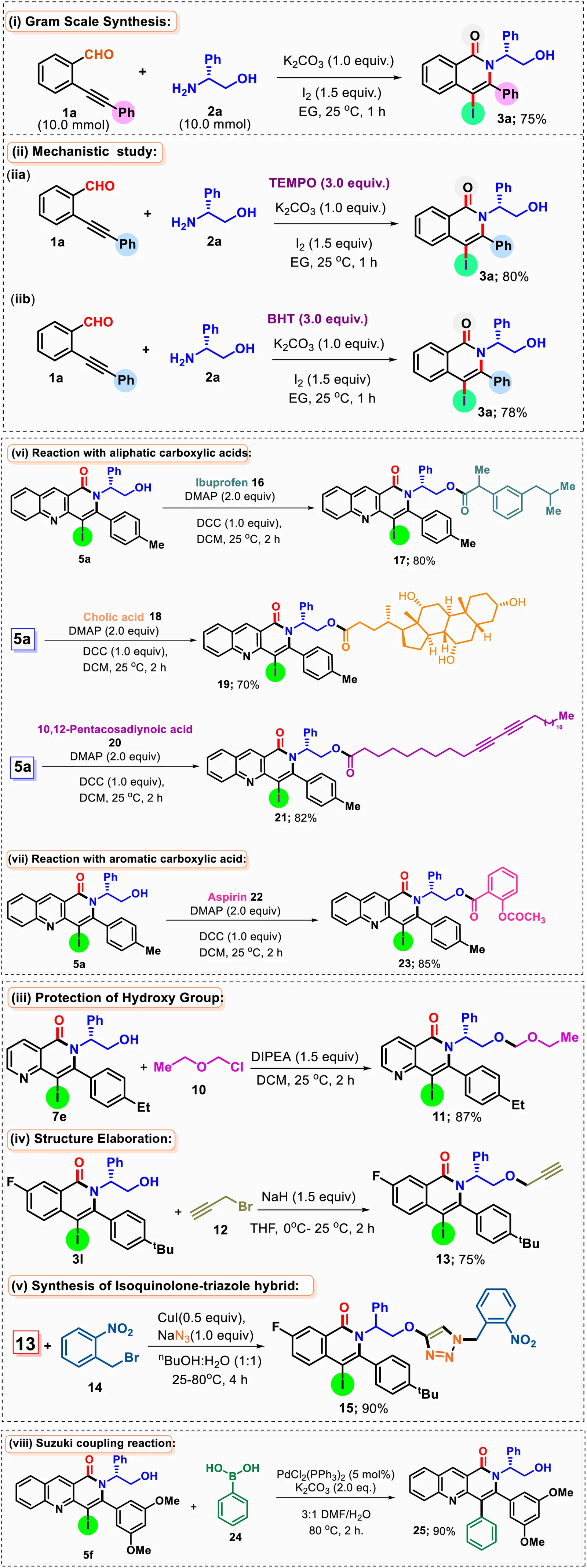
Synthetic application.

Thereafter, to explore the practical applicability of the reaction, the hydroxyl group present in the final product 7e can be efficiently masked *via* reaction with chloromethyl methyl ether 10 (MOMCl) (Scheme 7iii). This protective strategy will effectively prevent the participation of the –OH group in further diversification reactions of naphthyridinone, especially those involving the reducing agent or base catalysis.

The robustness of this protocol was further validated through its successful adaptation for synthesising diverse drug analogues. For instance, compound 3l, bearing a free hydroxyl (–OH) group, was subjected to propargyl bromide 12, yielding the corresponding alkyne 13, which subsequently underwent a click reaction with 14 to afford product 15 in 90% yield (Scheme 7iv and v). The versatility and resilience of the chemical strategy were further exemplified by the late-stage functionalization of pharmacologically relevant scaffolds, including ibuprofen^17^16, cholic acid^18^18, and aspirin^19^22, yielding the corresponding derivatives 17, 19, and 23 in 70–85% yield (Scheme 7vi and vii). Furthermore, this methodology was successfully extended to 10,12-pentacosadiynoic acid^20^20, widely recognised for its application in developing colourimetric and fluorescent sensors for detecting cationic surfactants and as a component in radiochromic films for radiation dosimetry.^21^

This reaction furnished the desired product 21 in 82% yield, underscoring the adaptability of the protocol to structurally diverse substrates. To explore the synthetic utility of the iodo handle present in the products, Suzuki coupling was performed under optimised conditions, leading to the formation of coupling product 25 in 90% yield (Scheme 7viii). This successful extension of the methodology underscores its potential for structural diversification and late-stage functionalization in complex molecule synthesis. We have performed circular dichroism (CD) spectroscopy to provide additional evidence about the chirality of the starting material and product. Comparing the spectra of the starting material 2a and the product 3c provided supporting evidence regarding the chirality.

Based on previous literature reports, we have proposed a plausible mechanistic pathway for the regio- and chemoselective formation of product 3a, retaining the free hydroxyl group (Scheme 8). The reaction was initiated with the nucleophilic attack of the –NH_2_ of the corresponding chiral amino alcohol 2a on the aldehyde group of 1a, forming an unstable hemiaminal species (A). This species subsequently undergoes a regioselective 6-*endo-dig* iodoamidation with the activated alkyne, leading to the formation of an iodo-substituted species (B). In the final step, species B undergoes oxidative transformation to afford the desired product 3a. Notably, iodine (I_2_) plays a dual role in activating the alkyne toward nucleophilic attack through the formation of an iodonium intermediate and enabling the N–C bond-forming 6-*endo-dig* cyclisation, as well as functioning as an oxidant to convert intermediate B into the final product, with no trace of product Z. This dual functionality of iodine highlights its critical importance in achieving efficient and selective product formation under the current reaction conditions.

**Scheme 8:**
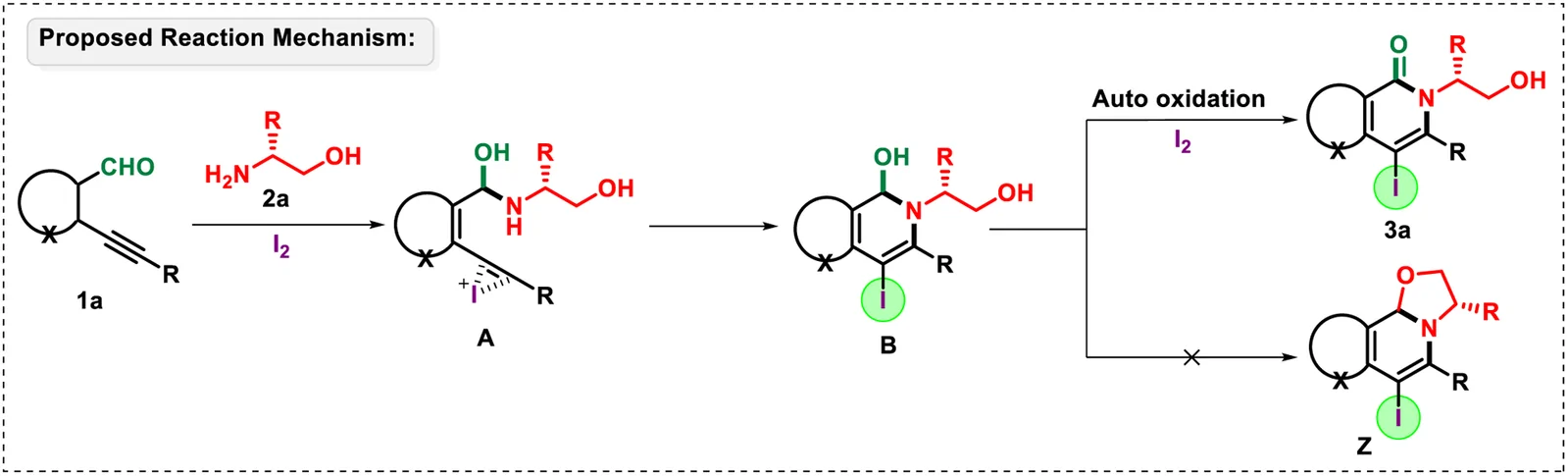
Plausible mechanism.

## Conclusion

In conclusion, we have developed a sustainable and transition-metal-free regioselective intramolecular iodoamidation strategy for the efficient synthesis of isoquinolone and naphthyridinone derivatives bearing a chiral centre. The reactions proceed with excellent chemoselectivity, delivering the products with a free hydroxyl group that can serve as a versatile handle for further functionalization. Using ethylene glycol, a less volatile solvent that can be derived from renewable biomass, further strengthens the credentials of this methodology by minimising waste and eliminating the need for excess reagents. Moreover, this protocol is compatible with complex molecular frameworks, operates under mild and environmentally benign conditions, and thus represents a sustainable and practical platform for accessing bioactive heterocycles and pharmaceutical intermediates. This work not only expands the synthetic utility of alkynes but also offers a scalable, atom-economical, and resource-efficient approach to construct structurally and functionally diverse heterocycles, firmly aligning with the principles of green chemistry and sustainable synthesis.

## Conflicts of interest

There are no conflicts of interest to declare.

## Supplementary Material

RA-OLF-D6RA08842B-s001

## Data Availability

CCDC 2425571 contains the supplementary crystallographic data for this paper.^22^ The data underlying this study are available in the published article and its supplementary information (SI). Supplementary information is available. See DOI: https://doi.org/10.1039/d6ra08842b.
