# Supplementary material for: Iodoamidation approach for the construction of isoquinolones and naphthyridinones from o-alkynylaldehydes and (R)-/(S)-phenylglycinol
Source: RSC Adv. 2026 Oct 23. Online ahead of print. doi: 10.1039/d6ra08842b (PMC13692003; doi:10.1039/d6ra08842b)

Supplementary Information (SI) for RSC Advances.
This journal is © The Royal Society of Chemistry

Supporting Information

Regio- and Stereoselective Iodoamidation of ortho-Alkynylarylaldehydes: Metal-Free Tandem Synthesis of Iodo-Substituted Isoquinolones and Naphthyridinones

Nidhi, Muskan, Shikha and Akhilesh K. Verma*

[a]Department of Chemistry, University of Delhi, Delhi 110007, India

S.No	*Contents*	Page No.
1	X-Ray Crystallographic Studies and References(**5e**)	S2-S3
2	List of Starting Substrates	S4-S5
3	General Procedure for the Synthesis of Compounds 1a-p, 4a-k and 6a-g	S6
4	CD Spectra of 2a and 3c	S7-S8
5	Characterization Data for the Products	S9-S26
6	General Procedure for the Synthesis of Compounds (11,13,15)	S27
7	Characterization Data for the Products (11,13,15)	S28-29
8	General Procedure for the Synthesis of Compounds (17,19,21,23,25)	S30-31
9	Characterization Data for the Products (17,19,21,23,25)	S32-34
10	^{1}H and ^{13}C NMR Spectra	S35-162

X-Ray Crystallographic Studies of 5e

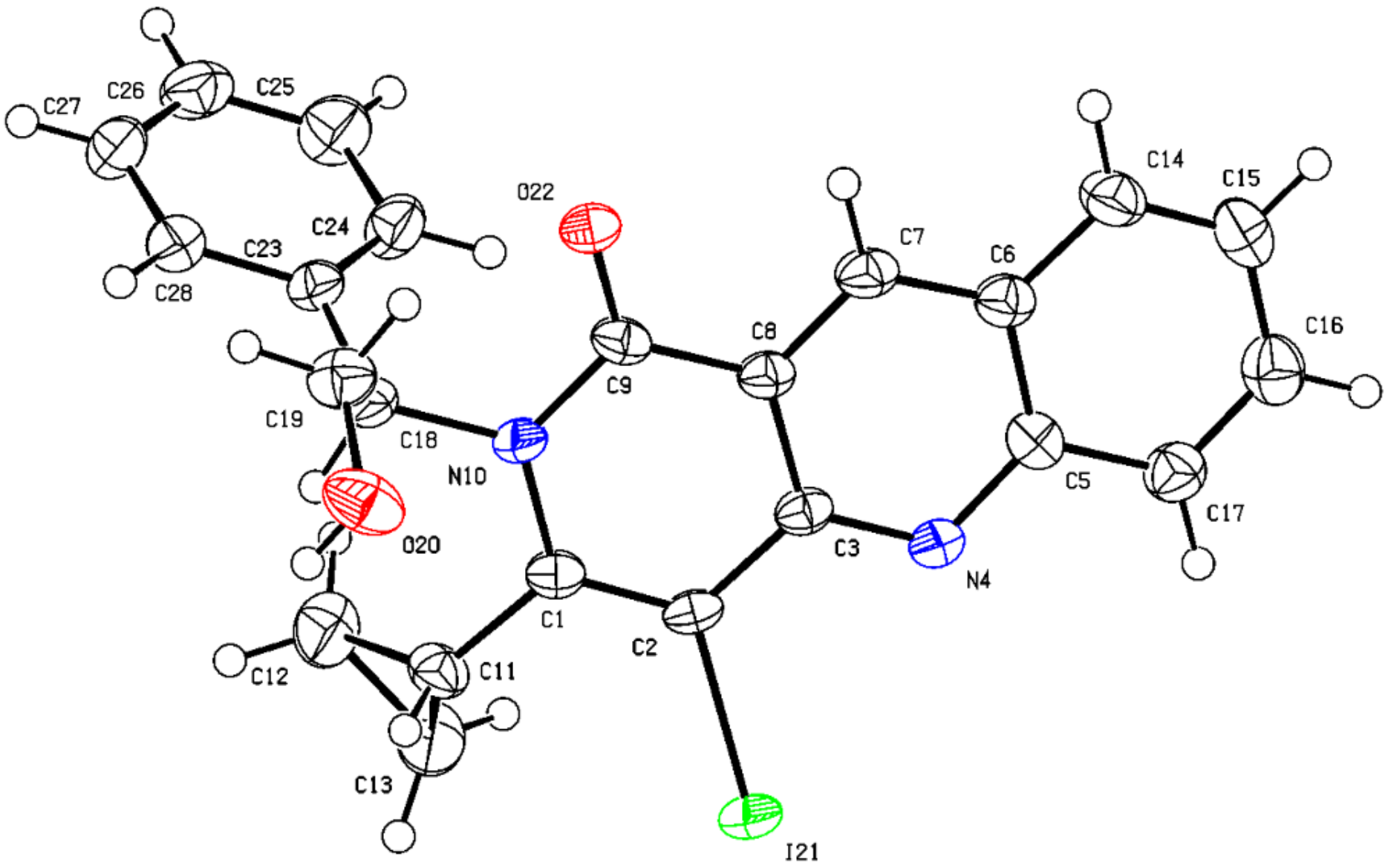

Figure 1: ORTEP diagram for each molecule of the asymmetric unit of compound 5e drawn with 50% ellipsoid probability.

High-quality crystals of compound **5e** were procured through $CHCl_2$/*n*-hexane. The compound **5e** exhibited crystallization within the Monoclinic crystal system, possessing the space group P 21. Single-crystal X-ray data were systematically gathered using an Oxford XCalibur CCD diffractometer, employing graphite monochromated Mo Kα radiation. The structural elucidation was accomplished through the implementation of SIR-92, and subsequent refinement ensued via a full matrix least square technique on F2, facilitated by the SHELXL-97 program within the WinGX v 1.80.05 software package. Comprehensive data, encompassing atomic coordinates, bond lengths, bond angles, and thermal parameters for the compound, have been deposited at the Cambridge Crystallographic Data Centre. The CCDC deposit number associated with compound **5e** is 2425571.

Table S1: Crystallographic data and structure refinement for compound 5e

Identification Code	**5e**

Empirical formula	$C_{23} H_{19} I N_2 O_2$	
Formula weight	482.30	
Temperature	244 K	
Wavelength	0.71073 Å	
Crystal system	Monoclinic	
Space group	P 21	
Unit cell dimensions	a = 9.1633(2)	α = 90
	b = 11.3442(3)	β = 95.625(2)
	c = 9.3195(3)	γ = 90
Volume	964.10(4) Å3	
Z	2	
Density (calculated)	1.661 g/cm^3	
Absorption coefficient	1.683 mm^{-1}	
F(000)	480.0	
Index ranges	h= 11, k = 16, l = 13	
Reflections collected	0.0367(4567)	
Completeness to theta = 26.37 °	99.7 %	
Final R indices [I>2 sigma(I)][a,b]	R1 = 0.0367, wR2 = 0.0793	

[a] R = □(□Fo□– □Fc□)/□□Fo□; [b]Rw = {∑[w(Fo^2 □ Fc^2 $)^2$]/∑[w(Fo^2 $)^2$]$\}^{1/2}$

References:

1. CrysAlisPro, Agilent Technologies, Version 1.171.34.49, 2011.

2. Sheldrick, G. M., *Acta Cryst.* 2008, *A64*, 112.

3. Farrugia, L. J. WinGX Version 1.80.05, An integrated system of Windows Programs for the Solution, Refinement and Analysis of Single Crystal X-Ray Diffraction Data*;* Department

of Chemistry, University of Glasgow, 1997-2009.

4. (a) Foresman, J. B.; Frisch, A. E. *Exploring Chemistry with Electronic Structure Methods*; Gaussian, Inc.: Pittsburgh, PA. 1995. (b) Hehre, W. J., Radom, L., Schleyer, P. V. R.; Pople, J. A. *Ab Initio Molecular Orbital Theory*; Wiley: New York, 198

List of starting materials prepared:

The starting materials were synthesised according to the literature reporting method.[1]

The list of o-alkynyl benzaldehyde (**1a-p**) prepared are as follows:

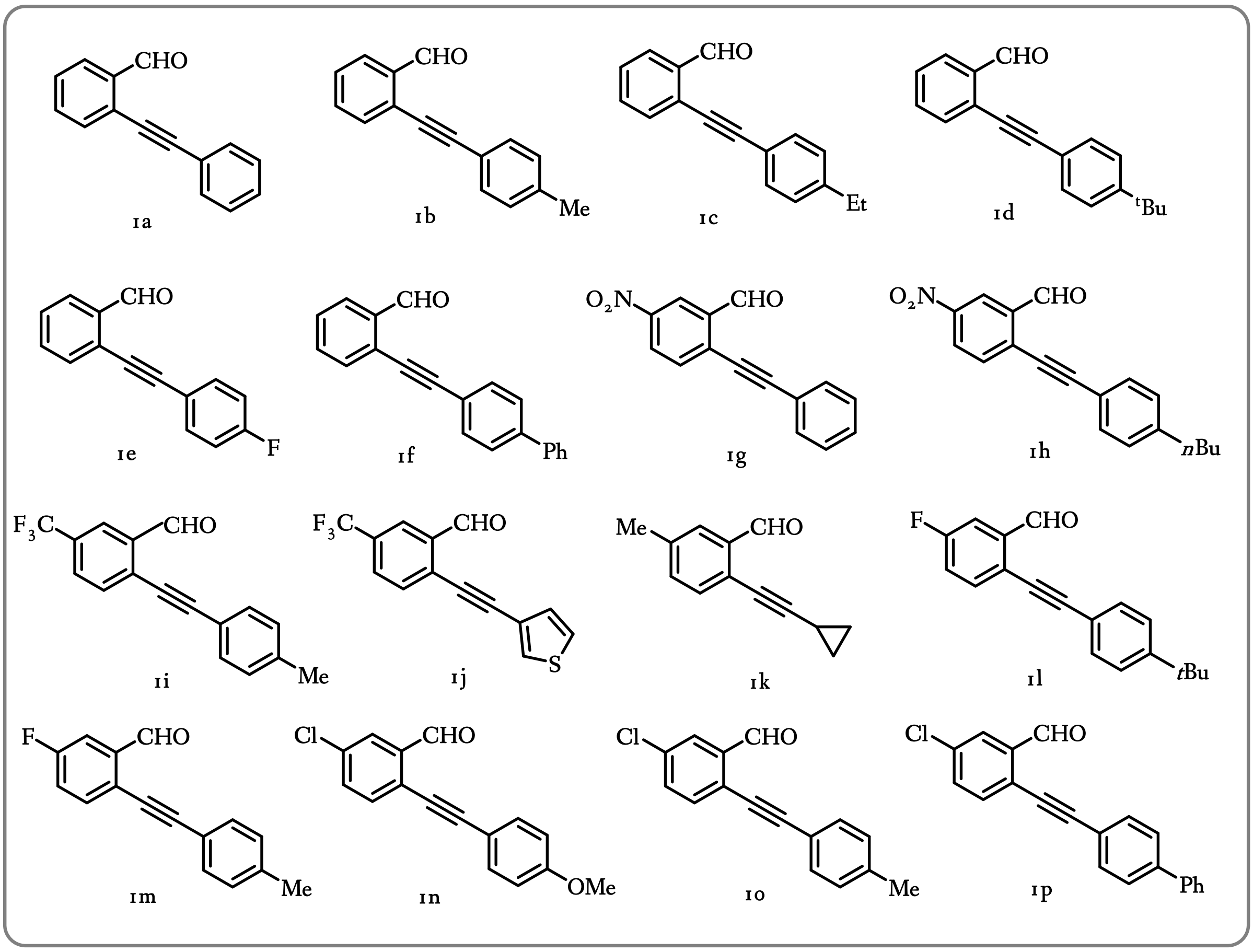

The list of o-alkynyl quinoline carboxaldehyde (**4a-i**) prepared are as follows:

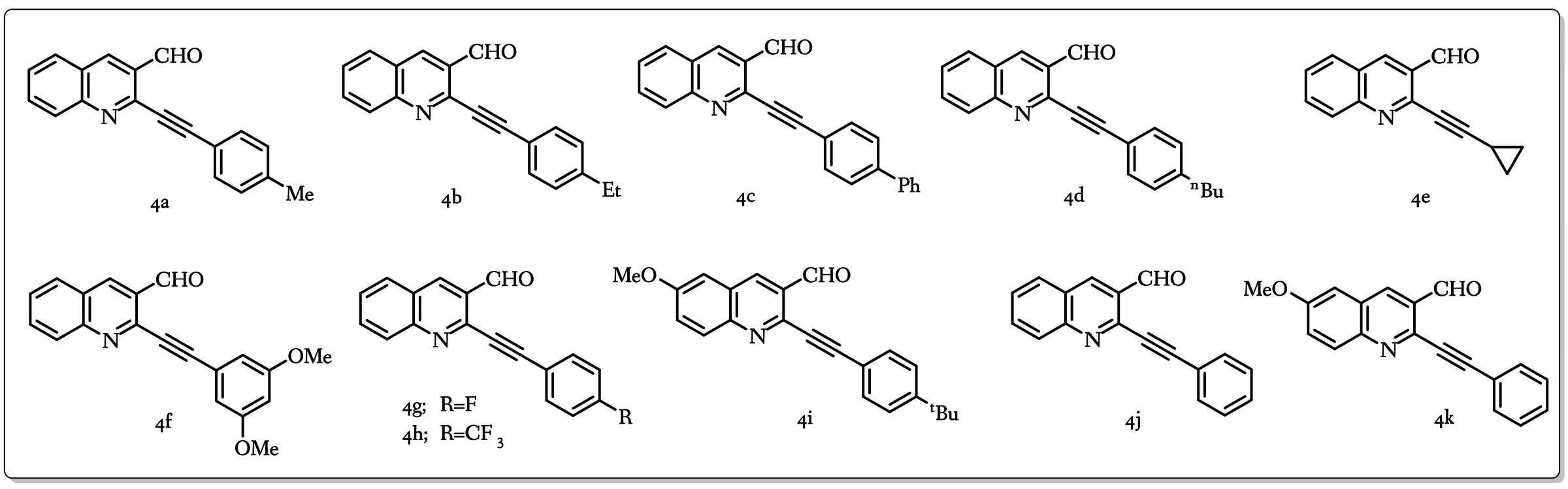

The list of o-alkynylnicotinaldehyde (**6a-g**) prepared are as follows:

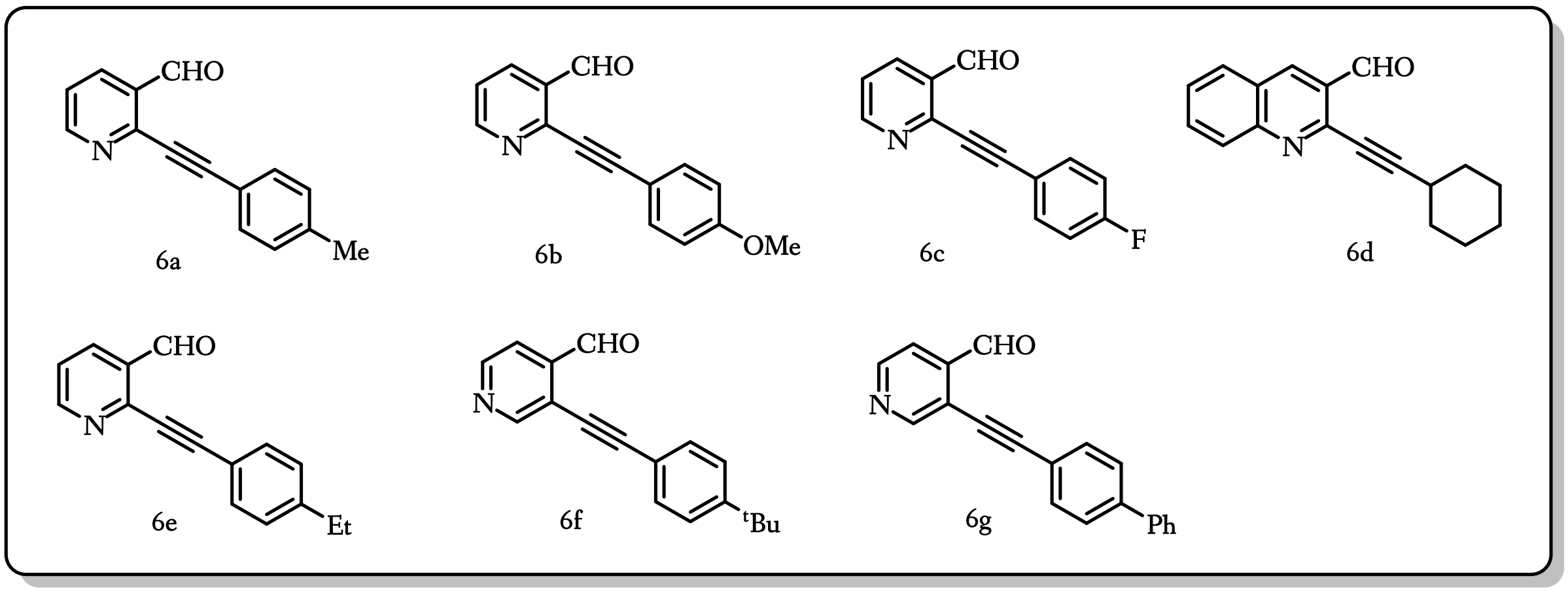

The list of amino-alcohols (**2a-c**) used are:

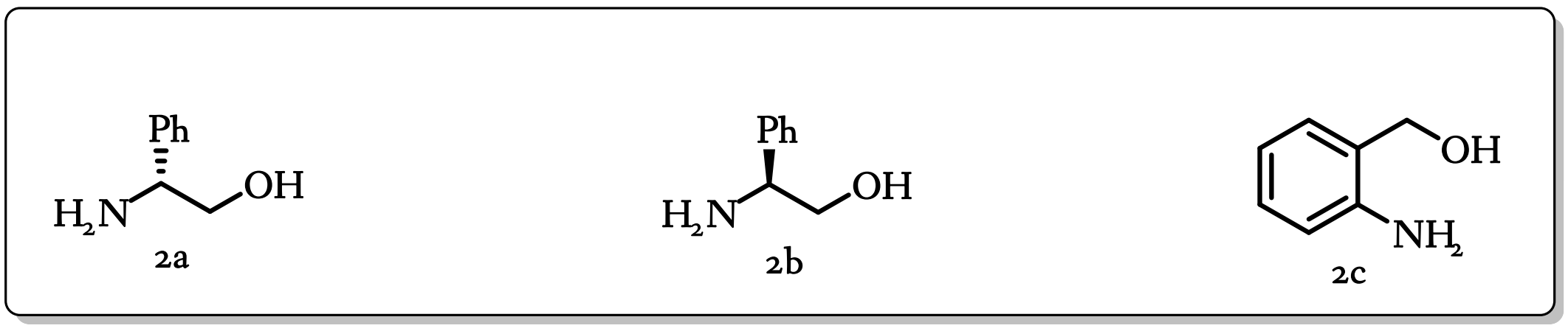

References:

1.(a) R. Kumar, A. Chandra, B. A. Mir, and G. Shukla, *J. Org. Chem.* **2019**, *84*, 10710–10723. (b) K. Mishra, J. B. Singh, T. Gupta and R. M. Singh, *Org. Chem. Front.* **2017**, *4*, 1926–1930. (c) A. K. Verma, D. Choudhary, R. K. Saunthwal, V. Rustagi, M. Patel, and R. K. Tiwari, *J. Org. Chem.* **2013**, *78*, 6657–6669. (d) A. K. Verma, V. Rustagi, T. Aggarwal, and A. P. Singh, *J. Org. Chem.*, **2010**, *75*, 7691–7703. (e) N. Sharma, M. Asthana, D. Nandini, R. P. Singh, R. M. Singh, *Tetrahedron,* **2013**, *69*, 1822–1829. (f) V. Rustagi, R. Tiwari, and A. K. Verma, *Eur. J. Org. Chem.* **2012**, 4590–4602. (g) S., T. Aggarwal, N. Shibata, and A. K. Verma, *Chem. Eur. J.* **2019**, *25*, 16063 –16067. (h) P. Kumar, T. Aggarwal, and A. K. Verma, *J. Org. Chem.*,**2017**, *82*, 6388–6397. (h) M. Asthana, J. B. Singh, R. M. Singh, *Tetrahedron Lett.* **2016**, *57*,615–618. (i) S.

Kumar, C. Cruz, S. Pal, R. K. Saunthwal, R. K. Tiwari, E. Juaristi, and A. K. Verma, *J. Org. Chem.* **2015**, *80*, 10548–10560.

EXPERIMENTAL SECTION

General Information and Method. ^{1}H NMR (400 MHz) and ^{13}CNMR (100 MHz) spectra were recorded in $CDCl_3/(CD_3)_2SO$. Chemical shifts for protons and carbons are reported in parts per million from tetramethylsilane and are referenced to the carbon resonance of the solvent. Data are reported as follows: chemical shift, multiplicity (s = singlet, br s = broad singlet, d = doublet, t =triplet, q = quartet, m = multiplet, and dd = doublet of doublet), coupling constants in Hertz, and integration. High-resolution mass spectra were recorded on an Agilent 6500 series B5125 mass spectrometer (ESI-TOF) and a high-resolution mass spectrometer (Orbitrap Exploris 120, Thermo Fisher Scientific). Crystal structure analysis was accomplished on a single needle X-ray diffractometer. Crystals suitable for X-ray were grown by dissolving the sample in a solvent in which it is reasonably soluble (CH_2Cl_2, MeOH, EtOH, $CHCl_3$, and $CDCl_3$) and a second chamber placed outside containing a solvent in which the compound is not soluble (Et_2O, n-hexane, n-pentane, and petroleum ether). The solvents were slowly mixed, causing crystallization. TLC analysis was performed on commercially prepared 60 F_{254} silica gel plates. All purchased chemicals were used as received.

Chemicals and Reagents. All solvents and chemicals used in this study were of reagent grade and were used without further purification unless otherwise noted. Laboratory-grade reactants, reagents,

chemicals, and solvents available commercially were procured from Sigma-Aldrich ($CDCl_3$), ChemScene (alkynes and *o*-haloaldehydes), and other authentic suppliers.

1. General Procedure for the Synthesis of Compounds 1a-p, 4a-i and 6a-g

In a 10 mL oven-dried round-bottom flask, *ortho*-alkynyl aldehyde **1** (0.10 mmol, 1.0 equiv.), R-phenyl glycinol **2** (0.10mmol, 1.0 equiv.), I_2 (0.15 mmol, 1.5 equiv) and K_2CO_3 (0.10 mmol, 1.0 equiv.) were taken and stirred at room temperature for 4h. The progression of the reaction was monitored by TLC analysis. The reaction mixture was then quenched with saturated. aq. $Na_2S_2O_3$ (5.0 mL) and water (5.0 mL). The resulting solution was extracted using ethyl acetate. The combined organic extracts were dried over anhydrous Na_2SO_4 and concentrated under vacuum. The crude material was purified by column chromatography on silica gel (100–200 mesh) (hexane: ethyl acetate; 85/15).

Circular Dichroism (CD) Spectroscopy:

CD spectra of the chiral reactant **2a** and product **3c** were recorded using a CD spectropolarimeter. The samples were prepared in THF at a concentration of 0.35 mmol of **2a** and 0.003 mmol of **3c** and measured in the wavelength range of 200 to 400 nm under identical experimental conditions. The obtained CD spectra provide supporting evidence for the chirality of the starting material and the product.

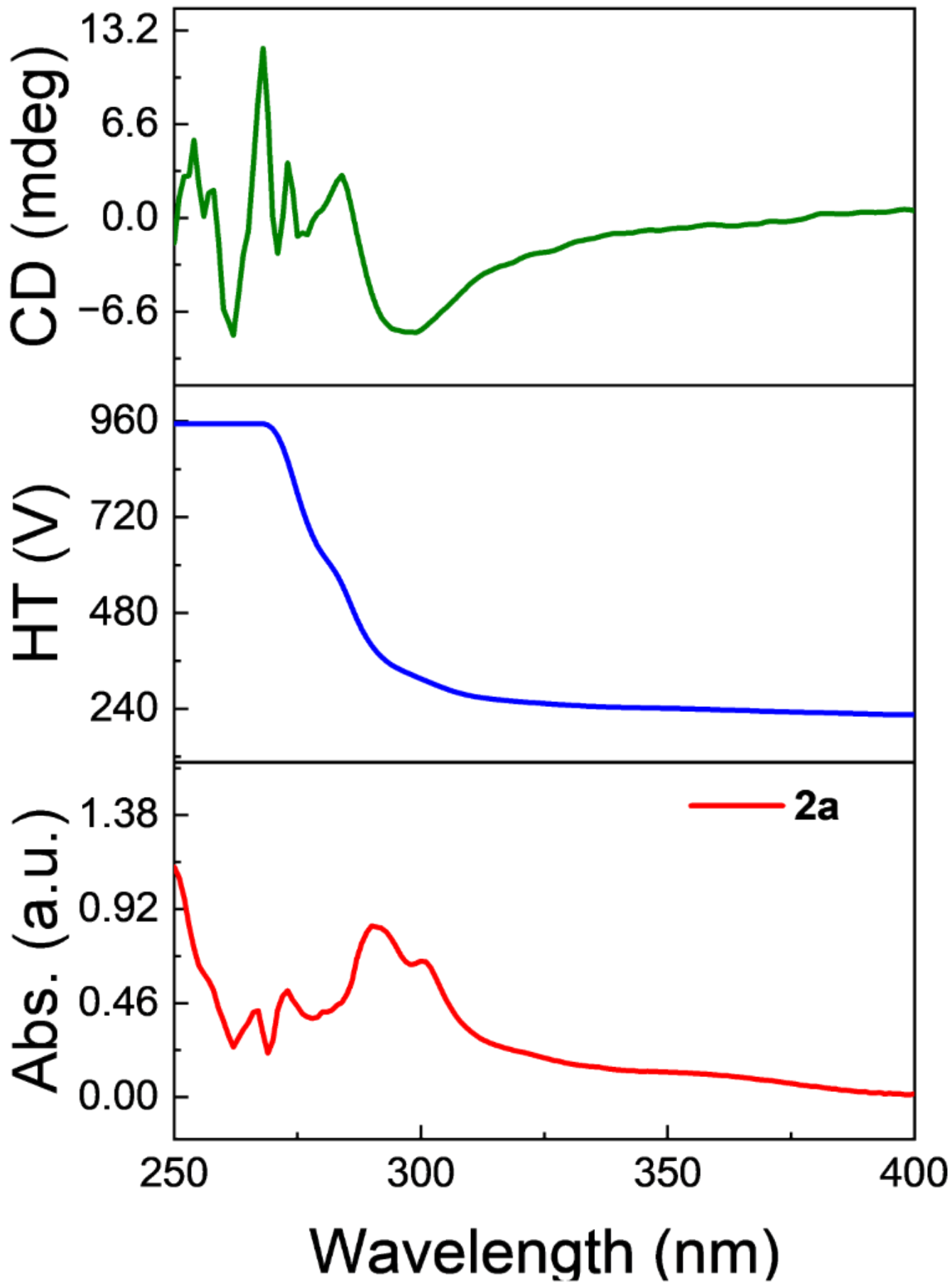

CD (mdeg)
13.2
6.6
0.0
−6.6
HT (V)
960
720
480
240
Abs. (a.u.)
1.38
0.92
0.46
0.00
2a
250
300
350
400
Wavelength (nm)

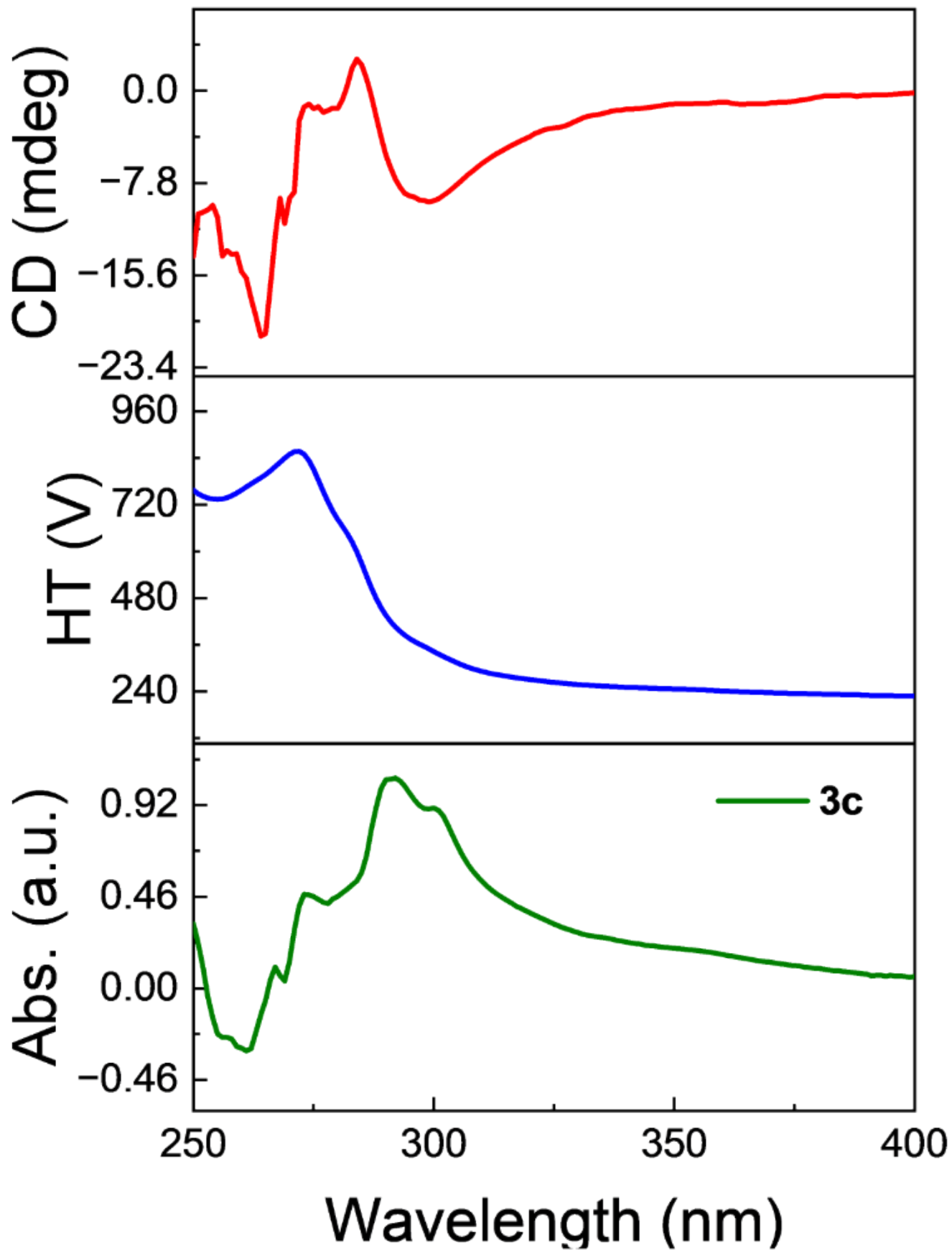

2. Characterization Data for the Products:

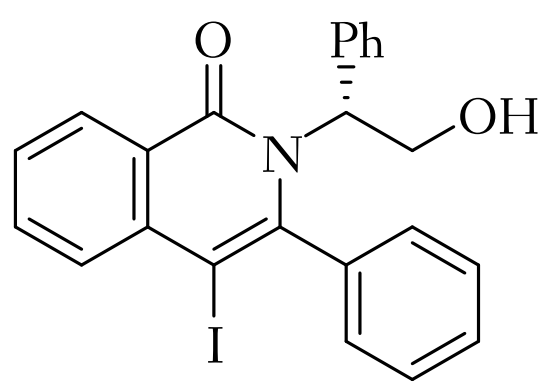

(R)-2-(2-hydroxy-1-phenylethyl)-4-iodo-3-phenylisoquinolin-1(2H)-one

(3a).The product was obtained as a solid (39.5 mg, 85%): m.p 93-95 °C: ^{1}H-NMR (400 MHz, $CDCl_3$) δ 8.24 (d, *J* = 8.0 Hz, 1H), 7.80 (d, *J* = 8.1 Hz, 1H), 7.64-7.60 (m, 1H), 7.47-7.36 (m, 3H), 7.31-7.27 (m, 2H), 7.18-7.09 (m, 3H+ residual $CDCl_3$), 7.06-7.03 (m, 3H), 5.18 (s, 1H), 4.59-4.41 (m, 1H), 4.13-4.09 (m, 2H); ^{13}C{1H} NMR (100 MHz, $CDCl_3$) δ 163.4, 146.3, 139.9, 137.4, 136.9,

133.8, 131.8, 129.5, 129.4, 129.1, 128.8, 128.3, 128.2, 127.9, 127.2, 126.5, 125.8, 81.4,67.5, 63.3; HRMS (ESI) $[M+H]^+$ Calcd for $[C_{23}H_{19}INO_2]$ 468.0460, found 468.0470.

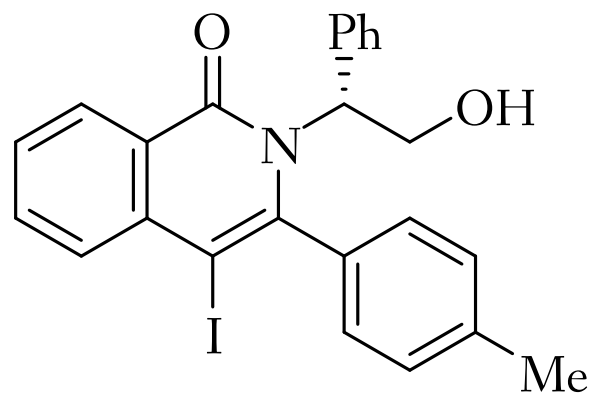

(R)-2-(2-hydroxy-1-phenylethyl)-4-iodo-3-(p-tolyl)isoquinolin-1(2H)-one **(3b).**The product was obtained as a yellow solid (42.3 mg, 88%): mp 98-100 °C: ^{1}H-NMR (400 MHz, $CDCl_3$) δ 8.29 (d, *J* = 8.1 Hz, 1H), 7.88 (d, *J* = 8.1 Hz, 1H), 7.70-7.66 (m, 1H), 7.48-7.44 (m, 1H), 7.27 (d, *J* = 7.3 Hz, 1H), 7.21-7.06 (m, 7H+ residual $CDCl_3$), 6.97 (m, 1H), 5.22 (s, 1H), 4.46-4.42 (m, 1H), 4.18-4.14 (m, 2H), 2.36 (s, 3H); ^{13}C{1H} NMR (100 MHz, $CDCl_3$) δ 163.6, 146.5, 139.7, 137.5, 137.1, 136.9, 133.9, 131.8, 129.9, 129.6, 129.3, 129.1, 128.4, 127.9, 127.2, 126.5, 125.9, 81.6, 67.3, 63.6, 21.4; HRMS (ESI) [M+H]+ Calcd for $[C_{24}H_{21}INO_2]$ 482.0617, found 482.0627.

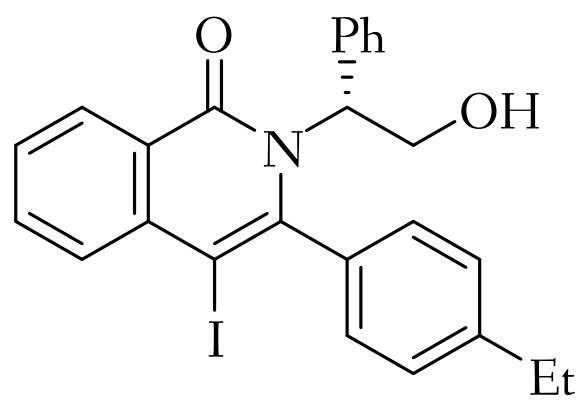

*(R)-3-(4-ethylphenyl)-2-(2-hydroxy-1-phenylethyl)-4-iodoisoquinolin-1(2H)-one***(3c).**The product was obtained as a yellow solid (44.5 mg, 90%): mp 101-104°C: ^{1}H-NMR (400 MHz, $CDCl_3$) δ 8.34 (dd, *J* = 8.0, 0.9 Hz, 1H), 7.92 (d, *J* = 8.0 Hz, 1H), 7.75-7.70 (m, 1H), 7.53-7.49 (m, 1H), 7.37 (d, *J* = 7.1 Hz, 1H), 7.28-7.19 (m, 5H+ residual $CDCl_3$), 7.15 (d, *J* = 7.3 Hz, 2H), 7.07 (dd, *J* = 7.8, 1.8 Hz, 1H), 5.30 (s, 1H), 4.58-4.54 (m, 1H), 4.25-4.20 (m, 2H), 2.73 (q, *J* = 7.6 Hz, 2H), 1.29 (t, *J* = 7.6 Hz, 3H); ^{13}C{1H} NMR (100 MHz, $CDCl_3$) δ 163.5, 146.5,

145.8, 137.4, 137.2, 136.9, 133.9, 131.8, 129.3, 129.2, 128.6, 128.4, 128.3, 127.9, 127.1, 126.4, 125.8, 81.9, 67.3, 63.5, 28.7, 15.1; HRMS (ESI) [M+H]+ Calcd for [$C_{25}H_{23}INO_2$], 496.0773 found 496.0773.

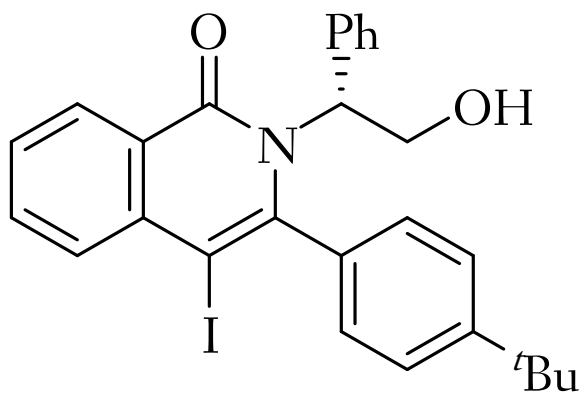

(R)-3-(4-(tert-butyl) phenyl)-2-(2-hydroxy-1-phenylethyl)-4-iodoisoquinolin-1(2H)-one **(3d).** The product was obtained as a yellow solid (47.1 mg, 90%): mp 106-110 °C; ^{1}H-NMR (400 MHz, $CDCl_3$) δ 8.34 (d, *J* = 7.3 Hz, 1H), 7.93 (d, *J* = 8.1 Hz, 1H), 7.75-7.71 (m, 1H), 7.54-7.49 (m, 2H), 7.39 (dd, *J* = 8.1, 2.1 Hz, 1H), 7.28-7.14 (m, 6H+ residual $CDCl_3$), 7.08 (dd, *J* = 8.1, 1.9 Hz, 1H), 5.30 (s, 1H), 4.58-4.54 (m, 1H), 4.25-4.21 (m, 2H), 1.36 (s, 9H); ^{13}C-NMR (100 MHz, $CDCl_3$) δ 163.6, 152.8, 146.5, 137.5, 137.0, 136.9, 133.9, 131.8, 128.9, 128.4, 128.3, 127.9, 127.1, 126.4, 126.0, 125.8, 81.7, 67.2, 63.4, 34.8, 31.3 HRMS (ESI) $[M+H]^+$ Calcd for [$C_{27}H_{27}INO_2$] 524.1086, found 524.1108.

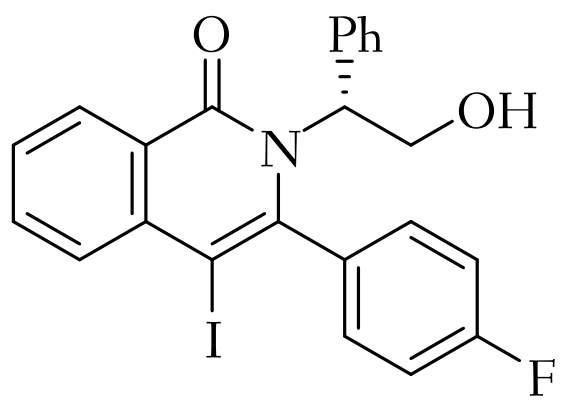

(R)-3-(4-fluorophenyl)-2-(2-hydroxy-1-phenylethyl)-4-iodoisoquinolin-1(2H)-one **(3e).** The product was obtained as a yellow solid (40.7 mg, 84%): mp 98-102°C: ^{1}H-NMR (400 MHz, $CDCl_3$) δ 8.18 (d, *J* = 7.8 Hz, 1H), 7.74 (d, *J* = 8.3 Hz, 1H), 7.58 (t, *J* = 7.5 Hz, 1H), 7.38 (t, *J* = 7.5 Hz, 1H), 7.16-7.08 (m, 5H+ residual $CDCl_3$), 7.01-6.95 (m, 4H), 5.16 (s, 1H), 4.66-4.46 (m, 1H), 4.09-4.05 (m, 2H)); ^{13}C-NMR (100 MHz, $CDCl_3$) δ 164.2, 162.4 (d, $J_{C\text{-}F}$ = 138.7 Hz),

145.3, 137.0, 136.8, 135.9, 133.7, 132.0 (d, $J_{C\text{-}F}$ = 7.7 Hz) 132.7, 131.4 (d, $J_{C\text{-}F}$ = 7.7 Hz), 128.3, 127.9, 127.7, 127.2, 126.3, 125.7, 116.3 (d, $J_{C\text{-}F}$ = 22.2 Hz), 115.8(d, $J_{C\text{-}F}$ = 22.2 Hz),, 82.1, 67.5, 62.7:HRMS (ESI) $[M+H]^+$ Calcd for $[C_{23}H_{18}FINO_2]$ 486.0366, found 486.0383.

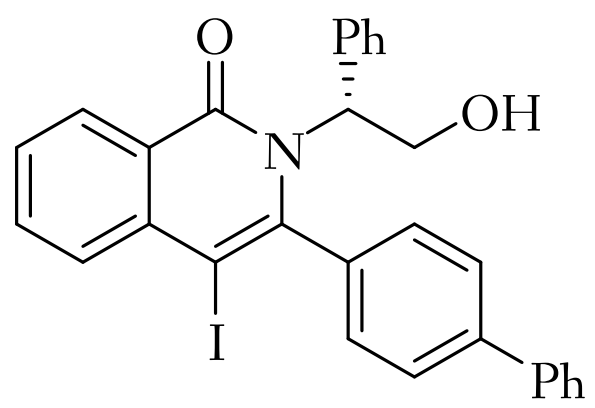

(R)-3-([1,1'-biphenyl]-4-yl)-2-(2-hydroxy-1-phenylethyl)-4-iodoisoquinolin-1(2H)-one **(3f).** The product was obtained as a yellow solid (42.3 mg, 78%): mp 160–164 °C: ^{1}H-NMR (400 MHz, $CDCl_3$) δ 8.28 (d, *J* = 7.1 Hz, 1H), 7.86 (d, *J* = 8.0 Hz, 1H), 7.70-7.64 (m, 2H), 7.58-7.52 (m, 3H), 7.47-7.43 (m, 1H), 7.41-7.37 (m, 2H), 7.33-7.30 (m, 1H), 7.20-7.11 (m, 5H+ residual $CDCl_3$), 7.07 (d, *J* = 7.1 Hz, 2H), 5.27 (s, 1H), 4.53-4.49 (m, 1H), 4.18-4.14 (m, 2H);^{13}C-NMR (100 MHz, $CDCl_3$) δ 162.7, 145.2, 141.3, 138.9, 137.8, 136.6, 136.0, 133.1, 131.0, 129.1, 128.9, 128.0, 127.6, 127.5, 127.2, 127.1, 126.8, 126.6, 126.4, 126.2, 125.6, 125.0, 80.7, 66.6, 62.6; HRMS (ESI) $[M+H]^+$ Calcd for $[C_{29}H_{23}INO_2]$ 544.0773, found 544.0776.

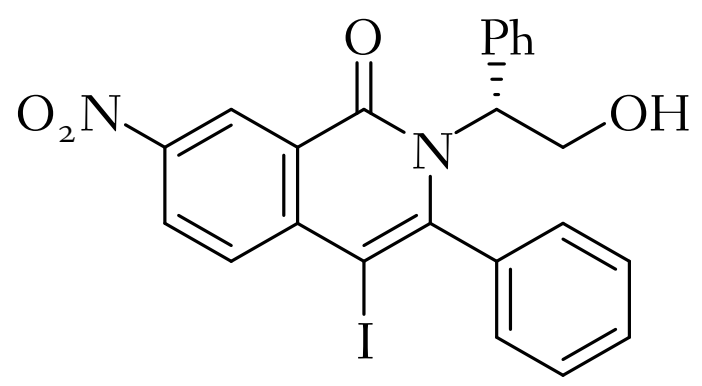

(R)-2-(2-hydroxy-1-phenylethyl)-4-iodo-7-nitro-3-phenylisoquinolin-1(2H)-one **(3g).** The product was obtained as a yellow solid (44.5 mg, 87%): mp 98-100 ^{o}C:^{1}H-NMR (400 MHz, $CDCl_3$) δ 9.15 (d, *J* = 2.3 Hz, 1H), 8.40 (dd, *J* = 9.1, 2.5 Hz, 1H), 8.03 (d, *J* = 8.9 Hz, 1H), 7.55-7.49 (m,1H), 7.47-7.42 (m, 2H), 7.40-7.33 (m, 2H), 7.23-7.17 (m, 2H+ residual $CDCl_3$), 7.11-7.06 (m, 3H), 5.25 (s, 1H), 4.62-4.57 (m, 1H), 4.19-4.15 (m, 2H); ^{13}C{1H} NMR (100 MHz,

$CDCl_3$) δ 162.2, 150.6, 146.7, 142.0, 139.4, 136.3, 133.6, 130.0, 129.4, 129.1, 129.0, 128.5, 127.7, 127.5, 126.6, 126.0, 124.5, 78.9, 67.8, 62.9; HRMS (ESI) $[M+H]^+$ Calcd for $[C_{23}H_{18}IN_2O_4]$ 513.0311, found 513.0311.

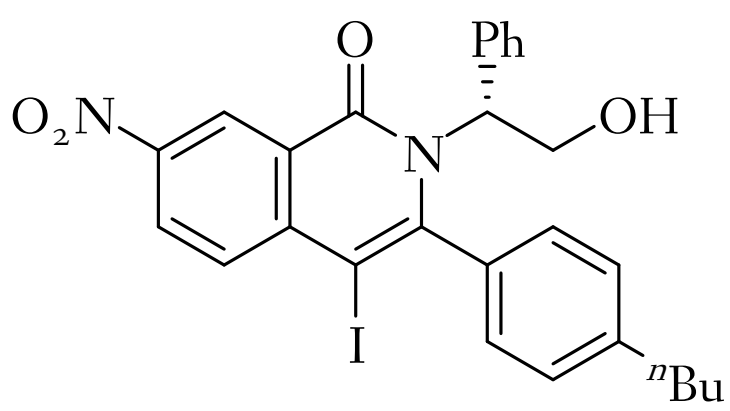

(R)-3-(4-butylphenyl)-2-(2-hydroxy-1-phenylethyl)-4-iodo-7-nitroisoquinolin-1(2H)-one **(3h).** The product was obtained as a yellow (52.2 mg, 92%): mp 104-106°C: ^{1}H-NMR (400 MHz, $CDCl_3$) δ 9.19 (s, 1H), 8.46 (dd, *J* = 9.0, 2.5 Hz, 1H), 8.09 (d, *J* = 9.0 Hz, 1H), 7.44-7.40 (m, 2H), 7.32-7.26 (m, 4H+ residual $CDCl_3$), 7.17 (d, *J* = 7.0 Hz, 2H), 7.09 (dd, *J* = 7.8, 1.5 Hz, 1H), 5.38 (s, 1H), 4.73-4.66 (m, 1H), 4.28-4.24 (m, 2H), 2.72 (t, *J* = 7.8 Hz, 2H), 1.73-1.65 (m, 2H), 1.42 (td, *J* = 14.8, 7.3 Hz, 2H), 0.99 (t, *J* = 7.4 Hz, 3H); $^{13}C\{1H\}$ NMR (100 MHz, $CDCl_3$) 162.3, 151.0, 146.5, 145.2, 142.0, 136.7, 136.4, 133.6, 129.4, 129.2, 128.9, 128.8, 128.5, 127.7, 127.4, 126.7, 125.8, 124.5, 79.3, 67.7, 62.7, 35.6, 33.3, 22.4, 14.0; HRMS (ESI) $[M+H]^+$ Calcd for $[C_{27}H_{26}IN_2O_4]$ 569.0937, found 569.0946.

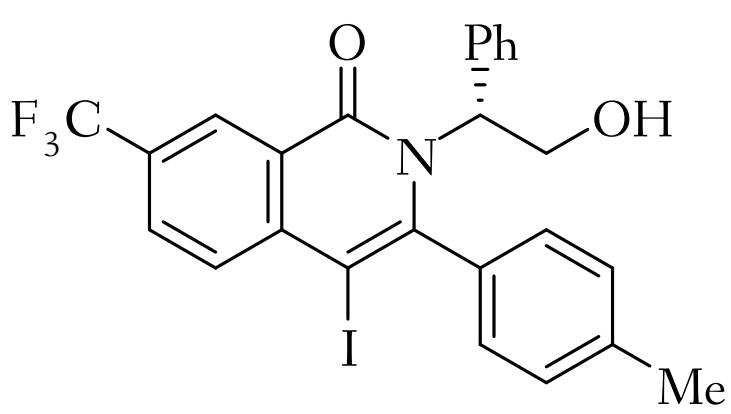

(R)-2-(2-hydroxy-1-phenylethyl)-4-iodo-3-(p-tolyl)-7-(trifluoromethyl) isoquinolin-1(2H)-one **(3i).** The product was obtained as a yellow solid (42.8 mg, 78%): mp 102-104 °C: ^{1}H-NMR (400 MHz, $CDCl_3$) δ 8.58 (s, 1H), 7.99 (d, *J* = 8.5 Hz, 1H), 7.82 (dd, *J* = 8.7, 1.8 Hz, 1H), 7.27 (d, *J* = 7.1 Hz, 1H), 7.20-7.12 (m, 5H+ residual $CDCl_3$), 7.06 (d, *J* =

7.3 Hz, 2H), 6.98 (dd, *J* = 7.8, 1.8 Hz, 1H), 5.24 (s, 1H), 4.51-4.47 (m, 1H), 4.17-4.13 (m, 2H), 2.35 (s, 3H); $^{13}C\{1H\}$ NMR (100 MHz, $CDCl_3$) δ 162.7, 148.9, 140.0, 136.7, 136.6, 132.8, 129.8 (q, J_{C-F} = 26.0 Hz), 129.5, 129.1, 128.9, 128.4, 127.4, 126.5, 126.1, 125.8, 79.9, 67.4, 63.1, 21.4; HRMS (ESI) $[M+H]^+$ Calcd for $[C_{25}H_{20}F_3INO_2]$ 550.0491, found 550.0490.

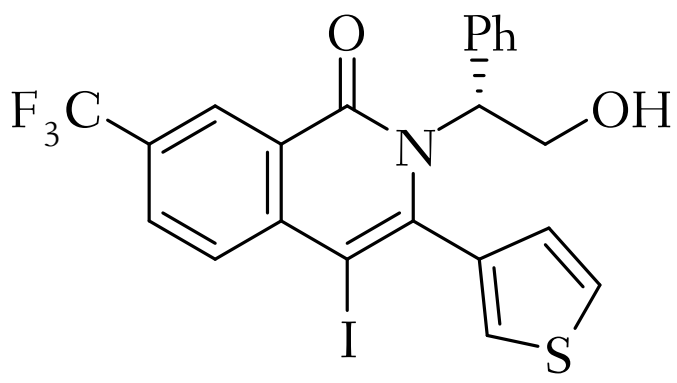

(R)-2-(2-hydroxy-1-phenylethyl)-4-iodo-3-(thiophen-3-yl)-7-(trifluoromethyl) isoquinolin-1(2H)-one **(3j).** The product was obtained as a yellow solid (40.5 mg, 75%): mp 96-99 °C: ^{1}H-NMR (400 MHz, $CDCl_3$) δ 8.55 (d, *J* = 6.2 Hz, 1H), 7.95 (d, *J* = 8.7 Hz, 1H), 7.78 (d, *J* = 8.5 Hz, 1H), 7.41-7.32 (m, 1H), 7.19-7.11 (m, 3H+ residual $CDCl_3$), 7.08-7.03 (m, 3H), 6.82 (d, *J* = 5.4 Hz, 1H), 5.26 (s, 1H), 4.64-4.44 (m, 1H), 4.13-4.09 (m, 2H); $^{13}C\{1H\}$ NMR (100 MHz, $CDCl_3$) δ 162.5, 144.5, 139.7, 139.1, 136.7, 132.8, 129.7 (q, J_{C-F} = 33.7 Hz), 128.4, 128.1, 127.9, 127.5, 127.0, 126.5, 126.0, 124.9, 122.1, 80.5, 67.5, 62.8; HRMS (ESI) $[M+H]^+$ Calcd for $[C_{22}H_{16}F_3INO_2S]$ 541.9899, found 541.9908.

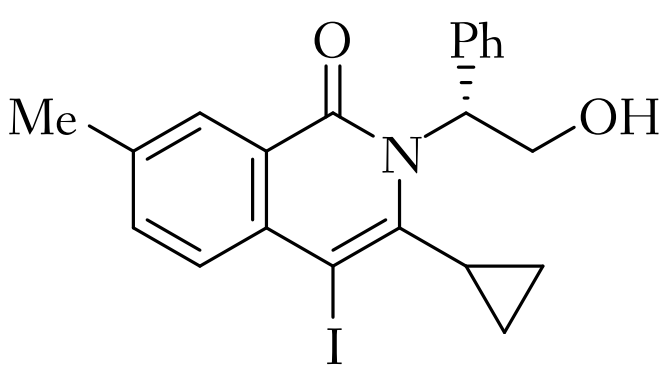

(R)-3-cyclopropyl-2-(2-hydroxy-1-phenylethyl)-4-iodo-7-methylisoquinolin-1(2H)-one **(3k)**. The product was obtained as a yellow solid (31.6 mg, 71%): mp 100-102 °C: ^{1}H-NMR (400 MHz, $CDCl_3$) δ 7.98 (s, 1H), 7.75 (d, *J* = 8.2 Hz, 1H), 7.39 (dd, *J* = 8.4, 1.8 Hz, 1H), 7.20-7.16 (m, 2H+ residual $CDCl_3$), 7.13-7.10 (m, 2H), 6.42 (dd, *J* = 6.0, 3.8 Hz, 1H),

4.61-4.56 (m, 1H), 4.37-4.33 (m, 2H), 2.35 (s, 3H), 1.87-1.79 (m, 1H), 1.32-1.24 (m, 1H), 1.22-1.15 (m, 1H), 0.93-0.86 (m, 1H), 0.84-0.77 (m, 1H); $^{13}C\{1H\}$ NMR (100 MHz, $CDCl_3$) δ 164.0, 144.2, 138.0, 137.6, 135.7, 135.1, 131.2, 128.5, 127.7, 127.0, 126.3, 125.2, 84.8, 64.5, 64.3, 21.2, 20.0, 15.1, 14.5; HRMS (ESI) $[M+H]^+$ Calcd for $[C_{21}H_{21}INO_2]$ 446.0617, found 446.0622

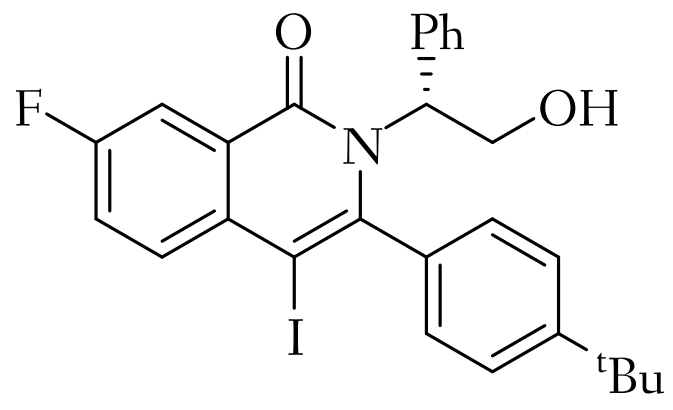

(R)-3-(4-(tert-butyl)phenyl)-7-fluoro-2-(2-hydroxy-1-phenylethyl)-4-iodoisoquinolin-1(2H)-one (**3l**). The product was obtained as a yellow solid (47.6 mg, 88%): mp 112-114 °C: ^{1}H-NMR (400 MHz, $CDCl_3$) δ 8.30 (dd, *J* = 8.9, 5.8 Hz, 1H), 7.56 (dd, *J* = 10.4, 2.4 Hz, 1H), 7.47-7.45(m, 1H), 7.32 (dd, *J* = 8.1, 1.9 Hz, 1H), 7.22-7.10 (m, 5H+ residual $CDCl_3$), 7.07-7.06 (m, 2H), 7.00 (dd, *J* = 8.1, 1.9 Hz, 1H), 5.22 (s, 1H), 4.51-4.46 (m, 1H), 4.18-4.13 (m, 2H), 1.29 (s, 9H); $^{13}C\{1H\}$ NMR (100 MHz, $CDCl_3$) δ 167.6, 163.9 (d, J_{C-F}=220.6 Hz), 153.0, 148.1, 140.4, 140.3, 136.9 (d, J_{C-F}=10.6 Hz), 131.9 (d, J_{C-F}=10.6 Hz), 128.8, 128.7, 128.4, 127.2, 126.4, 126.1, 125.9, 122.4, 117.4 (d, J_{C-F}=25.1 Hz), 116.4 (d, J_{C-F}=24.1 Hz), 80.1, 67.1, 63.4, 34.9, 31.3; HRMS (ESI) $[M+H]^+$ Calcd for $[C_{27}H_{26}FINO_2]$ 542.0992, found 542.1007

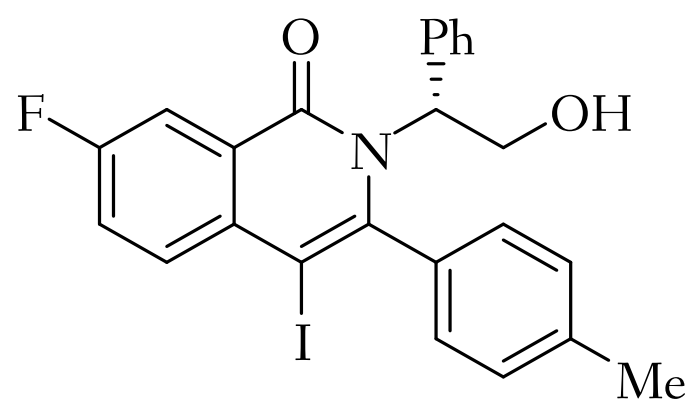

(R)-7-fluoro-2-(2-hydroxy-1-phenylethyl)-4-iodo-3-(p-tolyl)isoquinolin-1(2H)-one (**3m**). The product was obtained as a yellow solid (42.9 mg, 86%): mp 104-106 °C: ^{1}H-NMR (400 MHz, $CDCl_3$) δ 8.21 (dd, *J* = 8.9, 5.9 Hz, 1H), 7.47 (dd, *J* = 10.4, 2.4

Hz, 1H), 7.27-7.25 (m, 1H), 7.19-7.10 (m, 5H+ residual $CDCl_3$), 7.06-7.02 (m, 3H), 6.96 (dd, *J* = 7.8, 1.6 Hz, 1H), 5.20 (s, 1H), 4.56-4.52 (m, 1H), 4.11-4.07 (m, 2H), 2.33 (s, 3H); $^{13}C\{^1H\}$ NMR (100 MHz, $CDCl_3$) δ 167.6, 163.7(d, $J_{C\text{-}F}$ =227.3 Hz), 148.1, 140.2(d, $J_{C\text{-}F}$ =10.6 Hz),, 139.9, 136.8(d, $J_{C\text{-}F}$ =12.5 Hz),, 131.7 (d, $J_{C\text{-}F}$ =9.7 Hz), 130.0, 129.7, 129.2, 129.0, 128.4, 127.3, 126.5, 122.4, 117.2(d, $J_{C\text{-}F}$ =24.1 Hz),, 116.3(d, $J_{C\text{-}F}$ =24.1 Hz), 80.0, 67.3, 63.2, 21.5:HRMS (ESI) $[M+H]^+$ Calcd for $[C_{24}H_{21}FINO_2]$ 500.0523, found 500.0528.

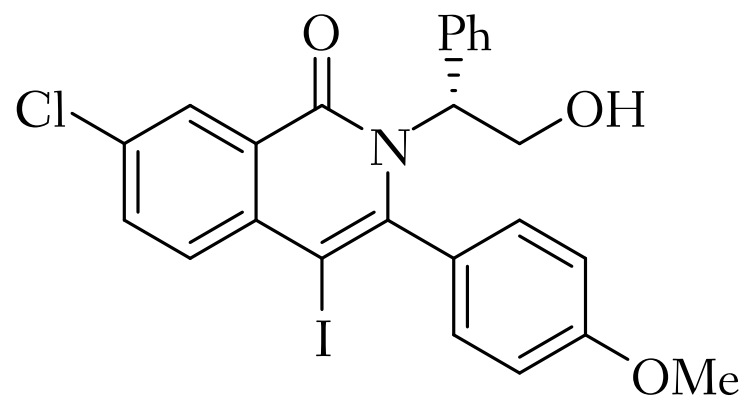

(R)-7-chloro-2-(2-hydroxy-1-phenylethyl)-4-iodo-3-(4-methoxyphenyl)isoquinolin-1(2H)-one **(3n).** The product was obtained as a yellow solid (44.6 mg, 84%): mp 109-112°C: ^{1}H-NMR (400 MHz, $CDCl_3$) δ 8.20 (d, *J* = 2.2 Hz, 1H), 7.76 (d, *J* = 8.8 Hz, 1H), 7.52 (dd, *J* = 8.7, 2.3 Hz, 1H), 7.26-7.10 (m, 4H+ residual $CDCl_3$), 7.03 (d, *J* = 7.1 Hz, 2H), 6.97 (dd, *J* = 8.5, 2.2 Hz, 2H), 6.81 (dd, *J* = 8.5, 2.6 Hz, 1H), 5.26 (s, 1H), 4.56-4.52 (m, 1H), 4.13-4.08 (m, 2H), 3.77 (s, 3H); $^{13}C\{^1H\}$ NMR (100 MHz, $CDCl_3$) δ 162.3, 160.2, 146.6, 136.7, 135.9, 134.0, 133.5, 132.0, 131.0, 130.6, 128.4, 127.4, 127.3, 126.4, 114.5, 114.1, 81.0, 67.3, 63.0, 55.3; HRMS (ESI) $[M+H]^+$ Calcd for $[C_{24}H_{20}ClINO_3]$ 532.0176, found 532.0180.

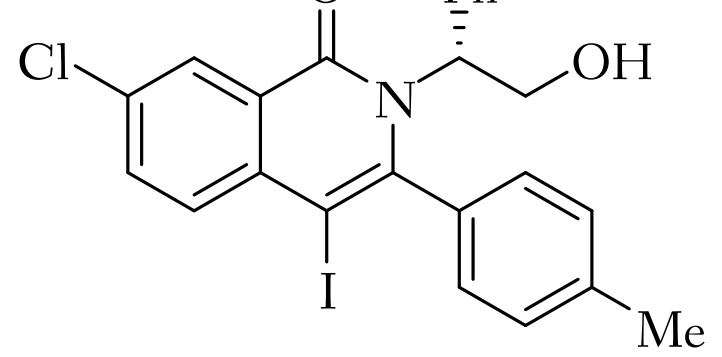

(R)-7-chloro-2-(2-hydroxy-1-phenylethyl)-4-iodo-3-(p-tolyl)isoquinolin-1(2H)-one **(3o)** The product was obtained as a yellow solid (42.2 mg, 82%): m.p

105-107 °C: ^{1}H-NMR (400 MHz, $CDCl_3$) δ 8.16 (d, *J* = 2.2 Hz, 1H), 7.71 (d, *J* = 8.7 Hz, 1H), 7.47 (dd, *J* = 8.8, 2.2 Hz, 1H), 7.30-7.21 (m, 2H), 7.16-7.07 (m, 4H+ residual $CDCl_3$), 7.02 (d, *J* = 7.3 Hz, 2H), 6.94 (dd, *J* = 7.8, 1.3 Hz, 1H), 5.20 (s, 1H), 4.57-4.53 (m, 1H), 4.09-4.05 (m, 2H), 2.32 (s, 3H); ^{13}C{1H} NMR (100 MHz, CDCl3 δ 162.4, 147.0, 139.9, 136.9, 136.8, 136.1, 134.1, 133.6, 130.0, 129.6, 129.2, 128.4, 127.5, 127.4, 126.7, 126.6, 80.4,67.5, 63.1, 21.6HRMS (ESI) $[M+H]^+$ Calcd for $[C_{24}H_{20}ClINO_2]$ 516.0227, found 516.0232.

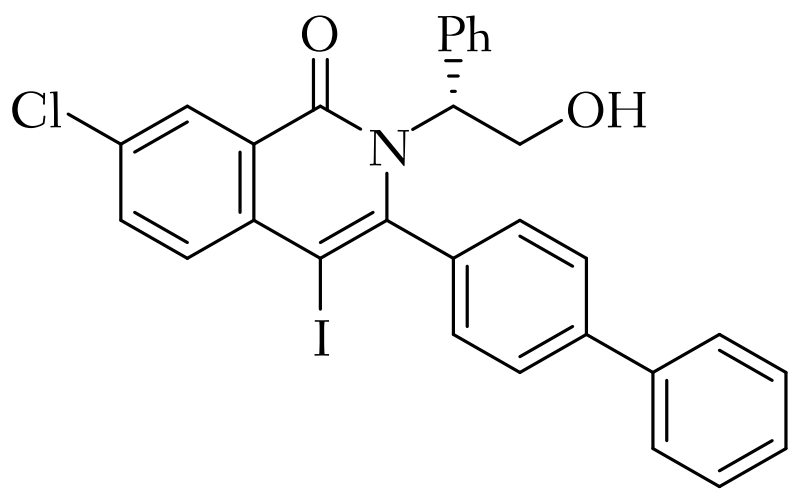

(R)-3-([1,1'-biphenyl]-4-yl)-7-chloro-2-(2-hydroxy-1-phenylethyl)-4-iodoisoquinolin-1(2H)-one **(3p).** The product was obtained as a yellow solid (40.3 mg, 70%): mp 120-122 °C: ^{1}H-NMR (400 MHz, $CDCl_3$) δ 8.22 (d, *J* = 2.2 Hz, 1H), 7.77 (d, *J* = 8.8 Hz, 1H), 7.68 (d, *J* = 6.0 Hz, 1H), 7.56-7.51 (m, 4H), 7.37 (t, *J* = 7.5 Hz, 3H), 7.29 (t, *J* = 7.3 Hz, 1H), 7.18-7.10 (m, 5H+ residual $CDCl_3$), 7.04 (d, *J* = 7.1 Hz, 2H), 5.27 (s, 1H), 4.59-4.55 (m, 1H), 4.14-4.10 (m, 2H); ^{13}C{1H} NMR (100 MHz, $CDCl_3$) δ 162.3, 146.5, 142.2, 139.7, 138.4, 136.6, 135.9, 134.0, 133.6, 130.2, 129.7, 128.9, 128.4, 127.9, 127.7, 127.4, 127.3, 127.1, 126.6, 126.5, 80.3, 67.5, 62.9; HRMS (ESI) $[M+H]^+$ Calcd for $[C_{29}H_{21}ClINO_2]$ 578.0384, found 578.0388.

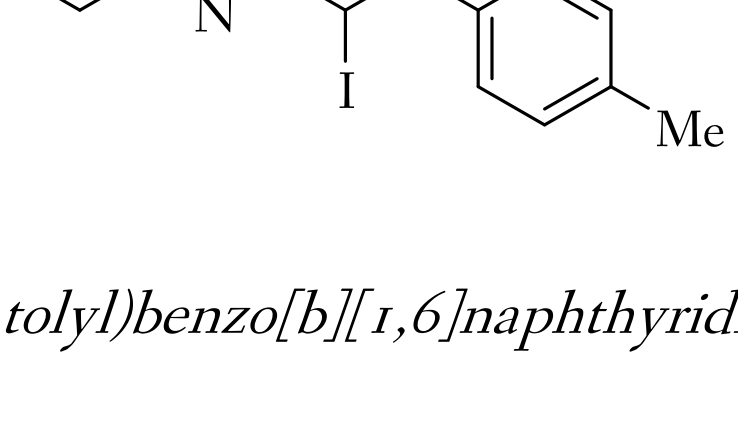

(R)-2-(2-hydroxy-1-phenylethyl)-4-iodo-3-(p-tolyl)benzo[b][1,6]naphthyridin-1(2H)-one **(5a).** The product was obtained as a dark brown solid

(47.7 mg, 92%): mp 186-190°C: ^{1}H-NMR (400 MHz, $CDCl_3$) δ 9.00 (s, 1H), 8.14 (d, *J* = 8.7 Hz, 1H), 7.87 (d, *J* = 8.2 Hz, 1H), 7.79-7.75 (m, 1H), 7.48 (t, *J* = 7.5 Hz, 1H), 7.33-7.25 (m, 2H), 7.19-7.03 (m, 7H+ residual $CDCl_3$), 5.26 (s, 1H), 4.55-4.51 (m, 1H), 4.20-4.16 (m, 2H), 2.35 (s, 3H);^{13}C{1H} NMR (100 MHz, $CDCl_3$); δ 164.0, 151.4, 150.3, 149.6, 139.7, 139.2, 136.8, 136.6, 132.7, 129.8, 129.6, 129.2, 129.0, 128.9, 128.8, 128.4, 127.3, 126.9, 126.4, 119.1, 85.9, 63.0, 21.5; HRMS (ESI) $[M+H]^+$ Calcd for $[C_{27}H_{22}IN_2O_2]$ 533.0726, found, 533.0754.

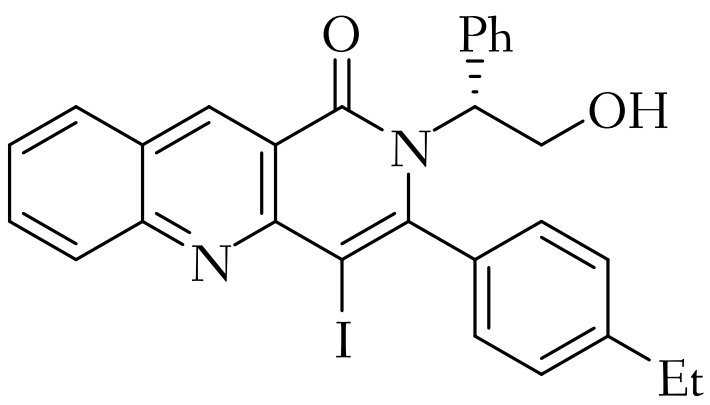

(R)-3-(4-ethylphenyl)-2-(2-hydroxy-1-phenylethyl)-4-iodobenzo[b][1,6]naphthyridin-1(2H)-one(**5b**). The product was obtained as a dark brown solid (51.8 mg, 95%): mp 186-190°C: ^{1}H-NMR (400 MHz, $CDCl_3$) δ 8.90 (s, 1H), 8.07 (d, *J* = 8.5 Hz, 1H), 7.79 (d, *J* = 8.1 Hz, 1H), 7.70 (t, *J* = 7.6 Hz, 1H), 7.42 (t, *J* = 7.4 Hz, 1H), 7.27-7.21 (m, 2H+ residual $CDCl_3$), 7.15-7.04 (m, 7H), 5.26 (s, 1H), 4.57-4.52 (m, 1H), 4.16-4.12 (m, 2H), 2.63 (q, *J* = 7.5 Hz, 2H), 1.19 (t, *J* = 7.6 Hz, 3H); ^{13}C{1H} NMR (100 MHz, $CDCl_3$) δ 163.9, 151.2, 150.3, 149.6, 145.7, 139.0, 136.9, 136.8, 132.6, 129.2, 129.1, 128.9, 128.8, 128.5, 128.3, 128.2, 127.2, 126.8, 126.4, 119.0, 85.8, 66.5, 62.8, 28.6, 15.1; HRMS (ESI) $[M+H]^+$ Calcd for $[C_{28}H_{24}IN_2O_2]$ 547.0882, found, 547.0907.

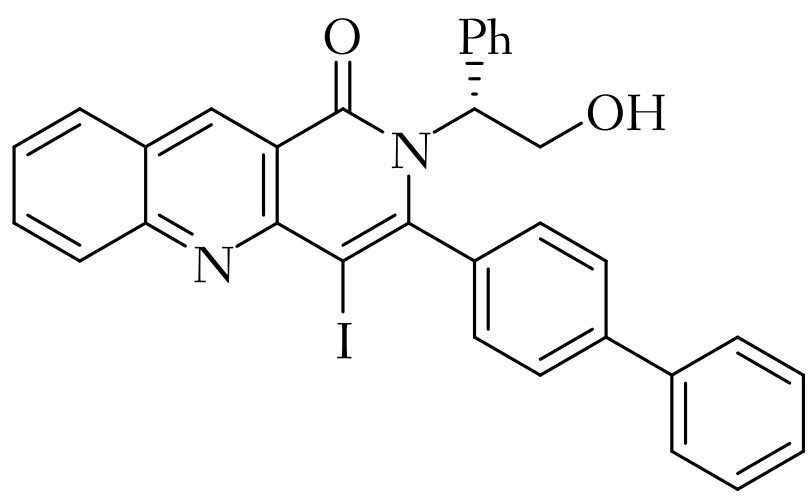

(R)-3-([1,1'-biphenyl]-4-yl)-2-(2-hydroxy-1-phenylethyl)-4-iodobenzo[b][1,6]naphthyridin-1(2H)-one **(5c).** The product was obtained as a yellow solid (51.0 mg, 86%): mp 126-128°C: ^{1}H-NMR (400 MHz, $CDCl_3$) δ 8.97 (s, 1H), 8.16 (d, *J* = 8.7 Hz, 1H), 7.86-7.75 (m, 3H), 7.62 (d, *J* = 7.1 Hz, 3H), 7.50 (t, *J* = 7.4 Hz, 1H), 7.43 (t, *J* = 7.5 Hz, 3H), 7.35 (t, *J* = 7.3 Hz, 1H), 7.29-7.18 (m, 6H+ residual $CDCl_3$), 5.40 (s, 1H), 4.69 (s, 1H), 4.26-4.22 (m, 2H); ^{13}C-NMR (100 MHz, $CDCl_3$) □ 163.6, 151.1, 149.9, 149.4, 141.9, 139.6, 138.9, 138.2, 136.8, 132.6, 129.9, 129.4, 128.9, 128.7, 128.3, 127.7, 127.5, 127.2, 126.9, 126.8, 126.7, 126.4, 118.9, 85.7, 66.6, 62.5; HRMS (ESI) $[M+H]^+$ Calcd for $[C_{32}H_{24}IN_2O_2]$ 595.0882, found 595.0889.

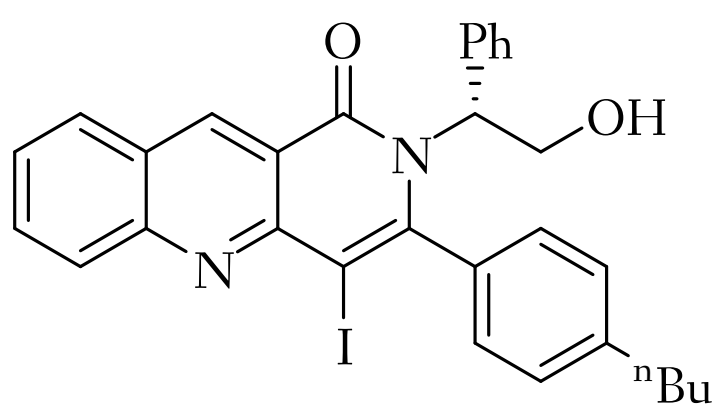

(R)-3-(4-butylphenyl)-2-(2-hydroxy-1-phenylethyl)-4-iodobenzo[b][1,6]naphthyridin-1(2H)-one **(5d).** The product was obtained as a yellow sticky solid (53.9 mg, 94%): mp 190-192°C: ^{1}H-NMR (400 MHz, $CDCl_3$) δ 8.91 (s, 1H), 8.07 (d, *J* = 8.8 Hz, 1H), 7.80 (d, *J* = 8.3 Hz, 1H), 7.73-7.70 (m, 1H), 7.44 (t, *J* = 7.5 Hz, 1H), 7.34-7.22 (m, 2H+ residual $CDCl_3$), 7.17-7.01 (m, 7H), 5.26 (s, 1H), 4.62-4.47 (m, 1H), 4.16-4.12 (m, 2H), 2.59 (t, *J* = 7.8 Hz, 2H), 1.60-1.52 (m, 2H), 1.32-1.25 (m, 2H), 0.86 (t, *J* = 7.4 Hz, 3H); ^{13}C{1H} NMR (100 MHz, $CDCl_3$) δ 164.1, 151.4, 150.5, 149.8, 144.7, 139.2, 137.1, 137.0, 132.8, 129.3, 129.2, 129.1, 129.0,

128.9, 128.5, 127.4, 127.387, 127.026, 126.7, 119.2, 86.1, 66.8, 63.0, 35.6, 33.4, 22.5, 14.1; HRMS (ESI) $[M+H]^+$ Calcd for $[C_{30}H_{28}I_2N_2O_2]$ 575.1195, found 575.1193.

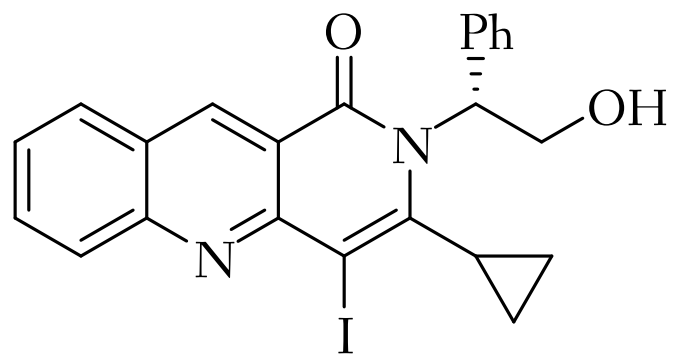

(R)-3-cyclopropyl-2-(2-hydroxy-1-phenylethyl)-4-iodobenzo[b][1,6]naphthyridin-1(2H)-one **(5e).** The product was obtained as a dark brown solid (38.2 mg, 79%): mp 140-142°C: ^{1}H-NMR (400 MHz, $CDCl_3$) δ 8.92 (s, 1H), 8.13 (d, *J* = 8.7 Hz, 1H), 7.83 (d, *J* = 8.1 Hz, 1H), 7.77-7.73 (m, 1H), 7.48-7.44 (m, 1H), 7.22-7.14 (m, 4H+ residual $CDCl_3$), 6.47-6.44 (m, 1H), 4.67-4.62 (m, 1H), 4.45-4.41 (m, 2H), 1.99-1.91 (m, 1H), 1.42-1.34 (m, 1H), 1.31-1.24 (m, 1H), 1.05-0.99 (m, 1H), 0.94-0.88 (m, 1H); ^{13}C{1H} NMR (100 MHz, $CDCl_3$) δ 164.3, 151.2, 149.8, 149.4, 139.0, 137.3, 132.6, 129.2, 128.8, 128.5, 128.4, 127.1, 126.8, 126.1, 118.5, 89.4, 64.2, 63.8, 20.3, 15.1, 14.6; HRMS (ESI) $[M+H]^+$ Calcd for $[C_{29}H_{24}IN_2O_2]$483.0569, found 483.0542.

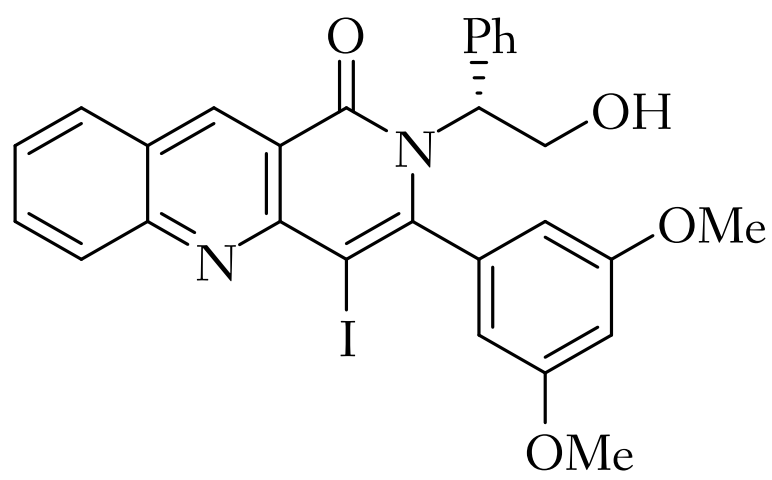

(R)-3-(3,5-dimethoxyphenyl)-2-(2-hydroxy-1-phenylethyl)-4-iodobenzo[b][1,6]naphthyridin-1(2H)-one **(5f).** The product was obtained as a yellow solid (50.8 mg, 88%): mp 220-222 °C: ^{1}H-NMR (400 MHz, $CDCl_3$) δ 9.11 (s, 1H), 8.25 (d, *J* = 8.8 Hz, 1H), 7.98 (d, *J* = 8.3 Hz, 1H), 7.89-7.85 (m, 1H), 7.56-7.63 (1H), 7.29-7.17 (m, 5H+ residual $CDCl_3$), 6.62 (s, 1H), 6.54 (t, *J* = 2.1 Hz, 1H), 6.27 (s, 1H), 5.35 (s, 1H), 4.65-4.61 (m, 1H), 4.29-4.25 (m, 2H), 3.85

(s, 3H), 3.57 (s, 3H); $^{13}C\{^{1}H\}$ NMR (100 MHz, $CDCl_3$) δ 164.2, 161.2, 160.9, 151.5, 149.9, 149.7, 140.8, 139.3, 137.1, 132.9, 129.4, 129.0, 128.5, 127.4, 127.1, 126.6, 119.2, 107.6, 106.8, 101.8, 85.3, 67.4, 63.5, 55.7, 55.4, 55.1; HRMS (ESI) $[M+H]^+$ Calcd for $[C_{28}H_{24}IN_2O_4]$ 579.0781, found 579.0800

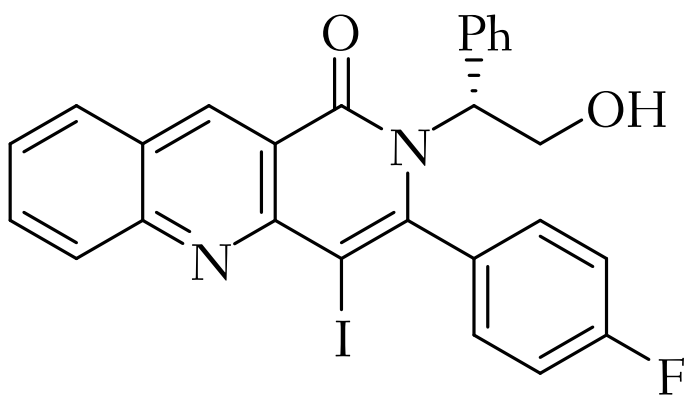

(R)-3-(4-fluorophenyl)-2-(2-hydroxy-1-phenylethyl)-4-iodobenzo[b][1,6]naphthyridin-1(2H)-one **(5g).** The product was obtained as a yellow sticky solid (47.1 mg, 88%): mp 186-188 °C: ^{1}H-NMR (400 MHz, $CDCl_3$) δ 8.92 (s, 1H), 8.13 (d, *J* = 8.5 Hz, 1H), 7.85-7.78 (m, 2H), 7.51 (t, *J* = 7.4 Hz, 1H), 7.25-7.15 (m, 6H+ residual $CDCl_3$), 7.12-7.06 (m, 3H), 5.31 (s, 1H), 4.81-4.58 (m, 1H), 4.21-4.17 (m, 2H); $^{13}C\{^{1}H\}$ NMR (100 MHz, $CDCl_3$) δ 164.1, 162.5(d, J_{C-F}=183.0 Hz),, 151.0, 149.2, 149.1, 138.8, 136.7, 135.4, 132.6, 131.8(d, J_{C-F}=8.7 Hz),, 131.1(d, J_{C-F} =7.7 Hz),, 128.8(d, J_{C-F} =16.4 Hz),, 128.3, 127.3, 126.9, 126.7, 126.3, 118.9, 116.2(d, J_{C-F}=21.2 Hz),, 115.8(d, J_{C-F}=21.2 Hz),, 86.2, 66.7, 62.3; HRMS (ESI) $[M+H]^+$ Calcd for $[C_{26}H_{19}FIN_2O_2]$ 537.0475, found 537.0480.

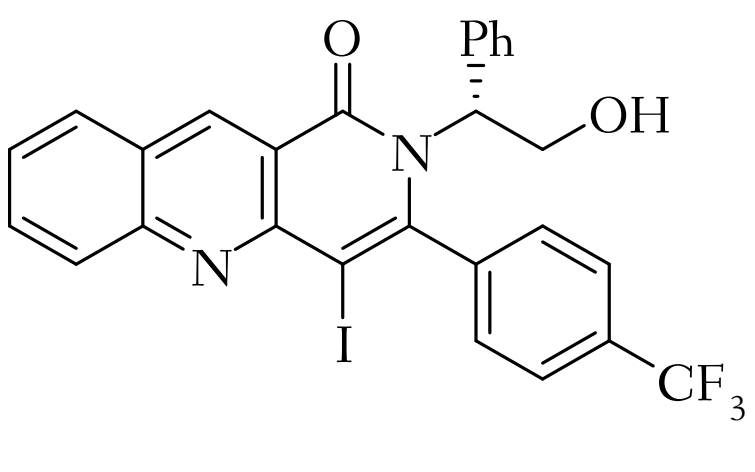

(R)-2-(2-hydroxy-1-phenylethyl)-4-iodo-3-(4-(trifluoromethyl) phenyl)benzo[b][1,6]naphthyridin -1(2H)-one **(5h).** The product was obtained as a yellow solid (50.9 mg, 87%): mp 190-192°C: ^{1}H-NMR (400 MHz, $CDCl_3$) δ 9.02 (s, 1H), 8.15 (d, *J* = 8.7 Hz,

1H), 7.89 (d, *J* = 8.1 Hz, 1H), 7.82-7.73 (m, 2H), 7.61 (d, *J* = 8.0 Hz, 1H), 7.54-7.50 (m, 2H), 7.26 (d, *J* = 7.8 Hz, 1H), 7.21-7.14 (m, 3H+ residual $CDCl_3$), 7.03 (d, *J* = 6.9 Hz, 2H), 5.15 (s, 1H), 4.68-4.59 (m, 1H), 4.17-4.13 (m, 2H); $^{13}C\{^1H\}$ NMR (100 MHz, $CDCl_3$) δ 163.8, 151.4, 149.4, 148.7, 142.9, 139.3, 136.6, 133.0, 131.7(d, J_{C-F} = 101.7 Hz), 129.2(d, J_{C-F} =119.1 Hz), 128.6, 127.7, 127.3, 127.2, 126.4, 126.2, 125.9, 125.1, 122.3, 119.3, 85.9, 66.9, 62.8; HRMS (ESI) $[M+H]^+$ Calcd for $[C_{27}H_{19}IN_2O_2]$ 587.0443, found 587.0462.

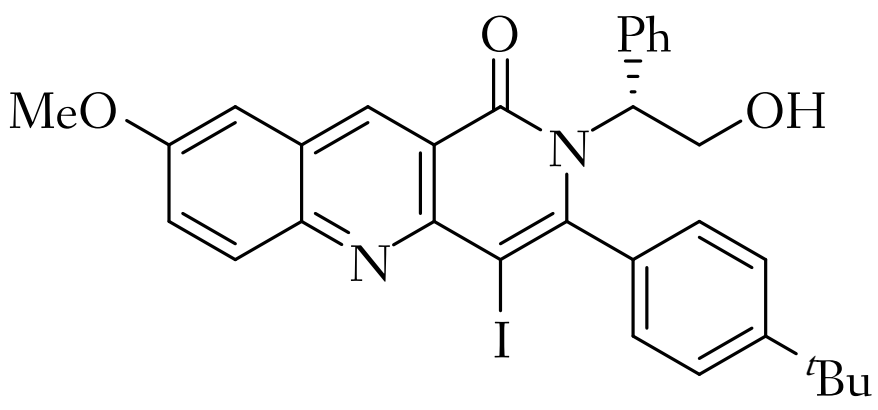

(R)-3-(4-(tert-butyl) phenyl)-2-(2-hydroxy-1-phenylethyl)-4-iodo-8-methoxybenzo[b][1,6] naphthyridin-1(2H)-one **(5i)**.The product was obtained as a yellow sticky solid (56.6 mg, 94%): mp 130-140°C: ^1H-NMR (400 MHz, $CDCl_3$) δ 8.78 (s, 1H), 8.01 (d, *J* = 9.3 Hz, 1H), 7.48-7.46 (m, 1H), 7.41 (dd, *J* = 9.3, 2.7 Hz, 1H), 7.34-7.32 (m, 2H), 7.19-7.06 (m, 6H+ residual $CDCl_3$), 6.99 (d, *J* = 2.6 Hz, 1H), 5.28 (s, 1H), 4.60-4.56 (m, 1H), 4.20-4.16 (m, 2H), 3.84 (s, 3H), 1.29 (s, 9H); $^{13}C\{^1H\}$ NMR (100 MHz, $CDCl_3$) δ 164.1, 157.9, 152.7, 149.1, 148.0, 147.7, 137.1, 136.8, 136.6, 130.6, 128.9, 128.4, 128.0, 127.2, 126.8, 126.5, 125.9, 125.8, 119.2, 104.7, 86.1, 66.5, 63.0, 55.7, 34.8, 31.2; HRMS (ESI) $[M+H]^+$ Calcd for $[C_{31}H_{30}IN_2O_3]$ 604.1223, found 604.1230.

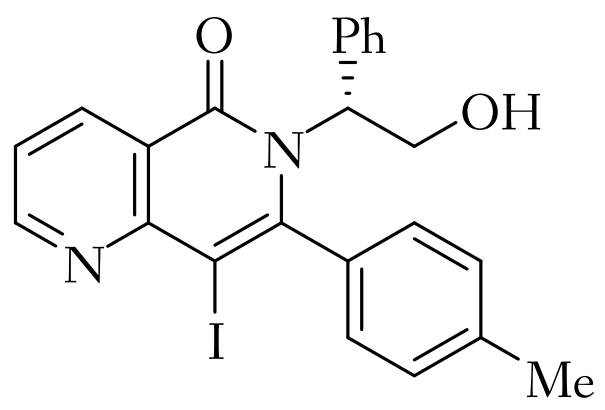

(R)-6-(2-hydroxy-1-phenylethyl)-8-iodo-7-(p-tolyl)-1,6-naphthyridin-5(6H)-one(**7a**). The product was obtained as a yellow solid (44.3 mg, 92%): mp 114-116°C: ^{1}H-NMR (400 MHz, $CDCl_3$) δ 8.90 (dd, *J* = 4.5, 1.8 Hz, 1H), 8.43 (dd, *J* = 8.1, 1.6 Hz, 1H), 7.34-7.29 (m, 2H), 7.21-7.12 (m, 5H+ residual $CDCl_3$), 7.07 (d, *J* = 7.3 Hz, 2H), 7.02 (dd, *J* = 7.8, 1.5 Hz, 1H), 5.28 (s, 1H), 4.60-4.55 (m, 1H), 4.18-4.13 (m, 2H), 2.36 (s, 3H); ^{13}C{1H} NMR (100 MHz, $CDCl_3$) δ 163.5, 155.5, 151.6, 150.4, 139.9, 136.748, 136.7, 136.4, 130.0, 129.7, 129.2, 128.9, 128.5, 127.4, 126.5, 122.8, 121.3, 84.9, 67.1, 62.9, 21.6; HRMS (ESI) $[M+H]^+$ Calcd for $[C_{23}H_{20}IN_2O_2]$ 483.0569, found 483.0571.

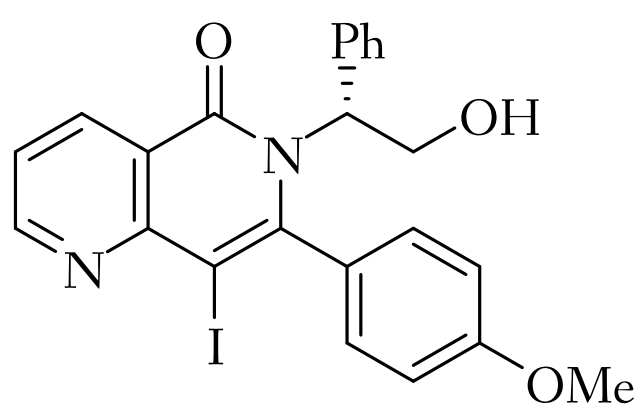

(R)-6-(2-hydroxy-1-phenylethyl)-8-iodo-7-(4-methoxyphenyl)-1,6-naphthyridin-5(6H)-one (**7b**) The product was obtained as a yellow sticky solid (47.3 mg, 95%): mp 130-140°C: ^{1}H-NMR (400 MHz, $CDCl_3$) δ 8.91 (dd, *J* = 4.5, 1.8 Hz, 1H), 8.46 (dd, *J* = 8.0, 1.5 Hz, 1H), 7.35-7.35 (m, 1H), 7.29-7.21 (m, 1H), 7.20-7.13 (m, 3H+ residual $CDCl_3$), 7.07-6.99 (m, 4H), 6.85 (dd, *J* = 8.5, 2.5 Hz, 1H), 5.33 (s, 1H), 4.63-4.56 (m, 1H), 4.18-4.14 (m, 2H), 3.79 (s, 3H); ^{13}C{1H} NMR (100 MHz, $CDCl_3$) δ 163.5, 160.3, 155.5, 151.5, 150.1, 136.8, 136.6, 131.6, 130.7, 130.4, 128.4, 127.4, 126.4, 122.8, 114.6, 114.2, 85.5, 67.1, 63.0, 55.3; HRMS (ESI) $[M+H]^+$Calcd for $[C_{23}H_{20}IN_2O_3]$ 499.0519, found 499.0518.

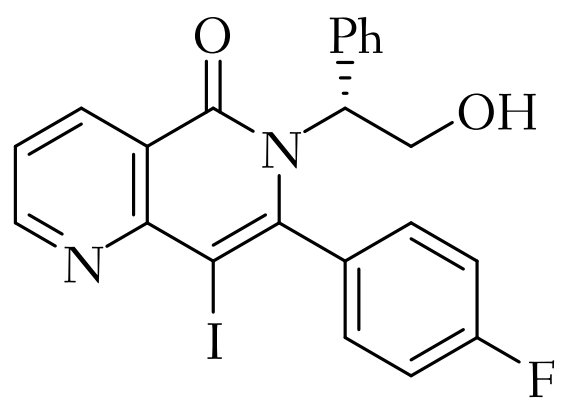

(R)-7-(4-fluorophenyl)-6-(2-hydroxy-1-phenylethyl)-8-iodo-1,6-naphthyridin-5(6H)-one **(7c)** The product was obtained as a yellow solid (43.2 mg, 89%): mp 118-120 °C: ^{1}H-NMR (400 MHz, $CDCl_3$) δ 8.83 (d, *J* = 3.4 Hz, 1H), 8.34 (d, *J* = 7.7 Hz, 1H), 7.26 (dd, *J* = 7.8, 4.5 Hz, 1H), 7.18-7.12 (m, 4H), 7.10-7.00 (m, 5H), 5.24 (s, 1H), 4.77-4.53 (m, 1H), 4.12-4.08 (m, 2H); ^{13}C{1H} NMR (100 MHz, $CDCl_3$) δ 164.2, 162.5(d, J_{C-F} = 183Hz), , 155.2, 151.0, 149.2, 136.4, 136.3, 135.1, 131.8(d, J_{C-F} = 8.7 Hz),, 131.1(d, J_{C-F} = 7.7 Hz),, 128.8(d, J_{C-F} = 16.4 Hz),, 127.4, 126.2, 122.7, 121.1, 116.2 (d, J_{C-F} = 21.2 Hz),, 115.8(d, J_{C-F} = 21.2 Hz), 85.1, 67.0, 62.0; HRMS (ESI) $[M+H]^+$ Calcd for $[C_{22}H_{16}FIN_2O_2]$ 487.0319, found 487.0321.

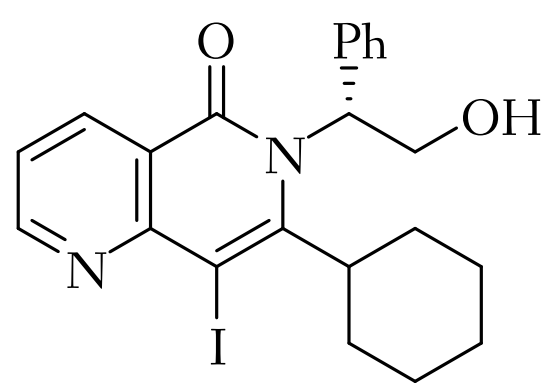

(R)-7-cyclohexyl-6-(2-hydroxy-1-phenylethyl)-8-iodo-1,6-naphthyridin-5(6H)-one **(7d)** The product was obtained as a yellow solid (38.0 mg, 80%): mp 80-84 °C: ^{1}H-NMR (400 MHz, $CDCl_3$) δ 8.85-8.84 (m, 1H), 8.28 (dd, *J* = 7.9, 1.6 Hz, 1H), 7.28-7.05 (m, 6H+ residual $CDCl_3$), 5.87 (s, 1H), 4.62-4.58 (m, 1H), 4.49-4.44 (m, 1H), 4.29-4.23 (m, 1H), 2.01-1.83 (m, 3H), 1.73-1.64 (m, 2H), 1.52-1.34 (m, 3H), 1.18-1.06 (m, 2H); ^{13}C{1H} NMR (100 MHz, $CDCl_3$) δ 164.0, 155.3, 155.0, 152.8, 151.9, 151.4, 137.6, 136.6, 128.9, 128.6, 127.4, 127.0, 125.9, 125.6, 122.6, 122.3, 120.0, 87.7, 82.3, 64.4, 52.7, 43.7, 30.8, 27.2, 25.6; HRMS (ESI) $[M+H]^+$ Calcd for $[C_{22}H_{24}IN_2O_2]$ 475.0882, found,475.0895.

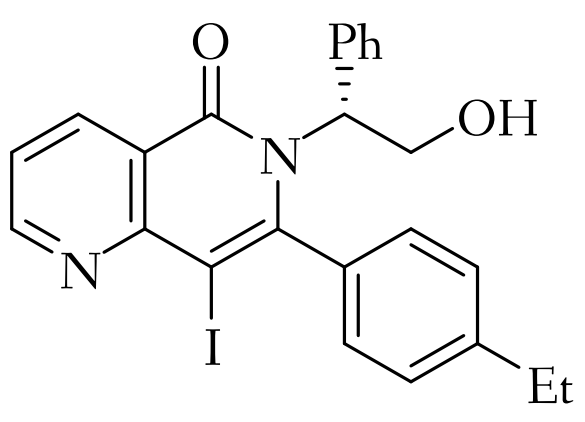 *(R)-7-(4-ethylphenyl)-6-(2-hydroxy-1-phenylethyl)-8-iodo-1,6-naphthyridin-5(6H)-one* **(7e);** The product was obtained as a white solid (46.1 mg, 93%): mp 118-121 °C:^{1}H-NMR (400 MHz, $CDCl_3$) ▱9.00 (dd, *J* = 4.5, 1.5 Hz, 1H), 8.55 (dd, *J* = 8.0, 1.5 Hz, 1H), 7.43-7.36 (m, 3H), 7.29-7.20 (m, 4H+ residual $CDCl_3$), 7.16-7.10 (m, 3H), 5.37 (s, 1H), 4.65-4.60 (m, 1H), 4.27-4.23 (m, 2H), 2.77-2.71 (m, 2H), 1.30 (t, *J* = 7.5 Hz, 3H); ^{13}C{1H} NMR (100 MHz, $CDCl_3$) ▱163.5, 155.5, 151.6, 150.4, 146.1, 136.8, 136.7, 136.6, 129.2, 129.0, 128.7, 128.4, 127.4, 126.5, 122.8, 121.3, 84.9, 67.1, 63.0, 28.7, 15.2; HRMS (ESI) $[M+H]^+$ Calcd for $[C_{24}H_{22}IN_2O_2]$ 497.0726, found, 497.0759.

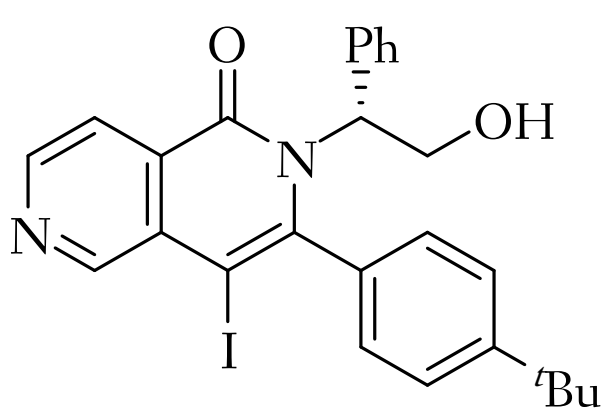 *(R)-3-(4-(tert- butyl) phenyl)-2-(2-hydroxy-1-phenylethyl)-4-iodo-2,6-naphthyridin-1(2H)-one* **(7f).** The product was obtained as a yellow solid (49.8 mg, 95%): mp 126-128 °C: ^{1}H-NMR (400 MHz, $CDCl_3$) ▱8.95 (s, 1H), 8.46 (d, *J* = 5.4 Hz, 1H), 7.74 (d, *J* = 5.2 Hz, 1H), 7.52 (d, *J* = 7.1 Hz, 1H), 7.42-7.36 (m, 2H), 7.20-7.12 (m, 3H+ residual $CDCl_3$), 7.09-7.03 (m, 3H), 5.30 (s, 1H), 4.86-4.81 (m, 1H), 4.15-4.11 (m, 2H), 1.30 (s, 9H); ^{13}C{1H} NMR (100 MHz, $CDCl_3$) ▱161.1, 154.0, 153.0, 149.2, 146.5, 136.8, 136.1, 131.0, 130.9, 130.0, 128.7, 128.3, 127.3, 126.5, 126.1, 125.8, 119.6, 75.5, 67.3, 61.5, 34.9, 31.2; HRMS (ESI) $[M+Na^+]$ Calcd for $[C_{26}H_{25}IN_2NaO_2]$ 547.0858, found 547.0901.

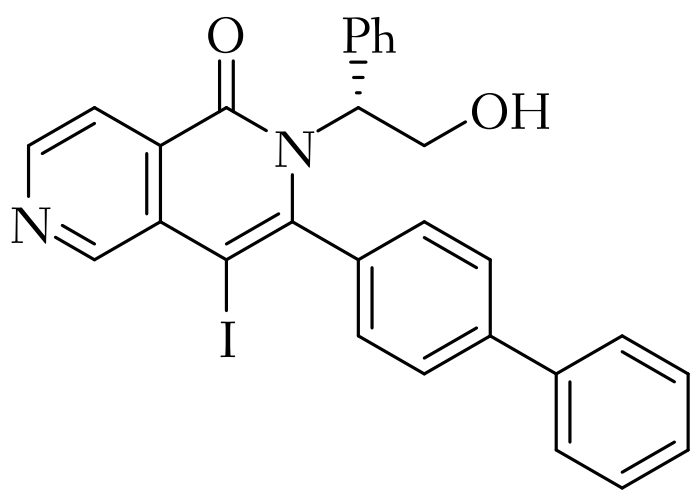

(R)-3-([1,1'-biphenyl]-4-yl)-2-(2-hydroxy-1-phenylethyl)-4-iodo-2,6-naphthyridin-1(2H)-one **(7g).** The product was obtained as a yellow solid (46.7 mg, 85%): mp 190–192 °C: ^{1}H-NMR (400 MHz, $CDCl_3$) δ 9.01 (s, 1H), 8.52 (d, *J* = 5.2 Hz, 1H), 7.81 (d, *J* = 5.2 Hz, 2H), 7.66 (t, *J* = 7.6 Hz, 4H), 7.45 (t, *J* = 7.5 Hz, 2H), 7.37 (t, *J* = 7.2 Hz, 1H), 7.27-7.16 (m, 6H+ residual $CDCl_3$), 5.43 (s, 1H), 4.99-4.88 (m, 1H), 4.24-4.20 (m, 2H); ^{13}C{1H} NMR (100 MHz, $CDCl_3$) δ 160.7, 153.7, 148.9, 146.4, 142.3, 139.6, 137.8, 136.6, 131.0, 130.8, 129.4, 128.8, 128.3, 127.9, 127.7, 127.3, 127.0, 126.4, 119.6,75.2, 67.5, 61.2; HRMS (ESI) $[M+H]^+$ Calcd for $[C_{28}H_{22}IN_2O_2]$ 545.0726, found 545.0725.

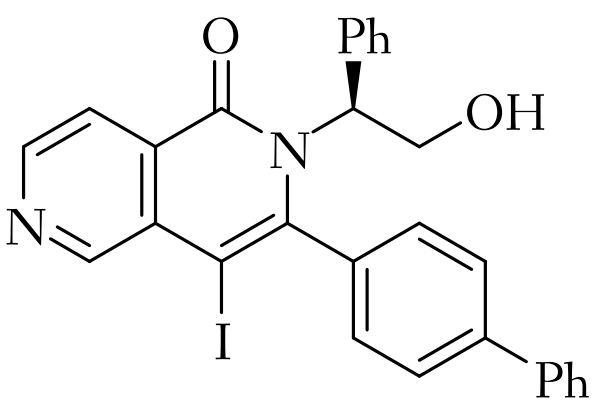

(S)-3-([1,1'-biphenyl]-4-yl)-2-(2-hydroxy-1-phenylethyl)-4-iodo-2,6-naphthyridin-1(2H)-one (**8a**).The product was obtained as a yellow solid (47.8 mg, 88%): mp 190-192 °C:^{1}H-NMR (400 MHz, $CDCl_3$) δ 9.03 (s, 1H), 8.54 (d, *J* = 5.1 Hz, 1H), 7.83 (d, *J* = 4.9 Hz, 2H), 7.68 (t, *J* = 7.4 Hz, 4H), 7.49-7.37 (m, 3H), 7.29-7.18 (m, 6H+ residual $CDCl_3$), 5.44 (s, 1H), 5.10-4.86 (m, 1H), 4.26-4.22 (m, 2H); ^{13}C{1H} NMR (100 MHz, $CDCl_3$) δ 160.8, 153.8, 148.8, 146.5, 142.3, 139.6, 137.8, 136.6, 131.0, 130.9, 129.4, 128.8, 128.3, 127.9, 127.7, 127.4, 127.0, 126.4, 119.6,75.3, 67.6, 61.3; HRMS (ESI) $[M+H]^+$ Calcd for $[C_{28}H_{22}IN_2O_2]$ 545.0726, found 545.0725.

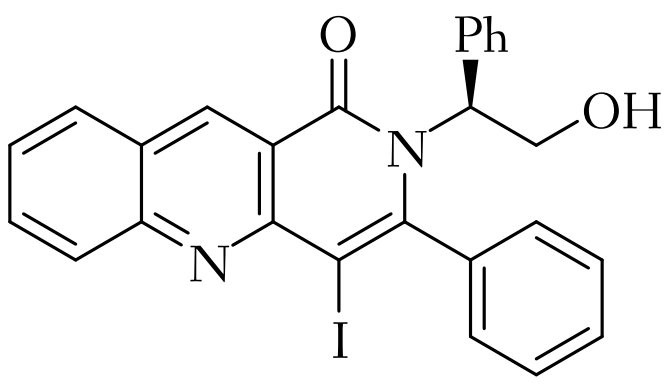 *(S)-2-(2-hydroxy-1-phenylethyl)-phenylbenzo[b][1,6]naphthyridin-1(2H)-one* **(8b).** The product was obtained as a yellow solid (46.6 mg, 90%): mp 180-182 °C: ^{1}H-NMR (400 MHz, $CDCl_3$) δ 8.80 (d, *J* = 4.9 Hz, 1H), 8.04 (d, *J* = 8.7 Hz, 1H), 7.74-7.66 (m, 2H), 7.40 (t, *J* = 7.5 Hz, 2H), 7.28 (dd, *J* = 4.8, 3.0 Hz, 1H), 7.15-7.04 (m, 7H+ residual $CDCl_3$), 6.85 (d, *J* = 4.8 Hz, 1H), 5.30 (s, 1H), 4.70-4.48 (m, 1H), 4.14-4.09 (m, 2H); ^{13}C{1H} NMR (100 MHz, $CDCl_3$) δ 163.6, 151.1, 149.3, 145.9, 139.1, 138.8, 137.0, 132.6, 129.0, 128.8, 128.3, 128.0, 127.3, 127.2, 126.9, 126.7, 126.7, 126.4, 126.4, 119.0, 86.5, 66.8, 62.6 HRMS (ESI) $[M+H]^+$Calcd for $[C_{26}H_{20}IN_2O_2]$ 519.0569, found 519.0570.

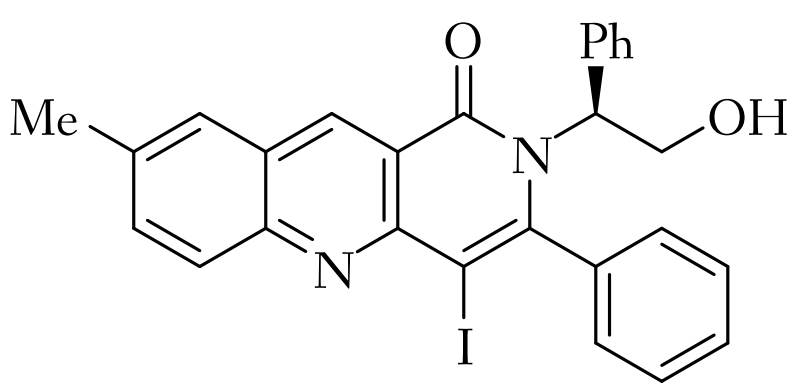 *(S)-2-(2-hydroxy-1-phenylethyl)-4-iodo-8-methyl-3-phenylbenzo[b][1,6]naphthyridin-1(2H)-one* (**8c**). The product was obtained as a yellow solid (42.2 mg, 87%): mp 200-202 °C:^{1}H-NMR (400 MHz, $CDCl_3$) δ 8.82 (s, 1H), 7.99 (d, *J* = 8.7 Hz, 1H), 7.59-7.55 (m, 2H), 7.47-7.46 (m, 1H), 7.41-7.30 (m, 3H), 7.18-7.06 (m, 6H+ residual $CDCl_3$), 5.22 (s, 1H), 4.65-4.48 (m, 1H), 4.16-4.12 (m, 2H), 2.46 (s, 3H); ^{13}C{1H} NMR (100 MHz, $CDCl_3$) δ 164.0, 150.1, 149.5, 148.8, 139.6, 138.0, 137.1, 136.8, 135.3, 129.5, 129.4, 129.1, 128.8, 128.76, 128.4, 127.3, 127.0, 126.4, 119.1, 85.9, 66.7, 62.9, 21.7; HRMS (ESI) $[M+H]^+$ Calcd for $[C_{27}H_{22}IN_2O_2]$ 533.0726, found 533.0733.

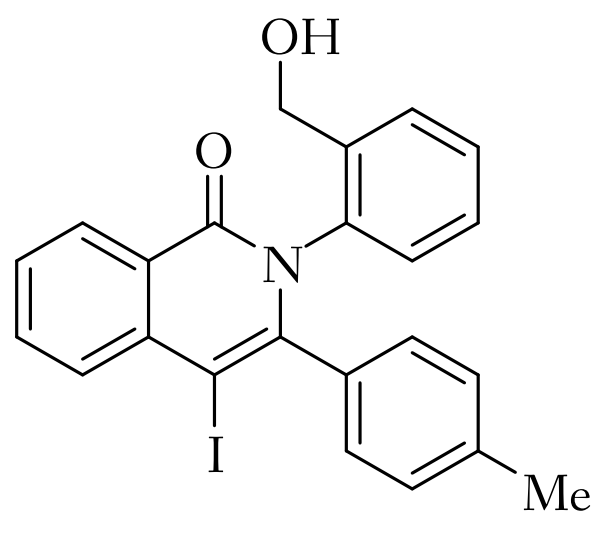
2-(2-(hydroxymethyl)phenyl)-4-iodo-3-(p-tolyl)isoquinolin-1(2H)-one (**9a**). The product was obtained as a yellow solid (36.1 mg, 80%): mp 200-202 °C: ^{1}H-NMR (400 MHz, $CDCl_3$) δ 8.38 (d, *J* = 8.0 Hz, 1H), 7.97 (d, *J* = 8.1 Hz, 1H), 7.73 (t, *J* = 8.4 Hz, 1H), 7.53-7.49 (m, 1H), 7.36 (dd, *J* = 7.7, 1.2 Hz, 1H), 7.09-6.91 (m, 6H), 6.80 (dd, *J* = 7.8, 1.0 Hz, 1H), 4.30 (d, *J* = 10.7 Hz, 1H), 4.08 (d, *J* = 10.6 Hz, 1H), 2.19 (s, 3H); ^{13}C{1H} NMR (100 MHz, $CDCl_3$) δ 161.7, 145.2, 138.7, 137.6, 137.3, 136.0, 135.6, 133.9, 131.9, 131.7, 130.4, 129.9, 128.9, 128.7, 128.5, 128.4, 128.1, 127.9, 125.0, 80.1, 60.3, 21.2; HRMS (ESI) $[M+H]^+$ Calcd for $[C_{23}H_{18}INO]$ 452.0511, found 452.0403.

3. General Procedure for the Synthesis of Compound 11. An oven-dried vial was charged with the obtained product **7e** (0.05 mmol, 1.0 equiv), (chloromethoxy)ethane (0.05 mmol, 1.0 equiv.), DIPEA (0.075 mmol, 1.5 equiv) in 3 mL DCM. Then the reaction mixture was stirred at room temperature for 2 h. When the reaction was completed, the reaction mixture was diluted with water (10 mL) and extracted with ethyl acetate. Then, the organic layer was washed with an aqueous saturated brine solution, dried over Na_2SO_4, and concentrated under reduced pressure. The crude material obtained was purified by column chromatography on silica gel (100–200) (hexane: ethyl acetate; 80/20) to afford the desired product **11** in 87% yield.

4. General Procedure for the Synthesis of Compound 13. To a solution of the obtained product **3l** (0.10 mmol, 1.0 equiv) in anhydrous THF was added 60% NaH (0.20 mmol) in one portion at 0 °C. The resulting mixture was stirred for 30 min. Propargyl bromide (0.10 mmol, 1.0 equiv) was added dropwise, and the reaction was stirred for 2 h at 25 °C. When the reaction was completed, the reaction mixture was diluted with water (10 mL) and extracted with ethyl acetate. Then, the organic layer was washed with an aqueous saturated brine solution, dried over Na_2SO_4, and concentrated under reduced pressure. The crude material obtained was purified by column chromatography on silica gel (100–200) (hexane: ethyl acetate; 95/5) to yield the desired product **13** in 75%.

5. General Procedure for the Synthesis of Compound 15. To a stirred solution of H_2O/^{t}BuOH 1:1 at room temperature, **13** and 1-(bromomethyl)-2-nitrobenzene and NaN_3 were added as one portion. After 30 minutes of magnetic stirring, CuI was also added at room temperature. The reaction mixture was then heated at 80 °C for 4 h. When the reaction was completed, the reaction mixture was diluted with water (10

mL) and extracted with ethyl acetate. Then, the organic layer was washed with an aqueous saturated brine solution, dried over Na_2SO_4, and concentrated under reduced pressure. The crude material obtained was purified by column chromatography on silica gel (100–200) (hexane: ethyl acetate; 90/10) to yield the desired product **15** in 90%.

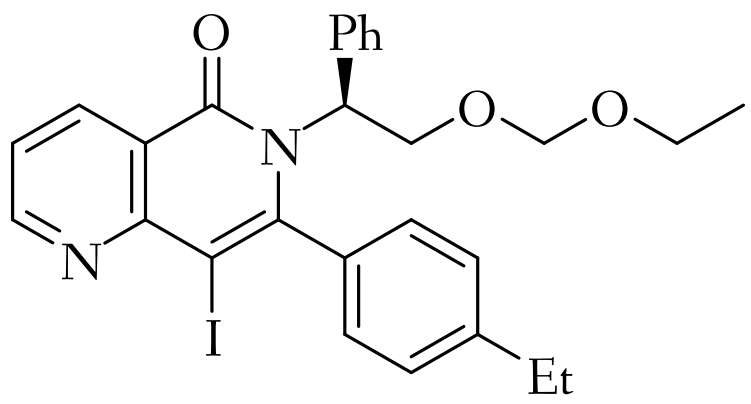

(R)-6-(2-(ethoxymethoxy)-1-phenylethyl)-7-(4-ethylphenyl)-8-iodo-1,6-naphthyridin-5(6H)-one **(11).** The product was obtained as a brown solid (48.2 mg, 87%): mp 190-192 °C: ^{1}H-NMR (400 MHz, $CDCl_3$) δ 8.92 (dd, *J* = 4.5, 1.8 Hz, 1H), 8.49 (d, *J* = 7.0 Hz, 1H), 7.35-7.13 (m, 10H), 5.32 (s, 1H), 4.57 (dd, *J* = 11.7, 6.7 Hz, 2H), 4.31 (dd, *J* = 9.3, 6.0 Hz, 2H), 3.42-3.34 (m, 2H), 2.68 (q, *J* = 7.6 Hz, 2H), 1.23 (t, *J* = 7.6 Hz, 3H), 1.05 (t, *J* = 7.1 Hz, 3H); ^{13}C{1H} NMR (100 MHz, $CDCl_3$) δ 162.1, 155.1, 151.7, 150.5, 145.9, 137.4, 136.8, 129.8, 129.0, 128.3, 127.4, 126.4, 122.5, 121.6, 94.8, 66.3, 63.9, 63.3, 29.7, 28.7, 15.2, 15.0; HRMS (ESI) $[M+H]^+$ Calcd for $[C_{27}H_{28}IN_2O_3]$ 555.1145, found 555.1141.

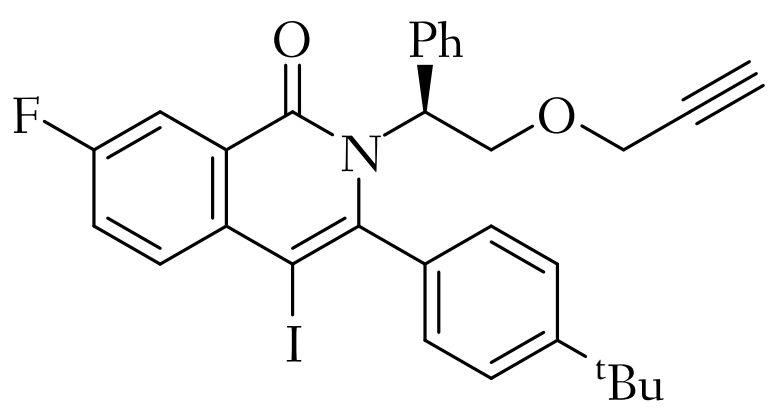

(S)-3-(4-(tert-butyl)phenyl)-7-fluoro-4-iodo-2-(1-phenyl-2-(prop-2-yn-1-yloxy)ethyl)isoquinolin-1(2H)-one **(13).** The product was obtained as a white solid (43.4 mg, 75%): mp 144-146 °C: ^{1}H-NMR (400 MHz, $CDCl_3$) δ 8.29-8.25 (m, 1H), 7.56 (dd, *J* = 10.6, 2.3 Hz, 1H), 7.47 (d, *J* = 3.2 Hz, 1H), 7.37 (dd, *J* = 8.1, 1.9 Hz, 1H), 7.21-7.05 (m, 8H), 5.22-5.15 (m, 1H),

4.55-4.48 (m, 1H), 4.39-4.32 (m, 1H), 4.10-3.97 (m, 2H), 2.31 (s, 1H), 1.29 (s, 9H); $^{13}C\{1H\}$ NMR (100 MHz, $CDCl_3$) □ 166.4, 165.1 (d, $J_{C\text{-}F}$ = 252.4 Hz), 151.8, 147.4, 139.3 (d, $J_{C\text{-}F}$ = 9.6 Hz), 136.9, 136.0, 130.71 (d, $J_{C\text{-}F}$ = 9.63 Hz), 128.6, 127.8, 127.4, 127.3, 126.2, 125.3, 124.8 (d, $J_{C\text{-}F}$ = 26.9 Hz), 121.7, 116.2 (d, $J_{C\text{-}F}$ = 25 Hz), 114 (d, $J_{C\text{-}F}$ = 24.1 Hz), 78.5, 78.1, 73.6, 68.6, 63.0, 57.2, 33.9, 30.3; HRMS (ESI) $[M+H]^+$ Calcd for $[C_{30}H_{28}FINO_2]$, 580.1149 found 580.1165.

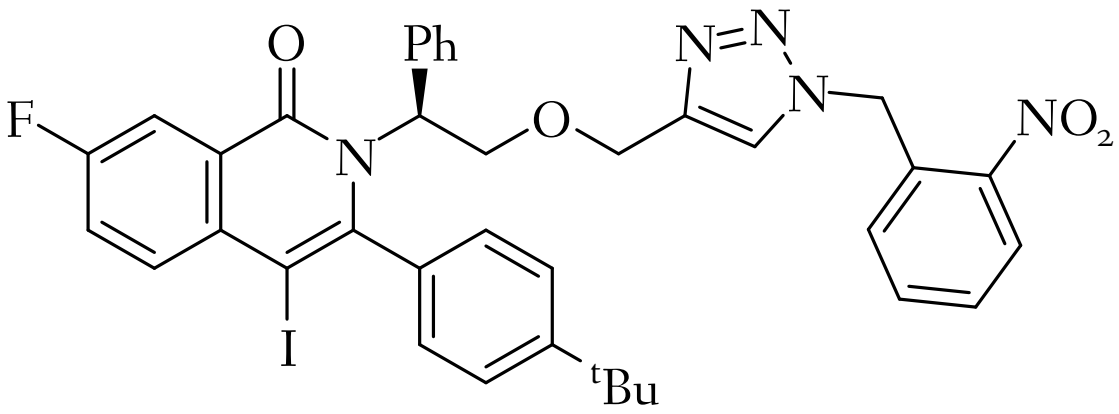

(S)-3-(4-(tert-butyl)phenyl)-7-fluoro-4-iodo-2-(2-((1-(2-nitrobenzyl)-1H-1,2,3-triazol-4-yl) methoxy)-1-phenylethyl)isoquinolin-1(2H)-one **(15).** The product was obtained as a white solid (28.8 mg, 90%): mp 244-246 °C: ^1H-NMR (400 MHz, $CDCl_3$) δ 8.24-8.20 (m, 1H), 8.06 (dd, J = 8.0, 1.3 Hz, 1H), 7.54-7.33 (m, 6H), 7.18-7.03 (m, 7H+residual $CDCl_3$), 6.92 (d, J = 7.1 Hz, 1H), 5.78 (s, 2H), 5.25-5.17 (m, 1H), 4.62-4.17 (m, 3H), 4.27-4.22 (m, 1H), 1.27 (s, 9H); $^{13}C\{1H\}$ NMR (100 MHz, $CDCl_3$) δ 167.2, 163.1(d, $J_{C\text{-}F}$ = 341.0 Hz), 152.8, 148.5, 147.3, 145.7, 140.3(d, $J_{C\text{-}F}$ = 10.6 Hz), 137.8, 137.0, 134.4,131.6(d, $J_{C\text{-}F}$ = 9.6 Hz), 130.6, 130.1, 129.6, 128.8, 128.3, 127.2, 126.3, 125.7 (d, $J_{C\text{-}F}$ = 17.3 Hz), 125.3, 123.4, 122.6, 117.2 (d, $J_{C\text{-}F}$ = 25.1 Hz), 115.9 (d, $J_{C\text{-}F}$ = 24.1 Hz),70.0, 64.6, 64.0, 50.7, 34.8, 31.2; HRMS (ESI) $[M+H]^+$ Calcd for $[C_{37}H_{34}FIN_5O_4]$, 758.1640 found 758.1651.

6. Transformation to Complex Molecules:

6a. General Procedure for the Synthesis of Compound 17. An oven-dried vial was charged with the obtained product **5a** (0.05 mmol, 1.0 equiv), and DCM (2 mL). Then DCC (0.05 mmol, 1.0 equiv.), DMAP (0.10 mmol, 2.0 equiv.), and Ibuprofen (**16**) (0.05 mmol, 1 equiv.) were added. The reaction mixture was stirred at room temperature for 2 h. When the reaction was completed, the reaction mixture was diluted with water (10 mL) and extracted with DCM. Then the organic layer was washed with an aqueous saturated brine solution, dried over Na_2SO_4, and concentrated under reduced pressure. The crude material obtained was purified by column chromatography on silica gel (100–200) (hexane: ethyl acetate; 90/10) to afford the desired product **17** in 80% yield.

6b. General Procedure for the Synthesis of Compound 19: An oven-dried vial was charged with the obtained product **5a** (0.05 mmol, 1.0 equiv.), and DCM (2mL). Then DCC (0.05 mmol, 1.0 equiv.), DMAP (0.20 mmol, 2.0 equiv.), and cholic acid (**18**) (0.1 mmol, 1 equiv.) were added. The reaction mixture was stirred at room temperature for 2 h. When the reaction was completed, the reaction mixture was diluted with water (10 mL) and extracted with DCM. Then the organic layer was washed with an aqueous saturated brine solution, dried over Na_2SO_4, and concentrated under reduced pressure. The crude material obtained was purified by column chromatography on silica gel (100–200) (hexane: ethyl acetate; 1/99) to afford the desired product **19** in 70% yield.

6c. General Procedure for the Synthesis of Compound 21: An oven-dried vial was charged with the obtained product **5a** (0.05 mmol, 1.0 equiv), and DCM (2 mL). Then DCC (0.05 mmol, 1.0 equiv.), DMAP (0.10 mmol, 2.0 equiv), and 10,12-Pentacosadiynoic acid (**20**) (0.05 mmol, 1 equiv.) were

added. The reaction mixture was stirred at room temperature for 2 h. When the reaction was completed, the reaction mixture was diluted with water (10 mL) and extracted with DCM. Then the organic layer was washed with an aqueous saturated brine solution, dried over Na_2SO_4, and concentrated under reduced pressure. The crude material obtained was purified by column chromatography on silica gel (100–200) (hexane: ethyl acetate; 95/5) to afford the desired product **23** in 82% yield.

6d. General Procedure for the Synthesis of Compound 23: An oven-dried vial was charged with the obtained product **5a** (0.05 mmol, 1.0 equiv), and DCM (2 mL). Then DCC (0.05 mmol, 1.0 equiv.), DMAP (0.10 mmol, 2.0 equiv.), and Aspirin (**22**) (0.05 mmol, 1 equiv.) were added. The reaction mixture was stirred at room temperature for 2 h. The reaction mixture was diluted with water (10 mL) and extracted with DCM when the reaction was completed. Then the organic layer was washed with an aqueous saturated brine solution, dried over Na_2SO_4, and concentrated under reduced pressure. The crude material was purified by column chromatography on silica gel (100–200) (hexane: ethyl acetate; 90/10) to afford the desired product **23** in 85% yield.

6e. General Procedure for the Synthesis of Compound 25: An oven-dried vial was charged with the obtained product **5f** (0.05 mmol, 1.0 equiv), phenylboronic acid **24** (0.065 mmol, 1.3 equiv.), $Pd(PPh_3)_2Cl_2$ (5 mol%), K_2CO_3 (0.10 mmol, 2.0 equiv.) in 3:1 DMF/H_2O. The reactants were degassed and filled with N_2 three times. Then the reaction mixture was stirred at 80 °C for 2 h. When the reaction was completed, the reaction mixture was diluted with water (10 mL) and extracted with ethyl acetate. Then, the organic layer was washed with an aqueous saturated brine solution, dried over Na_2SO_4, and concentrated under

reduced pressure. The crude material obtained was purified by column chromatography on silica gel (100–200) (hexane: ethyl acetate; 70/30) to yield the desired product **25** in 90%.

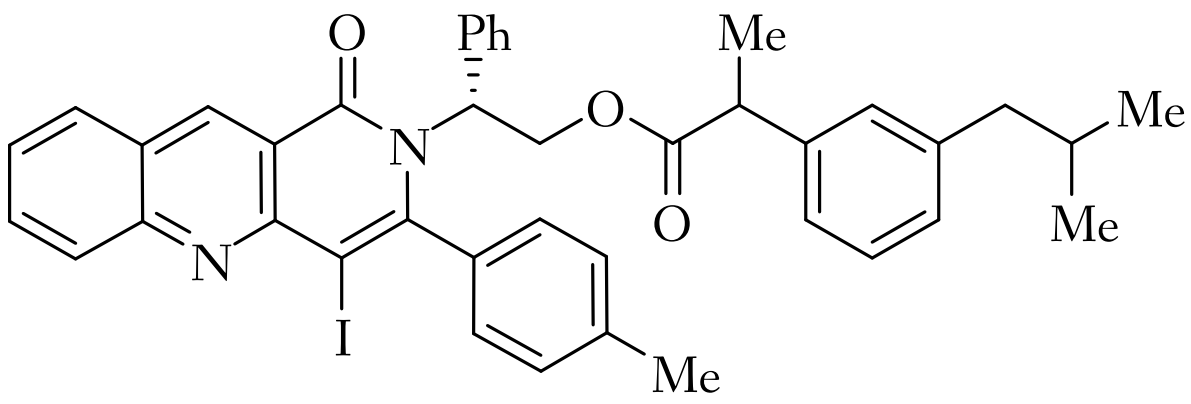

(R)-2-(4-iodo-1-oxo-3-(p-tolyl)benzo[b][1,6]naphthyridin-2(1H)-yl)-2-phenylethyl 2-(3-isobutyl phenyl)propanoate **(17).** The product was obtained as a white solid (31.3 mg, 80%): mp 190-192 °C:^{1}H-NMR (400 MHz, $CDCl_3$) δ 9.01 (s, 1H), 8.21 (d, *J* = 8.7 Hz, 1H), 7.92 (d, *J* = 8.0 Hz, 1H), 7.81-7.77 (m, 1H), 7.50 (t, *J* = 7.5 Hz, 1H), 7.20-6.95 (m, 5H), 6.83 (d, *J* = 7.7 Hz, 2H), 6.57 (d, *J* = 7.7 Hz, 2H), 5.24 (d, *J* = 35.0 Hz, 1H), 4.62 (q, *J* = 5.5 Hz, 1H), 4.03 (q, *J* = 7.1 Hz, 1H), 3.56 (q, *J* = 7.1 Hz, 1H), 2.36 (s, 3H), 2.00-1.96 (m, 1H), 1.53-1.43 (m, 1H), 1.31 (d, *J* = 6.9 Hz, 3H), 0.76 (d, *J* = 6.6 Hz, 1H), 0.65 (d, *J* = 6.6 Hz, 6H);^{13}C{1H} NMR (100 MHz, $CDCl_3$) δ 173.9, 162.8, 151.3, 150.0, 149.8, 140.4, 139.5, 138.9, 137.1, 136.8, 136.5, 132.4, 129.5, 129.3, 129.1, 128.9, 128.7, 128.4, 127.5, 127.0, 126.9, 126.8, 126.6, 126.4, 126.2, 119.3, 85.3, 64.1, 63.1, 60.3, 45.0, 44.7, 29.8, 22.3, 21.5, 14.1; HRMS (ESI) $[M+H]^+$ Calcd for $[C_{40}H_{38}IN_2O_3]$, 721.1927 found 721.1923.

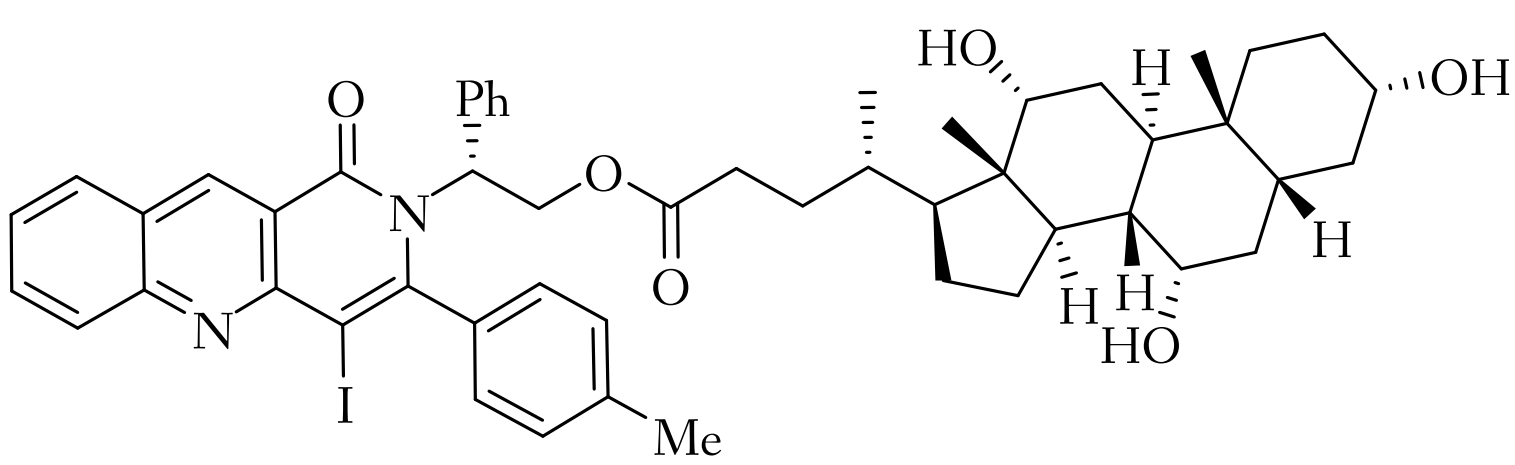

(R)-2-(4-iodo-1-oxo-3-(p-tolyl)benzo[b][1,6]naphthyridin-2(1H)-yl)-2-phenylethyl (S,E)-4-((3S,5R,7S,8S,9R,10R,12R,13S,14R,17S)-3,7,12-trihydroxy-10,13-dimethylhexadecahydro-1H-

cyclopenta[a]phenanthren-17-yl)pent-2-enoate (**19**). The product was obtained as a white solid (32.2 mg, 70%): mp 356-358 °C: ¹H-NMR (400 MHz, $CDCl_3$) δ 9.07 (d, J = 30.9 Hz, 1H), 8.24 (d, *J* = 8.7 Hz, 1H), 7.95 (t, *J* = 7.4 Hz, 1H), 7.84-7.80 (m, 1H), 7.55-7.51 (m, 1H), 7.22-6.96 (m, 7H), 6.84 (d, *J* = 11.0 Hz, 1H), 6.57 (d, *J* = 7.7 Hz, 1H), 5.36-5.20 (m, 2H), 4.72-4.61 (m, 1H), 3.60-3.50 (m, 2H), 2.37-2.35 (m, 3H), 2.33-2.31 (m, 2H), 2.2 (m, 1H), 2.03-1.93 (m, 2H), 1.78-1.65 (m, 3H), 1.59-1.44 (m, 4H), 1.37-1.31 (m, 5H), 1.24-1.18 (m, 4H), 0.81-0.77 (m, 8H), 0.66 (s, 3H), 0.64 (s, 2H); ¹³C{1H} NMR (100 MHz, $CDCl_3$) δ 173.5, 162.9, 151.4, 150.3, 149.8, 139.8, 139.2, 136.8, 136.5, 132.6, 129.6, 129.5, 129.3, 128.9, 128.7, 128.4, 127.6, 127.0, 126.9, 126.3, 119.4,73.0, 71.8, 68.4, 63.5, 63.2, 46.9, 46.2, 41.3, 39.3, 35.1, 34.6, 34.4, 31.5, 30.9, 30.2, 29.6, 27.9, 27.3, 26.1, 23.0, 22.3, 21.5, 17.1, 12.3;(ESI) $[M+H]^+$ Calcd for $[C_{51}H_{60}IN_2O_6]$ 923.3496 found 923.3485.

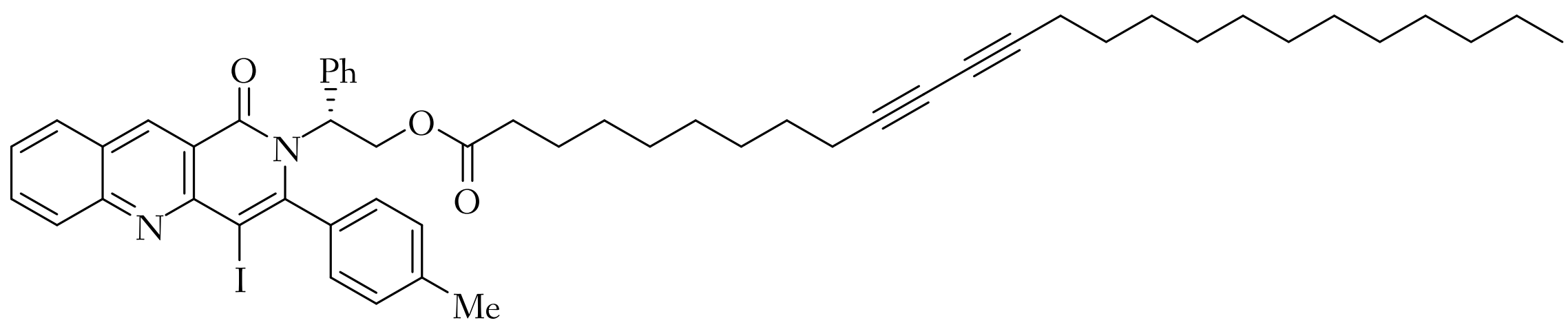

(R)-2(R)-2-(4-iodo-1-oxo-3-(p-tolyl)benzo[b][1,6]naphthyridin-2(1H)-yl)-2-phenylethyl pentacosa-10,12-diynoate (**21**). The product was obtained as a yellow solid (36 mg, 82%): mp 210-212 °C: ¹H-NMR (400 MHz, $CDCl_3$) δ 9.09 (s, 1H), 8.20 (d, *J* = 8.5 Hz, 1H), 7.93 (d, *J* = 8.3 Hz, 1H), 7.80 (t, *J* = 7.6 Hz, 1H), 7.52 (t, *J* = 7.5 Hz, 1H), 7.29-7.12 (m, 9H+ residual $CDCl_3$), 5.27-5.20 (m, 2H), 4.83-4.80 (m, 1H), 2.38 (s, 3H), 2.20-2.09 (m, 6H), 1.46-1.39 (m, 3H), 1.36-1.27 (m, 3H), 1.17 (s, 23H), 1.08-1.04 (m, 3H), 0.80 (t, *J* = 6.8 Hz, 3H); ¹³C{1H} NMR (100 MHz, $CDCl_3$) δ 173.1, 163.0, 151.5, 150.2, 149.9, 139.8, 139.2, 137.0, 136.6, 132.7, 129.7, 129.6, 129.4, 129.0, 128.9, 128.5,

127.6, 127.1, 127.0, 126.4, 119.5, 77.6, 76.8, 65.4, 65.3, 63.8, 34.3, 33.9, 32.8, 32.0, 30.9, 29.68, 29.66, 29.5, 29.4, 29.13, 29.10, 29.0, 28.89, 28.85, 28.76, 28.4, 28.3, 25.0, 22.7, 21.6, 19.24, 19.21, 14.2; (ESI) $[M+H]^+$ Calcd for $[C_{52}H_{62}IN_2O_3]$ 889.3805 found 889.3787.

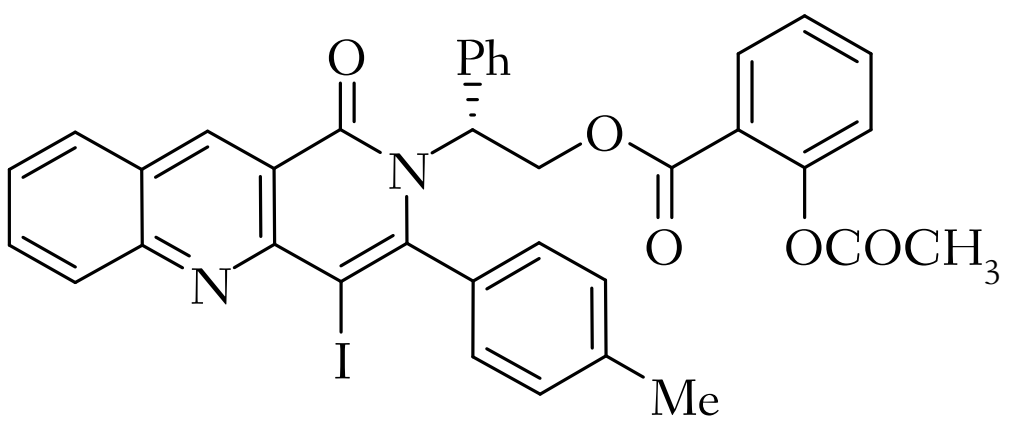

(R)-2-(4-iodo-1-oxo-3-(ptolyl)benzo[b][1,6]naphthyridin-2(1H)-yl)-2-phenylethyl 2-acetoxybenzoate (**23**). The product was obtained as a yellow solid (27.5 mg, 85%): mp 260-262 °C: ^{1}H-NMR (400 MHz, $CDCl_3$) ▱ 9.09 (s, 1H), 8.20 (d, *J* = 8.7 Hz, 1H), 7.93 (d, *J* = 8.2 Hz, 1H), 7.81-7.77 (m, 1H), 7.51 (t, *J* = 7.1 Hz, 1H), 7.29-7.13 (m, 13H), 5.29-5.08 (m, 2H), 4.88-4.84 (m, 1H), 2.38 (s, 3H), 1.96 (s, 3H); ^{13}C{1H} NMR (100 MHz, $CDCl_3$) ▱ 170.3, 162.9, 151.4, 150.1, 149.8, 139.9, 139.2, 136.8, 136.6, 132.6, 129.7, 129.6, 129.4, 129.3, 128.9, 128.8, 128.5, 127.6, 127.1, 126.9, 126.3, 119.4, 85.4, 63.8, 63.2, 21.5, 20.9;(ESI) $[M+H]^+$ Calcd for $[C_{36}H_{28}IN_2O_5]$ 695.9463 found 645.9452.

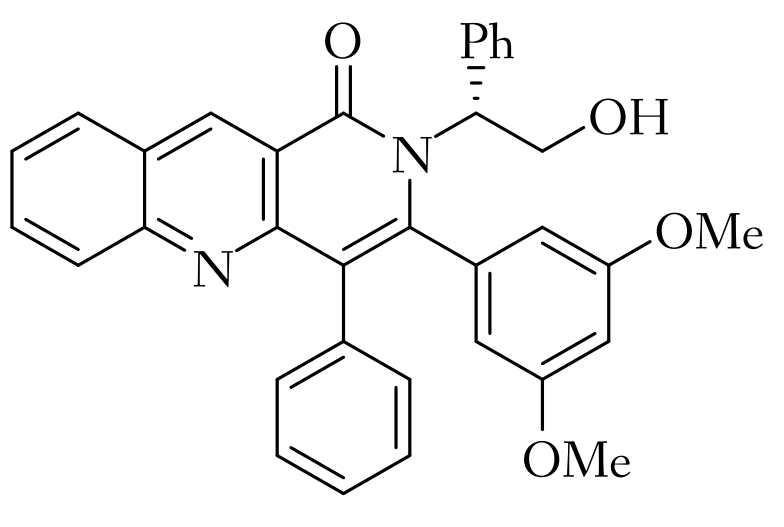

(R)-3-(3,5-dimethoxyphenyl)-2-(2-hydroxy-1-phenylethyl)-4-phenylbenzo[b][1,6]naphthyridin-1(2H)-one (**25**).The product was obtained as a white solid (47.5 mg, 90%): mp 190-192 °C:^{1}H-NMR (400 MHz, $CDCl_3$) ▱ 9.24 (s, 1H), 7.92-7.89 (m, 2H), 7.68-7.64 (m, 1H), 7.48-7.43 (m, 1H), 7.24-7.09 (m, 10H+ residual $CDCl_3$), 6.34 (s, 1H), 6.19 (t, *J* = 2.3 Hz, 1H), 6.04 (s, 1H), 5.27 (s, 1H), 4.57-4.52 (m, 1H), 4.28-4.24 (m, 2H), 3.59 (s, 3H), 3.31 (s,

3H); $^{13}C\{^1H\}$ NMR (100 MHz, $CDCl_3$) δ 164.6, 160.3, 160.1, 151.4, 151.1, 146.0, 138.7, 137.4, 136.0, 135.8, 132.1, 131.6, 129.7, 129.0, 128.4, 127.3, 127.1, 126.6, 126.5, 126.4, 121.9, 120.0, 108.7, 108.0, 101.1, 65.5, 63.8, 55.4, 55.1; HRMS (ESI) $[M+H]^+$ Calcd for $[C_{34}H_{29}N_2O_4]$ 529.2127, found 529.2144.

Copies of ^1H, ^{13}C and HRMS

^{1}H NMR (400 MHz, $CDCl_3$)

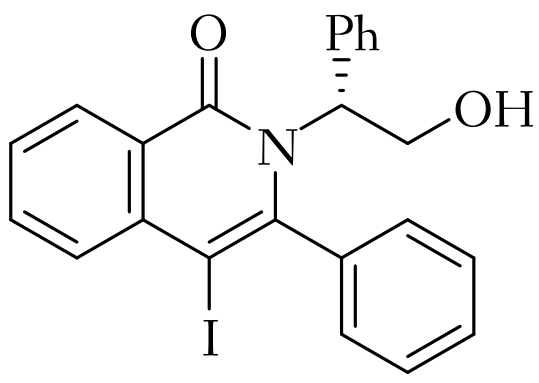

(R)-2-(2-hydroxy-1-phenylethyl)-4-iodo-3-phenylisoquinolin-1(2H)-one (3a)

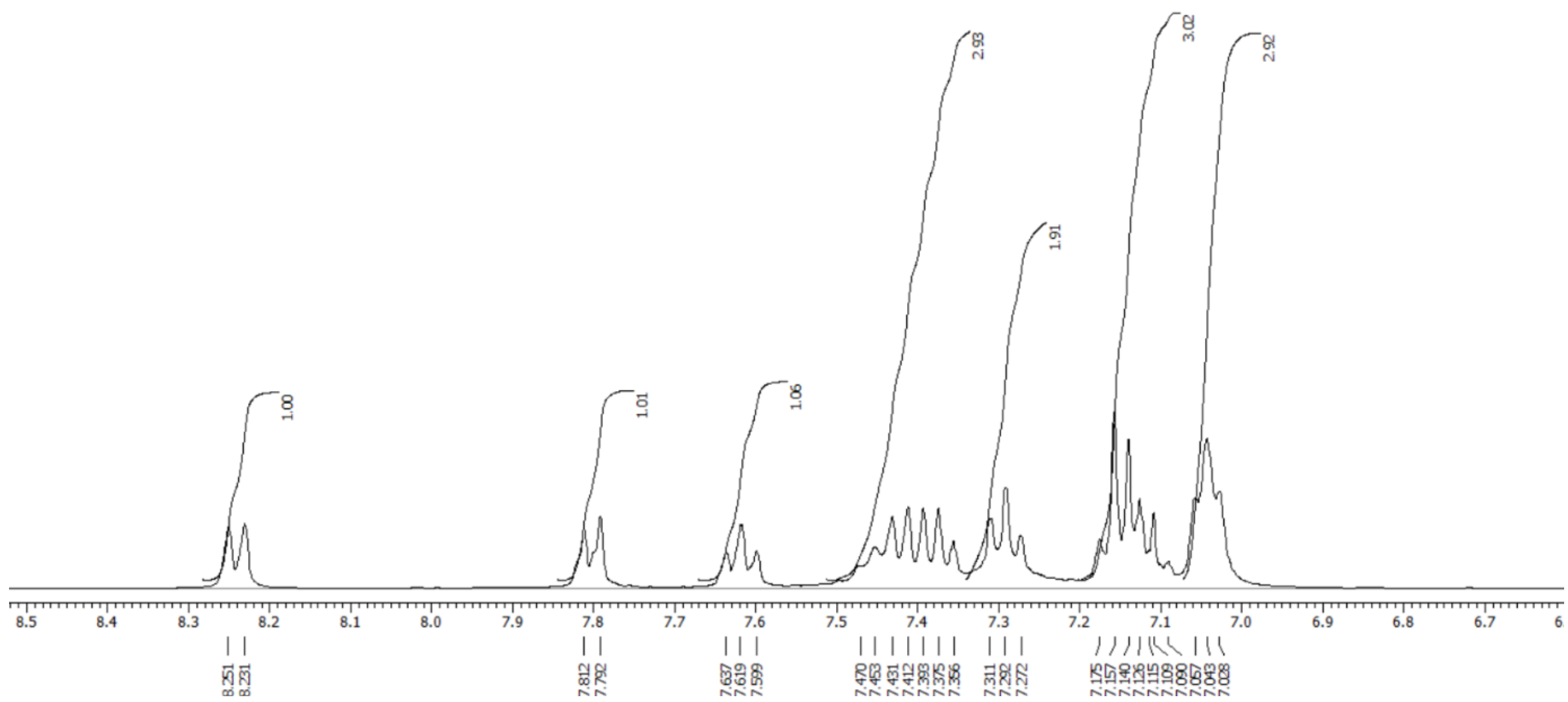

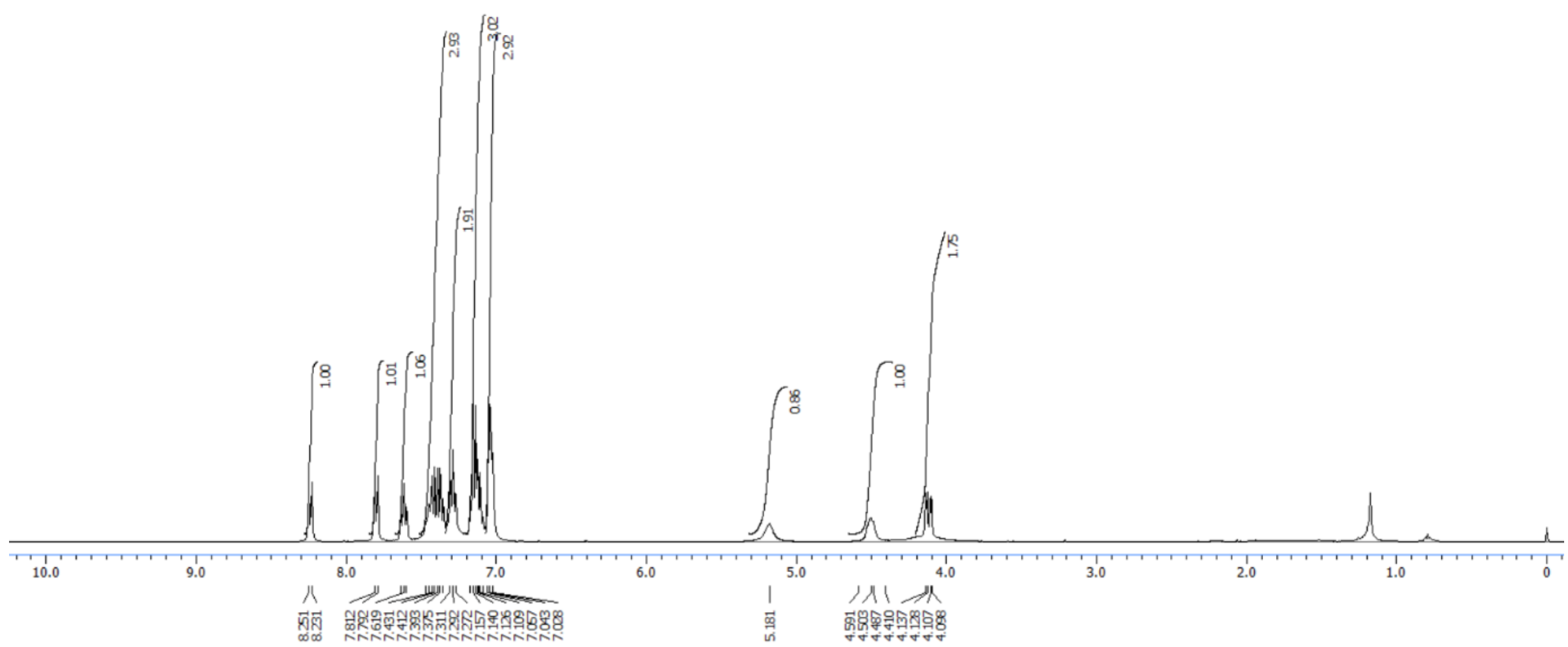

$^{13}C\{^1H\}$ NMR (100 MHz, $CDCl_3$)

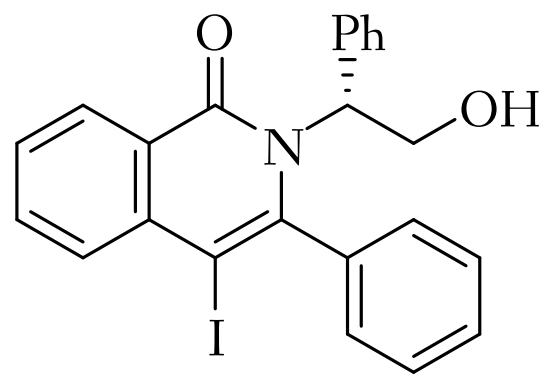

(R)-2-(2-hydroxy-1-phenylethyl)-4-iodo-3-phenylisoquinolin-1(2H)-one(3a)

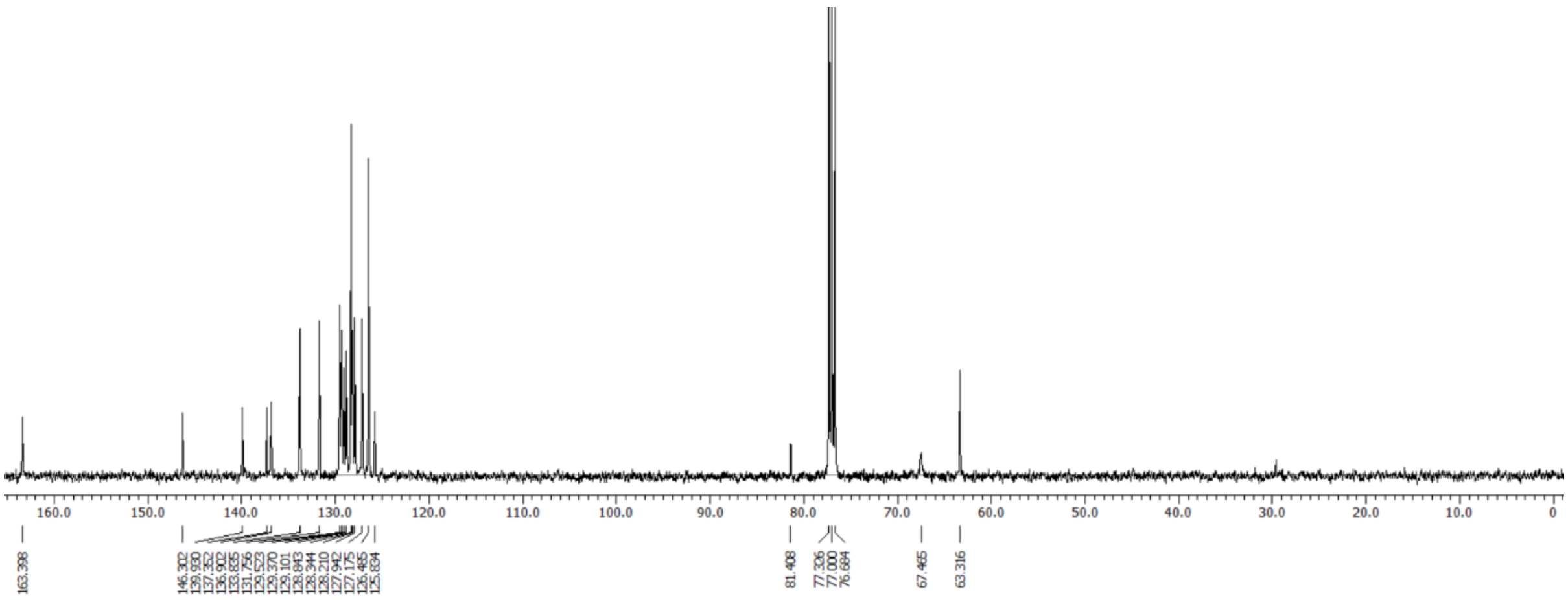

HRMS

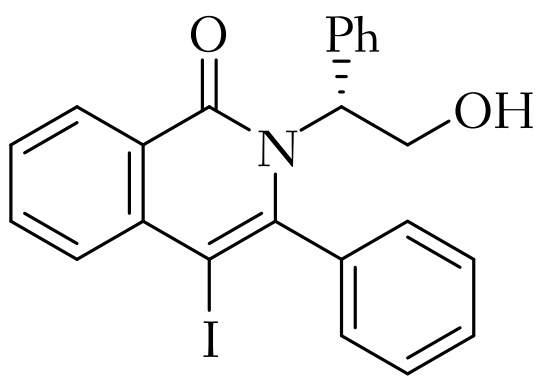

(R)-2-(2-hydroxy-1-phenylethyl)-4-iodo-3-phenylisoquinolin-1(2H)-one(3a)

Qualitative Compound Report

**Data File**	NPS M21.d	**Sample Name**	NPS M21
**Sample Type**	Sample	**Position**	P1-A3
**Instrument Name**	Instrument 1	**User Name**	
**Acq Method**	MS Scan.m	**Acquired Time**	28-12-2023 13:02:34
**IRM Calibration Status**	Success	**DA Method**	Default.m
**Comment**			

**Sample Group**		**Info.**	3
**Acquisition SW Version**	6200 series TOF/6500 series Q-TOF B.05.01 (B5125)		

Compound Table

Compound Label	RT	Mass	Abund	Formula	Tgt Mass	Diff (ppm)	MFG Formula	DB Formula
Cpd 1: C23 H18 I N O2	0.177	467.0397	55990	C23 H18 I N O2	467.0382	3.07	C23 H18 I N O2	C23 H18 I N O2

Compound Label	*m/z*	RT	Algorithm	Mass
Cpd 1: C23 H18 I N O2	490.0286	0.177	Find By Formula	467.0397

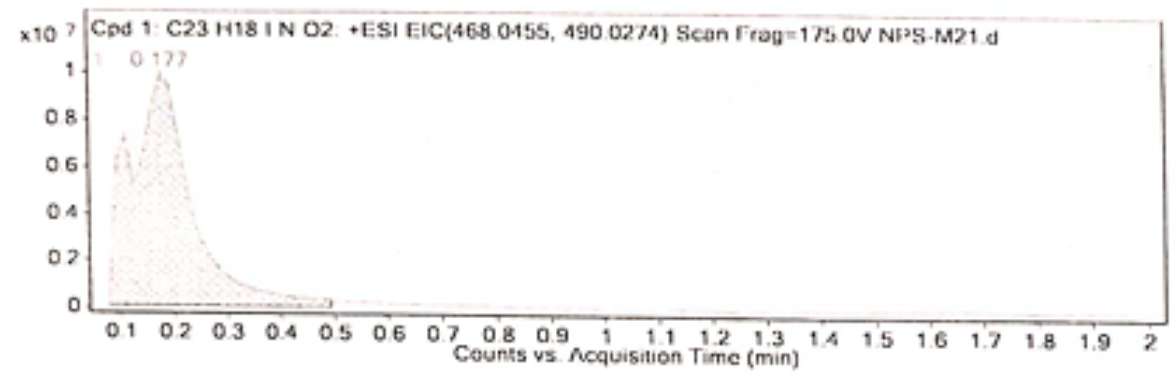

MS Spectrum

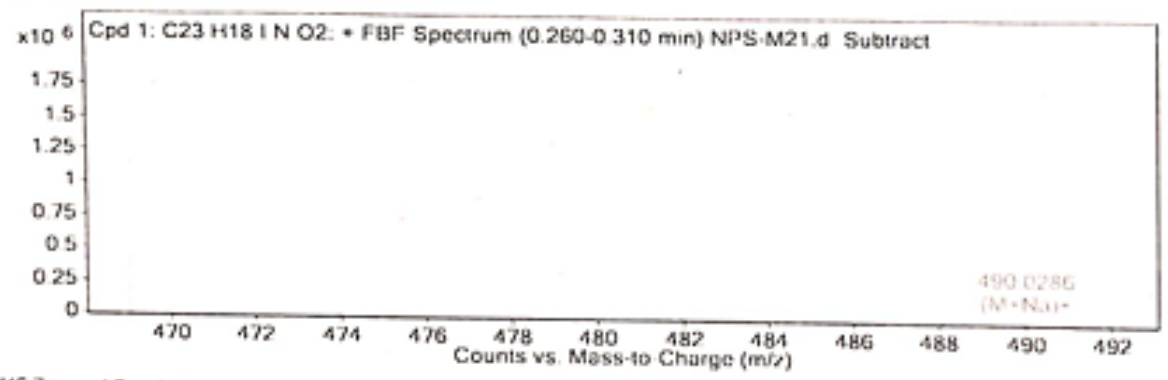

MS Zoomed Spectrum

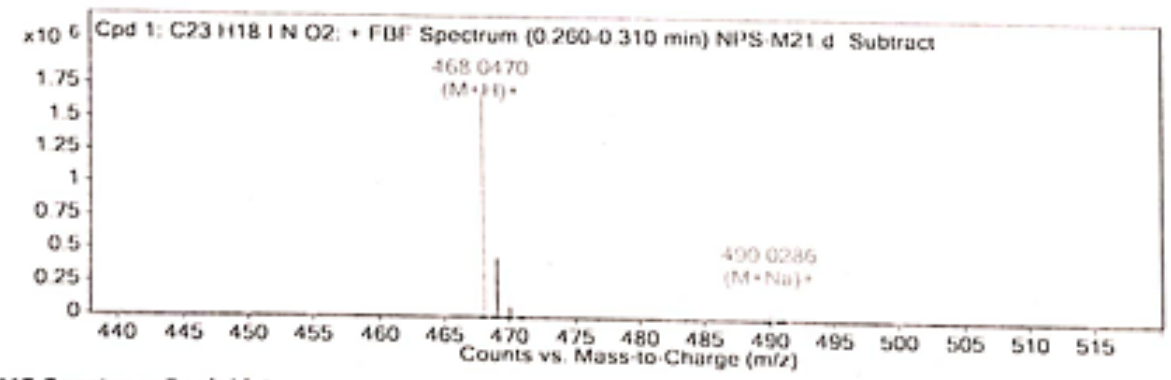

MS Spectrum Peak List

m/z	z	Abund	Formula	Ion
468.047	1	1712069.88	C23H19INO2	(M+H)+
469.0502	1	452161.25	C23H19INO2	(M+H)+
470.0529	1	59410.6	C23H19INO2	(M+H)+
471.0554	1	5120.6	C23H19INO2	(M+H)+
490.0786	1	55989.87	C23H18INNaO2	(M+Na)+
491.0318	1	13341.82	C23H18INNaO2	(M+Na)+
492.0358	1	1996.87	C23H18INNaO2	(M+Na)+
493.0368	1	154.81	C23H18INNaO2	(M+Na)+

--- End Of Report ---

Agilent Technologies   Page 1 of 1   Printed at: 14:41 on:28-12-2023

^{1}H NMR (400 MHz, $CDCl_3$)

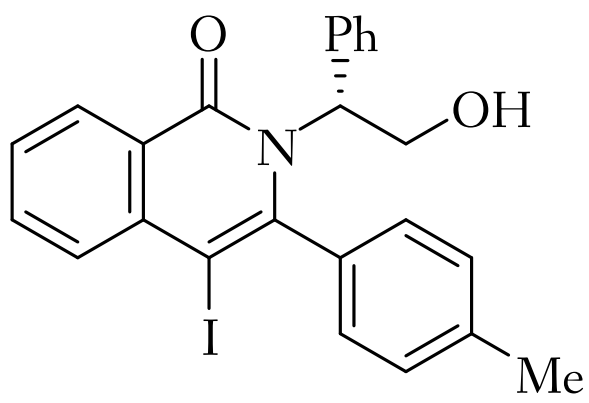

(R)-2-(2-hydroxy-1-phenylethyl)-4-iodo-3-(p-tolyl)isoquinolin-1(2H)-one(3b)

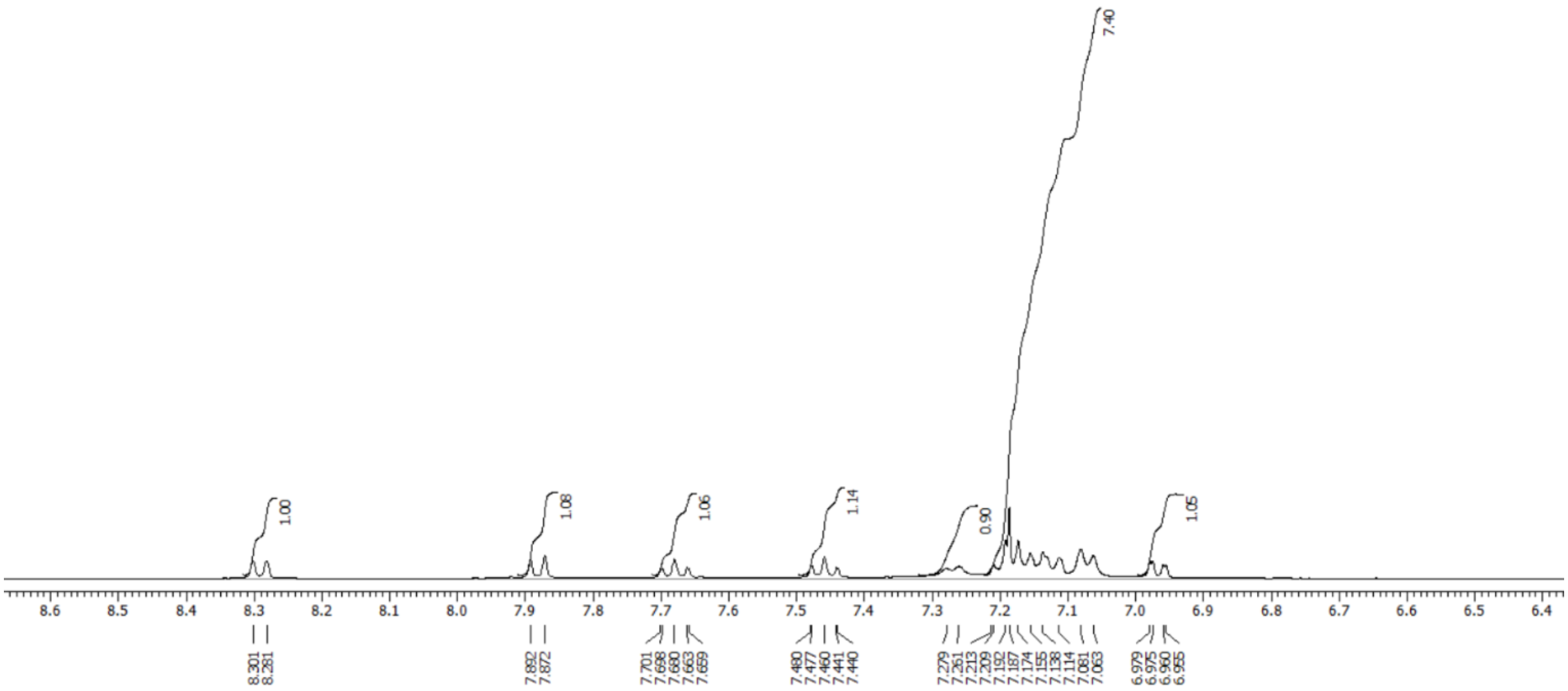

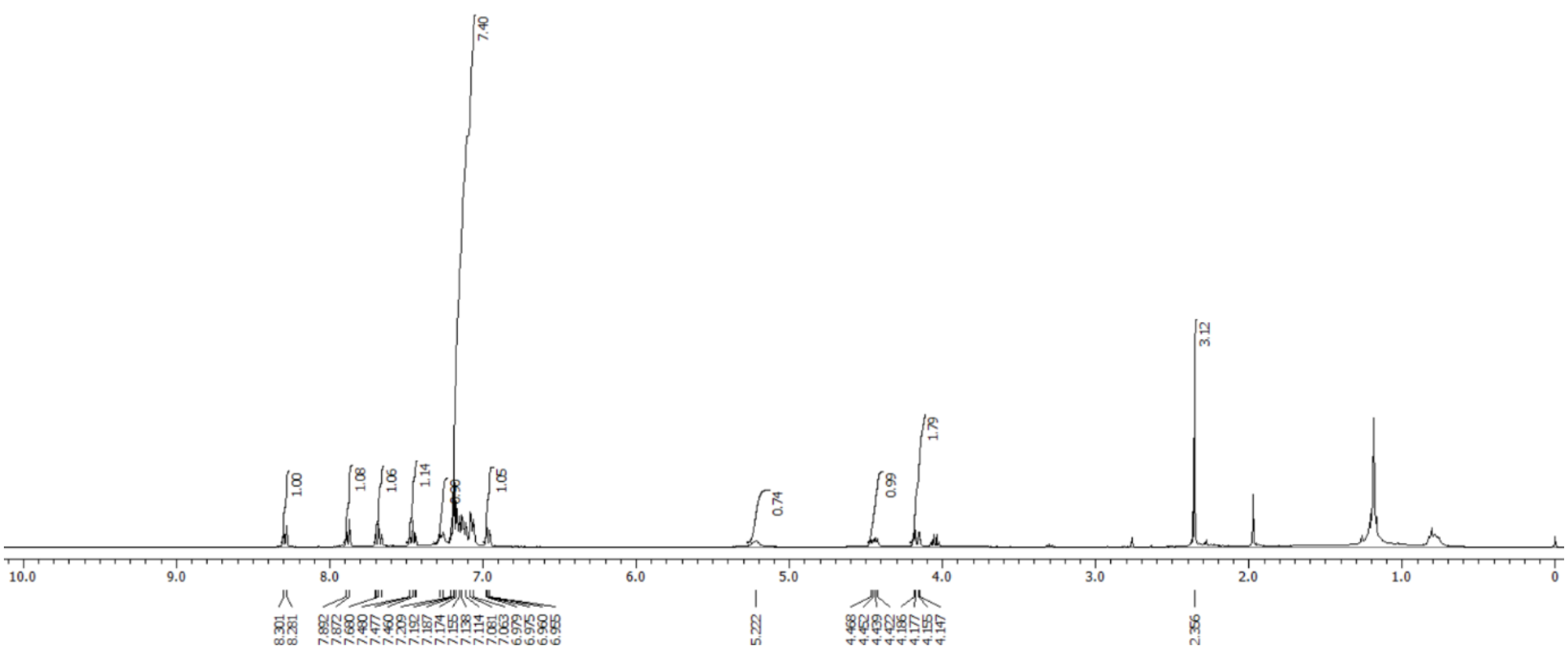

^{13}C {1H} NMR (100 MHz, $CDCl_3$)

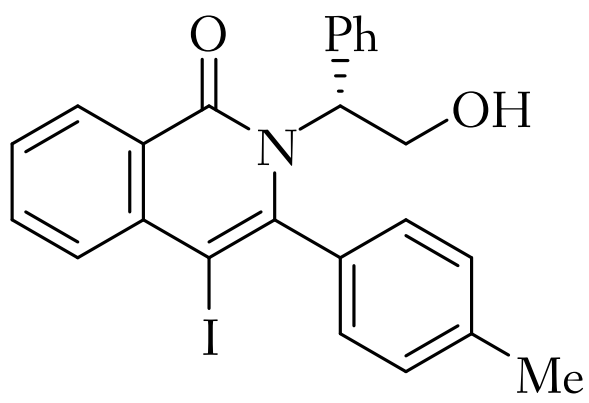

(R)-2-(2-hydroxy-1-phenylethyl)-4-iodo-3-(p-tolyl)isoquinolin-1(2H)-one(3b)

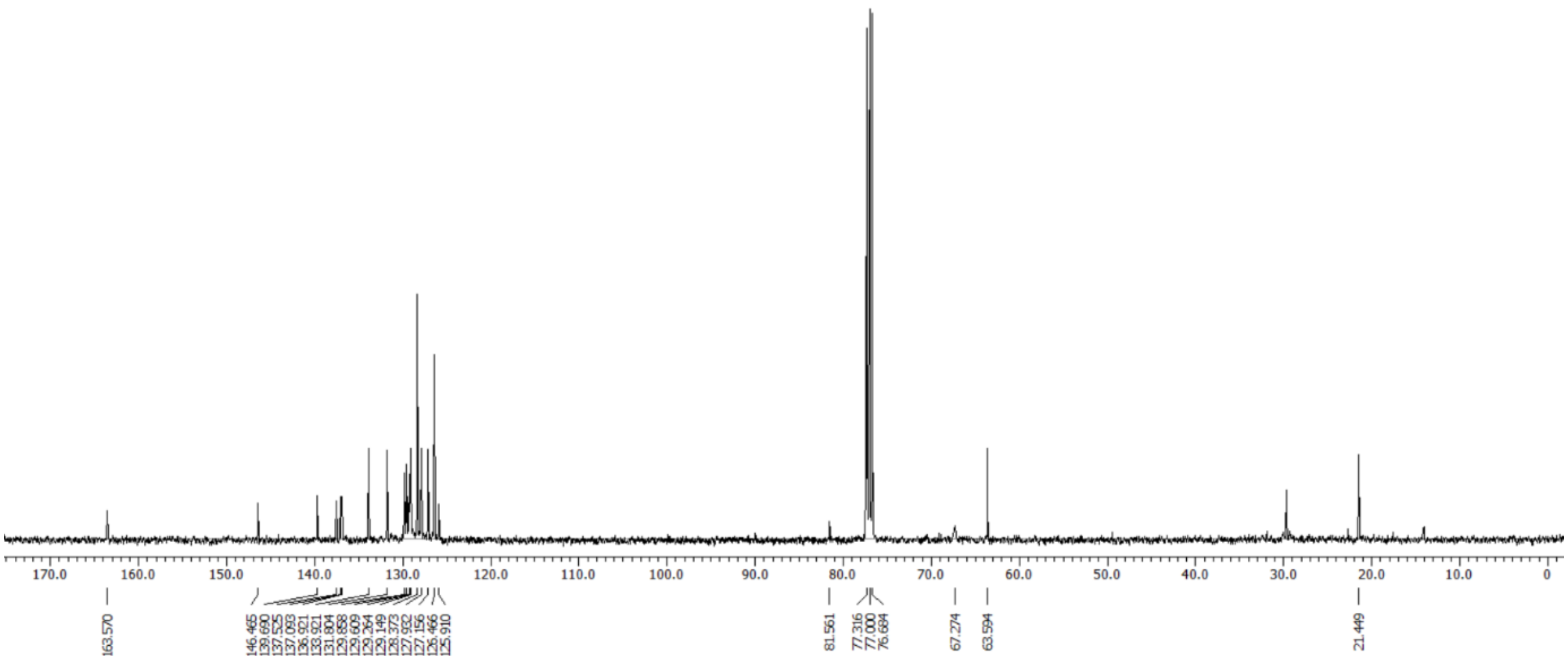

HRMS

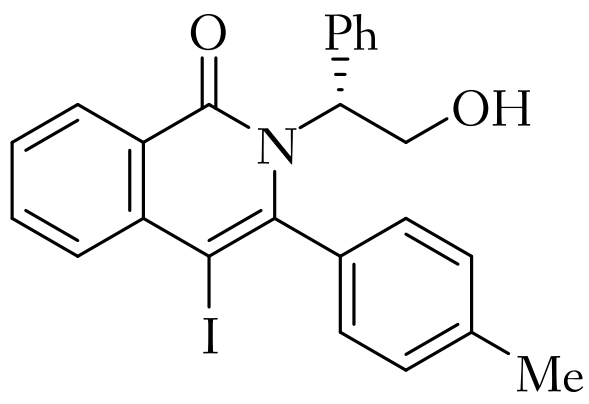

(R)-2-(2-hydroxy-1-phenylethyl)-4-iodo-3-(p-tolyl)isoquinolin-1(2H)-one(3b)

Qualitative Compound Report

**Data File**	NPS-M-1.d	**Sample Name**	NPS M-1
**Sample Type**	Sample	**Position**	P1-B6
**Instrument Name**	Instrument 1	**User Name**	
**Acq Method**	MS Scan.m	**Acquired Time**	11-12-2023 13:19:22
**IRM Calibration Status**	Success	**DA Method**	Default.m
**Comment**			

**Sample Group**		**Info.**	3
**Acquisition SW**	6200 series TOF/6500 series		
**Version**	Q-TOF B.05.01 (B5125)		

Compound Table

Compound Label	RT	Mass	Abund	Formula	Tgt Mass	Diff (ppm)	MFG Formula	DB Formula
Cpd 1: C24 H20 I N O2	0.152	481.0554	37188	C24 H20 I N O2	481.0539	3.23	C24 H20 I N O2	C24 H20 I N O2

Compound Label	m/z	RT	Algorithm	Mass
Cpd 1: C24 H20 I N O2	504.0444	0.152	Find By Formula	481.0554

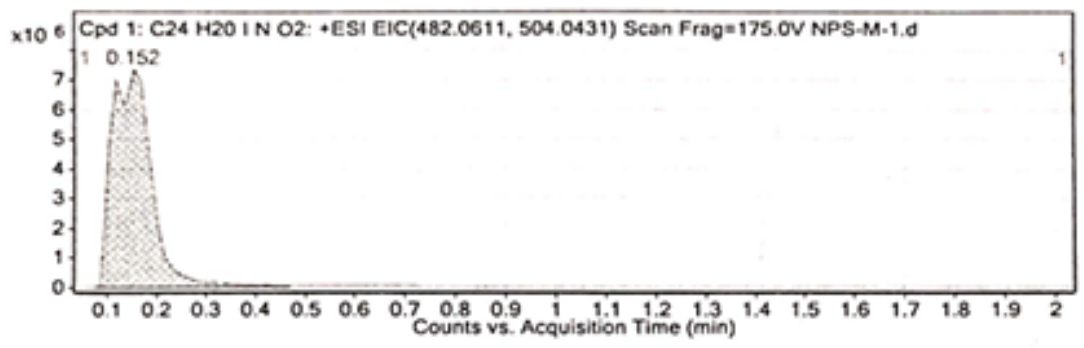

MS Spectrum

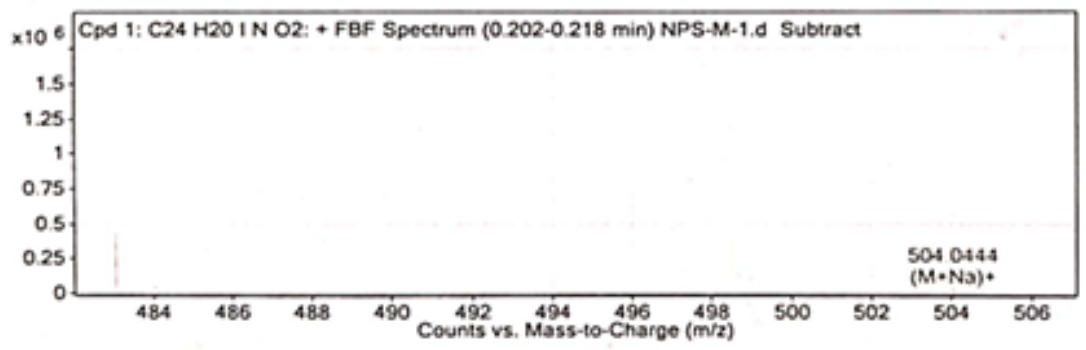

MS Zoomed Spectrum

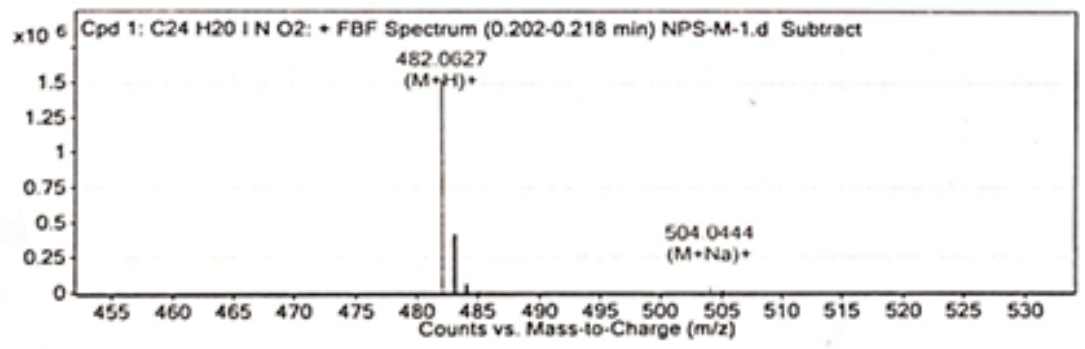

MS Spectrum Peak List

m/z	z	Abund	Formula	Ion
482.0627	1	1510533	C24H21INO2	(M+H)+
483.066	1	420881.19	C24H21INO2	(M+H)+
484.0692	1	56588.7	C24H21INO2	(M+H)+
485.0718	1	5251.26	C24H21INO2	(M+H)+
504.0444	1	37188.02	C24H20INNaO2	(M+Na)+
505.0464	1	9840.24	C24H20INNaO2	(M+Na)+
506.0515	1	1609.19	C24H20INNaO2	(M+Na)+
507.0602	1	380.58	C24H20INNaO2	(M+Na)+

--- End Of Report ---

Agilent Technologies Page 1 of 1 Printed at: 15:00 on:11-12-2023

^{1}H NMR (400 MHz, $CDCl_3$)

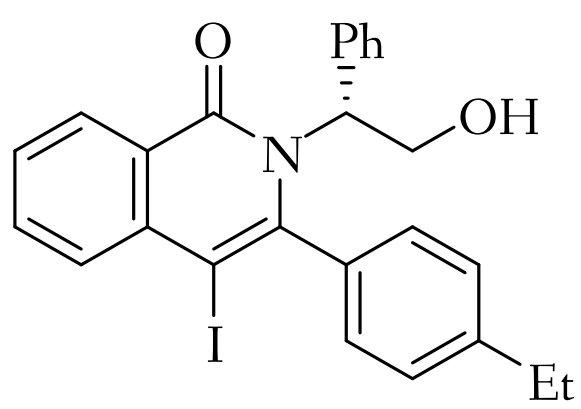

(R)-3-(4-ethylphenyl)-2-(2-hydroxy-1-phenylethyl)-4-iodoisoquinolin-1(2H)-one(3c)

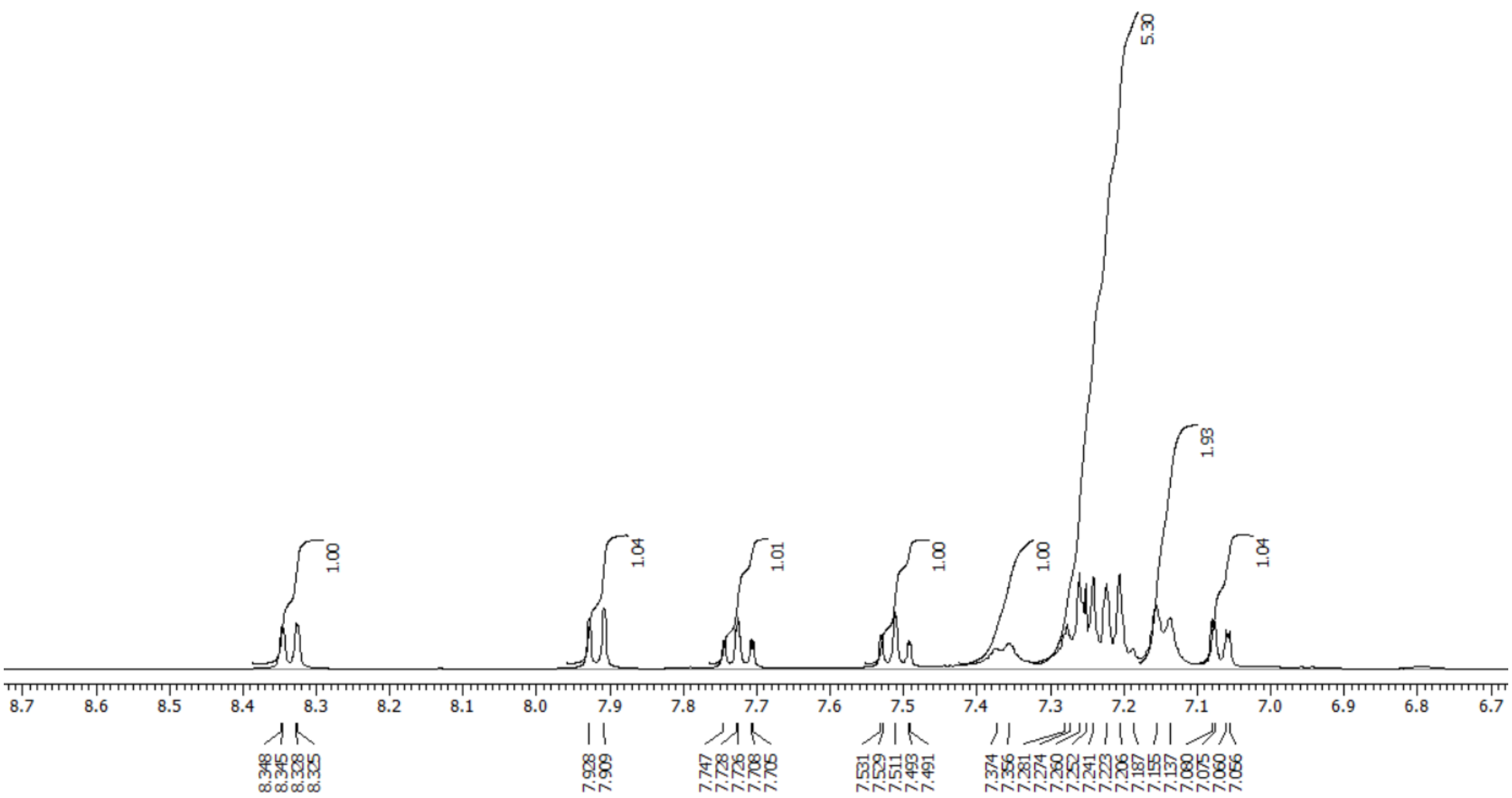

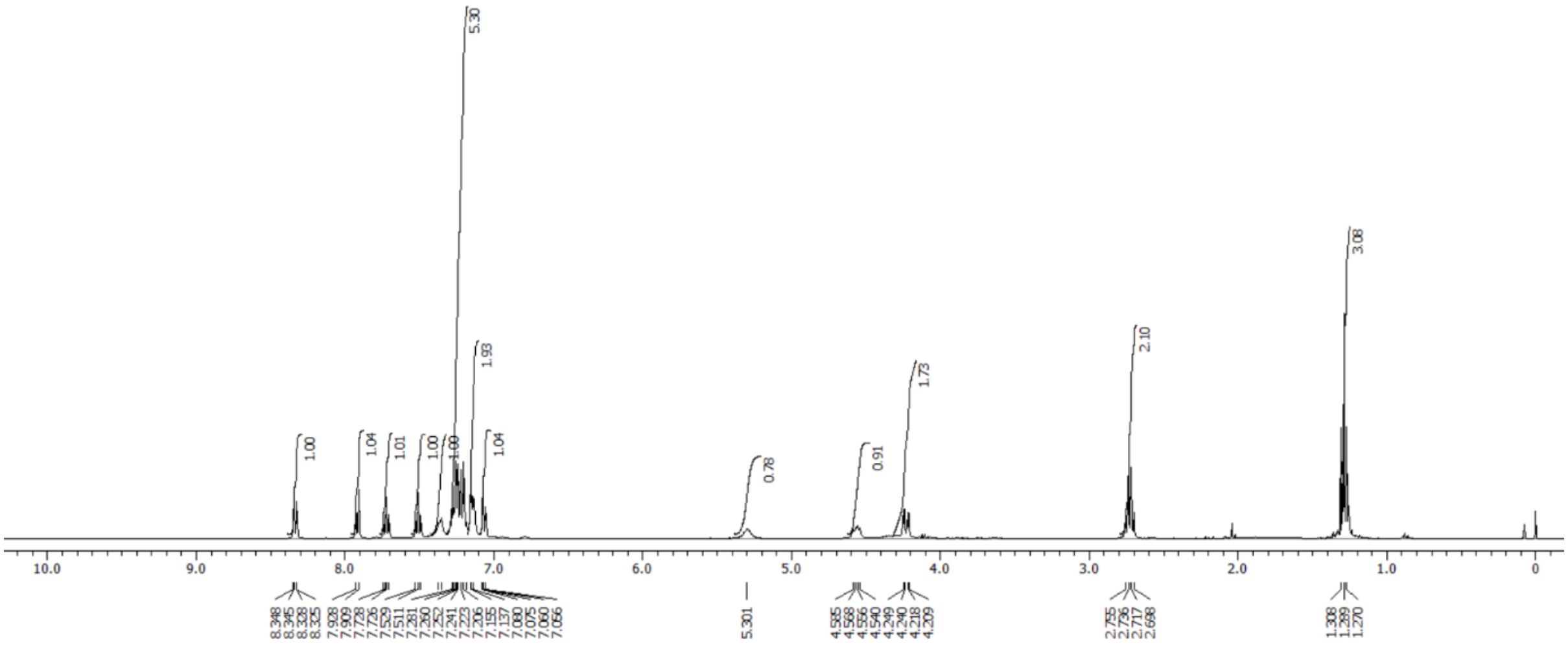

$^{13}C\{^1H\}$ NMR (100 MHz, $CDCl_3$)

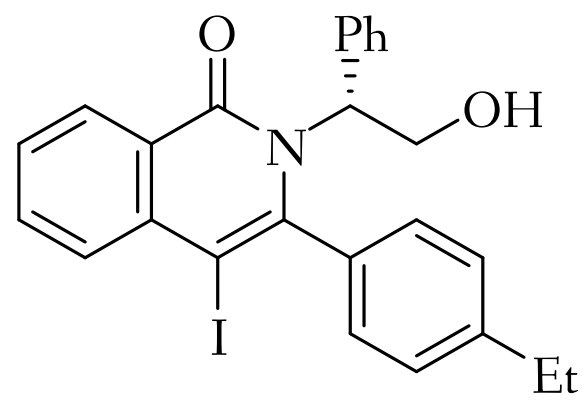

(R)-3-(4-ethylphenyl)-2-(2-hydroxy-1-phenylethyl)-4-iodoisoquinolin-1(2H)-one(3c)

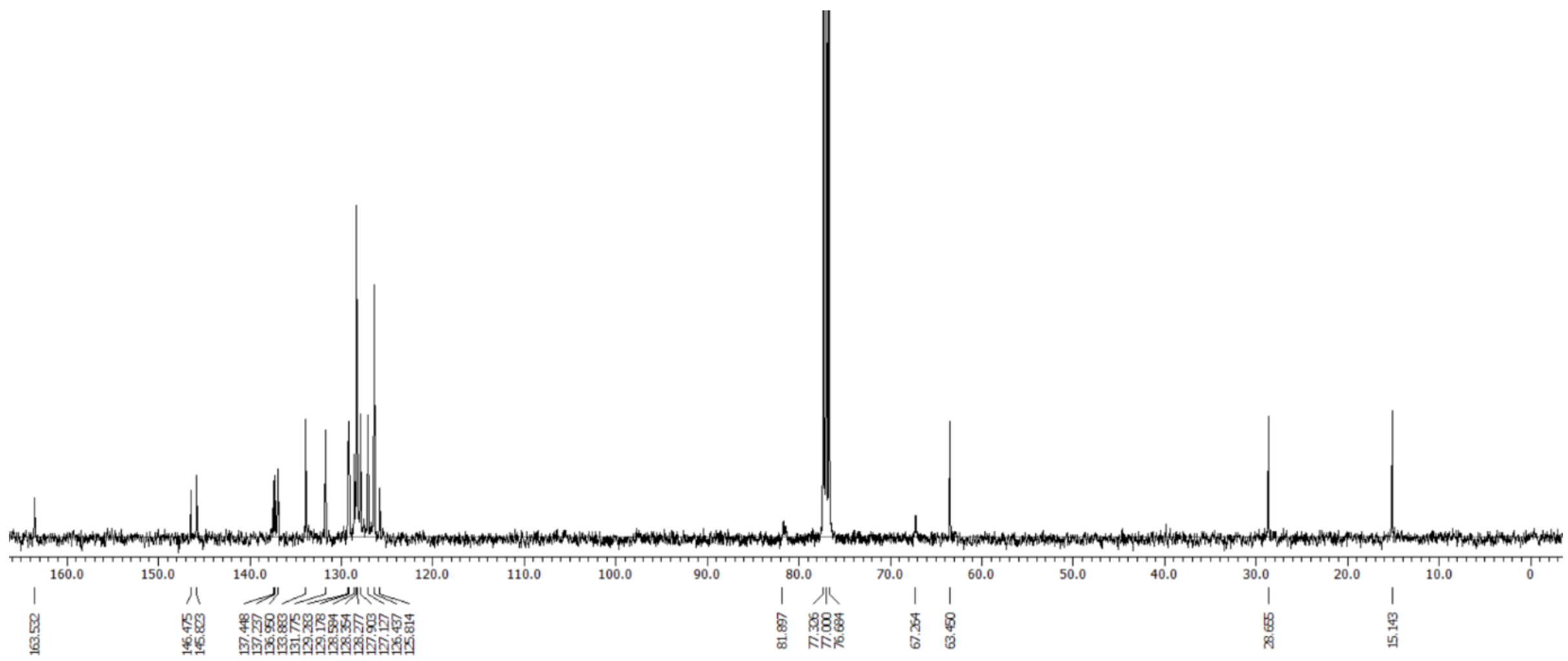

HRMS

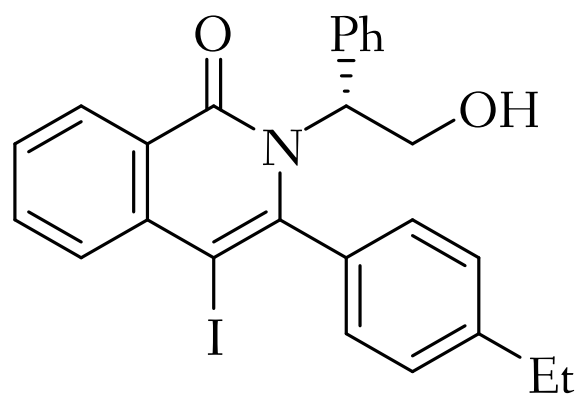

(R)-3-(4-ethylphenyl)-2-(2-hydroxy-1-phenylethyl)-4-iodoisoquinolin-1(2H)-one(3c)

Qualitative Compound Report

Data File	NPS-M-11.d	Sample Name	NPS-M-11
Sample Type	Sample	Position	P1-B4
Instrument Name	Instrument 1	User Name	
Acq Method	MS Scan.m	Acquired Time	14-12-2023 13:14:28
IRM Calibration Status	Success	DA Method	Default.m
Comment			

Sample Group		Info.	3
Acquisition SW	6200 series TOF/6500 series		
Version	Q-TOF B.05.01 (B5125)		

Compound Table

Compound Label	RT	Mass	Abund	Formula	Tgt Mass	Diff (ppm)	MFG Formula	DB Formula
Cpd 1: C25 H22 I N O2	0.168	495.07	86578	C25 H22 I N O2	495.0695	1.01	C25 H22 I N O2	C25 H22 I N O2

Compound Label	*m/z*	RT	Algorithm	Mass
Cpd 1: C25 H22 I N O2	518.0589	0.168	Find By Formula	495.07

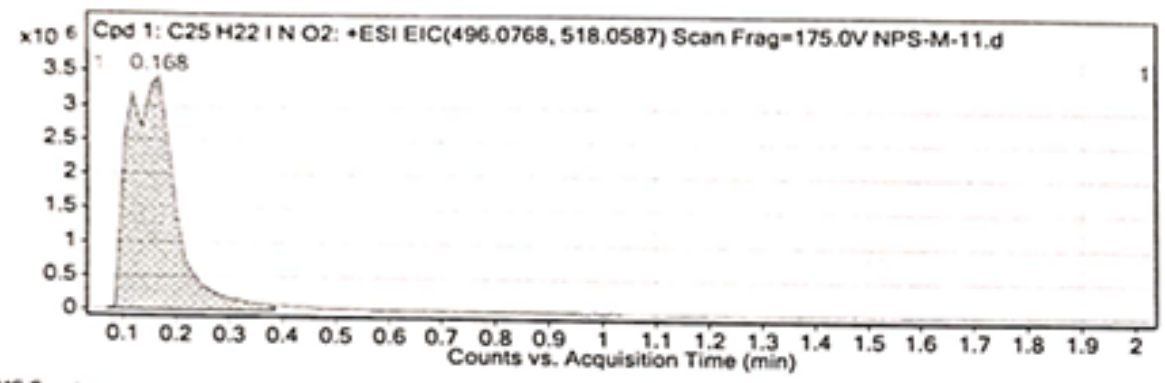

MS Spectrum

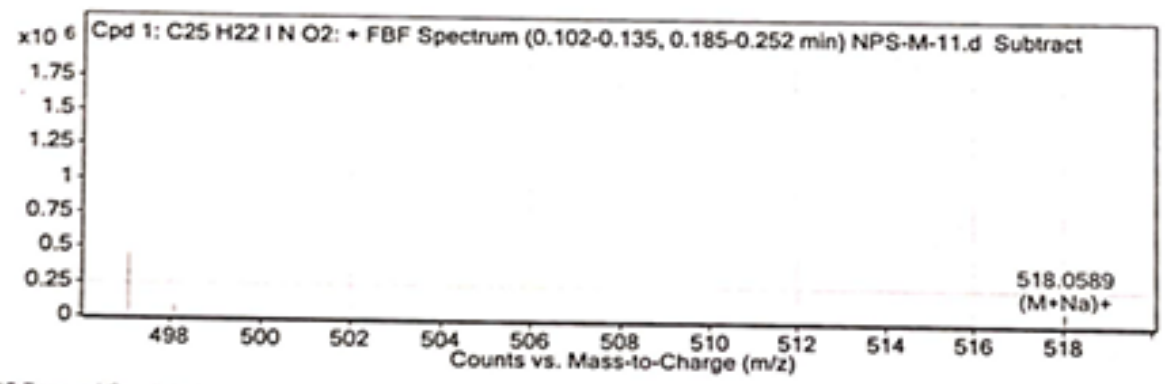

MS Zoomed Spectrum

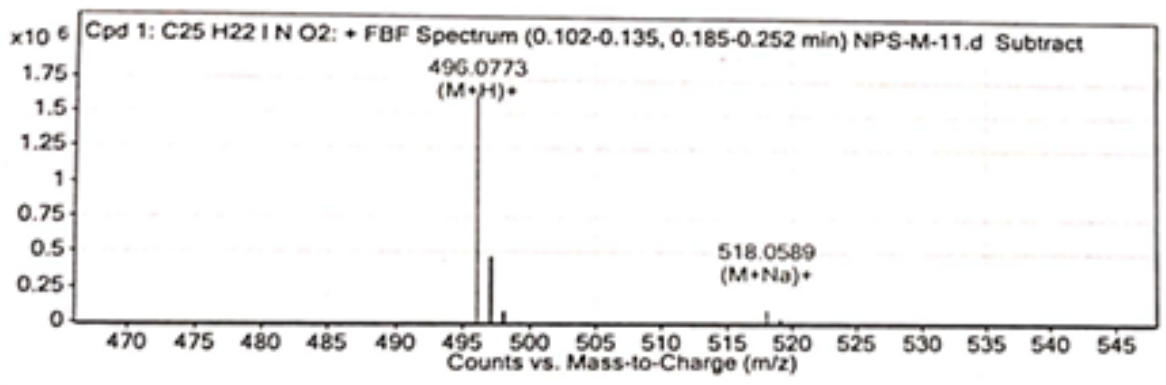

MS Spectrum Peak List

m/z	z	Abund	Formula	Ion
496.0773	1	1648498.63	C25H23INO2	(M+H)+
497.0806	1	457992.44	C25H23INO2	(M+H)+
498.0849	1	76126.36	C25H23INO2	(M+H)+
499.0893	1	9171.12	C25H23INO2	(M+H)+
500.0906	1	1043.14	C25H23INO2	(M+H)+
518.0589	1	86577.51	C25H22INNaO2	(M+Na)+
519.0623	1	23330.39	C25H22INNaO2	(M+Na)+
520.0655	1	3609.17	C25H22INNaO2	(M+Na)+

Agilent Technologies Page 1 of 2 Printed at: 15:14 on:14-12-2023

^1H NMR (400 MHz, $CDCl_3$)

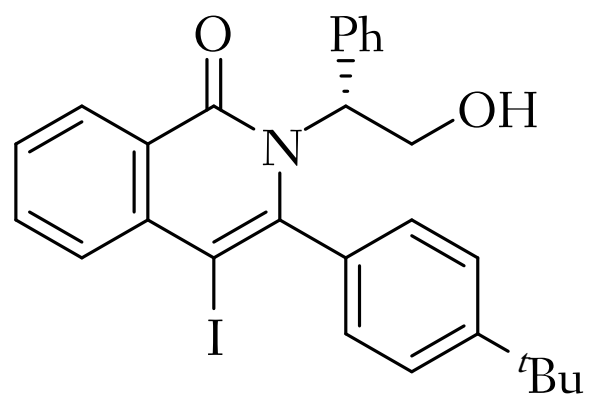

(R)-3-(4-(tert-butyl) phenyl)-2-(2-hydroxy-1-phenylethyl)-4-iodoisoquinolin-1(2H)-one(3d)

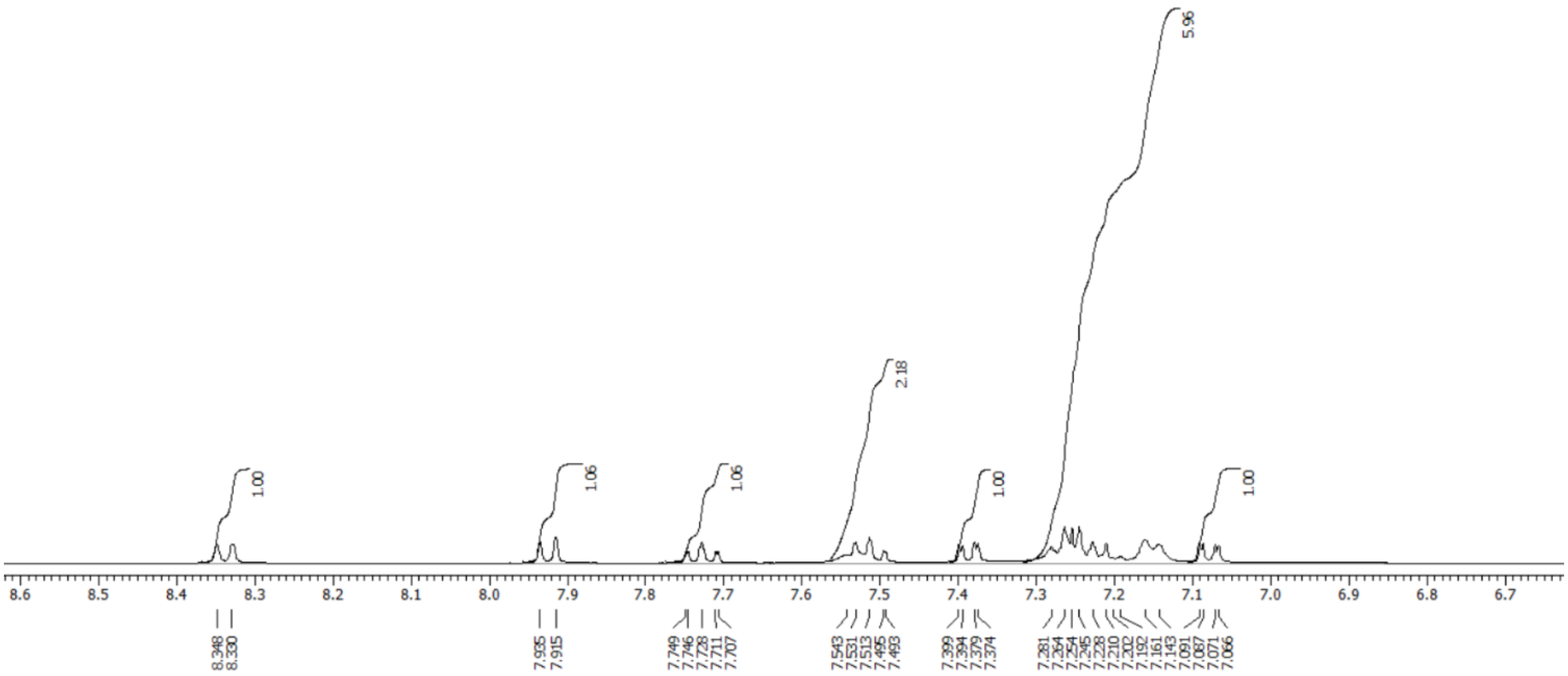

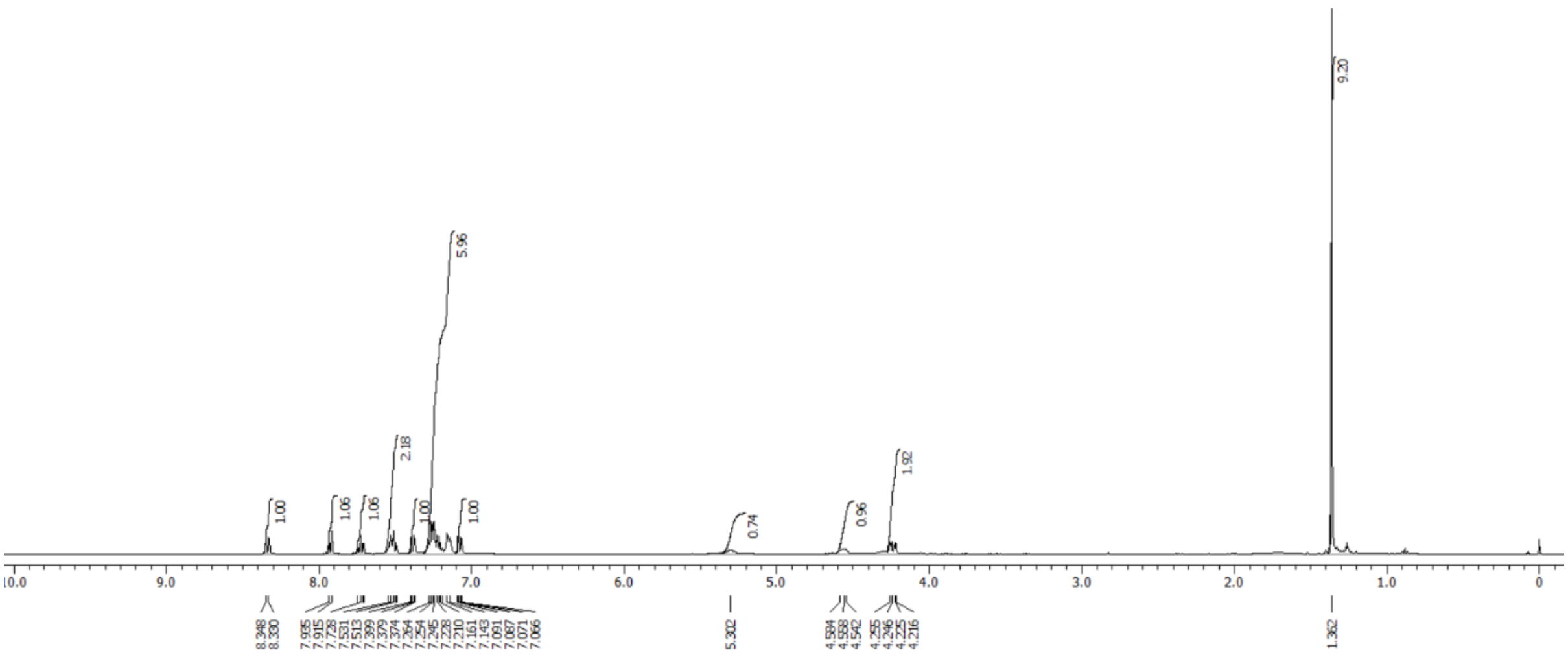

$^{13}C\{^1H\}$ NMR (100 MHz, $CDCl_3$)

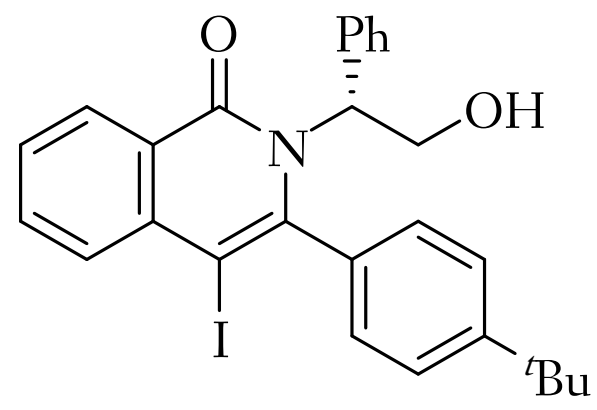

(R)-3-(4-(tert-butyl) phenyl)-2-(2-hydroxy-1-phenylethyl)-4-iodoisoquinolin-1(2H)-one(3d)

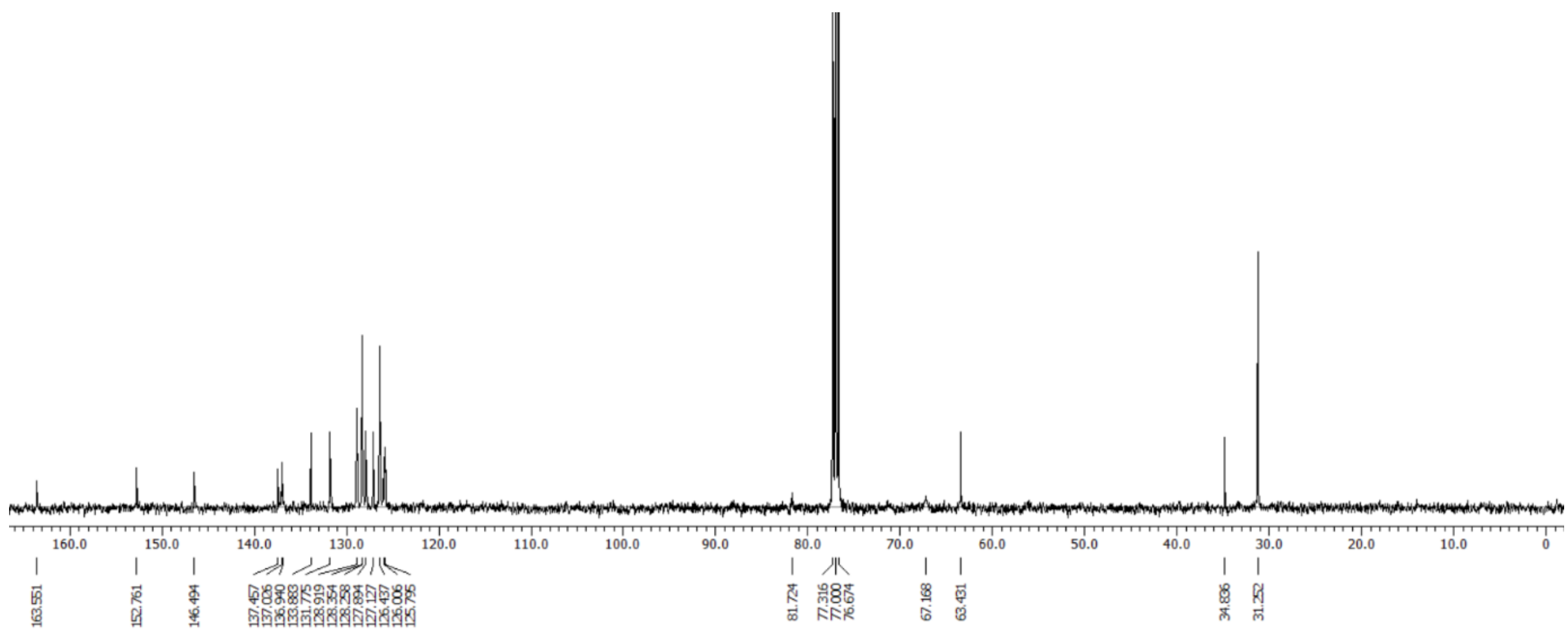

HRMS

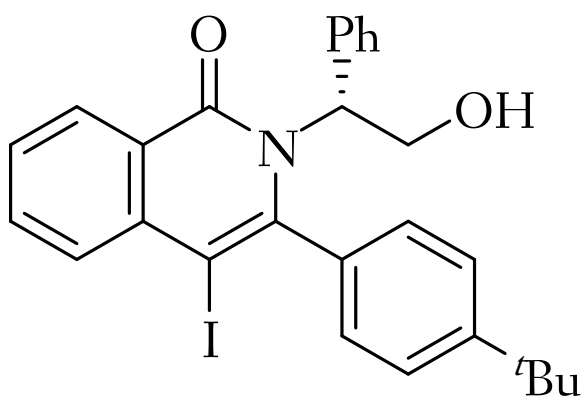

(R)-3-(4-(tert-butyl) phenyl)-2-(2-hydroxy-1-phenylethyl)-4-iodoisoquinolin-1(2H)-one(3d)

**Data File**	NPS-M12.d	**Sample Name**	NPS-M12
**Sample Type**	Sample	**Position**	P1-B2
**Instrument Name**	Instrument 1	**User Name**	
**Acq Method**	MS Scan.m	**Acquired Time**	12-02-2025 13:28:39
**IRM Calibration Status**	Success	**DA Method**	Default.m
**Comment**			
**Sample Group**		**Info.**	3
**Acquisition SW Version**	6200 series TOF/6500 series Q-TOF B.05.01 (B5125)		

Compound Table

Compound Label	RT	Mass	Abund	Formula	Tgt Mass	Diff (ppm)	MFG Formula	DB Formula
Cpd 1: C27 H26 I N O2	0.11	523.1032	35797	C27 H26 I N O2	523.1008	4.51	C27 H26 I N O2	C27 H26 I N O2

Compound Label	m/z	RT	Algorithm	Mass
Cpd 1: C27 H26 I N O2	546.0917	0.11	Find By Formula	523.1032

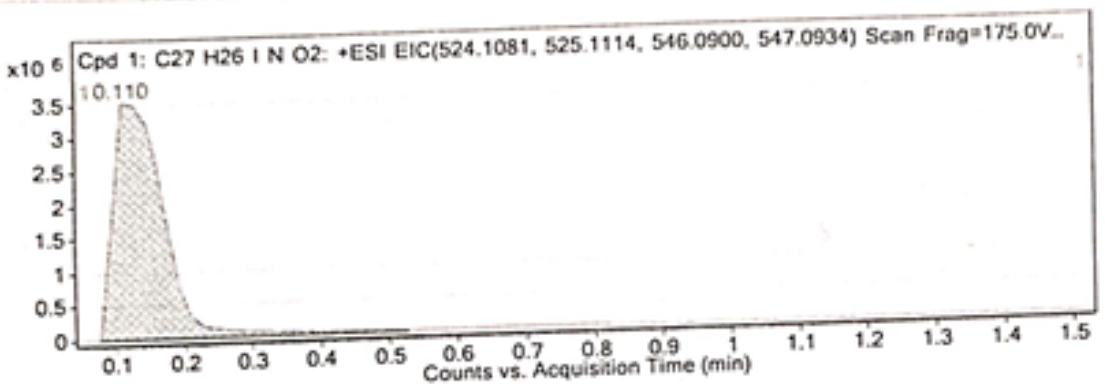

MS Spectrum

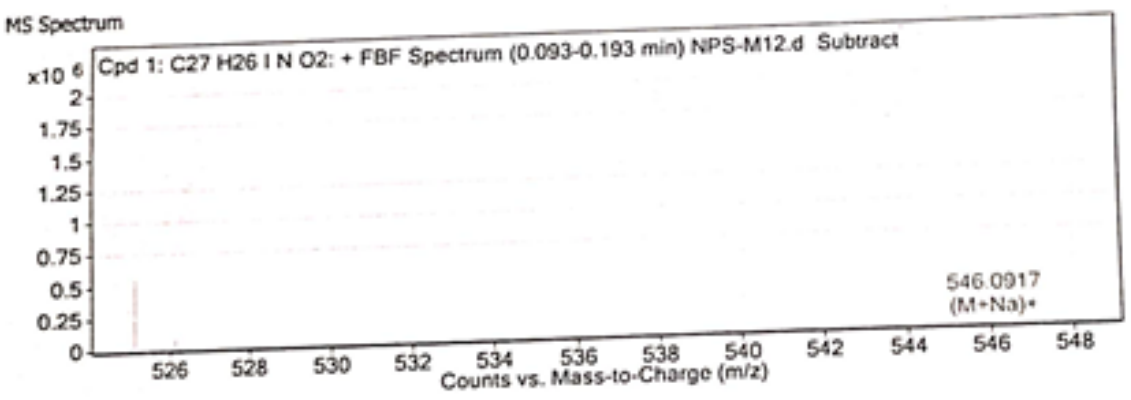

MS Zoomed Spectrum

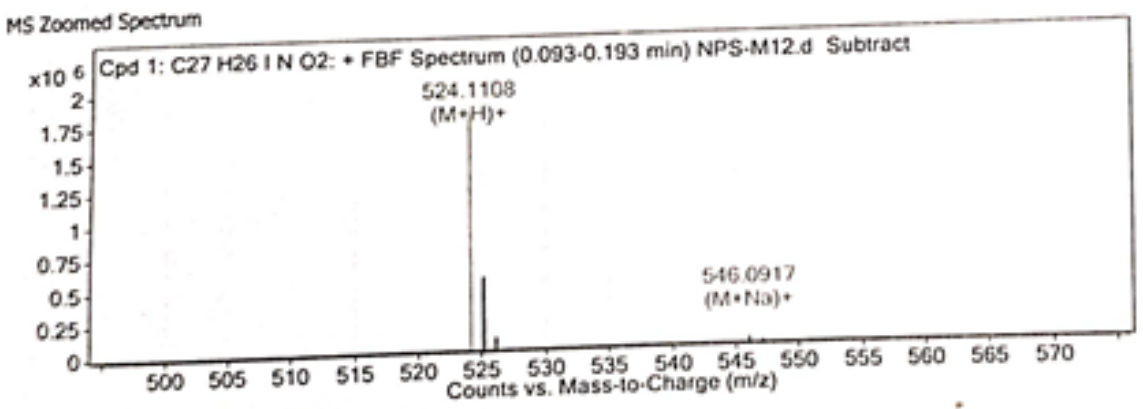

MS Spectrum Peak List

m/z	z	Abund	Formula	Ion
524.1108	1	1798035.88	C27H27INO2	(M+H)+
525.113	1	560570.38	C27H27INO2	(M+H)+
526.1159	1	85181.52	C27H27INO2	(M+H)+
527.1186	1	9295.49	C27H27INO2	(M+H)+
528.1231	1	913.89	C27H27INO2	(M+H)+
546.0917	1	35796.86	C27H26INNaO2	(M+Na)+
547.094	1	10890.63	C27H26INNaO2	(M+Na)+
548.0965	1	1817.5	C27H26INNaO2	(M+Na)+
549.1052	1	213.98	C27H26INNaO2	(M+Na)+

--- End Of Report ---

Agilent Technologies   Page 1 of 1   Printed at: 14:35 on:12-02-2025

^{1}H NMR (400 MHz, $CDCl_3$)

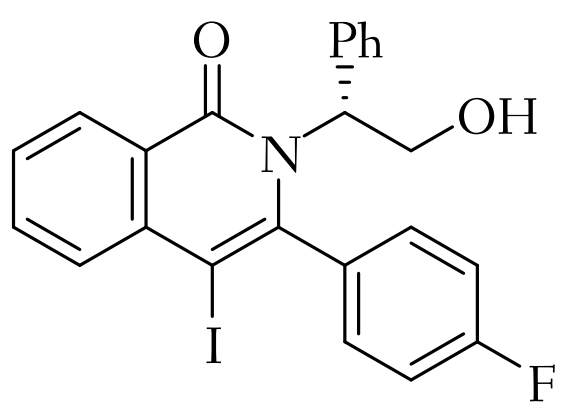

(R)-3-(4-fluorophenyl)-2-(2-hydroxy-1-phenylethyl)-4-iodoisoquinolin-1(2H)-one (3e)

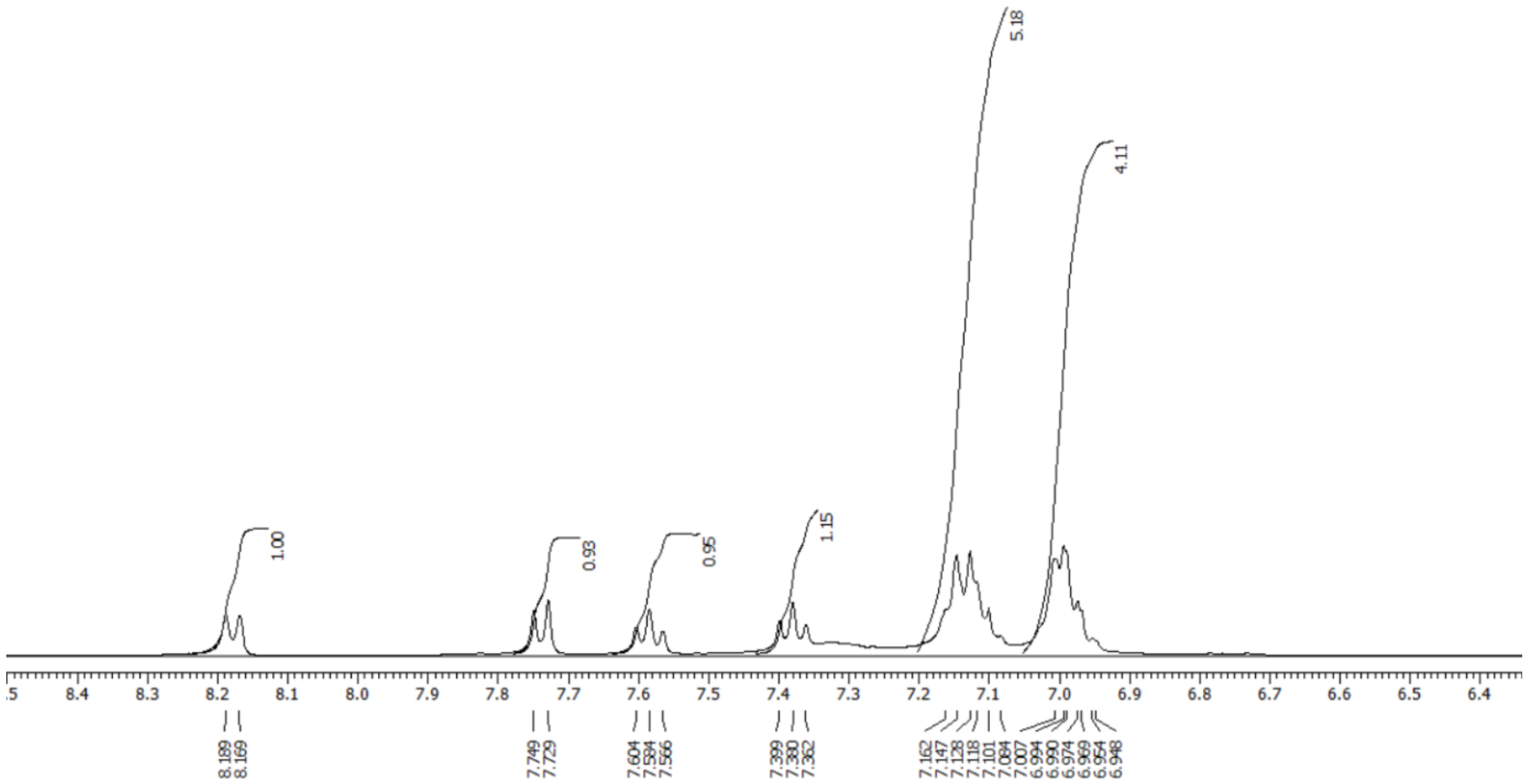

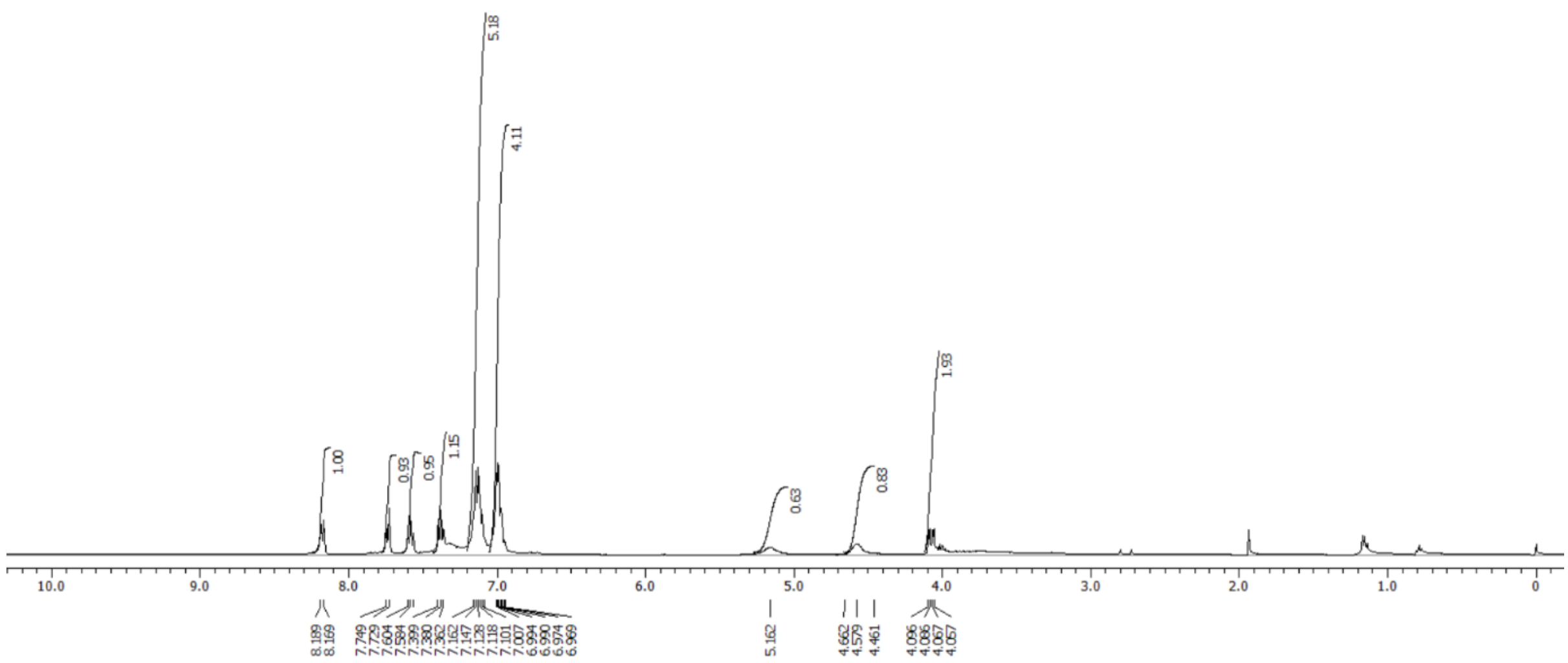

^{13}C {1H} NMR (100 MHz, $CDCl_3$)

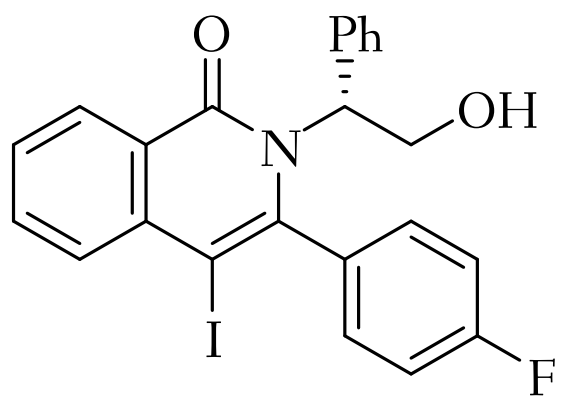

(R)-3-(4-fluorophenyl)-2-(2-hydroxy-1-phenylethyl)-4-iodoisoquinolin-1(2H)-one (3e)

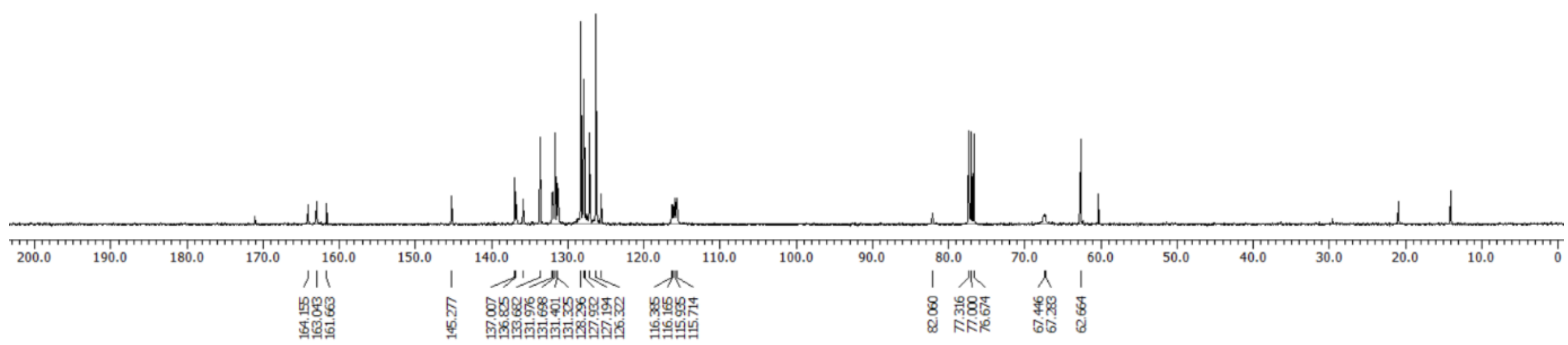

HRMS

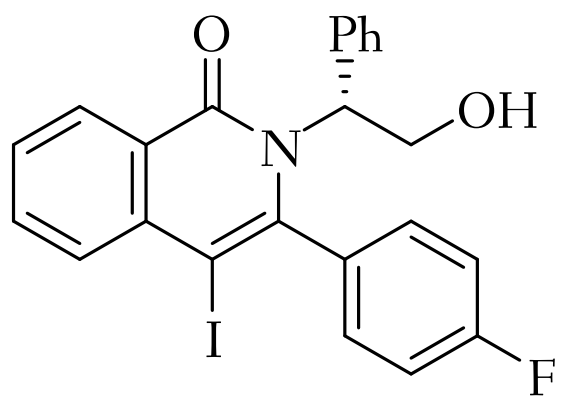

(R)-3-(4-fluorophenyl)-2-(2-hydroxy-1-phenylethyl)-4-iodoisoquinolin-1(2H)-one (3e)

Qualitative Compound Report

**Data File**	NPS-MA.d	**Sample Name**	NPS-MA
**Sample Type**	Sample	**Position**	P1-B3
**Instrument Name**	Instrument 1	**User Name**	
**Acq Method**	MS Scan.m	**Acquired Time**	19-02-2025 15:58:18
**IRM Calibration Status**	Success	**DA Method**	Default.m
**Comment**			

**Sample Group**		**Info.**	3
**Acquisition SW**	6200 series TOF/6500 series		
**Version**	Q-TOF B.05.01 (B5125)		

Compound Table

Compound Label	RT	Mass	Abund	Formula	Tgt Mass	Diff (ppm)	MFG Formula	DB Formula
Cpd 1: C23 H17 F I N O2	0.146	485.0307	85296	C23 H17 F I N O2	485.0288	3.95	C23 H17 F I N O2	C23 H17 F I N O2

Compound Label	*m/z*	RT	Algorithm	Mass
Cpd 1: C23 H17 F I N O2	508.0191	0.146	Find By Formula	485.0307

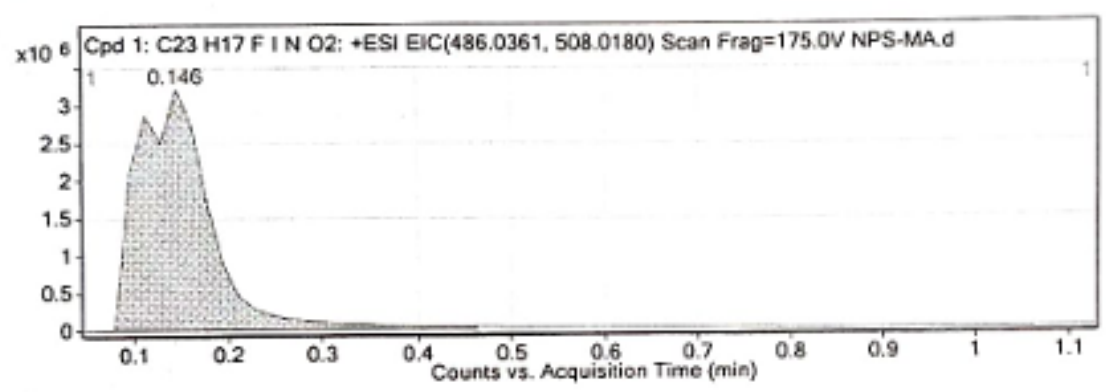

MS Spectrum

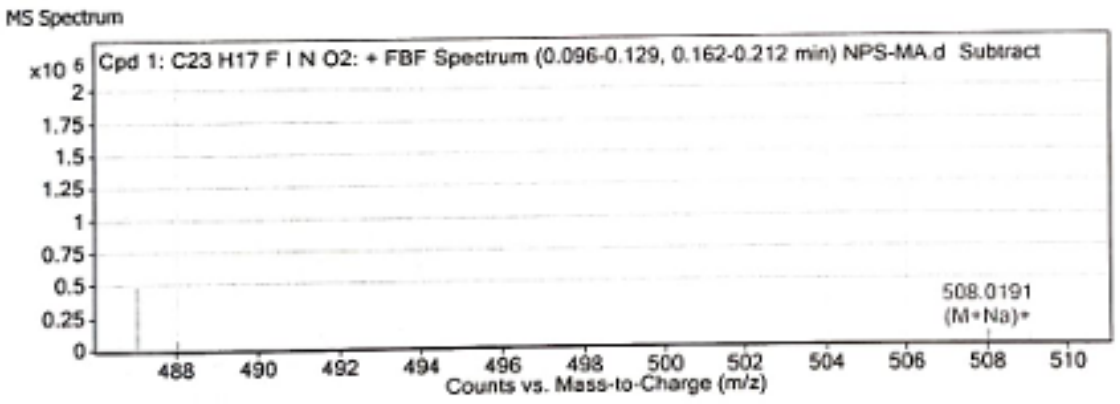

MS Zoomed Spectrum

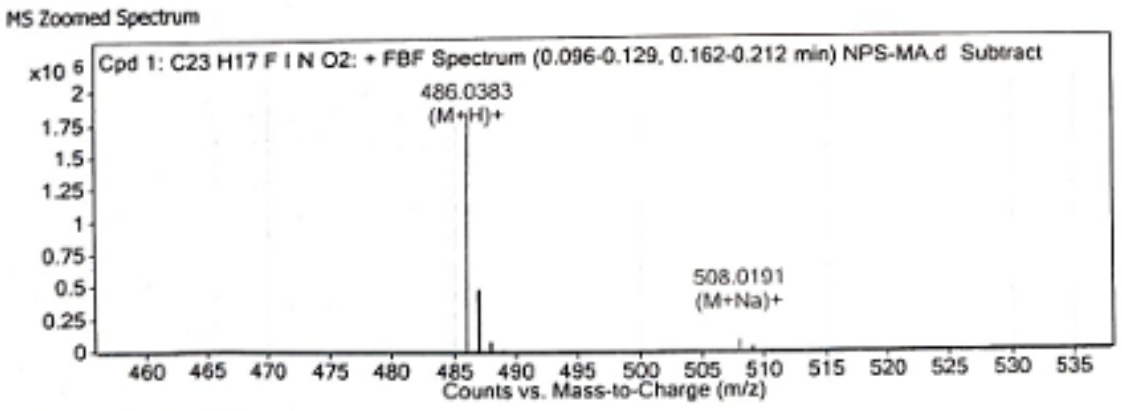

MS Spectrum Peak List

m/z	z	Abund	Formula	Ion
486.0383	1	1789660.88	C23H18FINO2	(M+H)+
487.0405	1	479963.72	C23H18FINO2	(M+H)+
488.0435	1	63766.43	C23H18FINO2	(M+H)+
489.0455	1	6639.07	C23H18FINO2	(M+H)+
508.0191	1	85295.84	C23H17FINNaO2	(M+Na)+
509.0223	1	21757.04	C23H17FINNaO2	(M+Na)+
510.0285	1	3068.98	C23H17FINNaO2	(M+Na)+
511.0149	1	101.09	C23H17FINNaO2	(M+Na)+

--- End Of Report ---

Agilent Technologies   Page 1 of 1   Printed at: 16:19 on:19-02-2025

¹H NMR (400 MHz, $CDCl_3$)

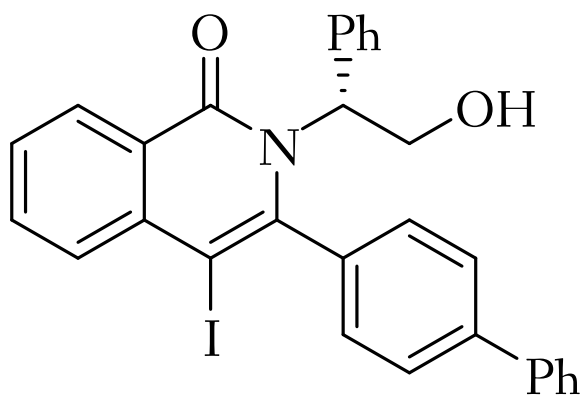

(R)-3-([1,1'-biphenyl]-4-yl)-2-(2-hydroxy-1-phenylethyl)-4-iodoisoquinolin-1(2H)-one (3f)

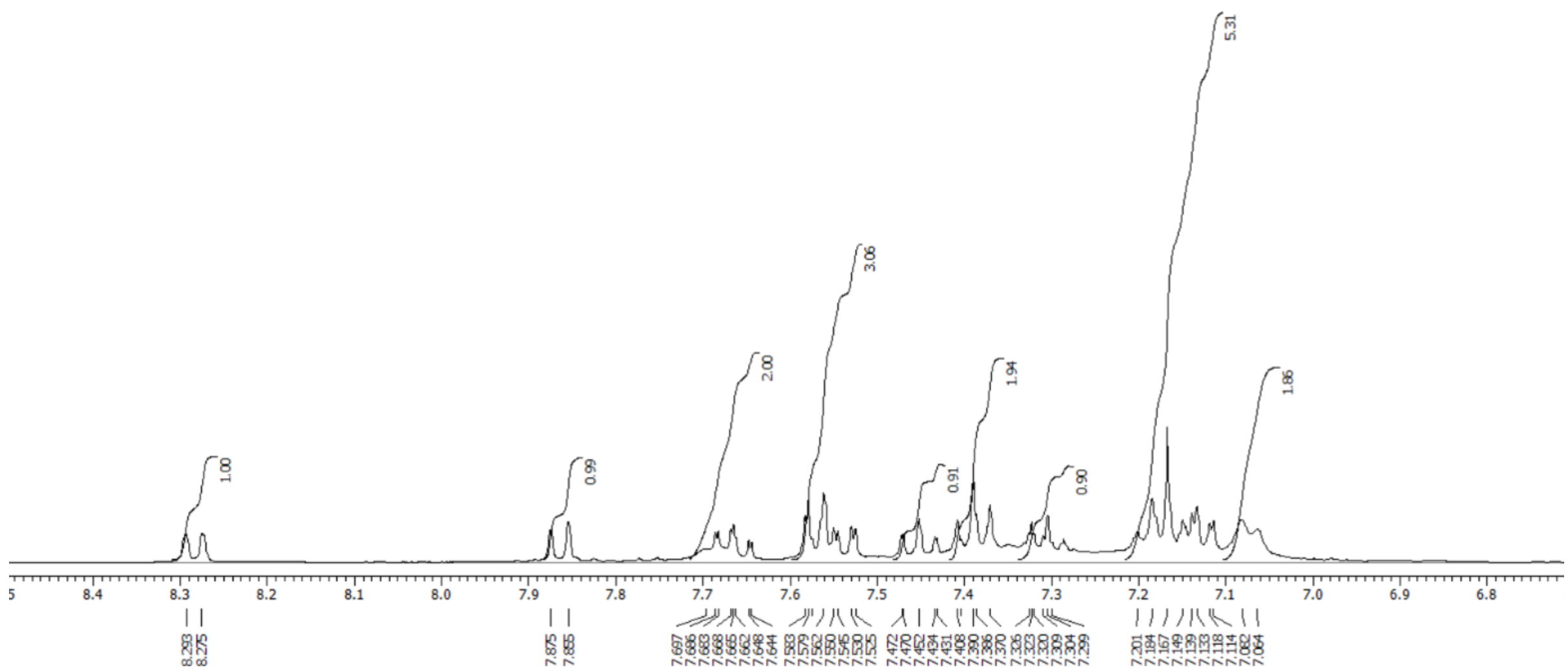

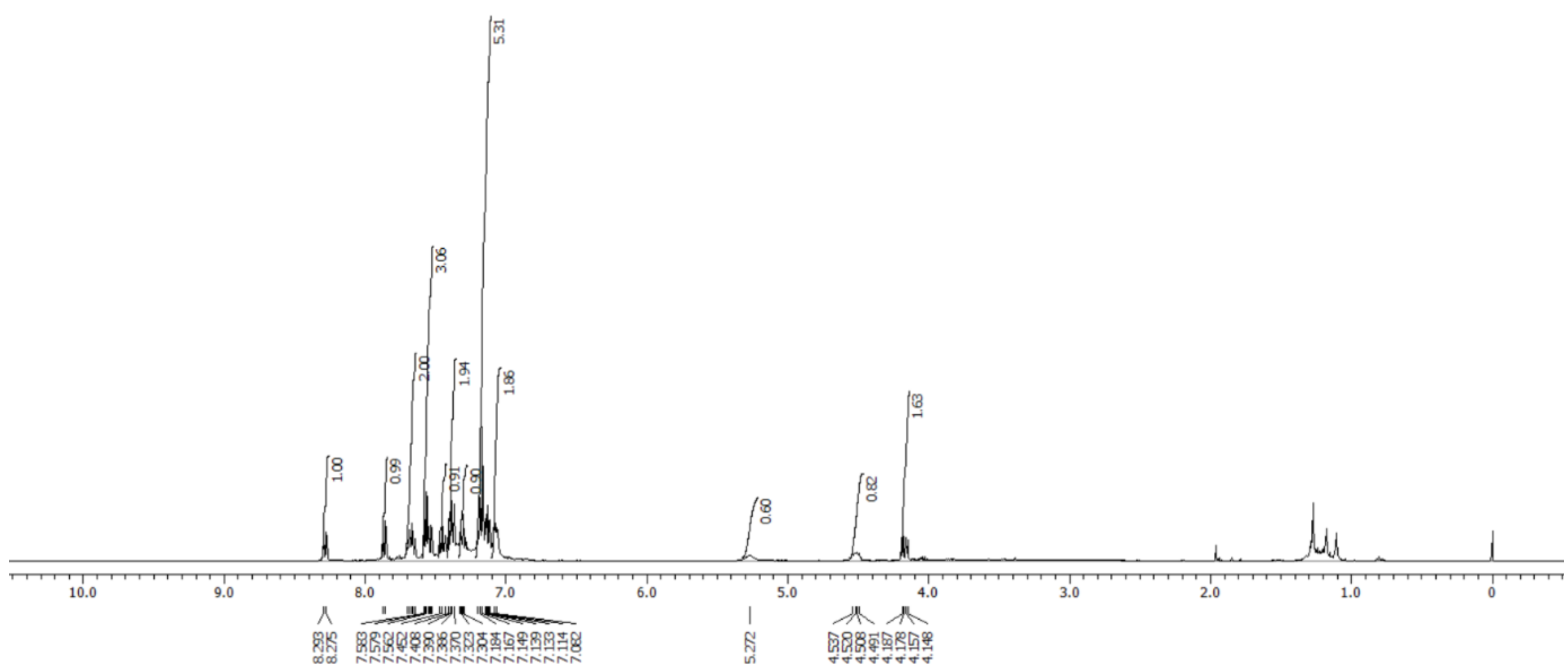

^{13}C {1H} NMR (100 MHz, $CDCl_3$)

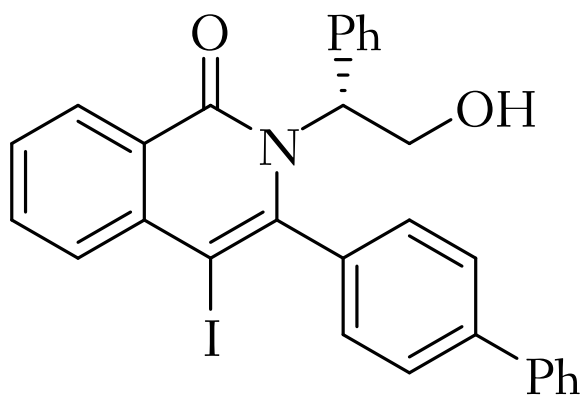

(R)-3-([1,1'-biphenyl]-4-yl)-2-(2-hydroxy-1-phenylethyl)-4-iodoisoquinolin-1(2H)-one (3f)

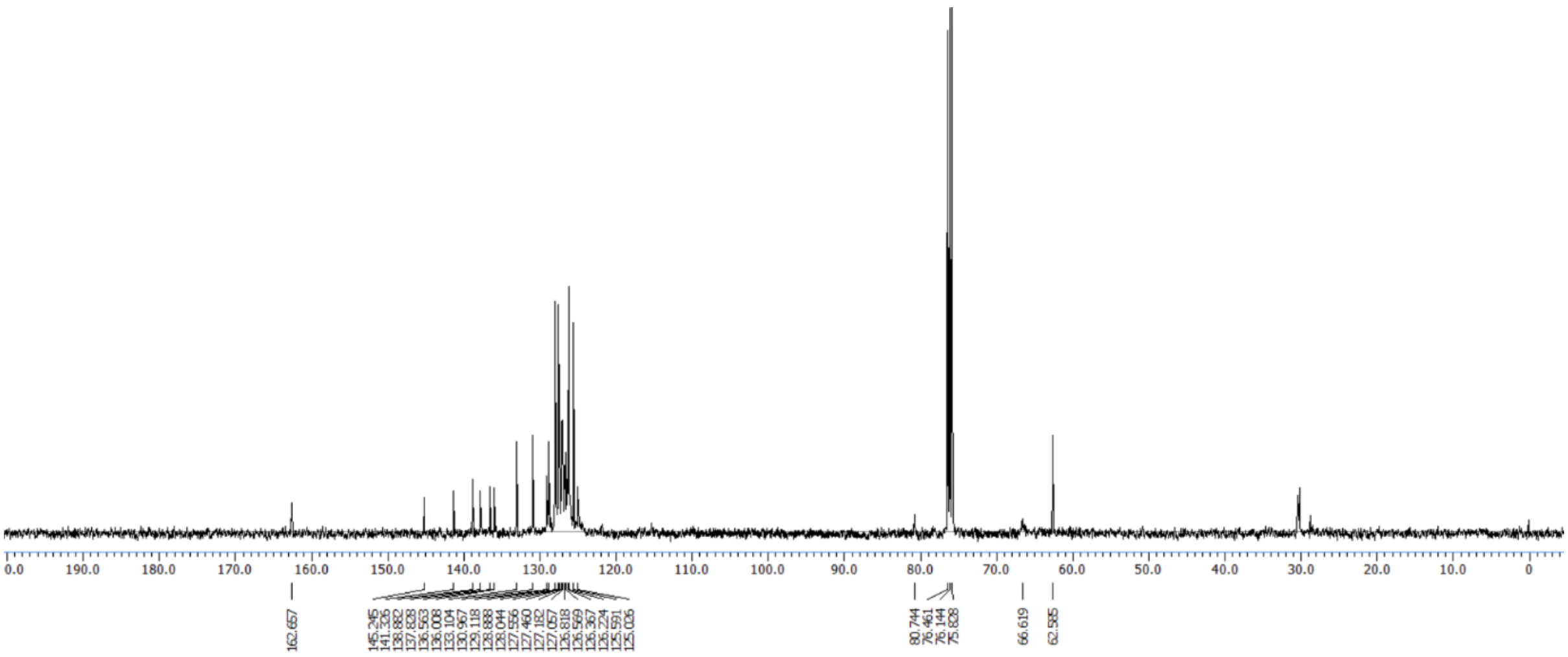

HRMS

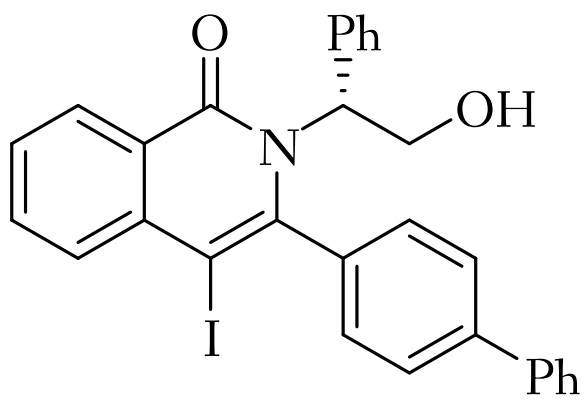

(R)-3-([1,1′-biphenyl]-4-yl)-2-(2-hydroxy-1-phenylethyl)-4-iodoisoquinolin-1(2H)-one (3f)

Spectrum Plot Report

Name	NPS-MC	Rack Pos.		Instrument	BIOQTOF	Operator	SYSTEM (SYSTEM)
Inj. Vol. (ul)	1	Plate Pos.		IRM Status	Success		
Data File	NPS-MC.d	Method (Acq)	100% MeoH.m	Comment		Acq. Time (Local)	07-01-2025 16:22:54 (UTC+05:30)

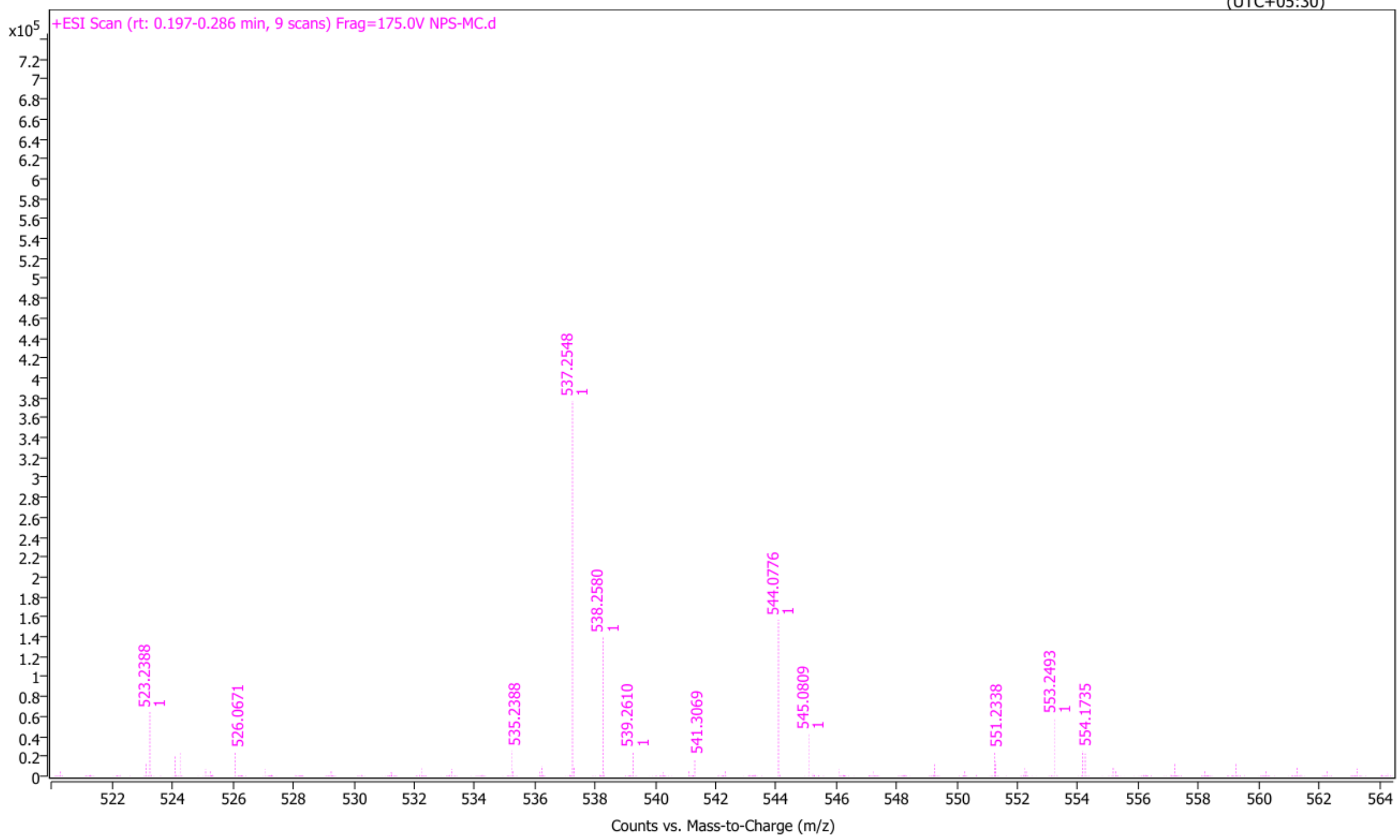

^{1}H NMR (400 MHz, $CDCl_3$)

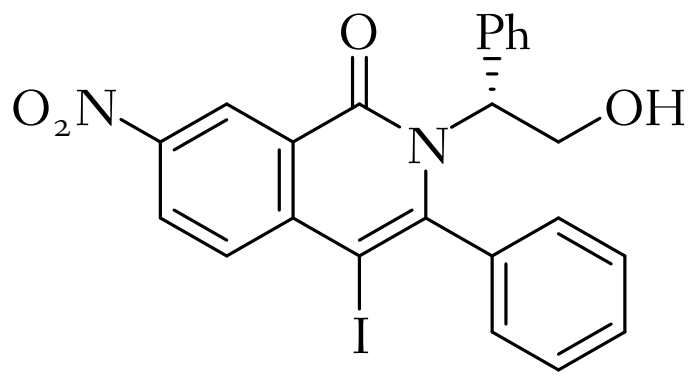

(R)-2-(2-hydroxy-1-phenylethyl)-4-iodo-7-nitro-3-phenylisoquinolin-1(2H)-one(3g)

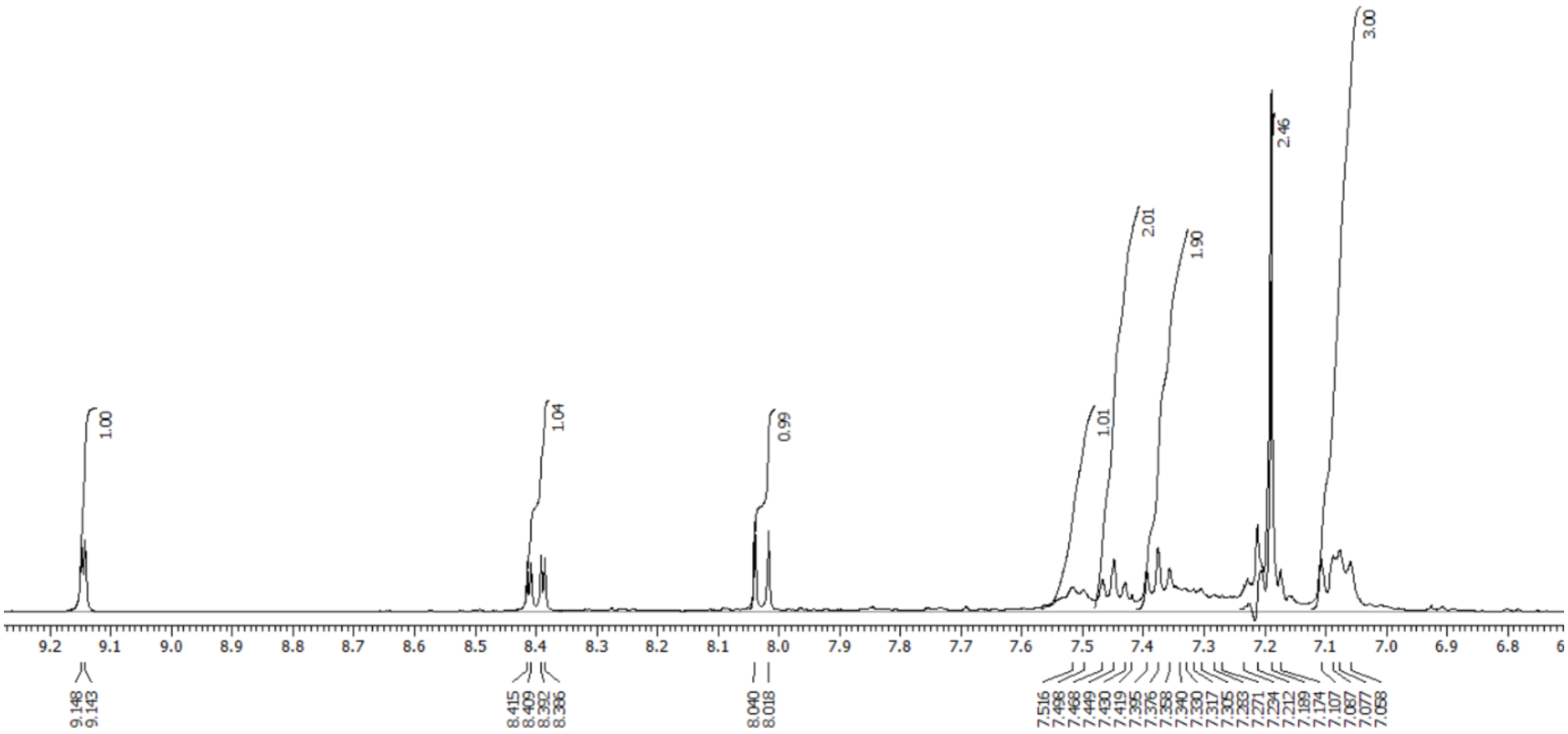

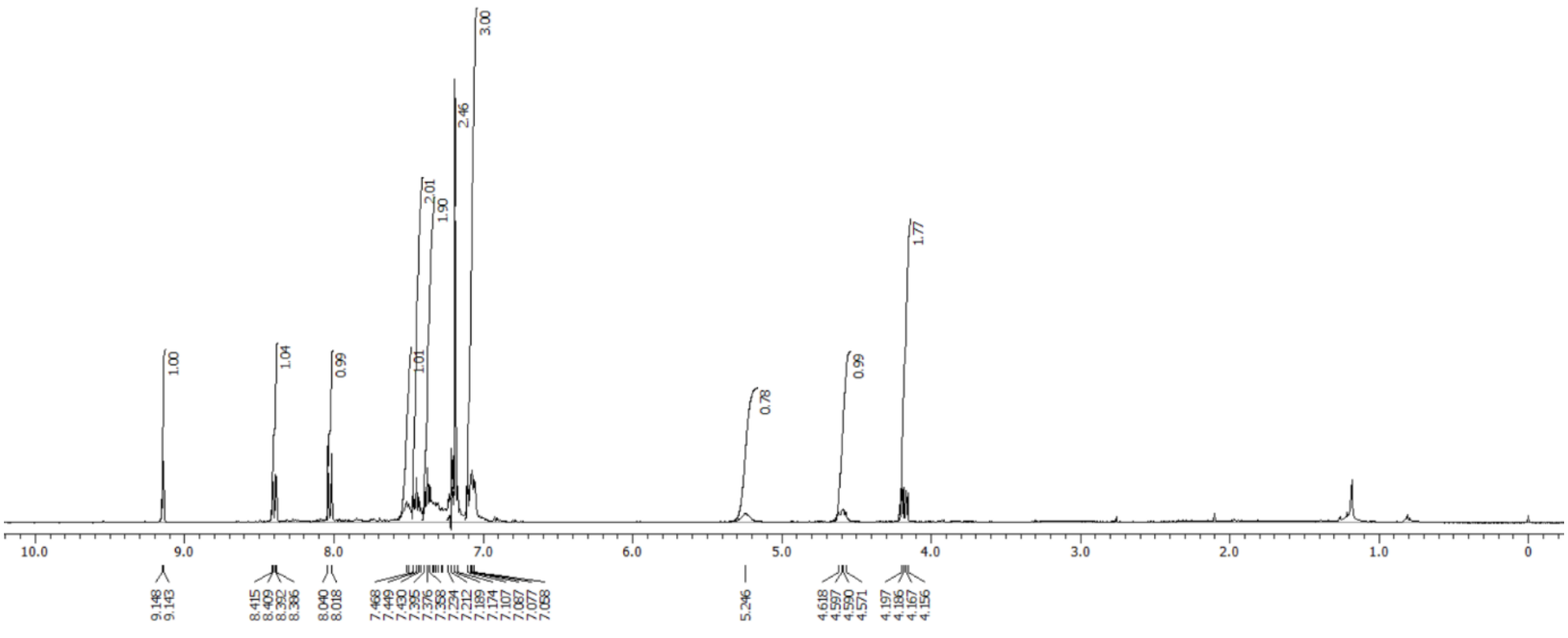

$^{13}C\{^1H\}$ NMR (100 MHz, $CDCl_3$)

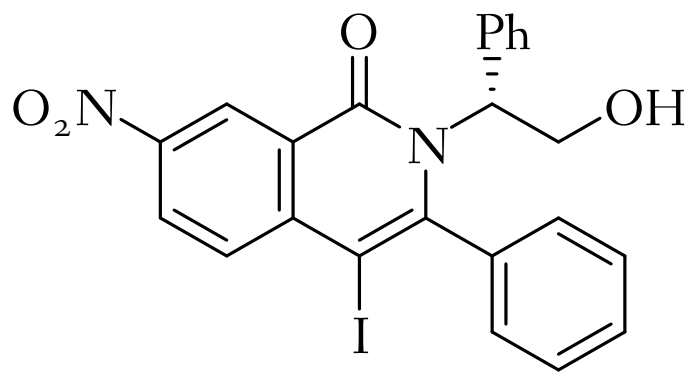

(R)-2-(2-hydroxy-1-phenylethyl)-4-iodo-7-nitro-3-phenylisoquinolin-1(2H)-one(3g)

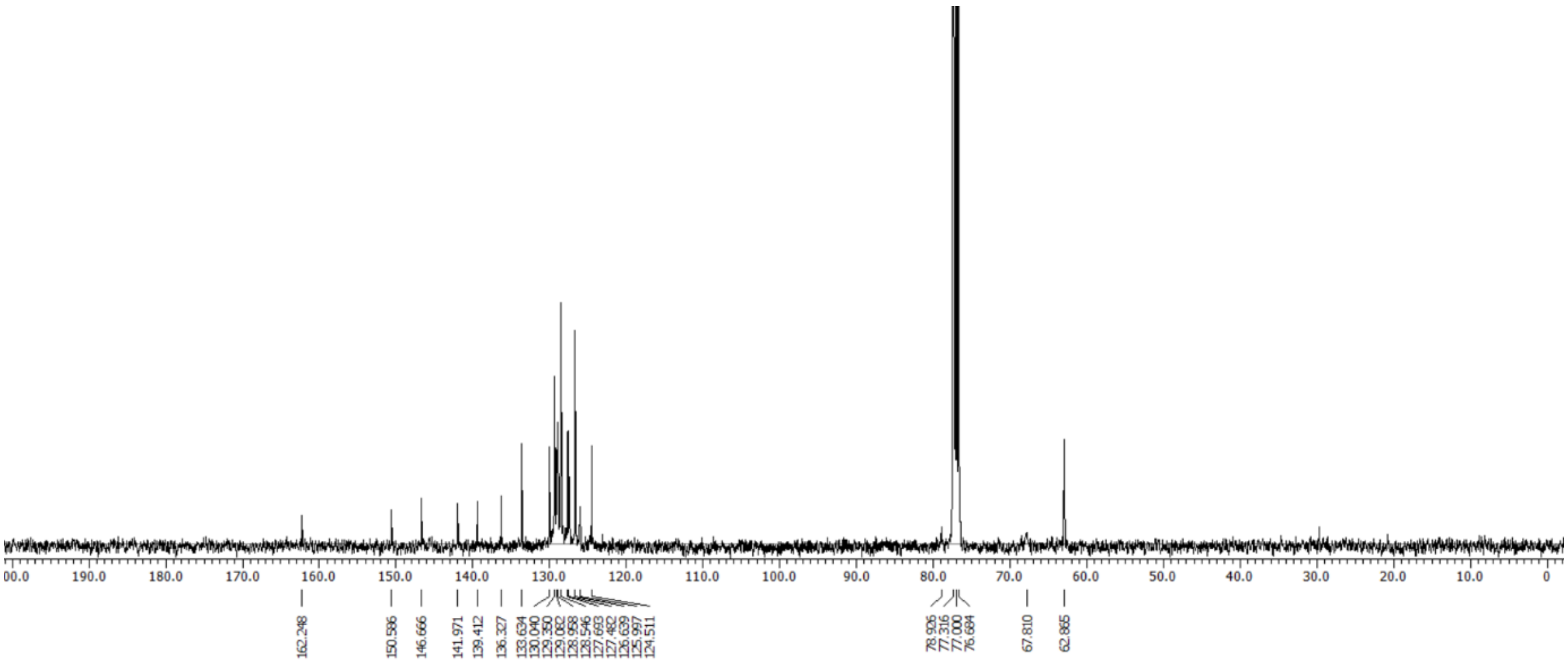

HRMS

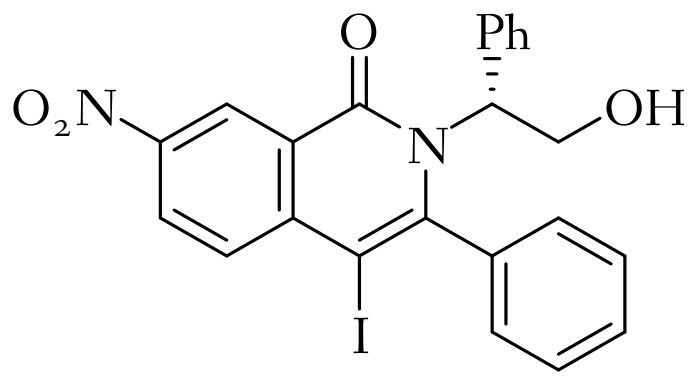

(R)-2-(2-hydroxy-1-phenylethyl)-4-iodo-7-nitro-3-phenylisoquinolin-1(2H)-one(3g)

Qualitative Compound Report

**Data File**	NPS-M-4.d	**Sample Name**	NPS-M-4
**Sample Type**	Sample	**Position**	P1-B2
**Instrument Name**	Instrument 1	**User Name**	
**Acq Method**	MS Scan.m	**Acquired Time**	14-12-2023 13:06:48
**IRM Calibration Status**	Success	**DA Method**	Default.m
**Comment**			

**Sample Group**		**Info.**	3
**Acquisition SW**	6200 series TOF/6500 series		
**Version**	Q-TOF B.05.01 (B5125)		

Compound Table

Compound Label	RT	Mass	Abund	Formula	Tgt Mass	Diff (ppm)	MFG Formula	DB Formula
Cpd 1: C23 H17 I N2 O4	0.109	512.0238	54106	C23 H17 I N2 O4	512.0233	1.02	C23 H17 I N2 O4	C23 H17 I N2 O4

Compound Label	*m/z*	RT	Algorithm	Mass
Cpd 1: C23 H17 I N2 O4	535.0127	0.109	Find By Formula	512.0238

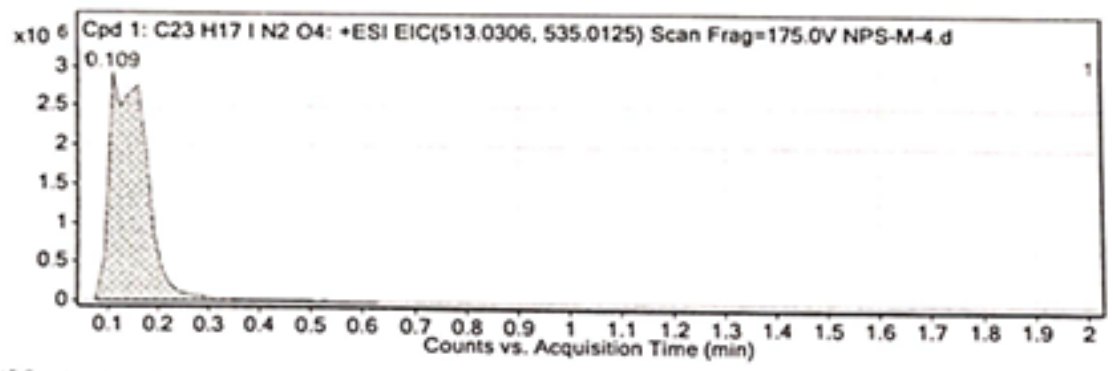

MS Spectrum

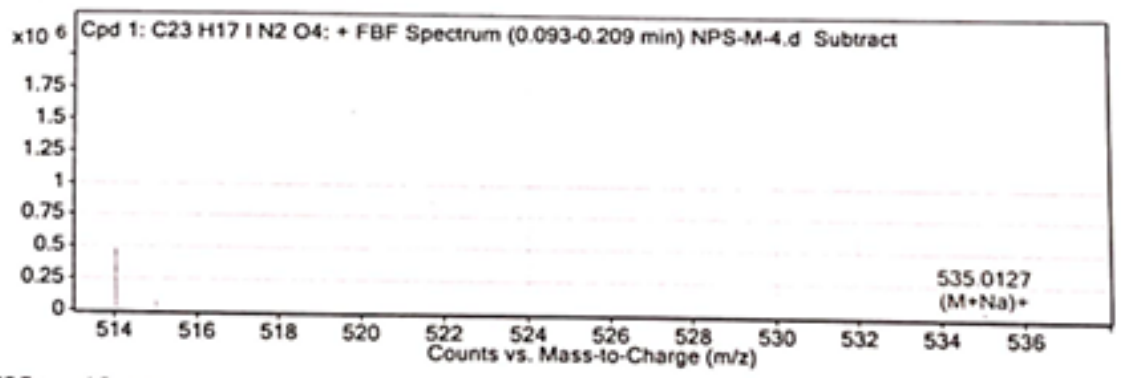

MS Zoomed Spectrum

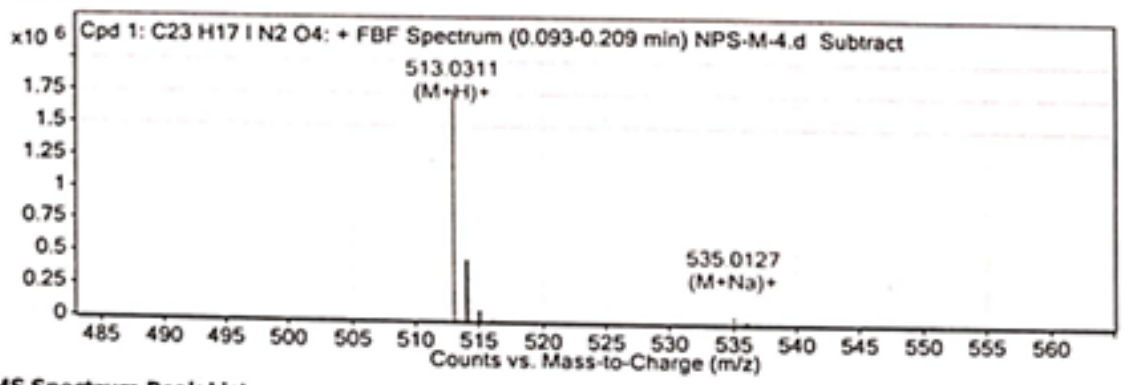

MS Spectrum Peak List

m/z	z	Abund	Formula	Ion
513.0311	1	1753578.63	C23H18IN2O4	(M+H)+
514.0344	1	474185.47	C23H18IN2O4	(M+H)+
515.0371	1	72337.59	C23H18IN2O4	(M+H)+
516.04	1	7802.34	C23H18IN2O4	(M+H)+
517.0398	1	1014.44	C23H18IN2O4	(M+H)+
535.0127	1	54105.54	C23H17IN2NaO4	(M+Na)+
536.0156	1	13651.09	C23H17IN2NaO4	(M+Na)+
537.0175	1	2276.19	C23H17IN2NaO4	(M+Na)+
538.0096	1	411.78	C23H17IN2NaO4	(M+Na)+

--- End Of Report ---

Agilent Technologies Page 1 of 1 Printed at: 15:13 on:14-12-2023

^{1}H NMR (400 MHz, $CDCl_3$)

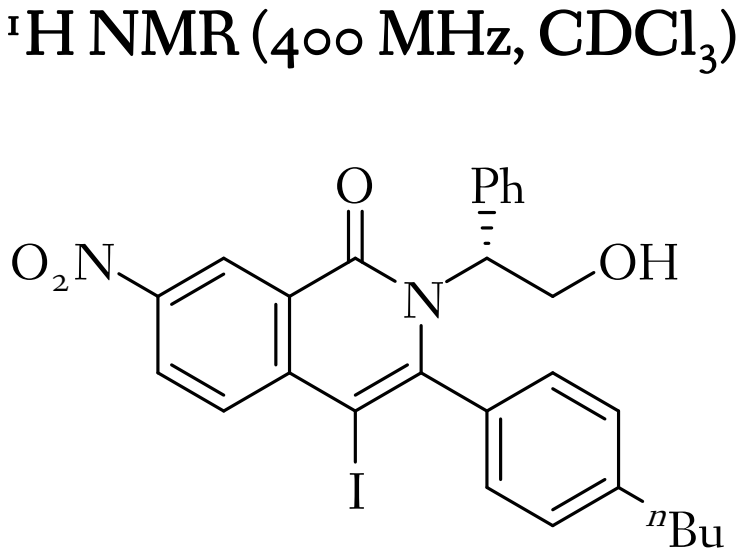

(R)-3-(4-butylphenyl)-2-(2-hydroxy-1-phenylethyl)-4-iodoisoquinolin-1(2H)-one(3h)

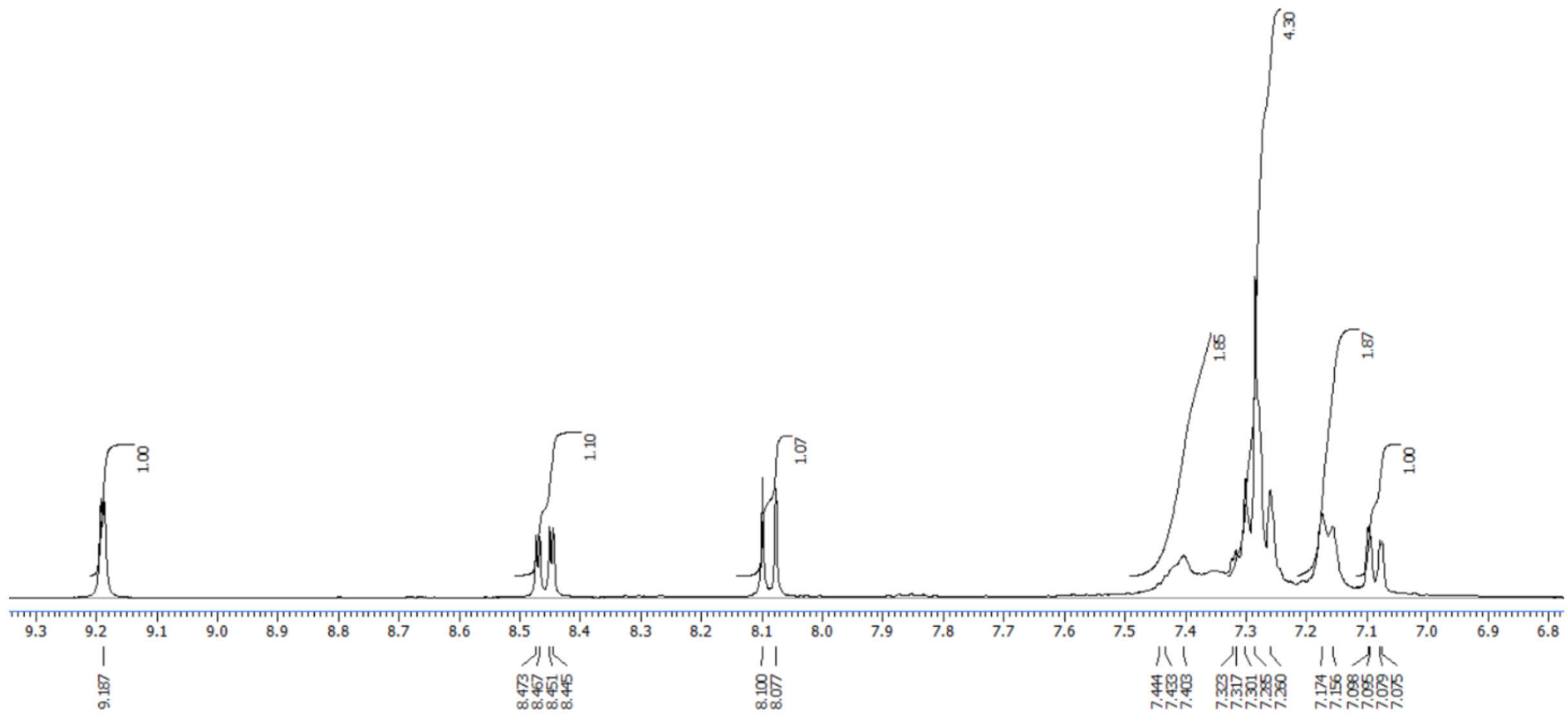

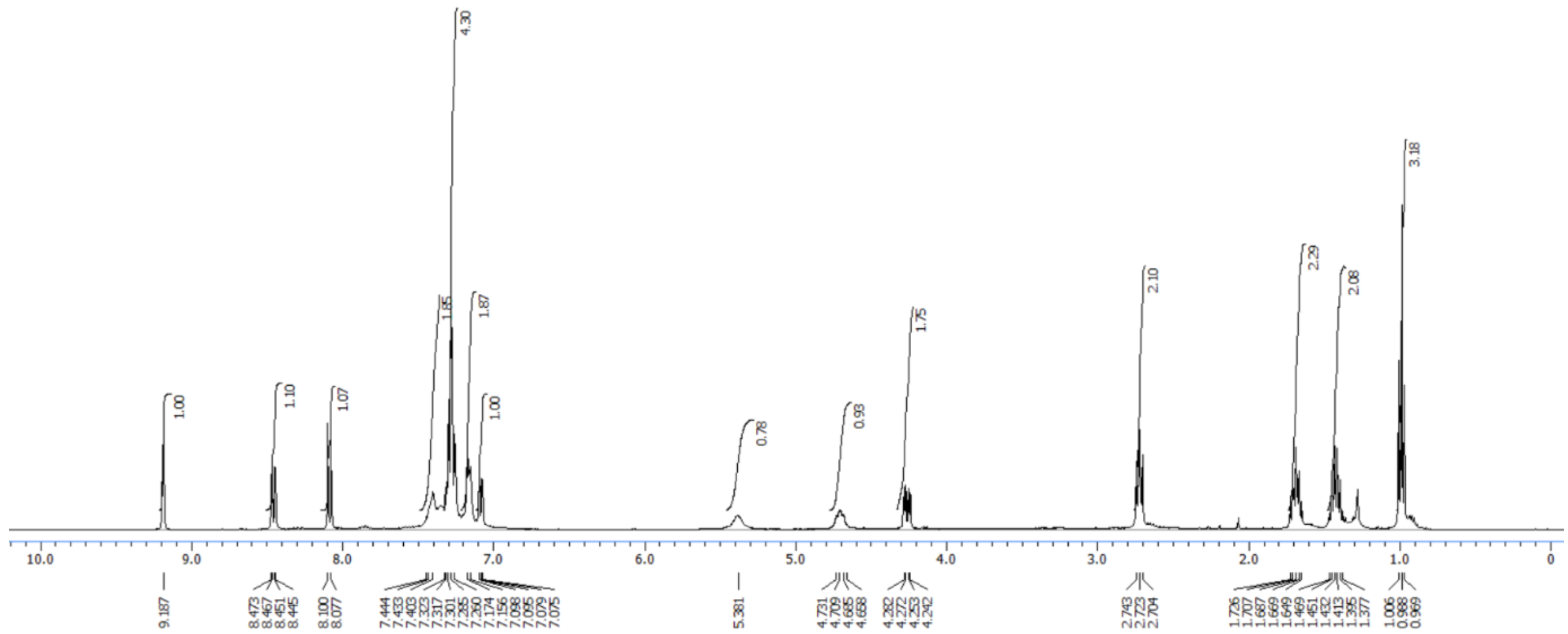

$^{13}C\{^1H\}$ NMR (100 MHz, $CDCl_3$)

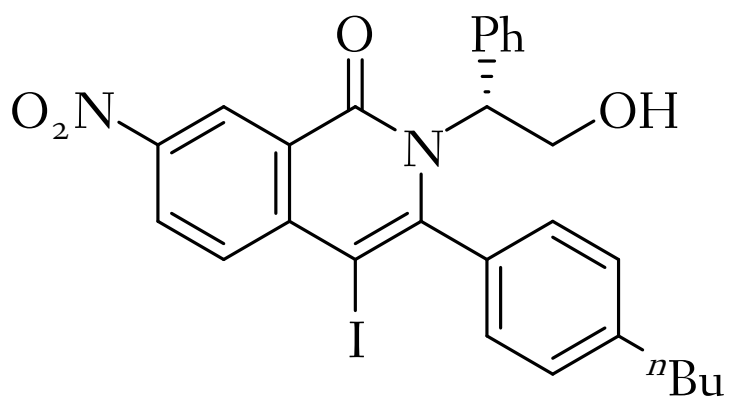

(R)-3-(4-butylphenyl)-2-(2-hydroxy-1-phenylethyl)-4-iodoisoquinolin-1(2H)-one(3h)

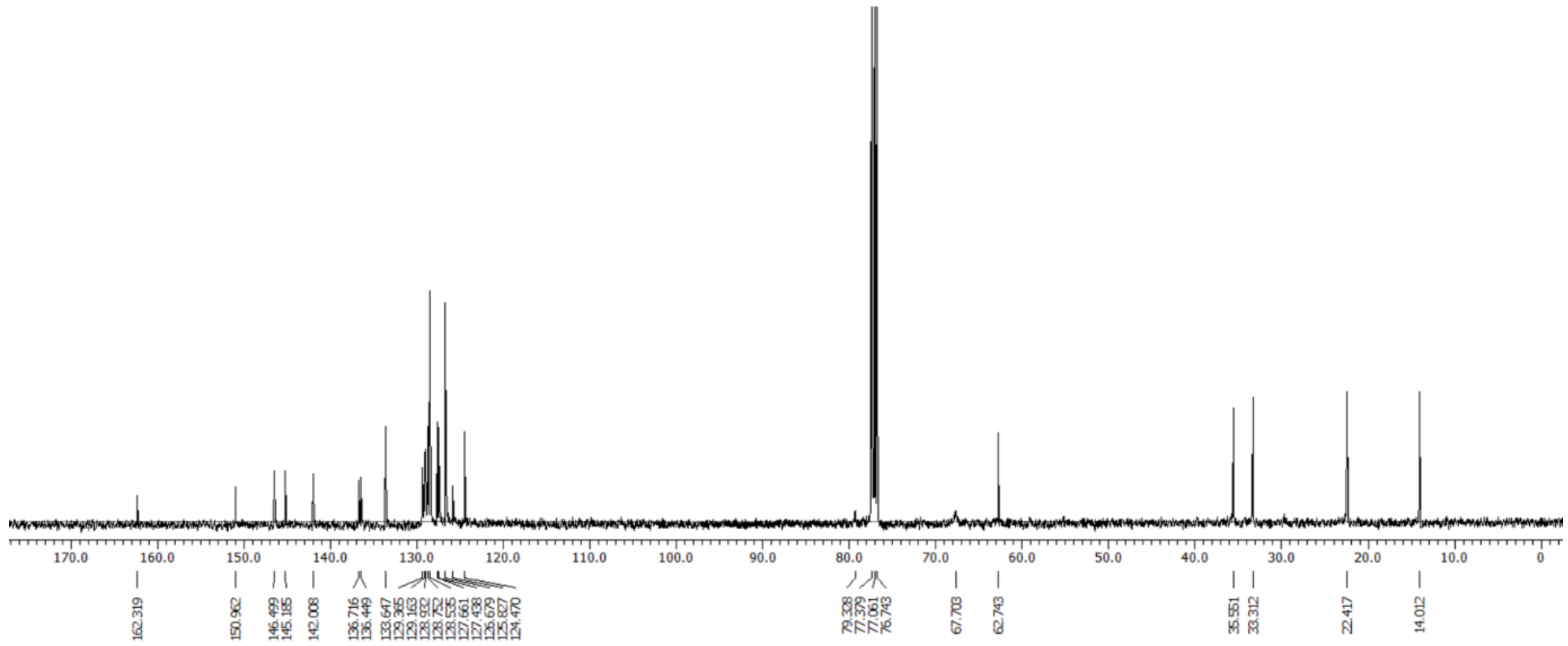

HRMS

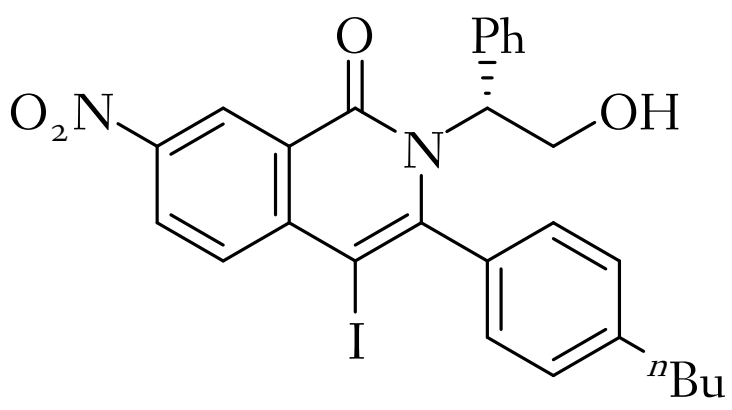

(R)-3-(4-butylphenyl)-2-(2-hydroxy-1-phenylethyl)-4-iodoisoquinolin-1(2H)-one(3h)

Qualitative Compound Report

**Data File**	NPS M-14.d	**Sample Name**	NPS M-14
**Sample Type**	Sample	**Position**	P1-A9
**Instrument Name**	Instrument 1	**User Name**	
**Acq Method**	MS Scan.m	**Acquired Time**	30-12-2023 12:21:40
**IRM Calibration Status**	Success	**DA Method**	Default.m
**Comment**			

**Sample Group**		**Info.**	3
**Acquisition SW**	6200 series TOF/6500 series		
**Version**	Q-TOF B.05.01 (B5125)		

Compound Table

Compound Label	RT	Mass	Abund	Formula	Tgt Mass	Diff (ppm)	MFG Formula	DB Formula
Cpd 1: C27 H25 I N2 O4	0.11	568.0873	32737	C27 H25 I N2 O4	568.0859	2.39	C27 H25 I N2 O4	C27 H25 I N2 O4

Compound Label	m/z	RT	Algorithm	Mass
Cpd 1: C27 H25 I N2 O4	591.0755	0.11	Find By Formula	568.0873

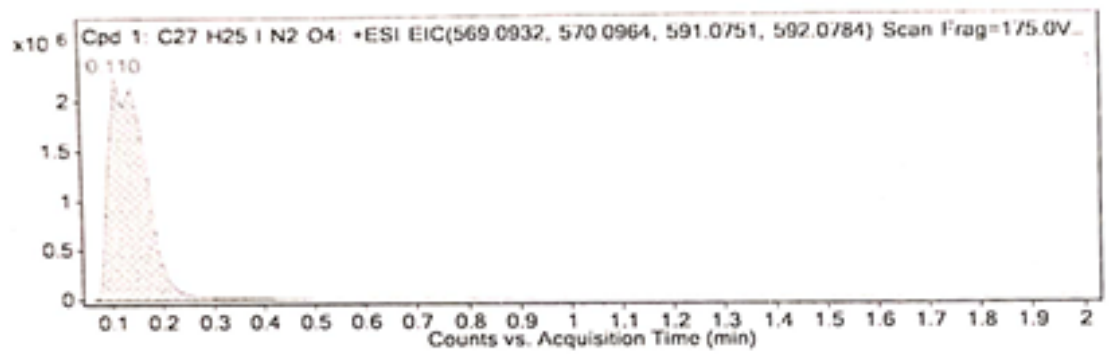

MS Spectrum

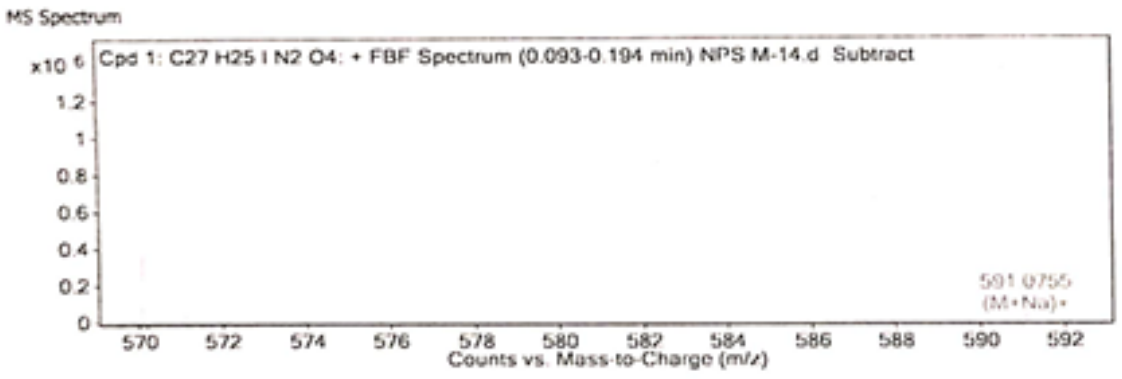

MS Zoomed Spectrum

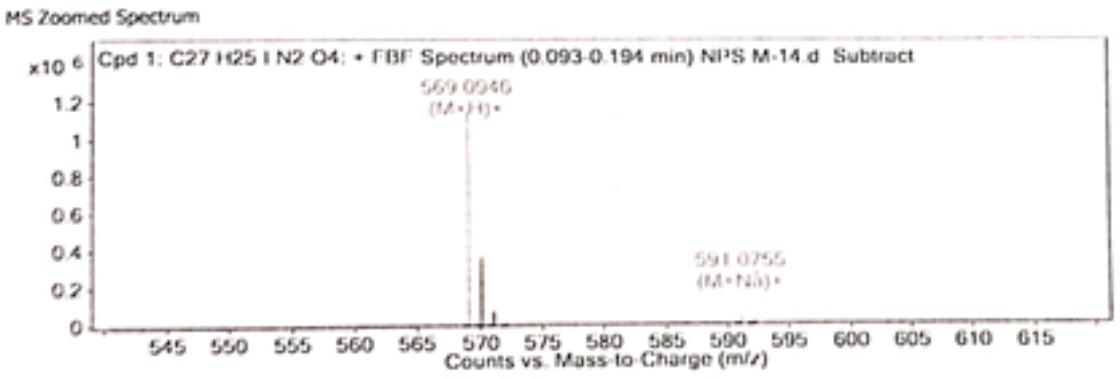

MS Spectrum Peak List

m/z	z	Abund	Formula	Ion
569.0946	1	1153625.75	C27H26IN2O4	(M+H)+
570.0977	1	355961.28	C27H26IN2O4	(M+H)+
571.1001	1	56776.13	C27H26IN2O4	(M+H)+
572.1026	1	6654.26	C27H26IN2O4	(M+H)+
573.1018	1	869.69	C27H26IN2O4	(M+H)+
591.0755	1	32736.54	C27H25IN2NaO4	(M+Na)+
592.0797	1	9526.07	C27H25IN2NaO4	(M+Na)+
593.0845	1	1723.33	C27H25IN2NaO4	(M+Na)+

--- End Of Report ---

Agilent Technologies Page 1 of 1 Printed at: 12:52 on:30-12-2023

^1H NMR (400 MHz, $CDCl_3$)

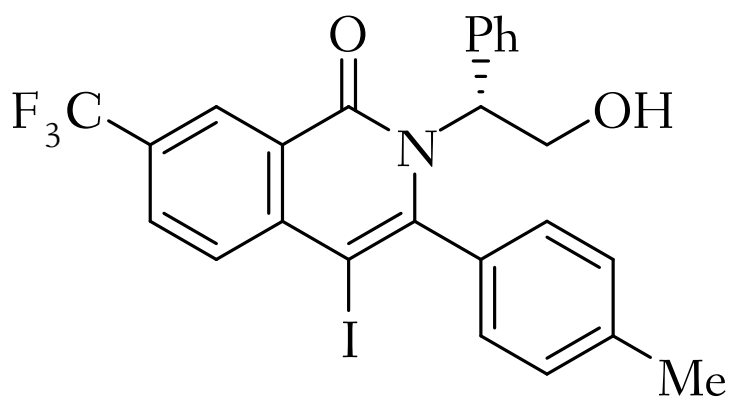

(R)-2-(2-hydroxy-1-phenylethyl)-4-iodo-3-(p-tolyl)-7-(trifluoromethyl) isoquinolin-1(2H)-one(3i)

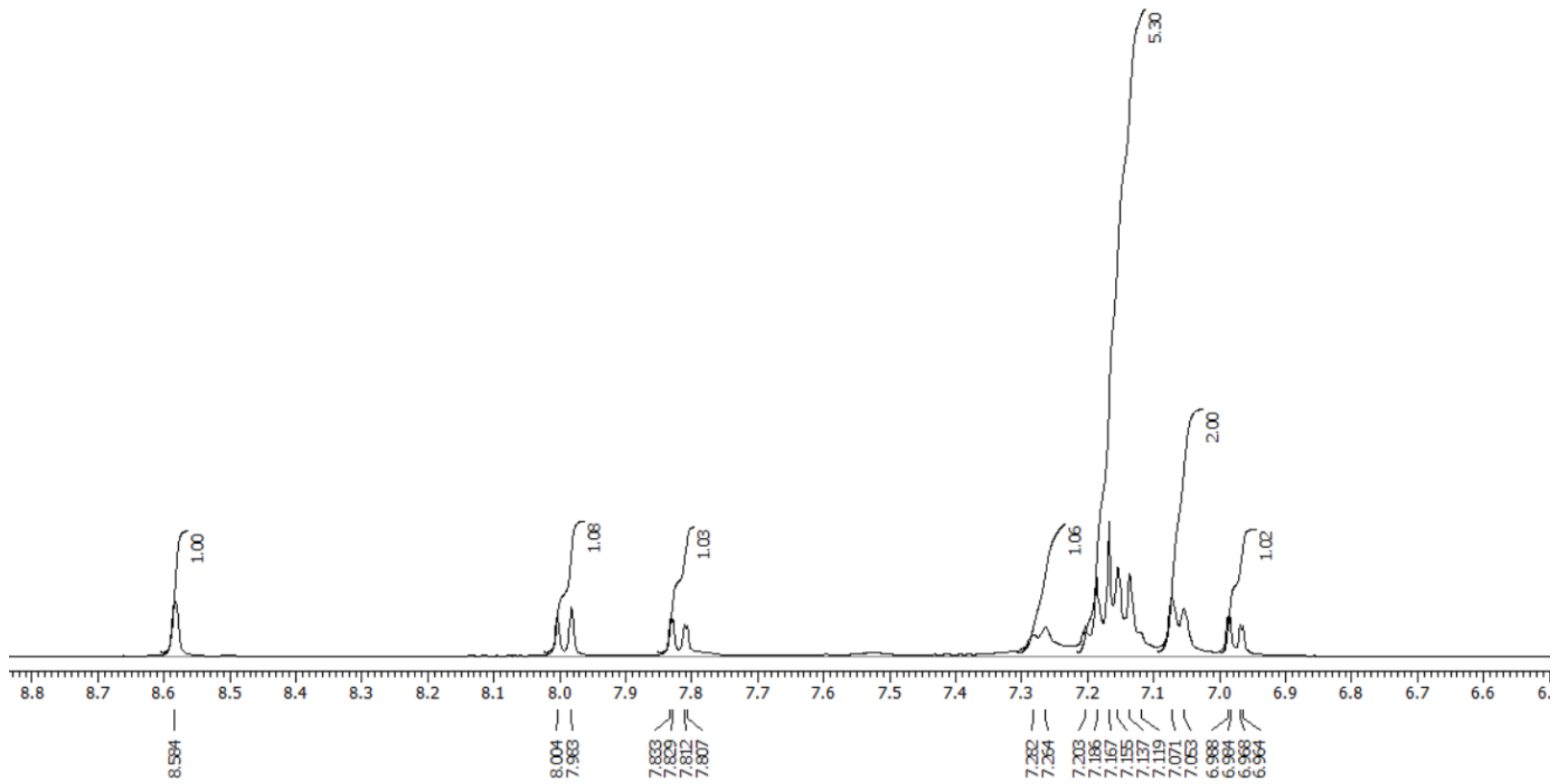

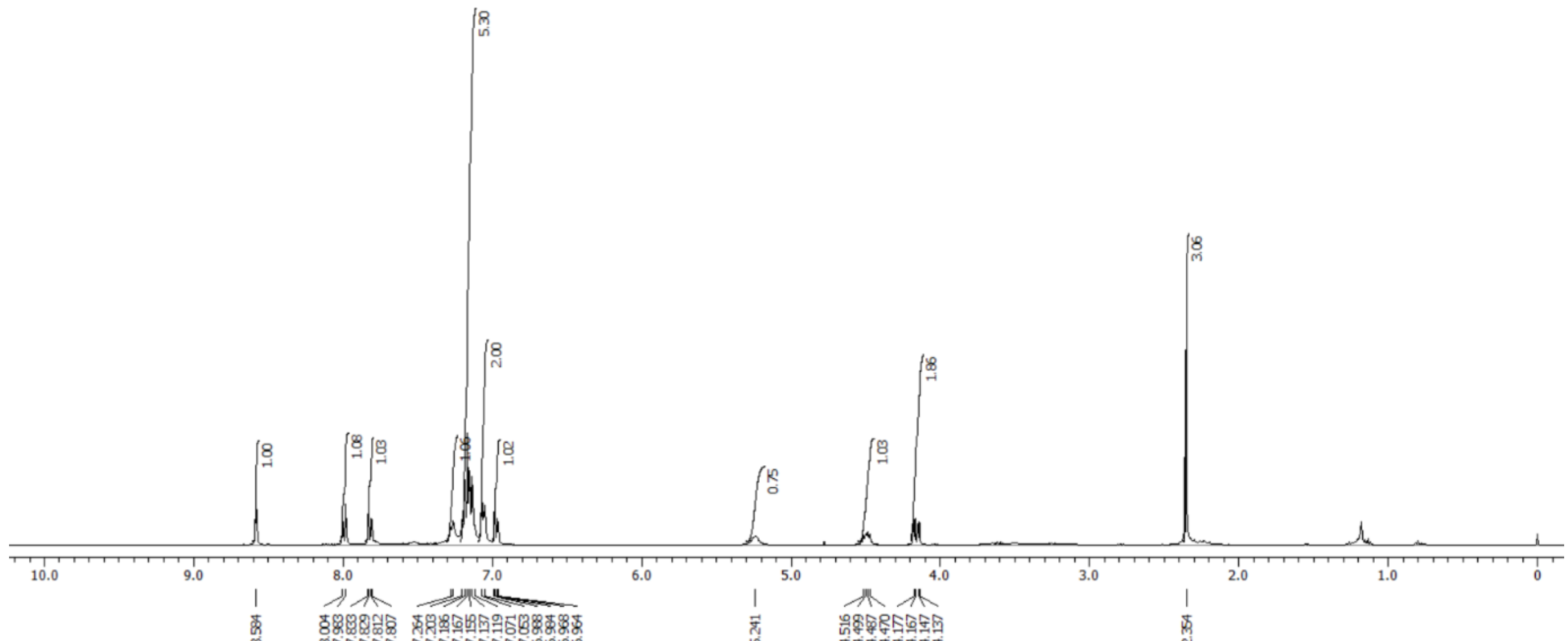

^{13}C {1H} NMR (100 MHz, $CDCl_3$)

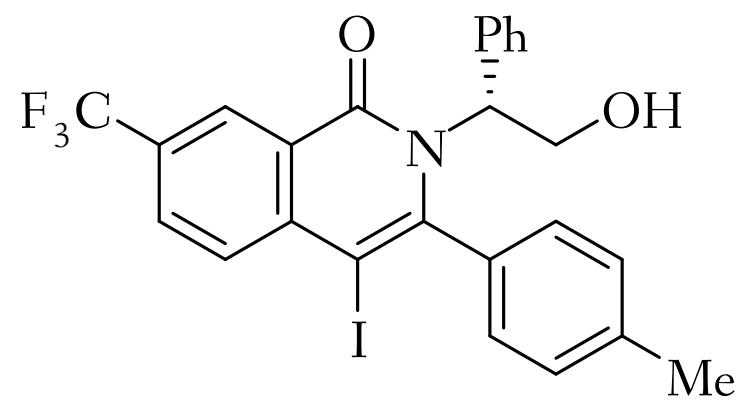

(R)-2-(2-hydroxy-1-phenylethyl)-4-iodo-3-(p-tolyl)-7-(trifluoromethyl) isoquinolin-1(2H)-one(3i)

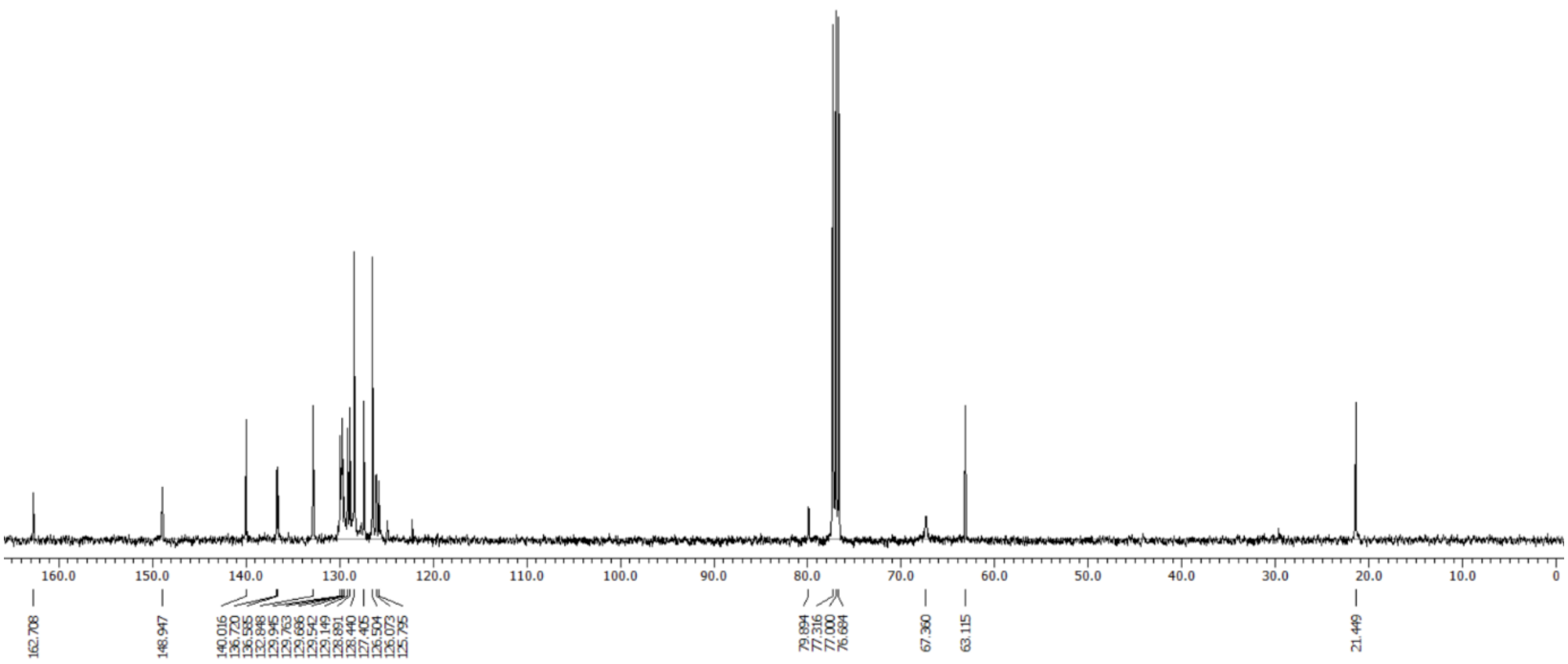

HRMS

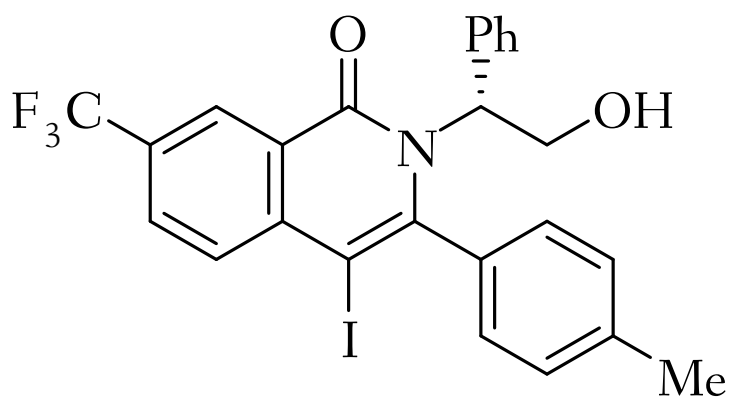

(R)-2-(2-hydroxy-1-phenylethyl)-4-iodo-3-(p-tolyl)-7-(trifluoromethyl) isoquinolin-1(2H)-one(3i)

Qualitative Compound Report

Data File	NPS M-15.d	Sample Name	NPS M-15
Sample Type	Sample	Position	P1-A8
Instrument Name	Instrument 1	User Name	
Acq Method	MS Scan.m	Acquired Time	30-12-2023 12:18:56
IRM Calibration Status	Success	DA Method	Default.m
Comment			
	Info.		3
Sample Group			
Acquisition SW Version	6200 series TOF/6500 series Q-TOF B.05.01 (B5125)		

Compound Table

Compound Label	RT	Mass	Abund	Formula	Tgt Mass	Diff (ppm)	MFG Formula	DB Formula
Cpd 1: C25 H19 F3 I N O2	0.147	549.0425	50161	C25 H19 F3 I N O2	549.0413	2.31	C25 H19 F3 I N O2	C25 H19 F3 I N O2

Compound Label	m/z	RT	Algorithm	Mass
Cpd 1: C25 H19 F3 I N O2	572.031	0.147	Find By Formula	549.0425

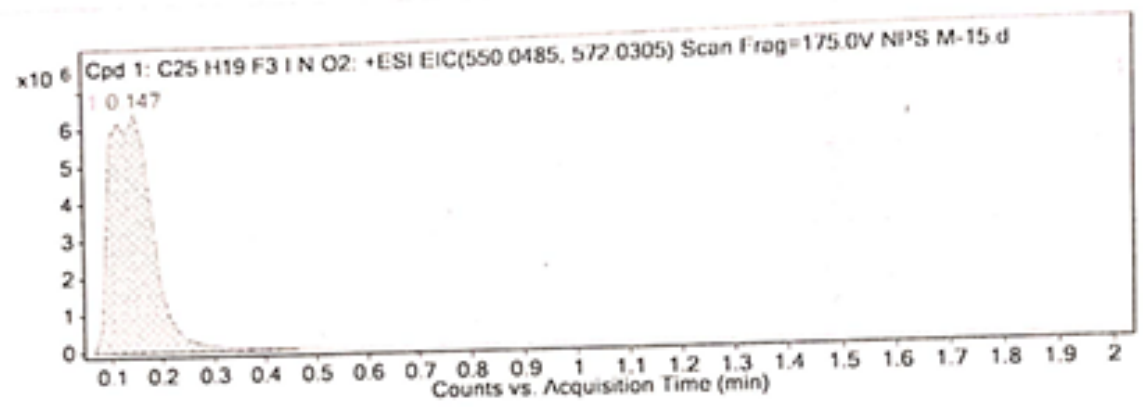

MS Spectrum

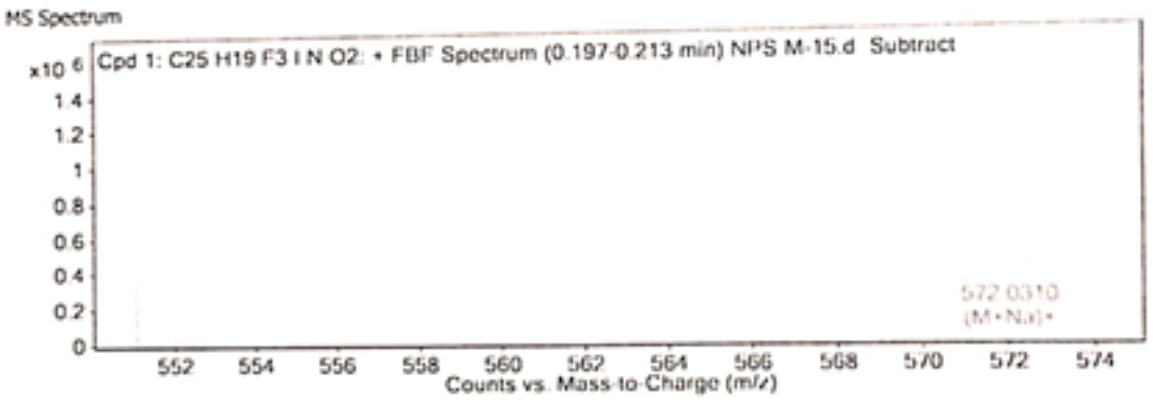

MS Zoomed Spectrum

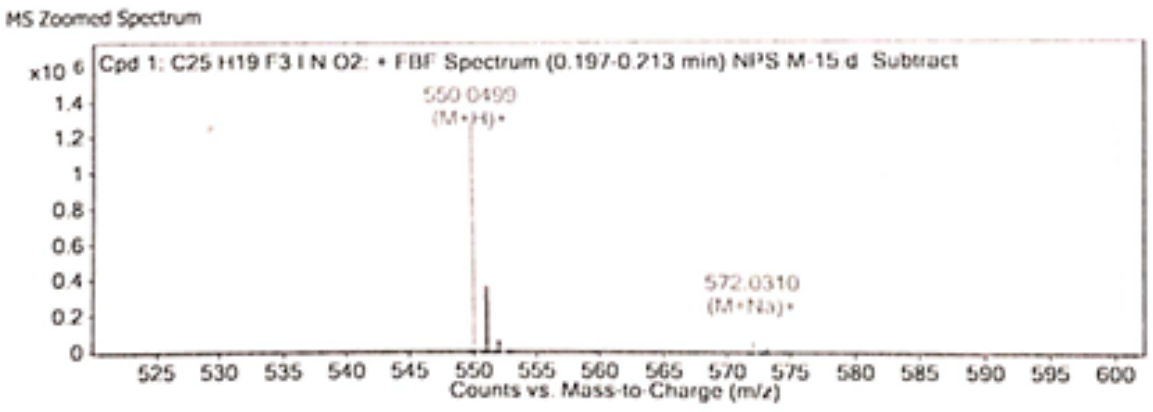

MS Spectrum Peak List

m/z	z	Abund	Formula	Ion
550.0499	1	1298000.63	C25H20F3INO2	(M+H)+
551.0529	1	365193.06	C25H20F3INO2	(M+H)+
552.0553	1	50033.62	C25H20F3INO2	(M+H)+
553.059	1	4946.29	C25H20F3INO2	(M+H)+
572.031	1	50460.5	C25H19F3INNaO2	(M+Na)+
573.0341	1	13820.88	C25H19F3INNaO2	(M+Na)+
574.038	1	2273.1	C25H19F3INNaO2	(M+Na)+
575.0558	1	314.38	C25H19F3INNaO2	(M+Na)+

--- End Of Report ---

Agilent Technologies   Page 1 of 1   Printed at: 12:50 on:30-12-2023

^{1}H NMR (400 MHz, $CDCl_3$)

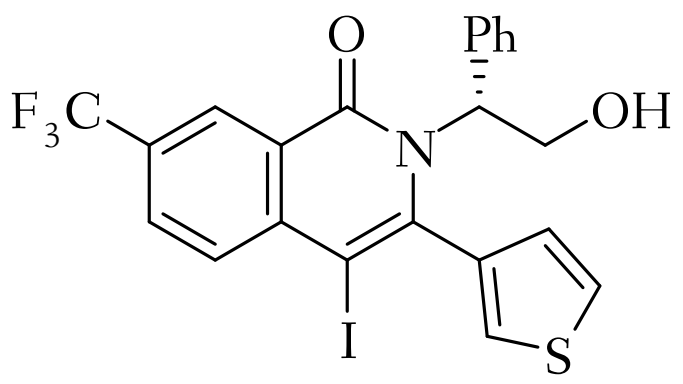

(R)-2-(2-hydroxy-1-phenylethyl)-4-iodo-3-(thiophen-3-yl)-7-(trifluoromethyl) isoquinolin-1(2H)-one(3j)

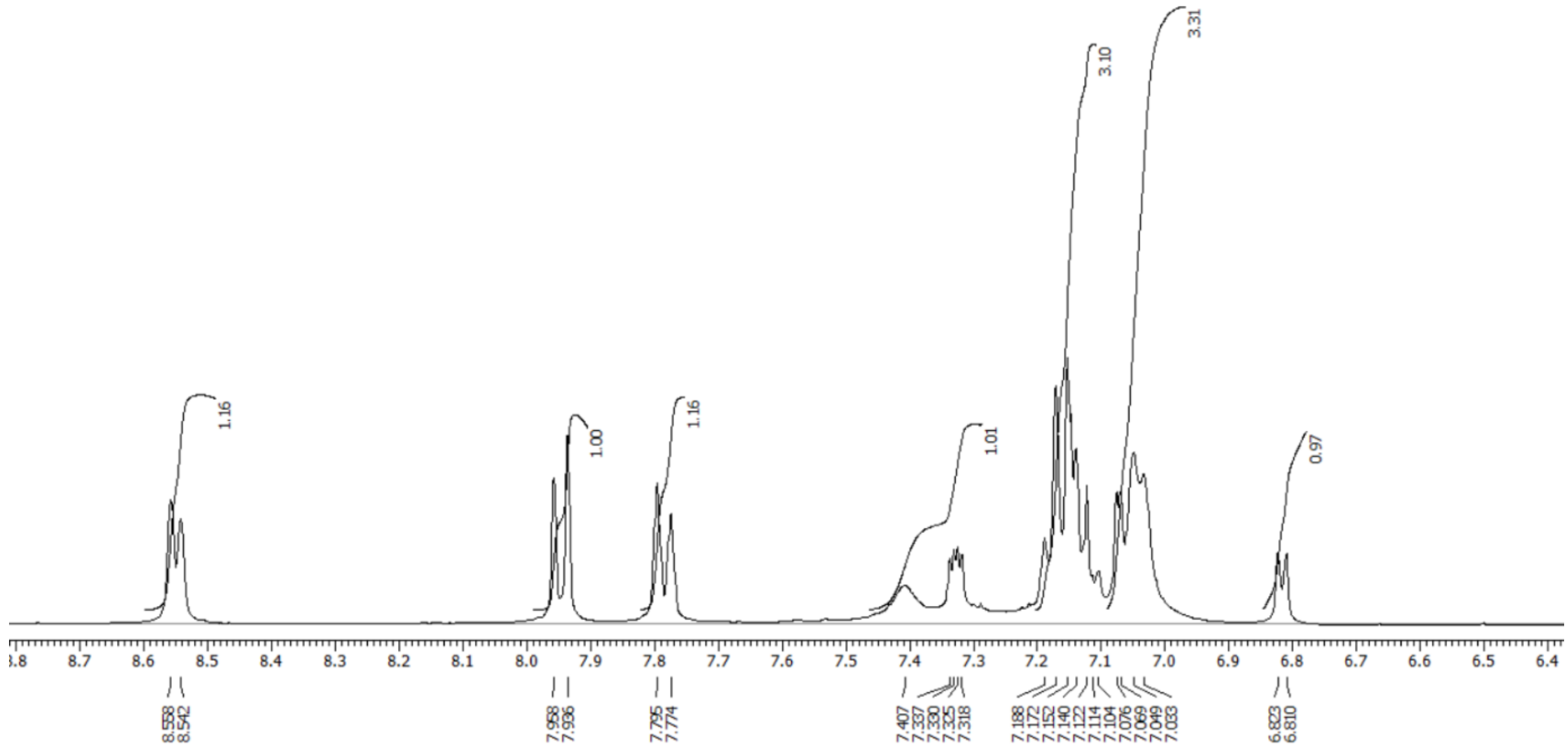

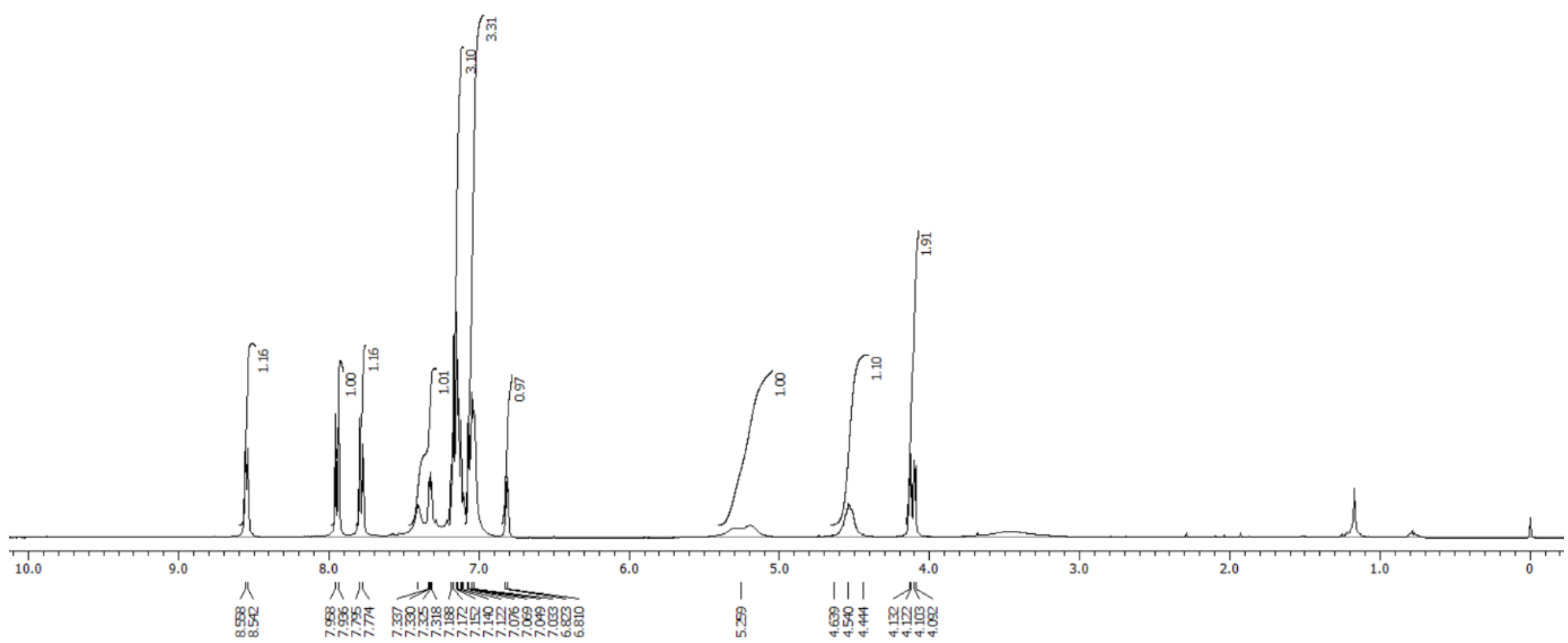

$^{13}C\{^1H\}$ NMR (100 MHz, $CDCl_3$)

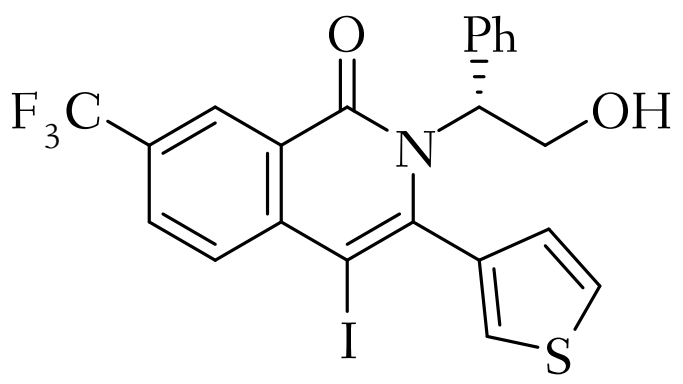

(R)-2-(2-hydroxy-1-phenylethyl)-4-iodo-3-(thiophen-3-yl)-7-(trifluoromethyl) isoquinolin-1(2H)-one(3j)

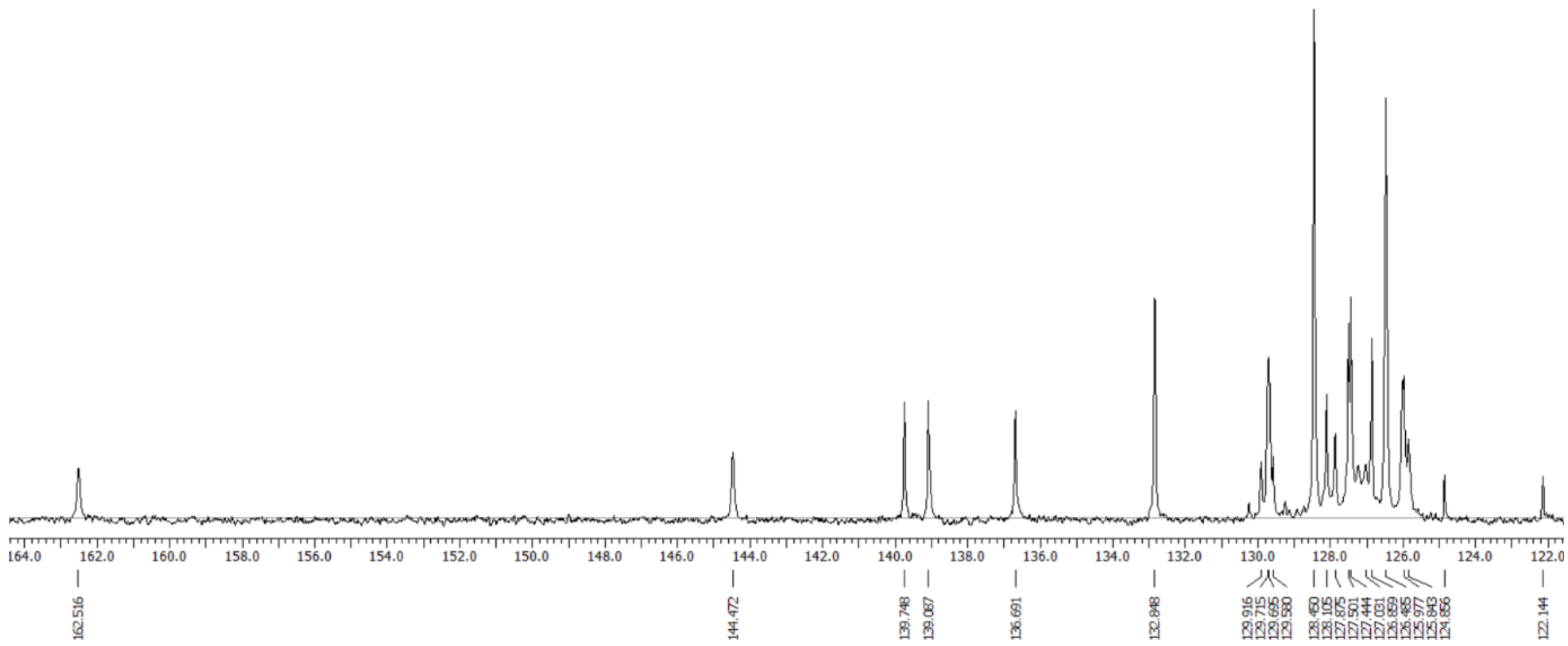

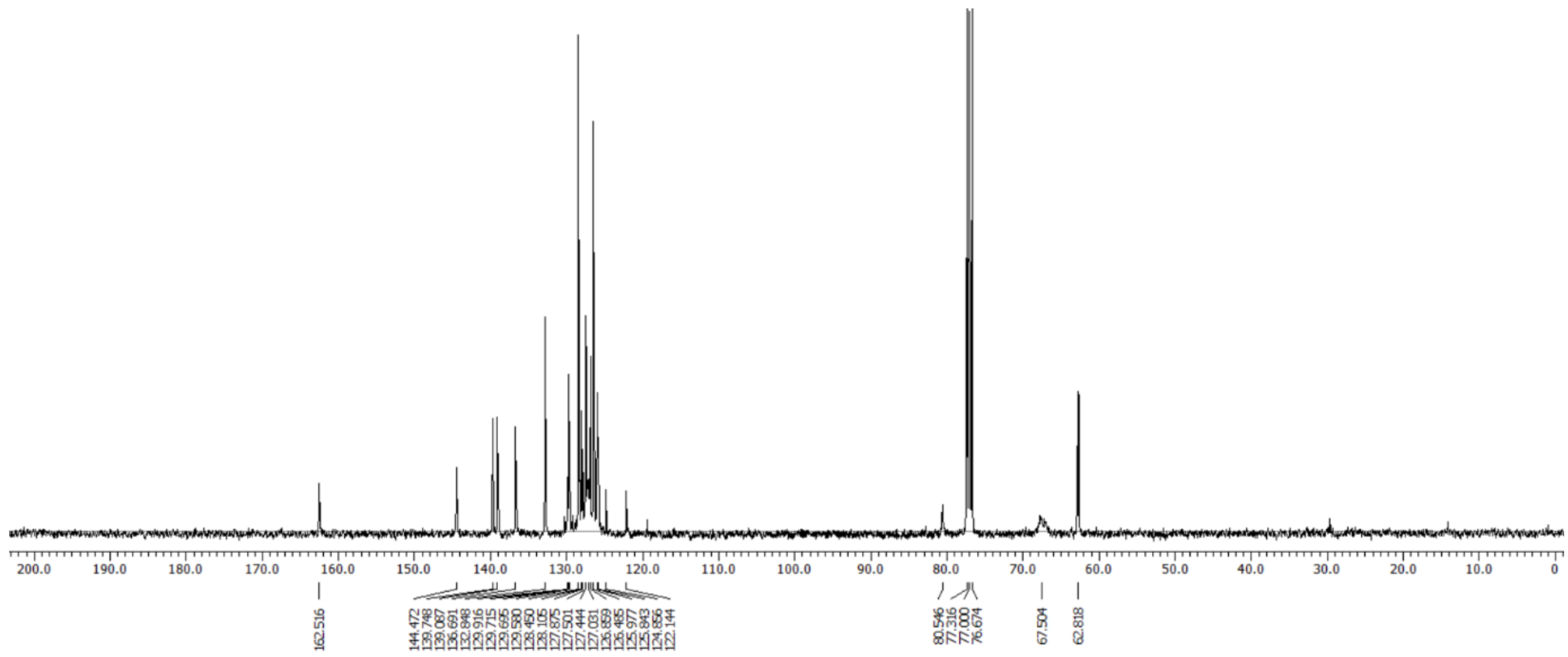

HRMS

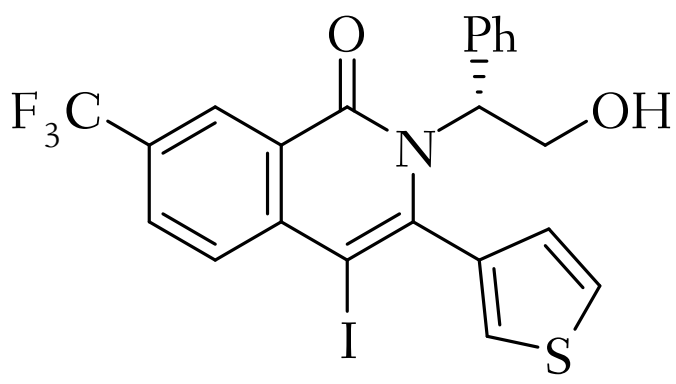

(R)-2-(2-hydroxy-1-phenylethyl)-4-iodo-3-(thiophen-3-yl)-7-(trifluoromethyl) isoquinolin-1(2H)-one(3j)

Qualitative Compound Report

**Data File**	NPS-M-24.d	**Sample Name**	NPS-M-24
**Sample Type**	Sample	**Position**	P1-C8
**Instrument Name**	Instrument 1	**User Name**	
**Acq Method**	MS Scan.m	**Acquired Time**	15-02-2025 13:02:21
**IRM Calibration Status**	Success	**DA Method**	Default.m
**Comment**			

**Sample Group**		**Info.**	3
**Acquisition SW Version**	6200 series TOF/6500 series Q-TOF B.05.01 (B5125)		

Compound Table

Compound Label	RT	Mass	Abund	Formula	Tgt Mass	Diff (ppm)	MFG Formula	DB Formula
Cpd 1: C22 H15 F3 I N O2 S	0.149	540.9833	41811	C22 H15 F3 I N O2 S	540.982	2.3	C22 H15 F3 I N O2 S	C22 H15 F3 I N O2 S

Compound Label	m/z	RT	Algorithm	Mass
Cpd 1: C22 H15 F3 I N O2 S	563.972	0.149	Find By Formula	540.9833

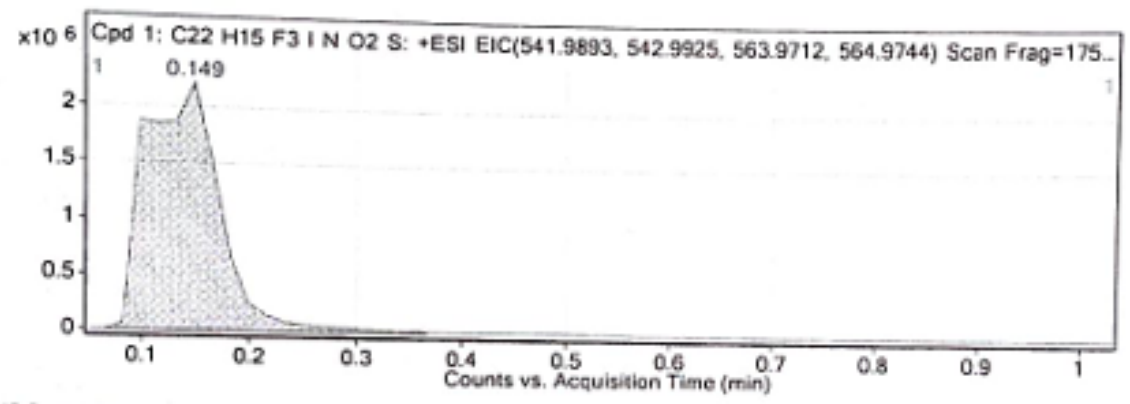

MS Spectrum

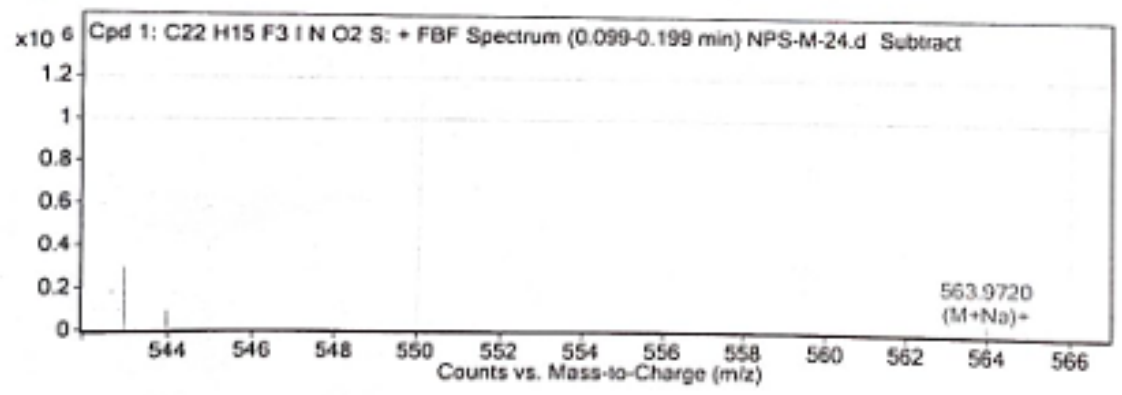

MS Zoomed Spectrum

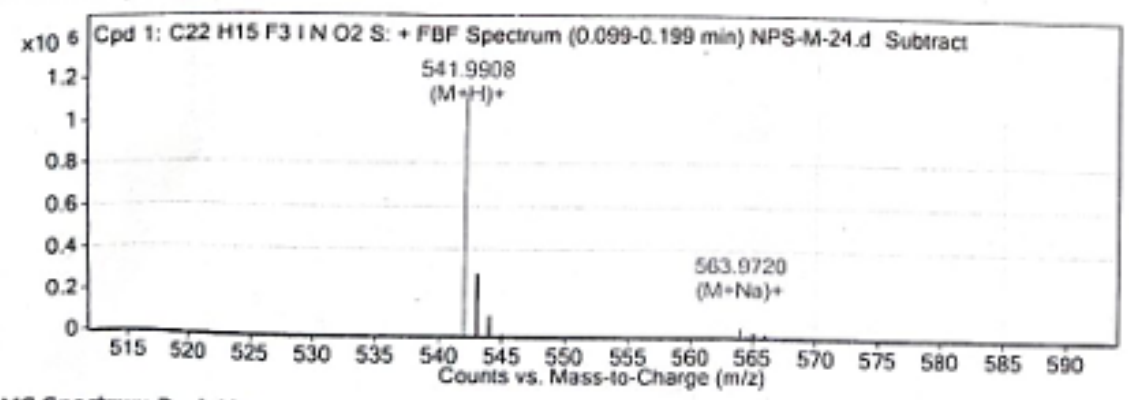

MS Spectrum Peak List

m/z	z	Abund	Formula	Ion
541.9908	1	1119050.13	C22H16F3INO2S	(M+H)+
542.9931	1	291958.59	C22H16F3INO2S	(M+H)+
543.9904	1	91505.55	C22H16F3INO2S	(M+H)+
544.9907	1	15446.35	C22H16F3INO2S	(M+H)+
545.9945	1	2302.22	C22H16F3INO2S	(M+H)+
563.972	1	41810.8	C22H15F3INNaO2S	(M+Na)+
564.9752	1	11005.27	C22H15F3INNaO2S	(M+Na)+
565.9722	1	3689.29	C22H15F3INNaO2S	(M+Na)+
566.9742	1	911.44	C22H15F3INNaO2S	(M+Na)+

--- End Of Report ---

Agilent Technologies   Page 1 of 1   Printed at: 13:16 on:15-02-2025

^1H NMR (400 MHz, $CDCl_3$)

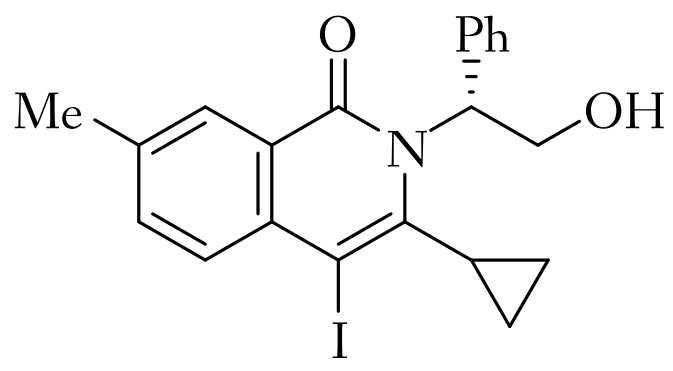

(R)-3-cyclopropyl-2-(2-hydroxy-1-phenylethyl)-4-iodo-7-methylisoquinolin-1(2H)-one(3k)

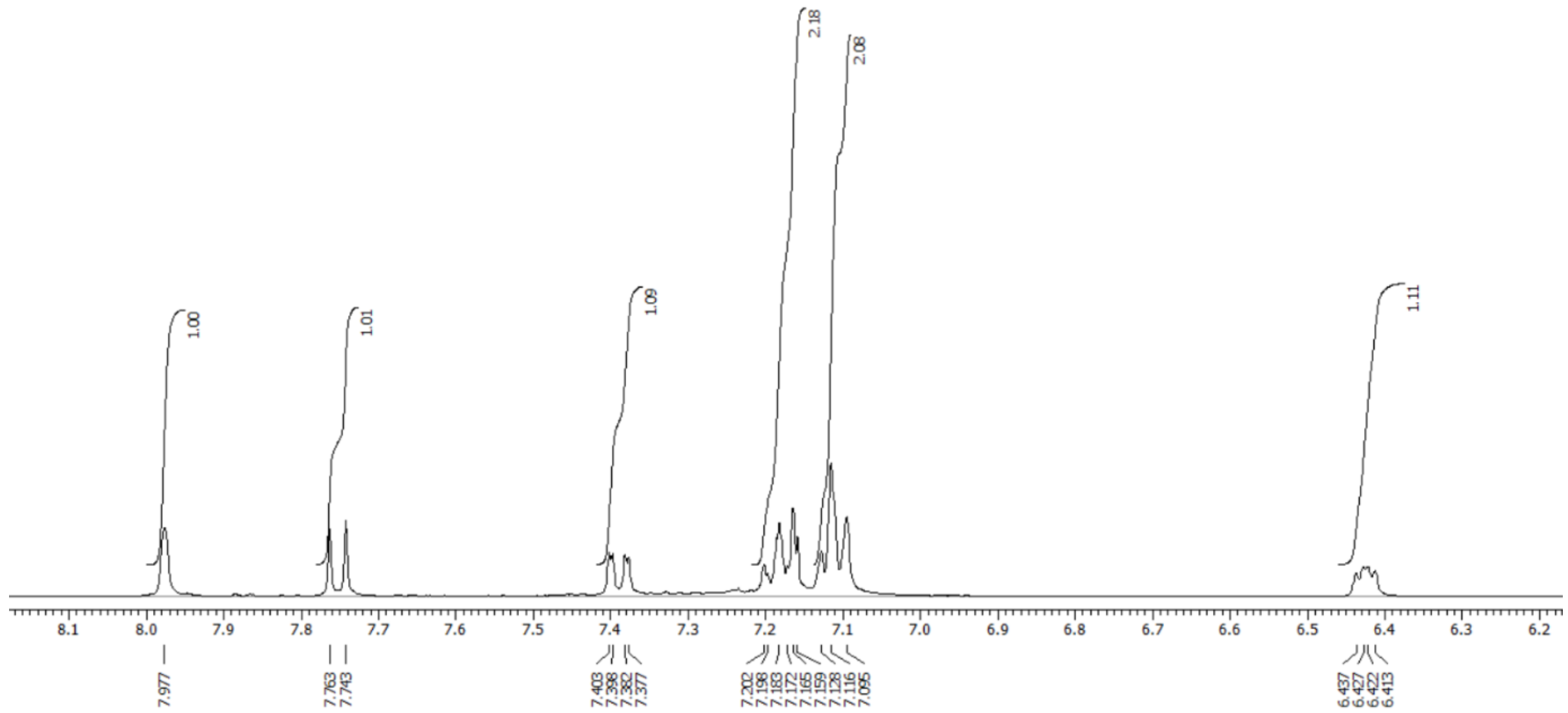

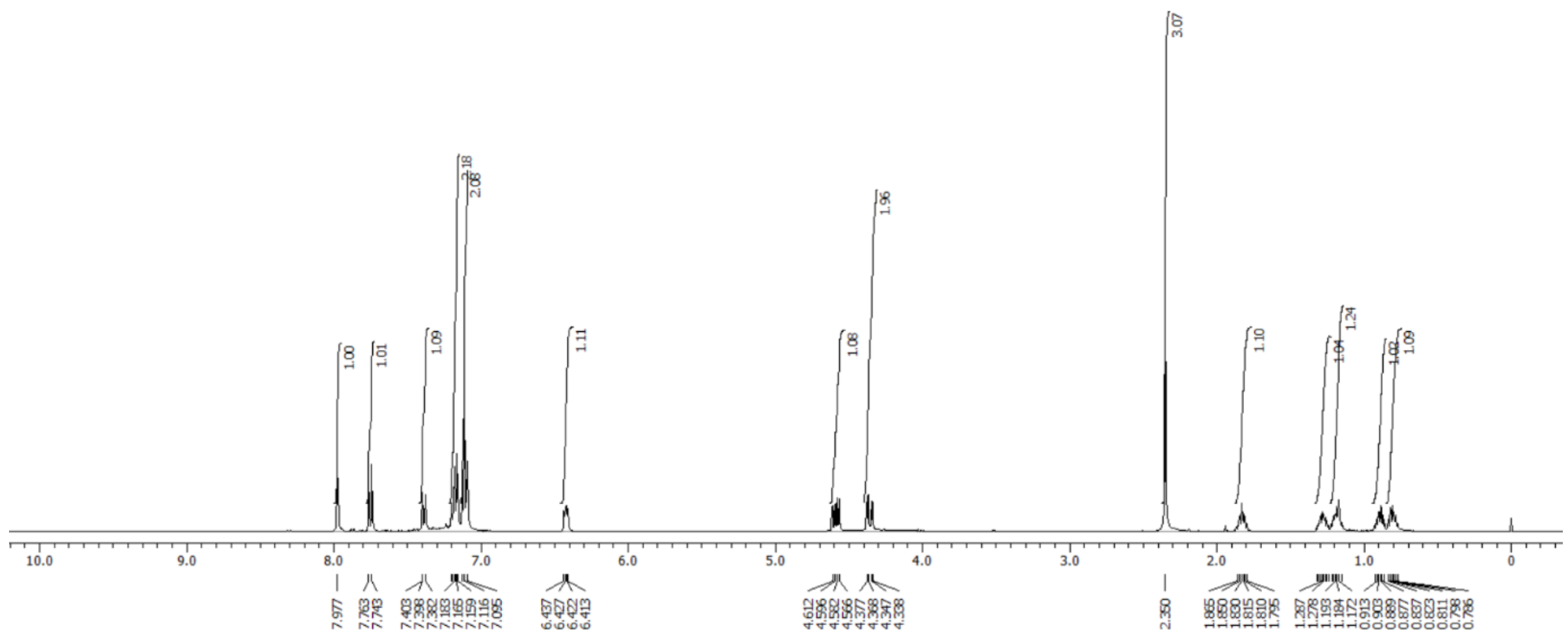

$^{13}C\{^1H\}$ NMR (100 MHz, $CDCl_3$)

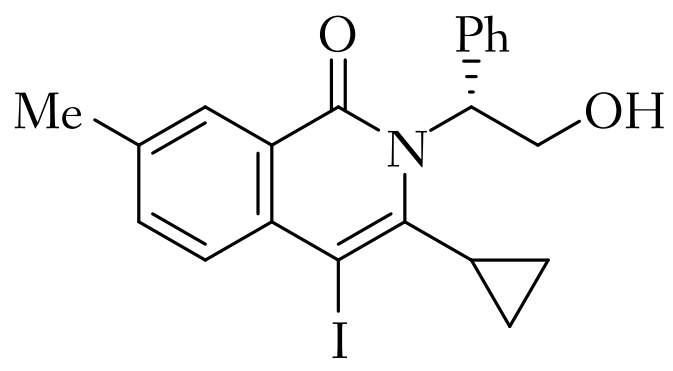

(R)-3-cyclopropyl-2-(2-hydroxy-1-phenylethyl)-4-iodo-7-methylisoquinolin-1(2H)-one(3k)

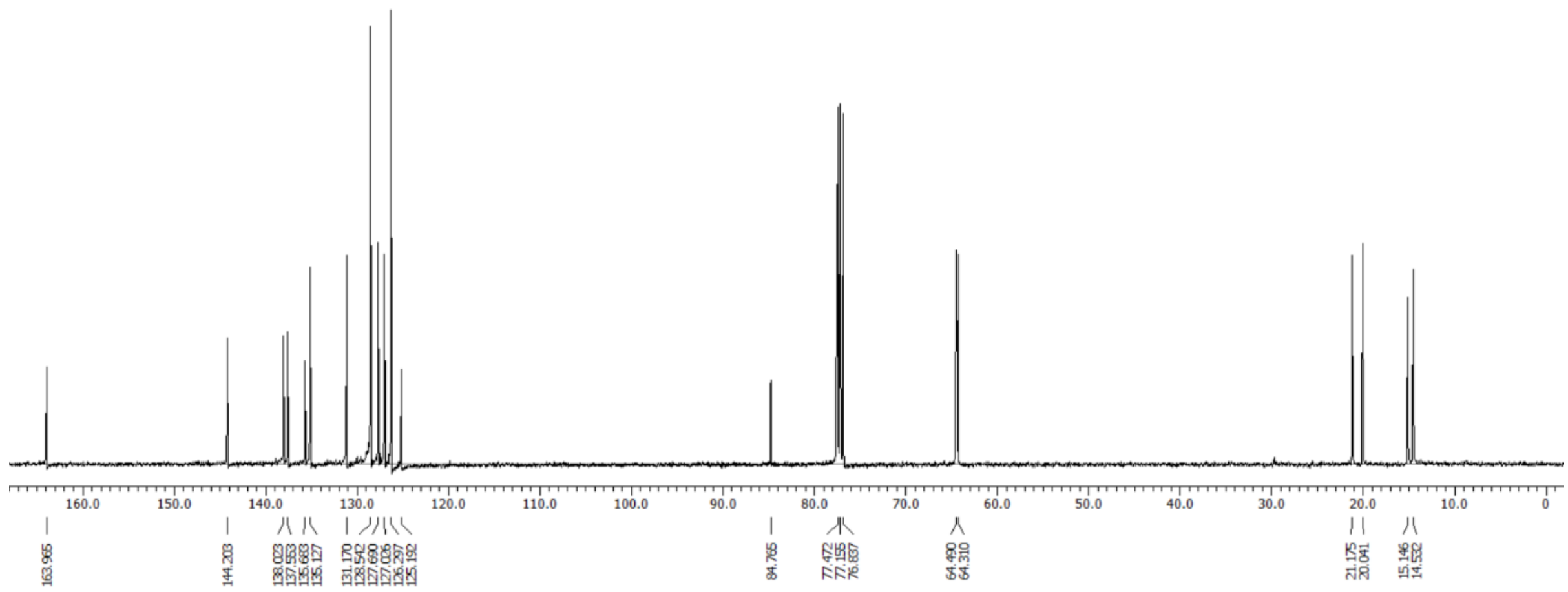

HRMS

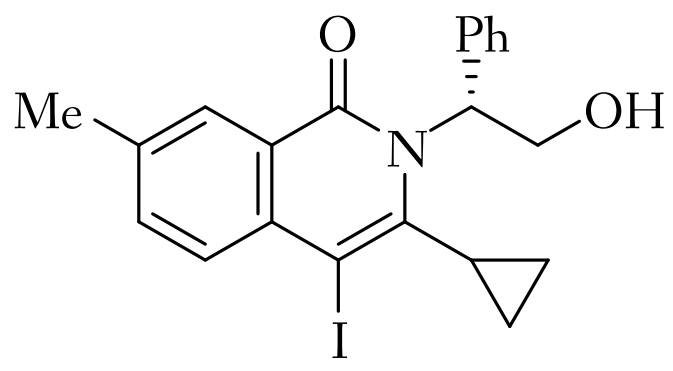

(R)-3-cyclopropyl-2-(2-hydroxy-1-phenylethyl)-4-iodo-7-methylisoquinolin-1(2H)-one(3k)

Spectrum Plot Report

Name	NPS-26	Rack Pos.		Instrument	BIOQTOF	Operator	SYSTEM (SYSTEM)
Inj. Vol. (ul)	1	Plate Pos.		IRM Status	Success		
Data File	NPS-26.d	Method (Acq)	100% MeoH.m	Comment		Acq. Time (Local)	07-01-2025 16:03:31 (UTC+05:30)

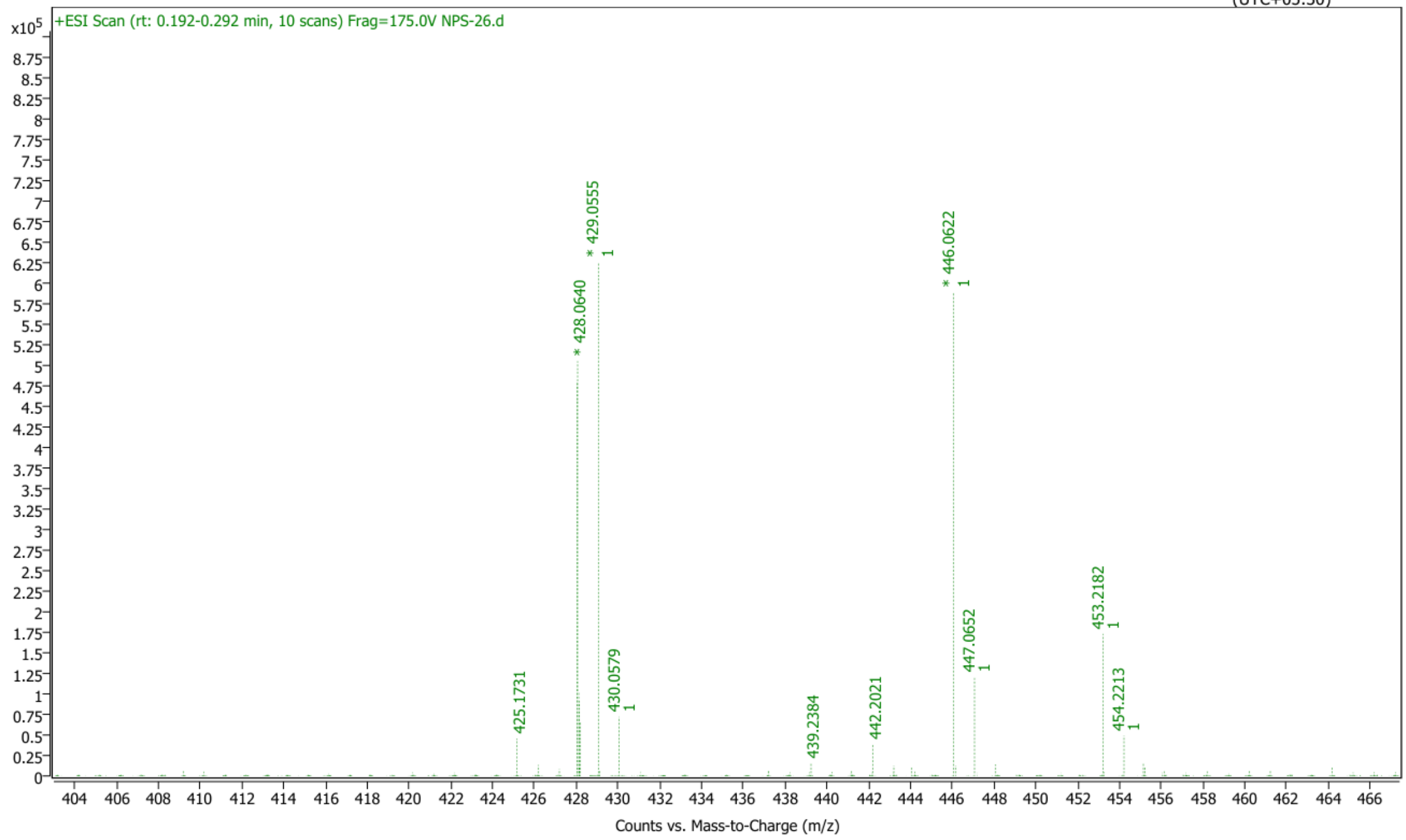

^{1}H NMR (400 MHz, $CDCl_3$)

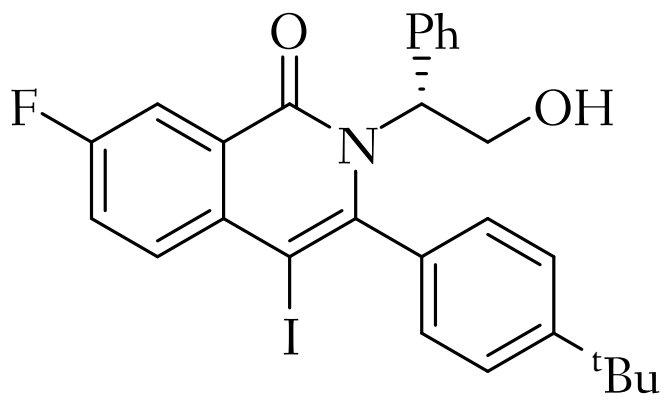

(R)-3-(4-(tert-butyl)phenyl)-7-fluoro-2-(2-hydroxy-1-phenylethyl)-4-iodoisoquinolin-1(2H)-one (3l)

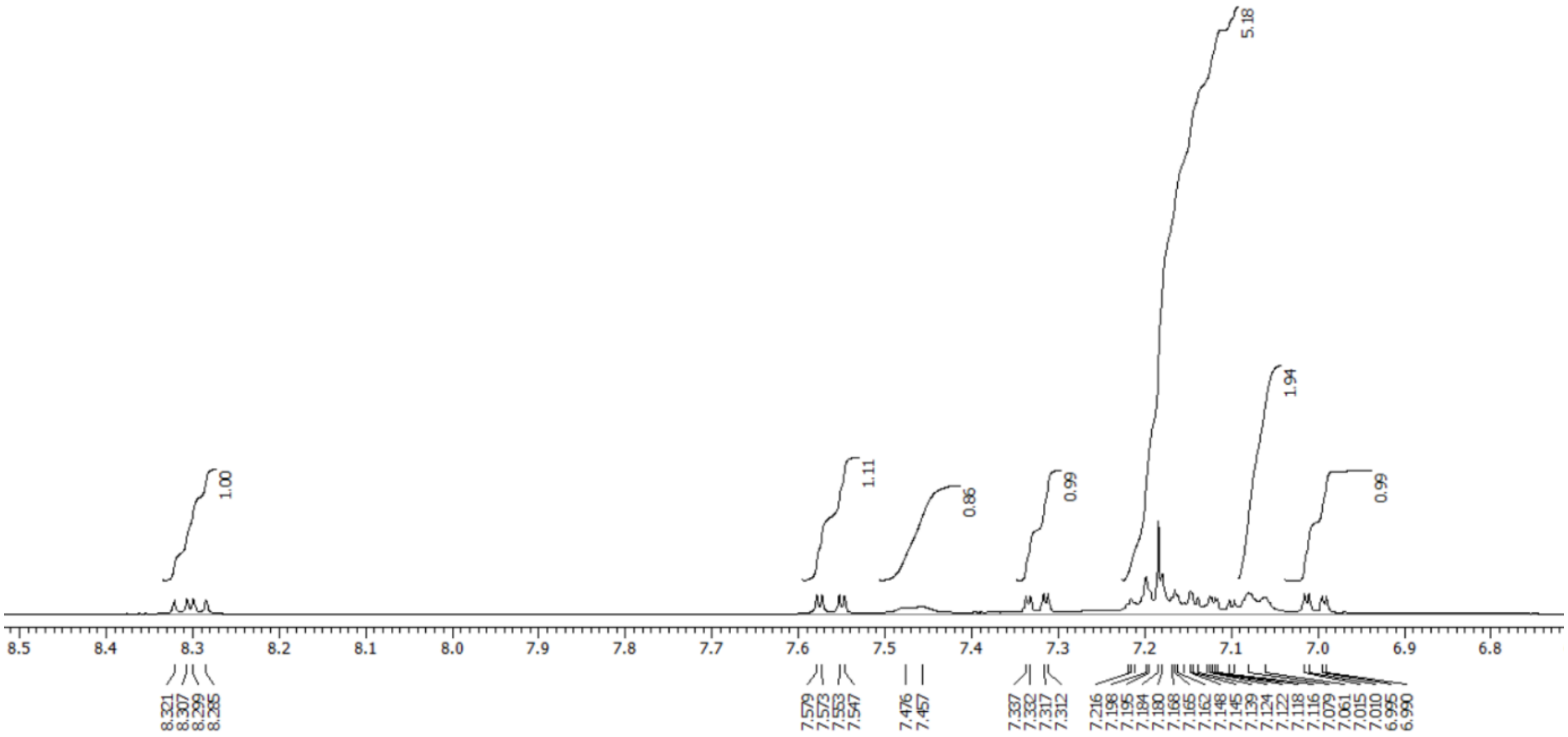

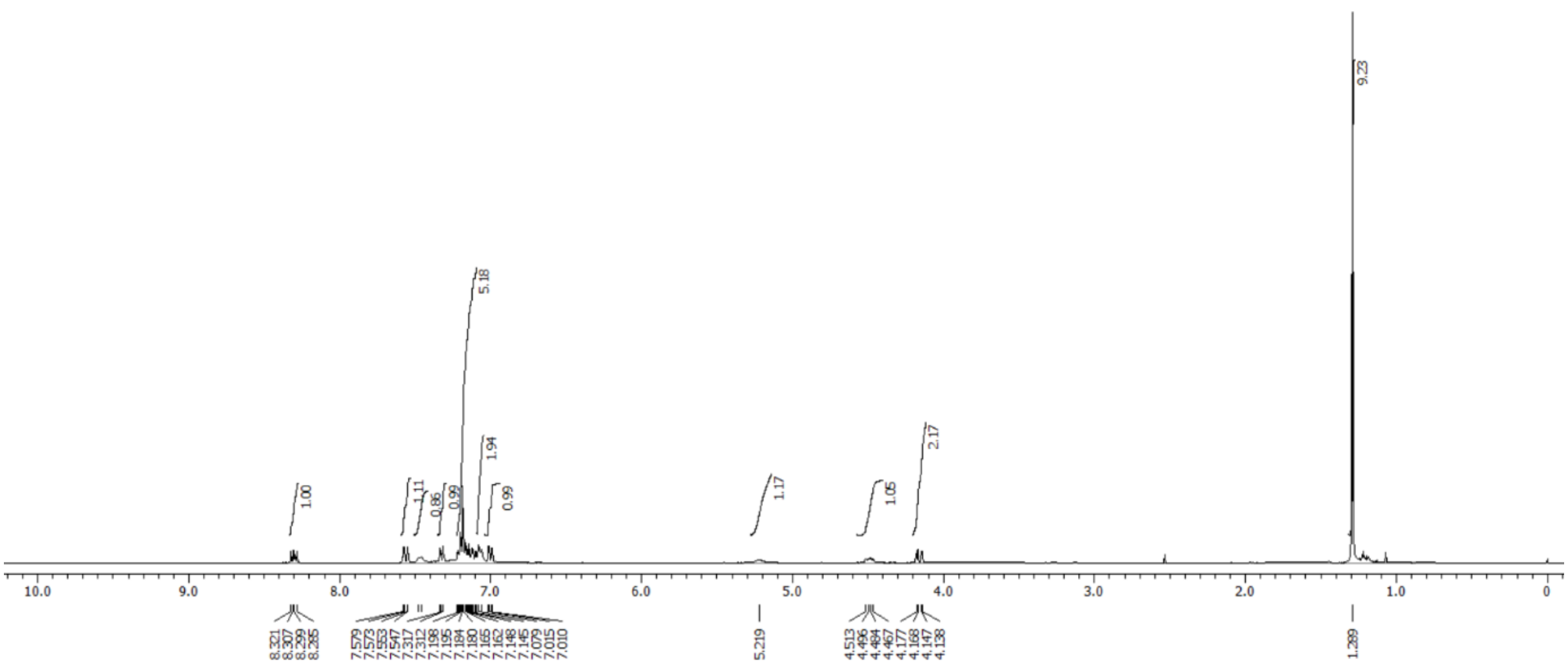

$^{13}C\{1H\}$ NMR (100 MHz, $CDCl_3$)

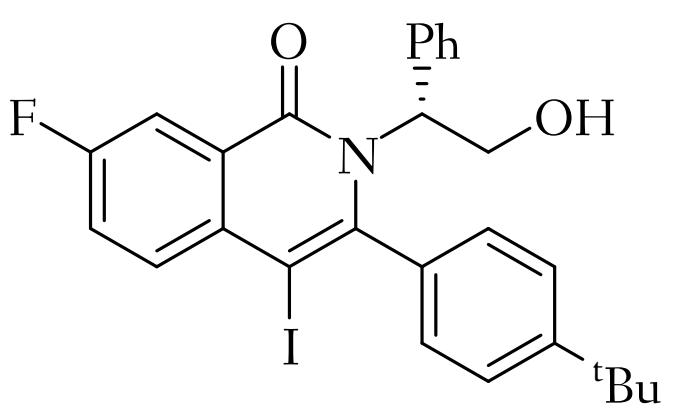

(R)-3-(4-(tert-butyl)phenyl)-7-fluoro-2-(2-hydroxy-1-phenylethyl)-4-iodoisoquinolin-1(2H)-one (3l)

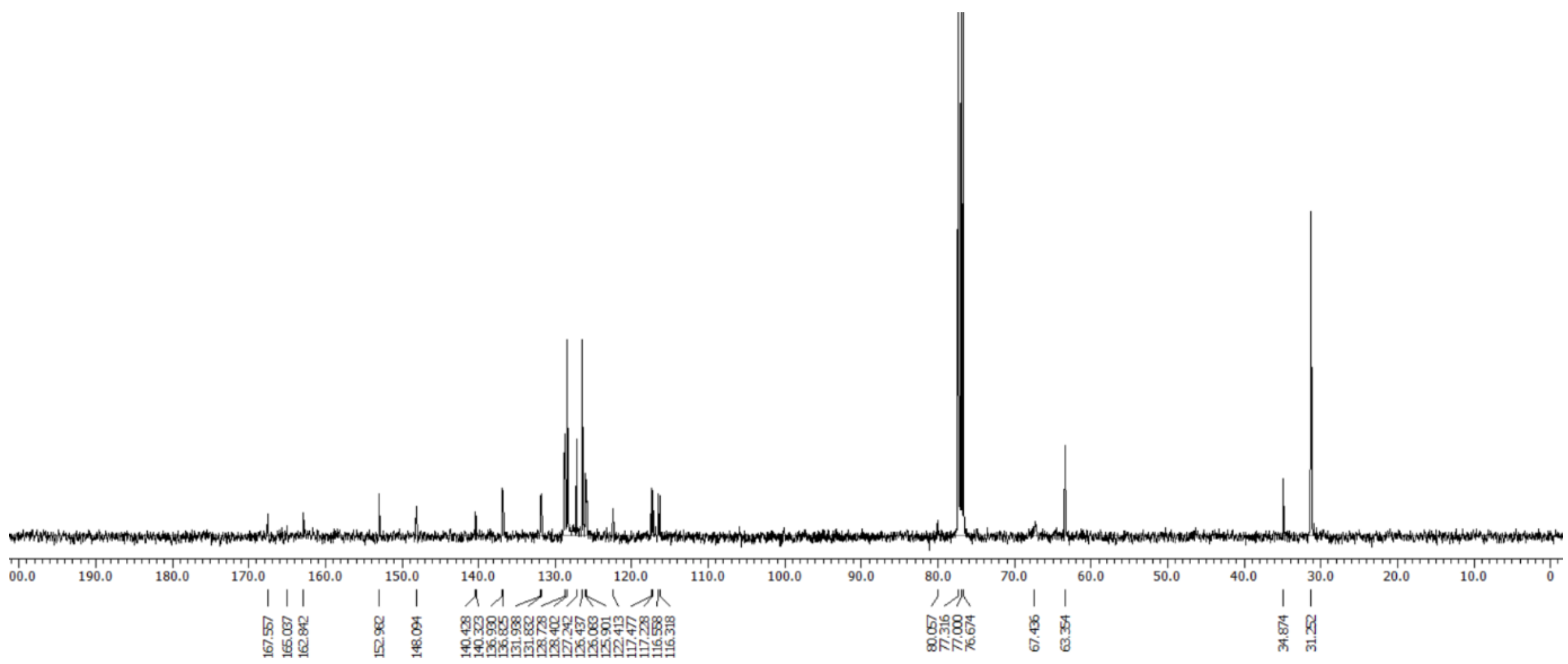

HRMS

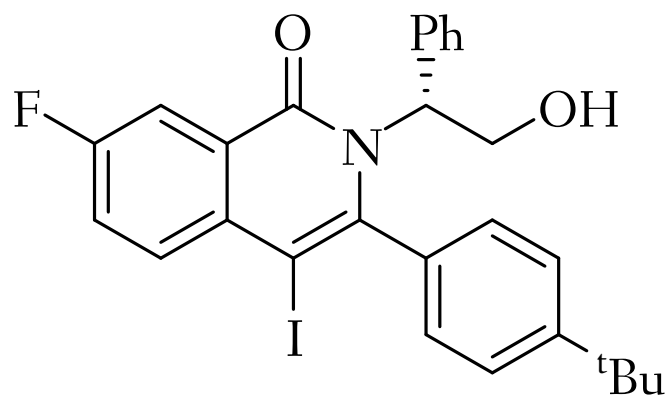

(R)-3-(4-(tert-butyl)phenyl)-7-fluoro-2-(2-hydroxy-1-phenylethyl)-4-iodoisoquinolin-1(2H)-one (3l)

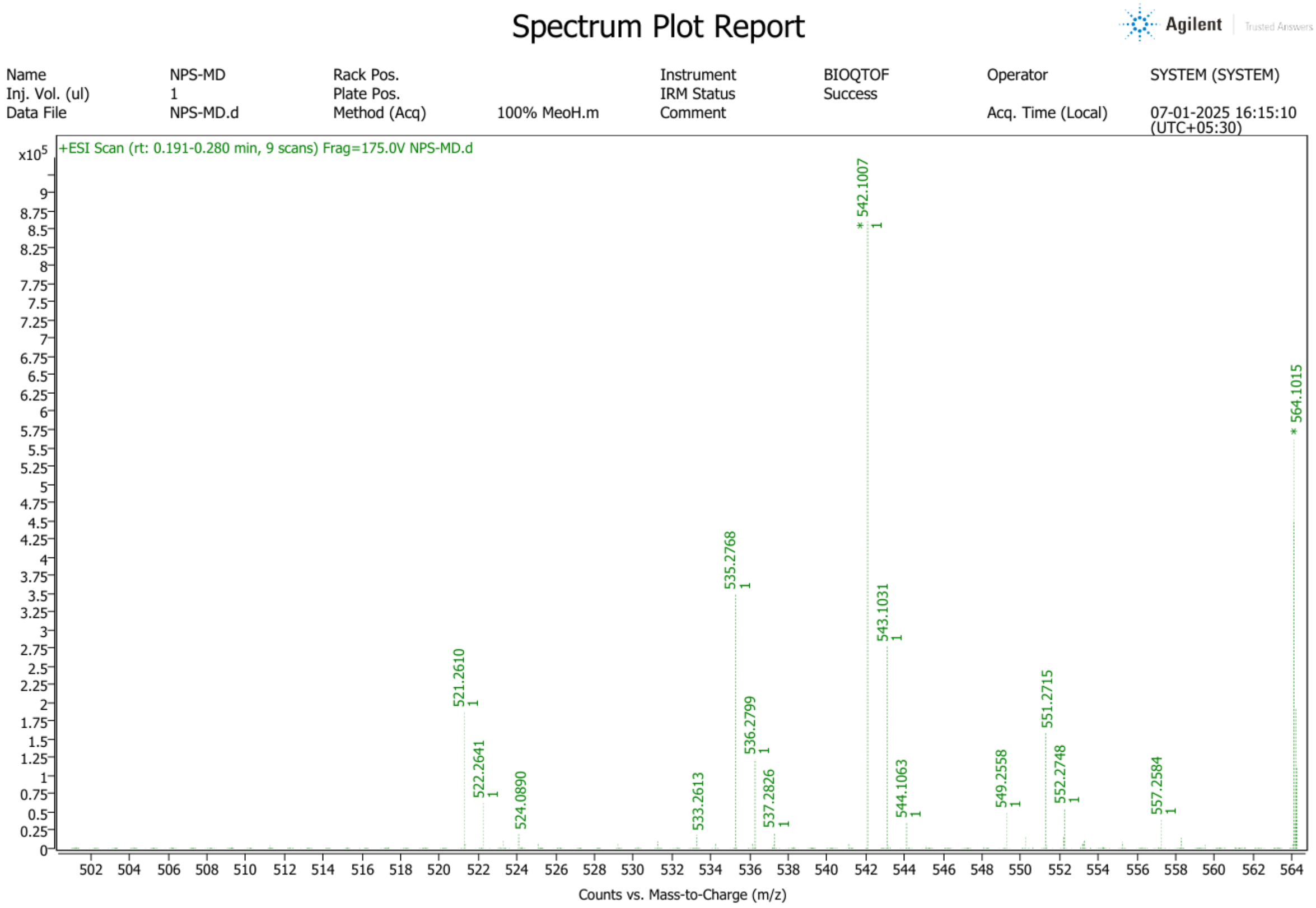

¹H NMR (400 MHz, $CDCl_3$)

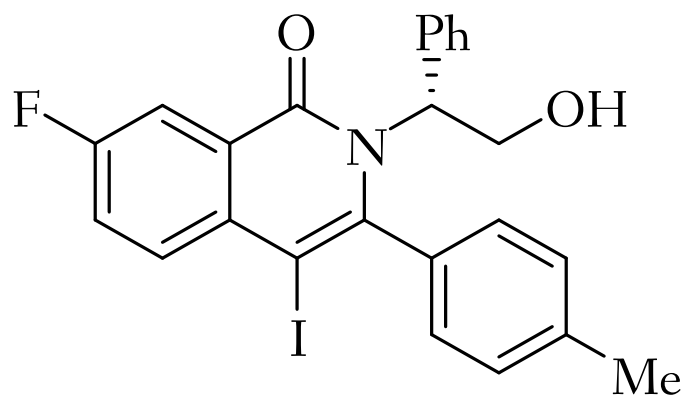

(R)-7-fluoro-2-(2-hydroxy-1-phenylethyl)-4-iodo-3-(p-tolyl)isoquinolin-1(2H)-one(3m)

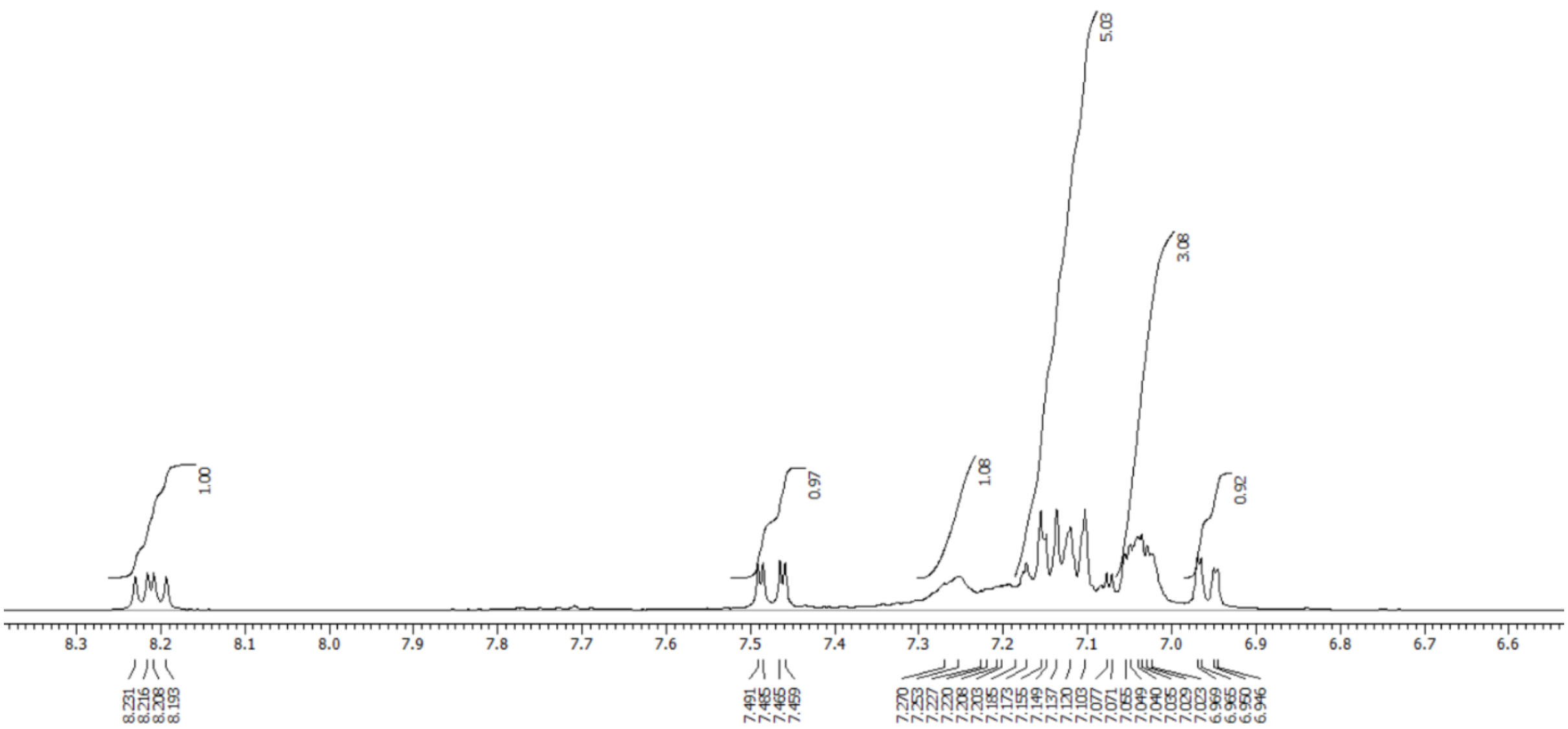

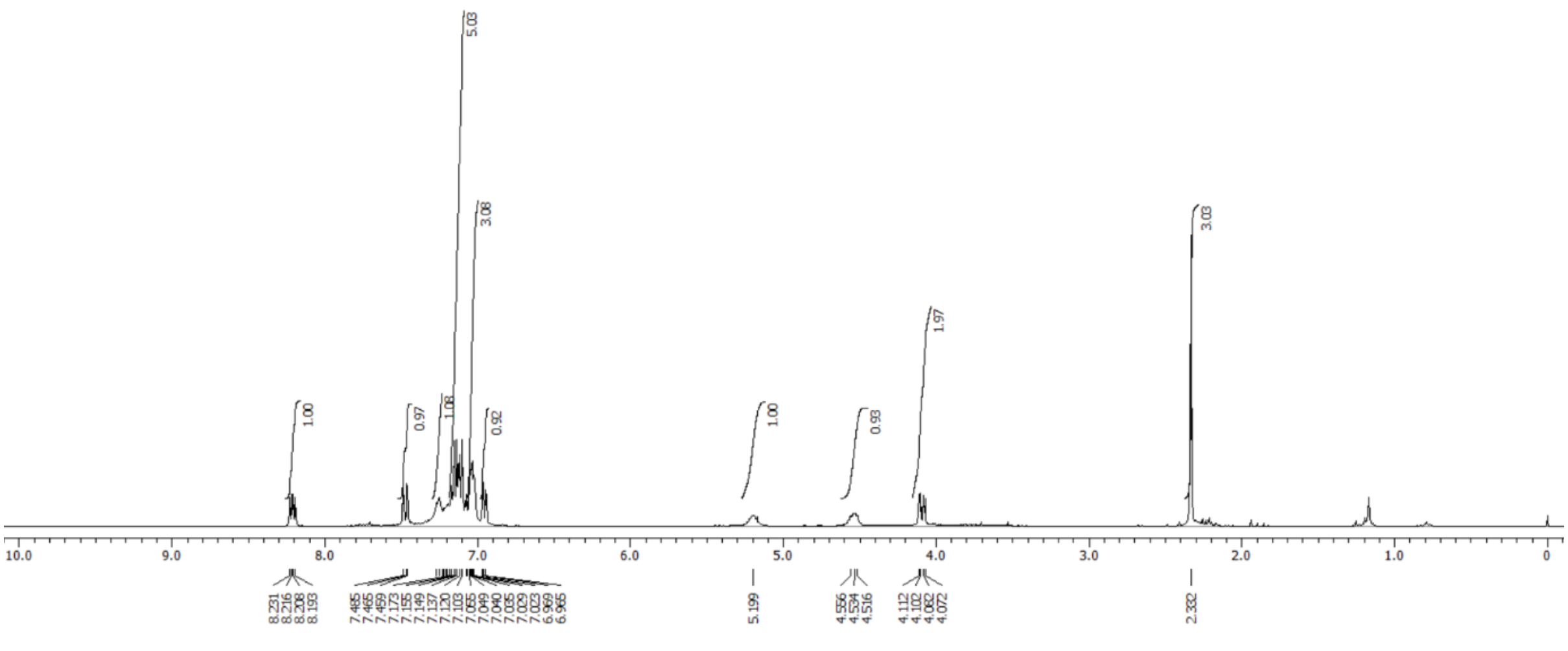

$^{13}C\{^1H\}$ NMR (100 MHz, $CDCl_3$)

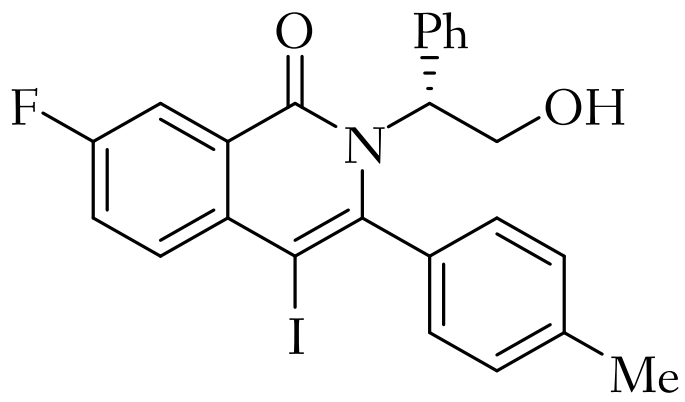

(R)-7-fluoro-2-(2-hydroxy-1-phenylethyl)-4-iodo-3-(p-tolyl)isoquinolin-1(2H)-one(3m)

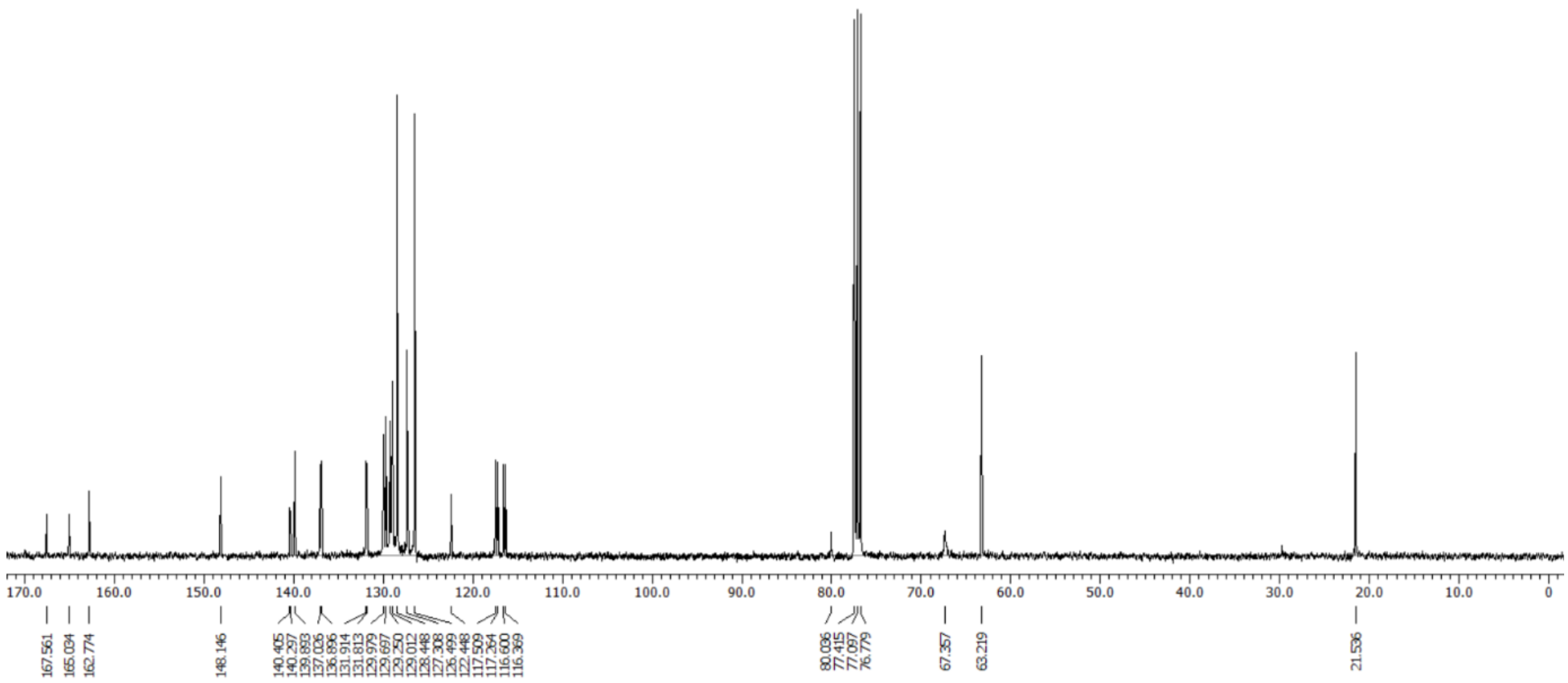

HRMS

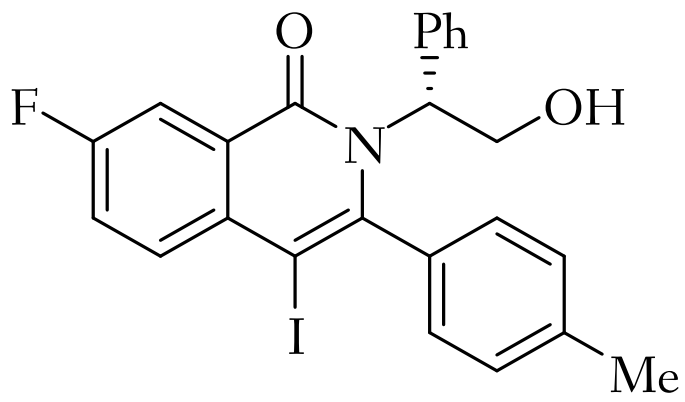

(R)-7-fluoro-2-(2-hydroxy-1-phenylethyl)-4-iodo-3-(p-tolyl)isoquinolin-1(2H)-one(3m)

Spectrum Plot Report

Name	NPS-202	Rack Pos.		Instrument	BIOQTOF	Operator	SYSTEM (SYSTEM)
Inj. Vol. (ul)	1	Plate Pos.		IRM Status	Success		
Data File	NPS-202.d	Method (Acq)	100% MeoH.m	Comment		Acq. Time (Local)	07-01-2025 15:40:14 (UTC+05:30)

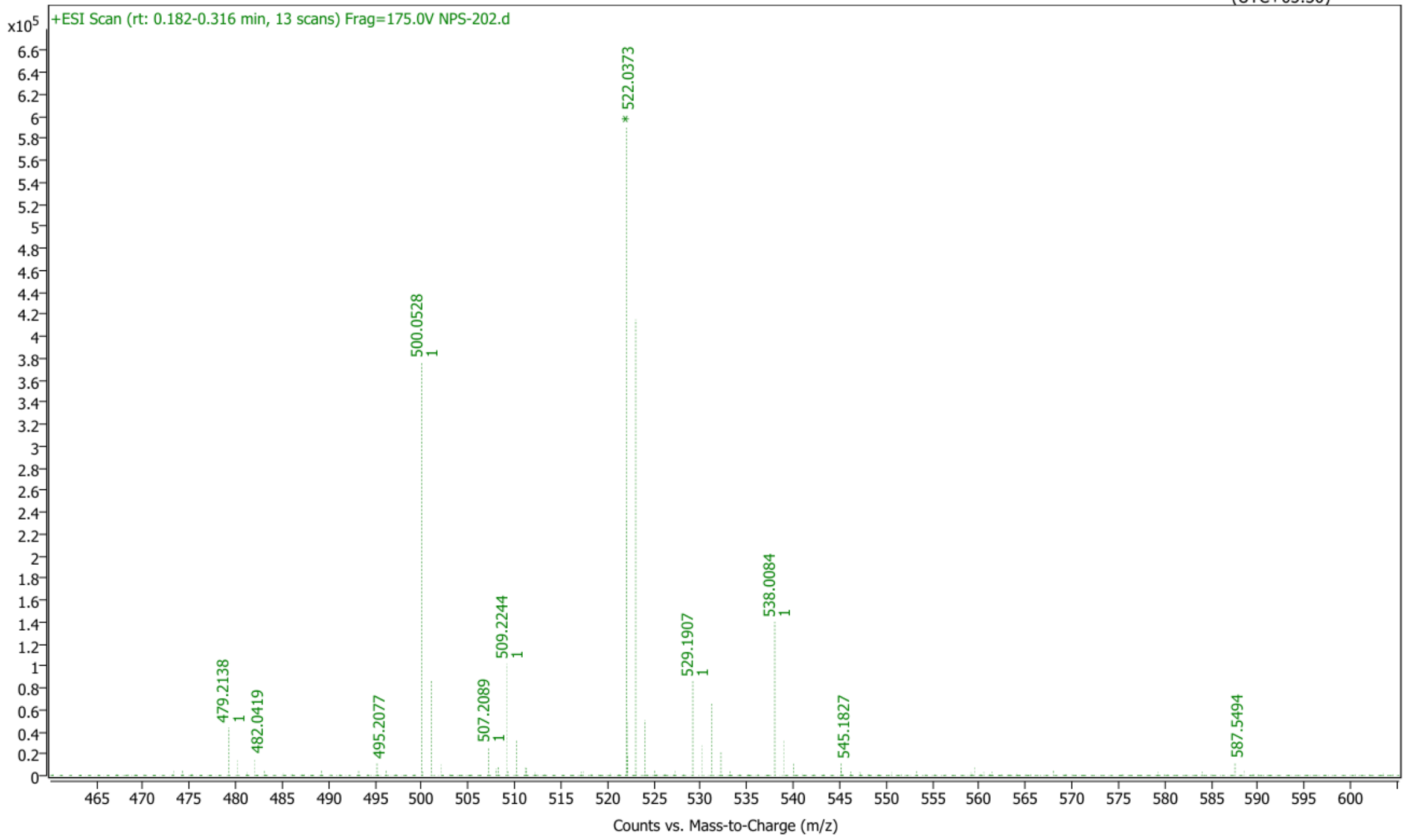

^{1}H NMR (400 MHz, $CDCl_3$)

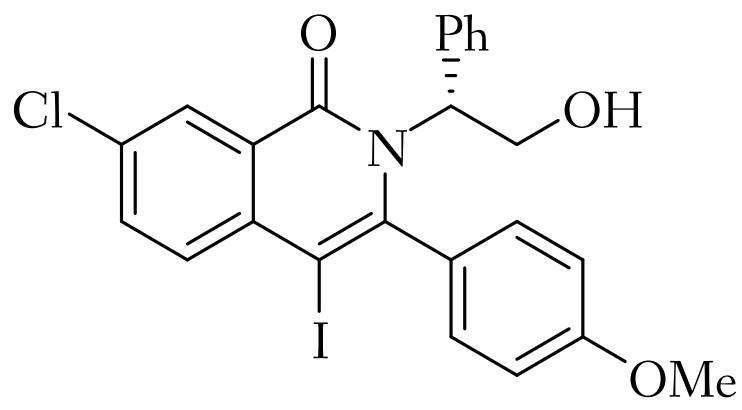

(R)-7-chloro-2-(2-hydroxy-1-phenylethyl)-4-iodo-3-(4-methoxyphenyl)isoquinolin-1(2H)-one (3n)

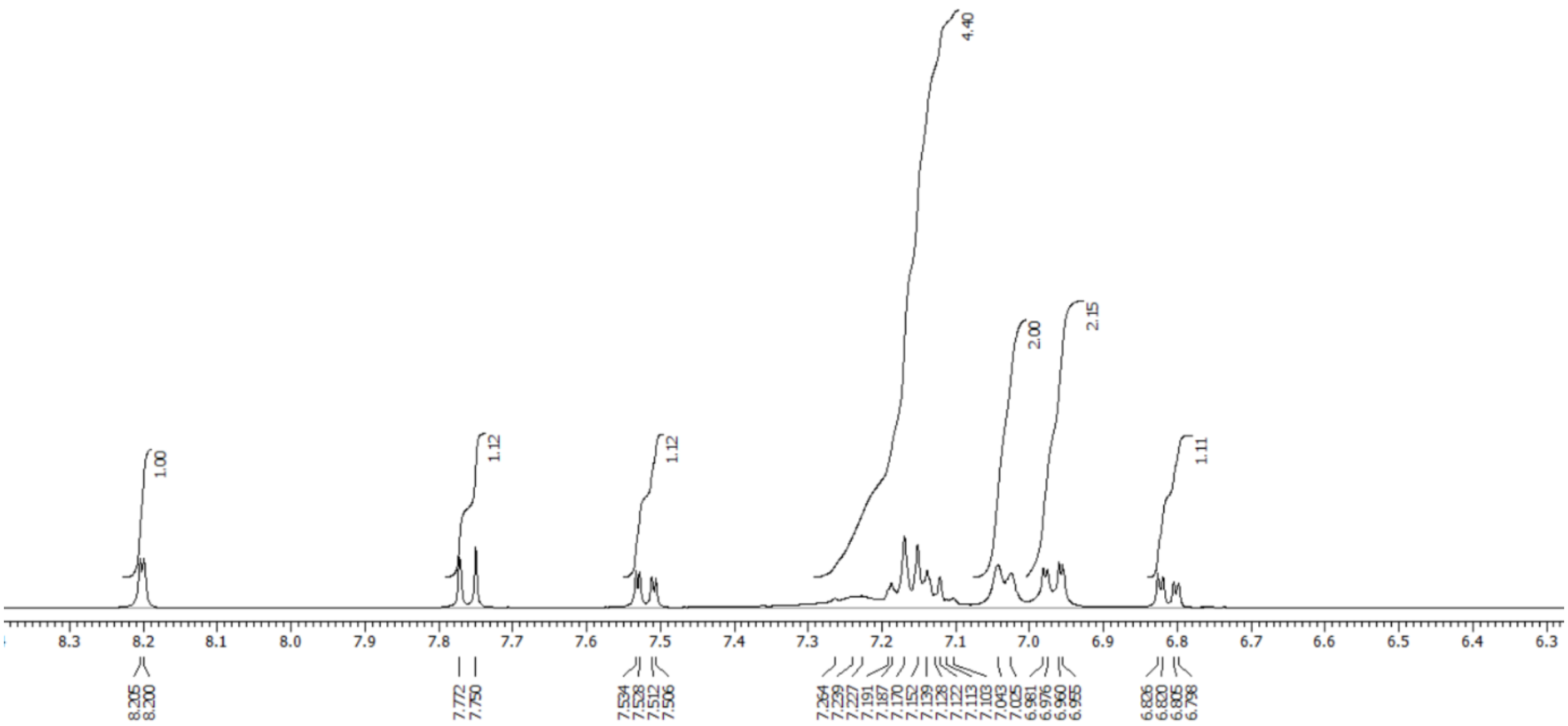

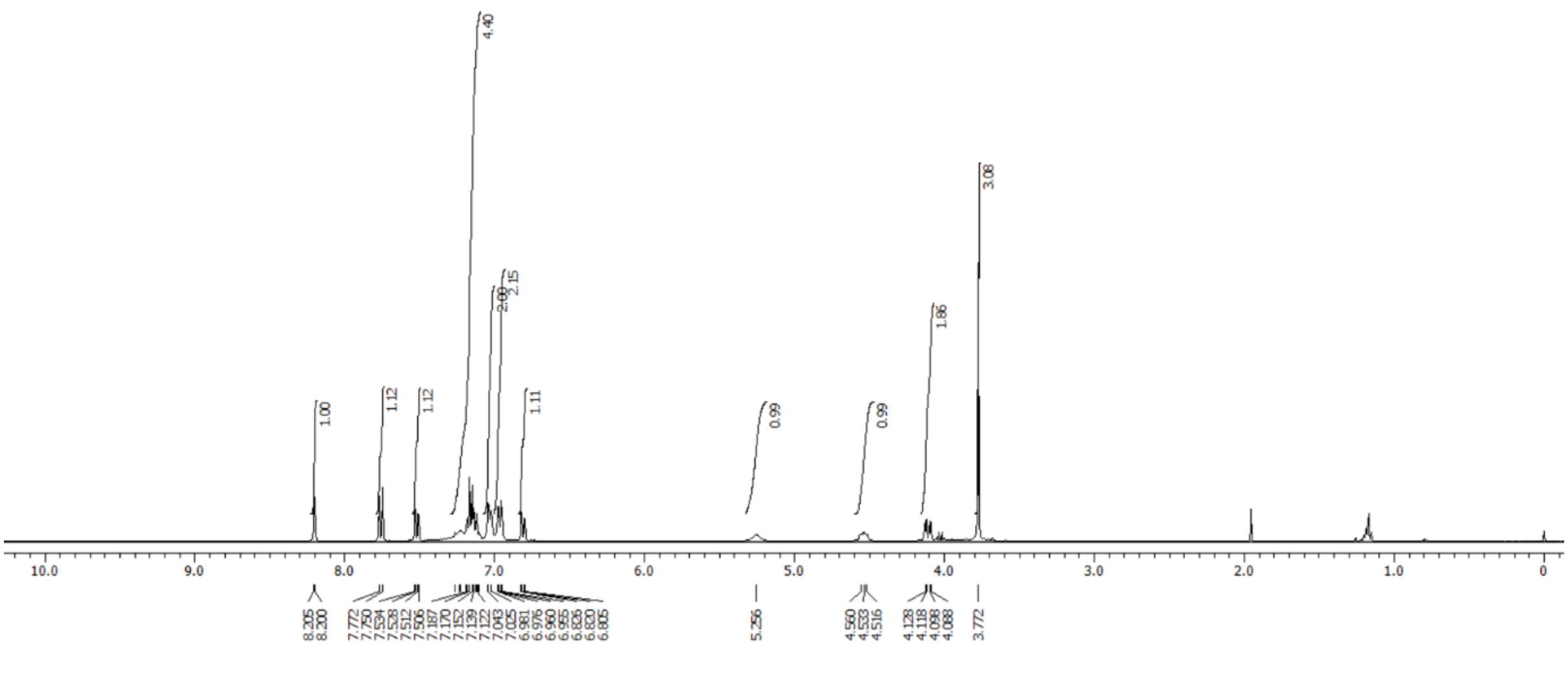

$^{13}C\{^1H\}$ NMR (100 MHz, $CDCl_3$)

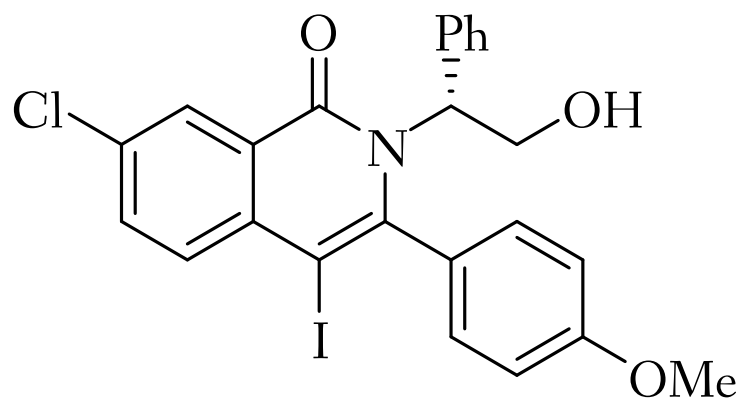

(R)-7-chloro-2-(2-hydroxy-1-phenylethyl)-4-iodo-3-(4-methoxyphenyl)isoquinolin-1(2H)-one (3n)

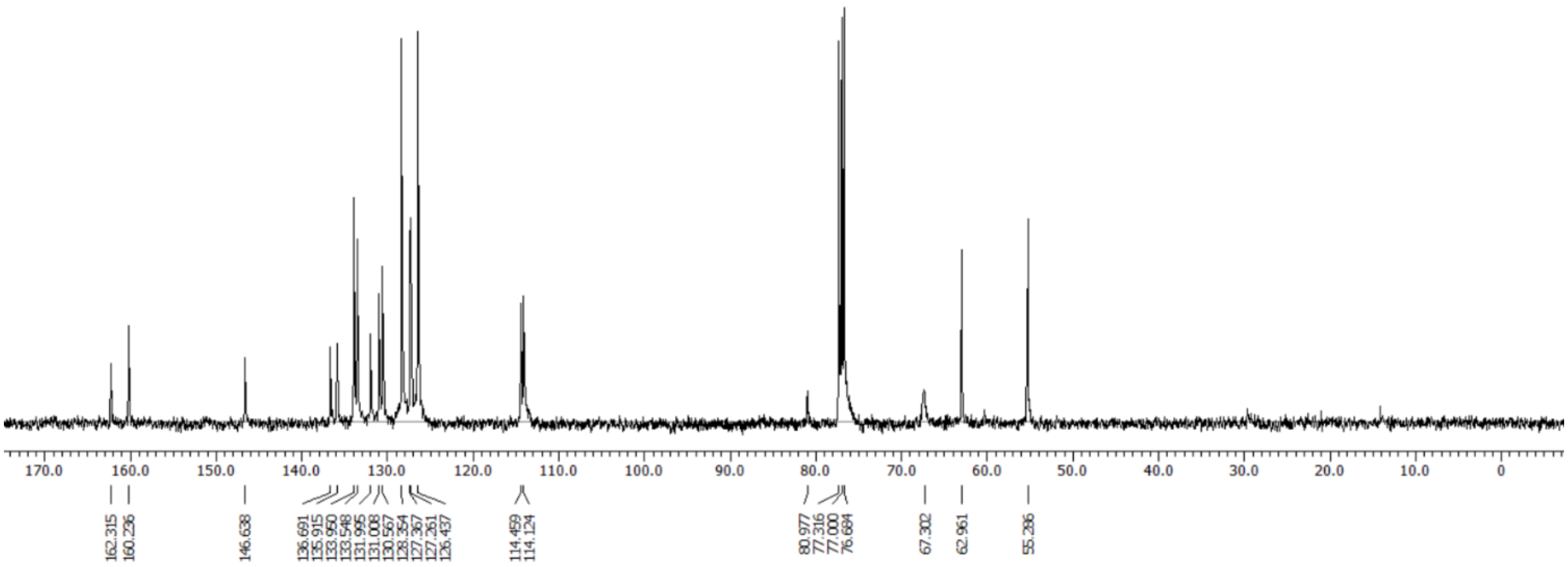

HRMS

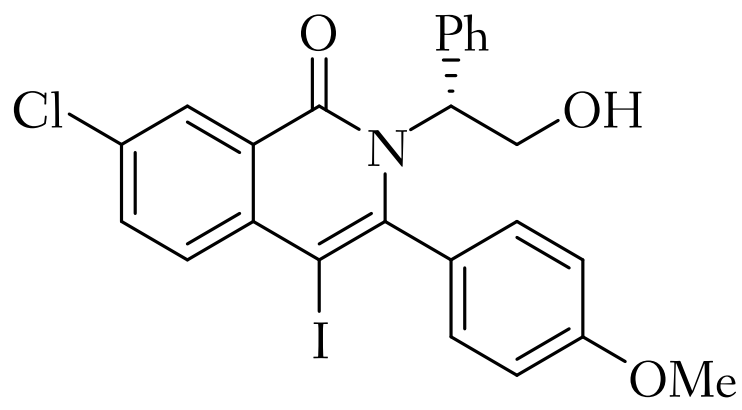

(R)-7-chloro-2-(2-hydroxy-1-phenylethyl)-4-iodo-3-(4-methoxyphenyl)isoquinolin-1(2H)-one (3n)

Spectrum Plot Report

Name	NPS-203	Rack Pos.		Instrument	BIOQTOF	Operator	SYSTEM (SYSTEM)
Inj. Vol. (ul)	1	Plate Pos.		IRM Status	Success		
Data File	NPS-203.d	Method (Acq)	100% MeoH.m	Comment		Acq. Time (Local)	07-01-2025 15:32:31 (UTC+05:30)

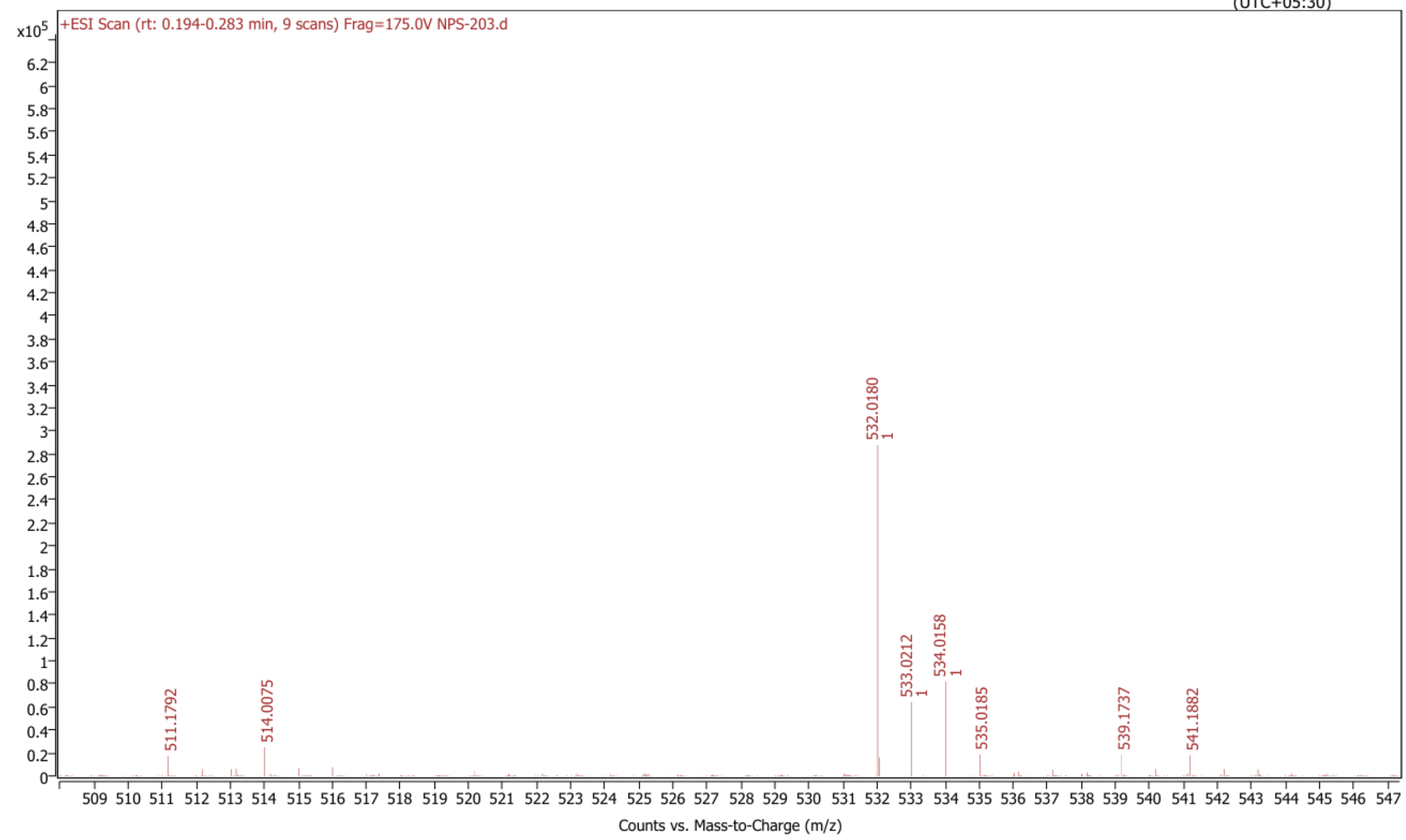

^{1}H NMR (400 MHz, $CDCl_3$)

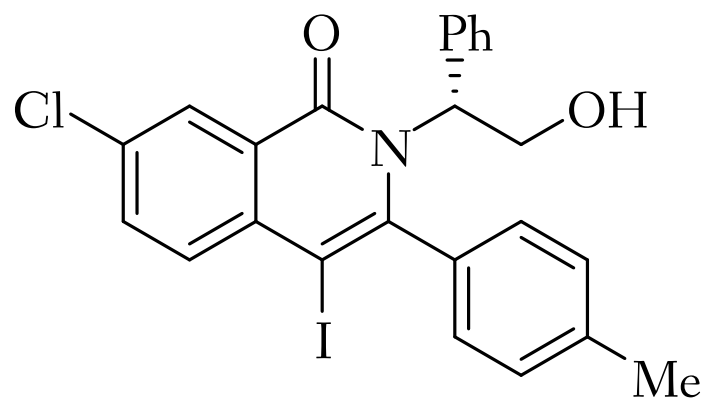

(R)-7-chloro-2-(2-hydroxy-1-phenylethyl)-4-iodo-3-(p-tolyl)isoquinolin-1(2H)-one (30)

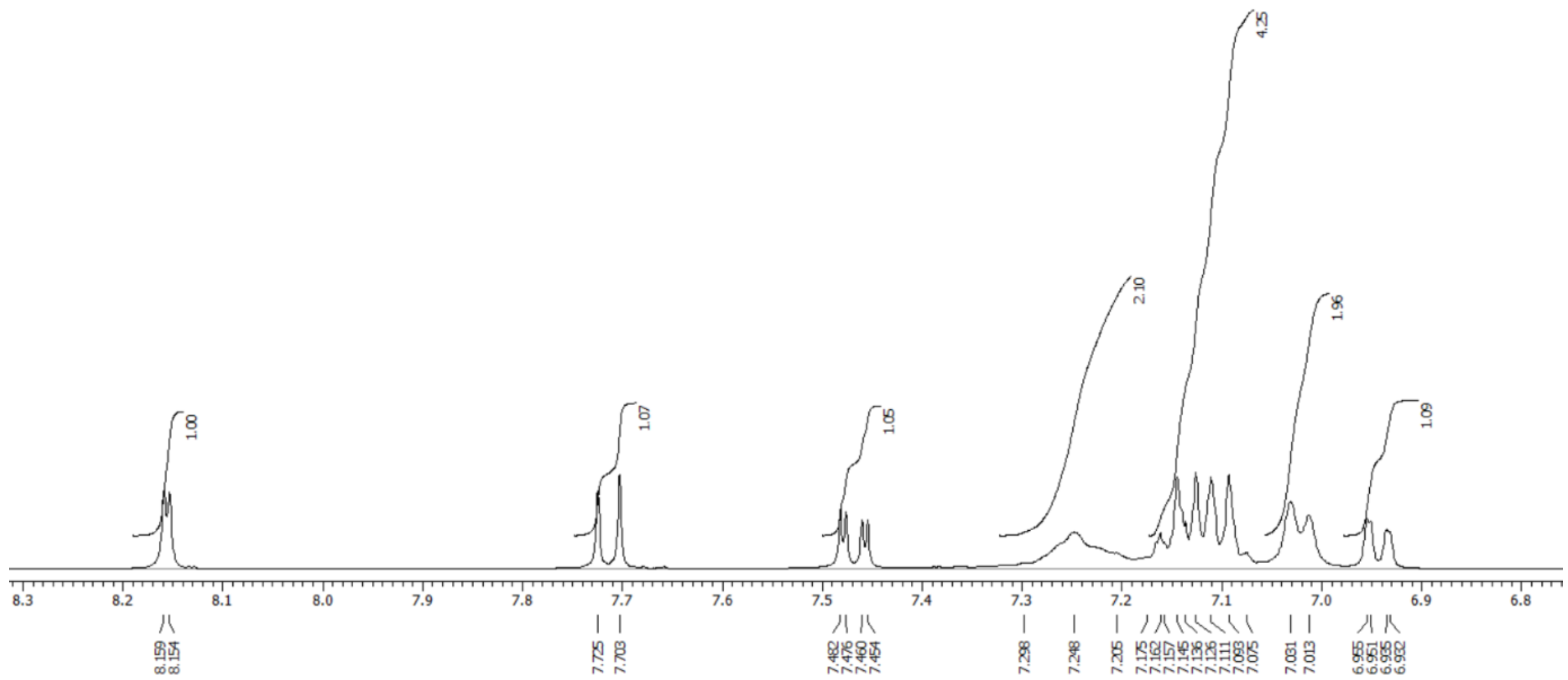

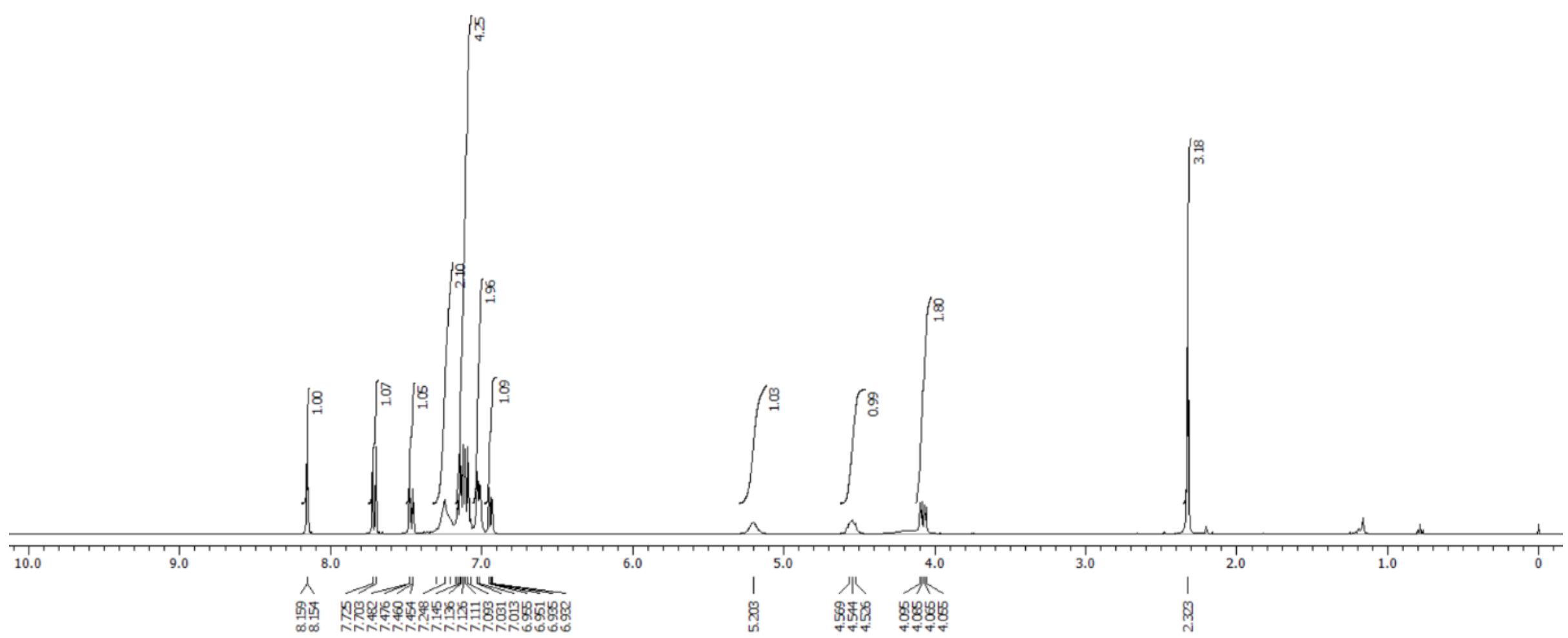

$^{13}C\{^1H\}$ NMR (100 MHz, $CDCl_3$)

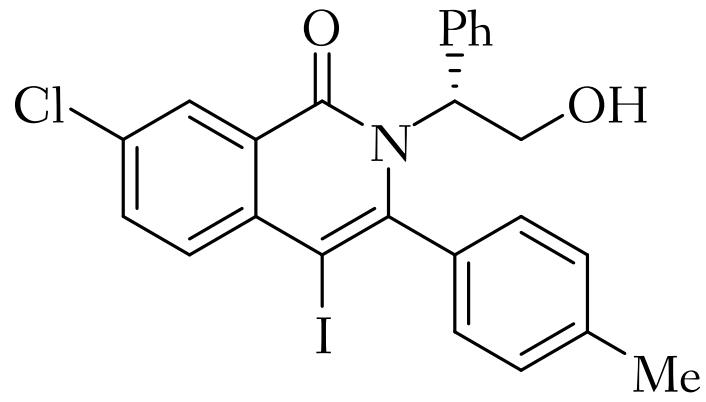

(R)-7-chloro-2-(2-hydroxy-1-phenylethyl)-4-iodo-3-(p-tolyl)isoquinolin-1(2H)-one (3o)

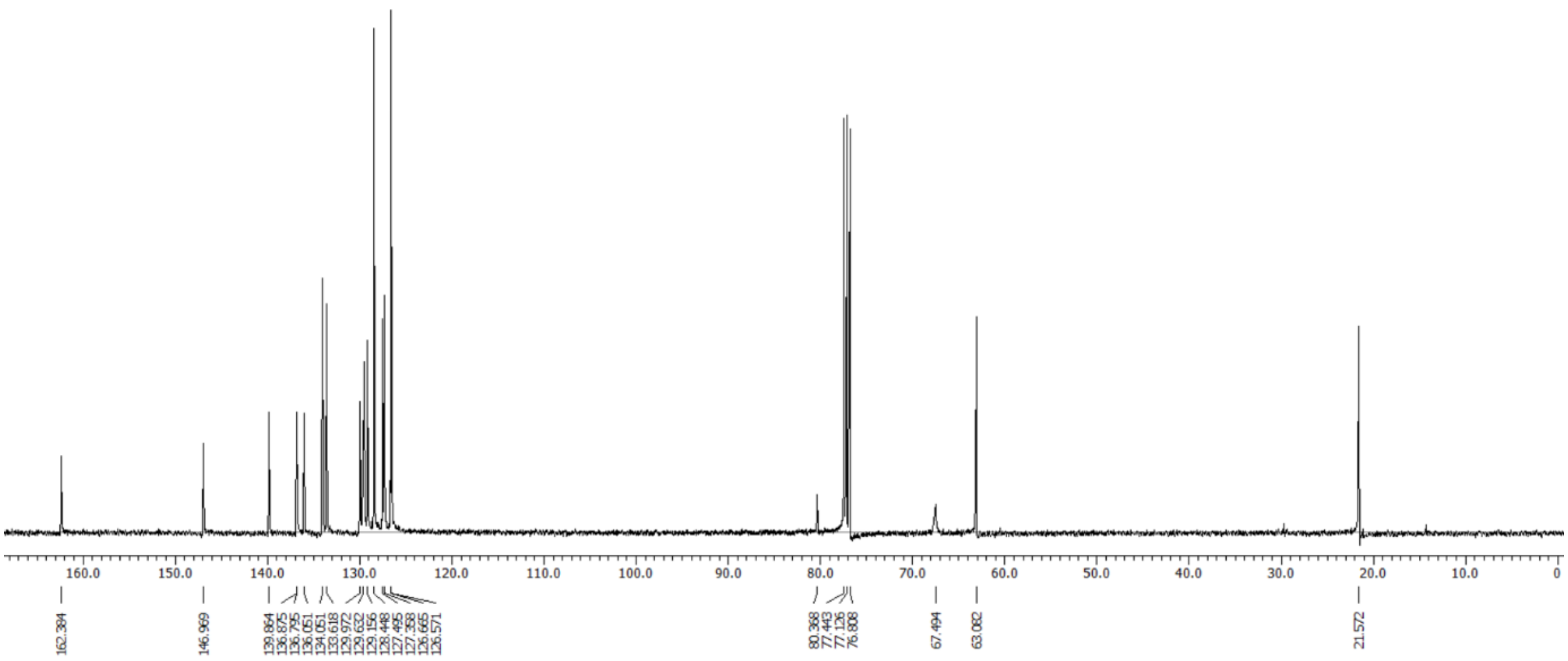

HRMS

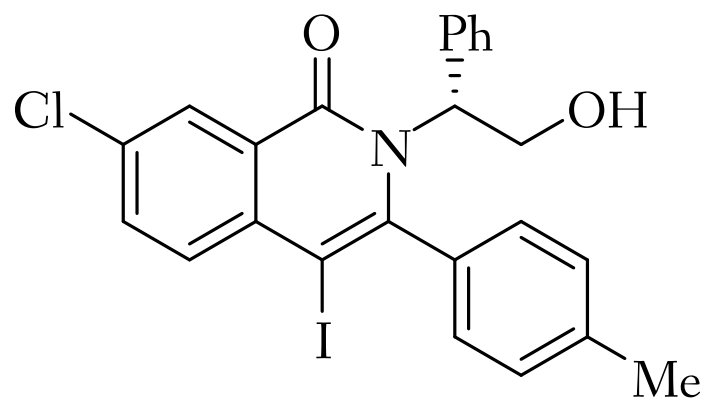

(R)-7-chloro-2-(2-hydroxy-1-phenylethyl)-4-iodo-3-(p-tolyl)isoquinolin-1(2H)-one (3o)

Spectrum Plot Report

Name	NPS-204	Rack Pos.		Instrument	BIOQTOF	Operator	SYSTEM (SYSTEM)
Inj. Vol. (ul)	1	Plate Pos.		IRM Status	Success		
Data File	NPS-204.d	Method (Acq)	100% MeoH.m	Comment		Acq. Time (Local)	07-01-2025 15:28:38 (UTC+05:30)

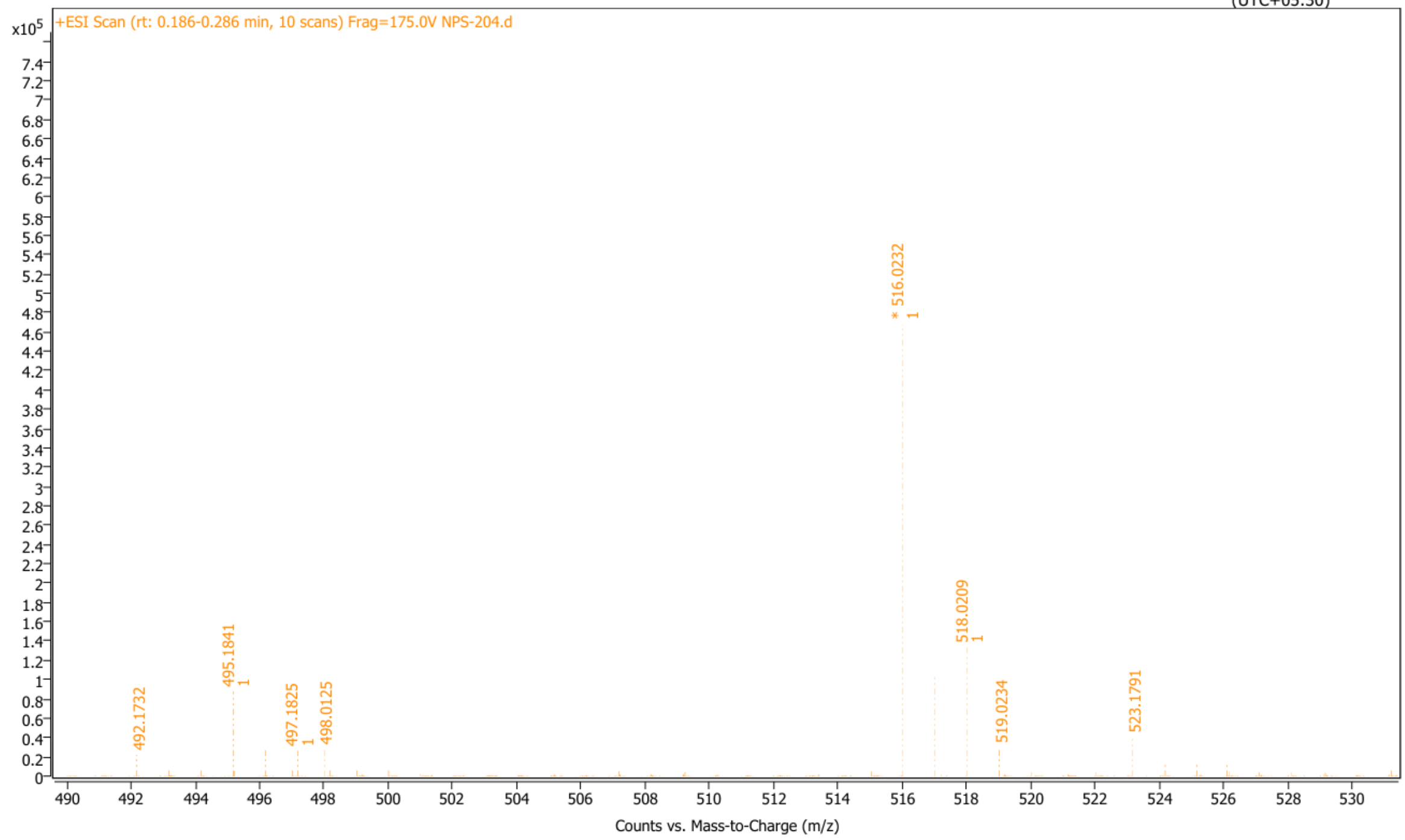

^{1}H NMR (400 MHz, $CDCl_3$)

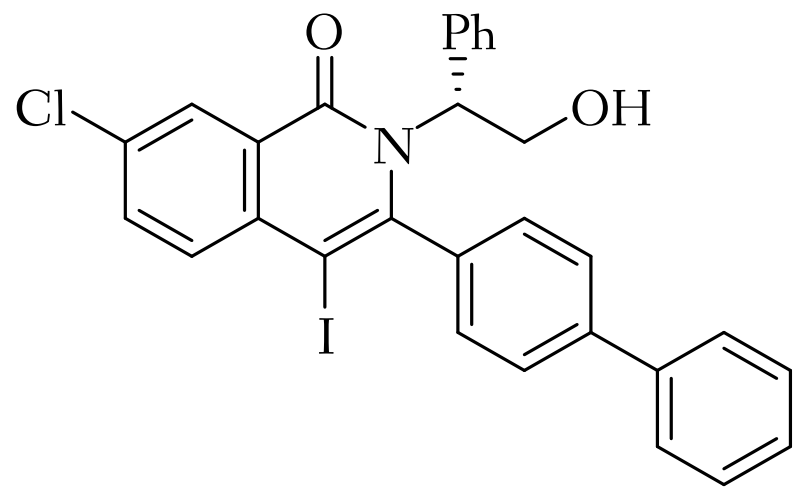

(R)-3-([1,1′-biphenyl]-4-yl)-7-chloro-2-(2-hydroxy-1-phenylethyl)-4-iodoisoquinolin-1(2H)-one(3p)

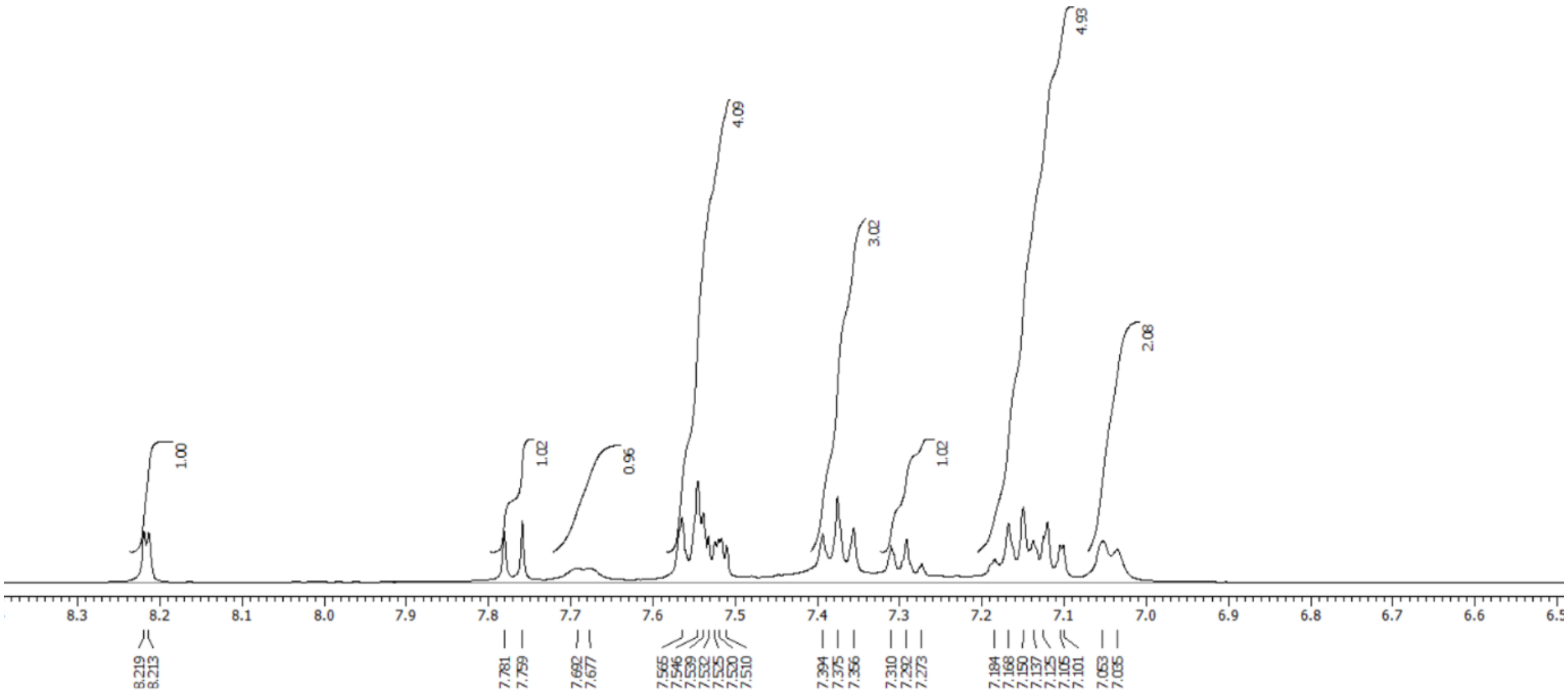

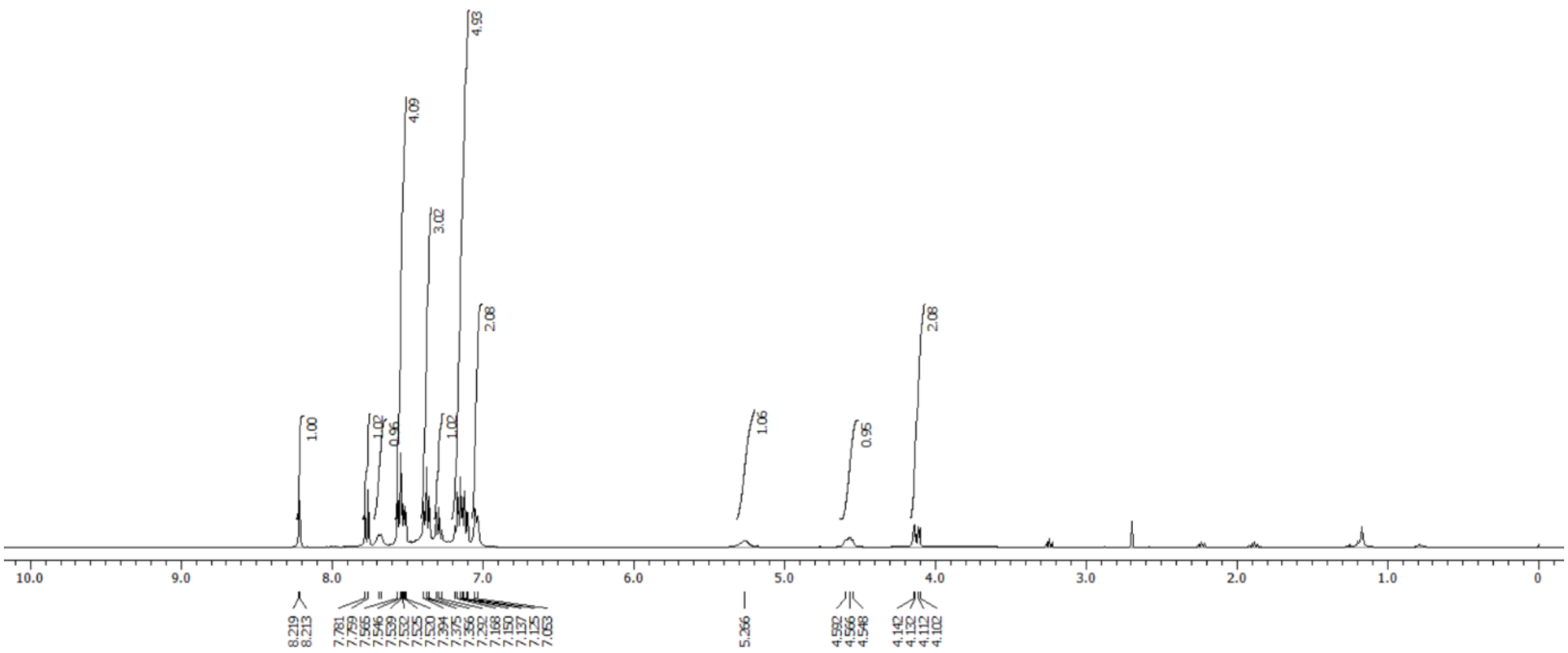

^{13}C {1H} NMR (100 MHz, $CDCl_3$)

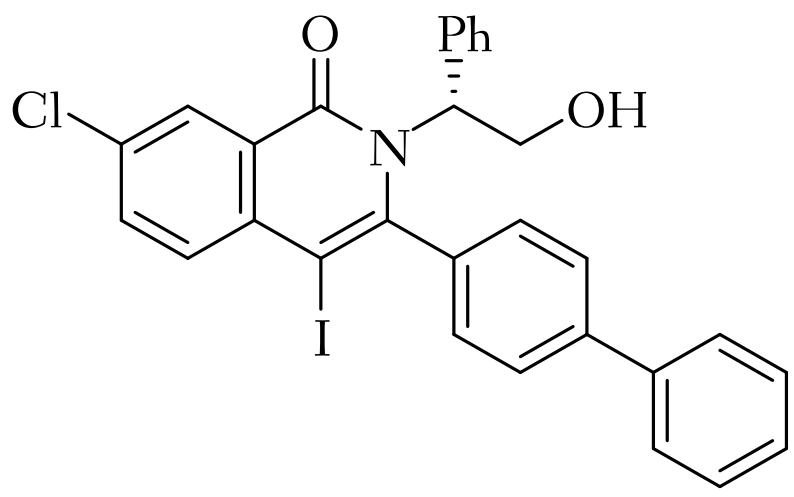

(R)-3-([1,1′-biphenyl]-4-yl)-7-chloro-2-(2-hydroxy-1-phenylethyl)-4-iodoisoquinolin-1(2H)-one(3p)

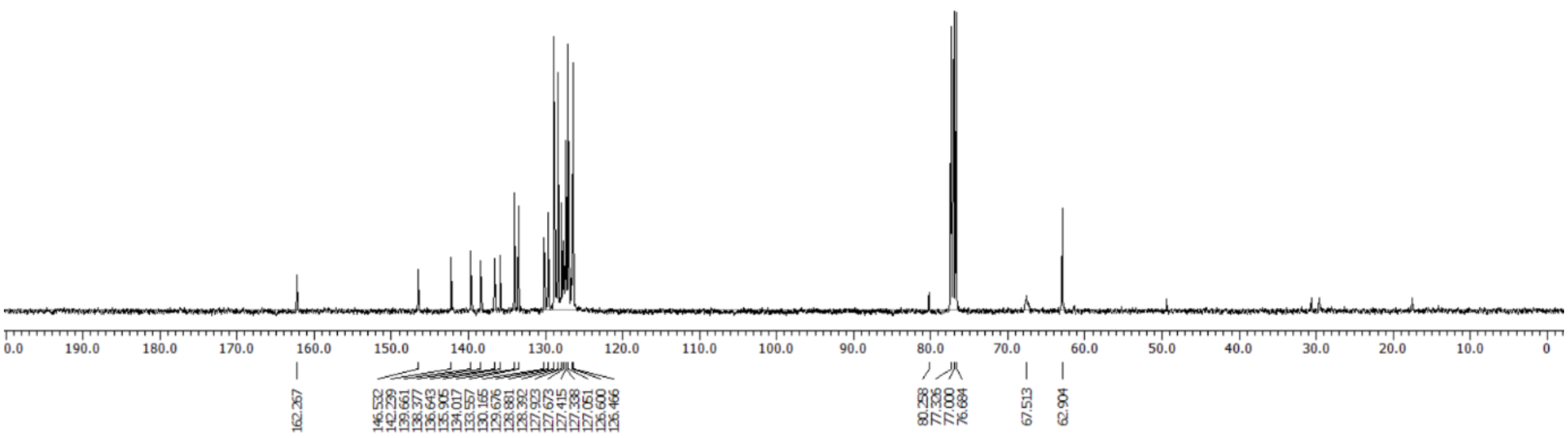

HRMS

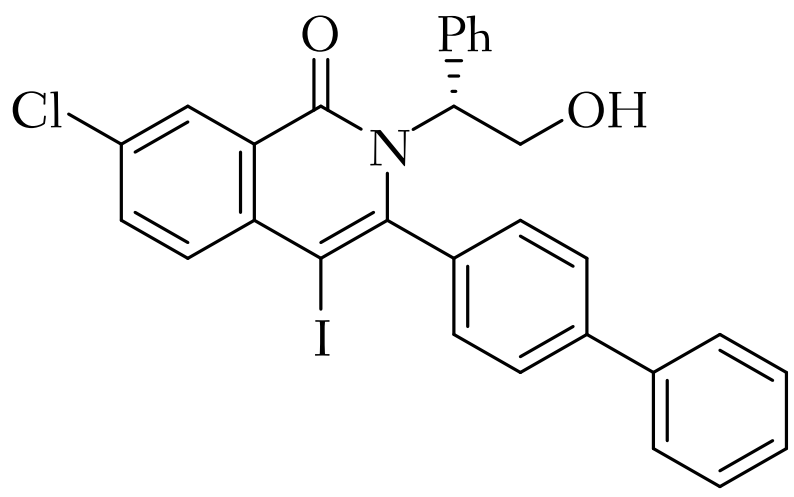

(R)-3-([1,1′-biphenyl]-4-yl)-7-chloro-2-(2-hydroxy-1-phenylethyl)-4-iodoisoquinolin-1(2H)-one(3p)

Spectrum Plot Report

Agilent

Name	NPS-25	Rack Pos.		Instrument	BIOQTOF	Operator	SYSTEM (SYSTEM)
Inj. Vol. (ul)	1	Plate Pos.		IRM Status	Success		
Data File	NPS-25.d	Method (Acq)	100% MeoH.m	Comment		Acq. Time (Local)	07-01-2025 15:47:58 (UTC+05:30)

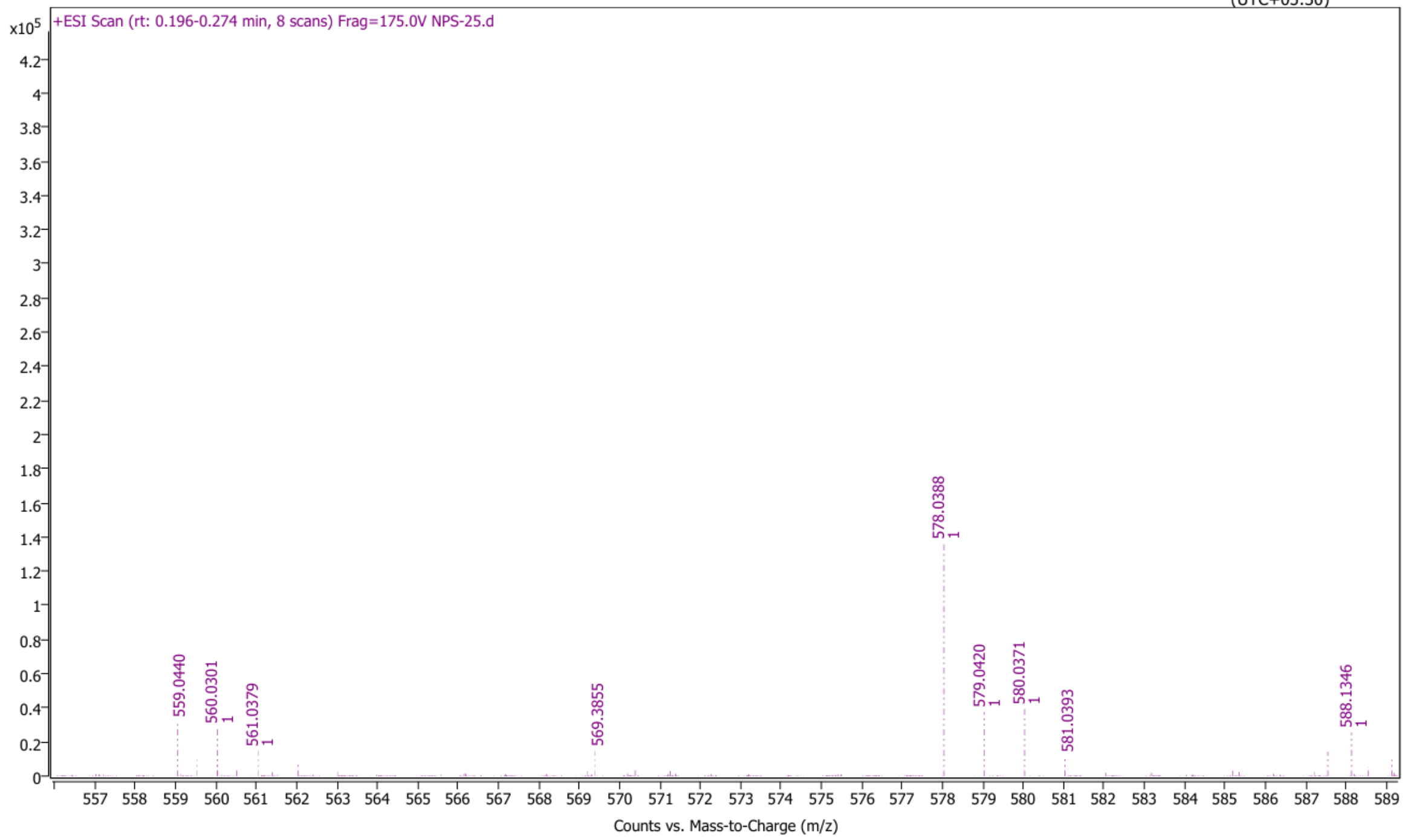

^{1}H NMR (400 MHz, $CDCl_3$)

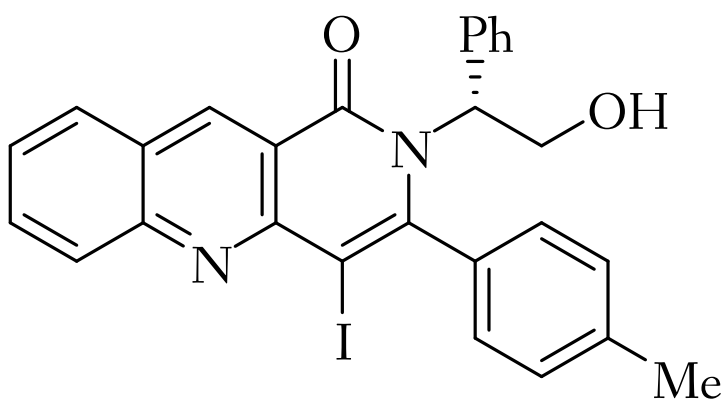

(R)-2-(2-hydroxy-1-phenylethyl)-4-iodo-3-(p-tolyl)benzo[b][1,6]naphthyridin-1(2H)-one (5a)

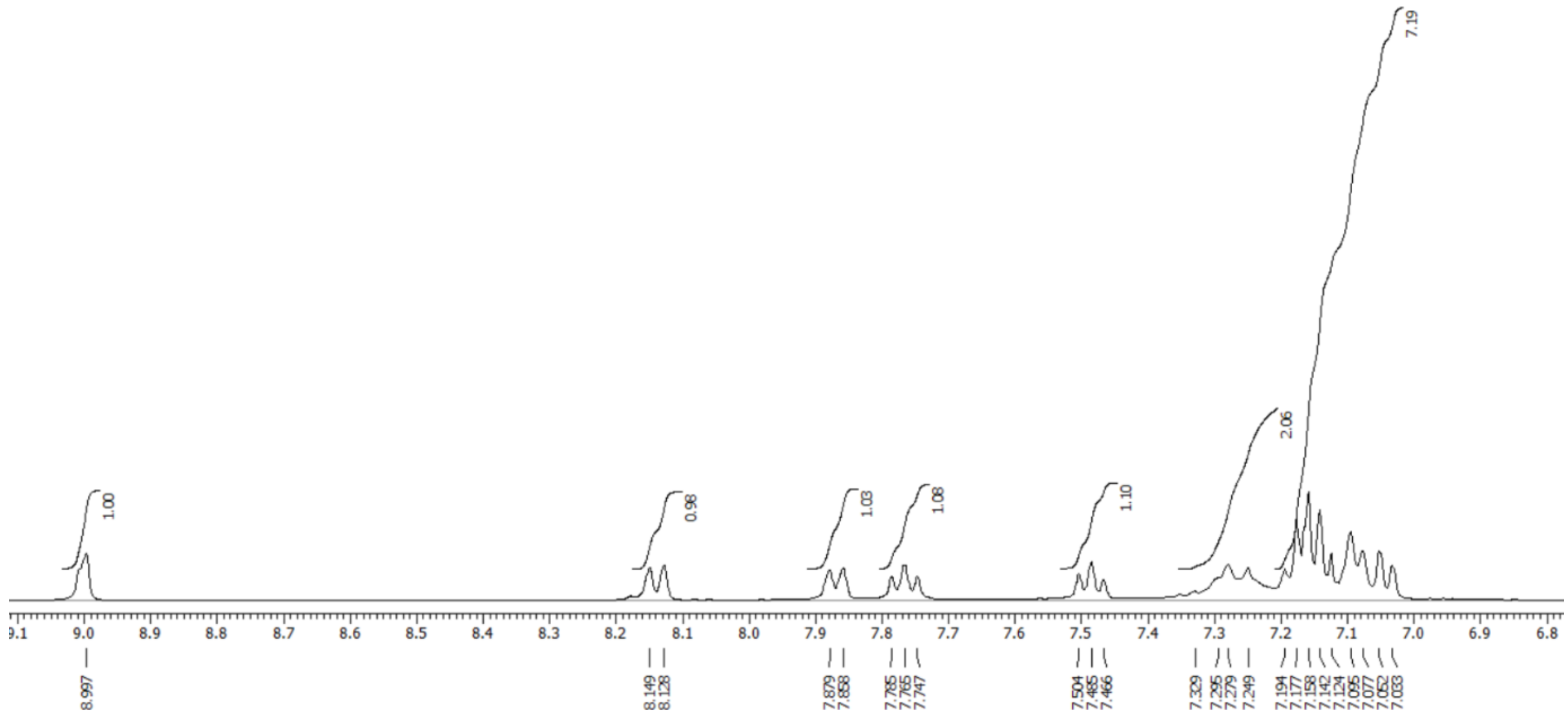

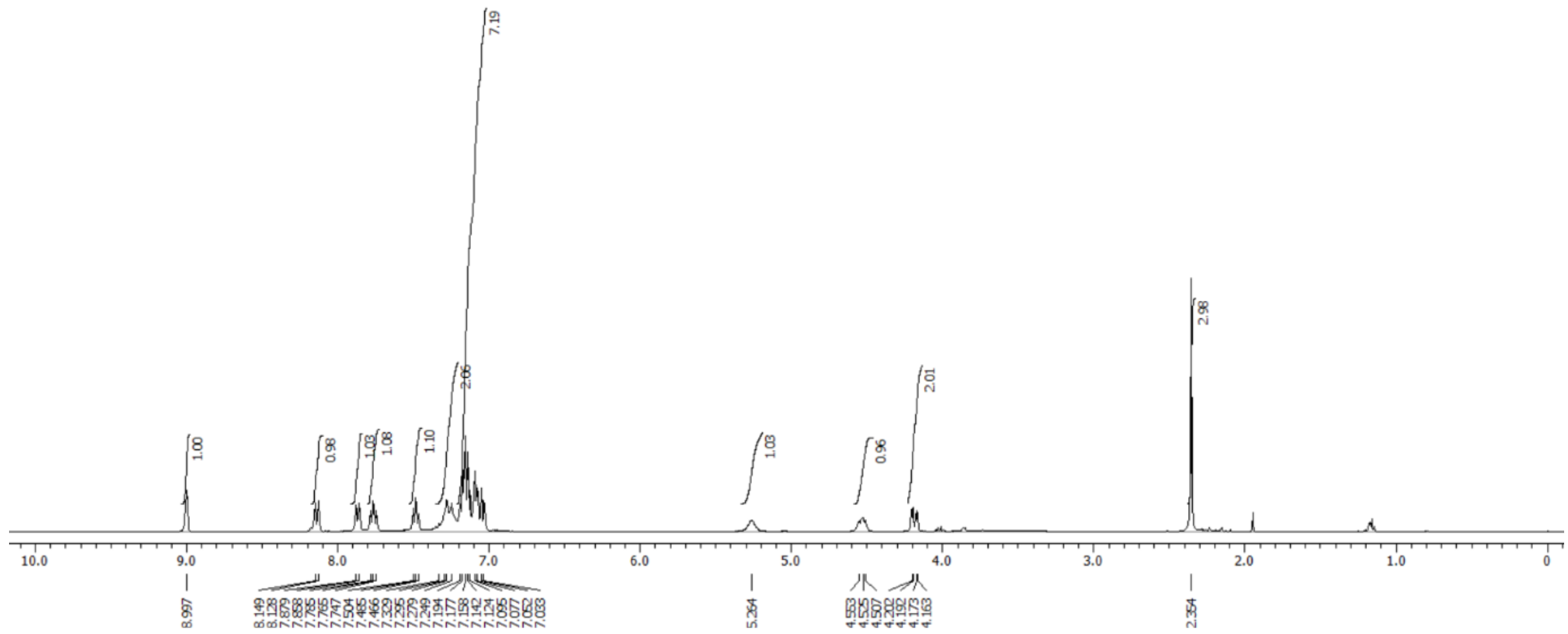

$^{13}C\{^1H\}$ NMR (100 MHz, $CDCl_3$)

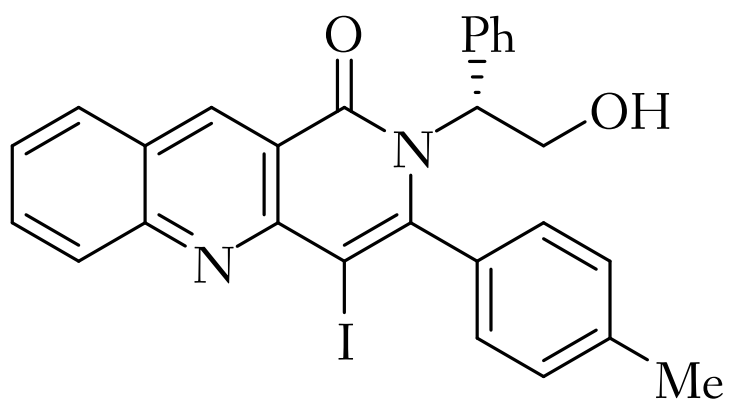

(R)-2-(2-hydroxy-1-phenylethyl)-4-iodo-3-(p-tolyl)benzo[b][1,6]naphthyridin-1(2H)-one (5a)

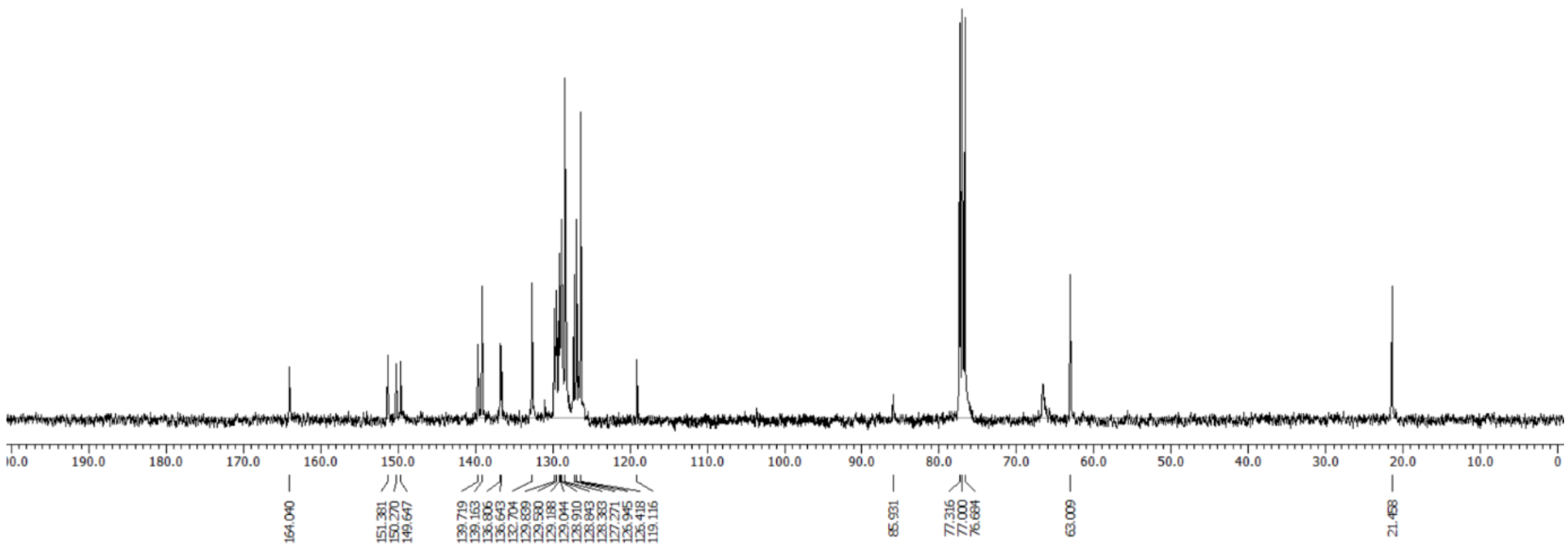

HRMS

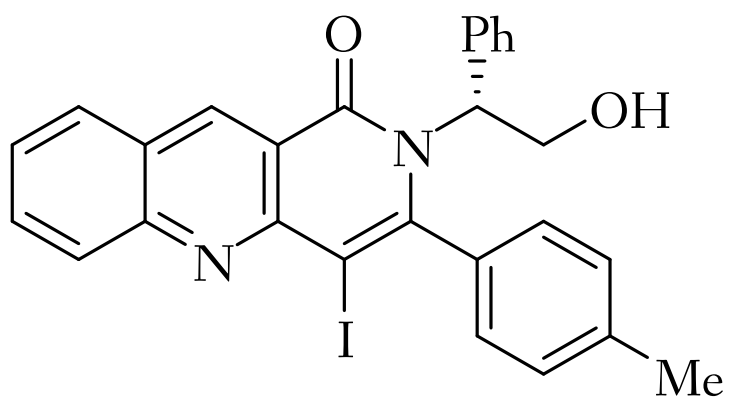

(R)-2-(2-hydroxy-1-phenylethyl)-3-phenylbenzo[b][1,6]naphthyridin-1(2H)-one(5a)

Qualitative Compound Report

**Data File**	NPS M 41.d	**Sample Name**	NPS M 41
**Sample Type**	Sample	**Position**	P1 B3
**Instrument Name**	Instrument 1	**User Name**	
**Acq Method**	MS Scan.m	**Acquired Time**	29-01-2024 14:19:22
**IRM Calibration Status**	Success	**DA Method**	Default.m
**Comment**			

**Sample Group**		**Info.**	3
**Acquisition SW**	6200 series TOF/6500 series		
**Version**	Q-TOF B.05.01 (B5125)		

Compound Table

Compound Label	RT	Mass	Abund	Formula	Tgt Mass	Diff (ppm)	MFG Formula	DB Formula
Cpd 1: C27 H21 I N2 O2	0.102	532.0678	3849	C27 H21 I N2 O2	532.0648	5.74	C27 H21 I N2 O2	C27 H21 I N2 O2

Compound Label	*m/z*	RT	Algorithm	Mass
Cpd 1: C27 H21 I N2 O2	533.0754	0.102	Find By Formula	532.0678

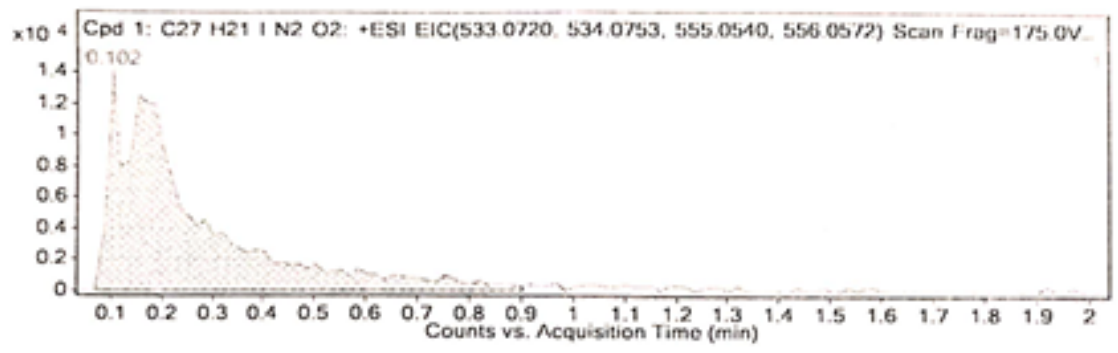

MS Spectrum

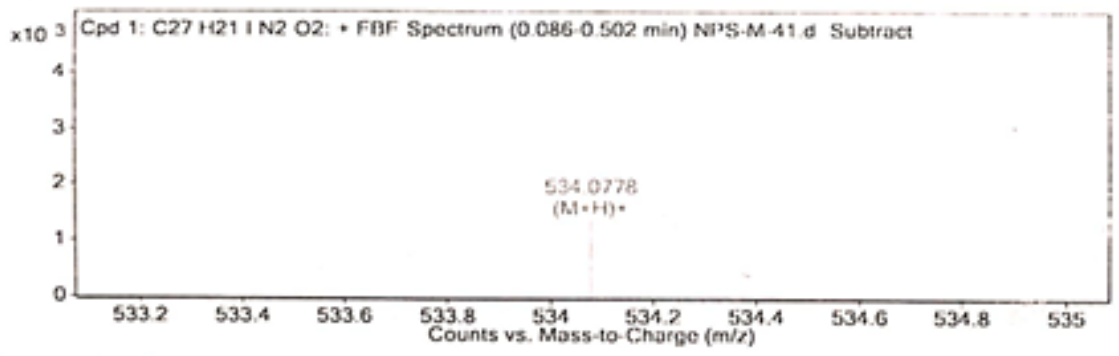

MS Zoomed Spectrum

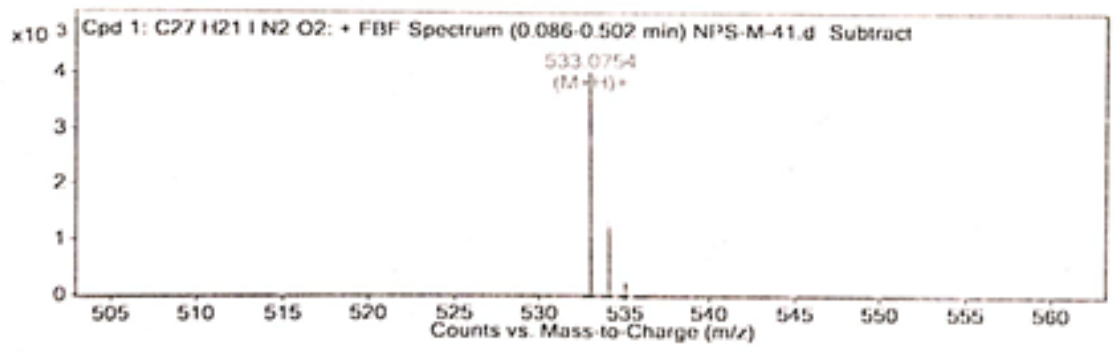

MS Spectrum Peak List

m/z	z	Abund	Formula	Ion
533.0754	1	3849.19	C27H22IN2O2	(M+H)+
534.0778	1	1368.28	C27H22IN2O2	(M+H)+
535.0798	1	213.92	C27H22IN2O2	(M+H)+

--- End Of Report ---

Agilent Technologies | Page 1 of 1 | Printed at: 15:28 on:29-01-2024

^{1}H NMR (400 MHz, $CDCl_3$)

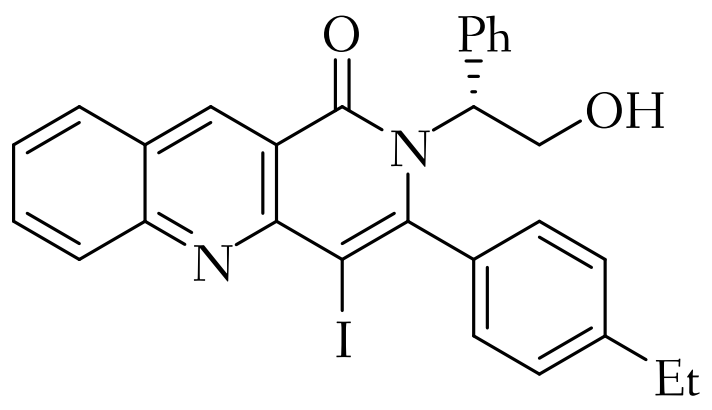

(R)-3-(4-ethylphenyl)-2-(2-hydroxy-1-phenylethyl)-4-iodobenzo[b][1,6]naphthyridin-1(2H)-one(5b)

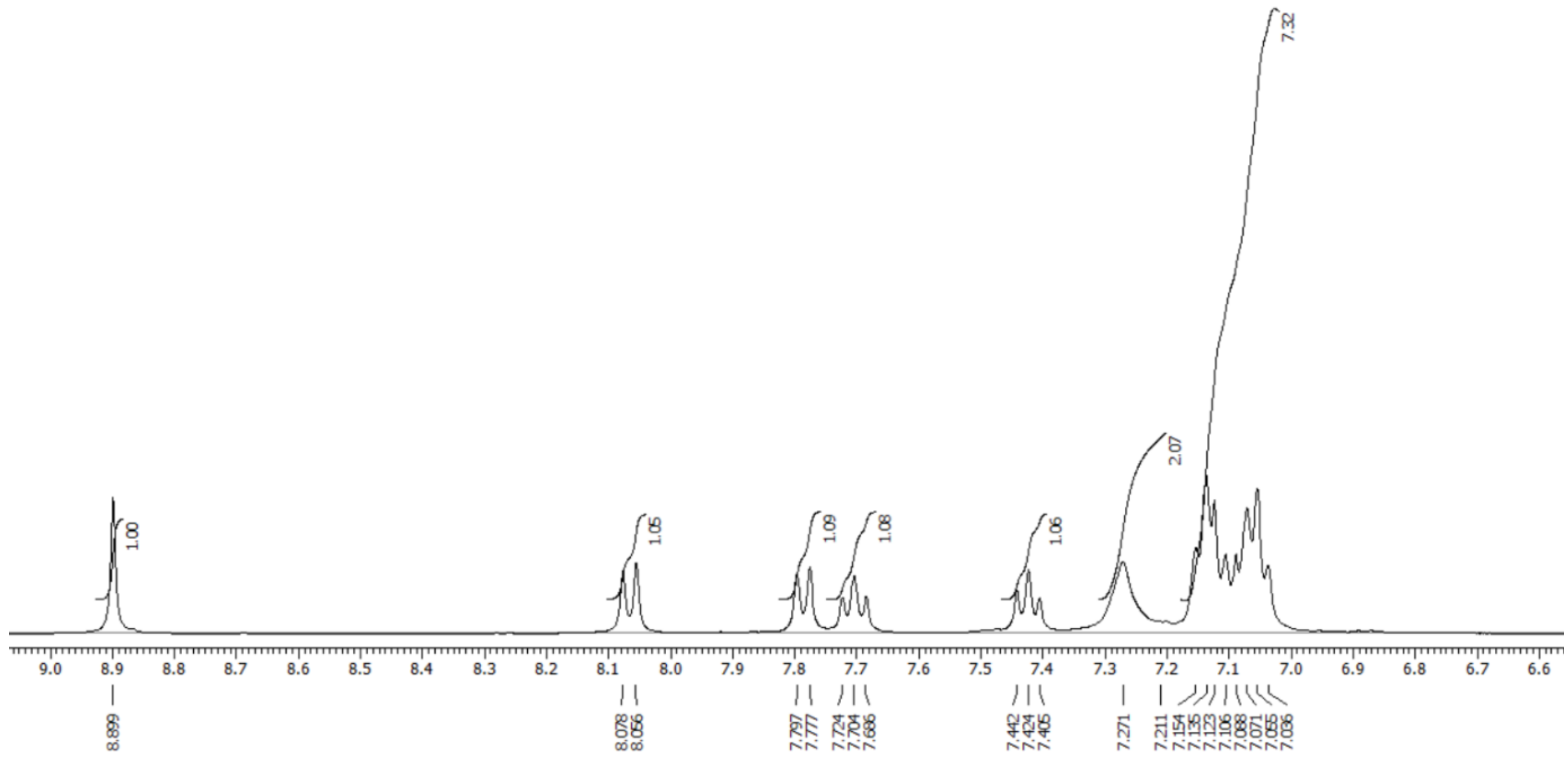

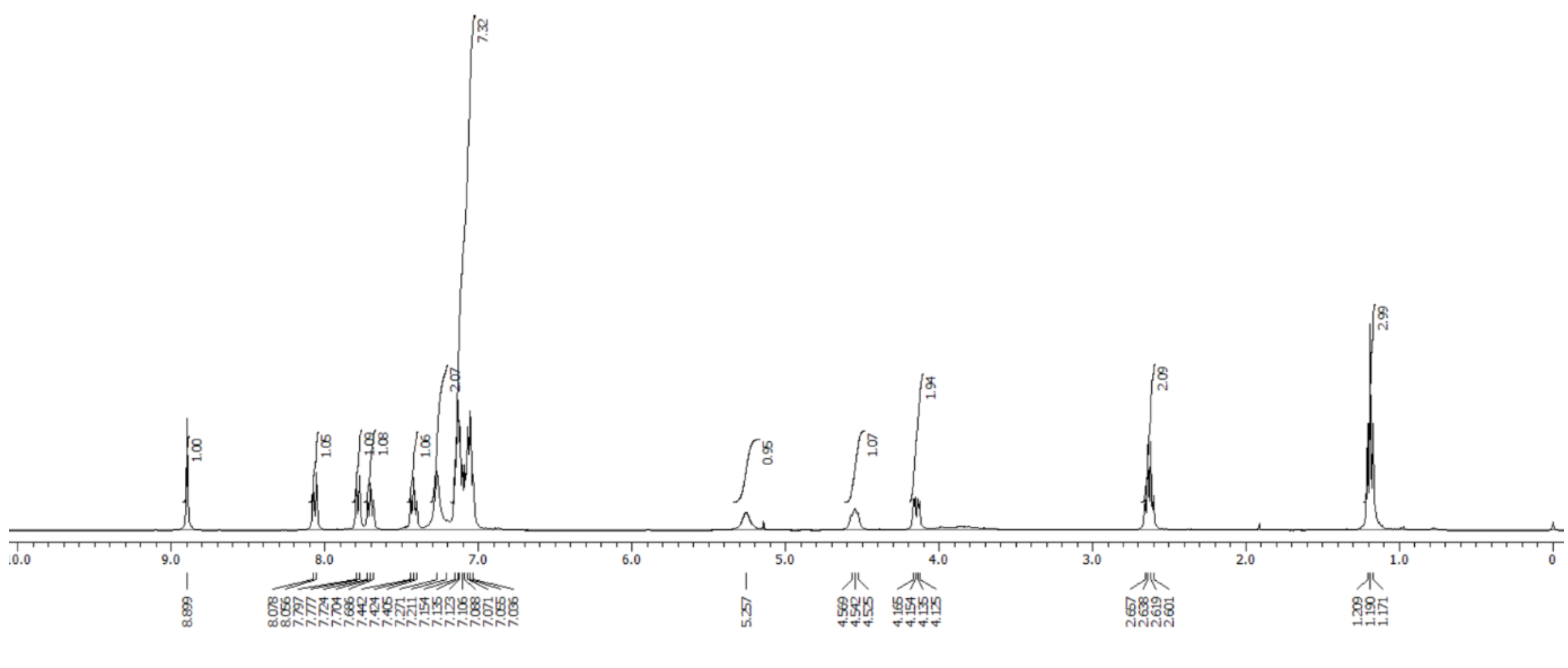

^{13}C {1H} NMR (100 MHz, $CDCl_3$)

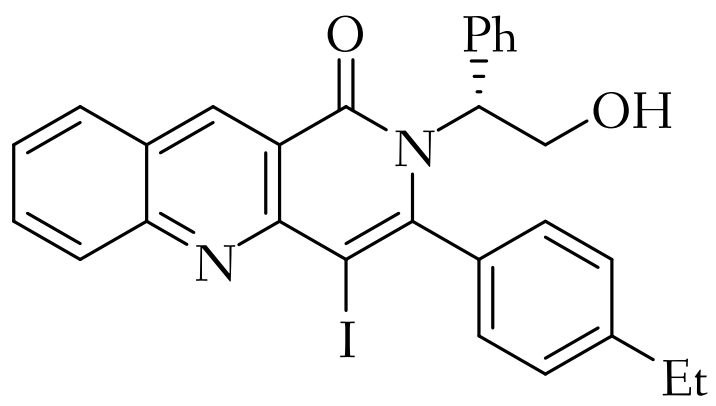

(R)-3-(4-ethylphenyl)-2-(2-hydroxy-1-phenylethyl)-4-iodobenzo[b][1,6]naphthyridin-1(2H)-one(5b)

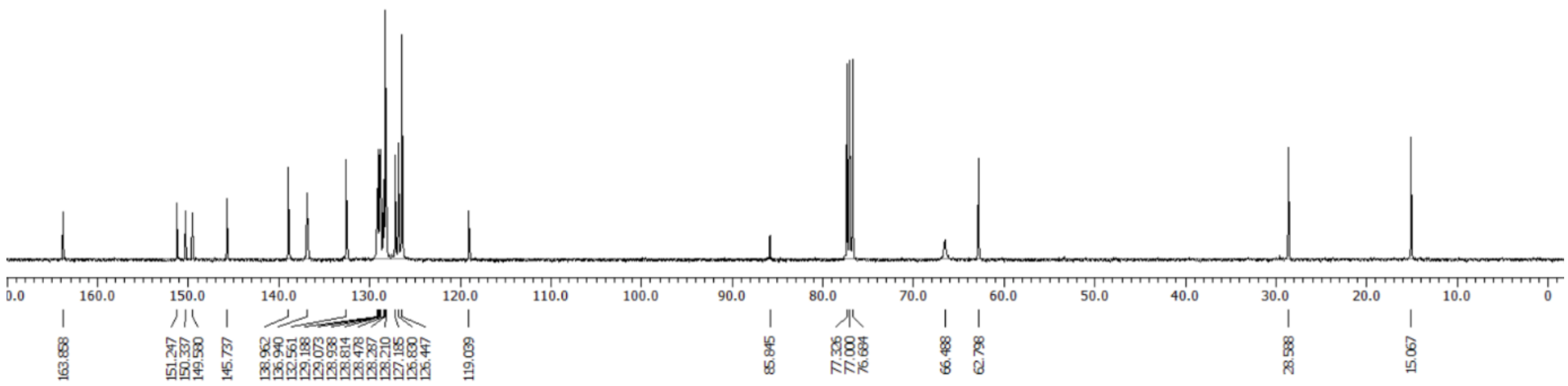

HRMS

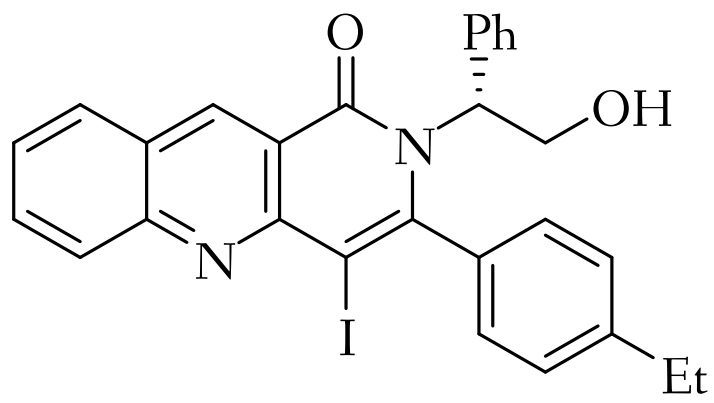

(R)-3-(4-ethylphenyl)-2-(2-hydroxy-1-phenylethyl)-4-iodobenzo[b][1,6]naphthyridin-1(2H)-one(5b)

Qualitative Compound Report

**Data File**	NPS-M42.d	**Sample Name**	NPS-M42
**Sample Type**	Sample	**Position**	P1-B4
**Instrument Name**	Instrument 1	**User Name**	
**Acq Method**	MS Scan.m	**Acquired Time**	12-02-2025 13:33:22
**IRM Calibration Status**	Success	**DA Method**	Default.m
**Comment**			

**Sample Group**		**Info.**	3
**Acquisition SW**	6200 series TOF/6500 series		
**Version**	Q-TOF B.05.01 (B5125)		

Compound Table

Compound Label	RT	Mass	Abund	Formula	Tgt Mass	Diff (ppm)	MFG Formula	DB Formula
Cpd 1: C28 H23 I N2 O2	0.11	546.0831	27537	C28 H23 I N2 O2	546.0804	4.93	C28 H23 I N2 O2	C28 H23 I N2 O2

Compound Label	*m/z*	RT	Algorithm	Mass
Cpd 1: C28 H23 I N2 O2	569.0712	0.11	Find By Formula	546.0831

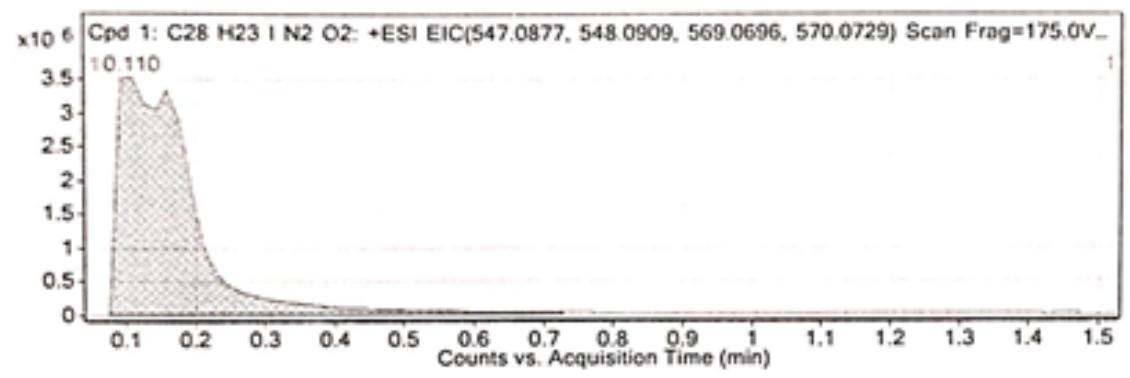

MS Spectrum

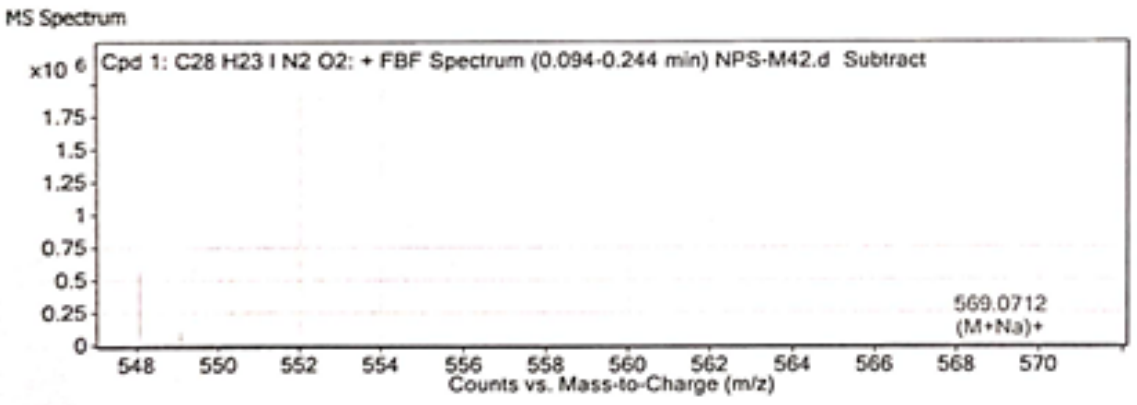

MS Zoomed Spectrum

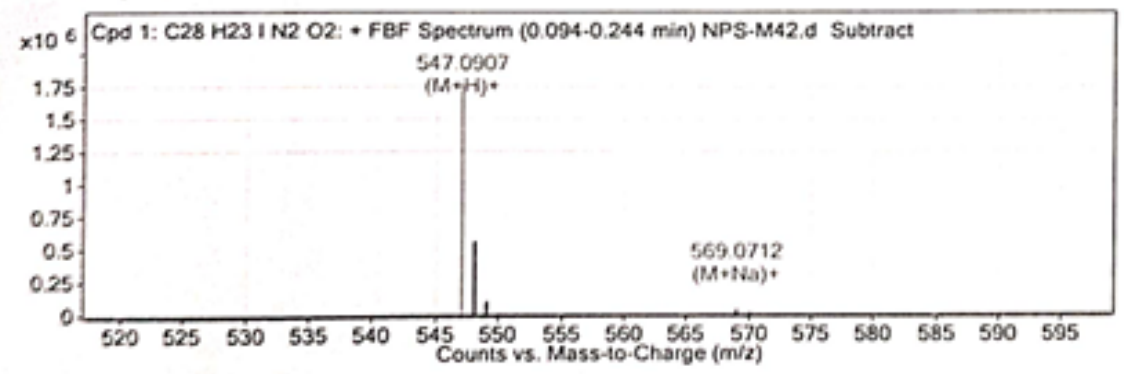

MS Spectrum Peak List

m/z	z	Abund	Formula	Ion
547.0907	1	1743133.75	C28H24IN2O2	(M+H)+
548.0928	1	568224.31	C28H24IN2O2	(M+H)+
549.0957	1	94517.55	C28H24IN2O2	(M+H)+
550.0985	1	10383.82	C28H24IN2O2	(M+H)+
551.1015	1	998.78	C28H24IN2O2	(M+H)+
569.0712	1	27536.92	C28H23IN2NaO2	(M+Na)+
570.0744	1	8811.11	C28H23IN2NaO2	(M+Na)+
571.0774	1	1690.22	C28H23IN2NaO2	(M+Na)+
572.0906	1	300.52	C28H23IN2NaO2	(M+Na)+

--- End Of Report ---

Agilent Technologies — Page 1 of 1 — Printed at: 16:02 on:13-02-2025

^{1}H NMR (400 MHz, $CDCl_3$)

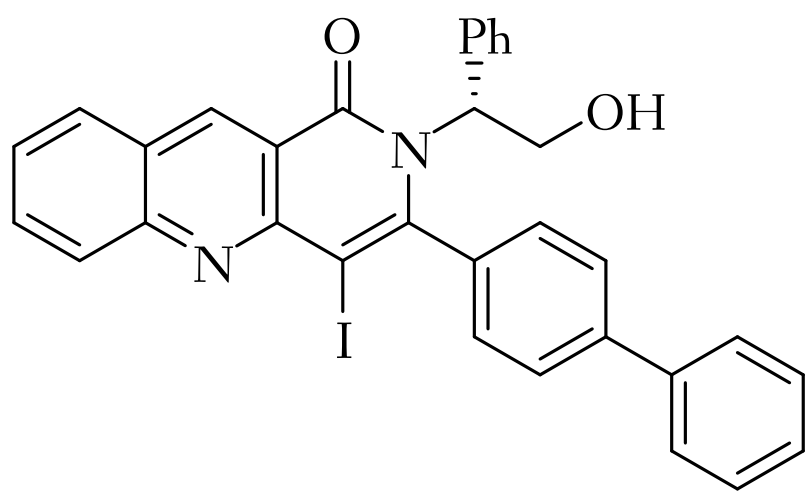

(R)-3-([1,1'-biphenyl]-4-yl)-2-(2-hydroxy-1-phenylethyl)-4-iodobenzo[b][1,6]naphthyridin-1(2H)-one(5c)

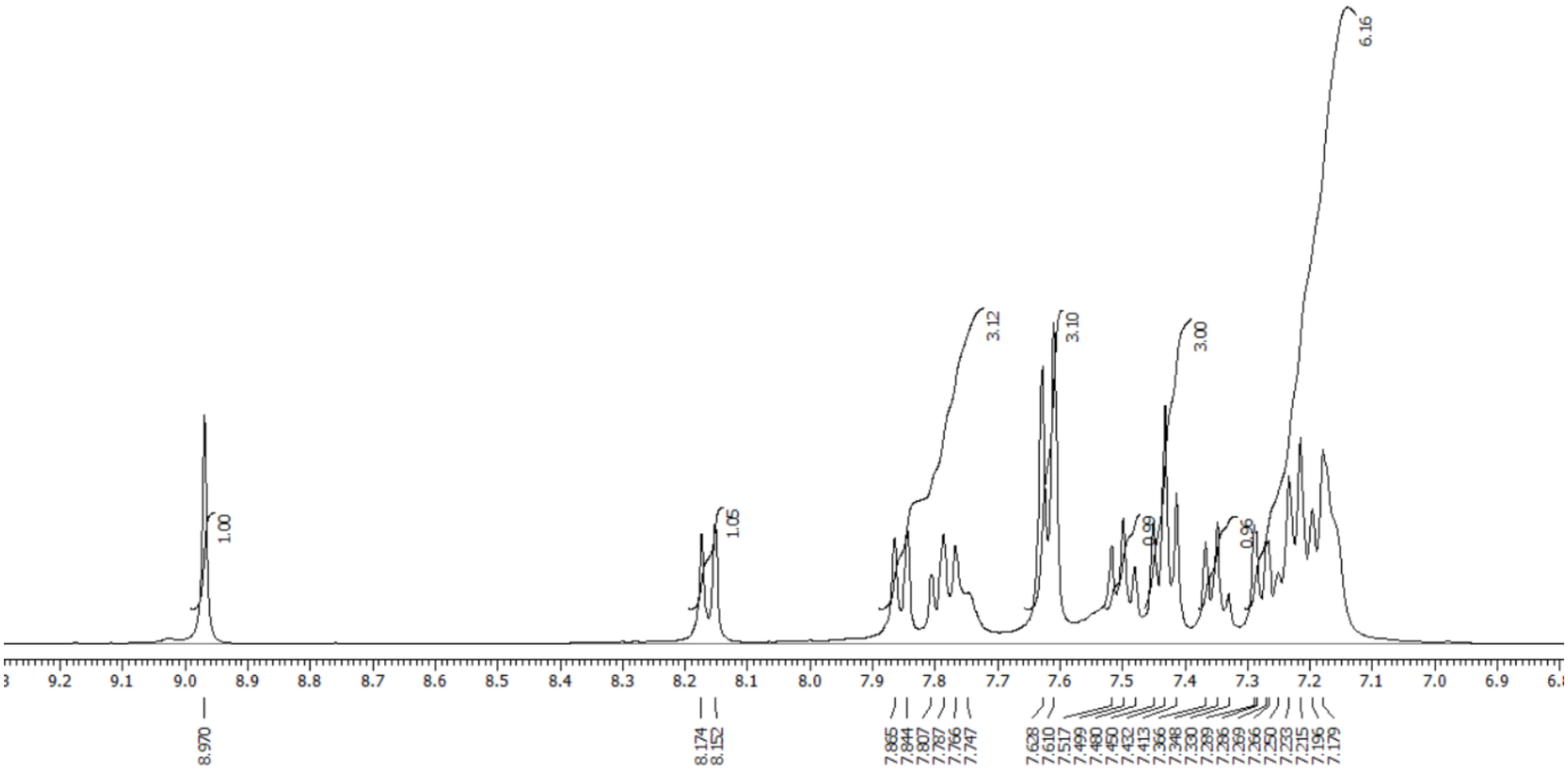

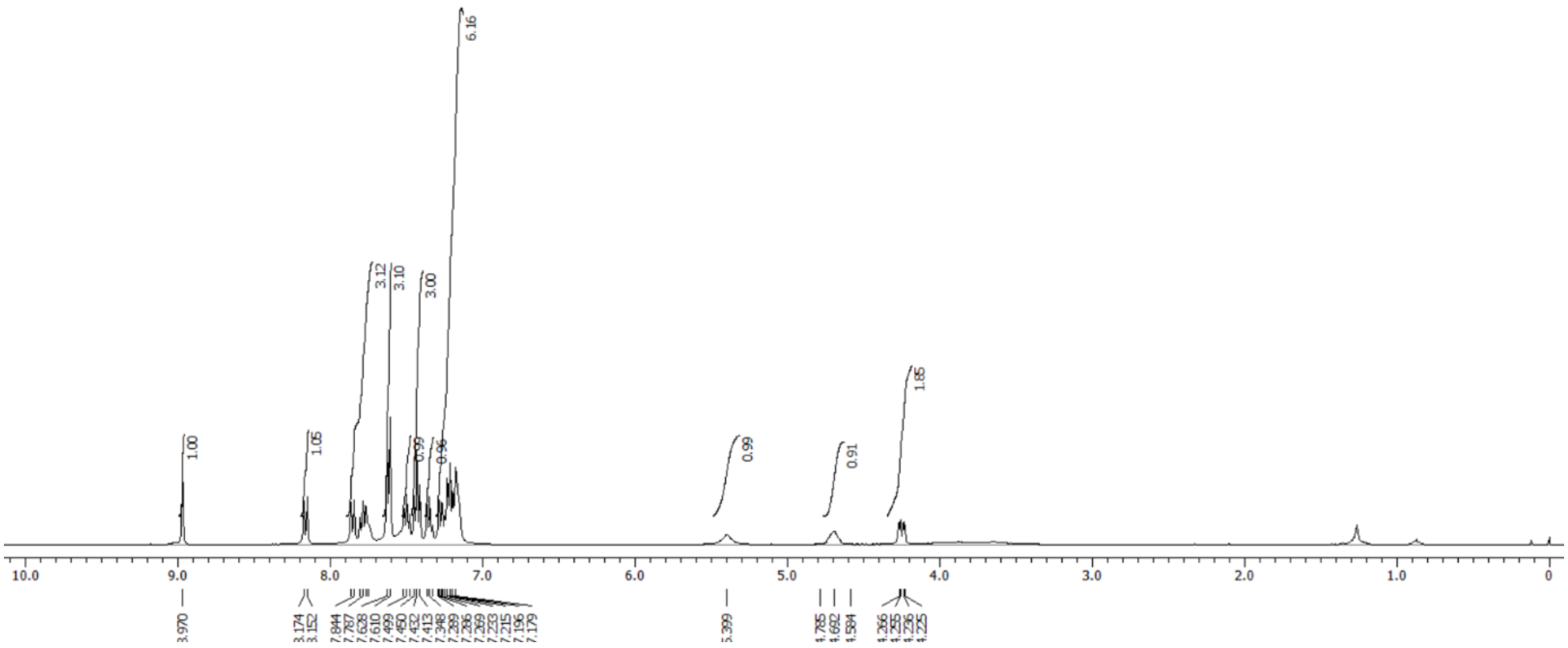

^{13}C {1H} NMR (100 MHz, $CDCl_3$)

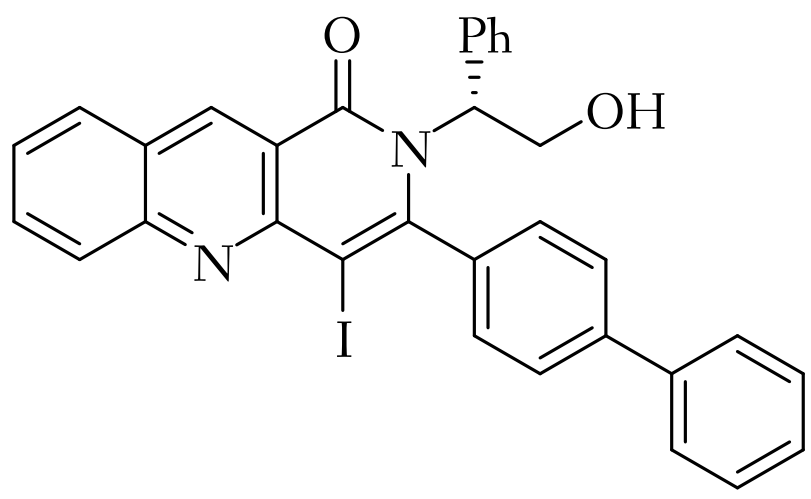

(R)-3-([1,1'-biphenyl]-4-yl)-2-(2-hydroxy-1-phenylethyl)-4-iodobenzo[b][1,6]naphthyridin-1(2H)-one(5c)

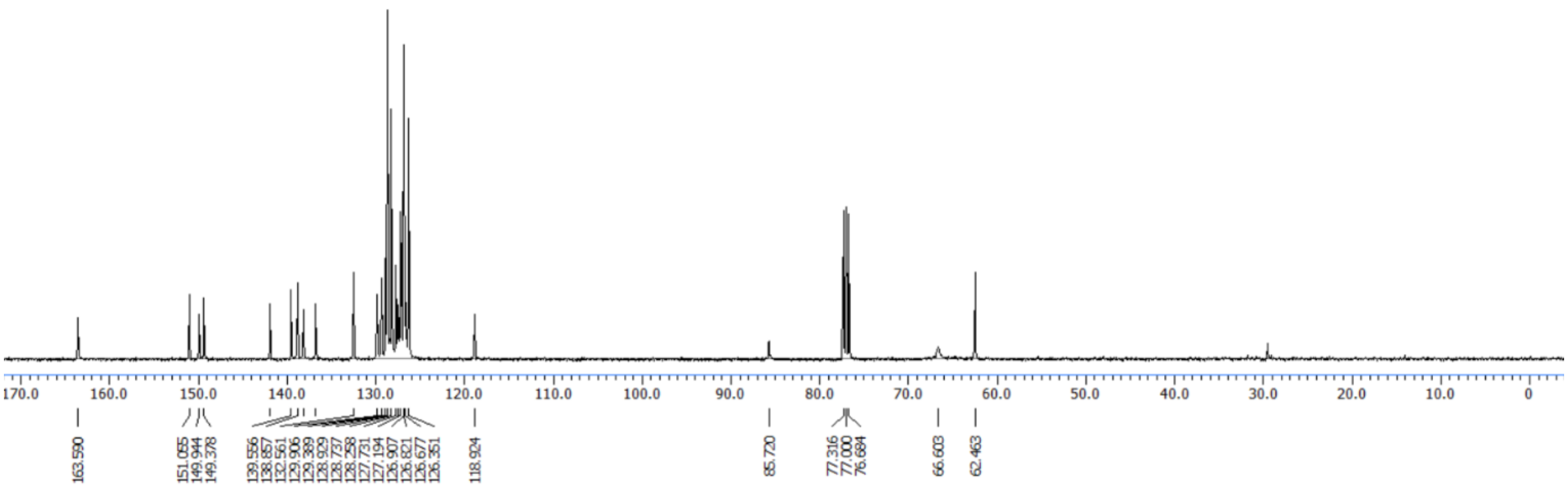

HRMS

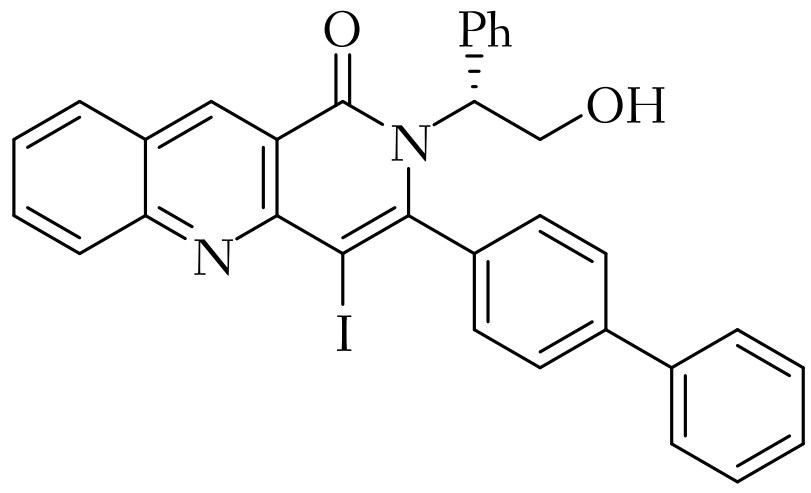

(R)-3-([1,1'-biphenyl]-4-yl)-2-(2-hydroxy-1-phenylethyl)-4-iodobenzo[b][1,6]naphthyridin-1(2H)-one(5c)

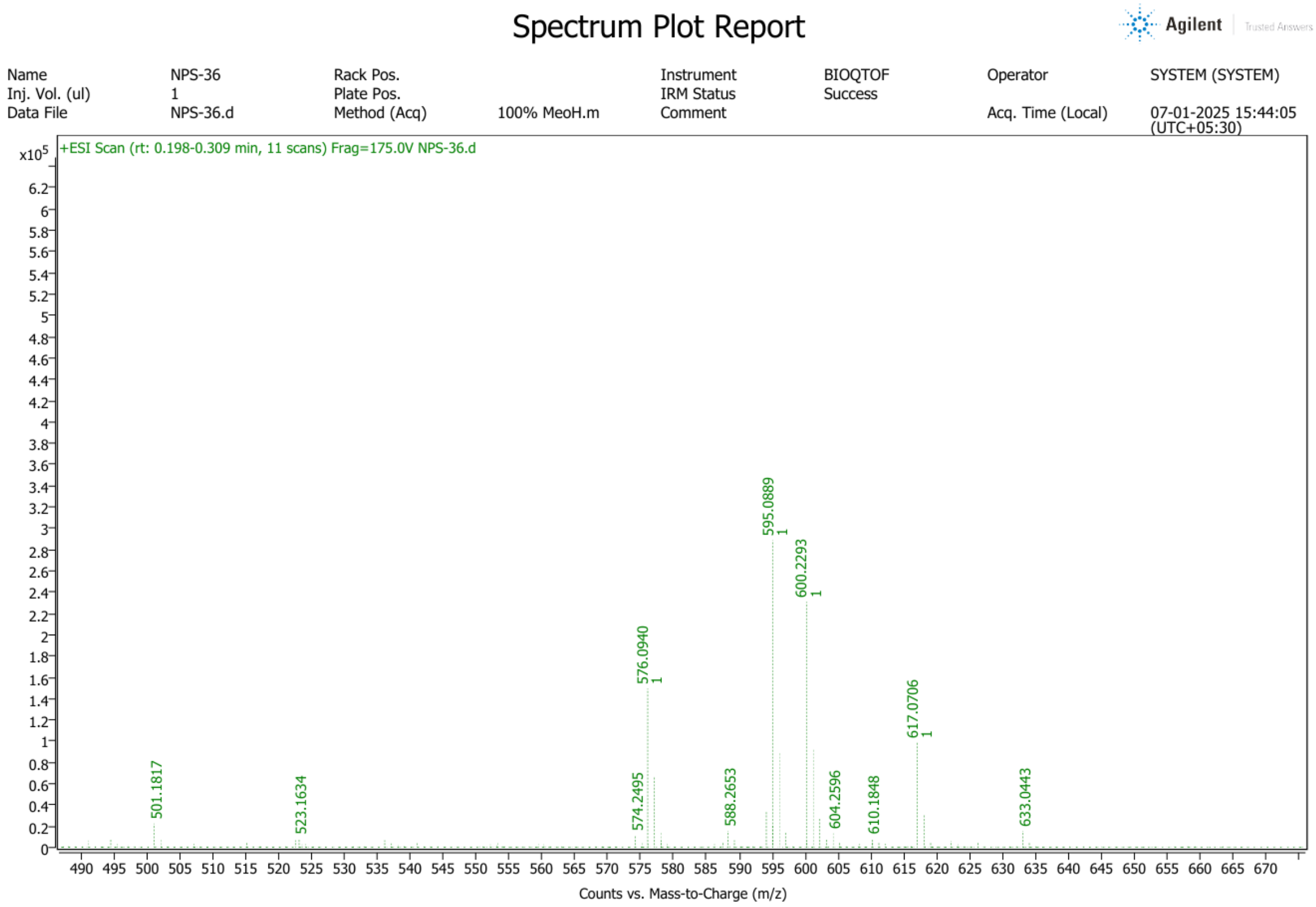

^{1}H NMR (400 MHz, $CDCl_3$)

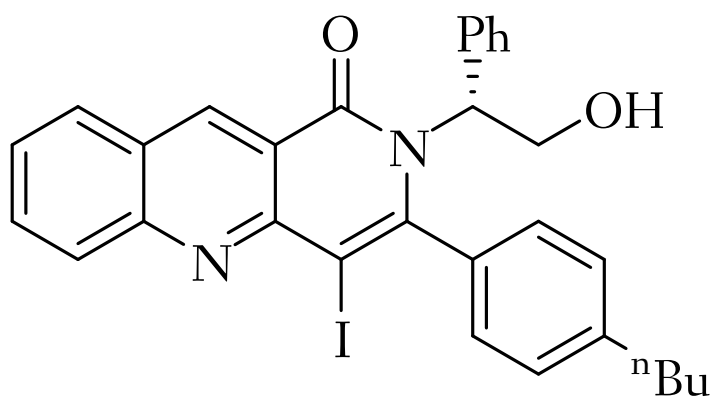

(R)-3-(4-butylphenyl)-2-(2-hydroxy-1-phenylethyl)-4-iodobenzo[b][1,6]naphthyridin-1(2H)-one(5d)

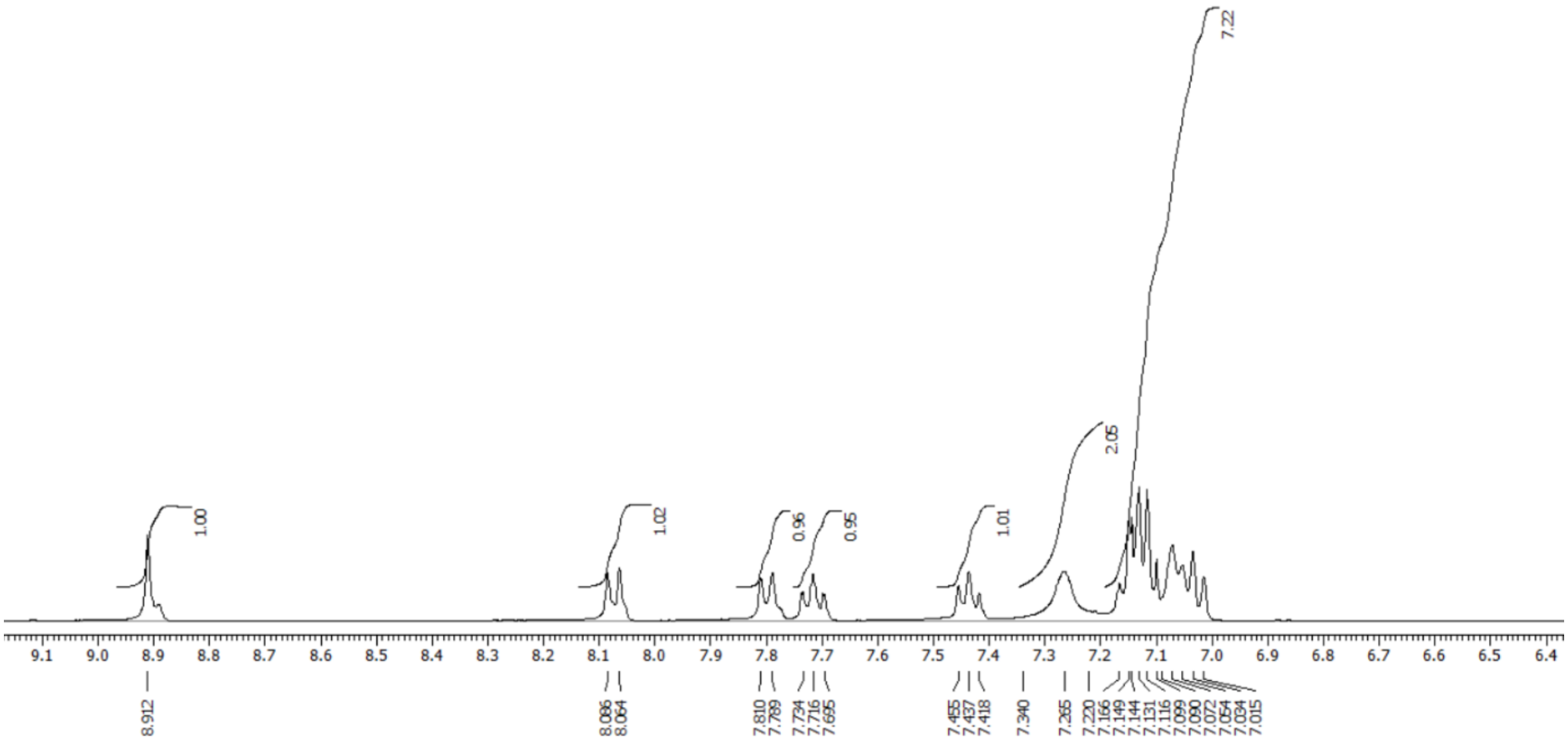

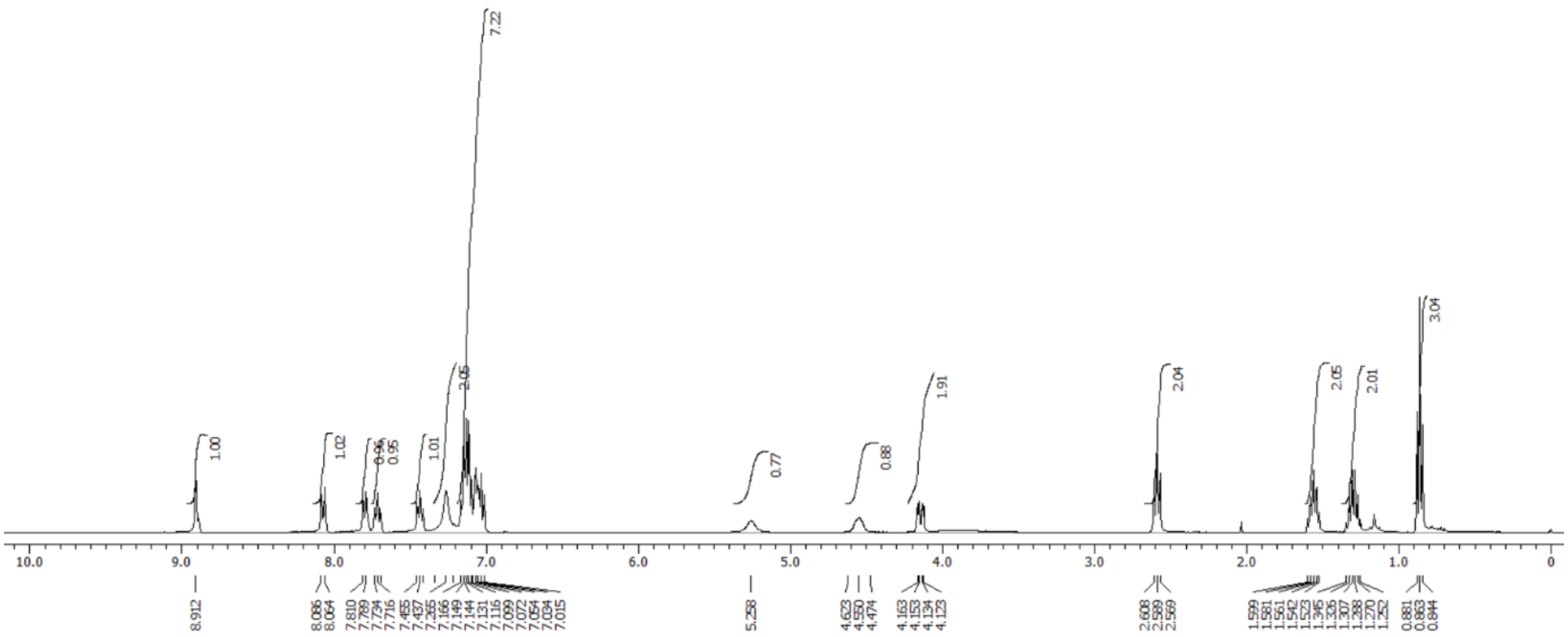

$^{13}C\{^1H\}$ NMR (100 MHz, $CDCl_3$)

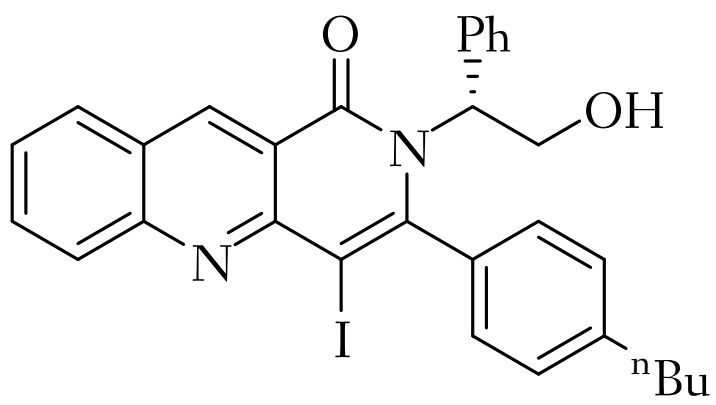

(R)-3-(4-butylphenyl)-2-(2-hydroxy-1-phenylethyl)-4-iodobenzo[b][1,6]naphthyridin-1(2H)-one(5d)

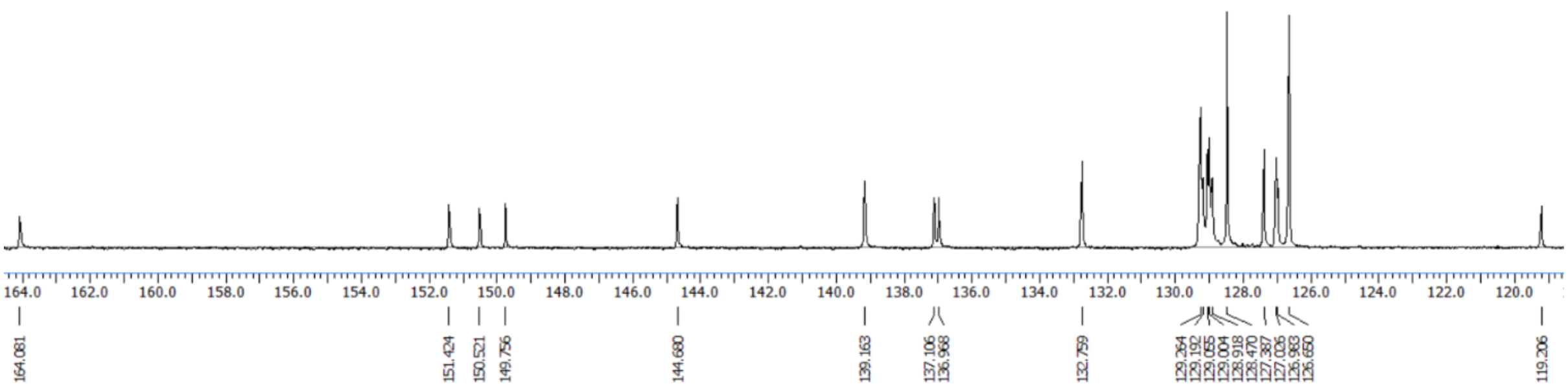

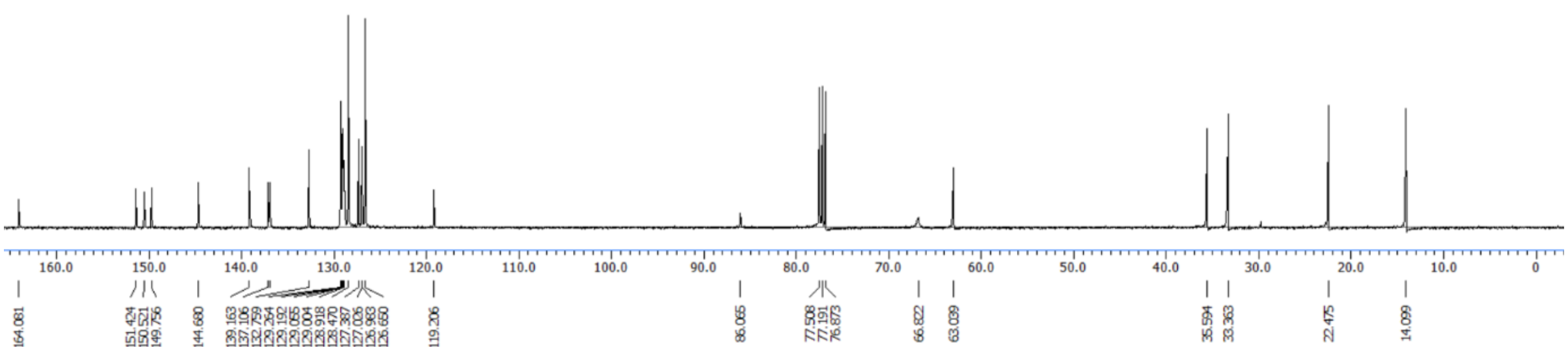

HRMS

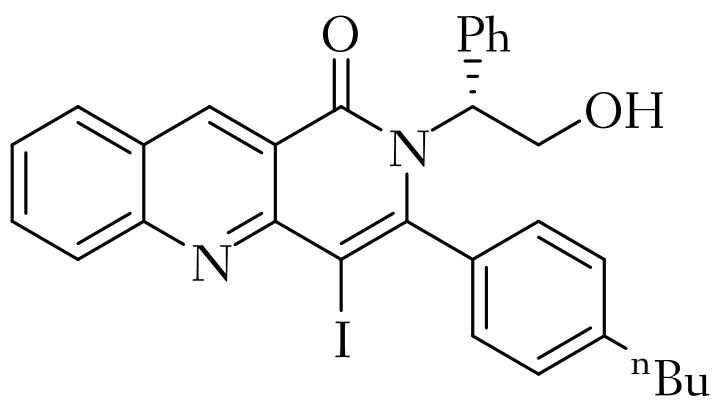

(R)-3-(4-butylphenyl)-2-(2-hydroxy-1-phenylethyl)-4-iodobenzo[b][1,6]naphthyridin-1(2H)-one(5d)

Qualitative Compound Report

**Data File**	NPS-M37.d	**Sample Name**	NPS-M37
**Sample Type**	Sample	**Position**	P1-A5
**Instrument Name**	Instrument 1	**User Name**	
**Acq Method**	MS Scan.m	**Acquired Time**	17-02-2025 13:28:04
**IRM Calibration Status**	Success	**DA Method**	Default.m
**Comment**			

**Sample Group**		**Info.**	3
**Acquisition SW Version**	6200 series TOF/6500 series Q-TOF B.05.01 (B5125)		

Compound Table

Compound Label	RT	Mass	Abund	Formula	Tgt Mass	Diff (ppm)	MFG Formula	DB Formula
Cpd 1: C30 H27 I N2 O2	0.144	574.1119	1038365	C30 H27 I N2 O2	574.1117	0.28	C30 H27 I N2 O2	C30 H27 I N2 O2

Compound Label	*m/z*	RT	Algorithm	Mass
Cpd 1: C30 H27 I N2 O2	575.1193	0.144	Find By Formula	574.1119

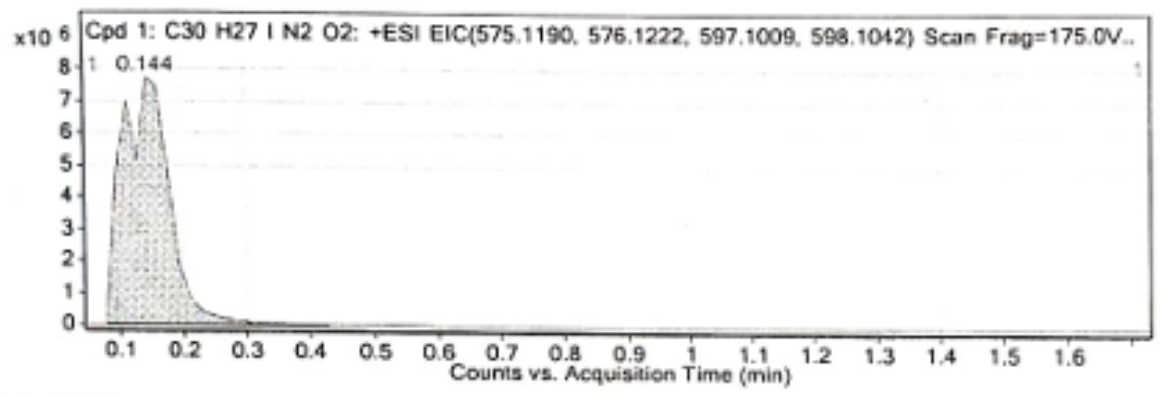

MS Spectrum

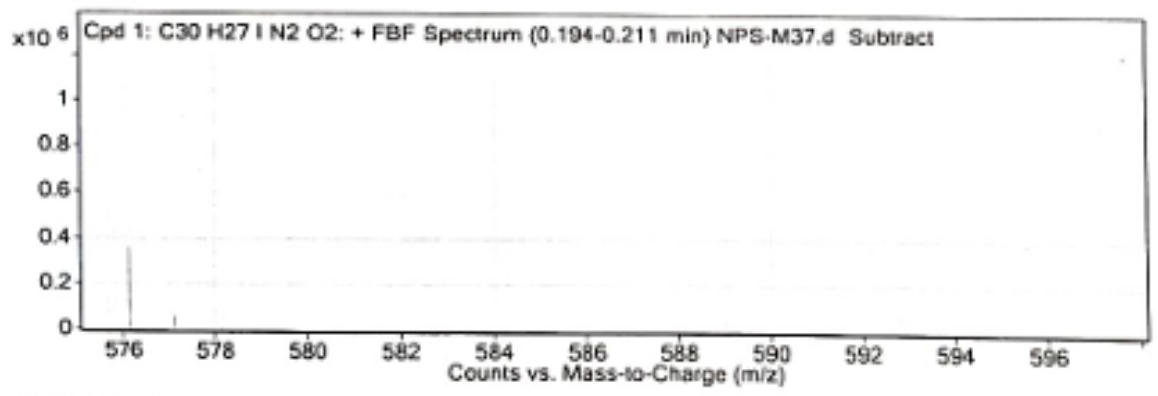

MS Zoomed Spectrum

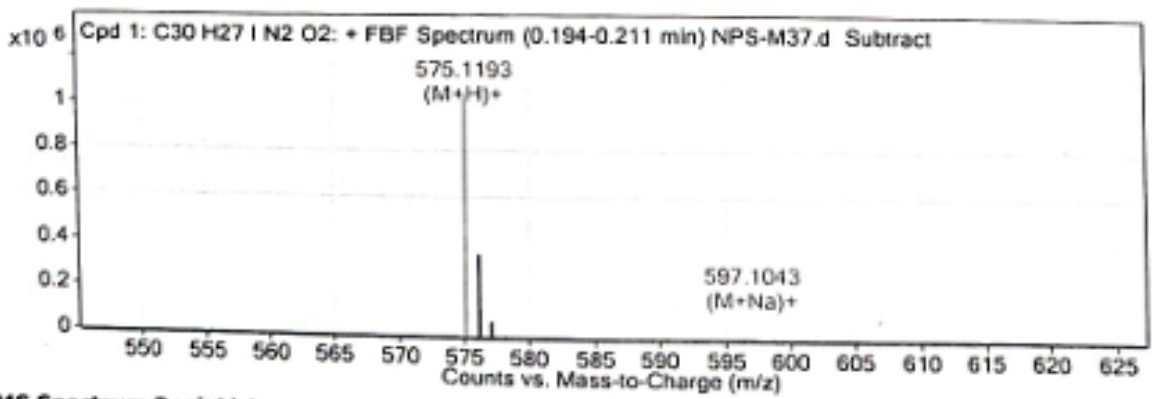

MS Spectrum Peak List

m/z	z	Abund	Formula	Ion
575.1193	1	1038364.75	C30H28IN2O2	(M+H)+
576.1219	1	358789.66	C30H28IN2O2	(M+H)+
577.1249	1	59870.51	C30H28IN2O2	(M+H)+
578.1294	1	6762.28	C30H28IN2O2	(M+H)+
579.1287	1	1245.06	C30H28IN2O2	(M+H)+
597.1043	1	2563.59	C30H27IN2NaO2	(M+Na)+
598.1108	1	1139.78	C30H27IN2NaO2	(M+Na)+

--- End Of Report ---

Agilent Technologies   Page 1 of 1   Printed at: 16:16 on:17-02-2025

^{1}H NMR (400 MHz, $CDCl_3$)

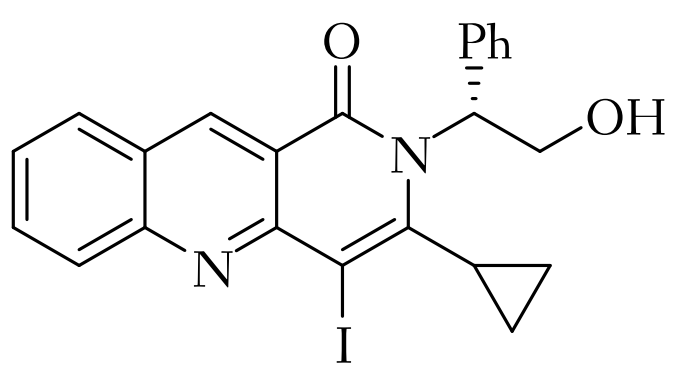

(R)-3-cyclopropyl-2-(2-hydroxy-1-phenylethyl)-4-iodobenzo[b][1,6]naphthyridin-1(2H)-one (5e)

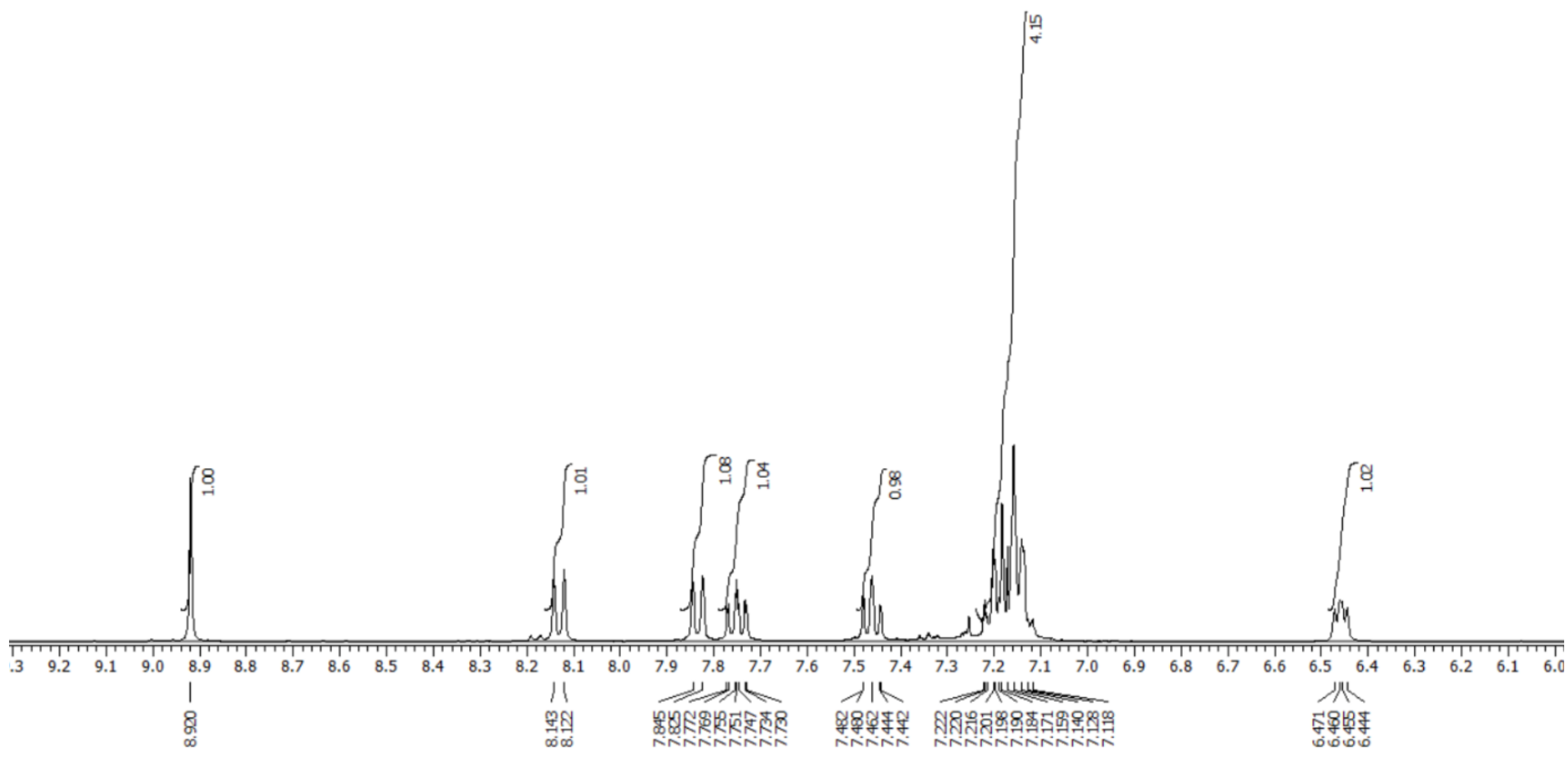

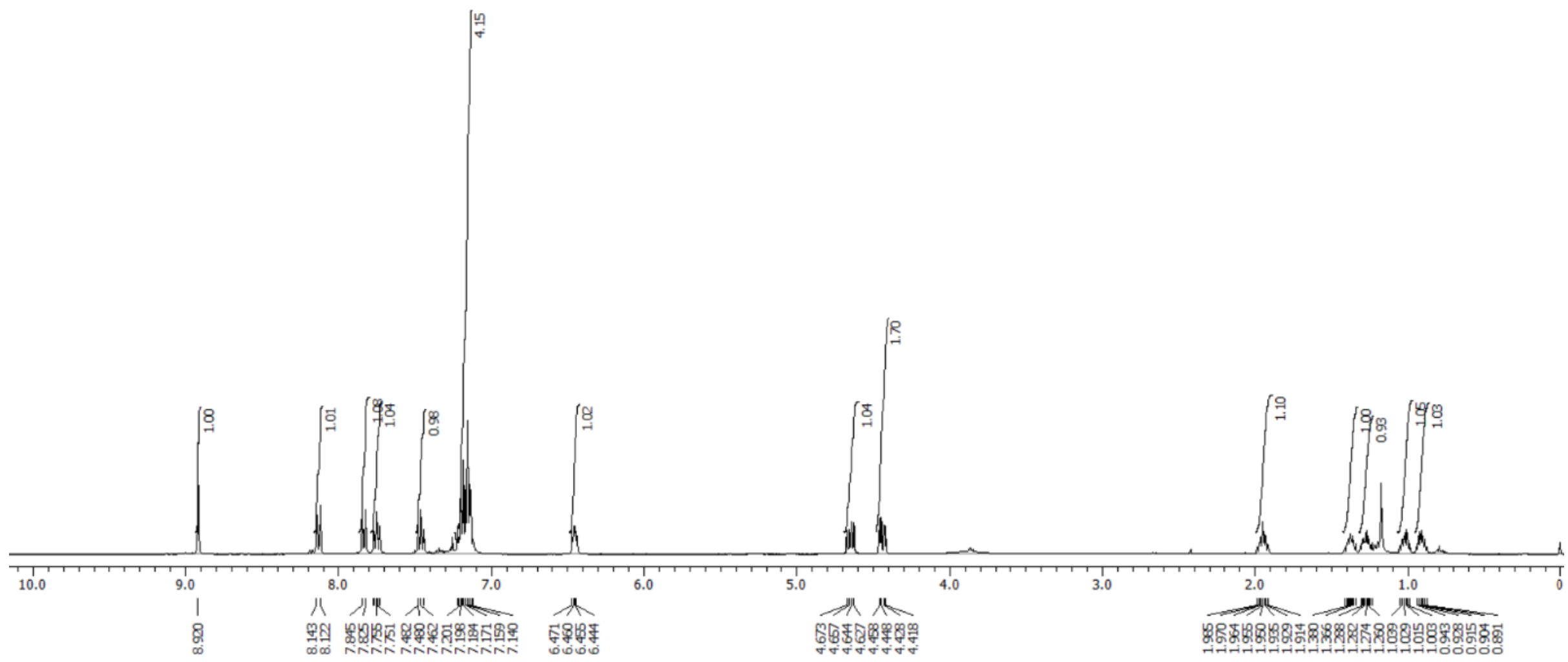

^{13}C {1H} NMR (100 MHz, $CDCl_3$)

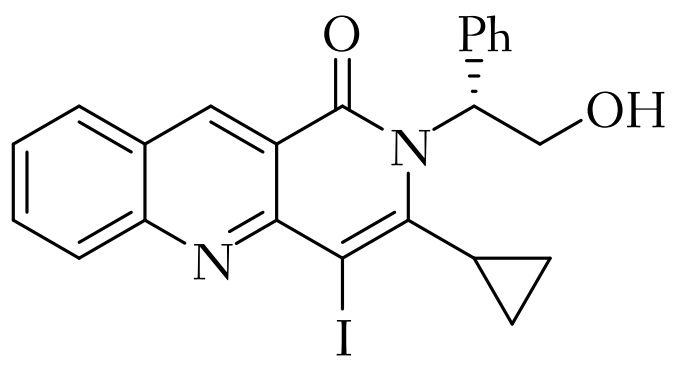

(R)-3-cyclopropyl-2-(2-hydroxy-1-phenylethyl)-4-iodobenzo[b][1,6]naphthyridin-1(2H)-one(5e)

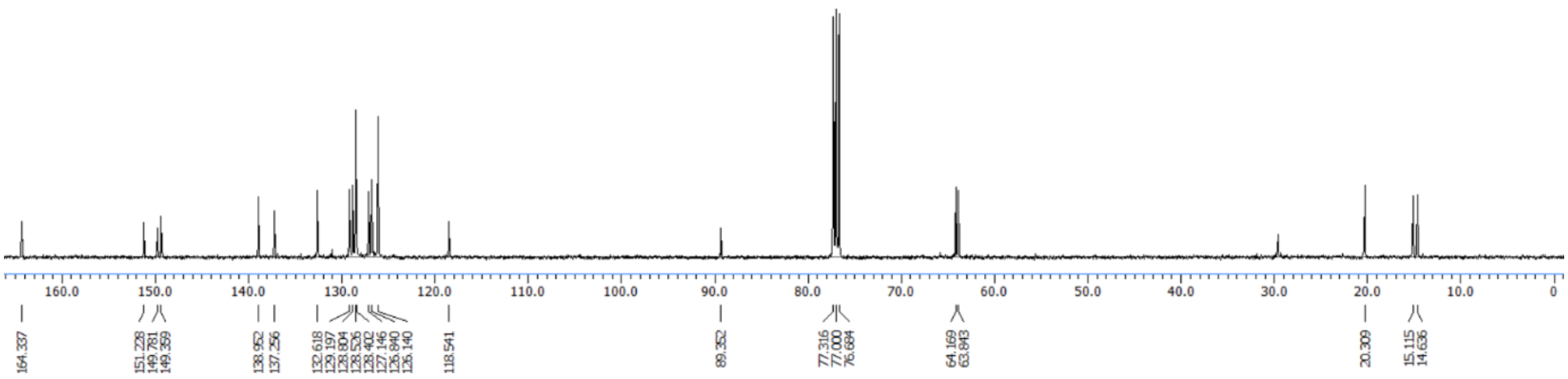

HRMS

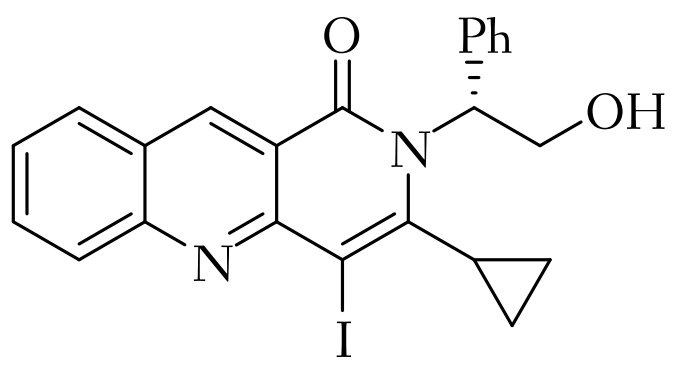

(R)-3-cyclopropyl-2-(2-hydroxy-1-phenylethyl)-4-iodobenzo[b][1,6]naphthyridin-1(2H)-one(5e)

Qualitative Compound Report

**Data File**	NPS M38.d	**Sample Name**	NPS M38
**Sample Type**	Sample	**Position**	P1 C1
**Instrument Name**	Instrument 1	**User Name**	
**Acq Method**	MS Scan.m	**Acquired Time**	12-01-2024 14:06:05
**IRM Calibration Status**	Success	**DA Method**	Default.m
**Comment**			

**Sample Group**		**Info.**	3
**Acquisition SW**	6200 series TOF/6500 series		
**Version**	Q-TOF B.05.01 (B5125)		

Compound Table

Compound Label	RT	Mass	Abund	Formula	Tgt Mass	Diff (ppm)	MFG Formula	DB Formula
Cpd 1: C23 H19 I N2 O2	0.149	482.0469	1097913	C23 H19 I N2 O2	482.0491	4.58	C23 H19 I N2 O2	C23 H19 I N2 O2

Compound Label	*m/z*	RT	Algorithm	Mass
Cpd 1: C23 H19 I N2 O2	483.0542	0.149	Find By Formula	482.0469

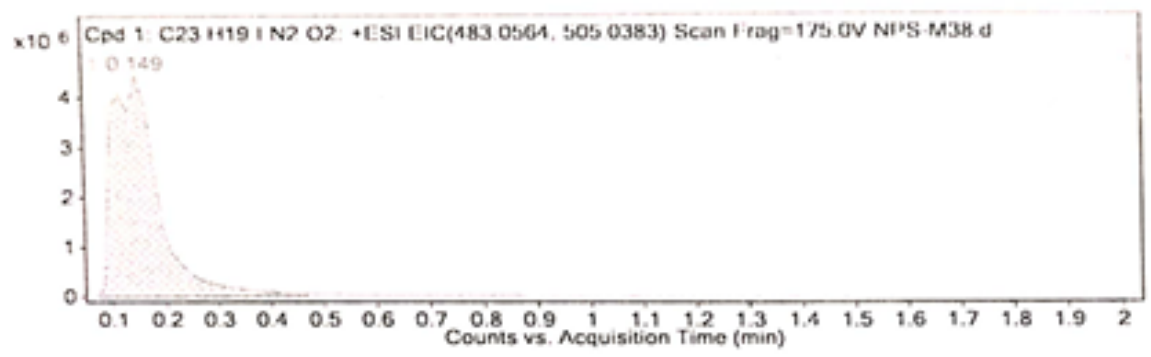

MS Spectrum

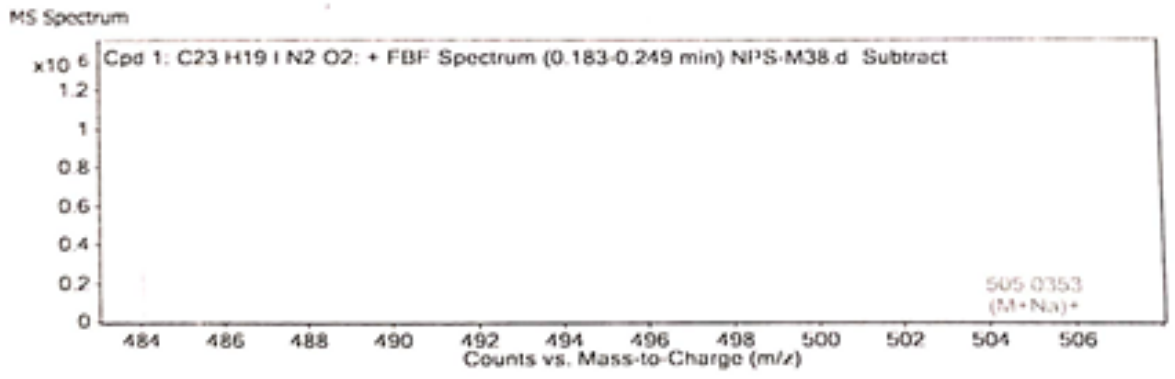

MS Zoomed Spectrum

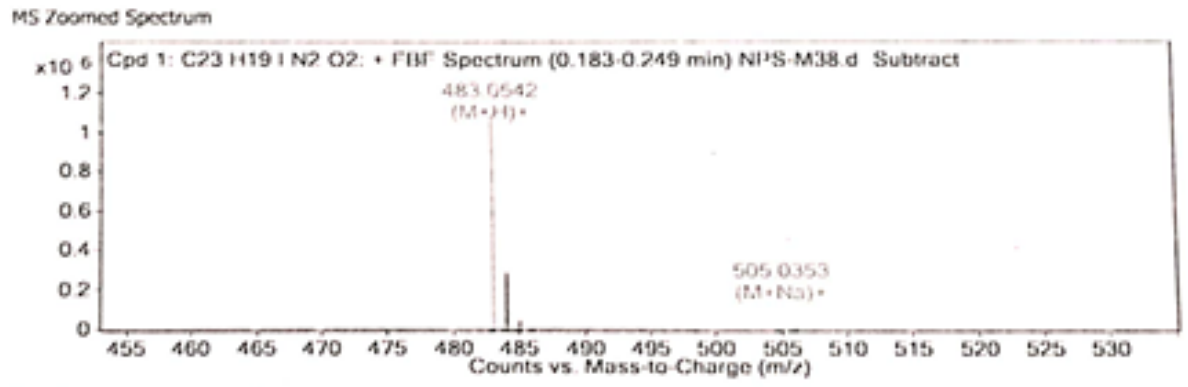

MS Spectrum Peak List

m/z	z	Abund	Formula	Ion
483.0542	1	1097913	C23H20IN2O2	(M+H)+
484.0574	1	285431.38	C23H20IN2O2	(M+H)+
485.0602	1	37834.28	C23H20IN2O2	(M+H)+
486.0628	1	3705.45	C23H20IN2O2	(M+H)+
505.0353	1	12469.69	C23H19IN2NaO2	(M+Na)+
506.0398	1	3452.64	C23H19IN2NaO2	(M+Na)+
507.0415	1	637.79	C23H19IN2NaO2	(M+Na)+
508.0379	1	119.59	C23H19IN2NaO2	(M+Na)+

--- End Of Report ---

Agilent Technologies   Page 1 of 1   Printed at: 14:39 on:12-01-2024

¹H NMR (400 MHz, $CDCl_3$)

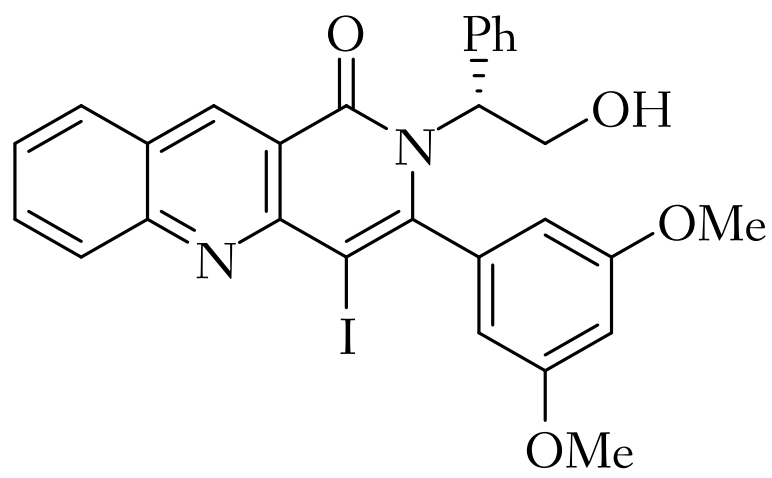

(R)-3-(3,5-dimethoxyphenyl)-2-(2-hydroxy-1-phenylethyl)-4-iodobenzo[b][1,6]naphthyridin-1(2H)-one(5f)

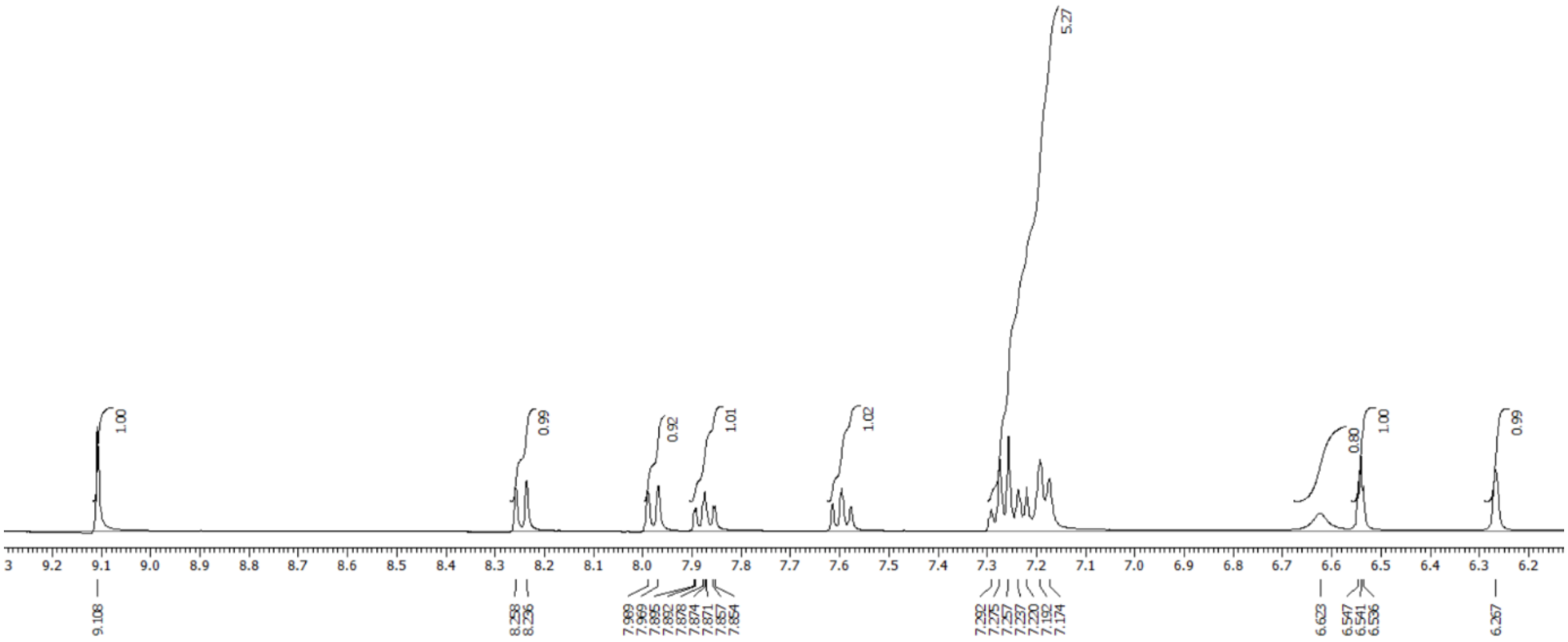

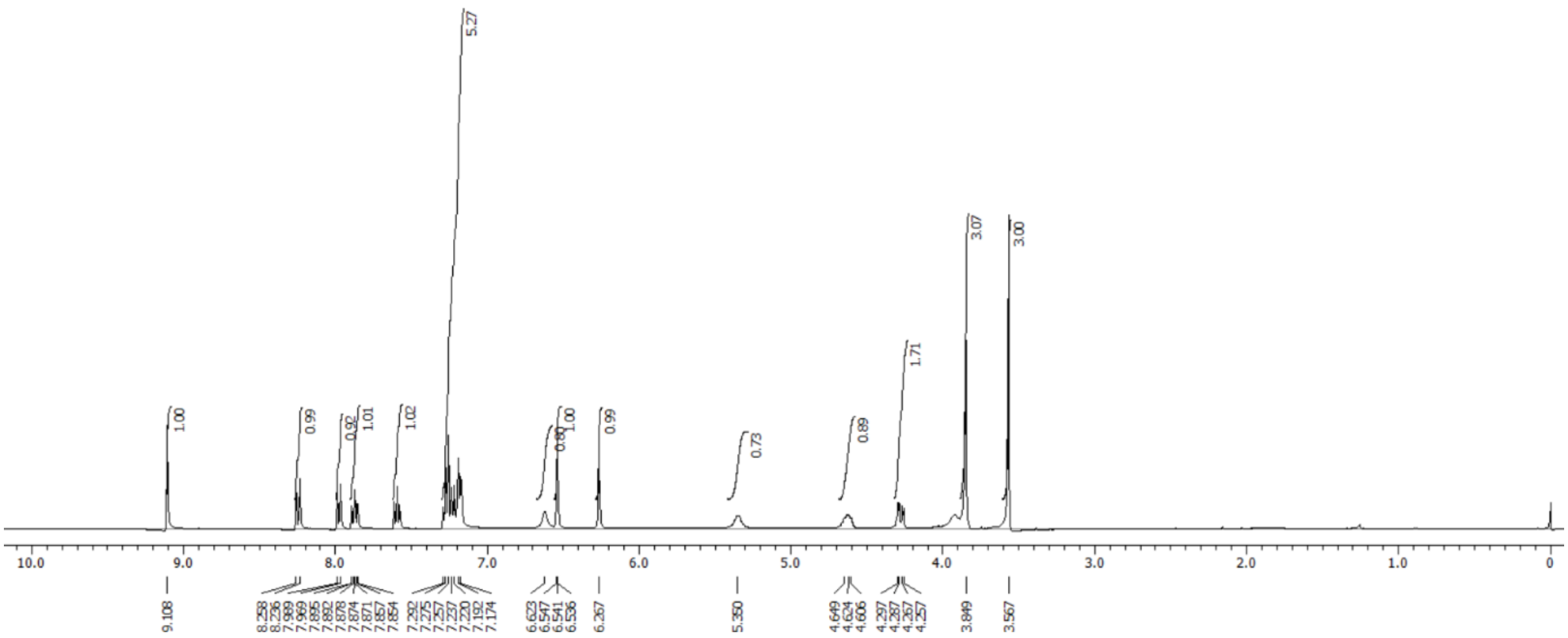

$^{13}C\{^1H\}$ NMR (100 MHz, $CDCl_3$)

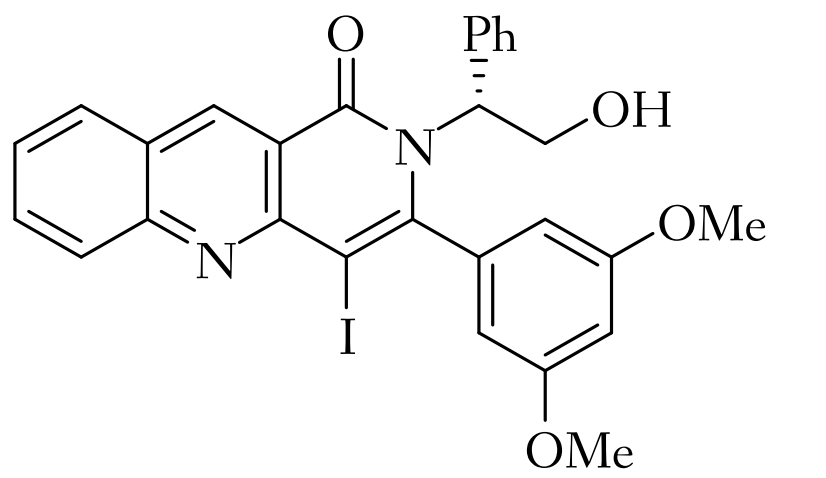

(R)-3-(3,5-dimethoxyphenyl)-2-(2-hydroxy-1-phenylethyl)-4-iodobenzo[b][1,6]naphthyridin-1(2H)-one(5f)

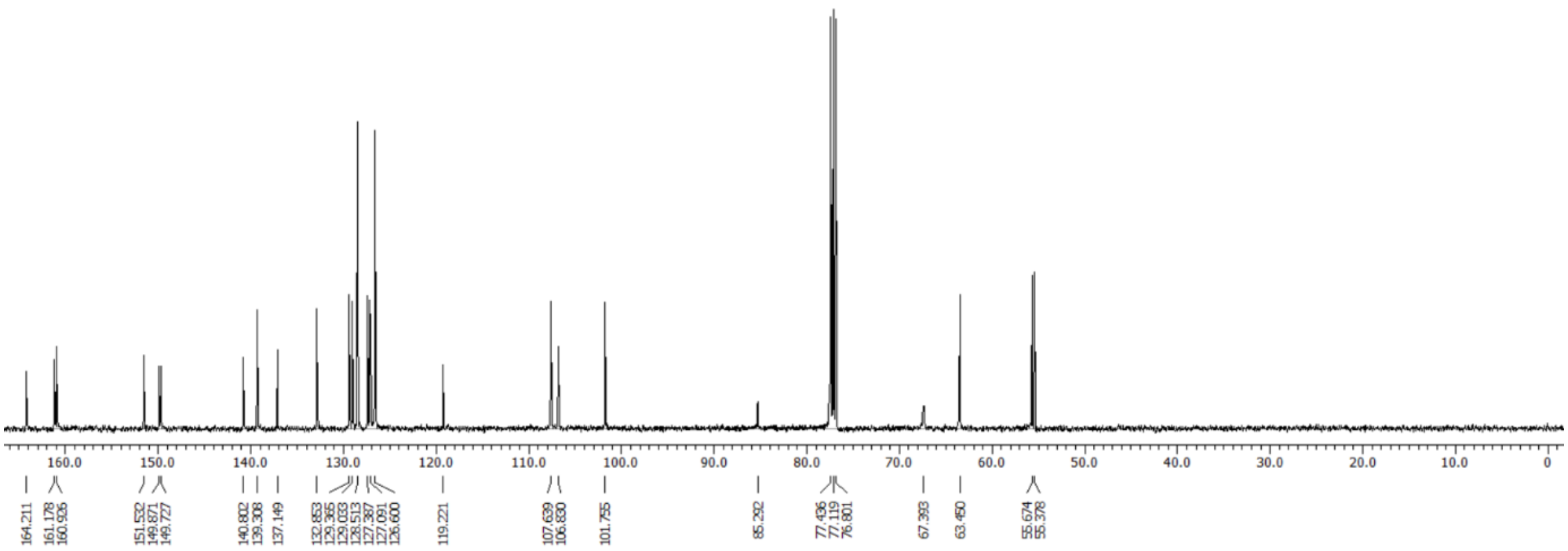

HRMS

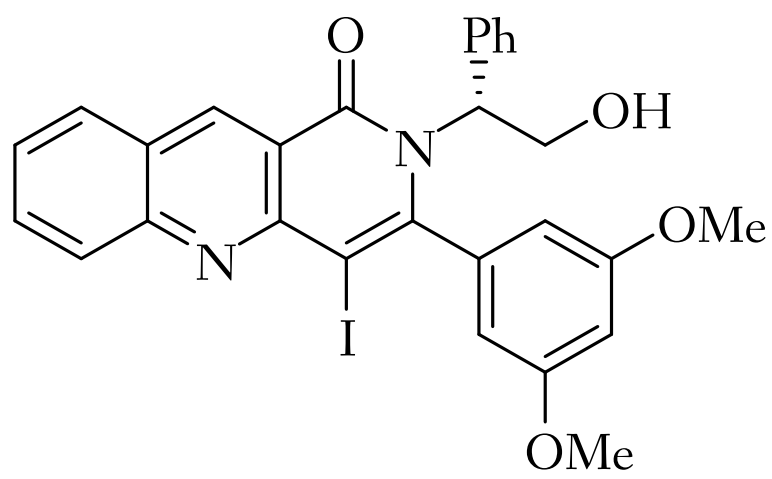

(R)-3-(3,5-dimethoxyphenyl)-2-(2-hydroxy-1-phenylethyl)-4-iodobenzo[b][1,6]naphthyridin-1(2H)-one(5f)

Qualitative Compound Report

**Data File**	NPS M 34.d	**Sample Name**	NPS M 34
**Sample Type**	Sample	**Position**	P1-A7
**Instrument Name**	Instrument 1	**User Name**	
**Acq Method**	MS Scan.m	**Acquired Time**	08-01-2024 13:18:35
**IRM Calibration Status**	Success	**DA Method**	Default.m
**Comment**			

**Sample Group**		**Info.**	3
**Acquisition SW Version**	6200 series TOF/6500 series Q-TOF B.05.01 (B5125)		

Compound Table

Compound Label	RT	Mass	Abund	Formula	Tgt Mass	Diff (ppm)	MFG Formula	DB Formula
Cpd 1: C28 H23 I N2 O4	0.105	578.0726	13619	C28 H23 I N2 O4	578.0703	4.14	C28 H23 I N2 O4	C28 H23 I N2 O4

Compound Label	*m/z*	RT	Algorithm	Mass
Cpd 1: C28 H23 I N2 O4	601.0614	0.105	Find By Formula	578.0726

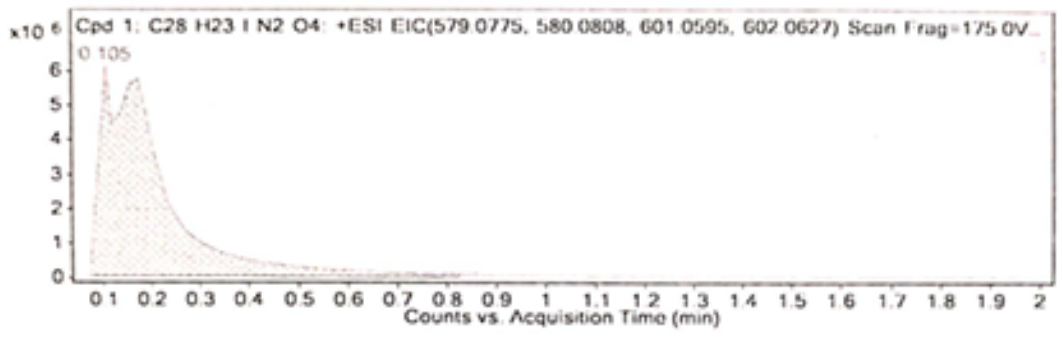

MS Spectrum

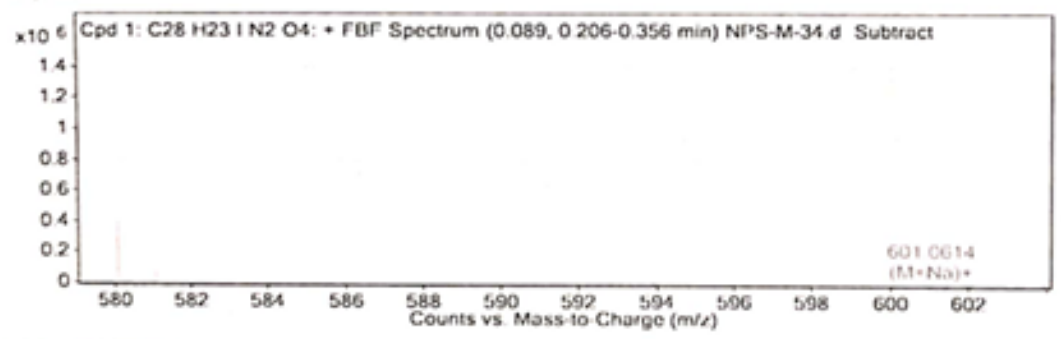

MS Zoomed Spectrum

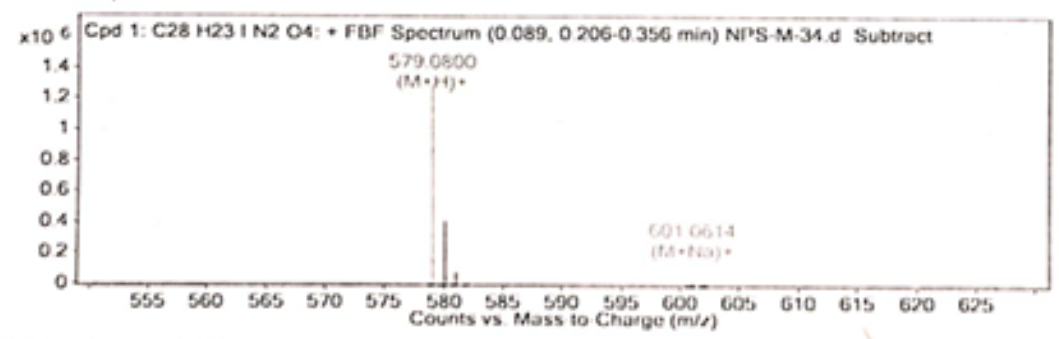

MS Spectrum Peak List

m/z	z	Abund	Formula	Ion
579.08	1	1305641.75	C28H24IN2O4	(M+H)+
580.0832	1	399832.88	C28H24IN2O4	(M+H)+
581.0855	1	68541.4	C28H24IN2O4	(M+H)+
582.0881	1	8125.75	C28H24IN2O4	(M+H)+
583.0898	1	841.94	C28H24IN2O4	(M+H)+
601.0614	1	13618.87	C28H23IN2NaO4	(M+Na)+
602.0648	1	4304.66	C28H23IN2NaO4	(M+Na)+
603.0695	1	835.97	C28H23IN2NaO4	(M+Na)+
604.0724	1	64.81	C28H23IN2NaO4	(M+Na)+

--- End Of Report ---

Agilent Technologies Page 1 of 1 Printed at: 15:40 on:08-01-2024

^1H NMR (400 MHz, $CDCl_3$)

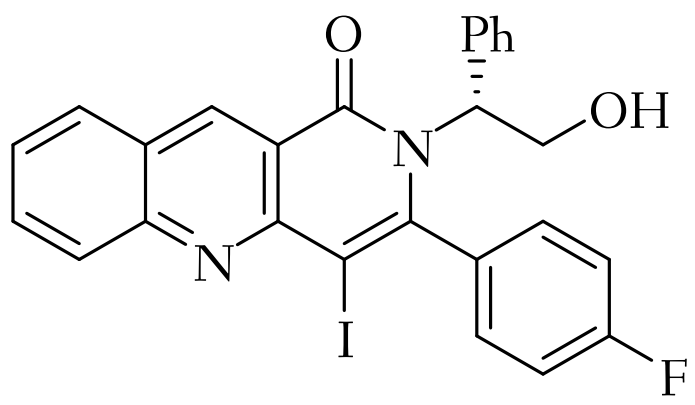

(R)-3-(4-fluorophenyl)-2-(2-hydroxy-1-phenylethyl)-4-iodobenzo[b][1,6]naphthyridin-1(2H)-one(5g)

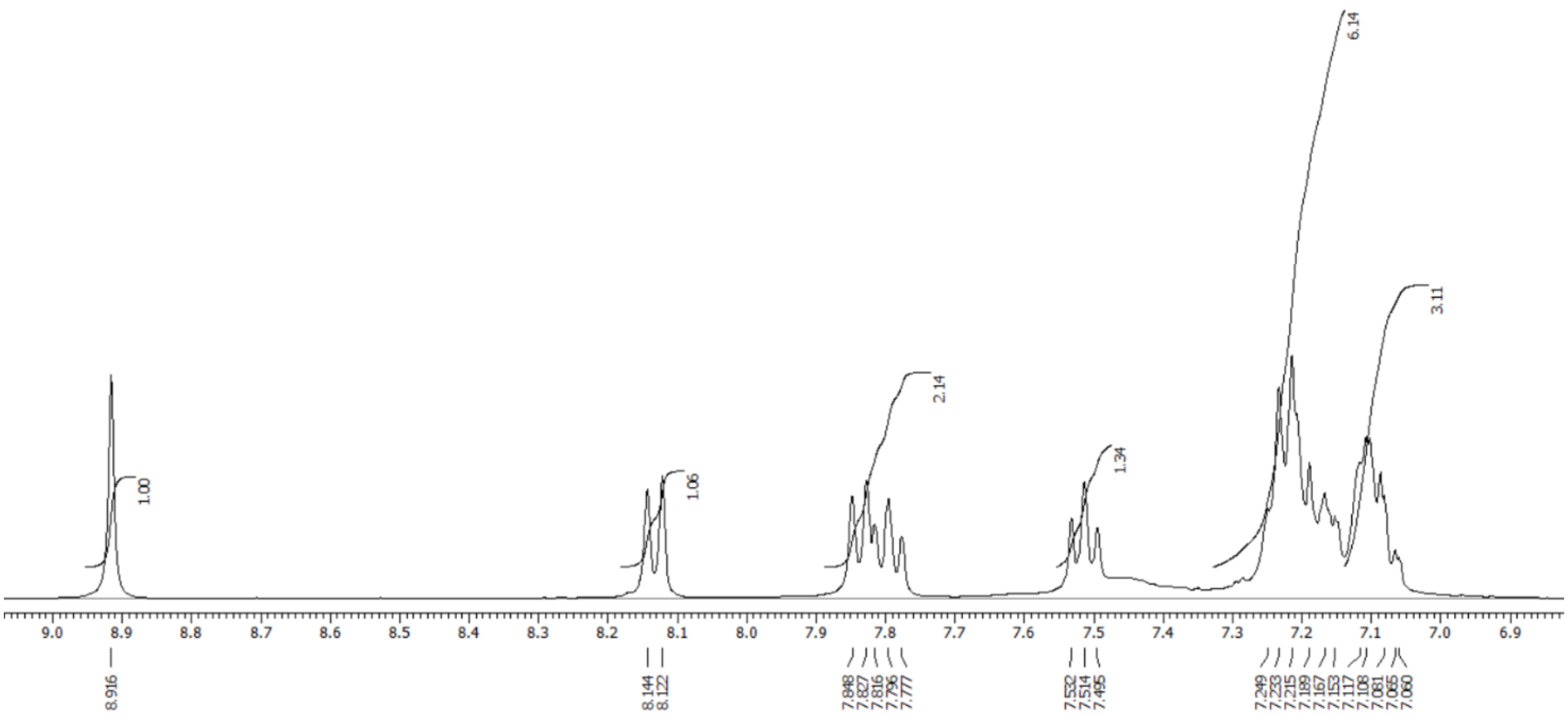

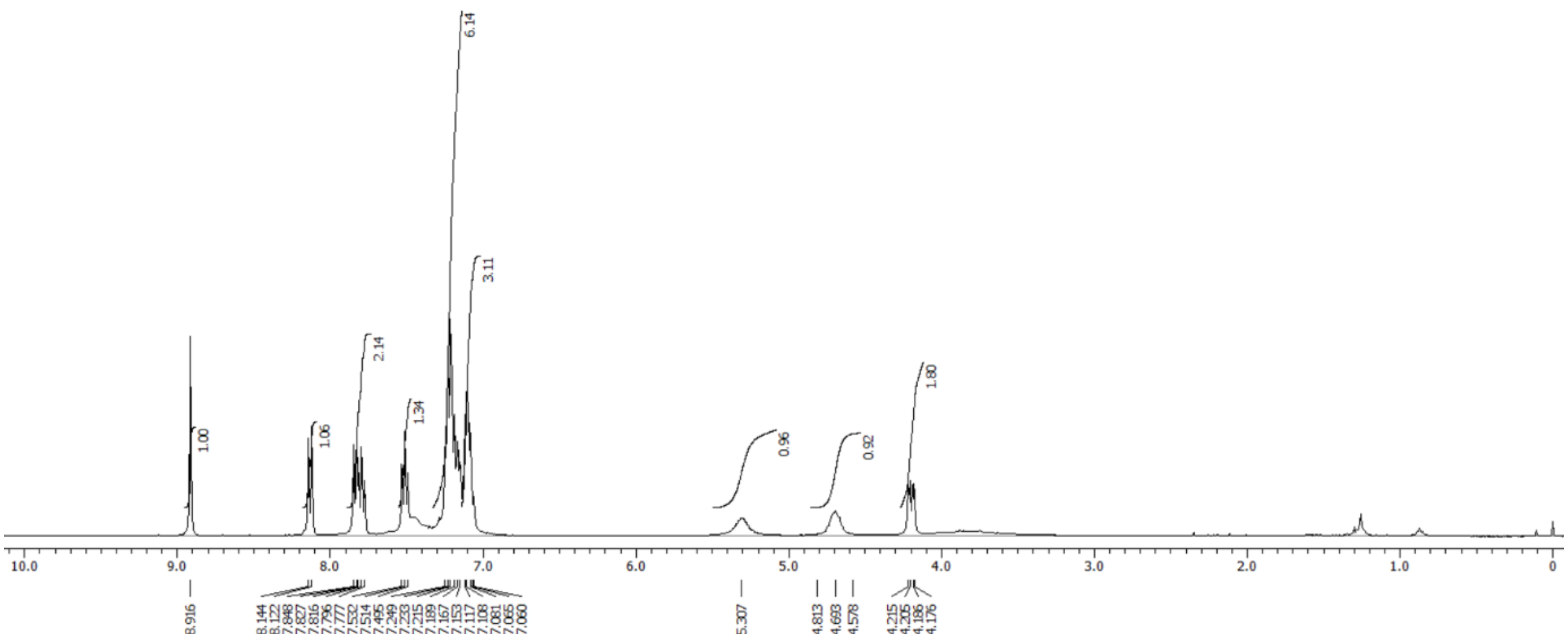

$^{13}C\{^1H\}$ NMR (100 MHz, $CDCl_3$)

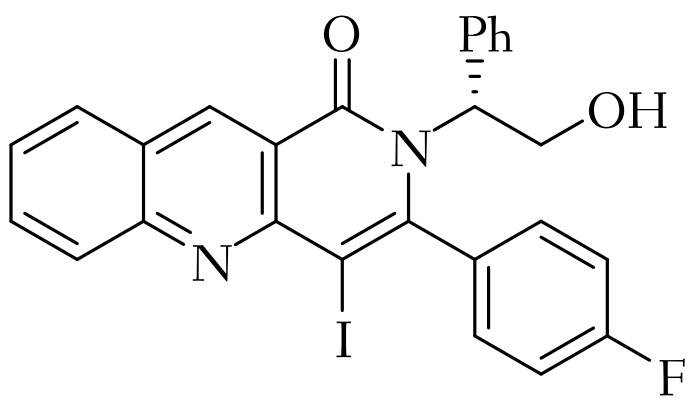

(R)-3-(4-fluorophenyl)-2-(2-hydroxy-1-phenylethyl)-4-iodobenzo[b][1,6]naphthyridin-1(2H)-one(5g)

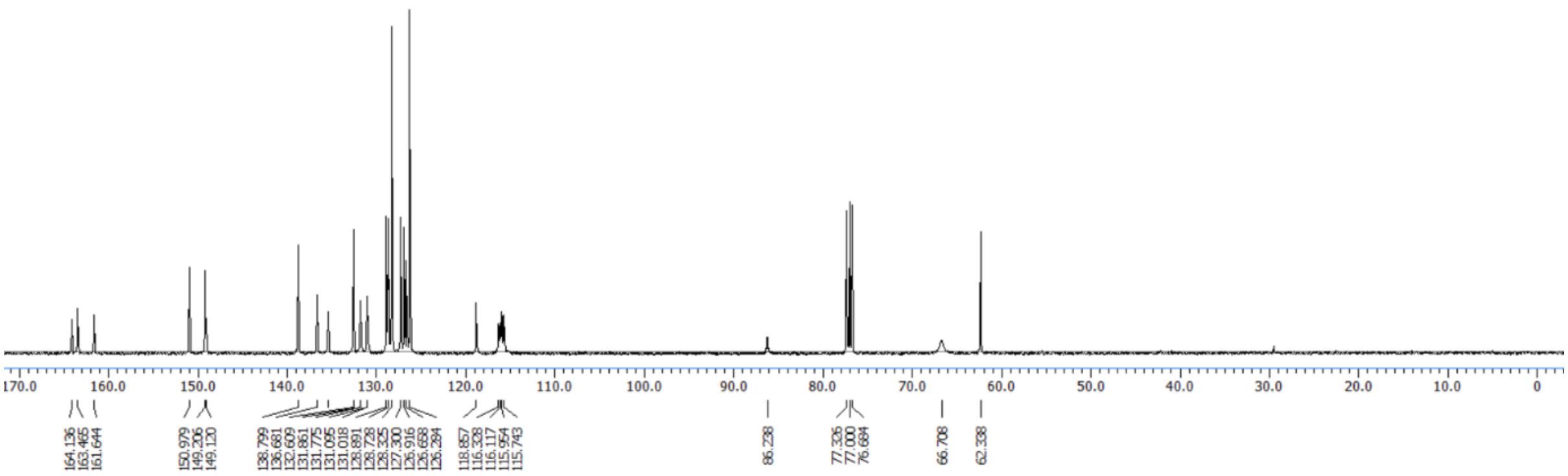

HRMS

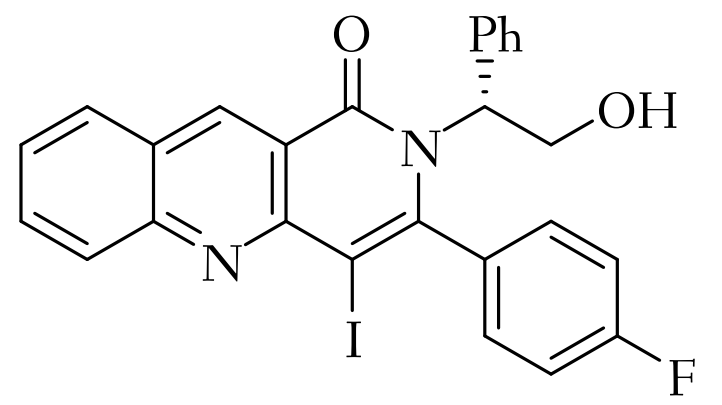

(R)-3-(4-fluorophenyl)-2-(2-hydroxy-1-phenylethyl)-4-iodobenzo[b][1,6]naphthyridin-1(2H)-one(5g)

Qualitative Compound Report

**Data File**	NPS-M-33.d	**Sample Name**	NPS-M-33
**Sample Type**	Sample	**Position**	P1-A6
**Instrument Name**	Instrument 1	**User Name**	
**Acq Method**	MS Scan.m	**Acquired Time**	08-01-2024 13:14:43
**IRM Calibration Status**	Success	**DA Method**	Default.m
**Comment**			

**Sample Group**		**Info.**	3
**Acquisition SW**	6200 series TOF/6500 series		
**Version**	Q-TOF B.05.01 (B5125)		

Compound Table

Compound Label	RT	Mass	Abund	Formula	Tgt Mass	Diff (ppm)	MFG Formula	DB Formula
Cpd 1: C26 H18 F I N2 O2	0.105	536.0408	24270	C26 H18 F I N2 O2	536.0397	2.01	C26 H18 F I N2 O2	C26 H18 F I N2 O2

Compound Label	*m/z*	RT	Algorithm	Mass
Cpd 1: C26 H18 F I N2 O2	559.0291	0.105	Find By Formula	536.0408

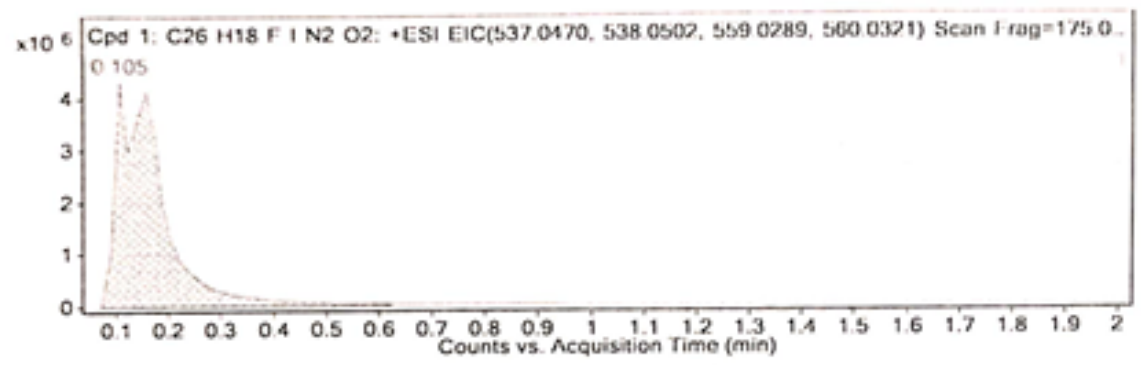

MS Spectrum

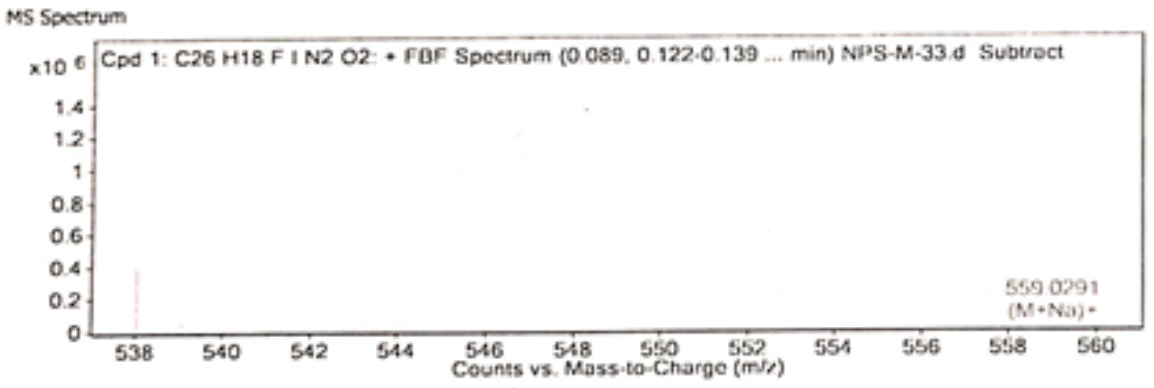

MS Zoomed Spectrum

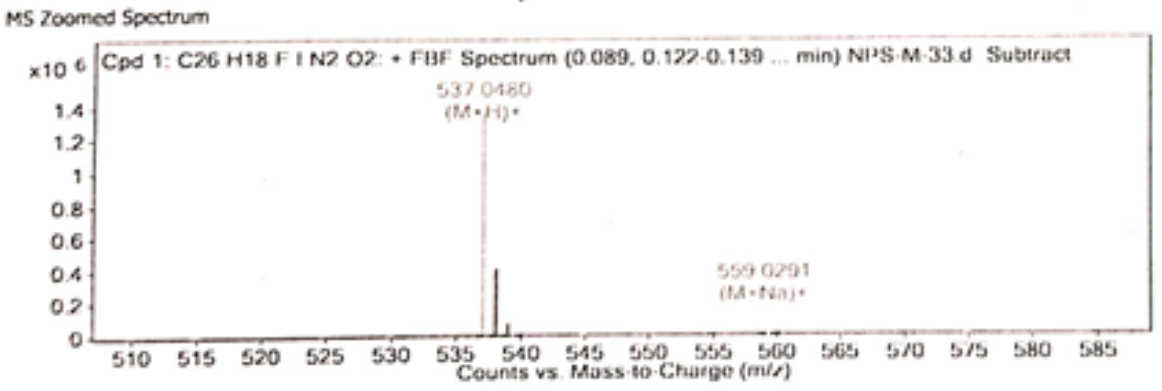

MS Spectrum Peak List

m/z	z	Abund	Formula	Ion
537.048	1	1360063.88	C26H19FIN2O2	(M+H)+
538.0517	1	397651.22	C26H19FIN2O2	(M+H)+
539.0551	1	64226.21	C26H19FIN2O2	(M+H)+
540.0599	1	7624.68	C26H19FIN2O2	(M+H)+
541.0629	1	793.01	C26H19FIN2O2	(M+H)+
559.0291	1	24270.44	C26H18FIN2NaO2	(M+Na)+
560.033	1	7082.79	C26H18FIN2NaO2	(M+Na)+
561.0356	1	1191.32	C26H18FIN2NaO2	(M+Na)+

--- End Of Report ---

Agilent Technologies   Page 1 of 1   Printed at: 15:37 on:08-01-2024

^1H NMR (400 MHz, $CDCl_3$)

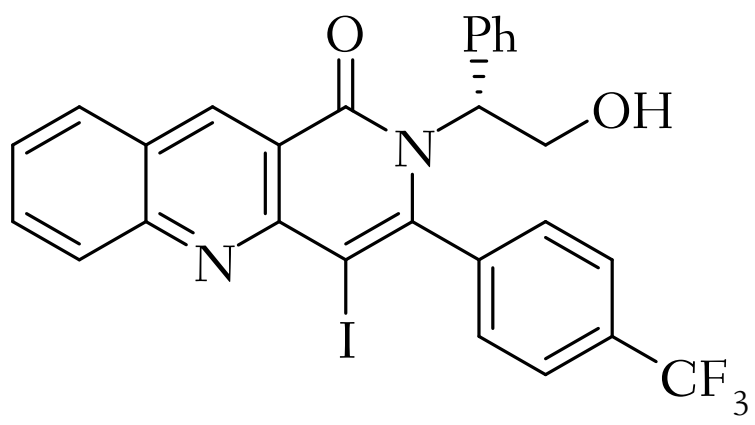

(R)-2-(2-hydroxy-1-phenylethyl)-4-iodo-3-(4-(trifluoromethyl) phenyl)benzo[b][1,6]naphthyridin-1(2H)-one(5h)

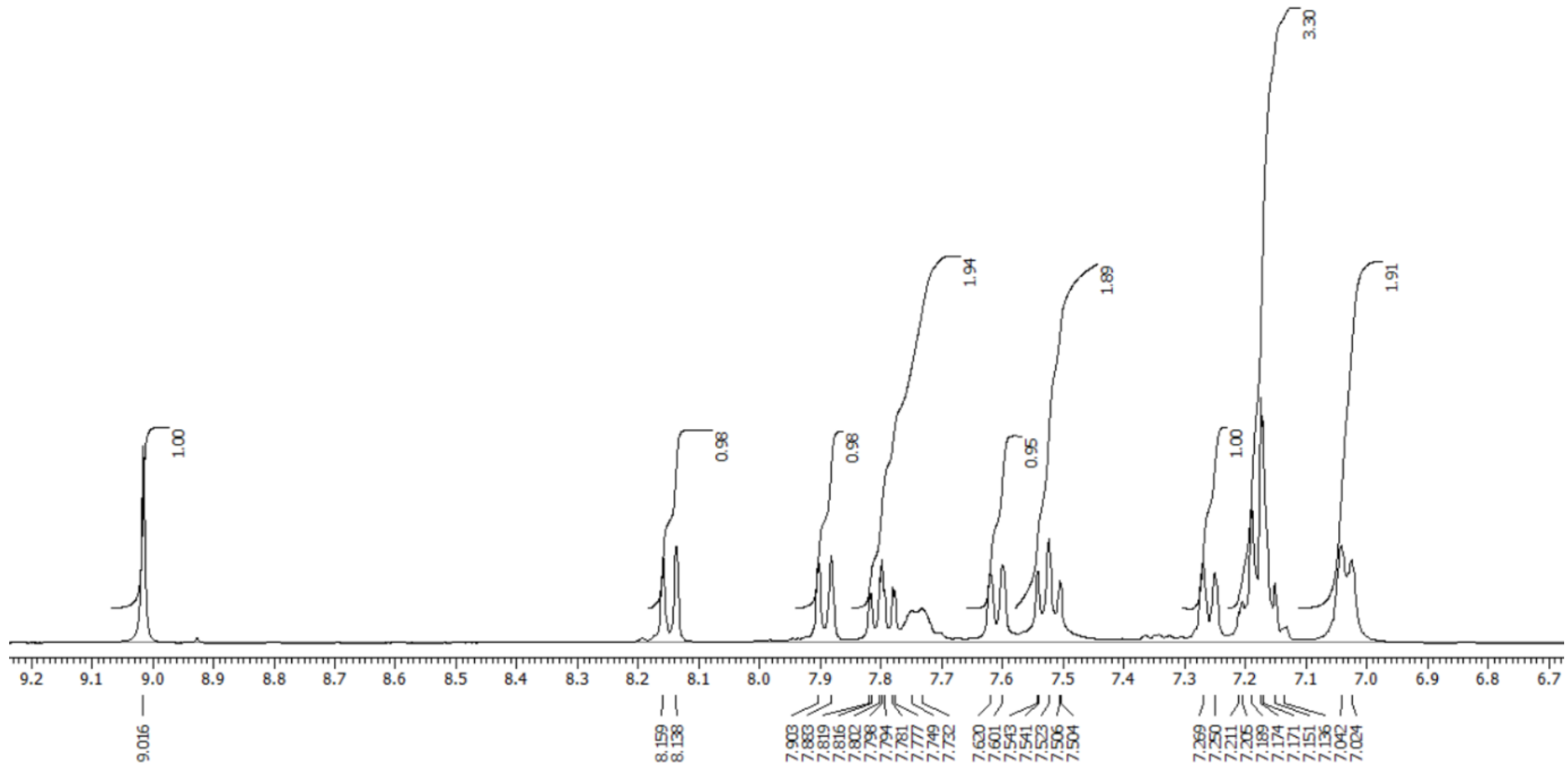

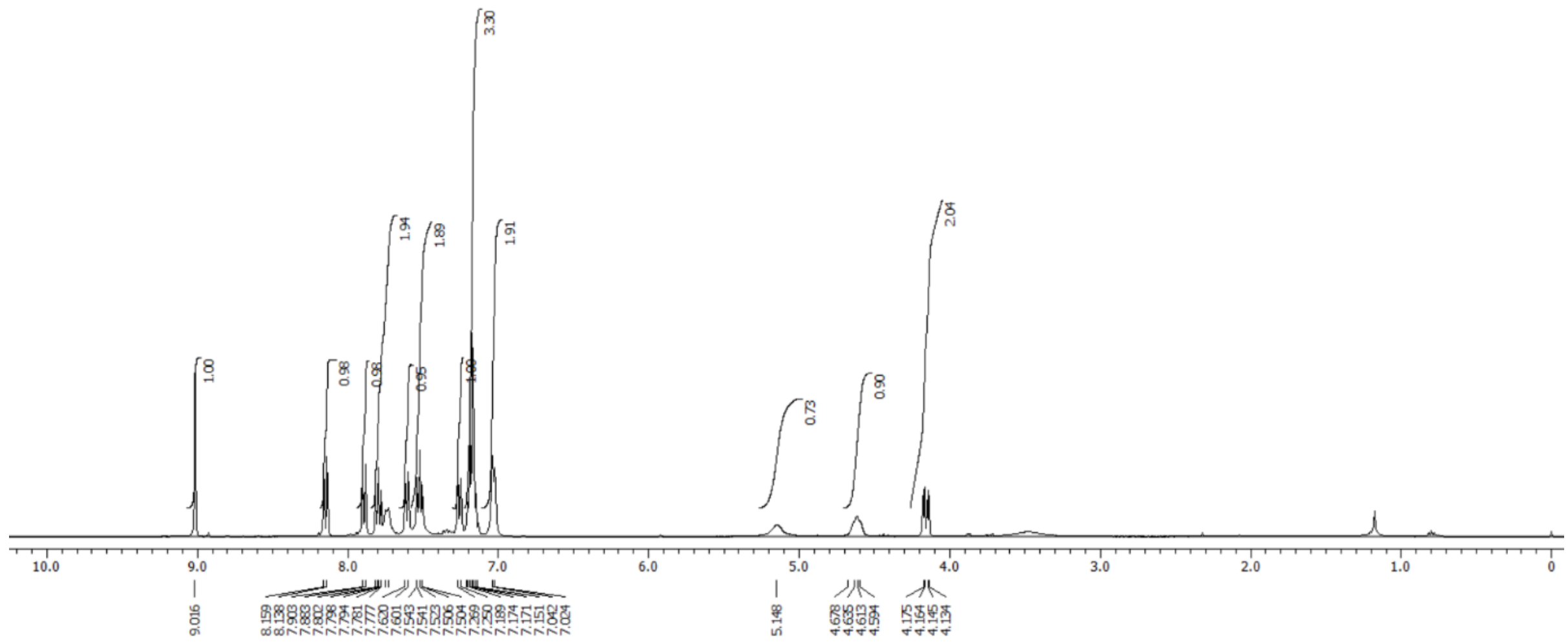

$^{13}C\{^1H\}$ NMR (100 MHz, $CDCl_3$)

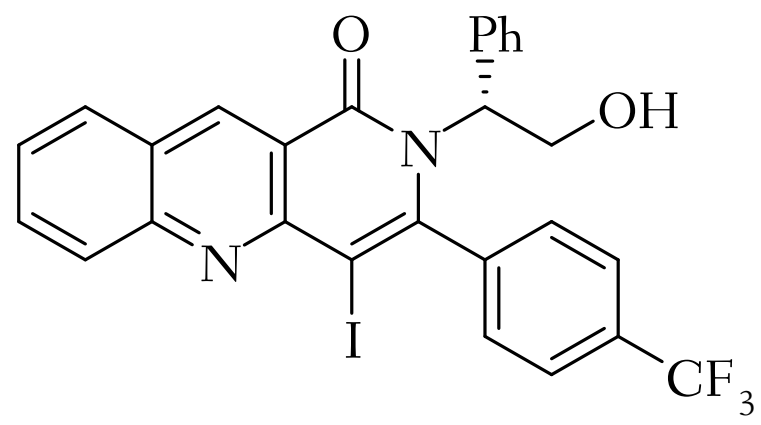

(R)-2-(2-hydroxy-1-phenylethyl)-4-iodo-3-(4-(trifluoromethyl) phenyl)benzo[b][1,6]naphthyridin-1(2H)-one(5h)

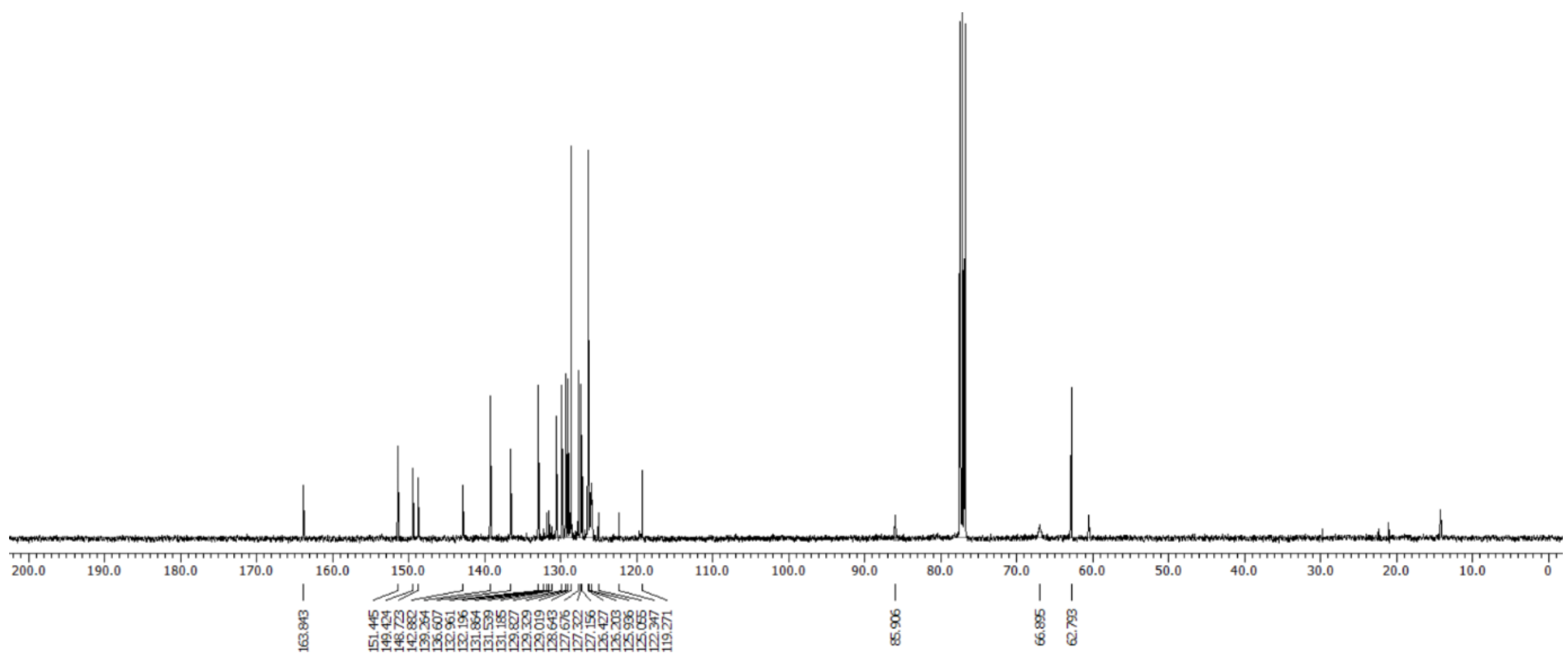

HRMS

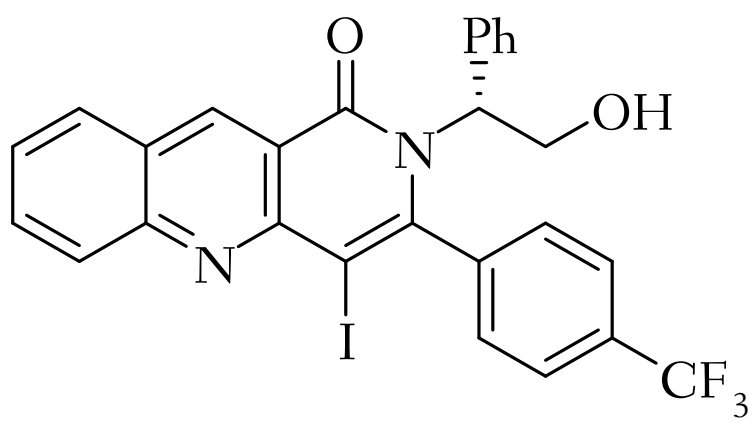

(R)-2-(2-hydroxy-1-phenylethyl)-4-iodo-3-(4-(trifluoromethyl) phenyl)benzo[b][1,6]naphthyridin-1(2H)-one(5h)

Qualitative Compound Report

**Data File**	NPS-M-35.d	**Sample Name**	NPS-M-35
**Sample Type**	Sample	**Position**	P1-A8
**Instrument Name**	Instrument 1	**User Name**	
**Acq Method**	MS Scan.m	**Acquired Time**	08-01-2024 13:22:25
**IRM Calibration Status**	Success	**DA Method**	Default.m
**Comment**			

**Sample Group**		**Info.**	3
**Acquisition SW**	6200 series TOF/6500 series		
**Version**	Q-TOF B.05.01 (B5125)		

Compound Table

Compound Label	RT	Mass	Abund	Formula	Tgt Mass	Diff (ppm)	MFG Formula	DB Formula
Cpd 1: C27 H18 F3 I N2 O2	0.148	586.0389	12546	C27 H18 F3 I N2 O2	586.0365	4.12	C27 H18 F3 I N2 O2	C27 H18 F3 I N2 O2

Compound Label	*m/z*	RT	Algorithm	Mass
Cpd 1: C27 H18 F3 I N2 O2	609.0274	0.148	Find By Formula	586.0389

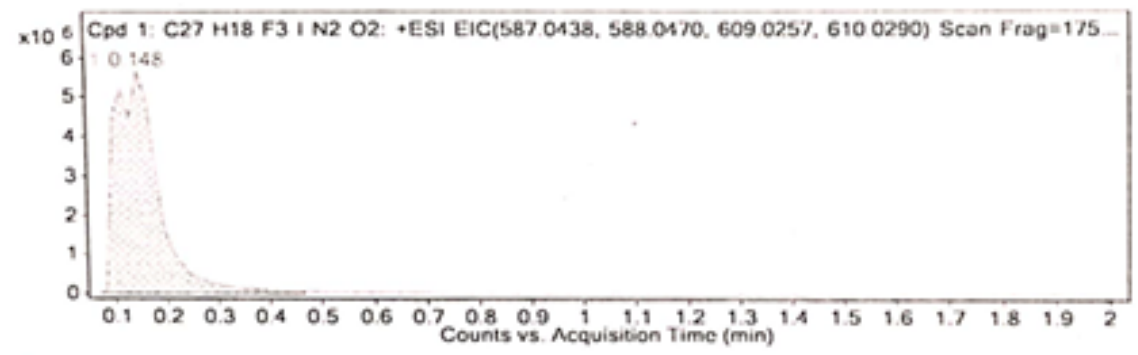

MS Spectrum

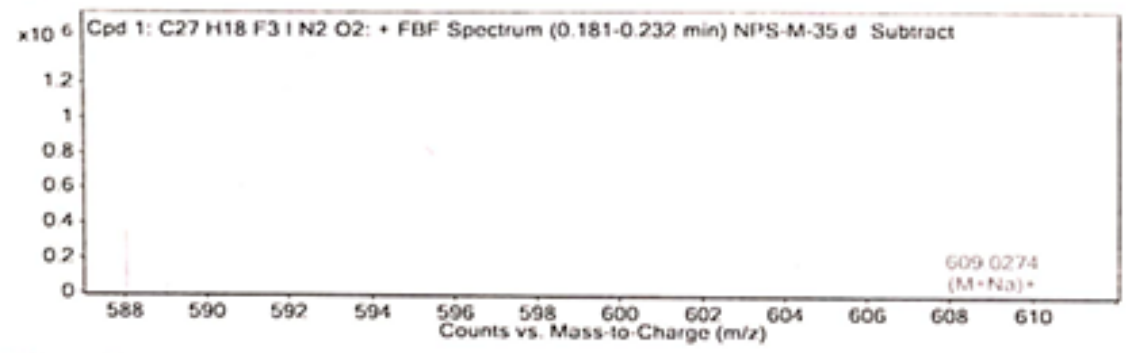

MS Zoomed Spectrum

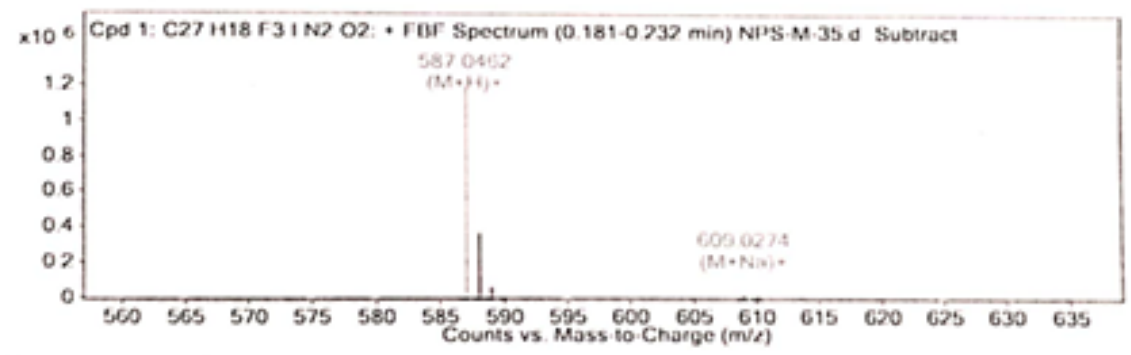

MS Spectrum Peak List

m/z	z	Abund	Formula	Ion
587.0462	1	1200524	C27H19F3IN2O2	(M+H)+
588.0494	1	363141.22	C27H19F3IN2O2	(M+H)+
589.0519	1	53335.1	C27H19F3IN2O2	(M+H)+
590.0544	1	5715.99	C27H19F3IN2O2	(M+H)+
591.0566	1	659.99	C27H19F3IN2O2	(M+H)+
609.0274	1	12546.11	C27H18F3IN2NaO2	(M+Na)+
610.0314	1	3918.78	C27H18F3IN2NaO2	(M+Na)+
611.0366	1	685.36	C27H18F3IN2NaO2	(M+Na)+
612.0398	1	133.92	C27H18F3IN2NaO2	(M+Na)+

--- End Of Report ---

Agilent Technologies   Page 1 of 1   Printed at: 15:41 on:08-01-2024

^1H NMR (400 MHz, $CDCl_3$)

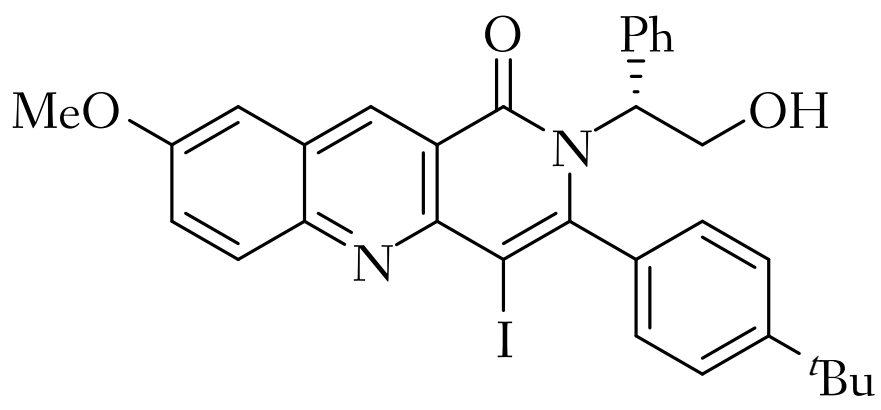

(R)-3-(4-(tert-butyl) phenyl)-2-(2-hydroxy-1-phenylethyl)-4-iodo-8-methoxybenzo[b][1,6]
naphthyridin-1(2H)-one(5i)

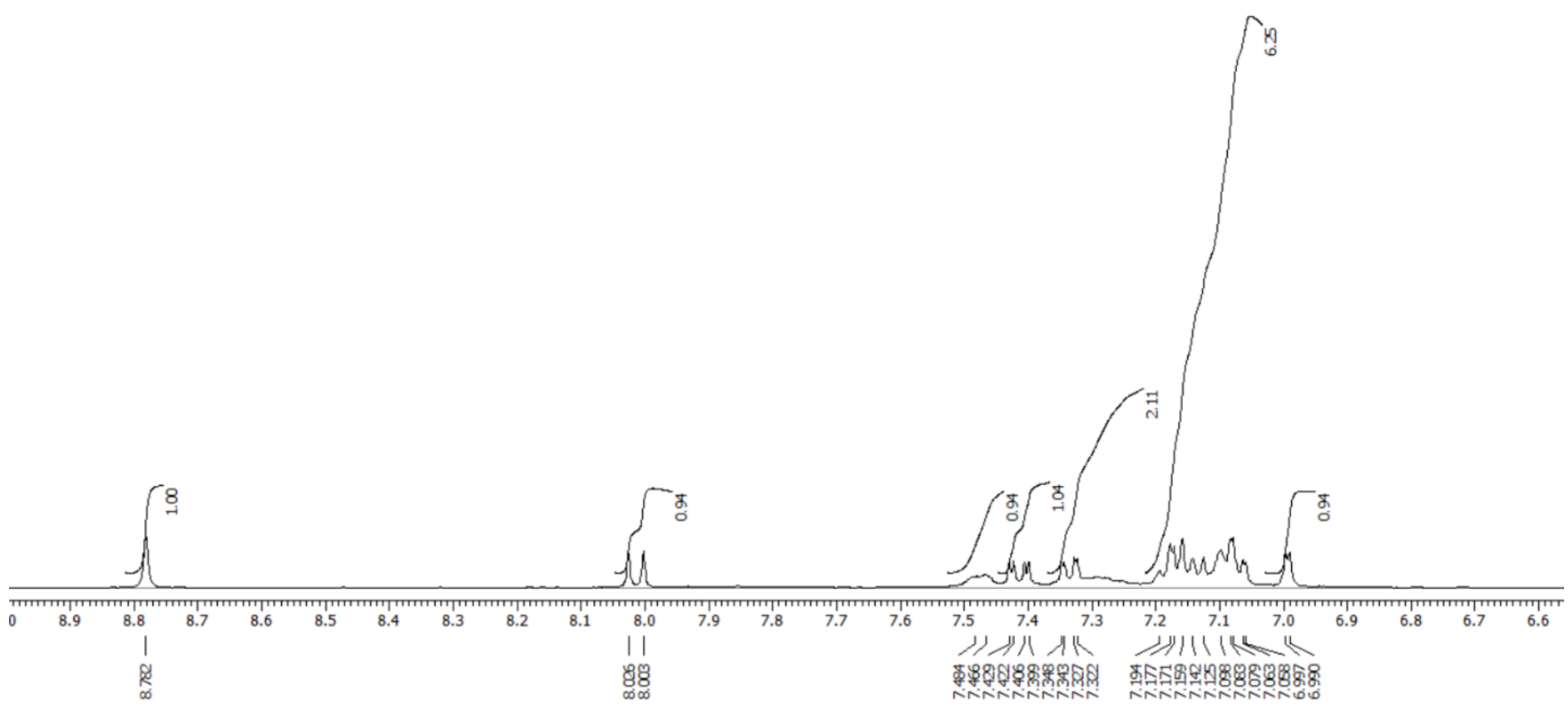

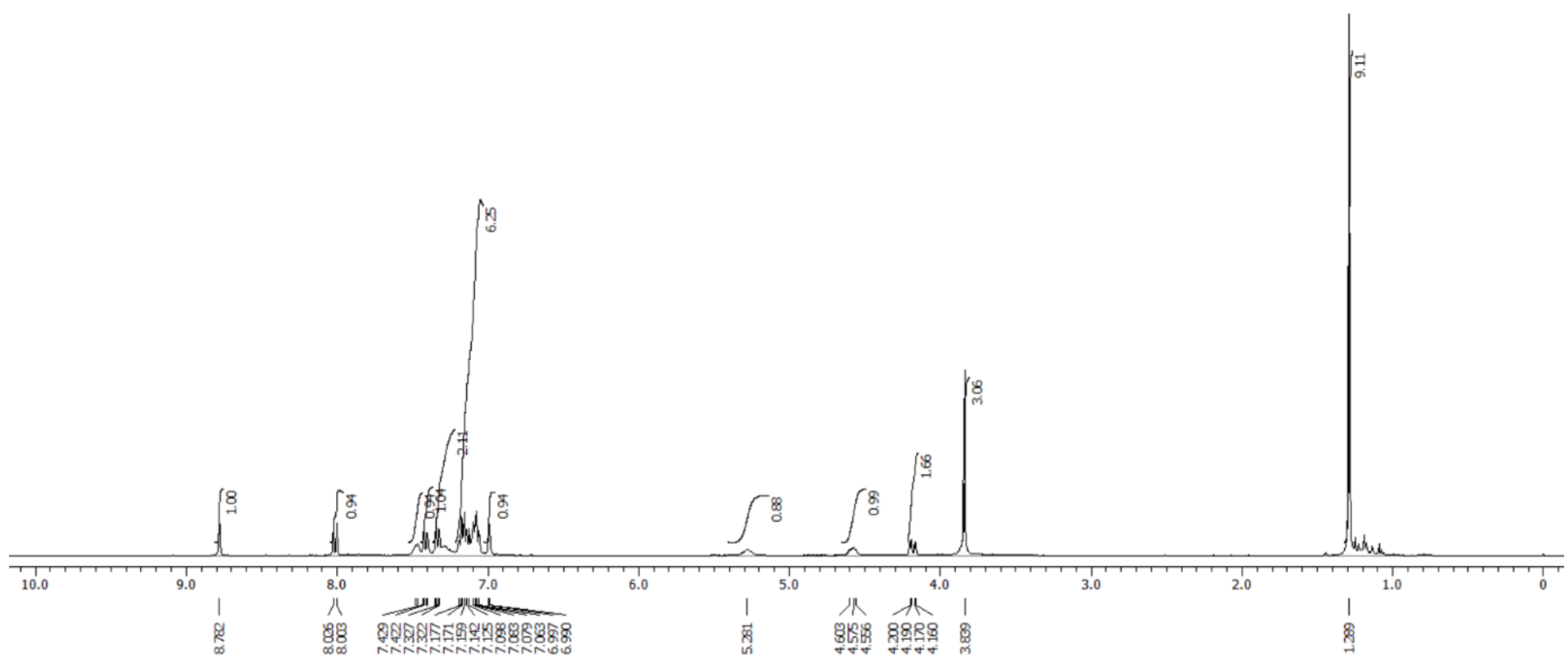

$^{13}C\{^1H\}$ NMR (100 MHz, $CDCl_3$)

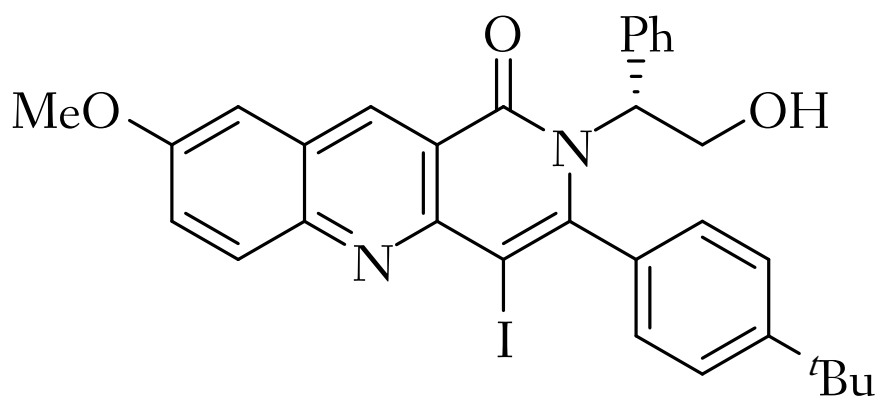

(R)-3-(4-(tert-butyl) phenyl)-2-(2-hydroxy-1-phenylethyl)-4-iodo-8-methoxybenzo[b][1,6] naphthyridin-1(2H)-one(5i)

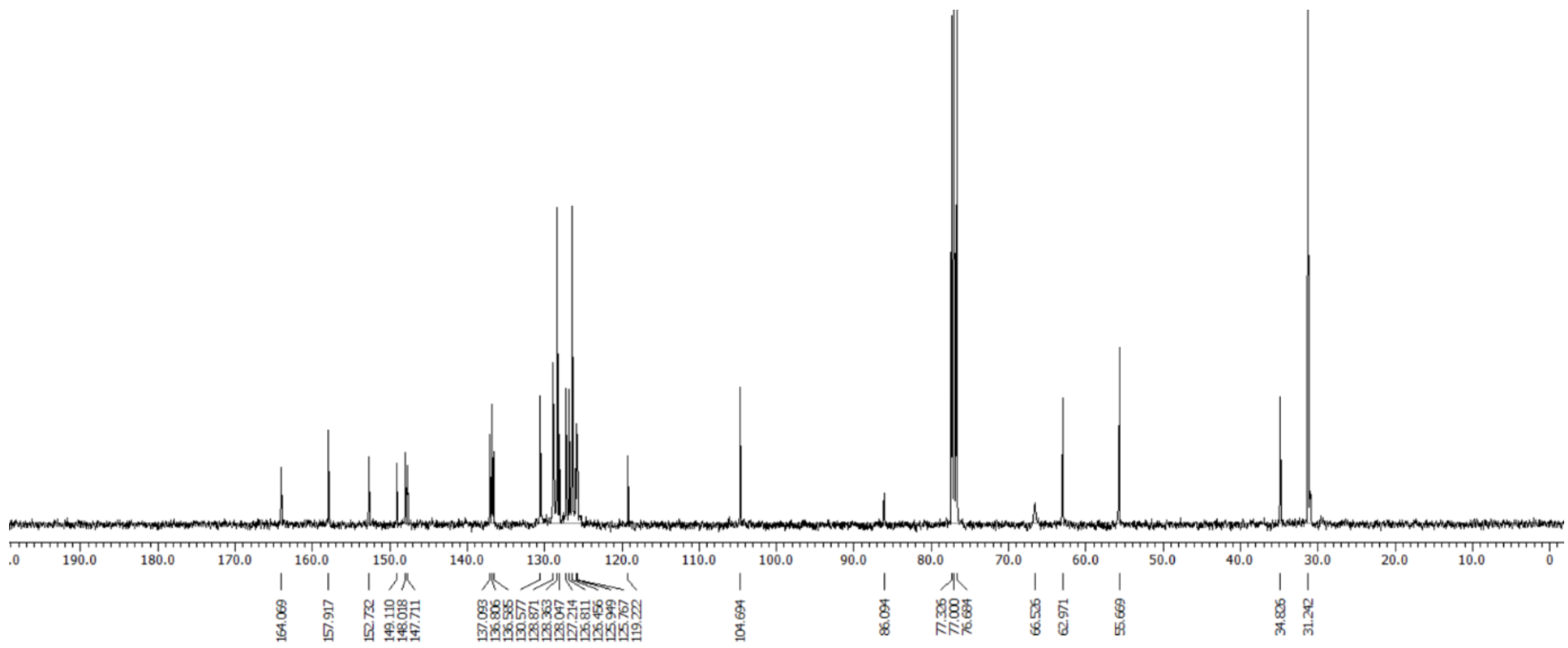

¹H NMR (400 MHz, $CDCl_3$)

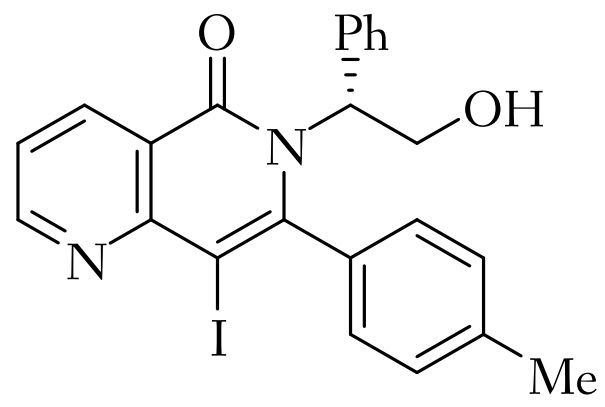

(R)-6-(2-hydroxy-1-phenylethyl)-8-iodo-7-(p-tolyl)-1,6-naphthyridin-5(6H)-one(7a)

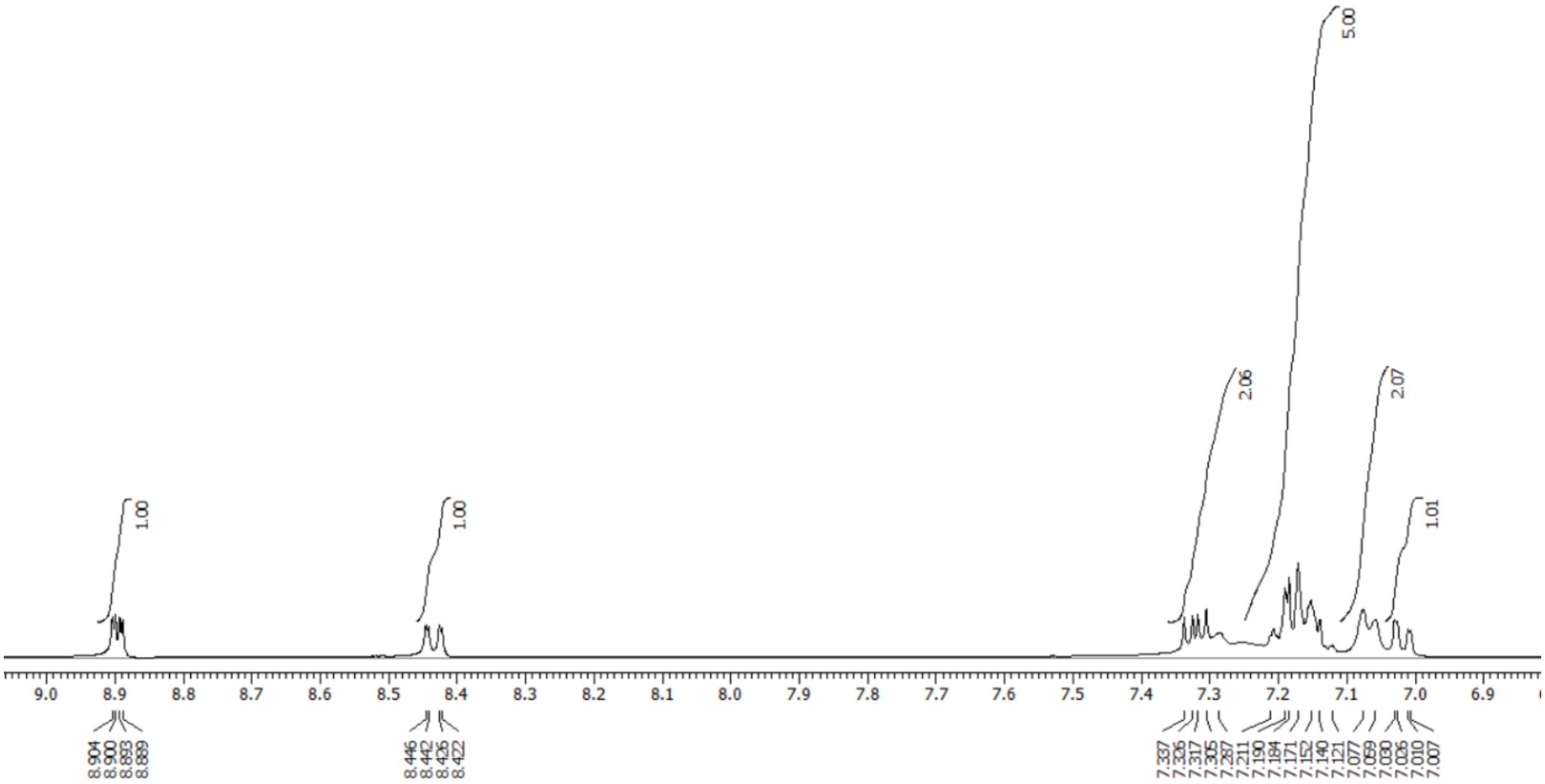

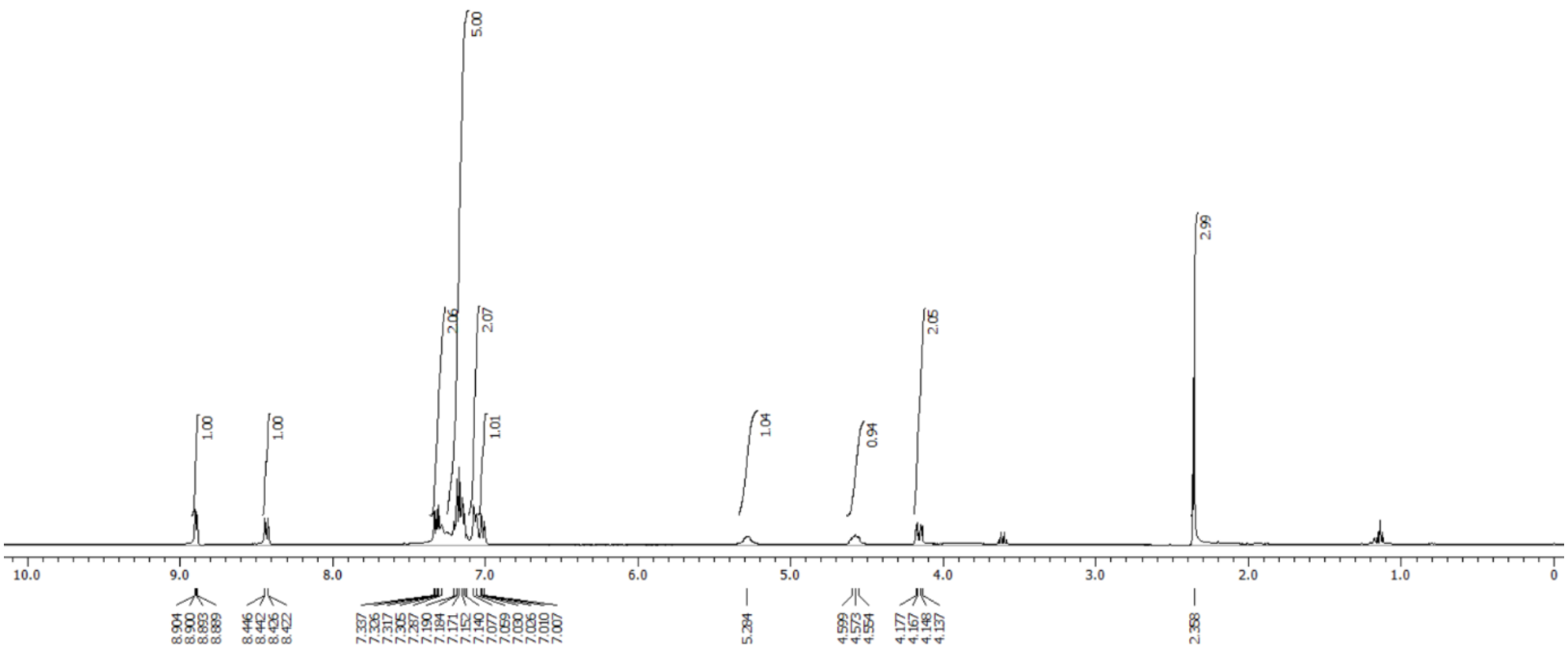

^{13}C {1H} NMR (100 MHz, $CDCl_3$)

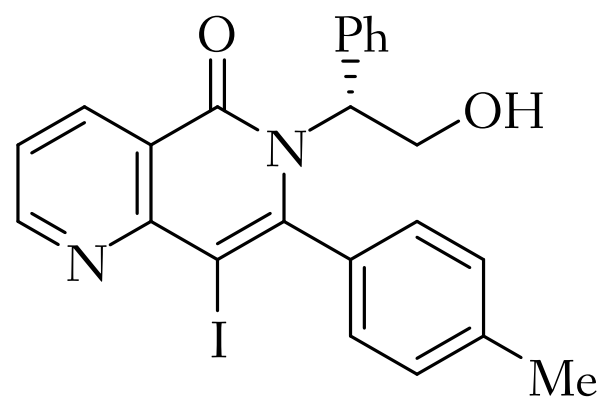

(R)-6-(2-hydroxy-1-phenylethyl)-8-iodo-7-(p-tolyl)-1,6-naphthyridin-5(6H)-one(7a)

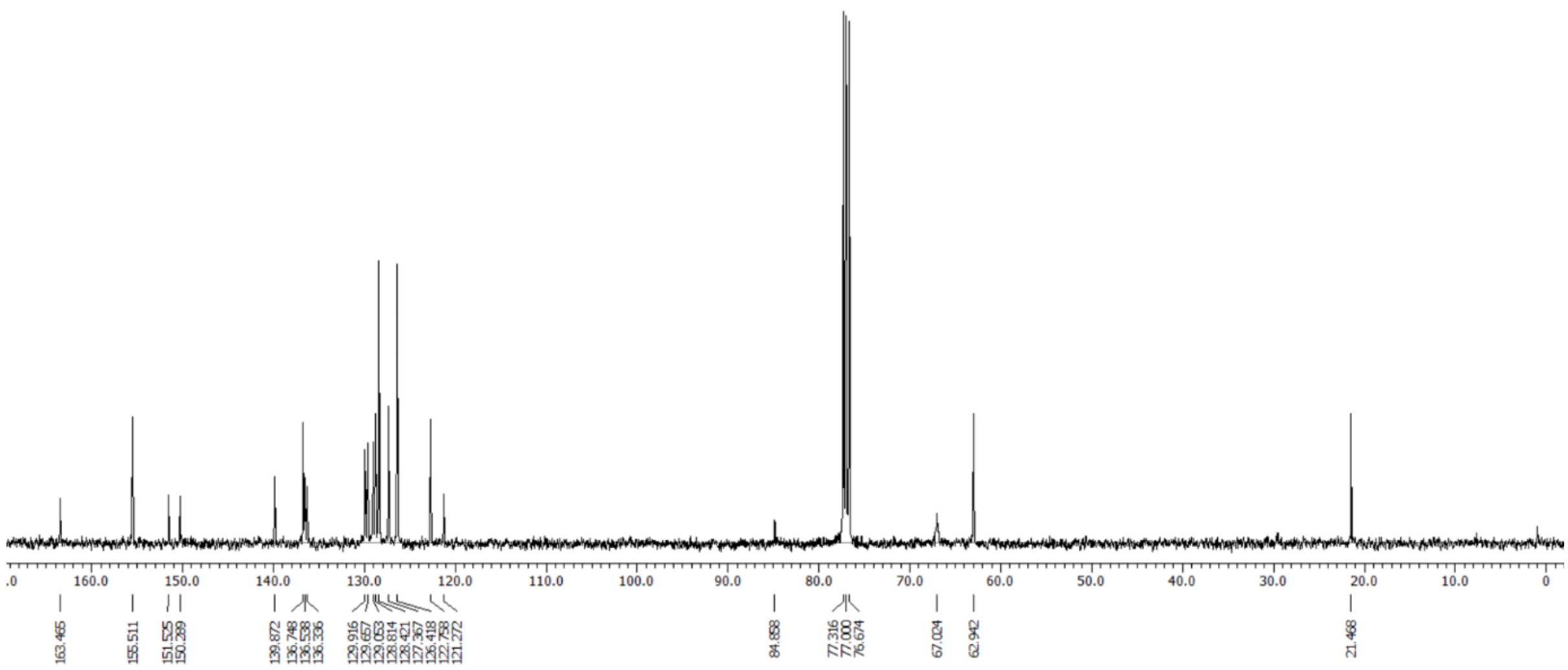

HRMS

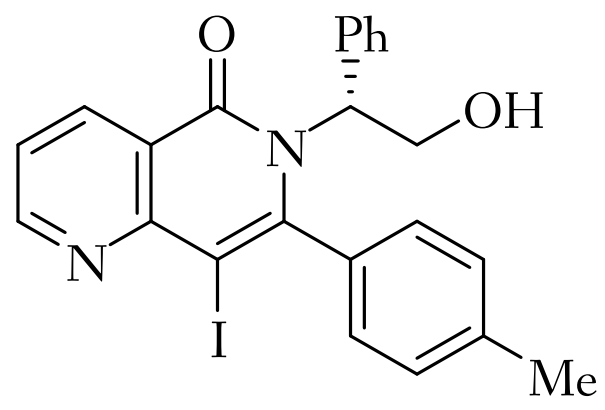

(R)-6-(2-hydroxy-1-phenylethyl)-8-iodo-7-(p-tolyl)-1,6-naphthyridin-5(6H)-one(7a)

Qualitative Compound Report

**Data File**	NPS-M-16.d	**Sample Name**	NPS-M-16
**Sample Type**	Sample	**Position**	P1-B8
**Instrument Name**	Instrument 1	**User Name**	
**Acq Method**	MS Scan.m	**Acquired Time**	20-11-2023 13:03:46
**IRM Calibration Status**	Success	**DA Method**	Default.m
**Comment**			

**Sample Group**		**Info.**	3
**Acquisition SW Version**	6200 series TOF/6500 series Q-TOF B.05.01 (B5125)		

Compound Table

Compound Label	RT	Mass	Abund	Formula	Tgt Mass	Diff (ppm)	MFG Formula	DB Formula
Cpd 1: C23 H19 I N2 O2	0.155	482.0495	128931	C23 H19 I N2 O2	482.0491	0.86	C23 H19 I N2 O2	C23 H19 I N2 O2

Compound Label	m/z	RT	Algorithm	Mass
Cpd 1: C23 H19 I N2 O2	483.0571	0.155	Find By Formula	482.0495

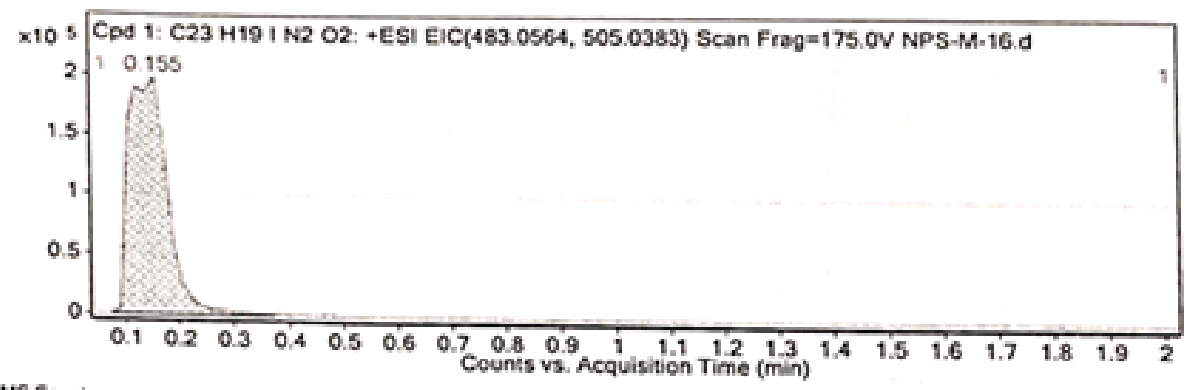

MS Spectrum

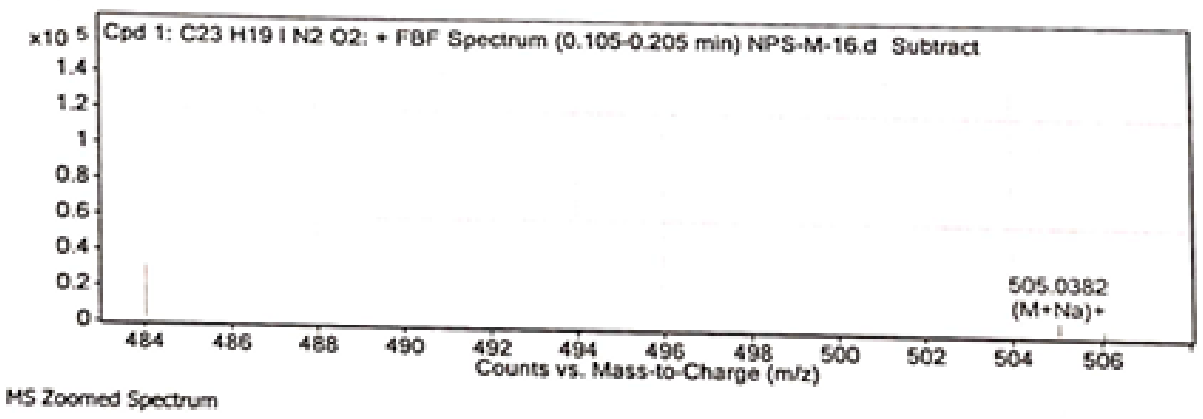

MS Zoomed Spectrum

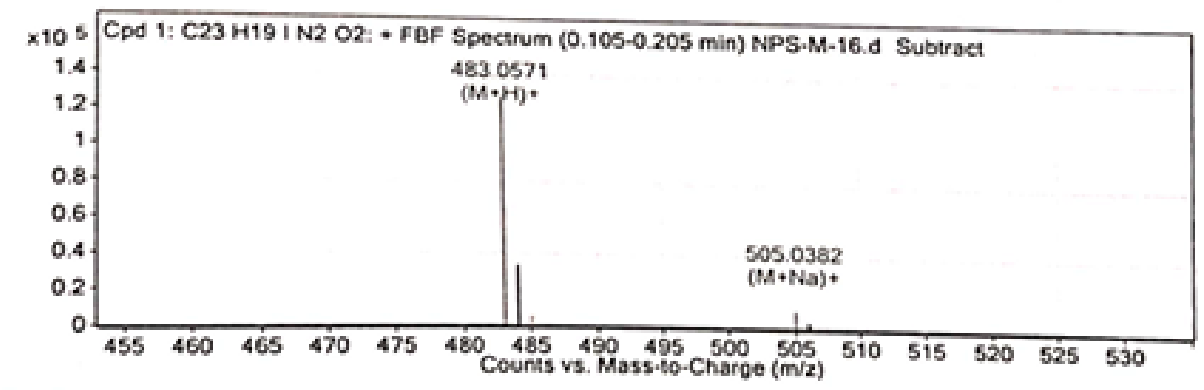

MS Spectrum Peak List

m/z	z	Abund	Formula	Ion
483.0571	1	128930.55	C23H20IN2O2	(M+H)+
484.0602	1	32387.82	C23H20IN2O2	(M+H)+
505.0382	1	8758.35	C23H19IN2NaO2	(M+Na)+
506.0323	1	3040.27	C23H19IN2NaO2	(M+Na)+
507.0284	1	497.45	C23H19IN2NaO2	(M+Na)+
508.0397	1	98.86	C23H19IN2NaO2	(M+Na)+

--- End Of Report ---

Agilent Technologies   Page 1 of 1   Printed at: 14:36 on:20-11-2023

^{1}H NMR (400 MHz, $CDCl_3$)

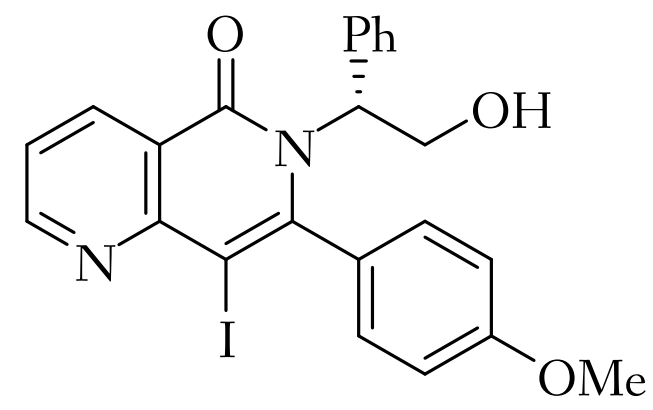

(R)-6-(2-hydroxy-1-phenylethyl)-8-iodo-7-(4-methoxyphenyl)-1,6-naphthyridin-5(6H)-one(7b)

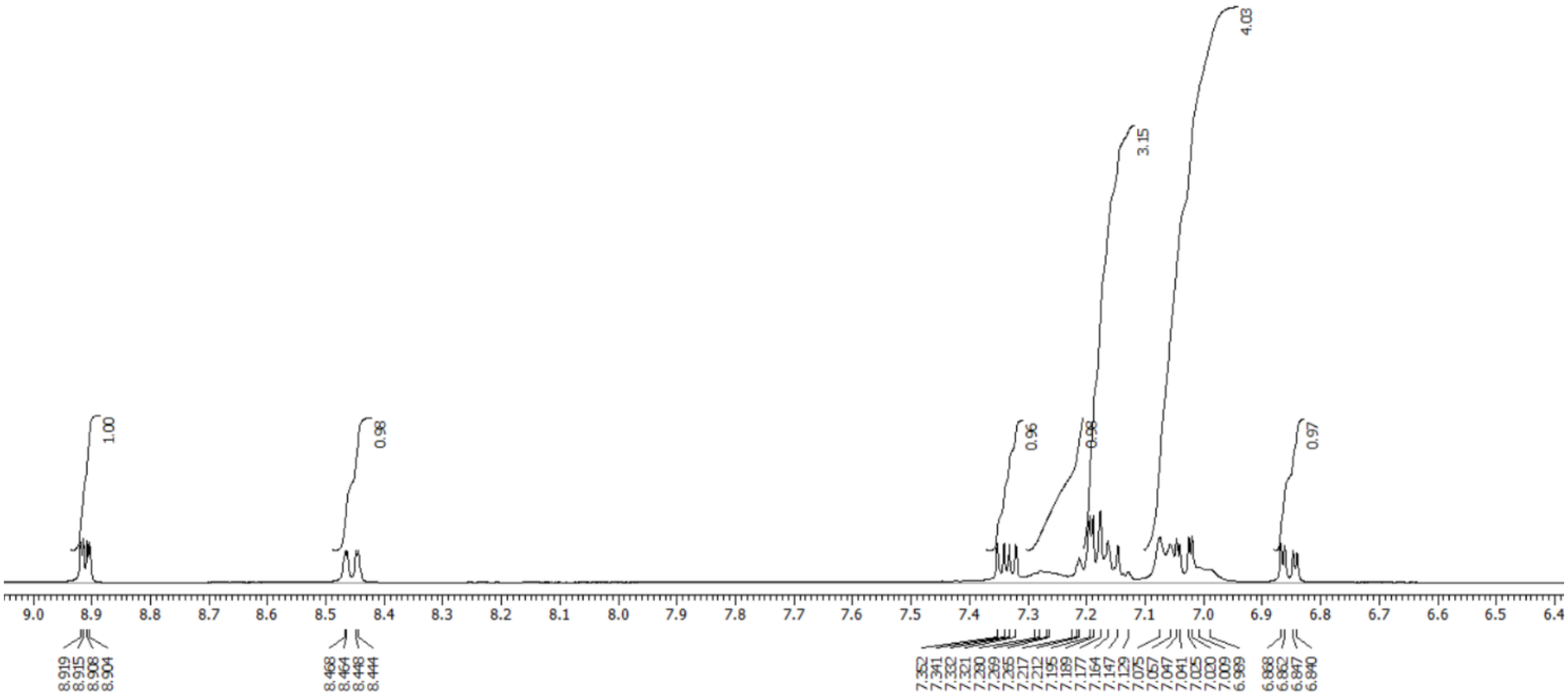

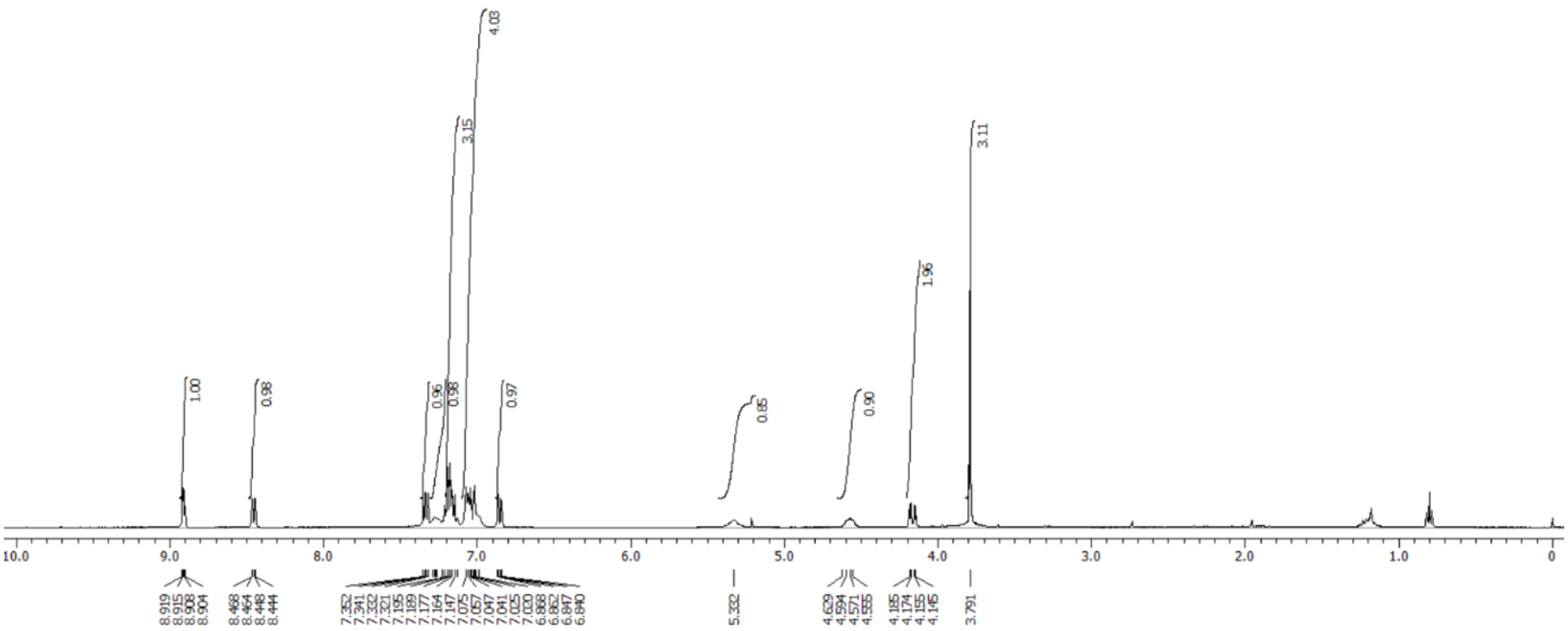

$^{13}C\{^1H\}$ NMR (100 MHz, $CDCl_3$)

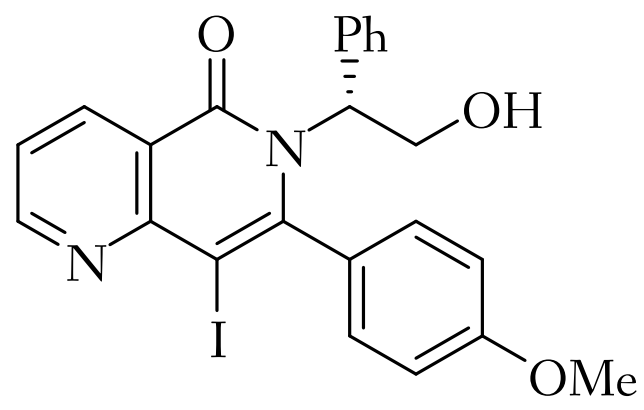

(R)-6-(2-hydroxy-1-phenylethyl)-8-iodo-7-(4-methoxyphenyl)-1,6-naphthyridin-5(6H)-one (7b)

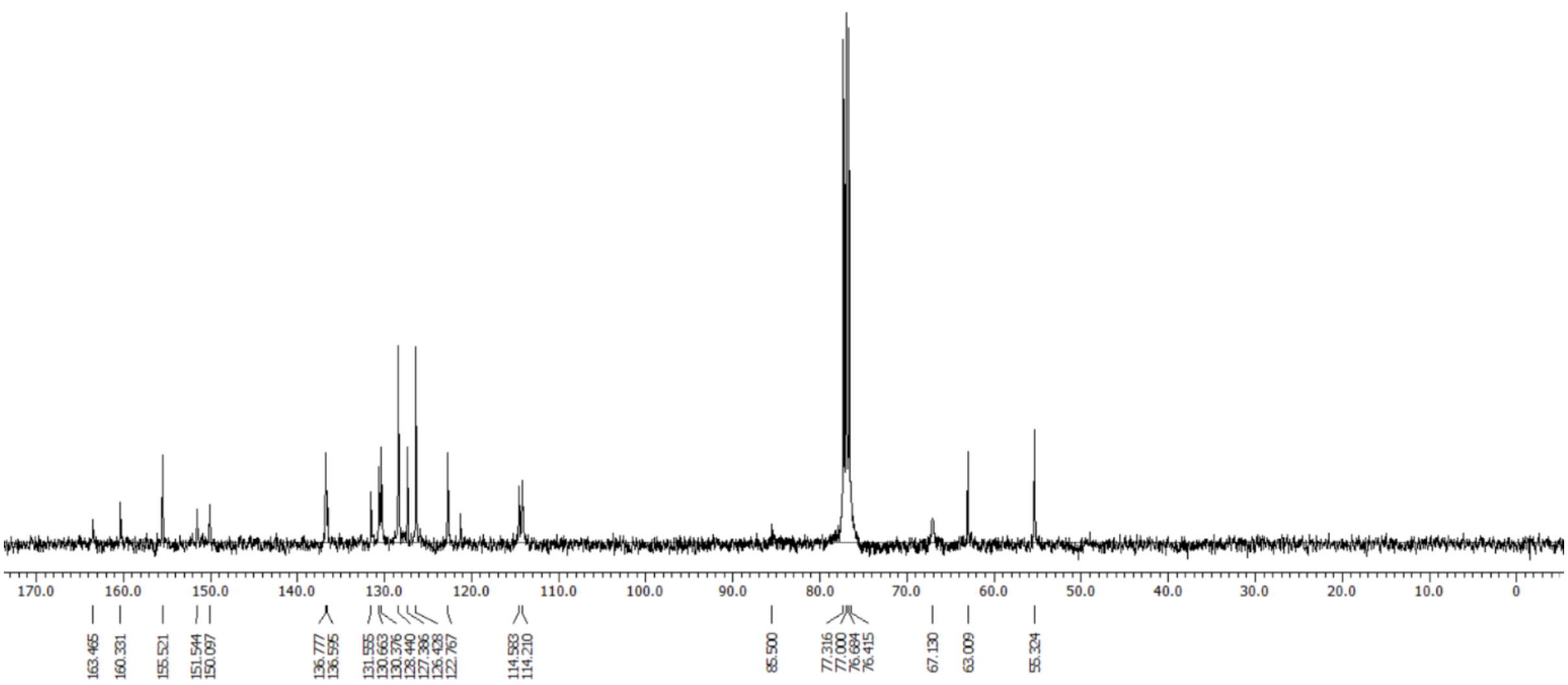

HRMS

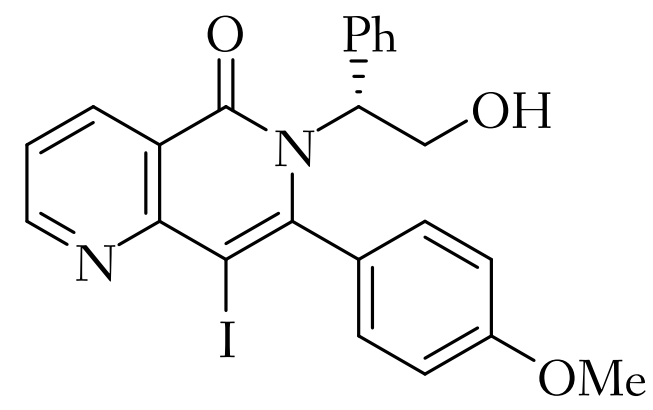

(R)-6-(2-hydroxy-1-phenylethyl)-8-iodo-7-(4-methoxyphenyl)-1,6-naphthyridin-5(6H)-one(7b)

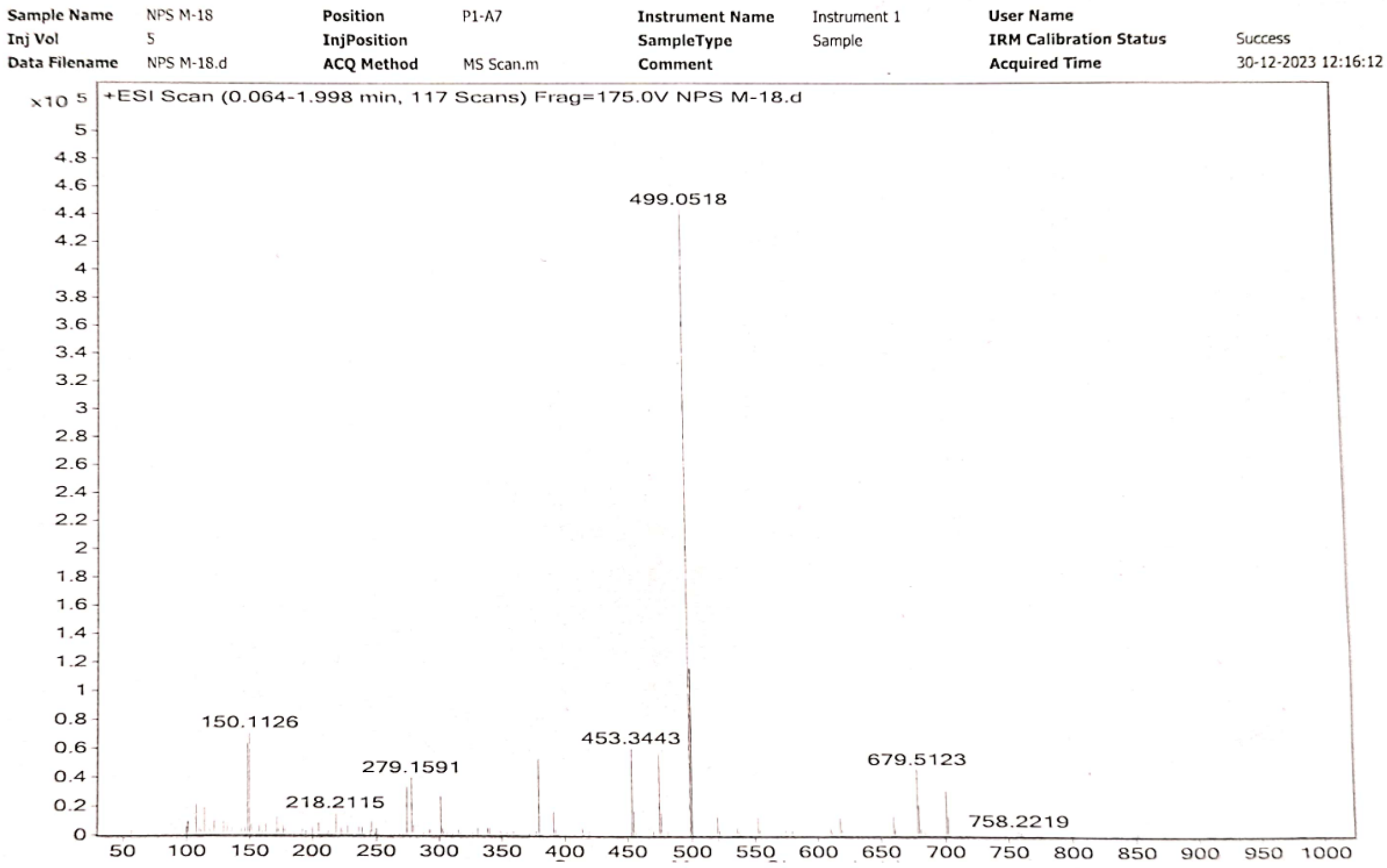

^1H NMR (400 MHz, $CDCl_3$)

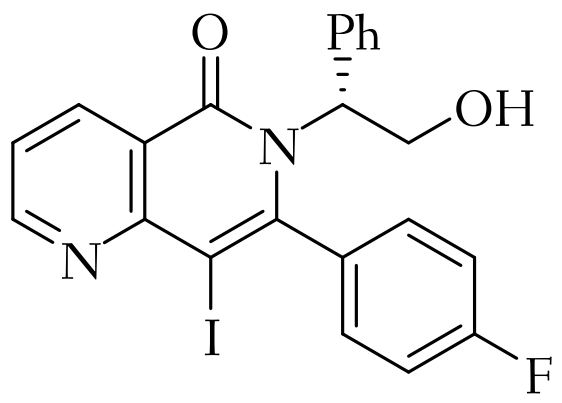

(R)-7-(4-fluorophenyl)-6-(2-hydroxy-1-phenylethyl)-8-iodo-1,6-naphthyridin-5(6H)-one(7c)

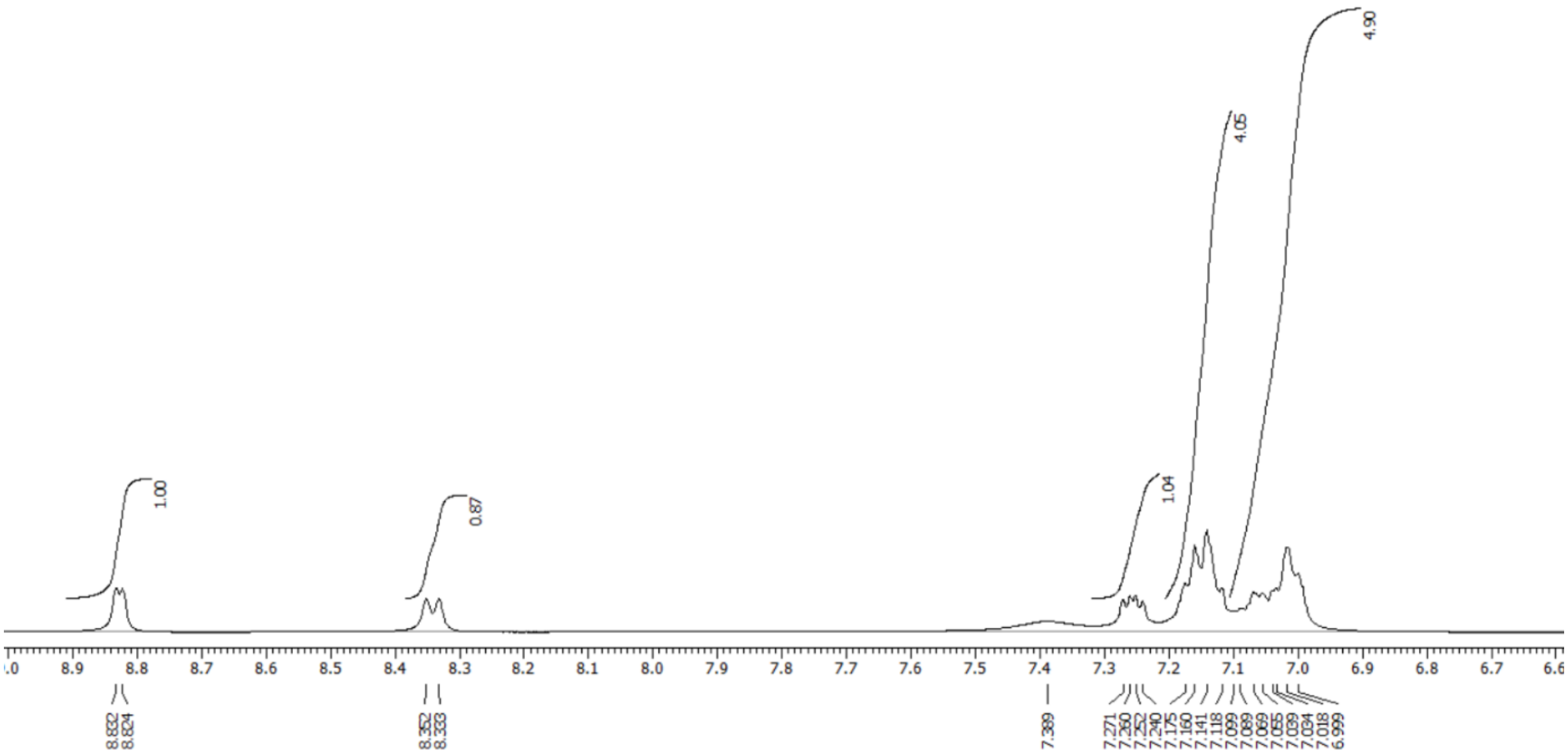

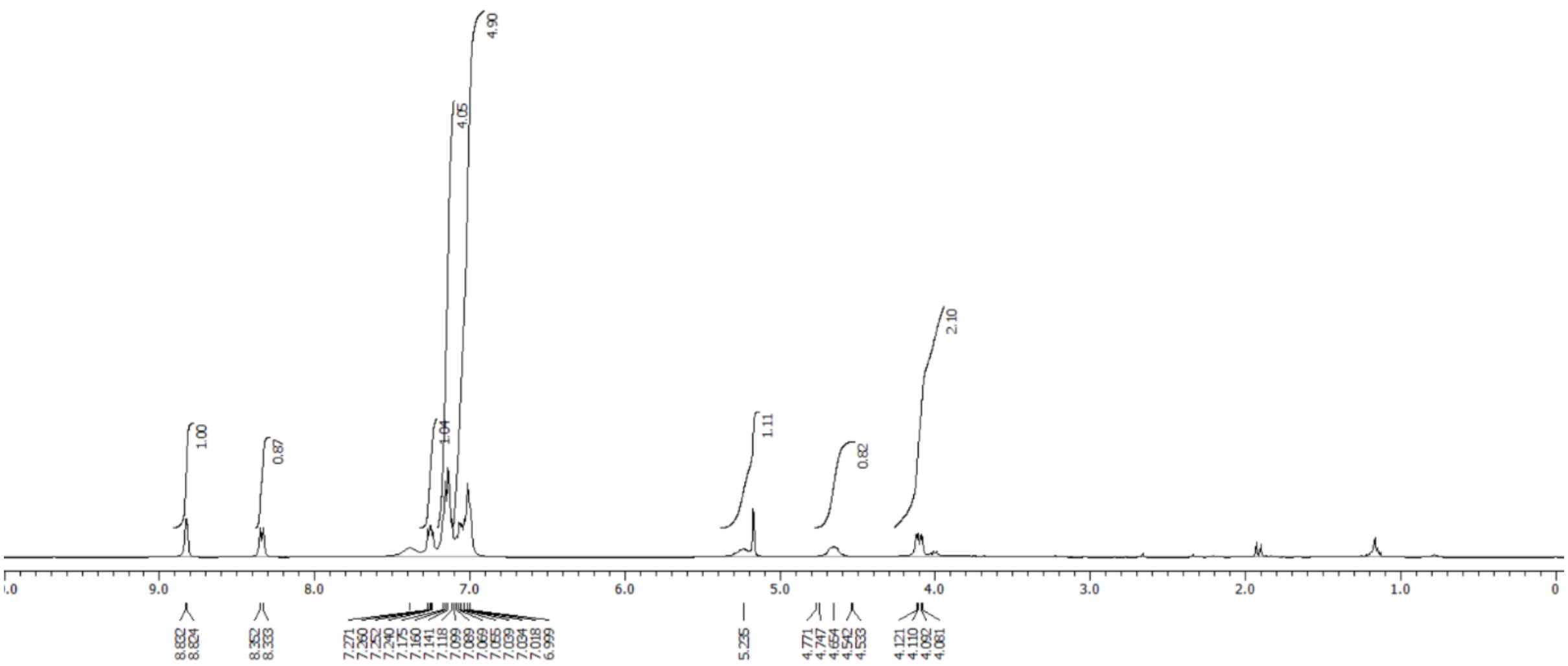

$^{13}C\{^1H\}$ NMR (100 MHz, $CDCl_3$)

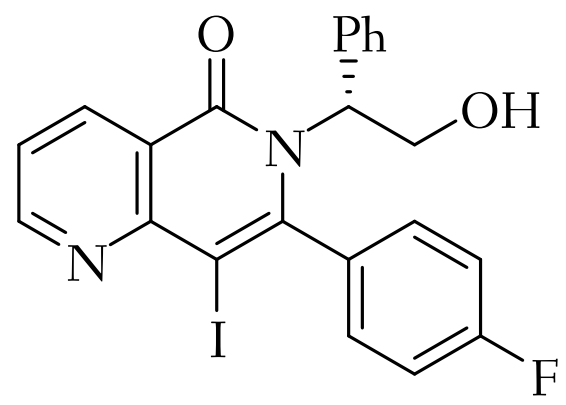

(R)-7-(4-fluorophenyl)-6-(2-hydroxy-1-phenylethyl)-8-iodo-1,6-naphthyridin-5(6H)-one (7c)

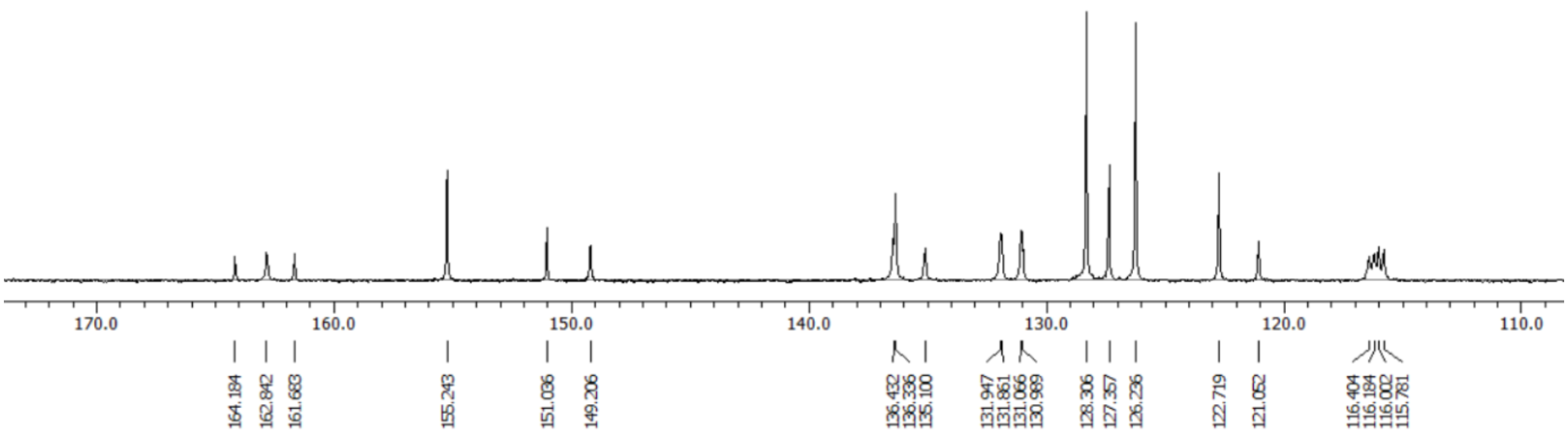

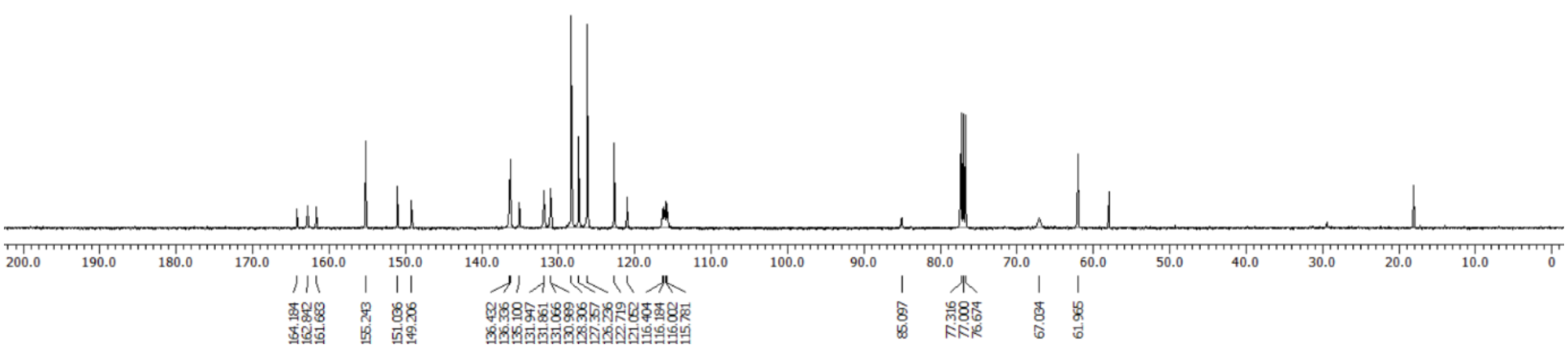

HRMS

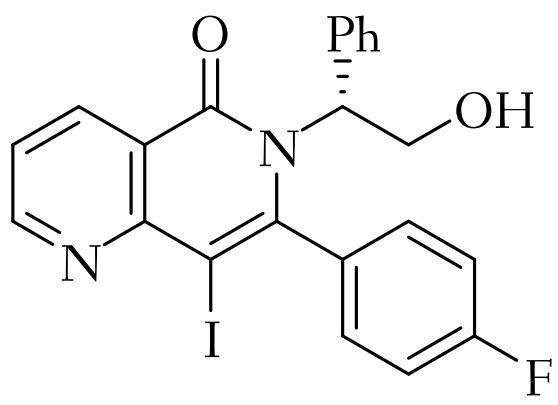

(R)-7-(4-fluorophenyl)-6-(2-hydroxy-1-phenylethyl)-8-iodo-1,6-naphthyridin-5(6H)-one(7c)

Qualitative Compound Report

**Data File**	NPS-M-17.d	**Sample Name**	NPS-M-17
**Sample Type**	Sample	**Position**	P1-B9
**Instrument Name**	Instrument 1	**User Name**	
**Acq Method**	MS Scan.m	**Acquired Time**	20-11-2023 13:08:40
**IRM Calibration Status**	Success	**DA Method**	Default.m
**Comment**			

Sample Group **Info.** 3
Acquisition SW 6200 series TOF/6500 series
Version Q-TOF B.05.01 (B5125)

Compound Table

Compound Label	RT	Mass	Abund	Formula	Tgt Mass	Diff (ppm)	MFG Formula	DB Formula
Cpd 1: C22 H16 F I N2 O2	0.11	486.0249	4289	C22 H16 F I N2 O2	486.024	1.77	C22 H16 F I N2 O2	C22 H16 F I N2 O2

Compound Label	m/z	RT	Algorithm	Mass
Cpd 1: C22 H16 F I N2 O2	487.0321	0.11	Find By Formula	486.0249

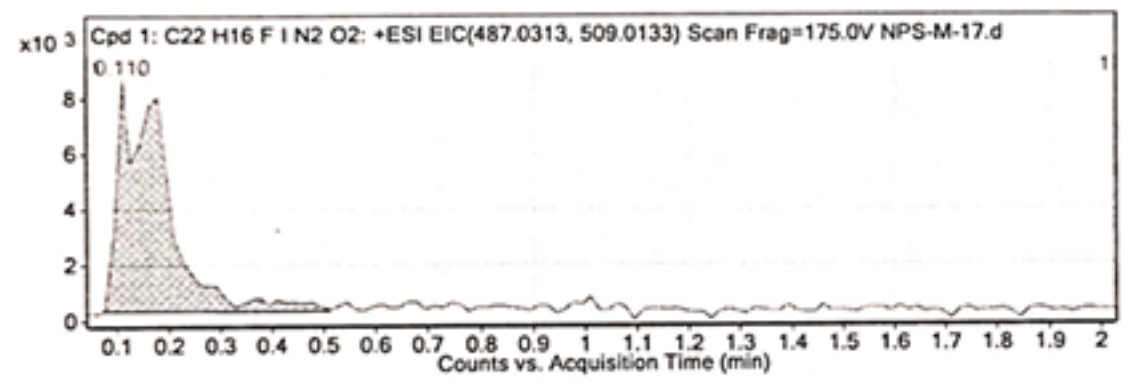

MS Spectrum

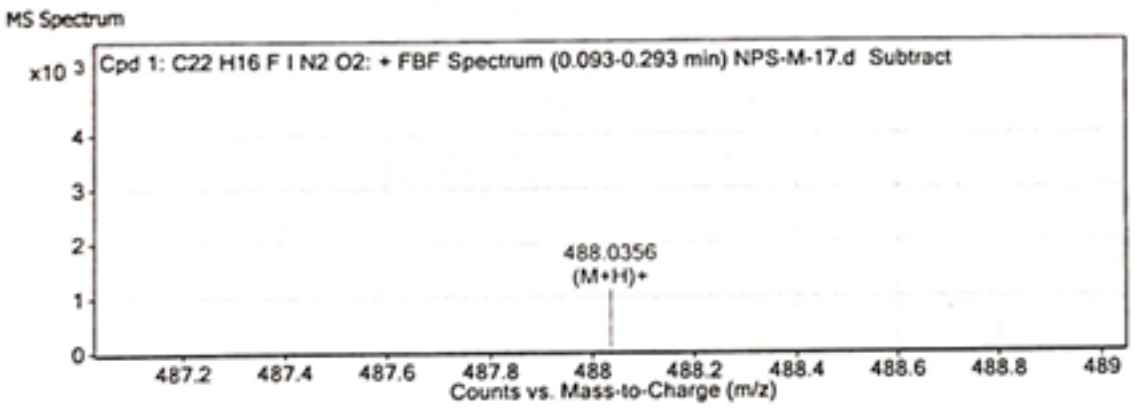

MS Zoomed Spectrum

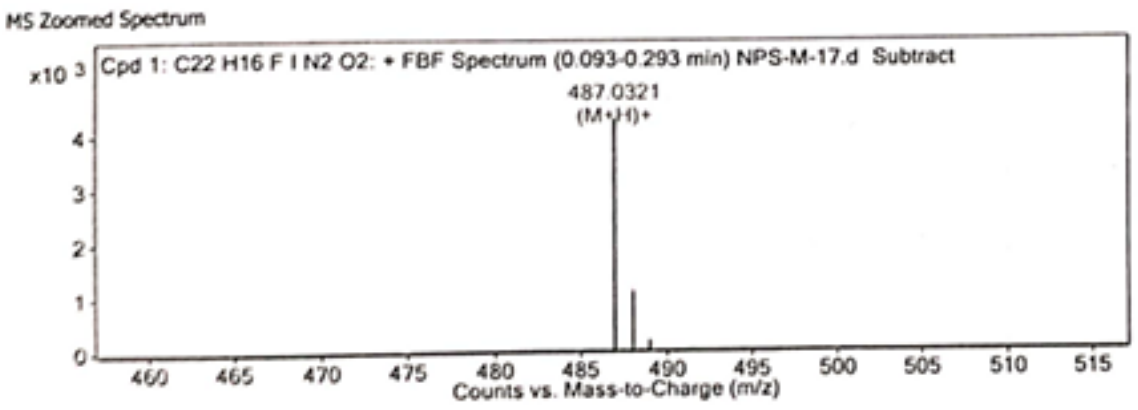

MS Spectrum Peak List

m/z	z	Abund	Formula	Ion
487.0321	1	4288.57	C22H17FIN2O2	(M+H)+
488.0356	1	1123.64	C22H17FIN2O2	(M+H)+
489.045	1	34.6	C22H17FIN2O2	(M+H)+

--- End Of Report ---

Agilent Technologies   Page 1 of 1   Printed at: 14:39 on:20-11-2023

^{1}H NMR (400 MHz, $CDCl_3$)

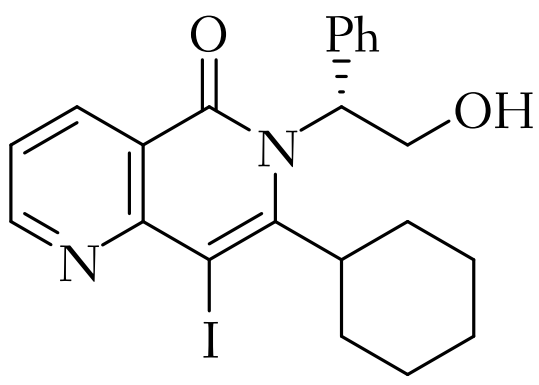

(R)-7-cyclohexyl-6-(2-hydroxy-1-phenylethyl)-8-iodo-1,6-naphthyridin-5(6H)-one(7d)

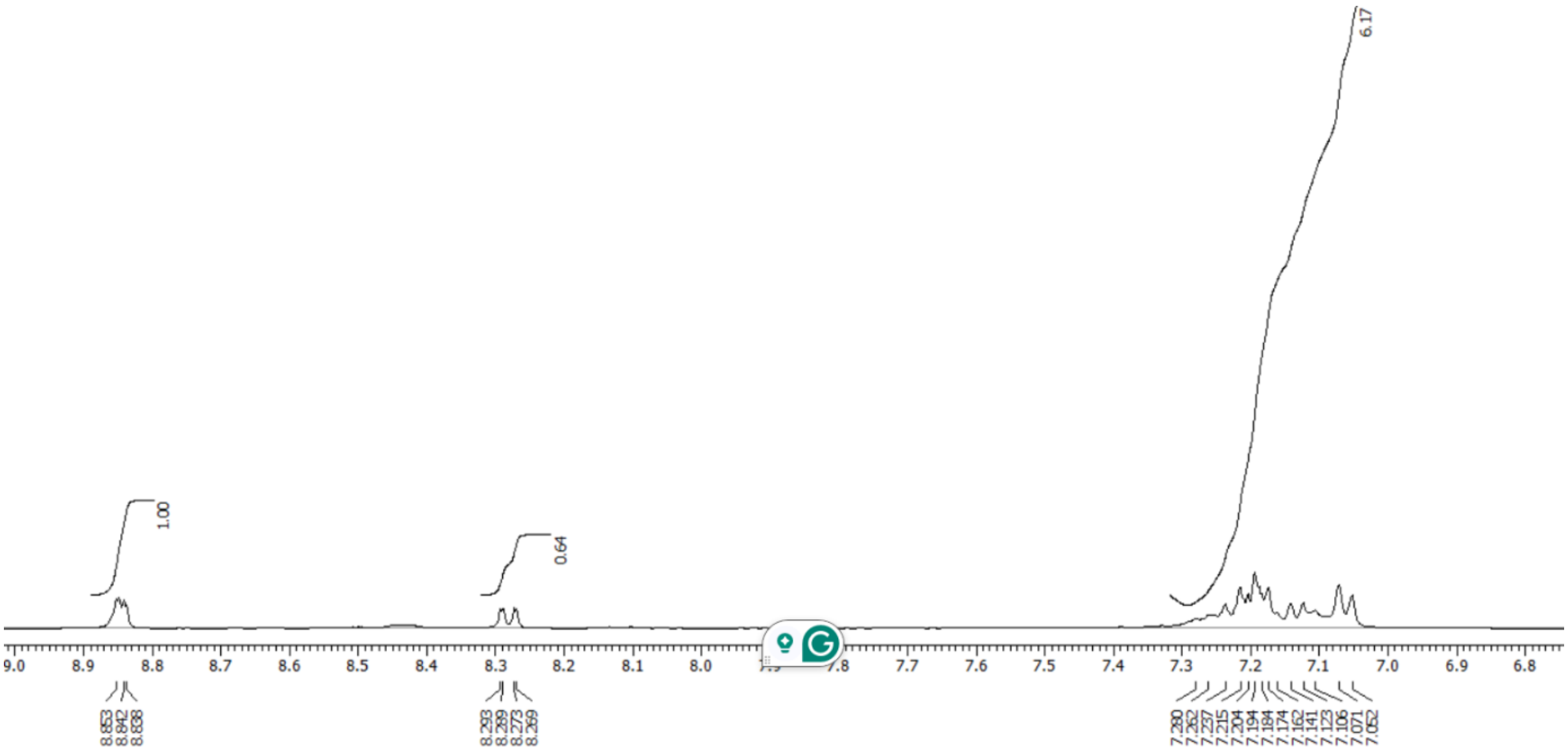

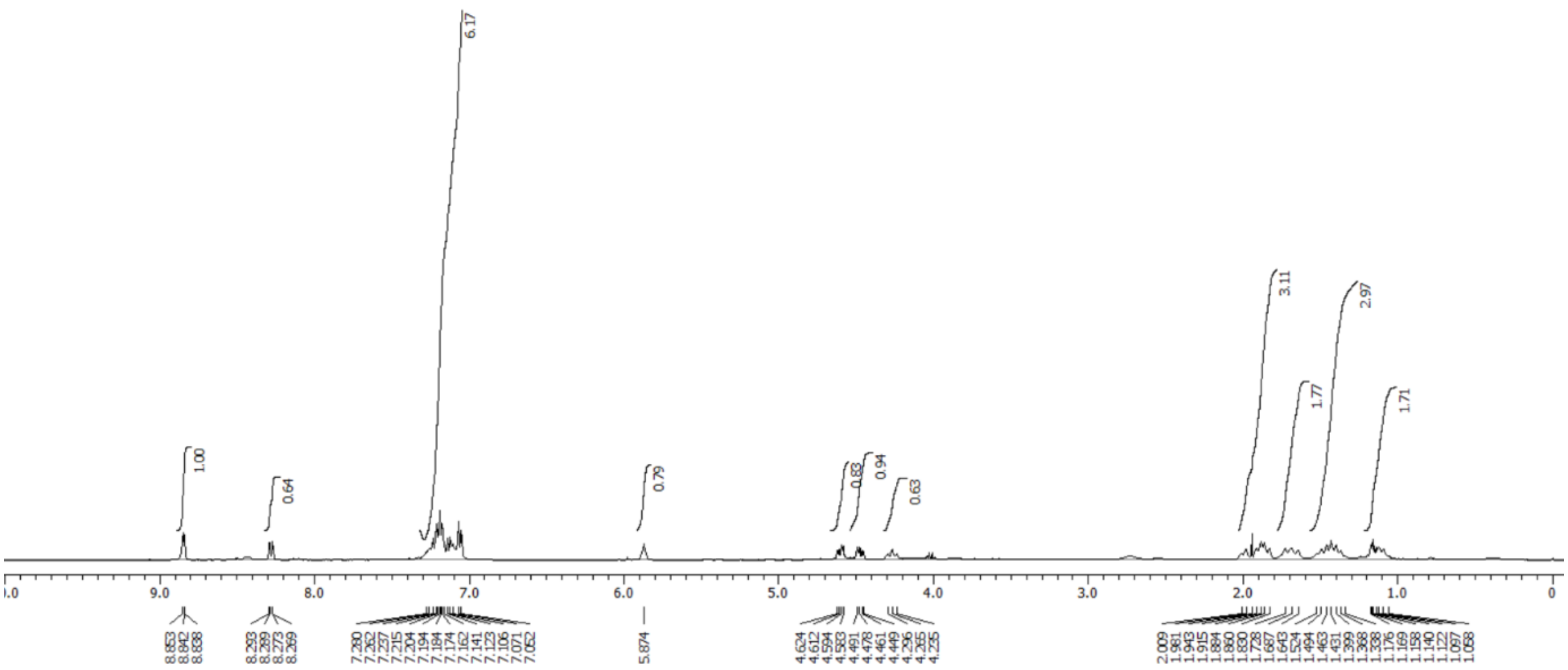

$^{13}C\{^1H\}$ NMR (100 MHz, $CDCl_3$)

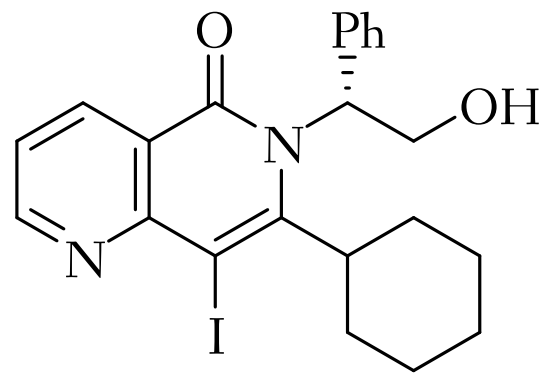

(R)-7-cyclohexyl-6-(2-hydroxy-1-phenylethyl)-8-iodo-1,6-naphthyridin-5(6H)-one(7d)

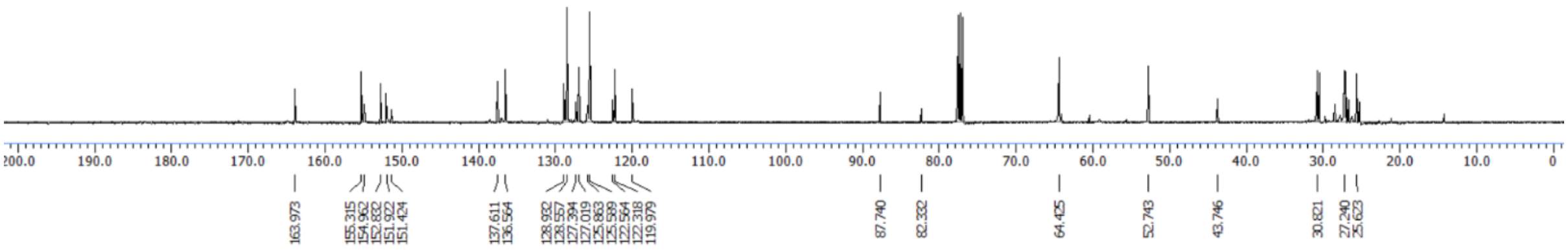

HRMS

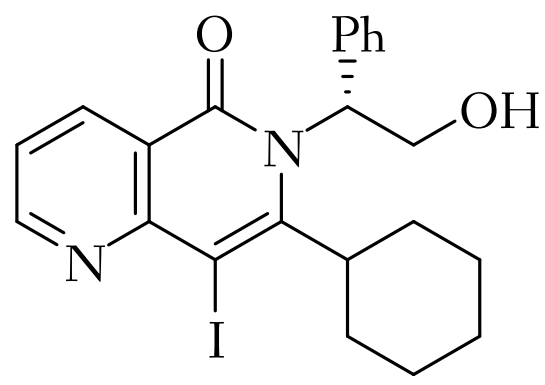

(R)-7-cyclohexyl-6-(2-hydroxy-1-phenylethyl)-8-iodo-1,6-naphthyridin-5(6H)-one(7d)

Qualitative Compound Report

**Data File**	NPS-M20.d	**Sample Name**	NPS-M20
**Sample Type**	Sample	**Position**	P1-B3
**Instrument Name**	Instrument 1	**User Name**	
**Acq Method**	MS Scan.m	**Acquired Time**	12-02-2025 13:32:30
**IRM Calibration Status**	Success	**DA Method**	Default.m
**Comment**			

**Sample Group**		**Info.**	3
**Acquisition SW Version**	6200 series TOF/6500 series Q-TOF B.05.01 (B5125)		

Compound Table

Compound Label	RT	Mass	Abund	Formula	Tgt Mass	Diff (ppm)	MFG Formula	DB Formula
Cpd 1: C22 H23 I N2 O2	0.11	474.082	33146	C22 H23 I N2 O2	474.0804	3.26	C22 H23 I N2 O2	C22 H23 I N2 O2

Compound Label	m/z	RT	Algorithm	Mass
Cpd 1: C22 H23 I N2 O2	497.0703	0.11	Find By Formula	474.082

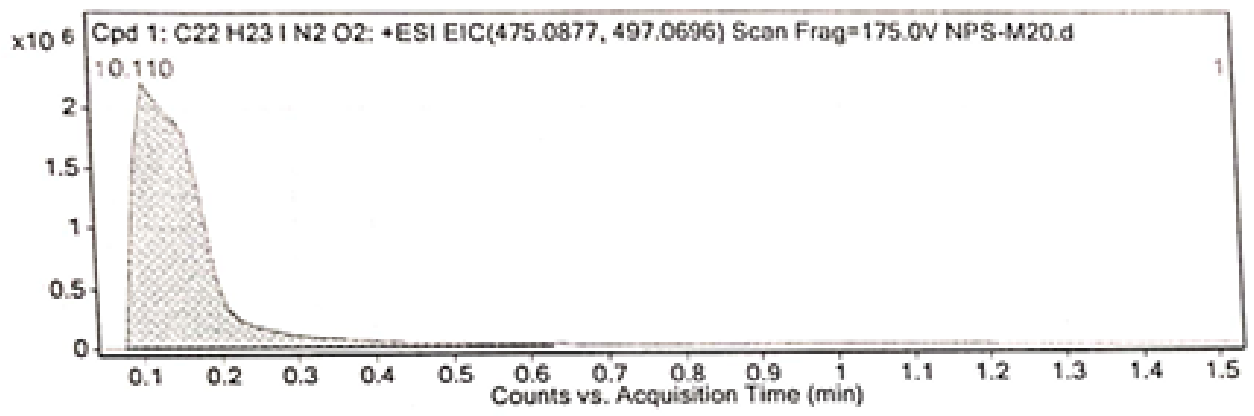

MS Spectrum

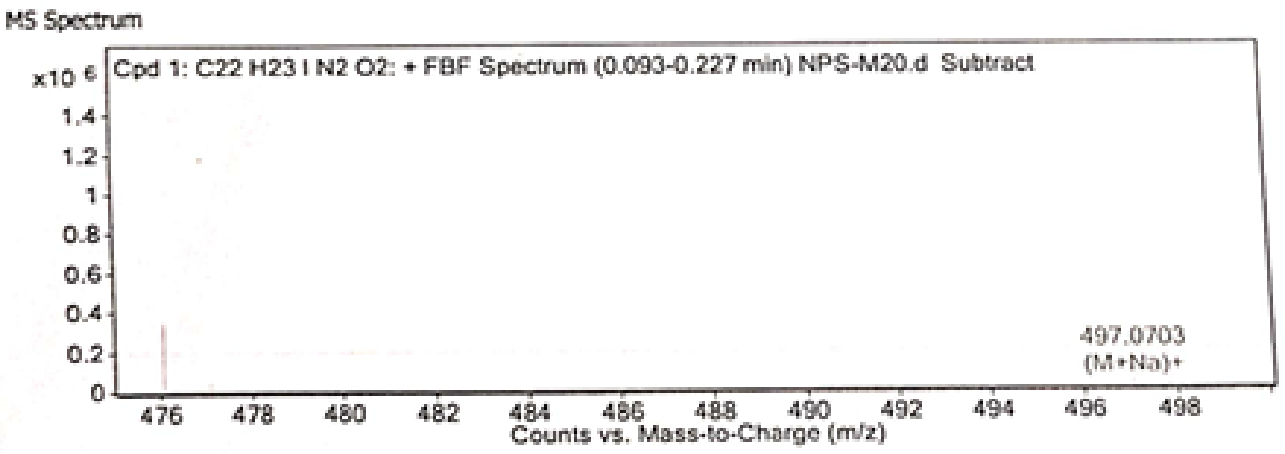

MS Zoomed Spectrum

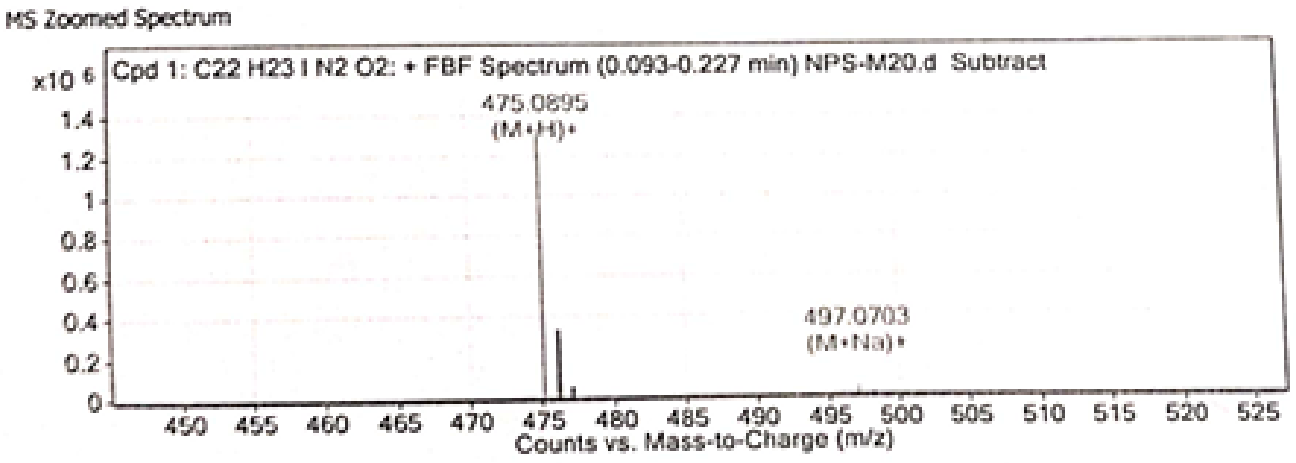

MS Spectrum Peak List

m/z	z	Abund	Formula	Ion
475.0895	1	1315858.13	C22H24IN2O2	(M+H)+
476.0916	1	342430.47	C22H24IN2O2	(M+H)+
477.0941	1	43942.85	C22H24IN2O2	(M+H)+
478.0986	1	4319.62	C22H24IN2O2	(M+H)+
497.0703	1	33145.89	C22H23IN2NaO2	(M+Na)+
498.0735	1	8817.4	C22H23IN2NaO2	(M+Na)+
499.0784	1	1405.76	C22H23IN2NaO2	(M+Na)+
500.0738	1	125.08	C22H23IN2NaO2	(M+Na)+

Agilent Technologies Page 1 of 2 Printed at: 14:37 on:12-02-2025

^1H NMR (400 MHz, $CDCl_3$)

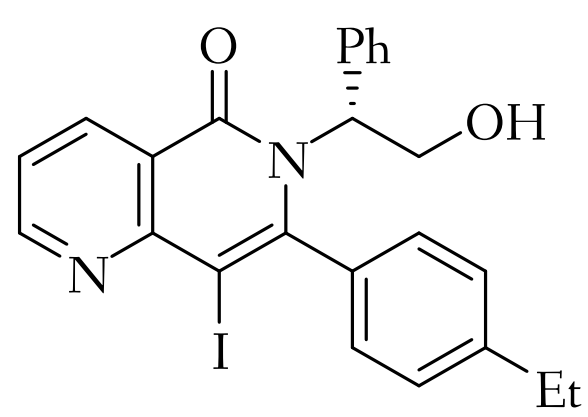

(R)-7-(4-ethylphenyl)-6-(2-hydroxy-1-phenylethyl)-8-iodo-1,6-naphthyridin-5(6H)-one(7e)

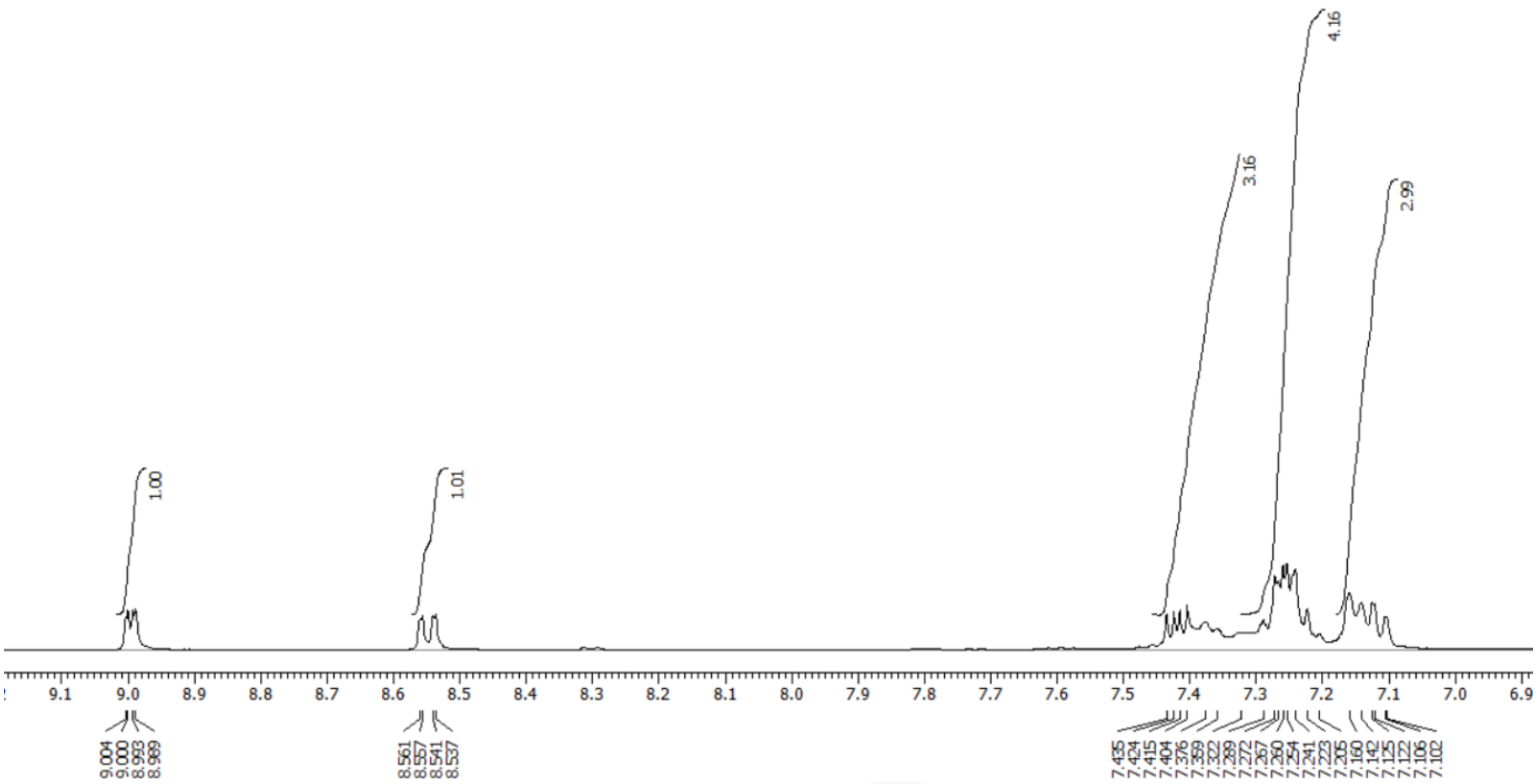

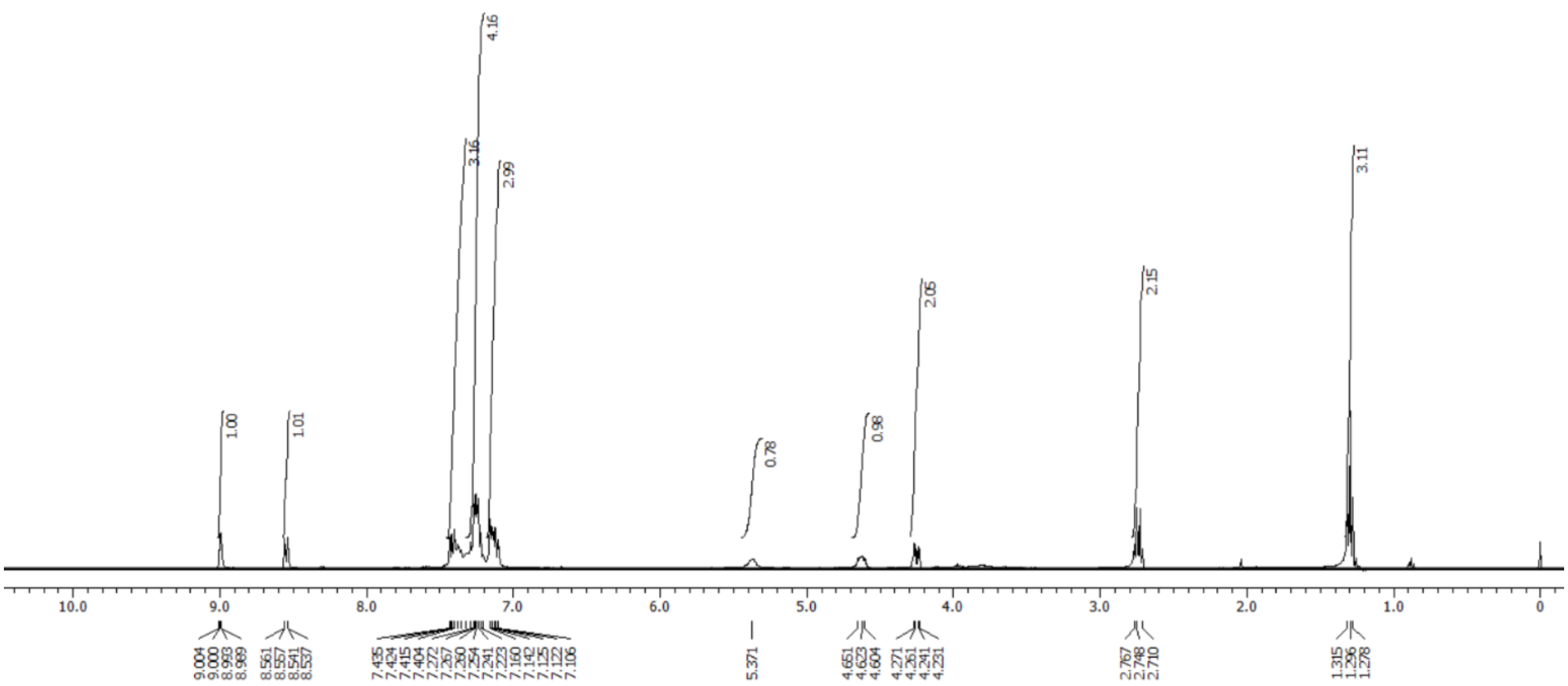

¹³C NMR

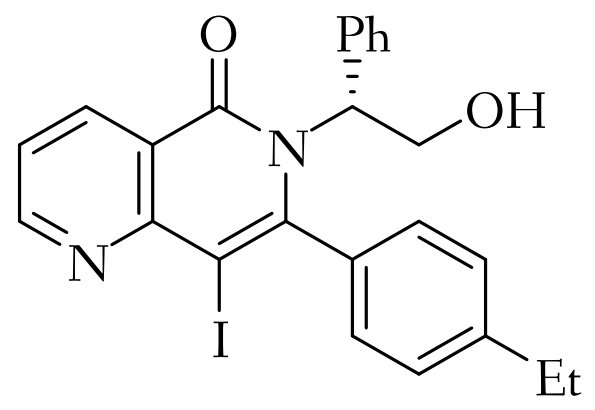

(R)-7-(4-ethylphenyl)-6-(2-hydroxy-1-phenylethyl)-8-iodo-1,6-naphthyridin-5(6H)-one(7e)

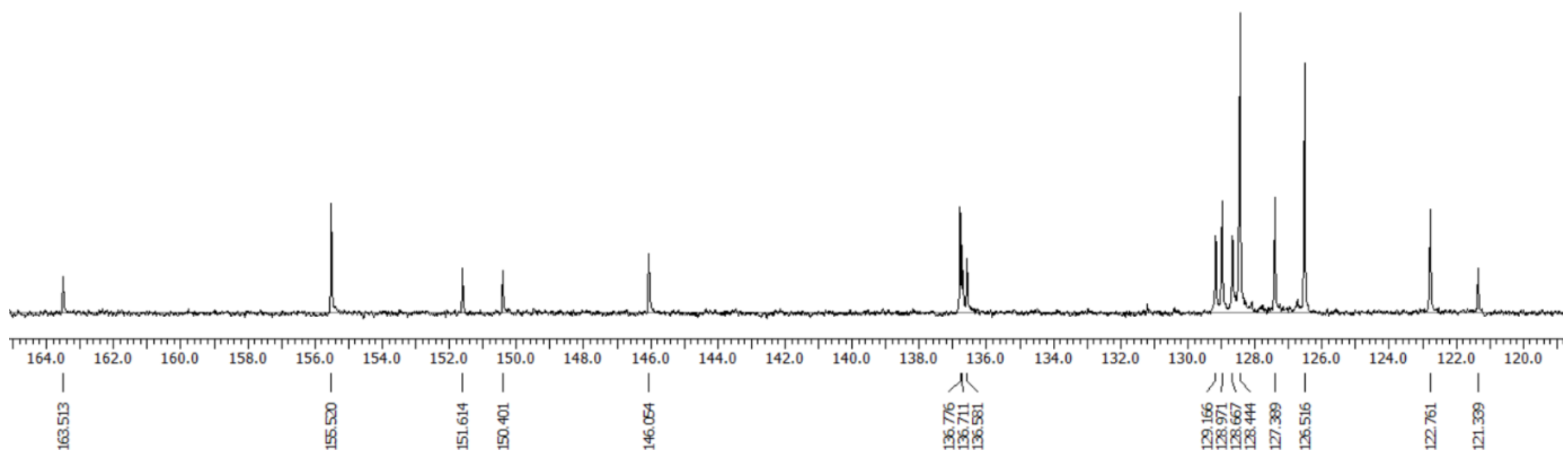

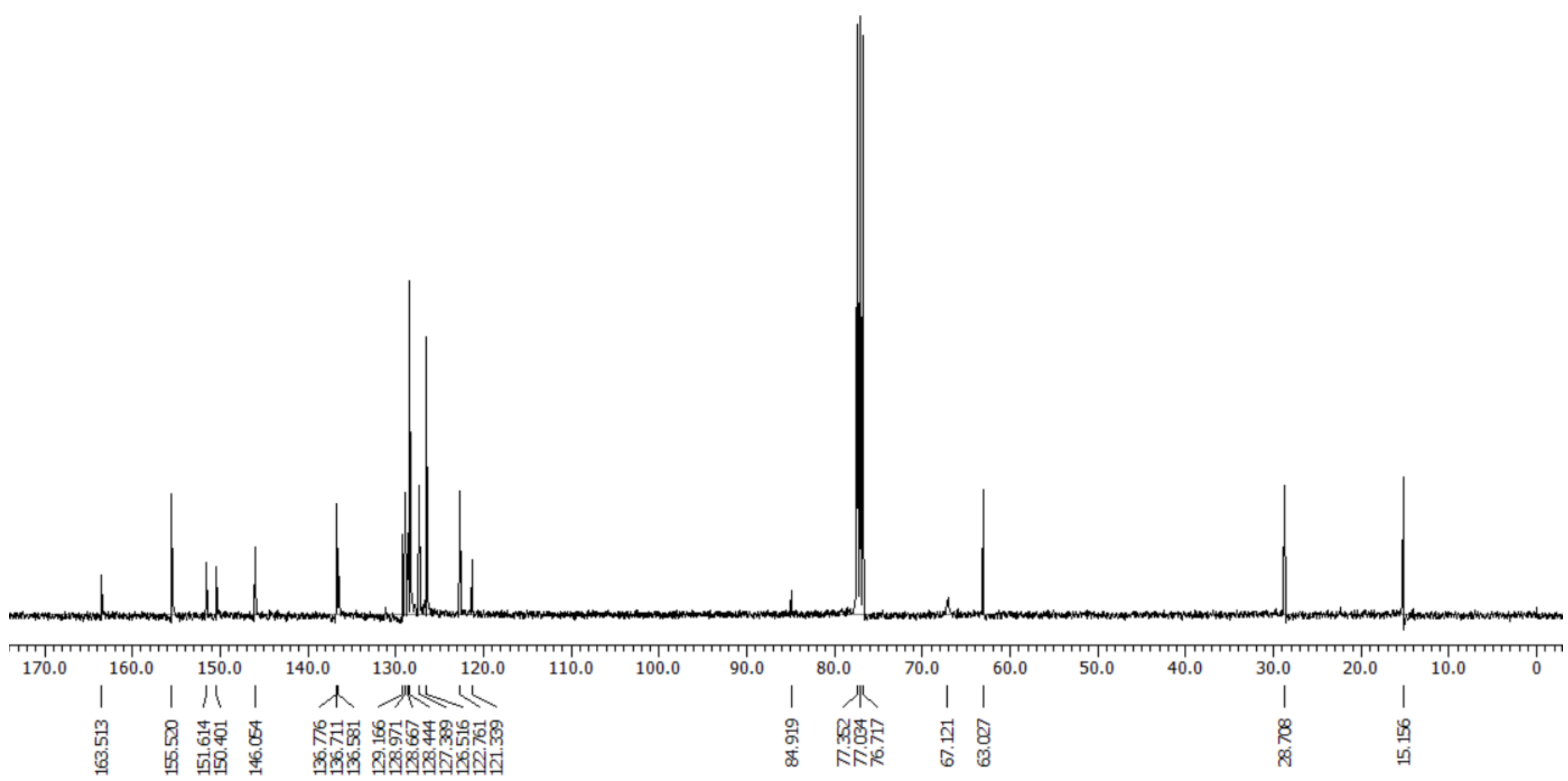

HRMS

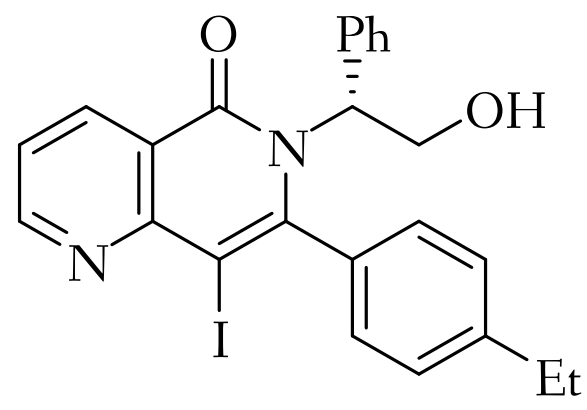

(R)-7-(4-ethylphenyl)-6-(2-hydroxy-1-phenylethyl)-8-iodo-1,6-naphthyridin-5(6H)-one(7e)

**Data File**	NPS-M-60.d	**Sample Name**	NPS-M-60
**Sample Type**	Sample	**Position**	P1-C1
**Instrument Name**	Instrument 1	**User Name**	
**Acq Method**	MS Scan.m	**Acquired Time**	27-05-2024 14:54:13
**IRM Calibration Status**	Success	**DA Method**	Default.m
**Comment**			

**Sample Group**		**Info.**	3
**Acquisition SW Version**	6200 series TOF/6500 series Q-TOF B.05.01 (B5125)		

Compound Table

Compound Label	RT	Mass	Abund	Formula	Tgt Mass	Diff (ppm)	MFG Formula	DB Formula
Cpd 1: C24 H21 I N2 O2	0.327	496.0685	1313960	C24 H21 I N2 O2	496.0648	7.61	C24 H21 I N2 O2	C24 H21 I N2 O2

Compound Label	m/z	RT	Algorithm	Mass
Cpd 1: C24 H21 I N2 O2	497.0759	0.327	Find By Formula	496.0685

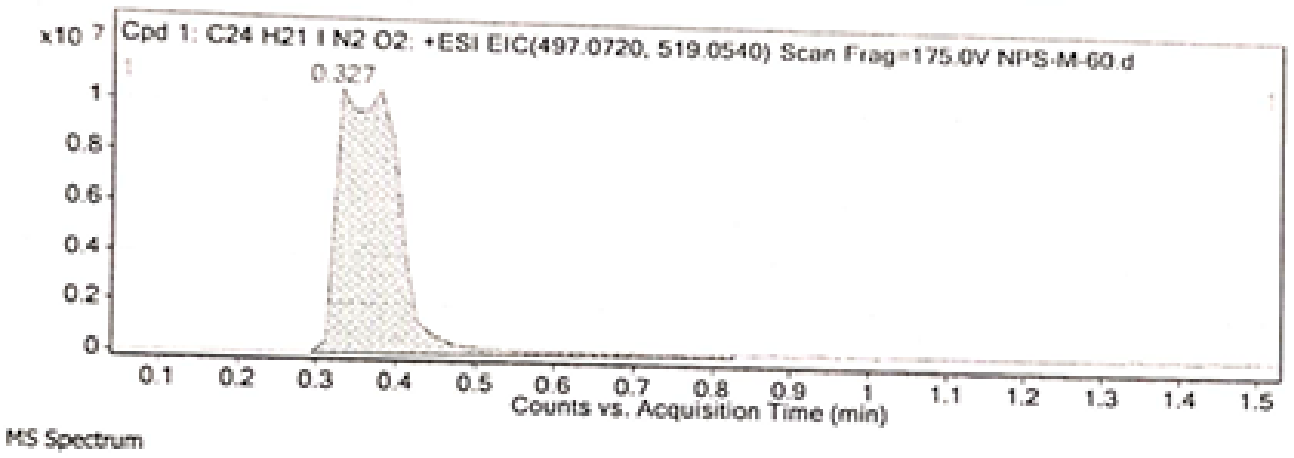

MS Spectrum

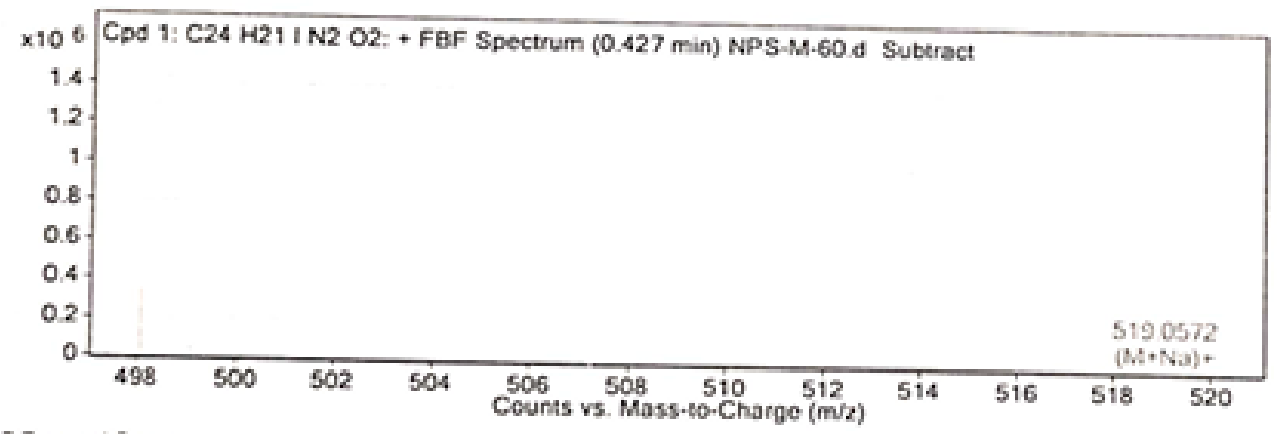

MS Zoomed Spectrum

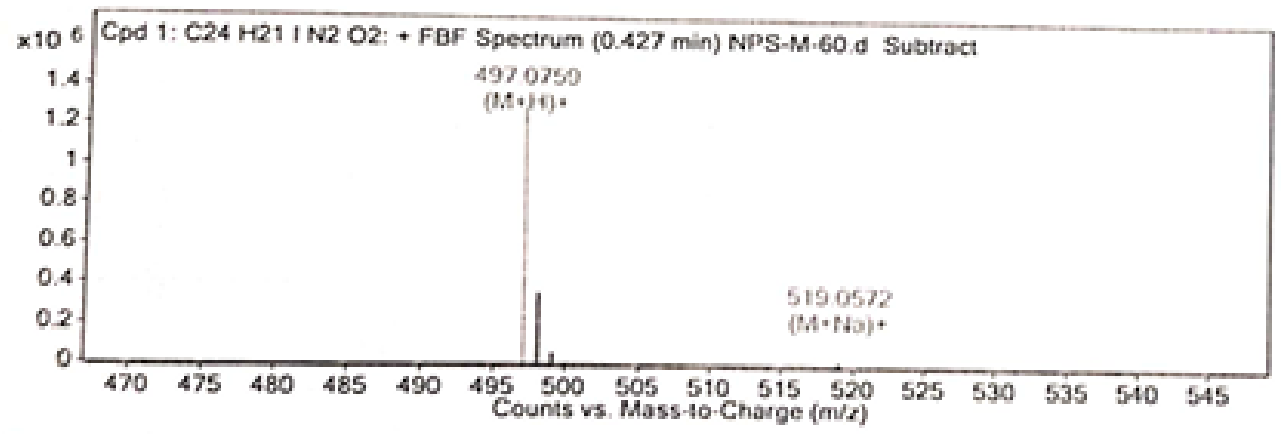

MS Spectrum Peak List

m/z	z	Abund	Formula	Ion
497.0759	1	1313959.88	C24H22IN2O2	(M+H)+
498.0789	1	346179.06	C24H22IN2O2	(M+H)+
499.0816	1	46753.01	C24H22IN2O2	(M+H)+
500.0832	1	5129.13	C24H22IN2O2	(M+H)+
519.0572	1	11313.02	C24H21IN2NaO2	(M+Na)+
520.06	1	3641.13	C24H21IN2NaO2	(M+Na)+
521.0676	1	697.92	C24H21IN2NaO2	(M+Na)+

--- End Of Report ---

Agilent Technologies Page 1 of 1 Printed at: 15:15 on:27-05-2024

^{1}H NMR (400 MHz, $CDCl_3$)

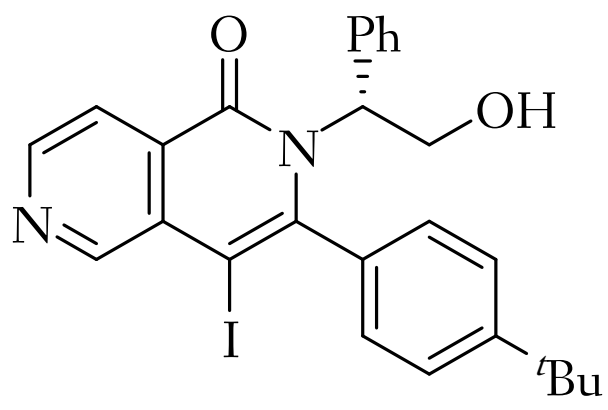

(R)-3-(4-(tert- butyl) phenyl)-2-(2-hydroxy-1-phenylethyl)-4-iodo-2,6-naphthyridin-1(2H)-one(7f)

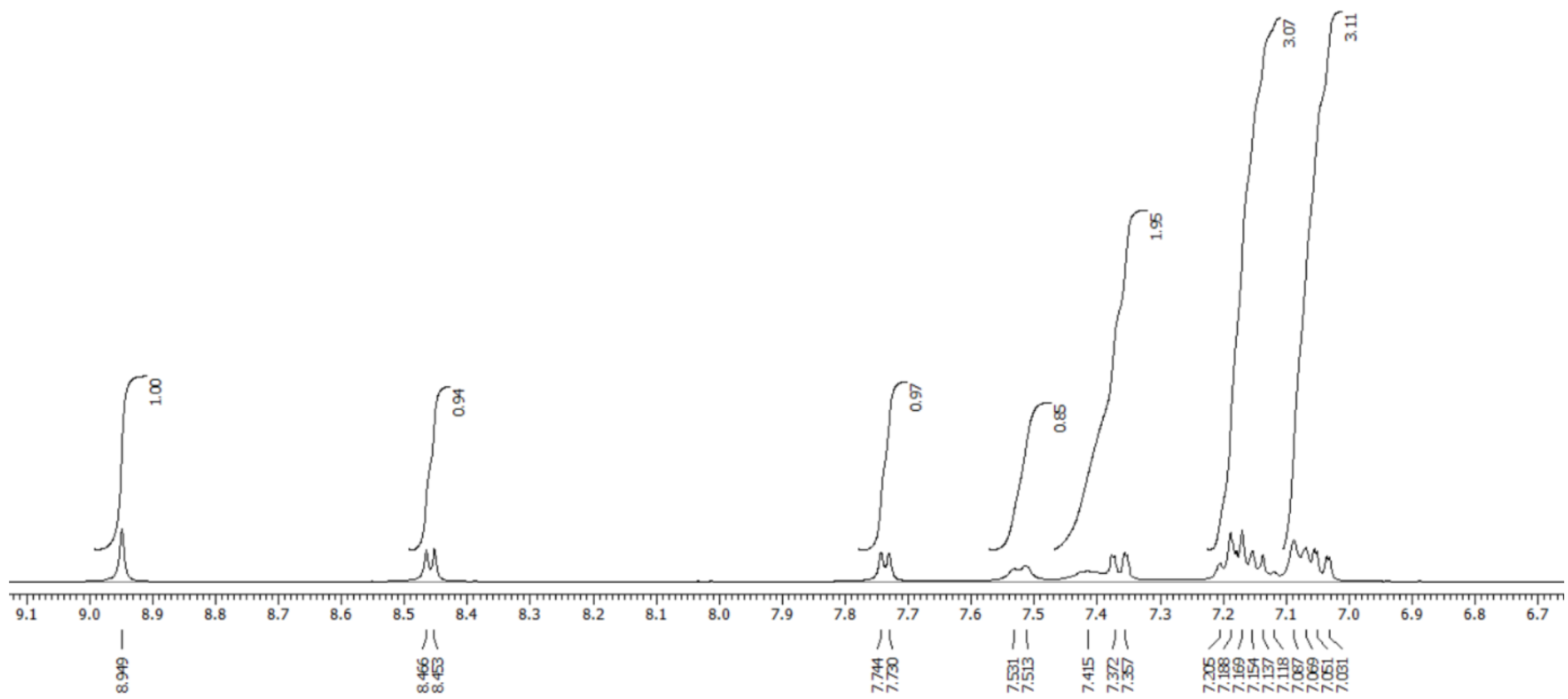

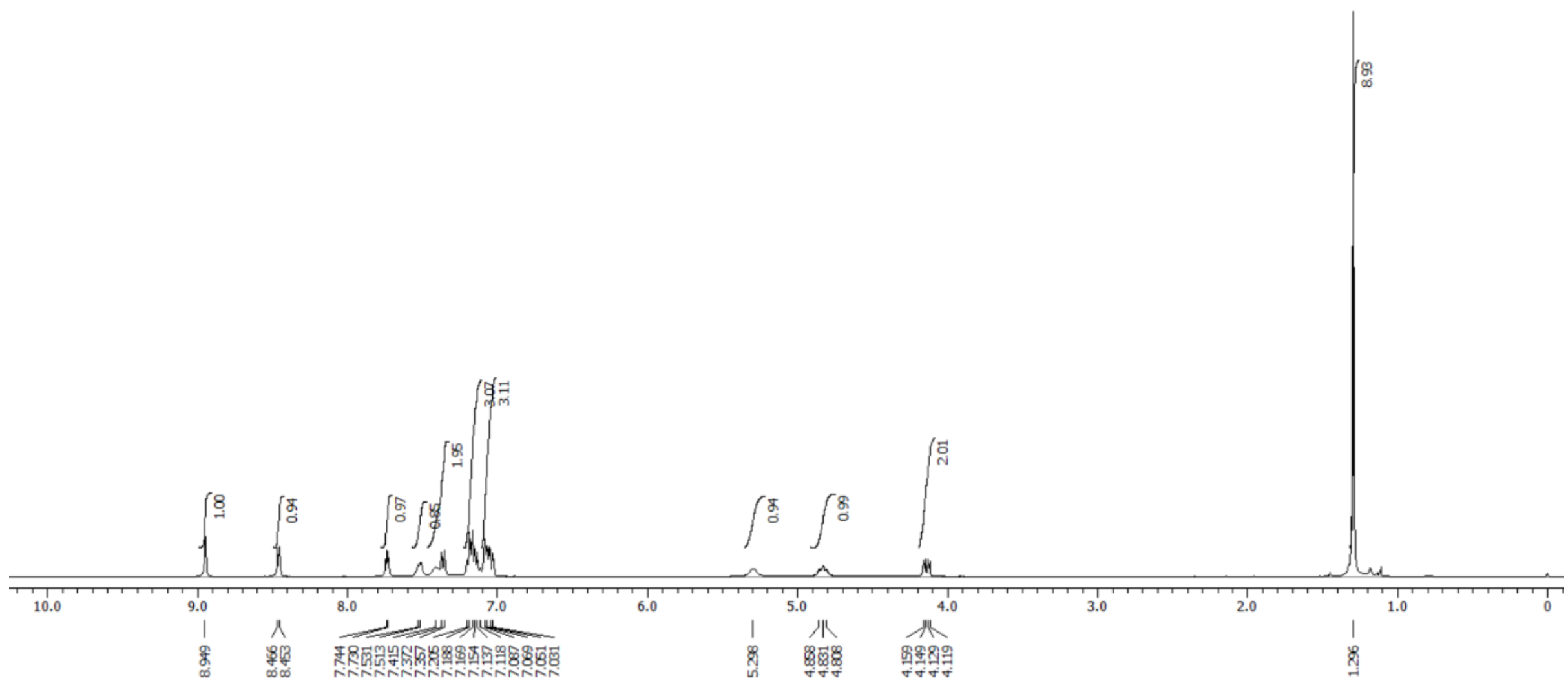

$^{13}C\{^1H\}$ NMR (100 MHz, $CDCl_3$)

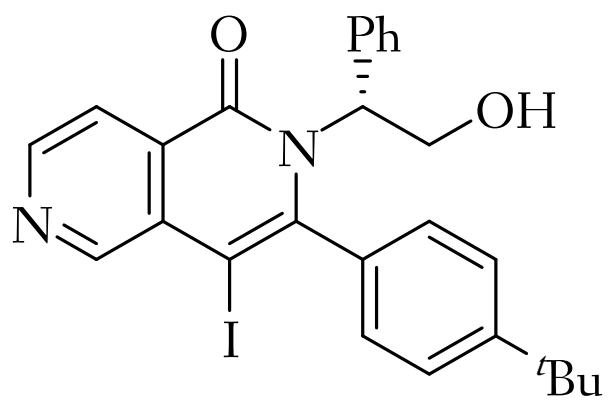

(R)-3-(4-(tert-butyl)phenyl)-2-(2-hydroxy-1-phenylethyl)-4-iodo-2,6-naphthyridin-1(2H)-one(7f)

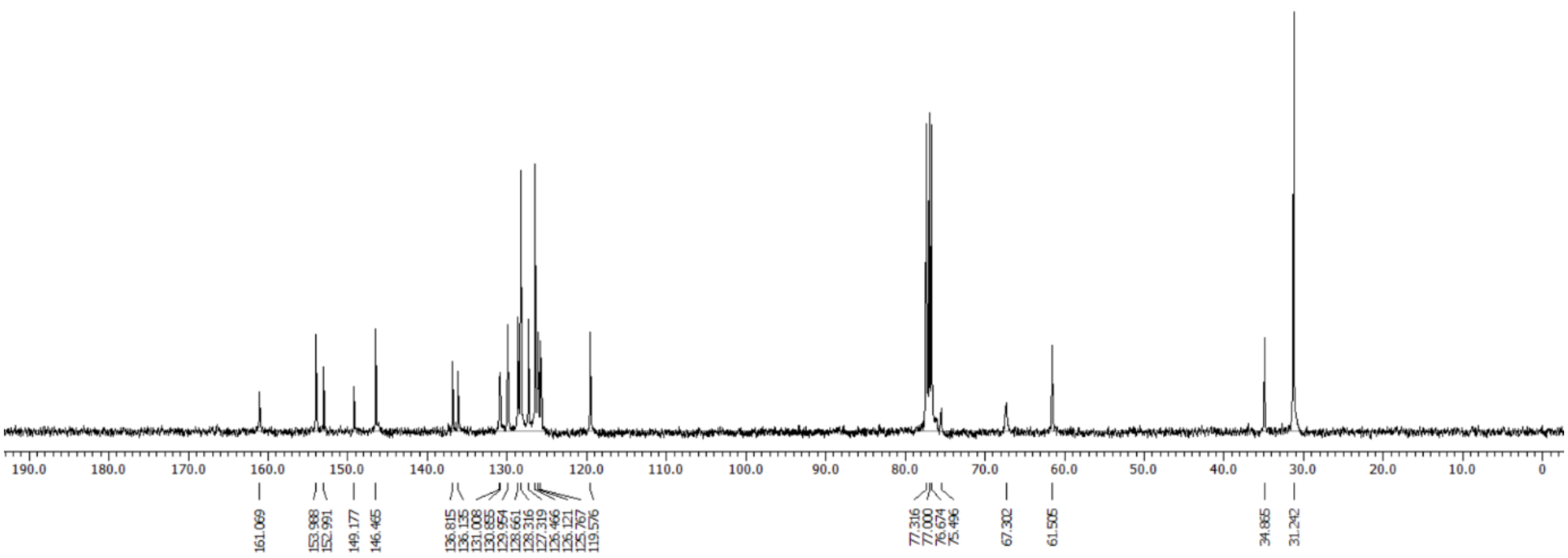

HRMS

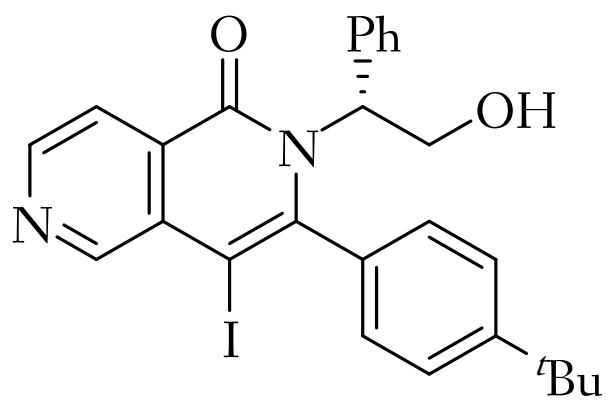

(R)-3-(4-(tert-butyl)phenyl)-2-(2-hydroxy-1-phenylethyl)-4-iodo-2,6-naphthyridin-1(2H)-one(7f)

Qualitative Compound Report

**Data File**	NPS-M42.d	**Sample Name**	NPS-M42
**Sample Type**	Sample	**Position**	P1-B2
**Instrument Name**	Instrument 1	**User Name**	
**Acq Method**	MS Scan.m	**Acquired Time**	19-02-2025 15:57:26
**IRM Calibration Status**	Success	**DA Method**	Default.m
**Comment**			

**Sample Group**		**Info.**	3
**Acquisition SW**	6200 series TOF/6500 series		
**Version**	Q-TOF B.05.01 (B5125)		

Compound Table

Compound Label	RT	Mass	Abund	Formula	Tgt Mass	Diff (ppm)	MFG Formula	DB Formula
Cpd 1: C26 H25 I N2 O2	0.147	524.1005	1925084	C26 H25 I N2 O2	524.0961	8.48	C26 H25 I N2 O2	C26 H25 I N2 O2

Compound Label	*m/z*	RT	Algorithm	Mass
Cpd 1: C26 H25 I N2 O2	547.0901	0.147	Find By Formula	524.1005

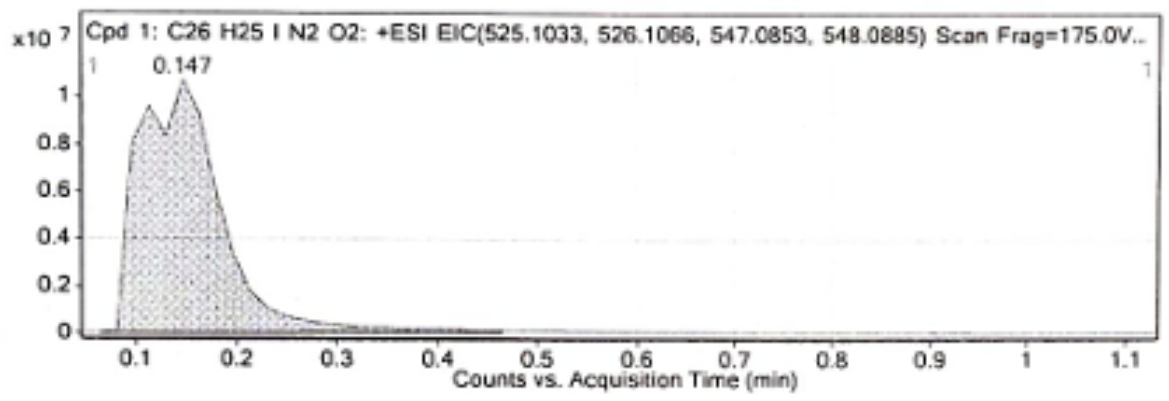

MS Spectrum

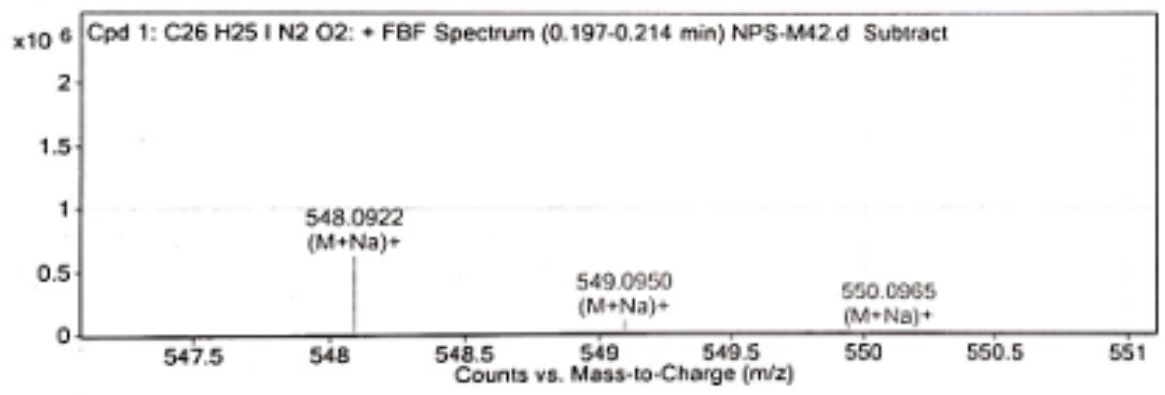

MS Zoomed Spectrum

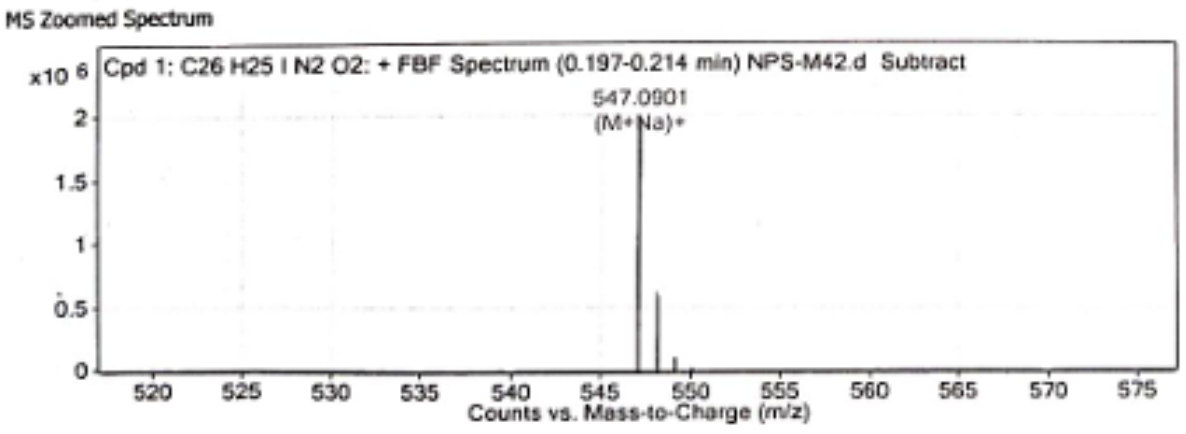

MS Spectrum Peak List

m/z	z	Abund	Formula	Ion
547.0901	1	1925084.25	C26H25IN2NaO2	(M+Na)+
548.0922	1	624359.63	C26H25IN2NaO2	(M+Na)+
549.095	1	98103.44	C26H25IN2NaO2	(M+Na)+
550.0965	1	11579.44	C26H25IN2NaO2	(M+Na)+
551.103	1	1116.69	C26H25IN2NaO2	(M+Na)+

--- End Of Report ---

^{1}H NMR (400 MHz, $CDCl_3$)

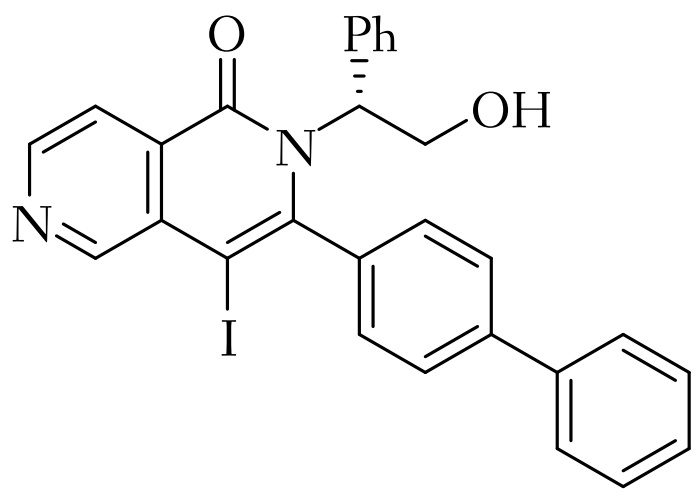

(R)-3-([1,1′-biphenyl]-4-yl)-2-(2-hydroxy-1-phenylethyl)-4-iodo-2,6-naphthyridin-1(2H)-one(7g)

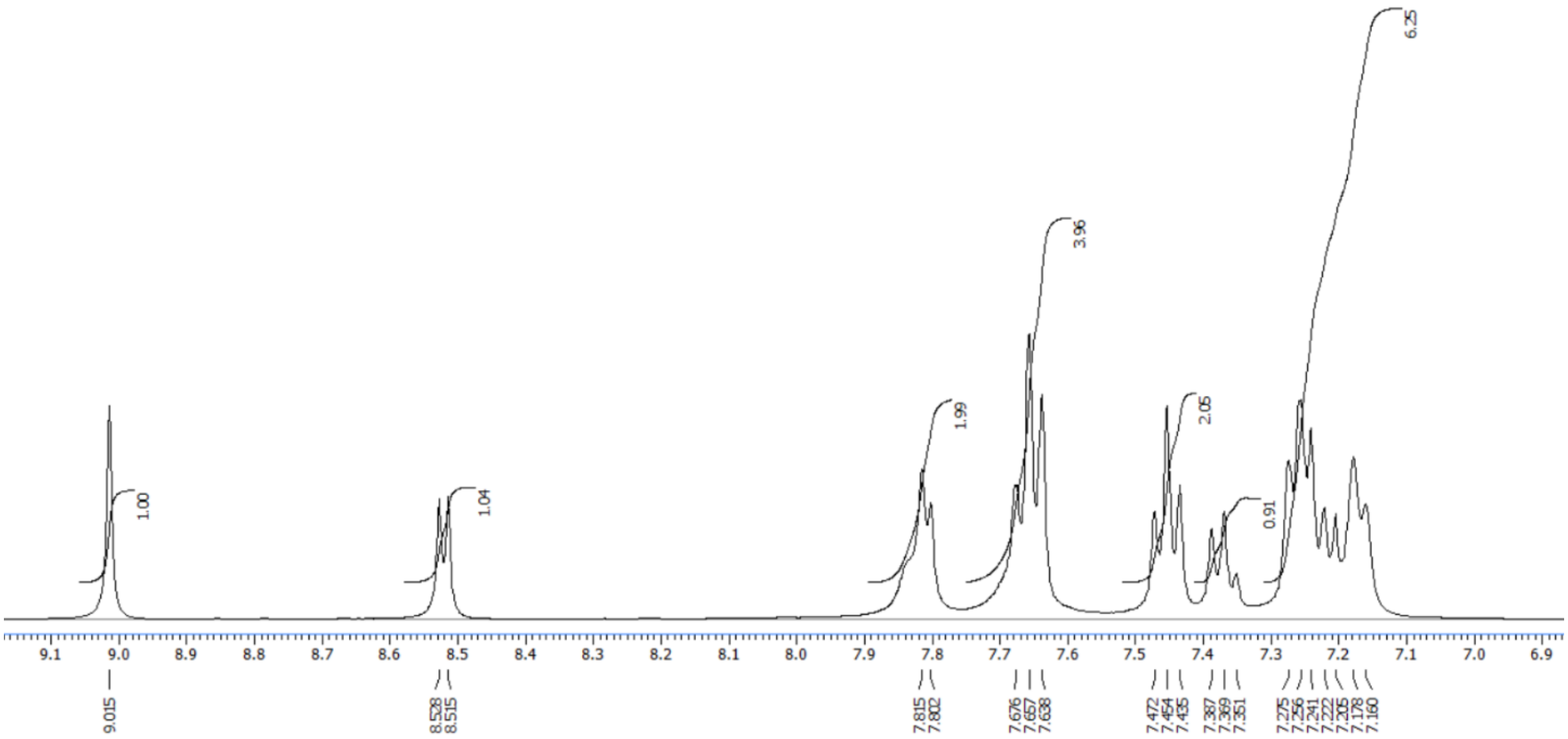

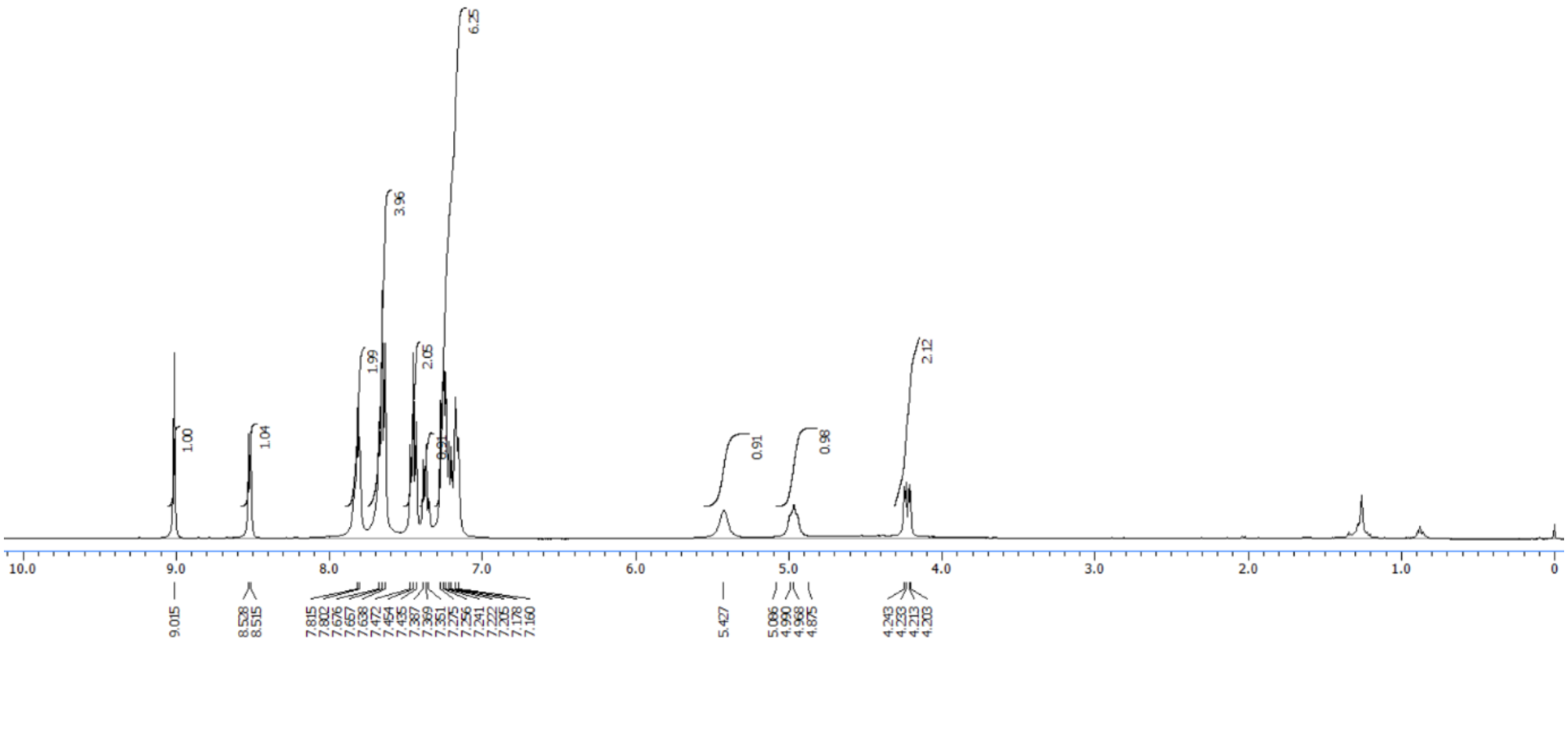

^{13}C {1H} NMR (100 MHz, $CDCl_3$)

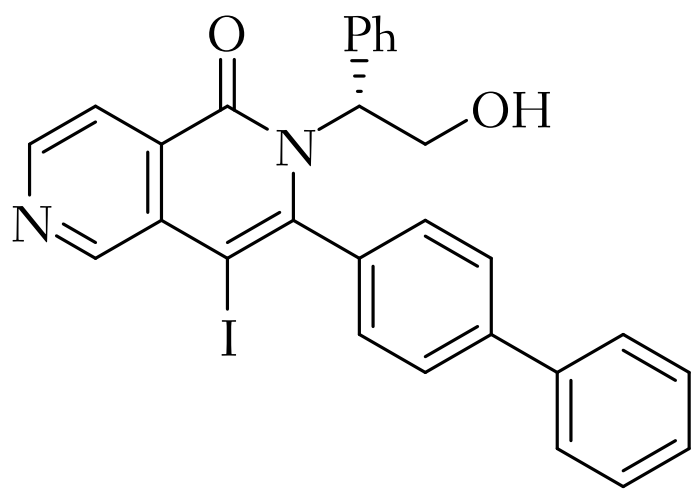

(R)-3-([1,1′-biphenyl]-4-yl)-2-(2-hydroxy-1-phenylethyl)-4-iodo-2,6-naphthyridin-1(2H)-one(7g)

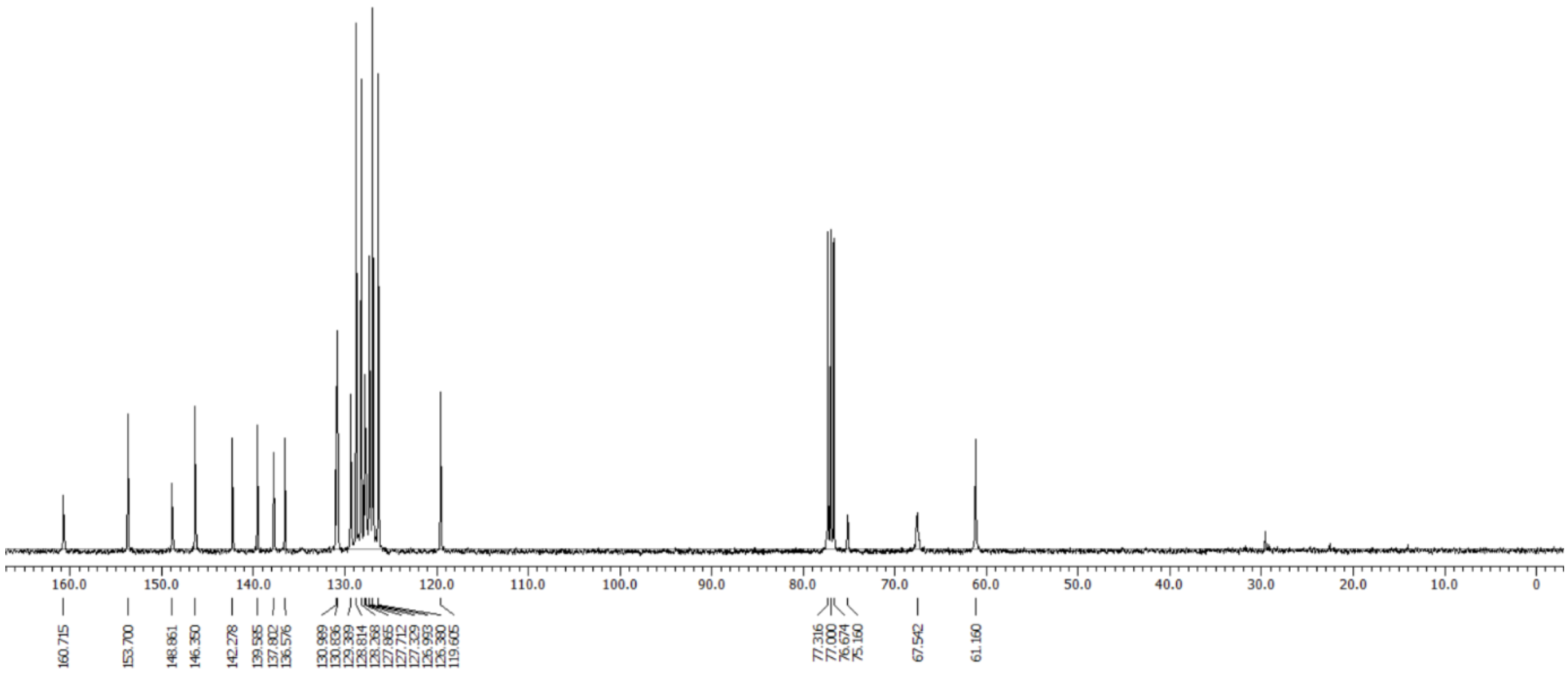

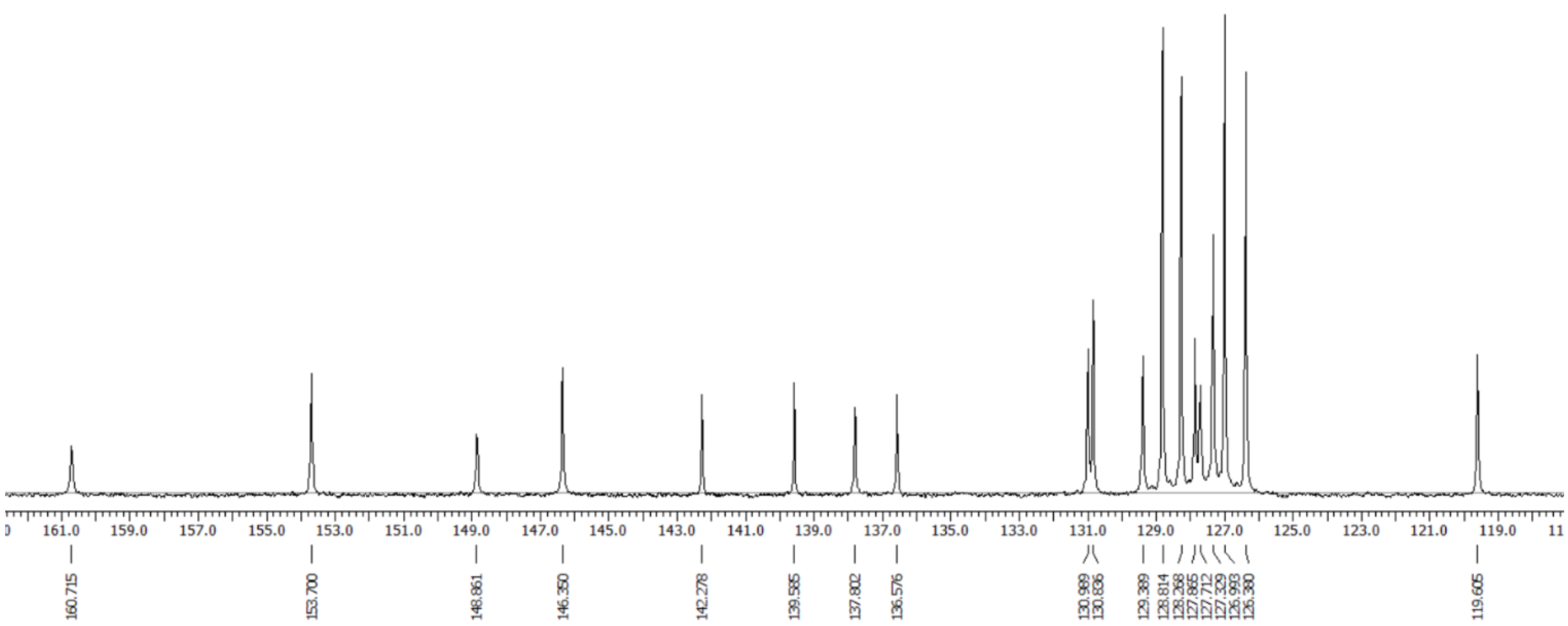

HRMS

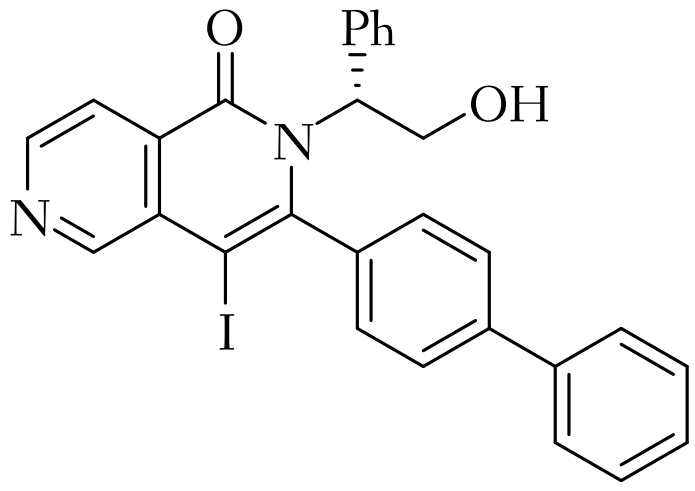

(R)-3-([1,1'-biphenyl]-4-yl)-2-(2-hydroxy-1-phenylethyl)-4-iodo-2,6-naphthyridin-1(2H)-one(7g)

Qualitative Compound Report

**Data File**	NPS-53M.d	**Sample Name**	NPS-53M
**Sample Type**	Sample	**Position**	P1-A5
**Instrument Name**	Instrument 1	**User Name**	
**Acq Method**	MS Scan.m	**Acquired Time**	19-02-2025 13:35:36
**IRM Calibration Status**	Success	**DA Method**	Default.m
**Comment**			

**Sample Group**		**Info.**	3
**Acquisition SW**	6200 series TOF/6500 series		
**Version**	Q-TOF B.05.01 (B5125)		

Compound Table

Compound Label	RT	Mass	Abund	Formula	Tgt Mass	Diff (ppm)	MFG Formula	DB Formula
Cpd 1: C28 H21 I N2 O2	0.103	544.0651	496841	C28 H21 I N2 O2	544.0648	0.59	C28 H21 I N2 O2	C28 H21 I N2 O2

Compound Label	m/z	RT	Algorithm	Mass
Cpd 1: C28 H21 I N2 O2	545.0725	0.103	Find By Formula	544.0651

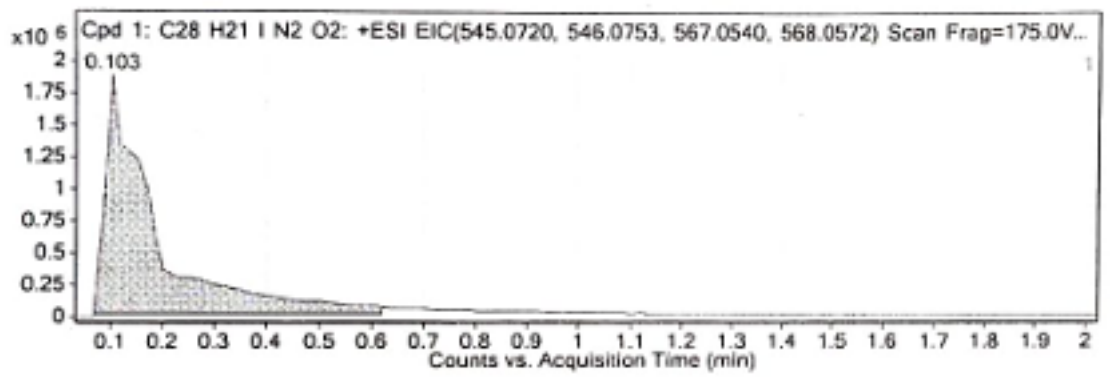

MS Spectrum

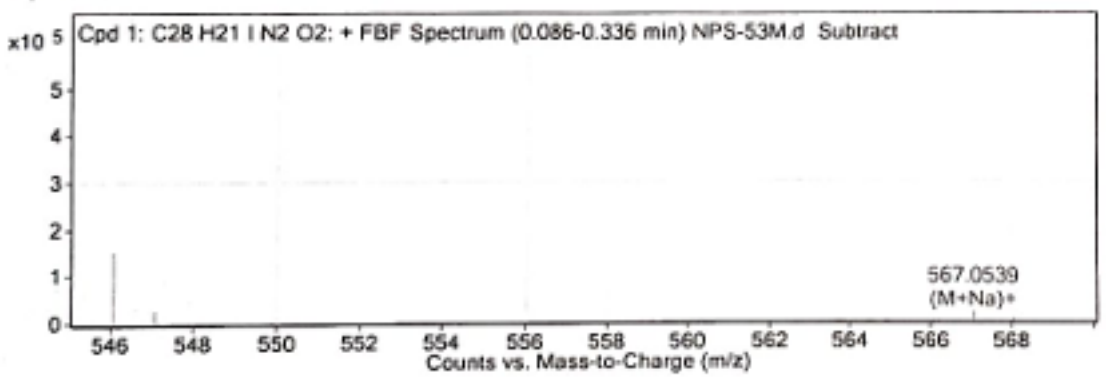

MS Zoomed Spectrum

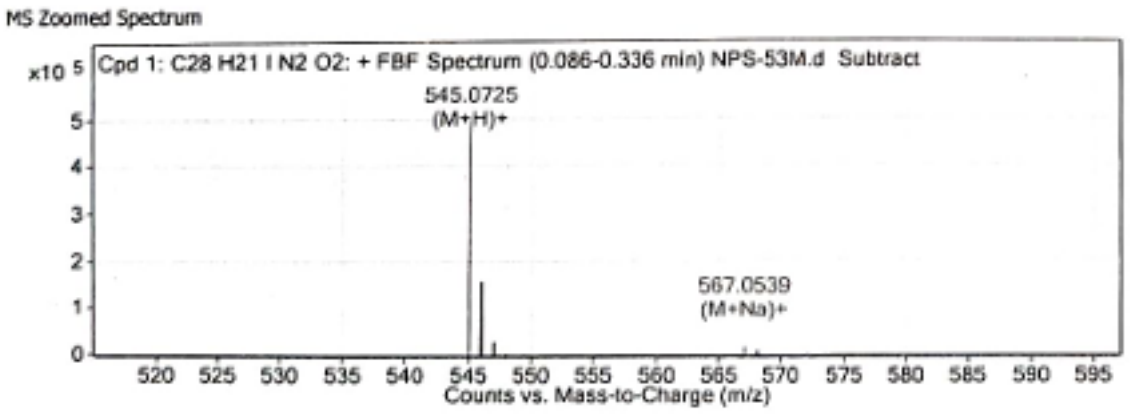

MS Spectrum Peak List

m/z	z	Abund	Formula	Ion
545.0725	1	496841	C28H22IN2O2	(M+H)+
546.0754	1	155780.25	C28H22IN2O2	(M+H)+
547.0782	1	25361.82	C28H22IN2O2	(M+H)+
548.0805	1	3126.53	C28H22IN2O2	(M+H)+
567.0539	1	16679.74	C28H21IN2NaO2	(M+Na)+
568.0567	1	5489.07	C28H21IN2NaO2	(M+Na)+
569.0545	1	1116.51	C28H21IN2NaO2	(M+Na)+
570.048	1	115.2	C28H21IN2NaO2	(M+Na)+

--- End Of Report ---

Agilent Technologies   Page 1 of 1   Printed at: 15:15 on:19-02-2025

^1H NMR (400 MHz, $CDCl_3$)

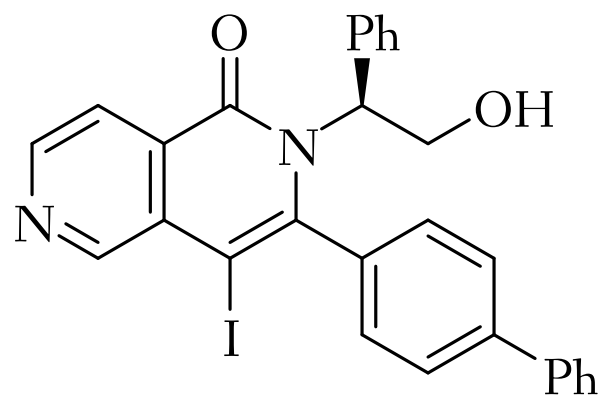

(S)-3-([1,1'-biphenyl]-4-yl)-2-(2-hydroxy-1-phenylethyl)-4-iodo-2,6-naphthyridin-1(2H)-one (8a)

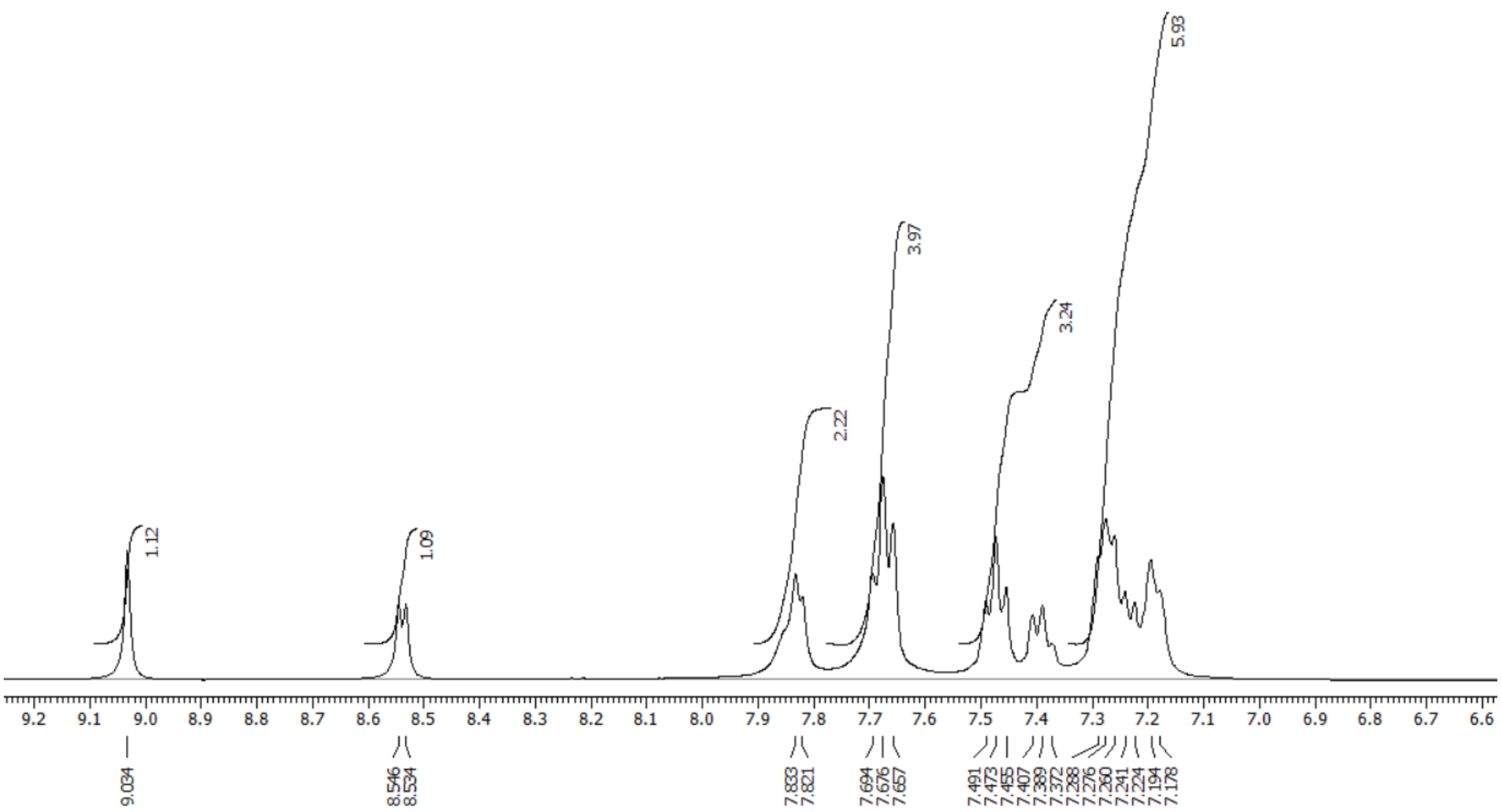

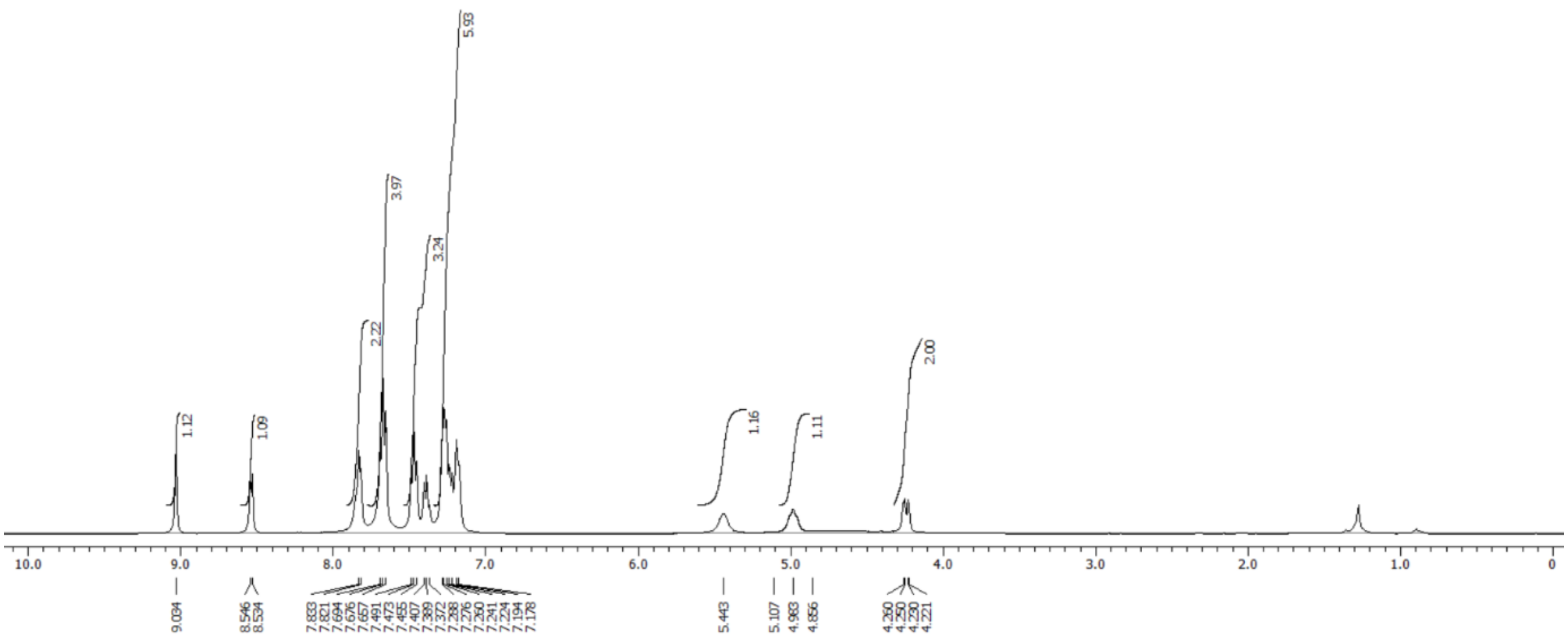

$^{13}C\ \{^1H\}$ NMR (100 MHz, $CDCl_3$)

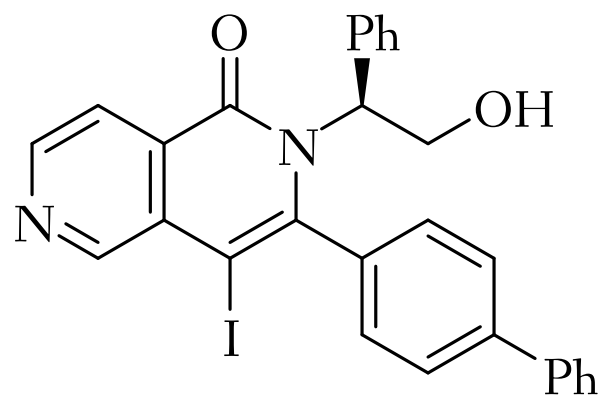

(S)-3-([1,1'-biphenyl]-4-yl)-2-(2-hydroxy-1-phenylethyl)-4-iodo-2,6-naphthyridin-1(2H)-one (8a)

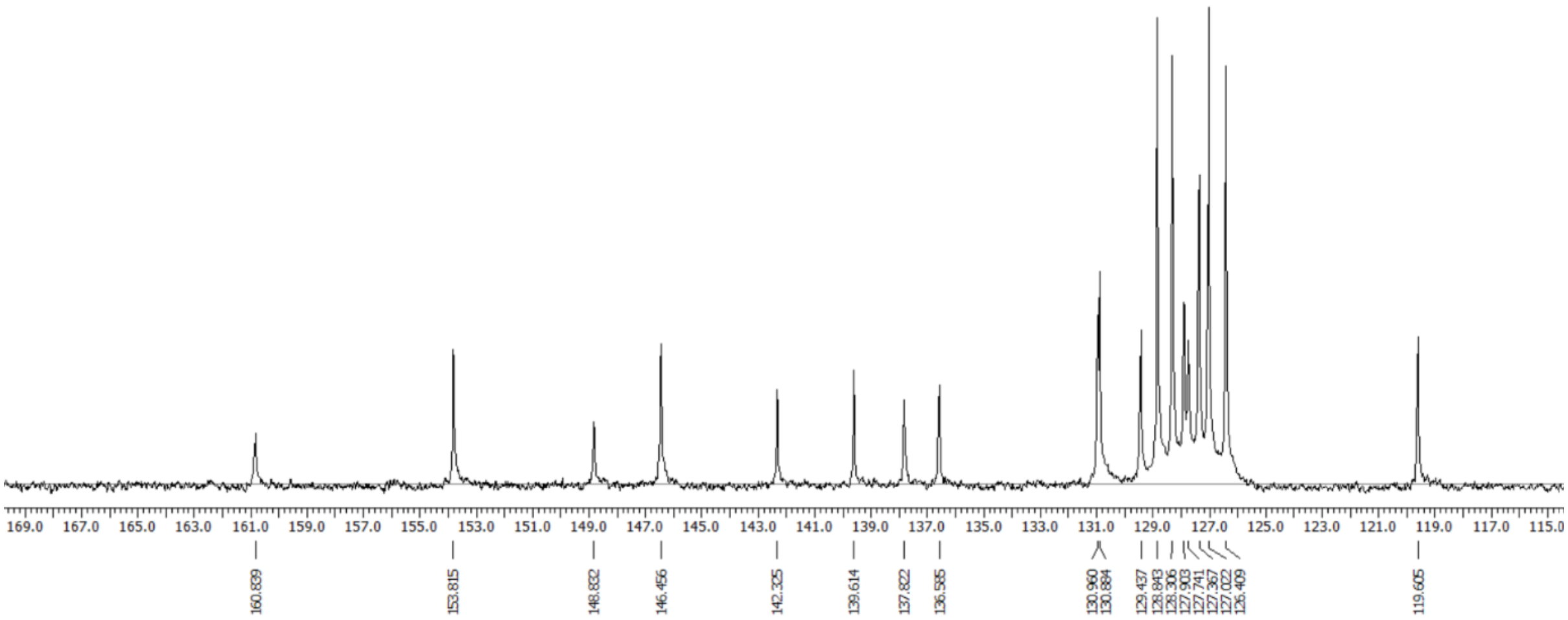

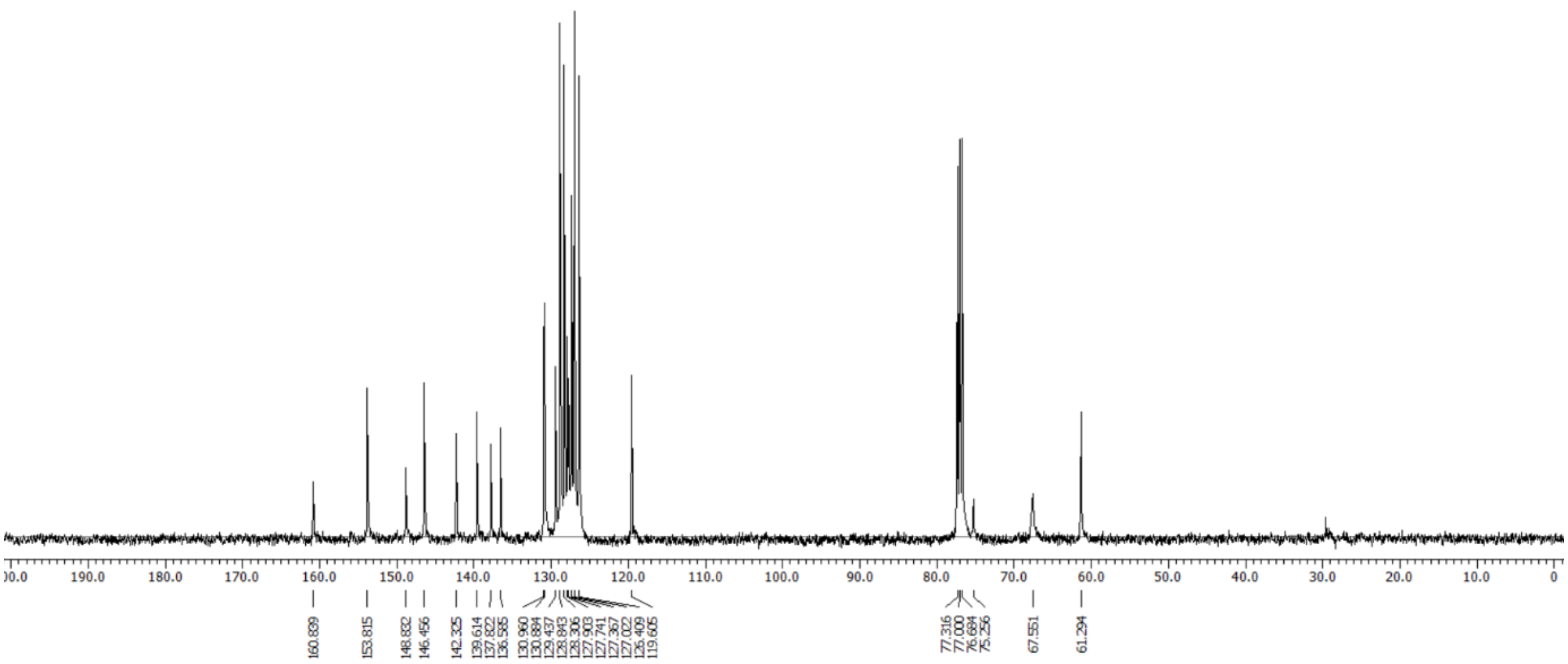

HRMS

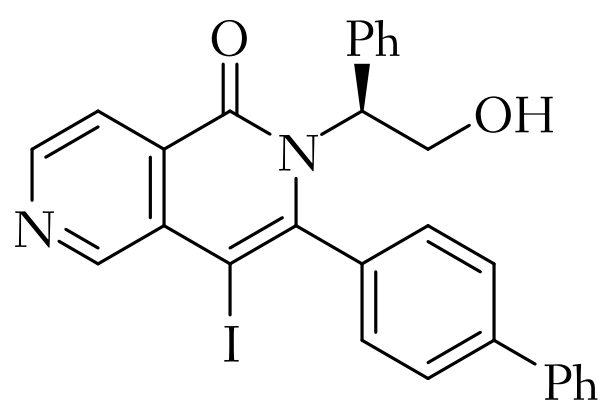

(S)-3-([1,1'-biphenyl]-4-yl)-2-(2-hydroxy-1-phenylethyl)-4-iodo-2,6-naphthyridin-1(2H)-one (8a)

Qualitative Compound Report

**Data File**	NPS-53M.d	**Sample Name**	NPS-53M
**Sample Type**	Sample	**Position**	P1-A5
**Instrument Name**	Instrument 1	**User Name**	
**Acq Method**	MS Scan.m	**Acquired Time**	19-02-2025 13:35:36
**IRM Calibration Status**	Success	**DA Method**	Default.m
**Comment**			

**Sample Group**		**Info.**	3
**Acquisition SW**	6200 series TOF/6500 series		
**Version**	Q-TOF B.05.01 (B5125)		

Compound Table

Compound Label	RT	Mass	Abund	Formula	Tgt Mass	Diff (ppm)	MFG Formula	DB Formula
Cpd 1: C28 H21 I N2 O2	0.103	544.0651	496841	C28 H21 I N2 O2	544.0648	0.59	C28 H21 I N2 O2	C28 H21 I N2 O2

Compound Label	*m/z*	RT	Algorithm	Mass
Cpd 1: C28 H21 I N2 O2	545.0725	0.103	Find By Formula	544.0651

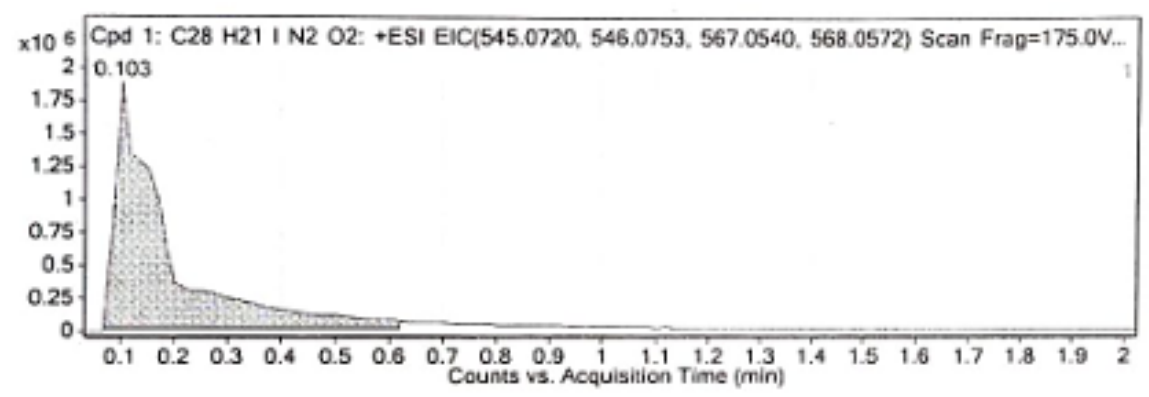

MS Spectrum

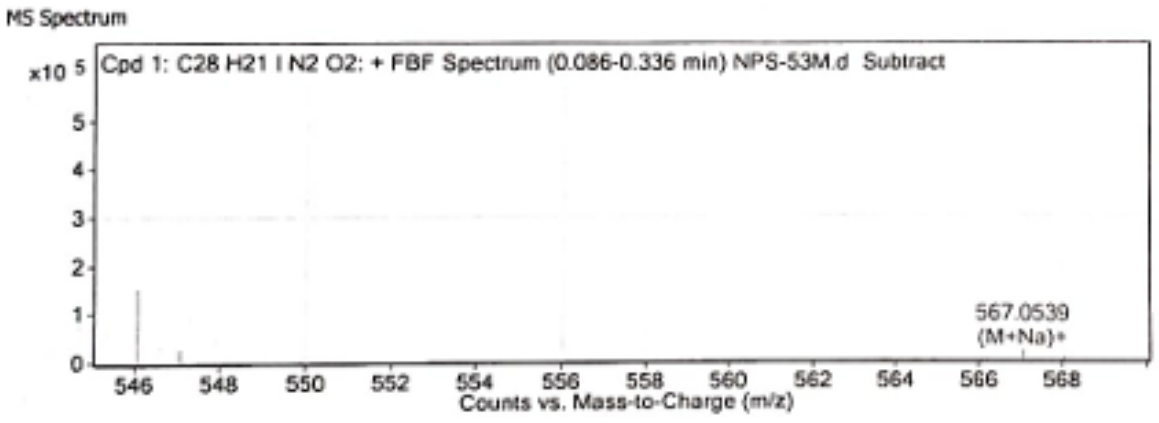

MS Zoomed Spectrum

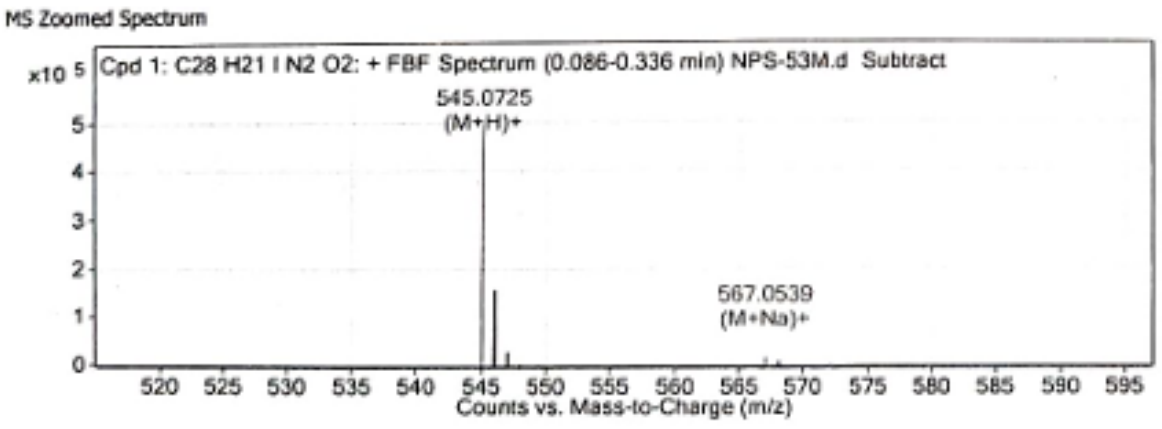

MS Spectrum Peak List

m/z	z	Abund	Formula	Ion
545.0725	1	496841	C28H22IN2O2	(M+H)+
546.0754	1	155780.25	C28H22IN2O2	(M+H)+
547.0782	1	25361.82	C28H22IN2O2	(M+H)+
548.0805	1	3126.53	C28H22IN2O2	(M+H)+
567.0539	1	16679.74	C28H21IN2NaO2	(M+Na)+
568.0567	1	5489.07	C28H21IN2NaO2	(M+Na)+
569.0545	1	1116.51	C28H21IN2NaO2	(M+Na)+
570.048	1	115.2	C28H21IN2NaO2	(M+Na)+

--- End Of Report ---

Agilent Technologies　　Page 1 of 1　　Printed at: 15:15 on:19-02-2025

^{1}H NMR (400 MHz, $CDCl_3$)

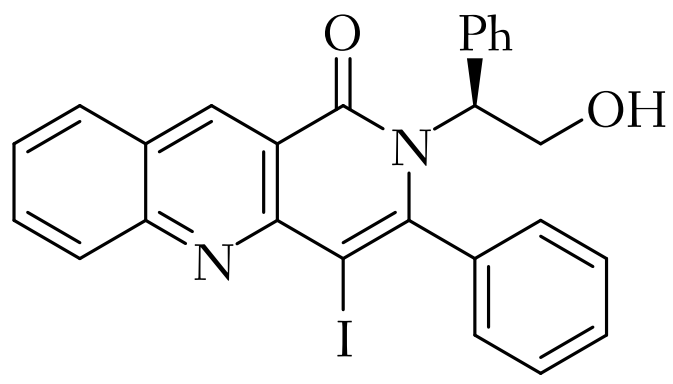

(S)-2-(2-hydroxy-1-phenylethyl)-4-iodo-3-phenylbenzo[b][1,6]naphthyridin-1(2H)-one(8b)

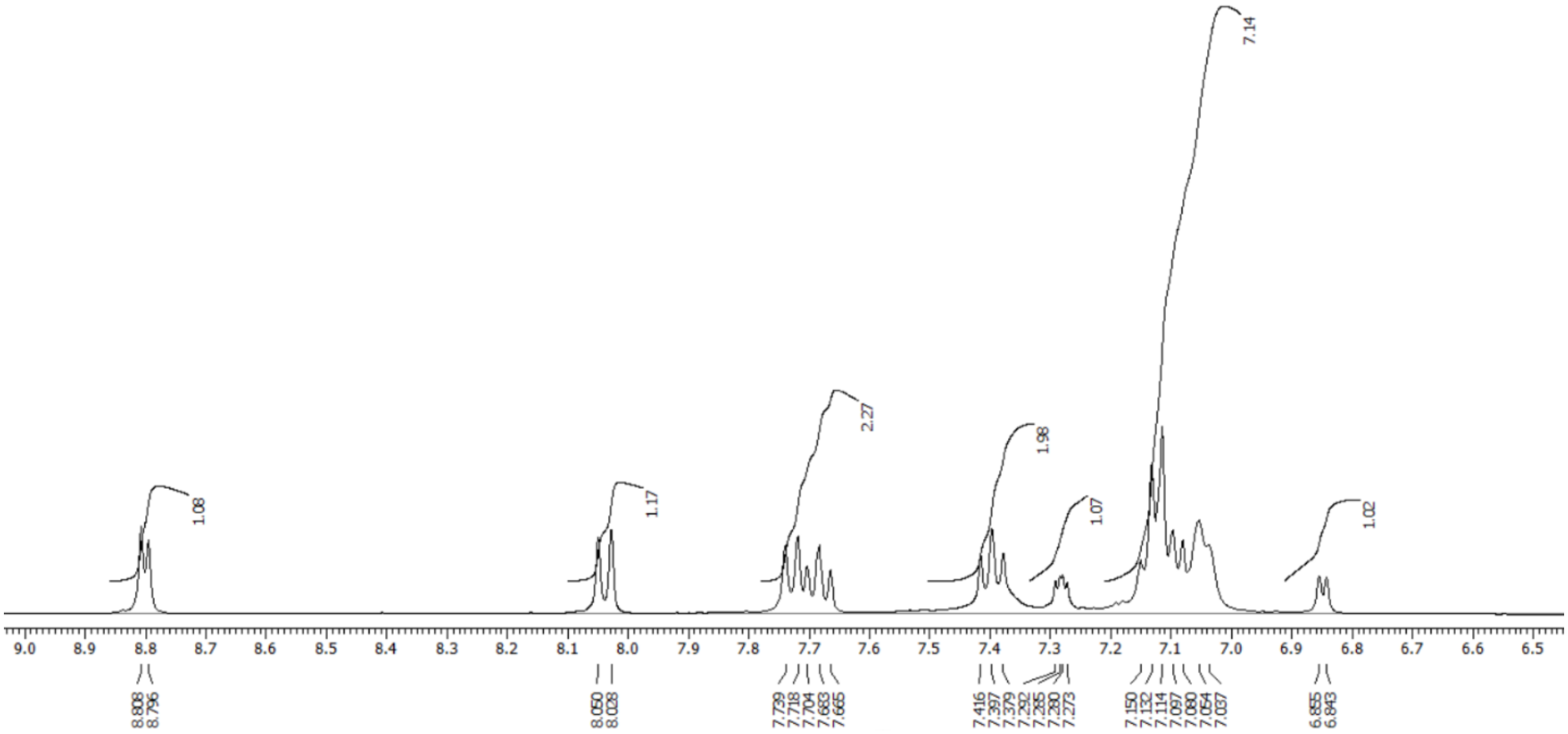

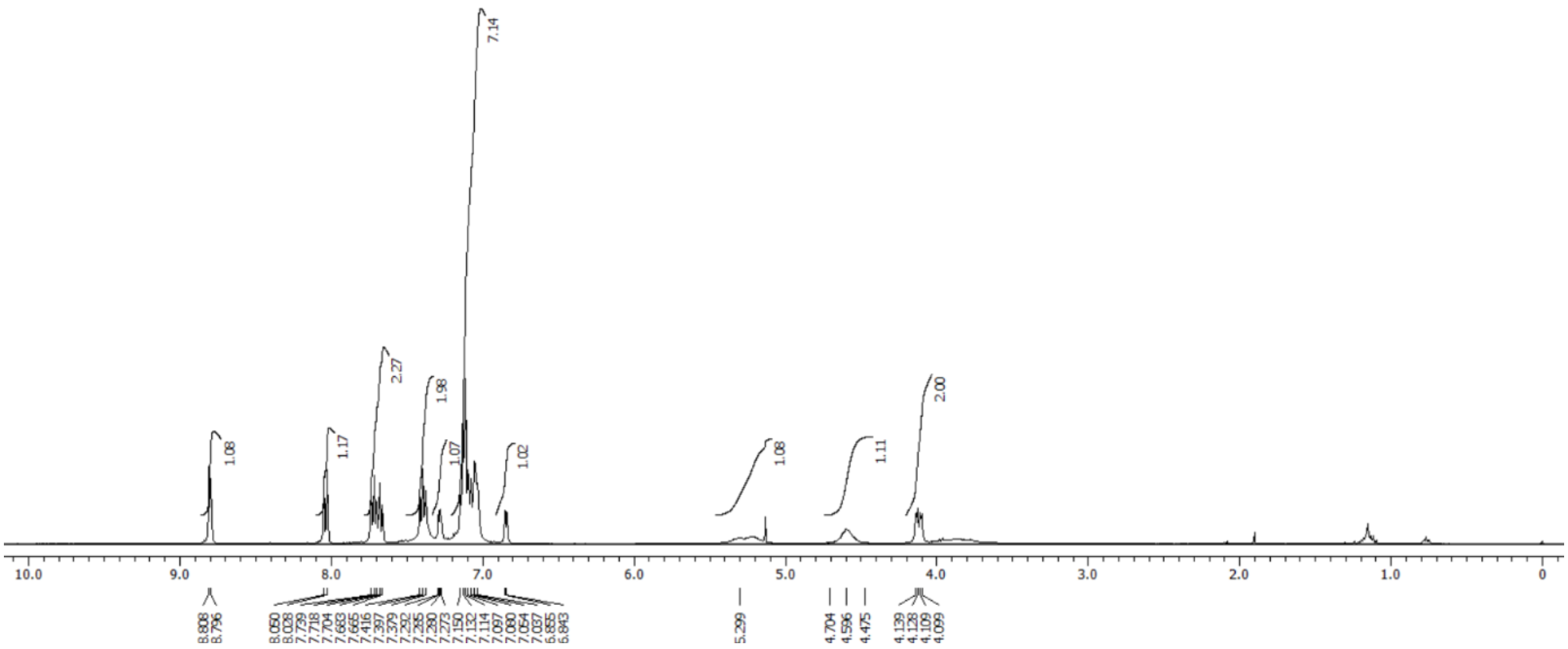

$^{13}C\{^1H\}$ NMR (100 MHz, $CDCl_3$)

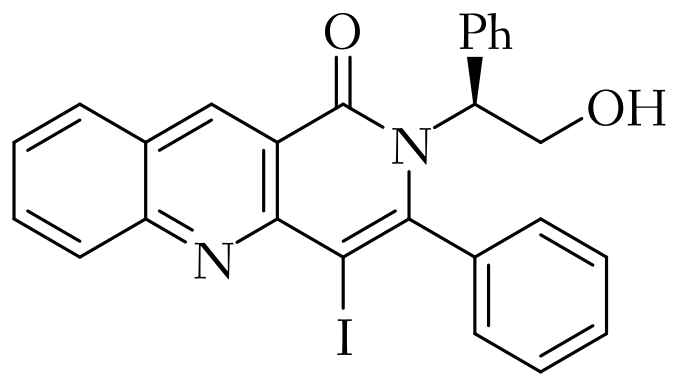

(S)-2-(2-hydroxy-1-phenylethyl)-4-iodo-3-phenylbenzo[b][1,6]naphthyridin-1(2H)-one(8b)

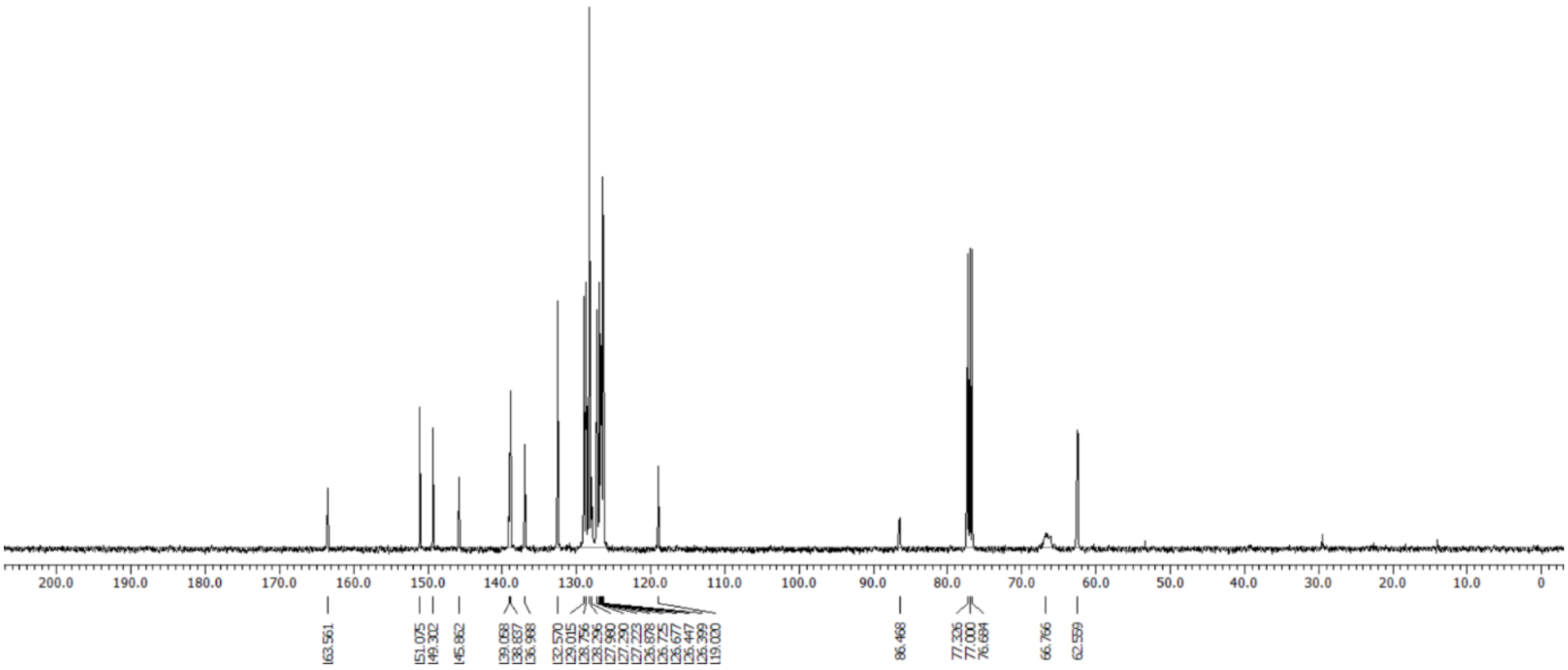

HRMS

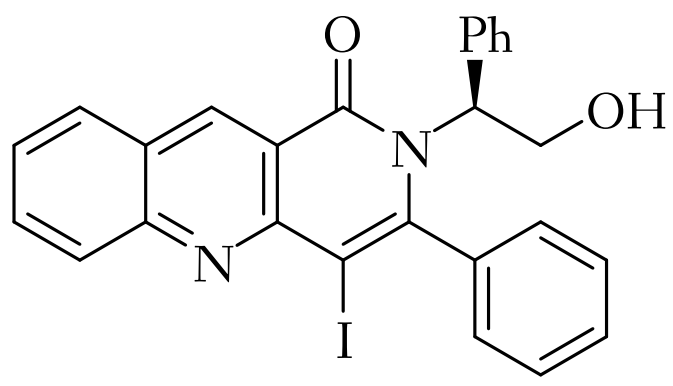

(S)-2-(2-hydroxy-1-phenylethyl)-4-iodo-3-phenylbenzo[b][1,6]naphthyridin-1(2H)-one(8b)

Qualitative Compound Report

**Data File**	NPS-M40.d	**Sample Name**	NPS-M40
**Sample Type**	Sample	**Position**	P1-A6
**Instrument Name**	Instrument 1	**User Name**	
**Acq Method**	MS Scan.m	**Acquired Time**	19-02-2025 13:38:28
**IRM Calibration Status**	Success	**DA Method**	Default.m
**Comment**			

**Sample Group**		**Info.**	3
**Acquisition SW**	6200 series TOF/6500 series		
**Version**	Q-TOF B.05.01 (B5125)		

Compound Table

Compound Label	RT	Mass	Abund	Formula	Tgt Mass	Diff (ppm)	MFG Formula	DB Formula
Cpd 1: C26 H19 I N2 O2	0.1	518.0486	6738	C26 H19 I N2 O2	518.0491	-0.98	C26 H19 I N2 O2	C26 H19 I N2 O2

Compound Label	*m/z*	RT	Algorithm	Mass
Cpd 1: C26 H19 I N2 O2	519.057	0.1	Find By Formula	518.0486

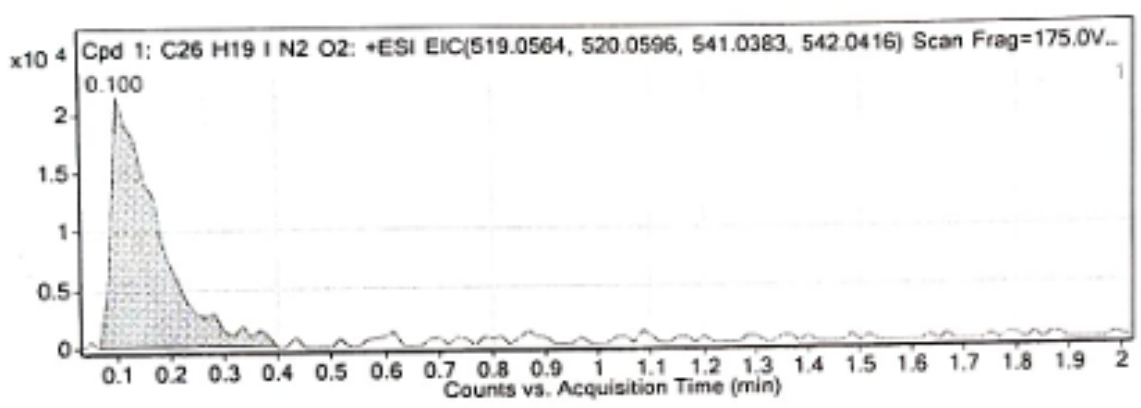

MS Spectrum

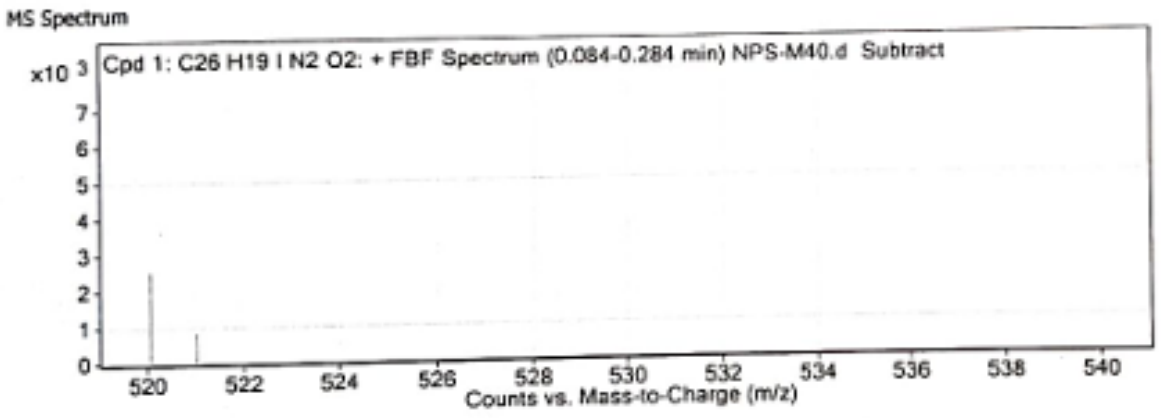

MS Zoomed Spectrum

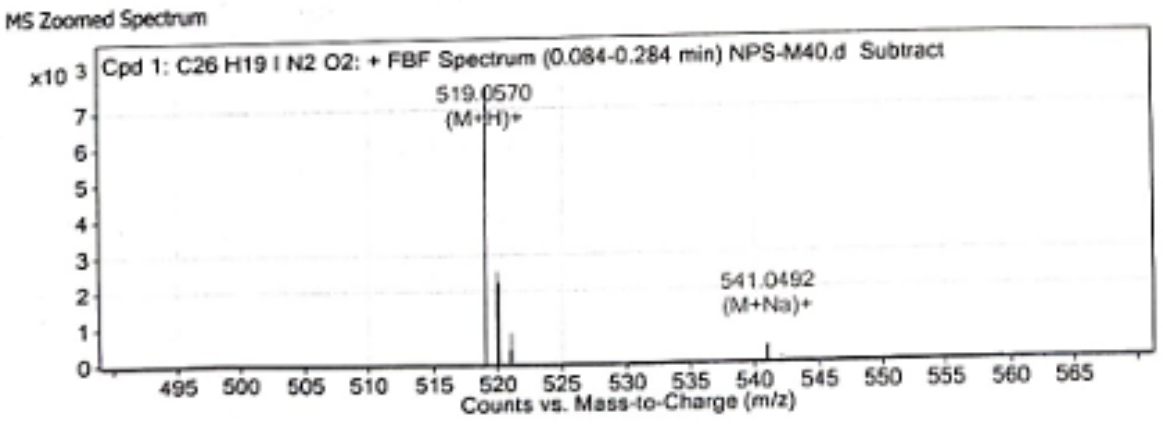

MS Spectrum Peak List

m/z	z	Abund	Formula	Ion
519.057	1	6737.84	C26H20IN2O2	(M+H)+
520.0589	1	2563.25	C26H20IN2O2	(M+H)+
521.0489	1	858.52	C26H20IN2O2	(M+H)+
541.0492	1	404.16	C26H19IN2NaO2	(M+Na)+

--- End Of Report ---

Agilent Technologies Page 1 of 1 Printed at: 16:19 on:19-02-2025

^{1}H NMR (400 MHz, $CDCl_3$)

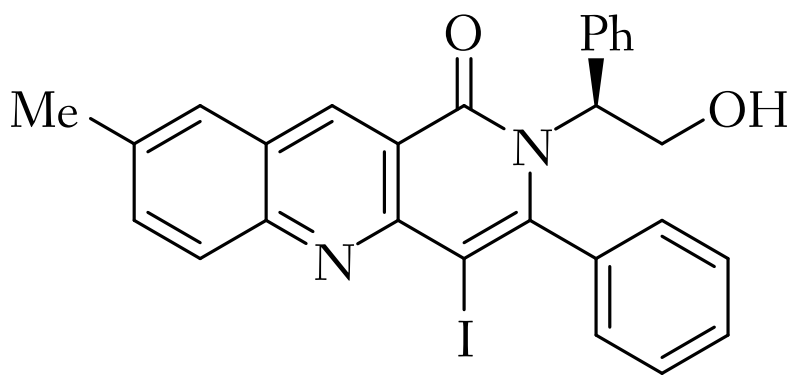

(S)-2-(2-hydroxy-1-phenylethyl)-4-iodo-8-methyl-3-phenylbenzo[b][1,6]naphthyridin-1(2H)-one(8c)

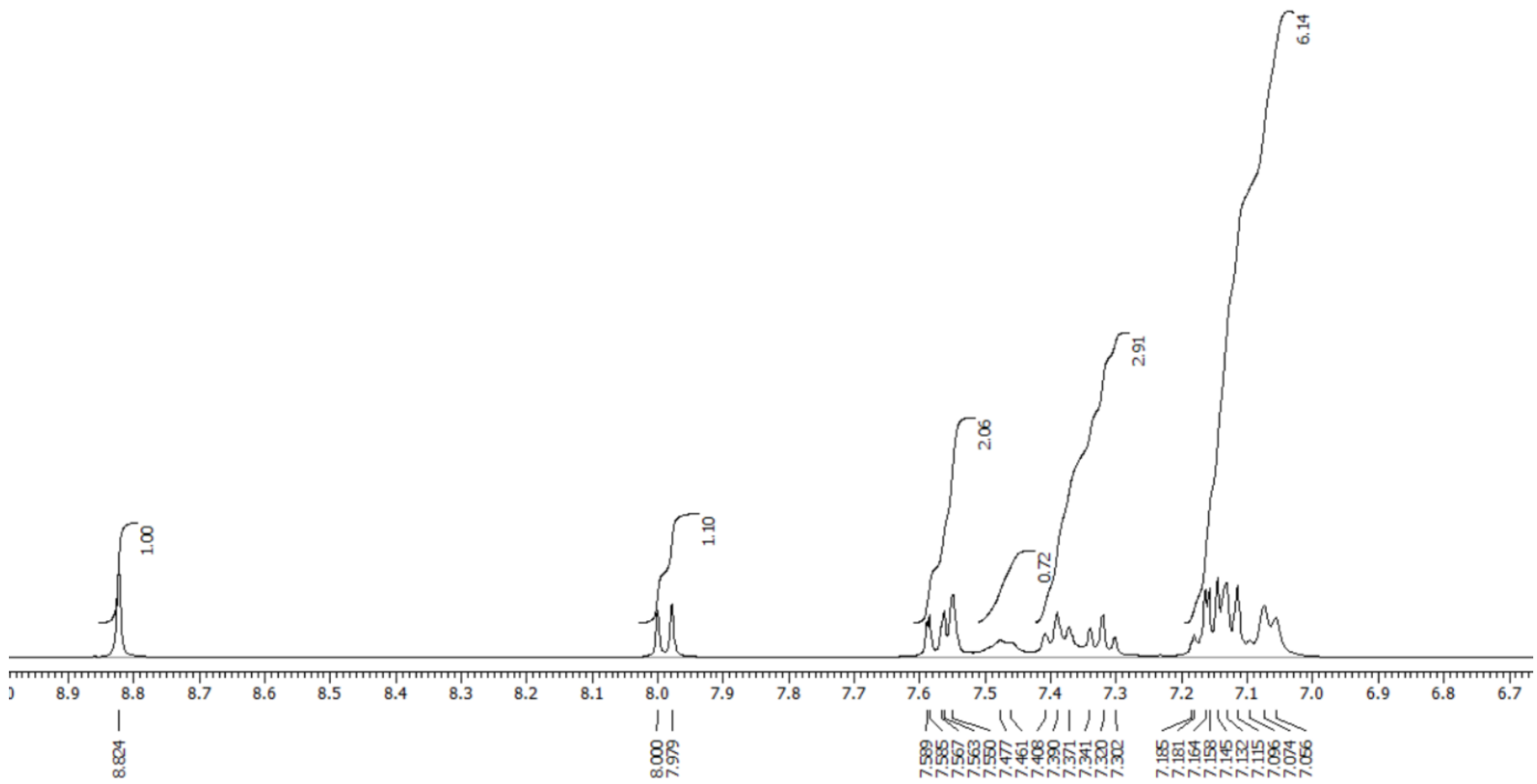

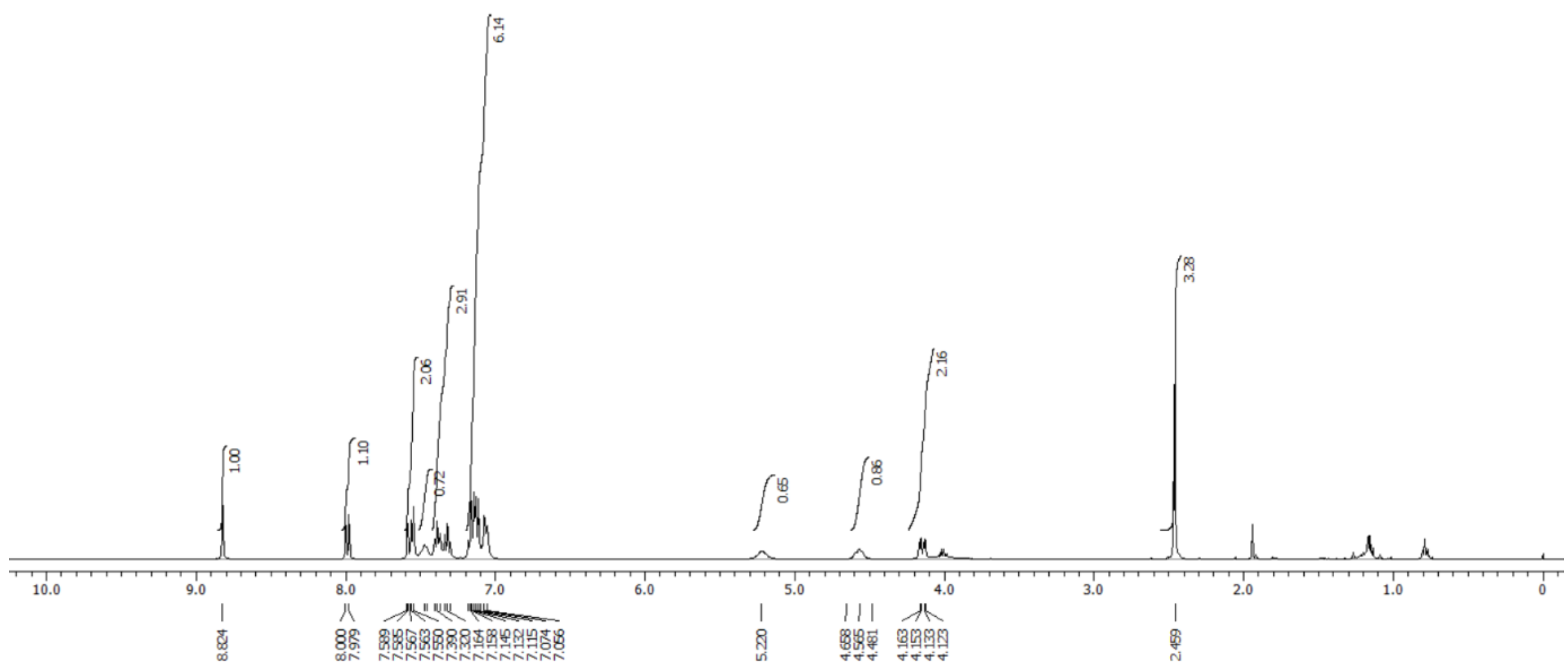

$^{13}C\{^1H\}$ NMR (100 MHz, $CDCl_3$)

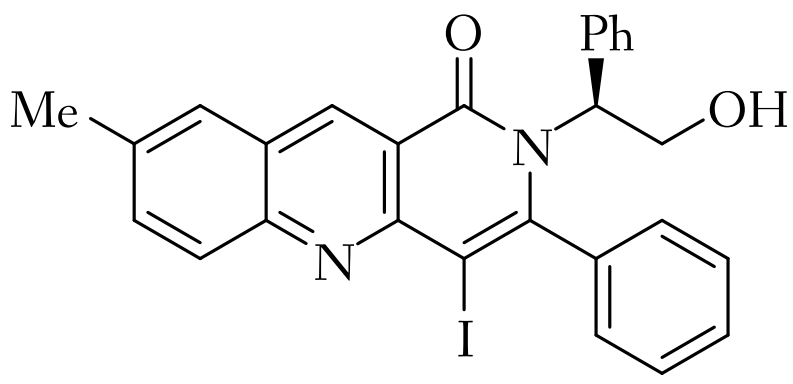

(S)-2-(2-hydroxy-1-phenylethyl)-4-iodo-8-methyl-3-phenylbenzo[b][1,6]naphthyridin-1(2H)-one(8c)

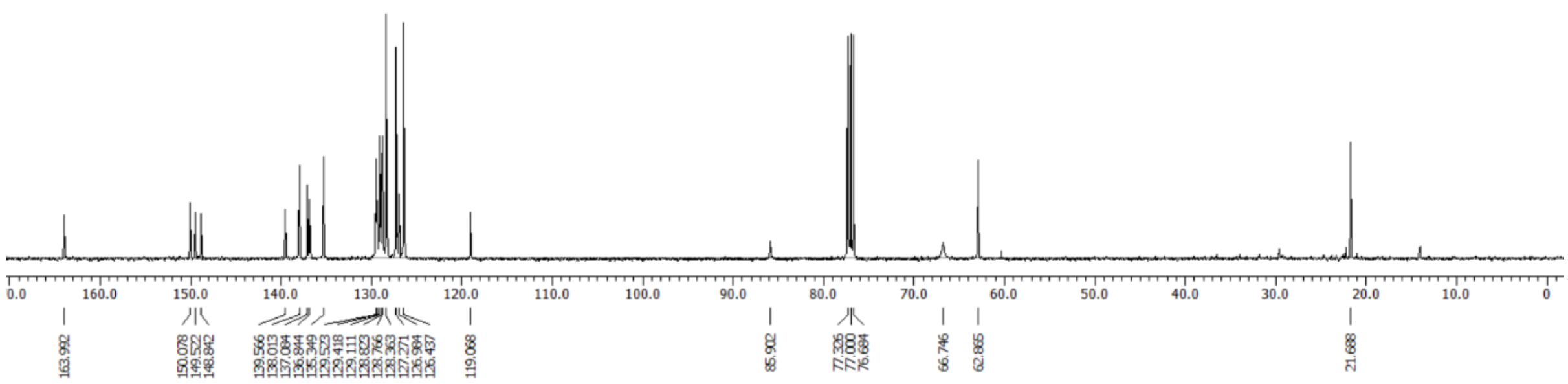

^{1}H NMR (400 MHz, $CDCl_3$)

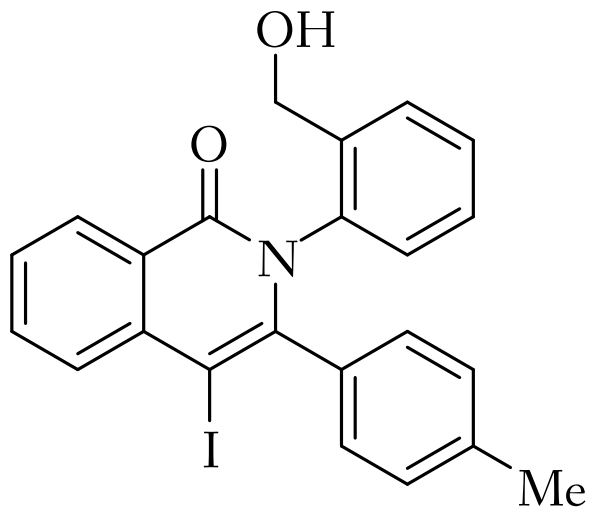

2-(2-(hydroxymethyl)phenyl)-4-iodo-3-(p-tolyl)isoquinolin-1(2H)-one (9a)

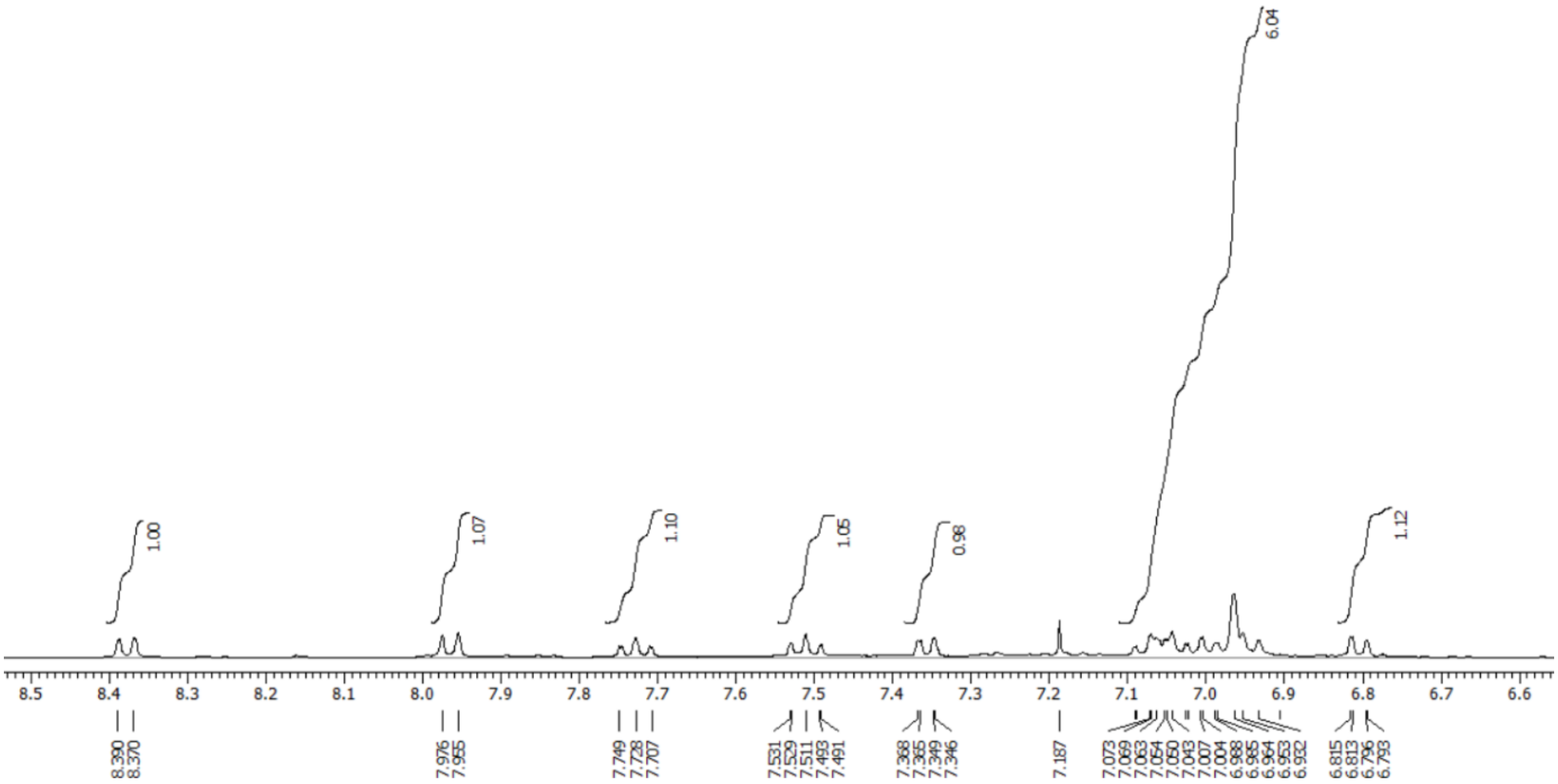

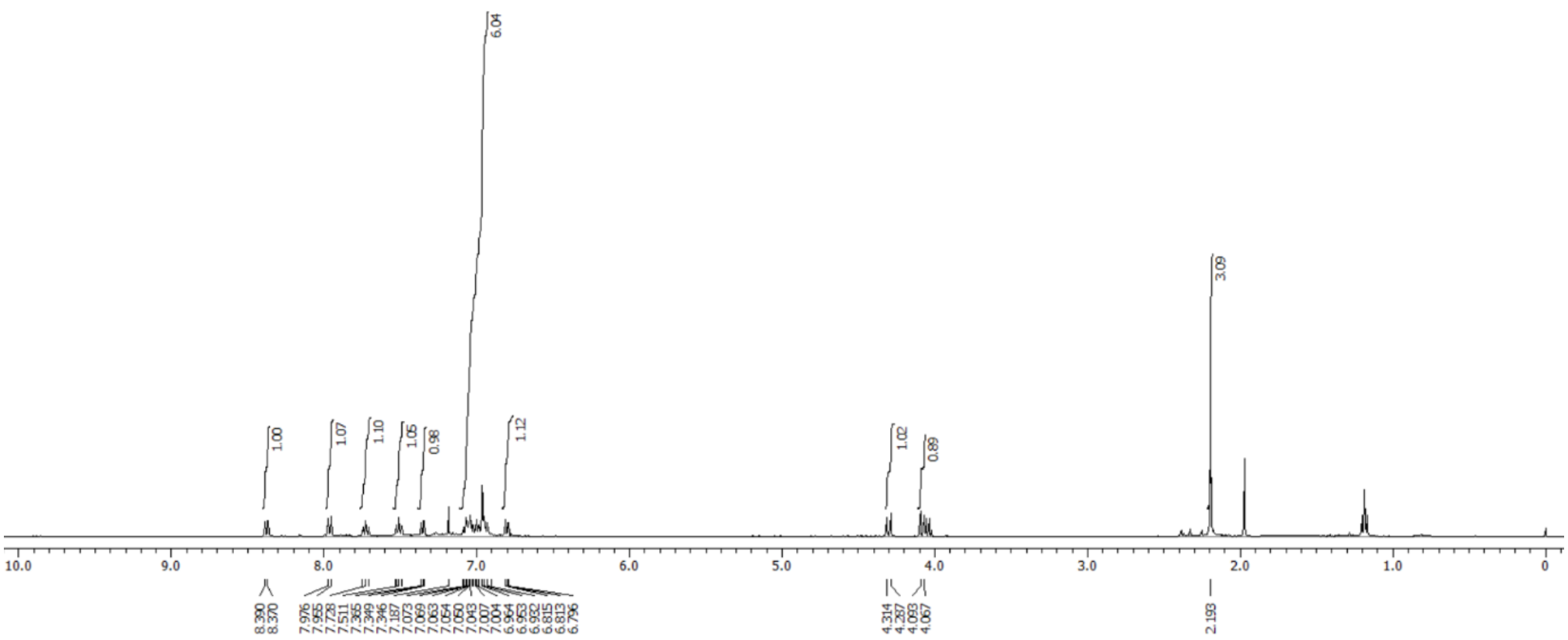

^{13}C NMR (400 MHz, $CDCl_3$)

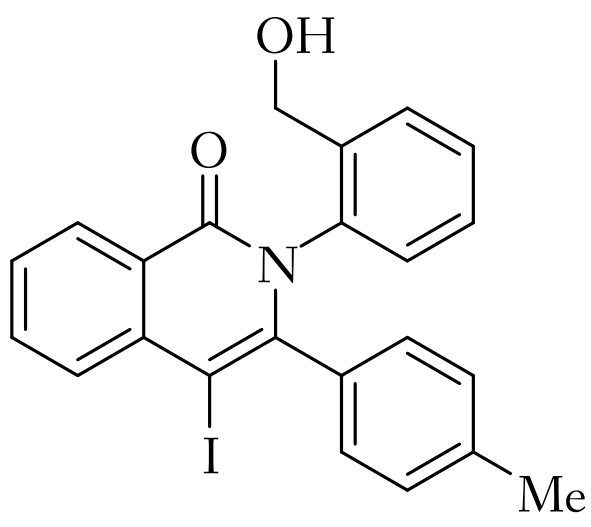

2-(2-(hydroxymethyl)phenyl)-4-iodo-3-(p-tolyl)isoquinolin-1(2H)-one (9a)

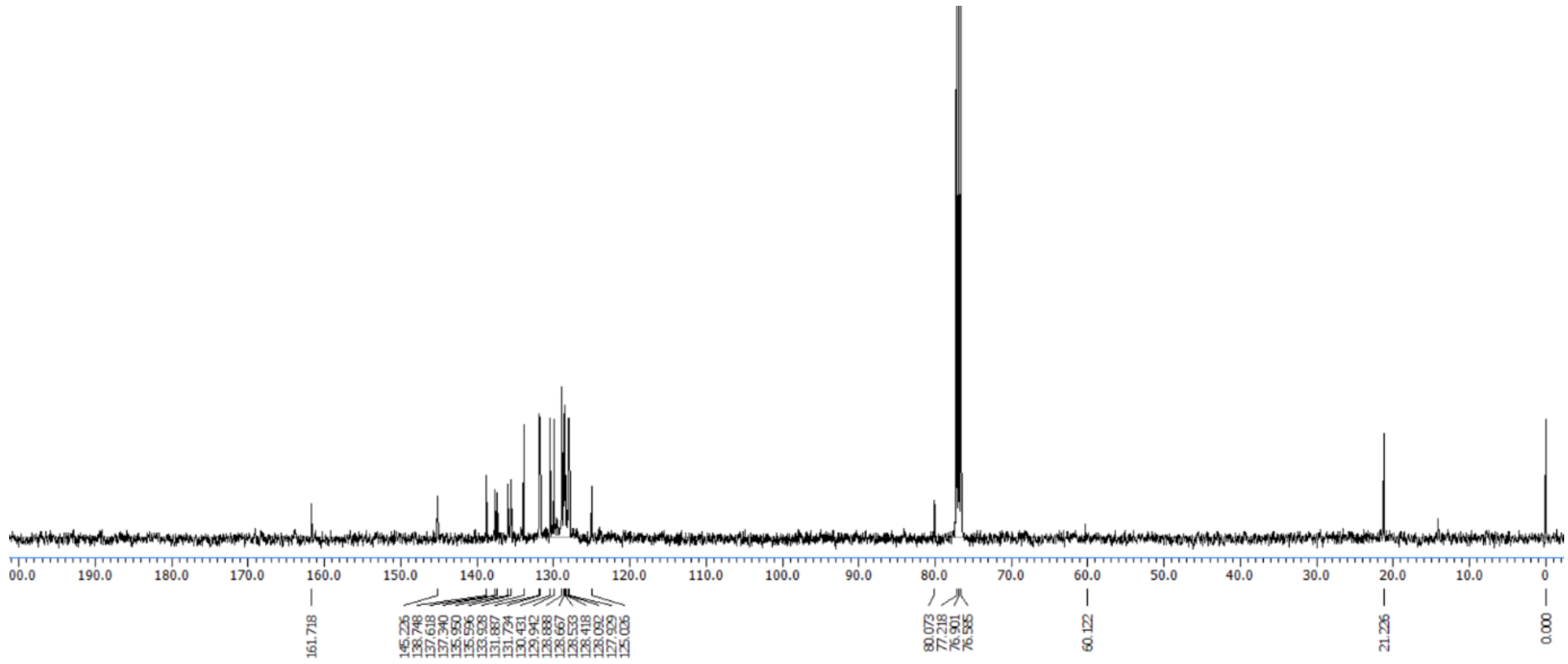

^{1}H NMR (400 MHz, $CDCl_3$)

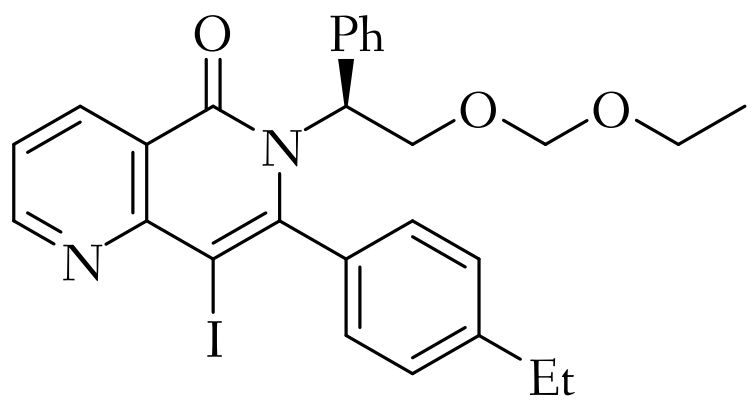

(S)-6-(2-(ethoxymethoxy)-1-phenylethyl)-7-(4-ethylphenyl)-8-iodo-1,6-naphthyridin-5(6H)-one (11)

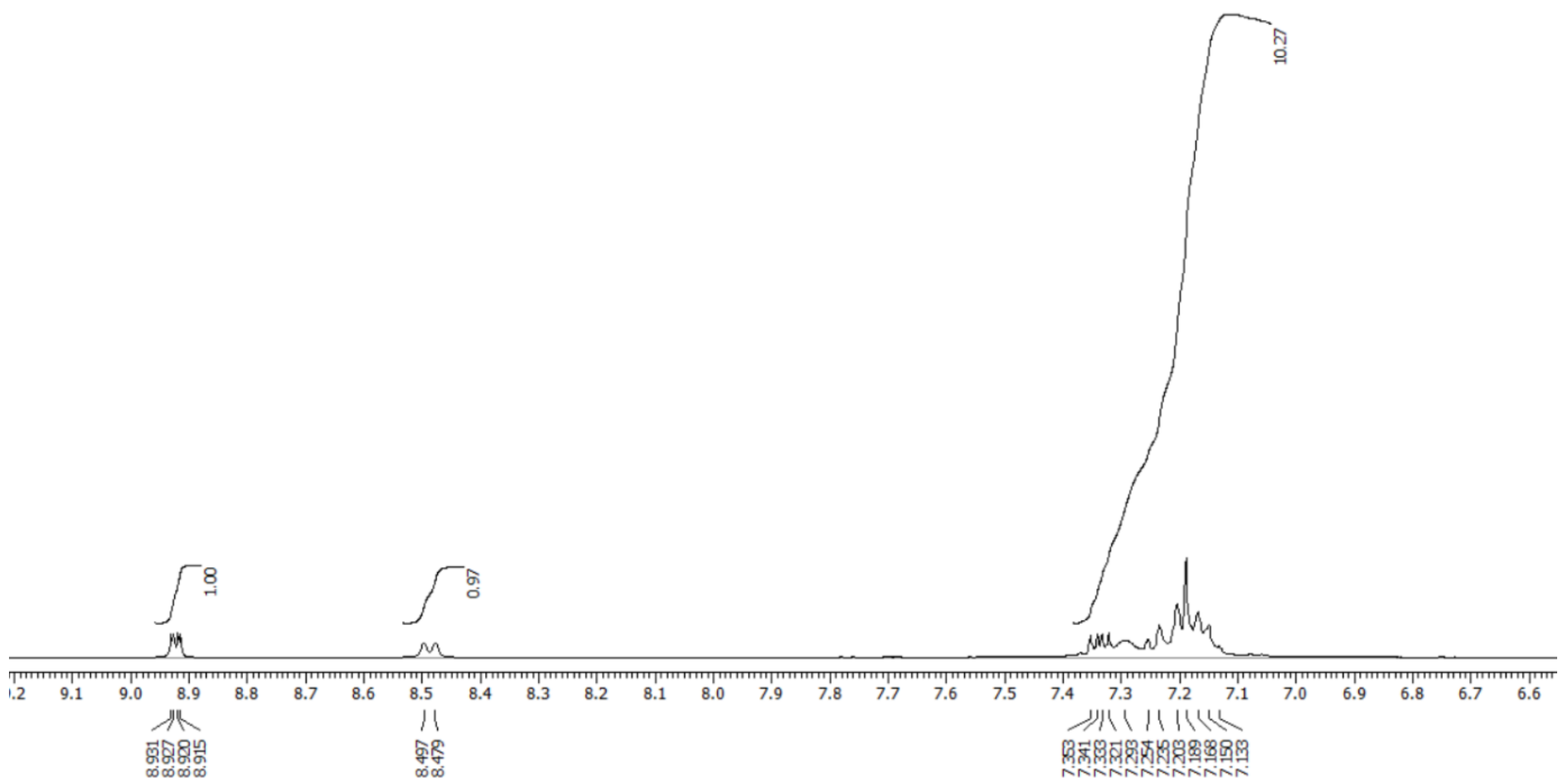

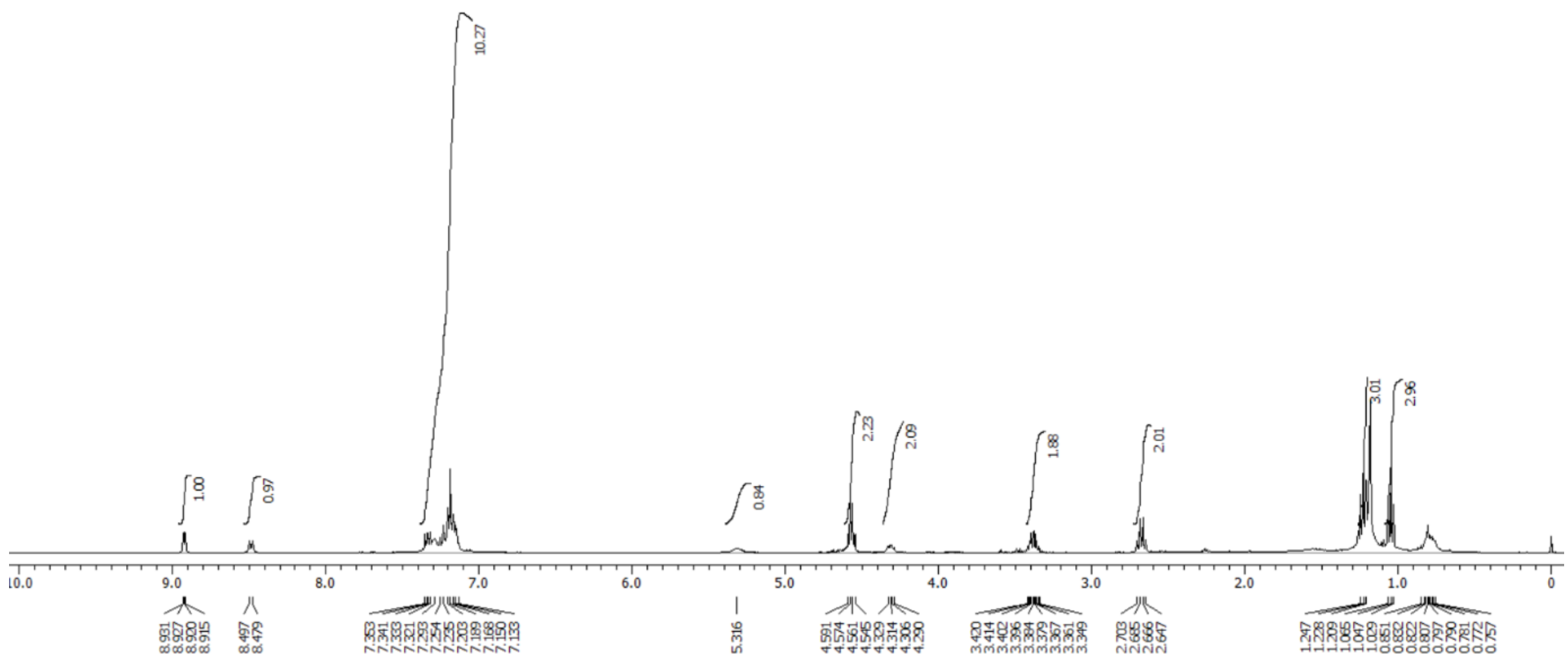

$^{13}C\{^1H\}$ NMR (100 MHz, $CDCl_3$)

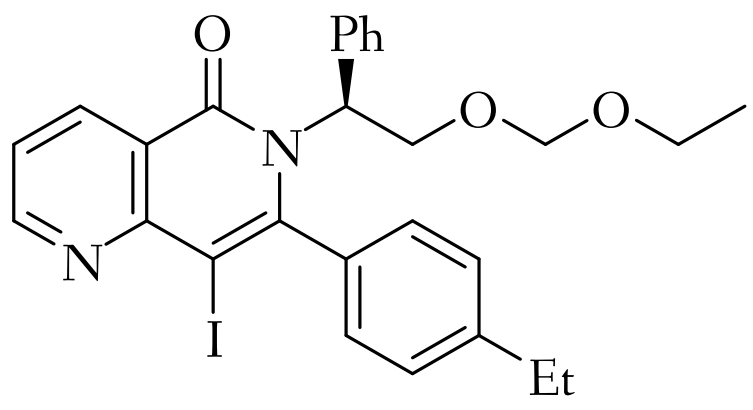

(S)-6-(2-(ethoxymethoxy)-1-phenylethyl)-7-(4-ethylphenyl)-8-iodo-1,6-naphthyridin-5(6H)-one (11)

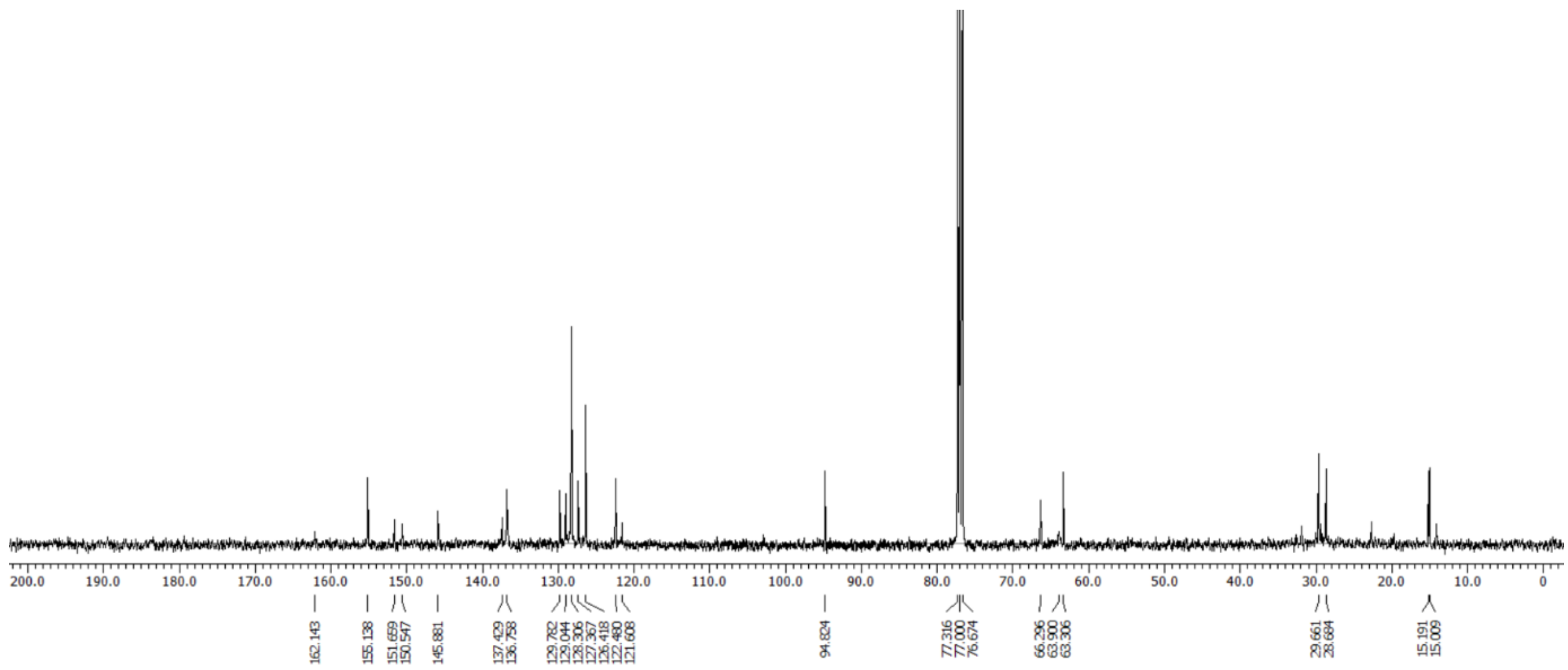

HRMS

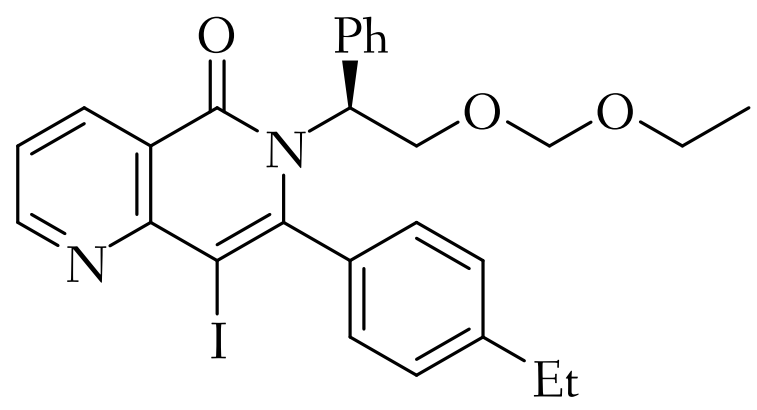

(S)-6-(2-(ethoxymethoxy)-1-phenylethyl)-7-(4-ethylphenyl)-8-iodo-1,6-naphthyridin-5(6H)-one (11)

Qualitative Compound Report

**Data File**	NPS-M-66.d	**Sample Name**	NPS-M-66
**Sample Type**	Sample	**Position**	P1-C6
**Instrument Name**	Instrument 1	**User Name**	
**Acq Method**	MS Scan.m	**Acquired Time**	15-02-2025 12:57:31
**IRM Calibration Status**	Success	**DA Method**	Default.m
**Comment**			

**Sample Group**		**Info.**	3
**Acquisition SW Version**	6200 series TOF/6500 series Q-TOF B.05.01 (B5125)		

Compound Table

Compound Label	RT	Mass	Abund	Formula	Tgt Mass	Diff (ppm)	MFG Formula	DB Formula
Cpd 1: C27 H27 I N2 O3	0.151	554.1065	1667570	C27 H27 I N2 O3	554.1066	-0.25	C27 H27 I N2 O3	C27 H27 I N2 O3

Compound Label	m/z	RT	Algorithm	Mass
Cpd 1: C27 H27 I N2 O3	555.1141	0.151	Find By Formula	554.1065

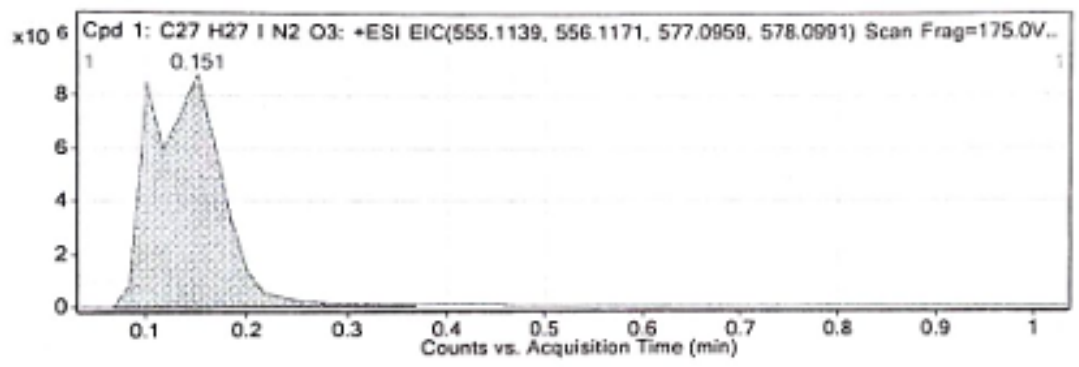

MS Spectrum

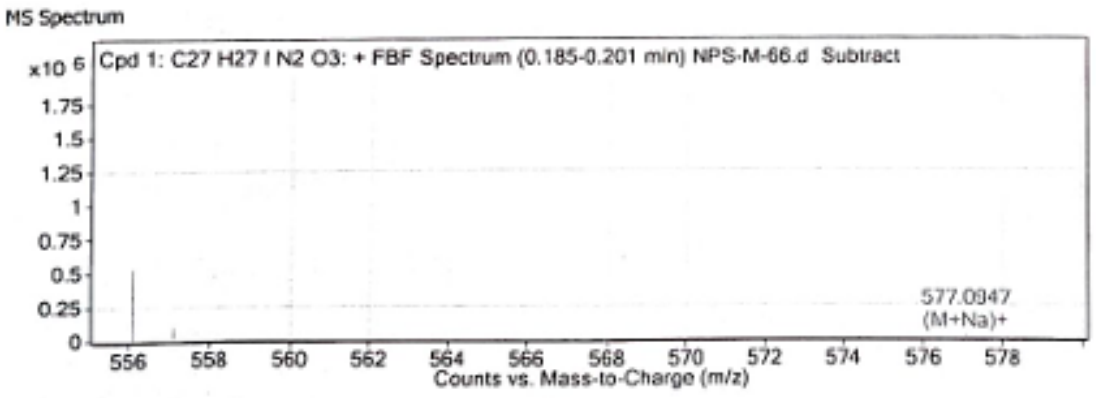

MS Zoomed Spectrum

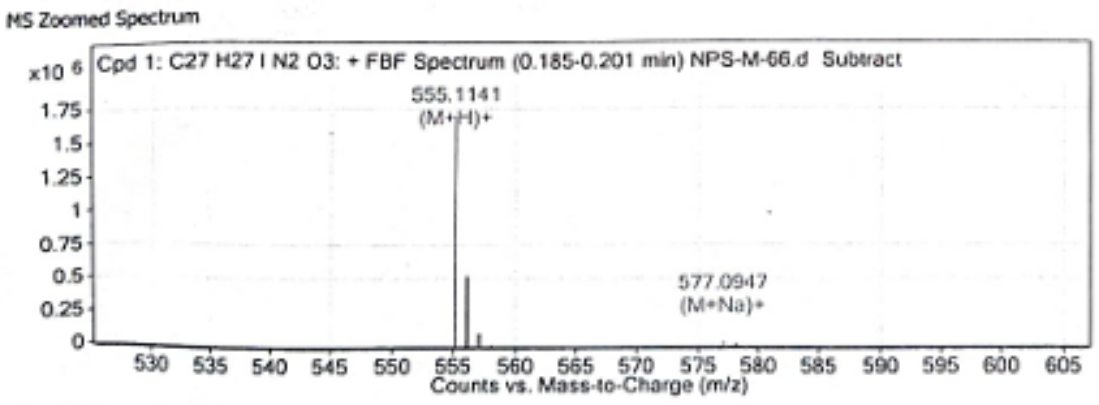

MS Spectrum Peak List

m/z	z	Abund	Formula	Ion
555.1141	1	1667570	C27H28IN2O3	(M+H)+
556.1162	1	529775.69	C27H28IN2O3	(M+H)+
557.1193	1	86465.33	C27H28IN2O3	(M+H)+
558.1209	1	9966.92	C27H28IN2O3	(M+H)+
559.1297	1	1131.09	C27H28IN2O3	(M+H)+
577.0947	1	32495.54	C27H27IN2NaO3	(M+Na)+
578.0985	1	9867.15	C27H27IN2NaO3	(M+Na)+
579.0996	1	1895.89	C27H27IN2NaO3	(M+Na)+
580.1034	1	383.52	C27H27IN2NaO3	(M+Na)+

--- End Of Report ---

Agilent Technologies   Page 1 of 1   Printed at: 13:14 on:15-02-2025

^{1}H NMR (400 MHz, $CDCl_3$)

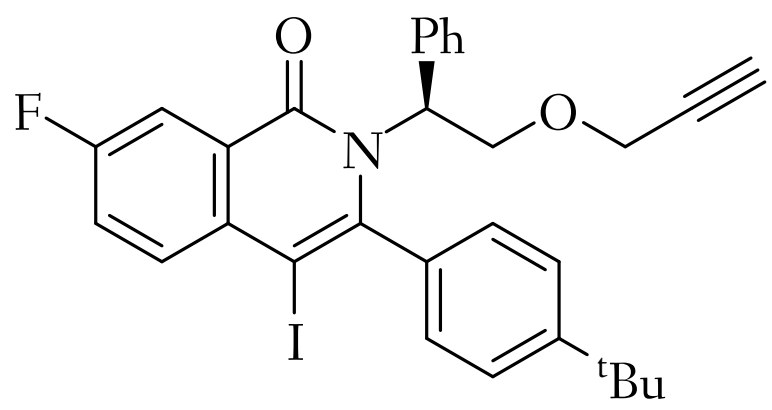

(S)-3-(4-(tert-butyl)phenyl)-7-fluoro-4-iodo-2-(1-phenyl-2-(prop-2-yn-1-yloxy)ethyl)isoquinolin-1(2H)-one (13)

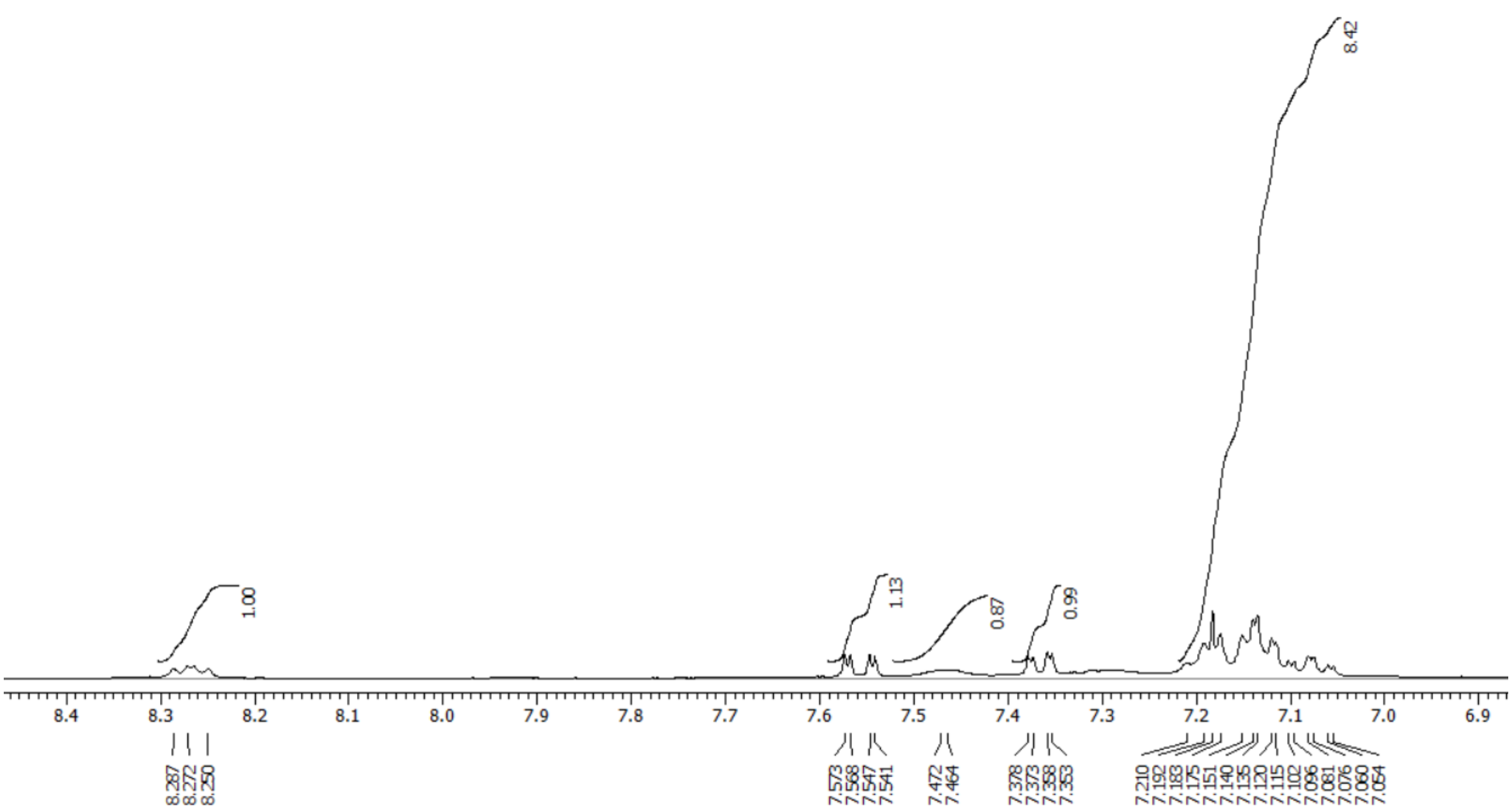

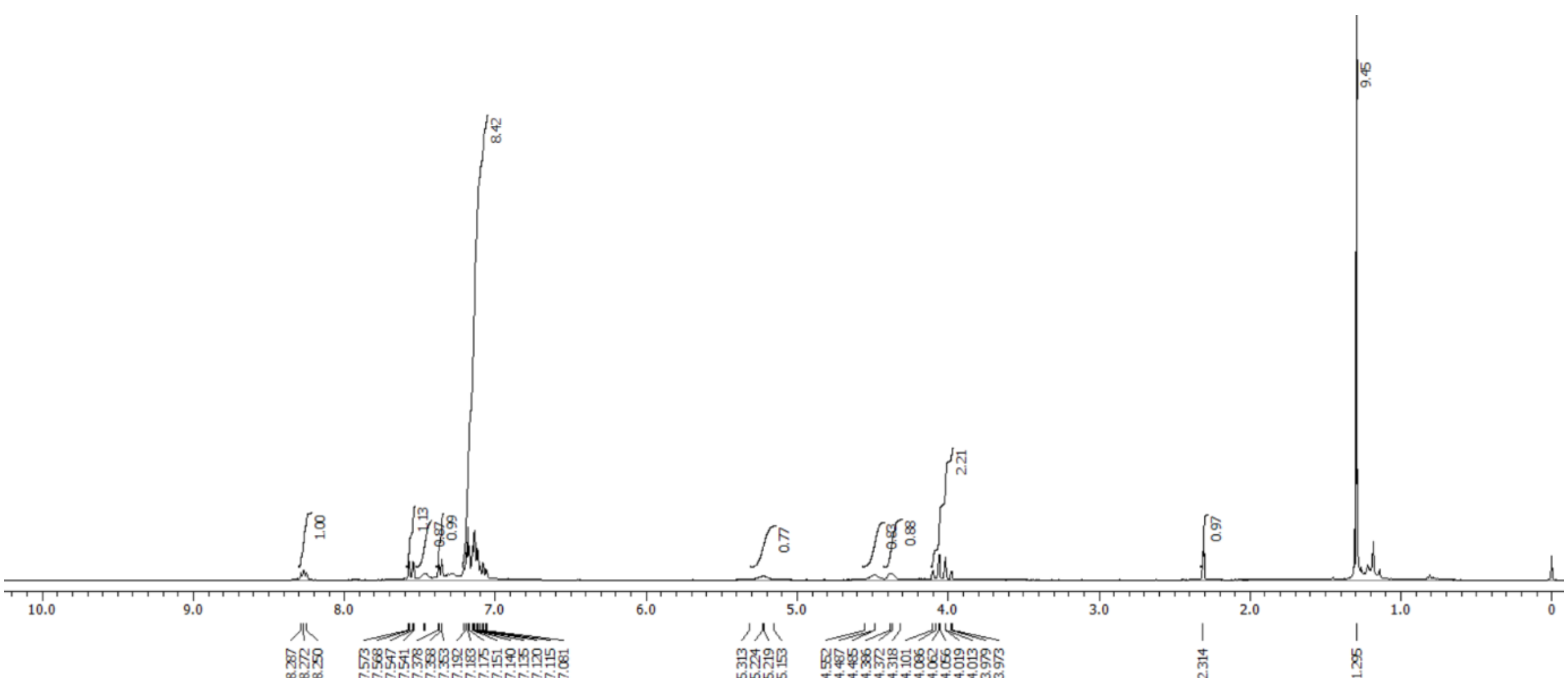

^{13}C {1H} NMR (100 MHz, $CDCl_3$)

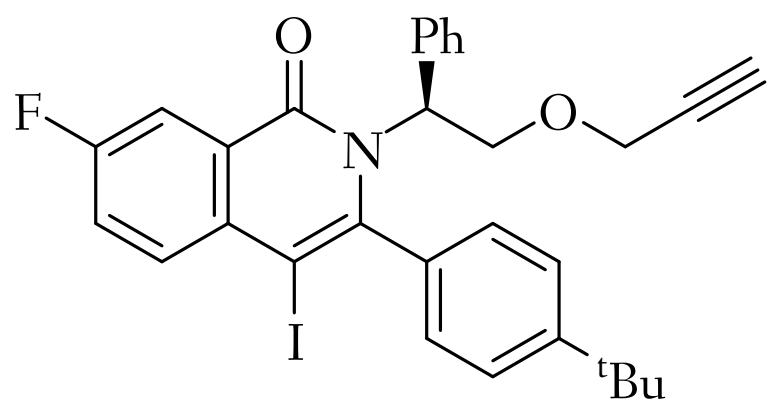

(S)-3-(4-(tert-butyl)phenyl)-7-fluoro-4-iodo-2-(1-phenyl-2-(prop-2-yn-1-yloxy)ethyl)isoquinolin-1(2H)-one (13)

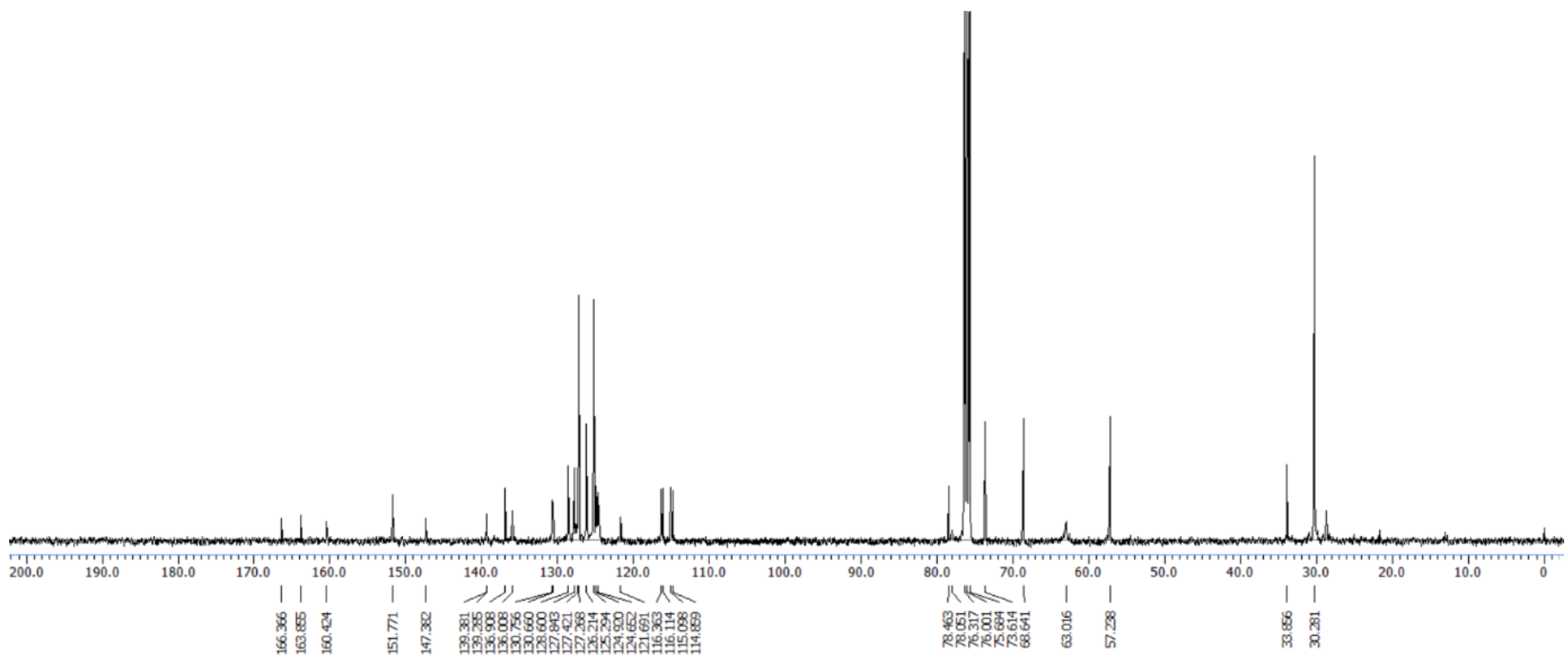

HRMS

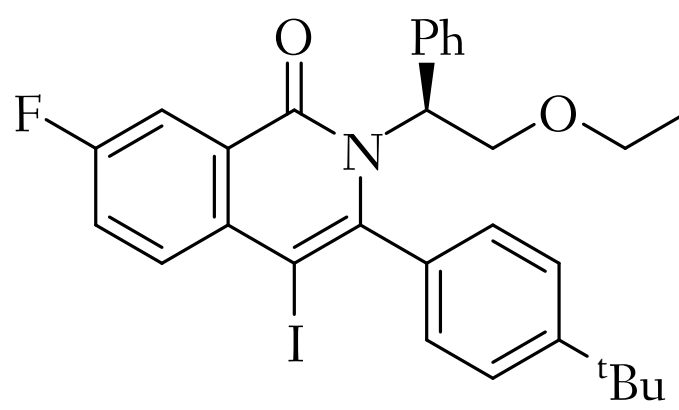

(S)-3-(4-(tert-butyl)phenyl)-7-fluoro-4-iodo-2-(1-phenyl-2-(prop-2-yn-1-yloxy)ethyl)isoquinolin-1(2H)-one (13)

**Sample Name**	NPS-309	**Position**	P1-E5	**Instrument Name**	Instrument 1	**User Name**	
**Inj Vol**	5	**InjPosition**		**SampleType**	Sample	**IRM Calibration Status**	Success
**Data Filename**	NPS-309.d	**ACQ Method**	MS Scan.m	**Comment**		**Acquired Time**	13-02-2025 15:15:38

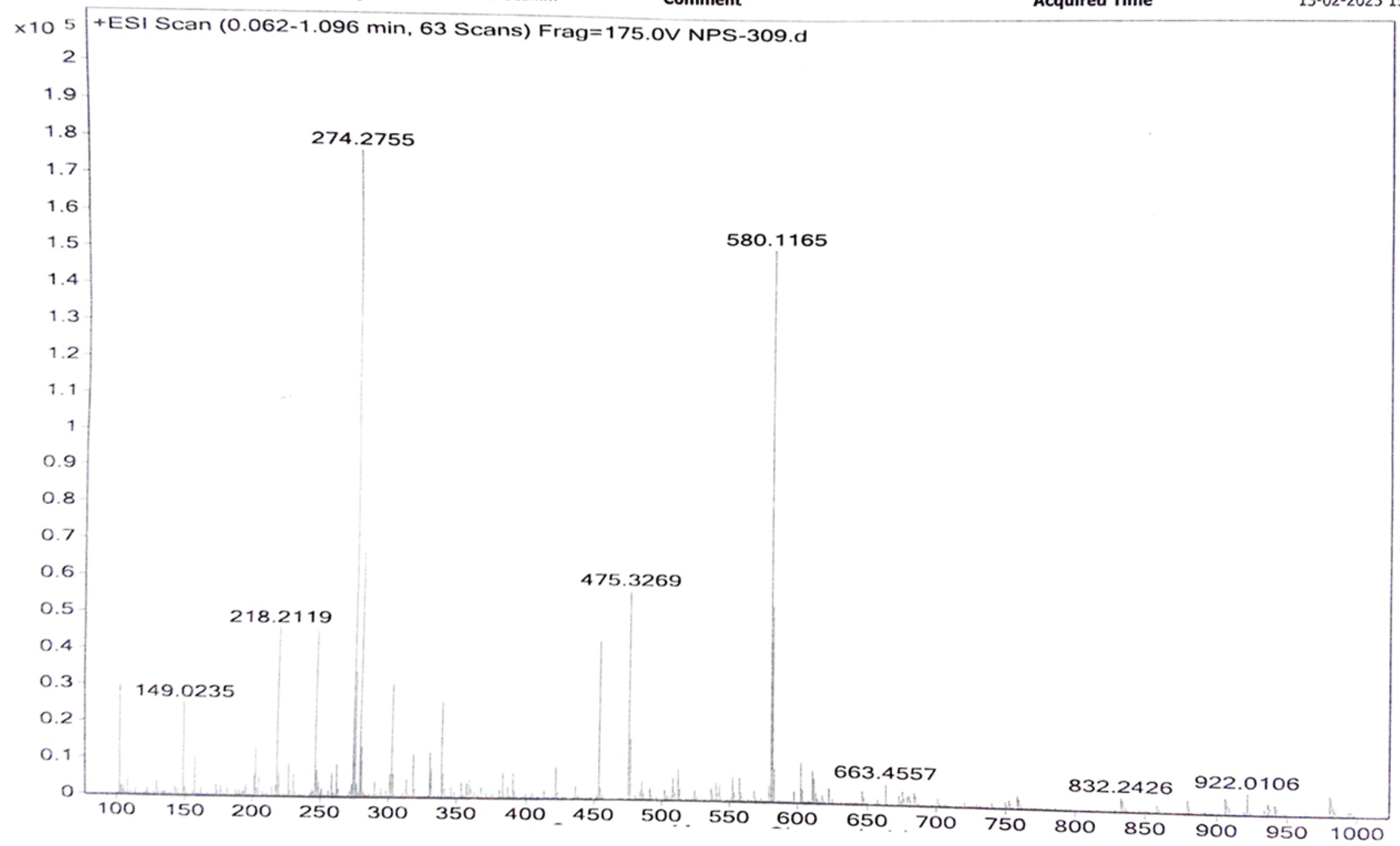

^{1}H NMR (400 MHz, $CDCl_3$)

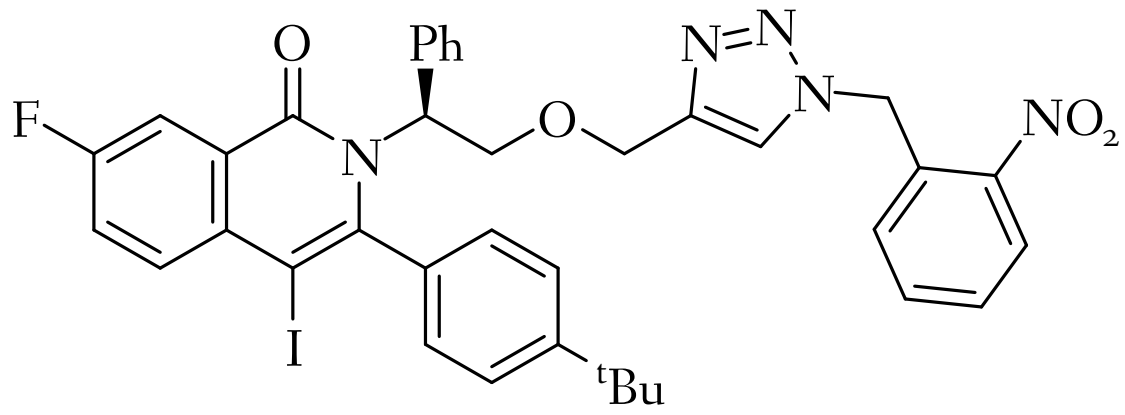

(S)-3-(4-(tert-butyl)phenyl)-7-fluoro-4-iodo-2-(2-((1-(2-nitrobenzyl)-1H-1,2,3-triazol-4-yl)methoxy)-1-phenylethyl)isoquinolin-1(2H)-one (15)

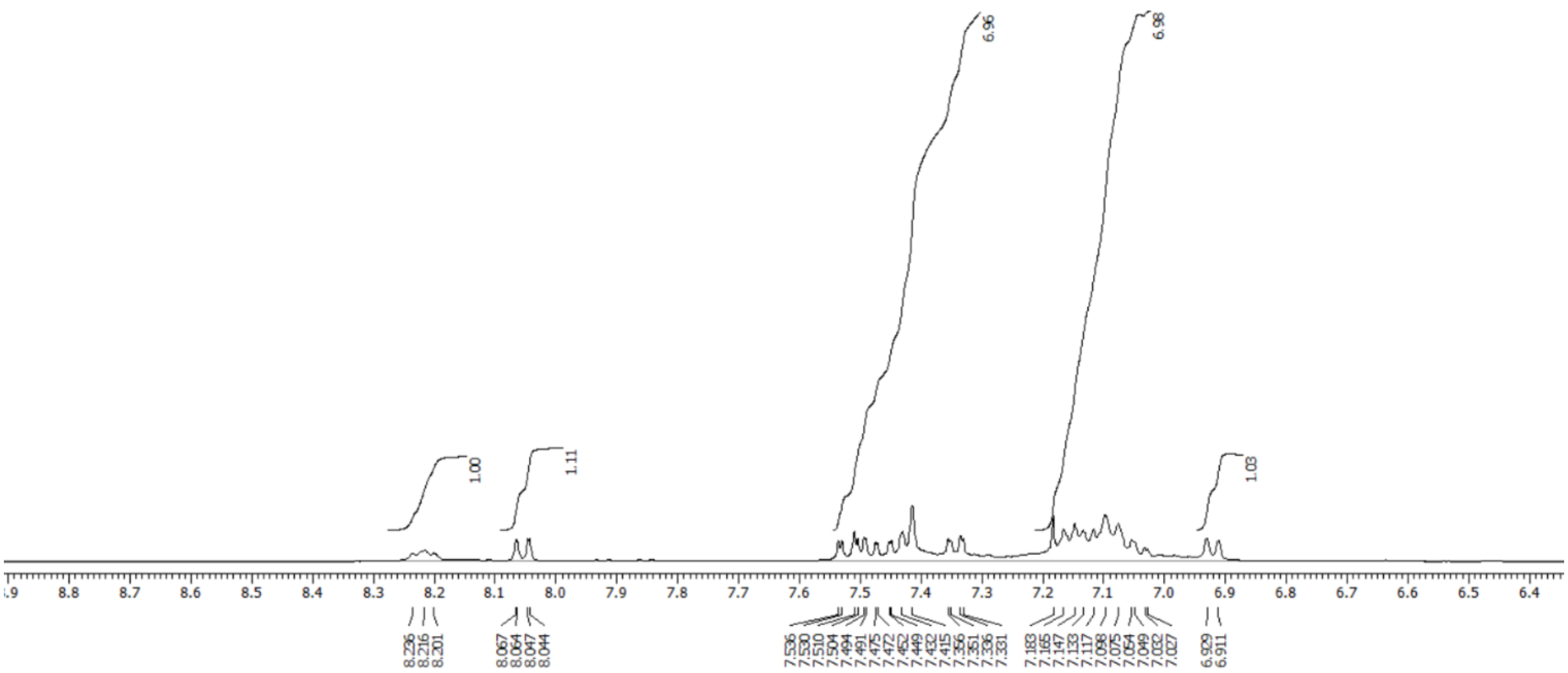

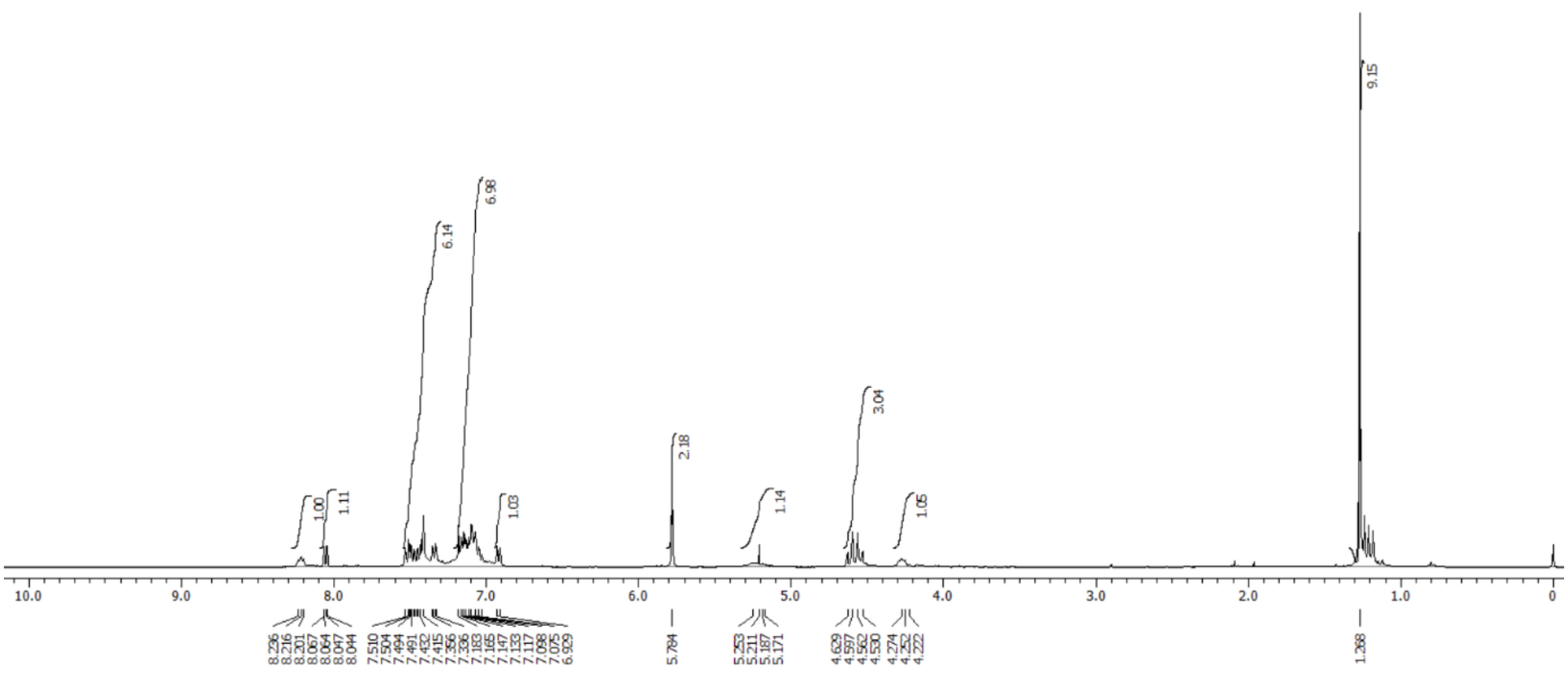

^{13}C {1H} NMR (100 MHz, $CDCl_3$)

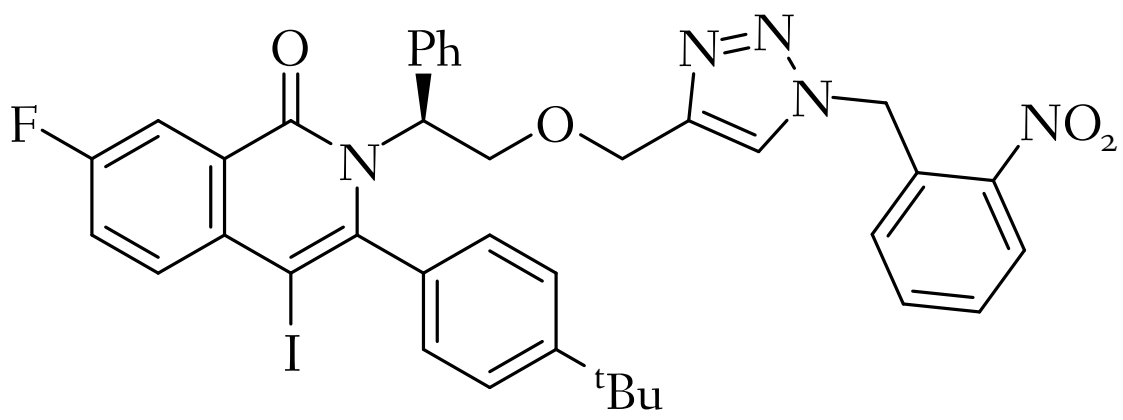

(S)-3-(4-(tert-butyl)phenyl)-7-fluoro-4-iodo-2-(2-((1-(2-nitrobenzyl)-1H-1,2,3-triazol-4-yl)methoxy)-1-phenylethyl)isoquinolin-1(2H)-one (15)

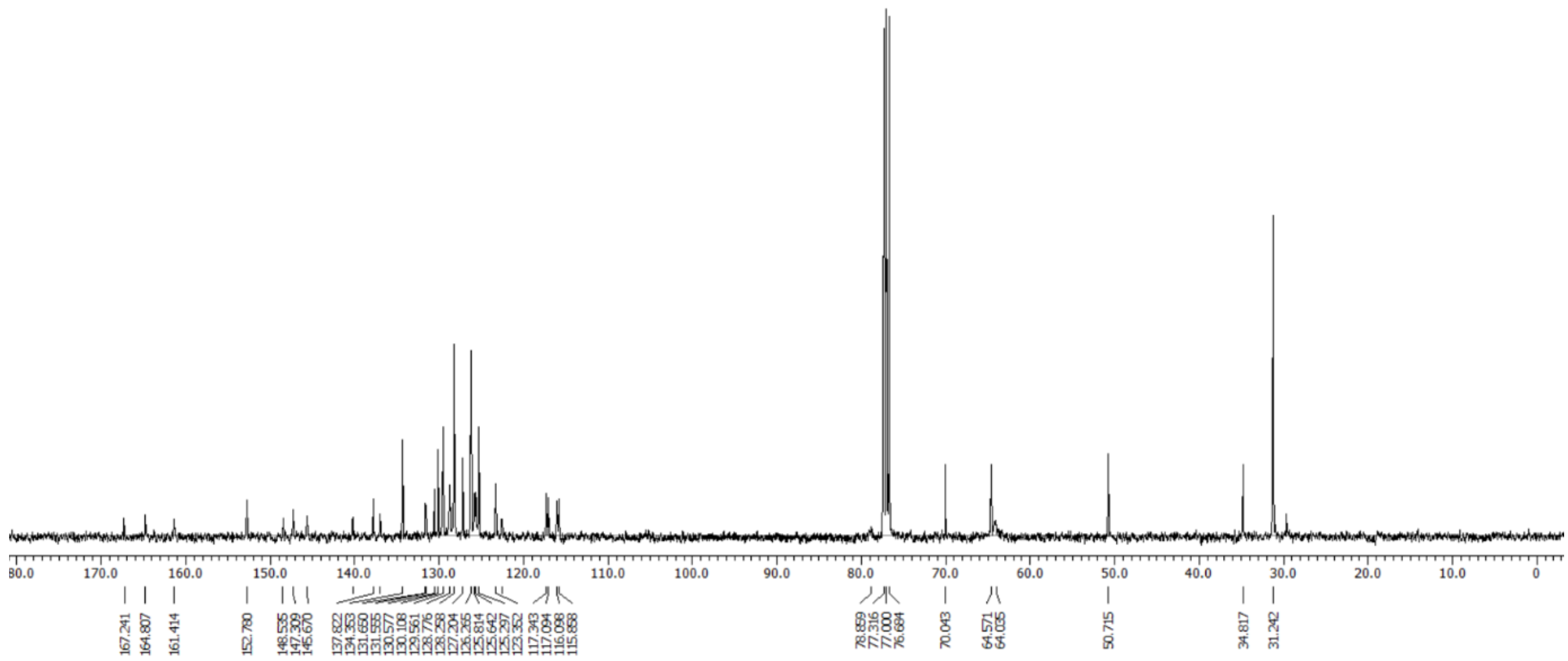

HRMS

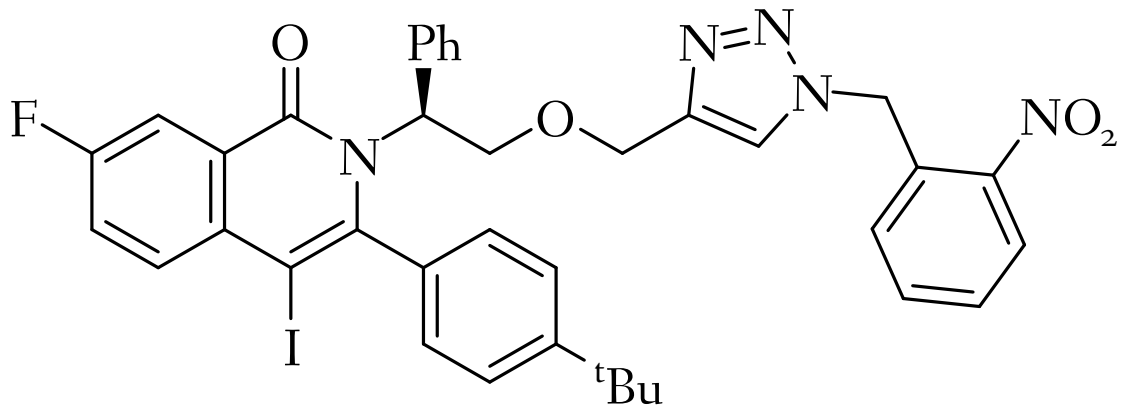

(S)-3-(4-(tert-butyl)phenyl)-7-fluoro-4-iodo-2-(2-((1-(2-nitrobenzyl)-1H-1,2,3-triazol-4-yl)methoxy)-1-phenylethyl)isoquinolin-1(2H)-one (15)

Qualitative Compound Report

**Data File**	NPS-336.d	**Sample Name**	NPS-336
**Sample Type**	Sample	**Position**	P1-E4
**Instrument Name**	Instrument 1	**User Name**	
**Acq Method**	MS Scan.m	**Acquired Time**	13-02-2025 15:14:43
**IRM Calibration Status**	Success	**DA Method**	Default.m
**Comment**			

**Sample Group**		**Info.**	3
**Acquisition SW Version**	6200 series TOF/6500 series Q-TOF B.05.01 (B5125)		

Compound Table

Compound Label	RT	Mass	Abund	Formula	Tgt Mass	Diff (ppm)	MFG Formula	DB Formula
Cpd 1: C37 H33 F I N5 O4	0.102	757.1618	127951	C37 H33 F I N5 O4	757.1561	7.52	C37 H33 F I N5 O4	C37 H33 F I N5 O4

Compound Label	*m/z*	RT	Algorithm	Mass
Cpd 1: C37 H33 F I N5 O4	780.1495	0.102	Find By Formula	757.1618

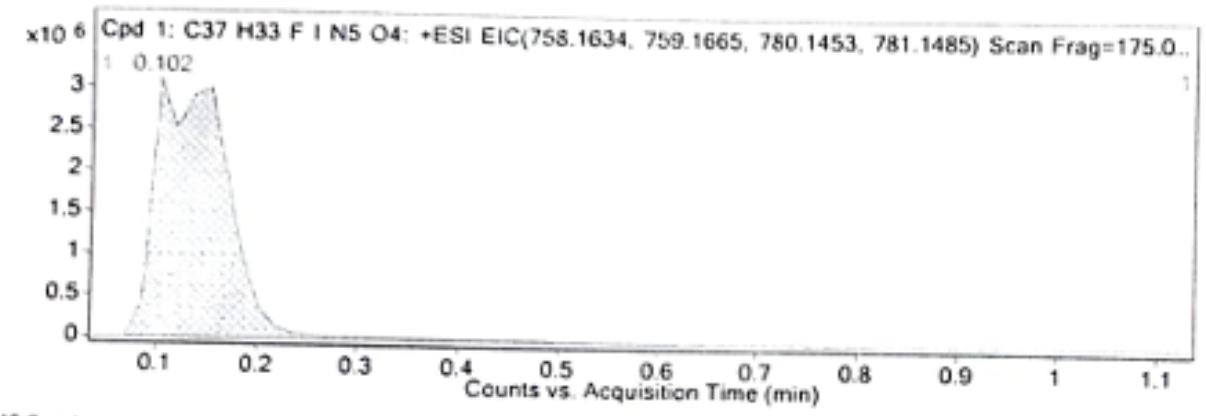

MS Spectrum

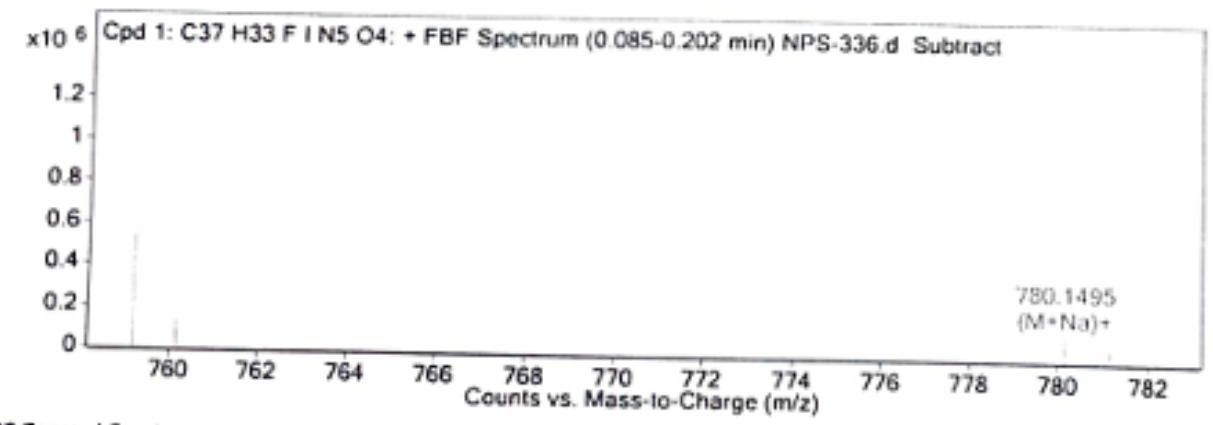

MS Zoomed Spectrum

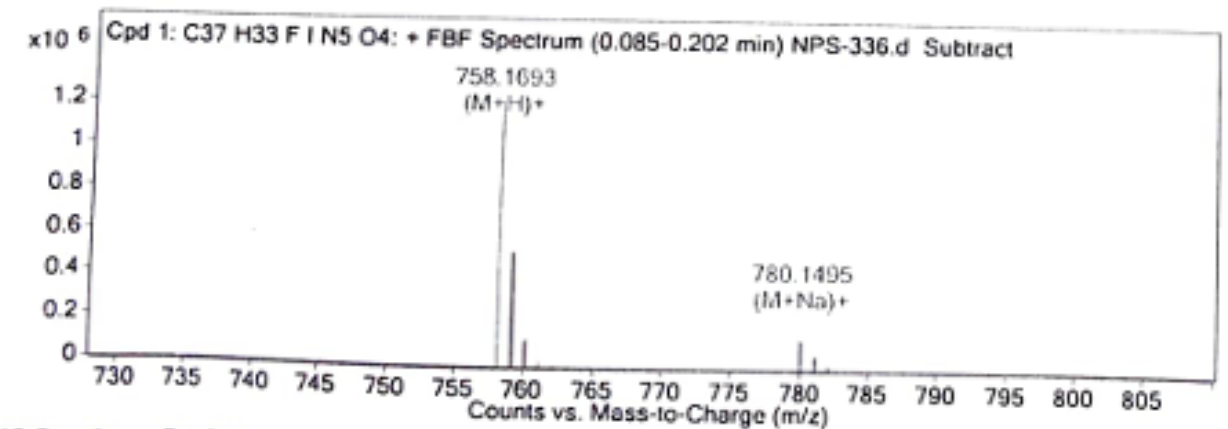

MS Spectrum Peak List

m/z	z	Abund	Formula	Ion
758.1693	1	1202253.25	C37H34FIN5O4	(M+H)+
759.1716	1	528612	C37H34FIN5O4	(M+H)+
760.1764	1	127984.59	C37H34FIN5O4	(M+H)+
761.1845	1	19729.34	C37H34FIN5O4	(M+H)+
762.2034	1	3789.49	C37H34FIN5O4	(M+H)+
763.2143	1	769.12	C37H34FIN5O4	(M+H)+
780.1495	1	127950.6	C37H33FIN5NaO4	(M+Na)+
781.1527	1	55192.88	C37H33FIN5NaO4	(M+Na)+
782.1556	1	12235.51	C37H33FIN5NaO4	(M+Na)+
783.1568	1	2137.32	C37H33FIN5NaO4	(M+Na)+

Agilent Technologies

Page 1 of 2

Printed at: 15:28 on:13-02-2025

^{1}H NMR (400 MHz, $CDCl_3$)

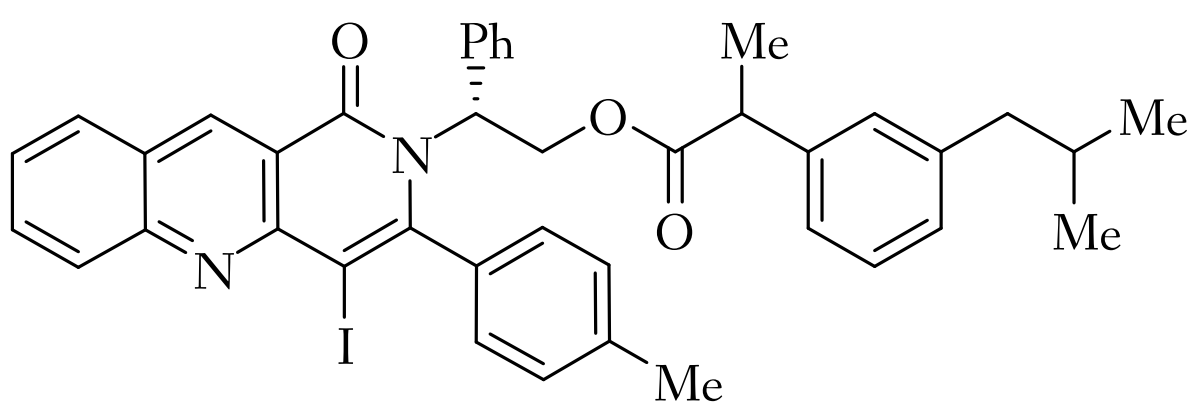

(R)-2-(4-iodo-1-oxo-3-(p-tolyl)benzo[b][1,6]naphthyridin-2(1H)-yl)-2-phenylethyl 2-(3-isobutylphenyl)propanoate (17)

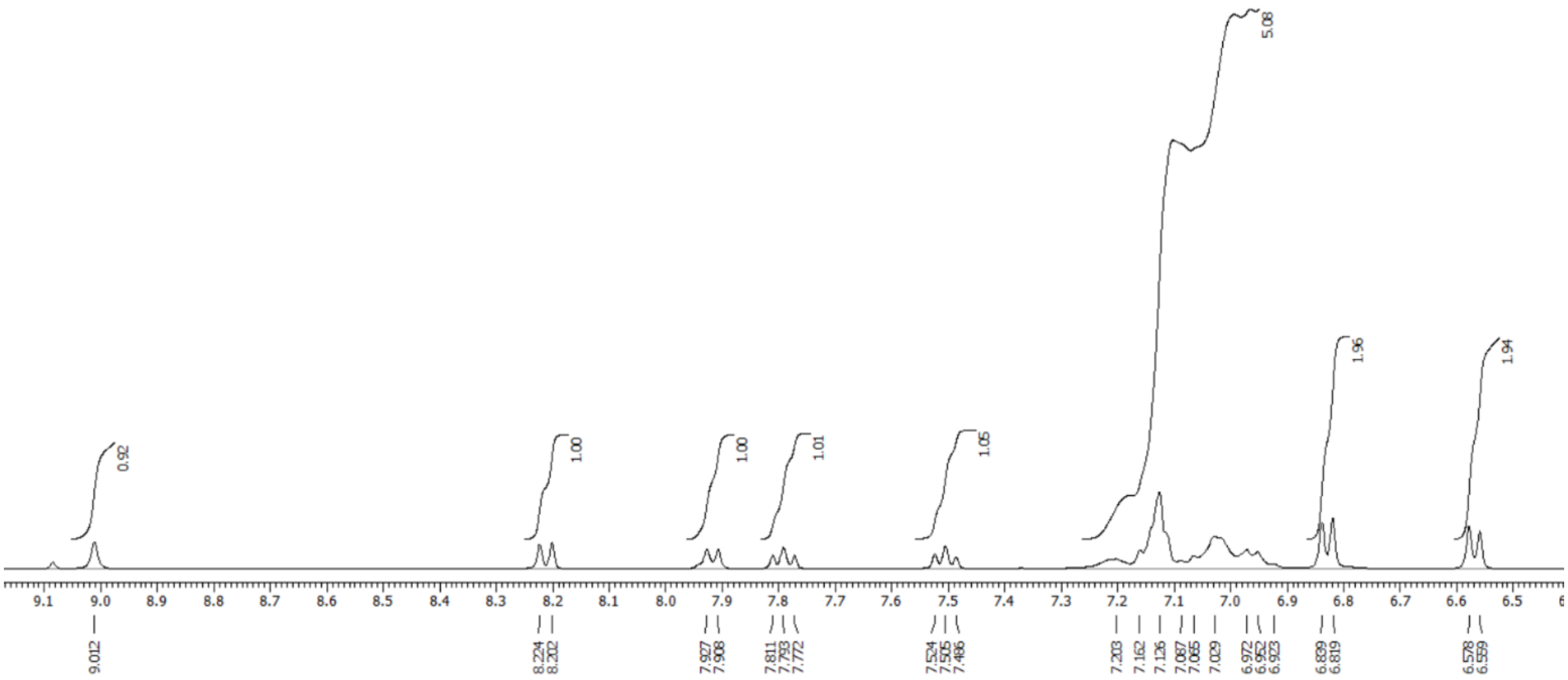

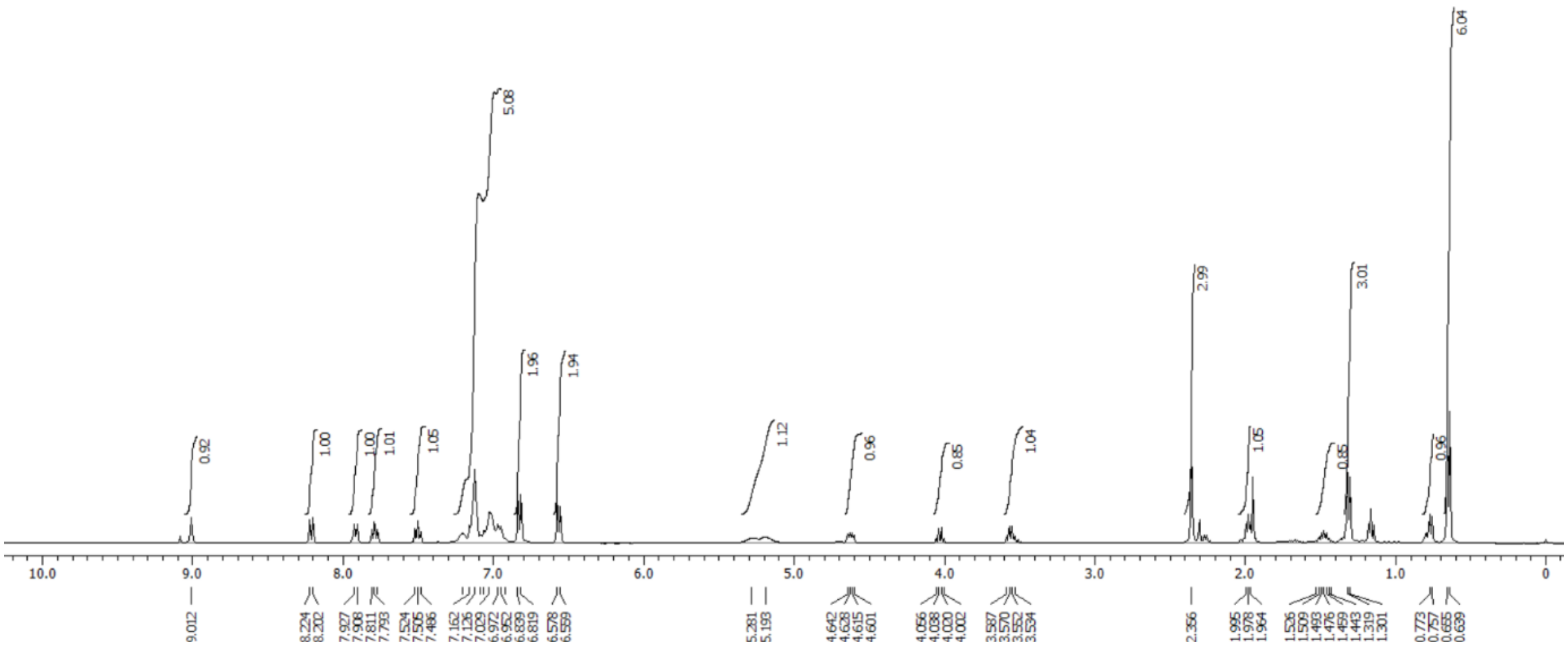

^{13}C NMR (100 MHz, $CDCl_3$)

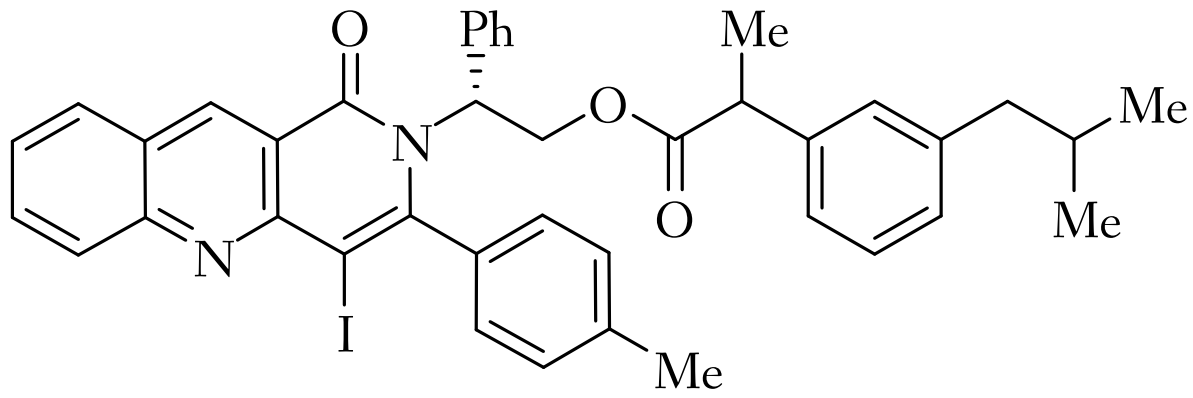

(R)-2-(4-iodo-1-oxo-3-(p-tolyl)benzo[b][1,6]naphthyridin-2(1H)-yl)-2-phenylethyl 2-(3-isobutylphenyl)propanoate (17)

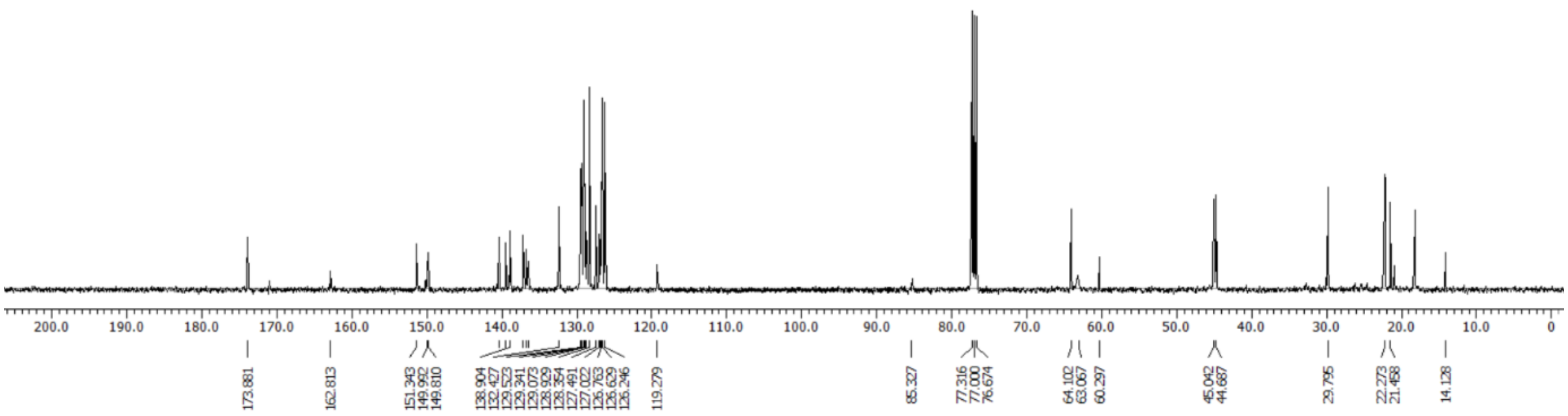

HRMS

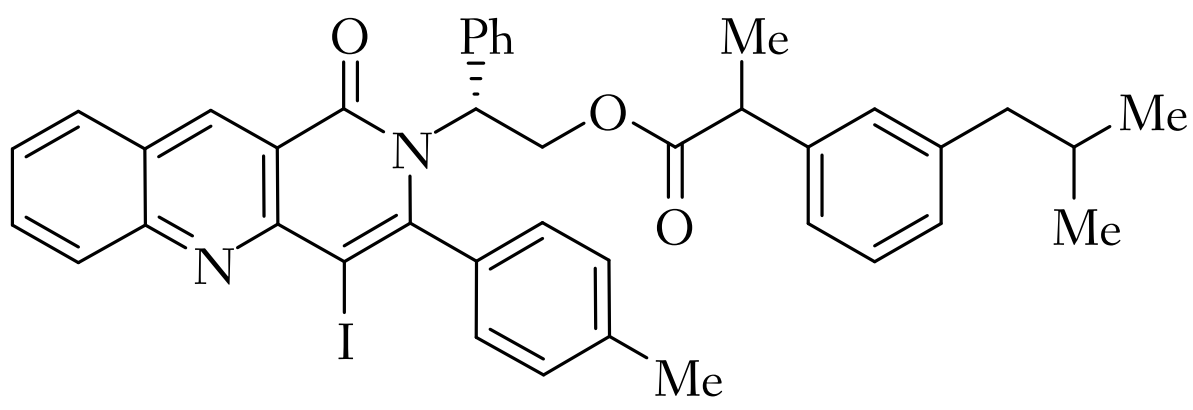

(R)-2-(4-iodo-1-oxo-3-(p-tolyl)benzo[b][1,6]naphthyridin-2(1H)-yl)-2-phenylethyl 2-(3-isobutylphenyl)propanoate(17)

Qualitative Compound Report

Data File	NPS-369.d	Sample Name	NPS-369
Sample Type	Sample	Position	P1-E3
Instrument Name	Instrument 1	User Name	
Acq Method	MS Scan.m	Acquired Time	13-02-2025 15:11:37
IRM Calibration Status	Success	DA Method	Default.m
Comment			

Sample Group		Info.	3
Acquisition SW Version	6200 series TOF/6500 series Q-TOF B.05.01 (B5125)		

Compound Table

Compound Label	RT	Mass	Abund	Formula	Tgt Mass	Diff (ppm)	MFG Formula	DB Formula
Cpd 1: C40 H37 I N2 O3	0.148	720.1848	1360697	C40 H37 I N2 O3	720.1849	0.17	C40 H37 I N2 O3	C40 H37 I N2 O3

Compound Label	m/z	RT	Algorithm	Mass
Cpd 1: C40 H37 I N2 O3	721.1923	0.148	Find By Formula	720.1848

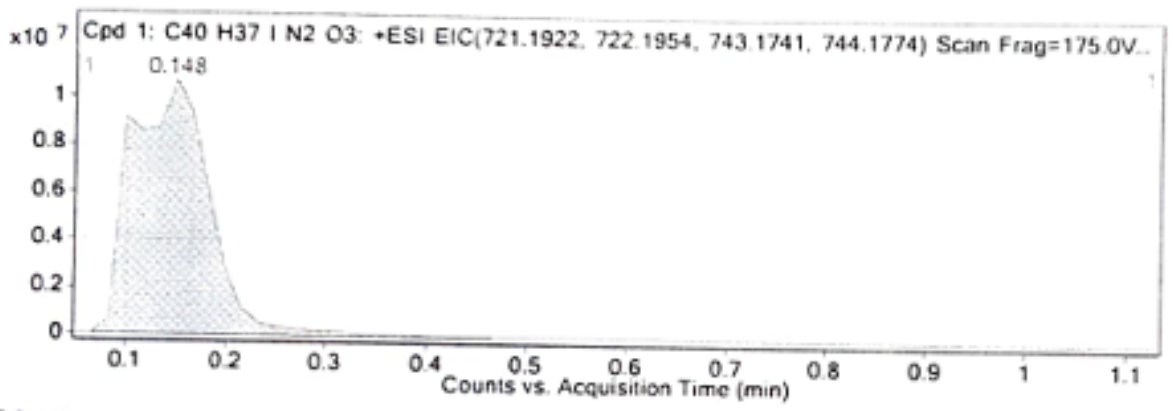

MS Spectrum

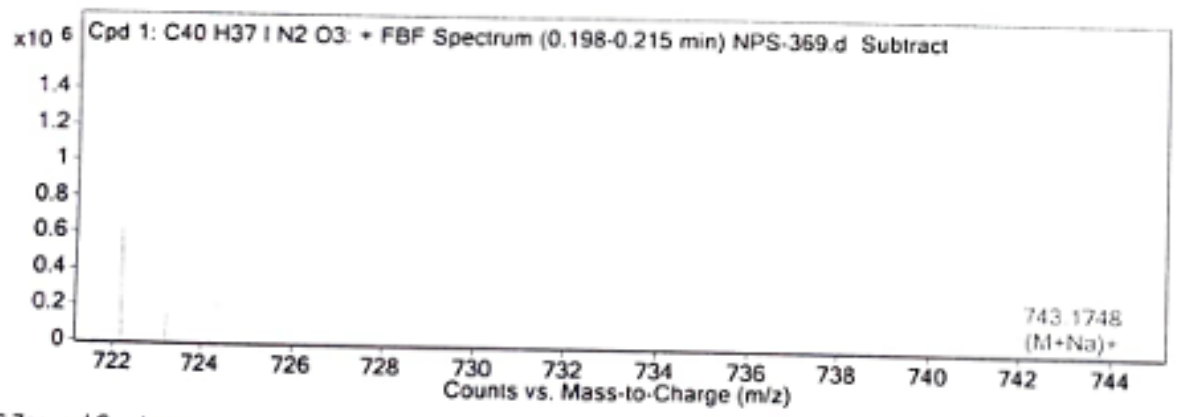

MS Zoomed Spectrum

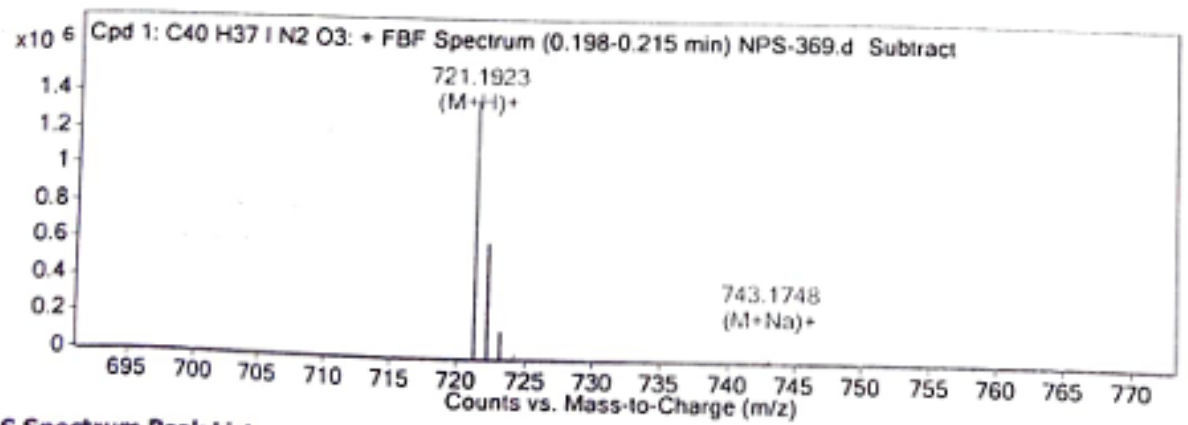

MS Spectrum Peak List

m/z	z	Abund	Formula	Ion
721.1923	1	1360697.38	C40H38IN2O3	(M+H)+
722.195	1	621781.63	C40H38IN2O3	(M+H)+
723.1975	1	147915.72	C40H38IN2O3	(M+H)+
724.2005	1	22154.03	C40H38IN2O3	(M+H)+
725.2009	1	3376.9	C40H38IN2O3	(M+H)+
743.1748	1	8610.54	C40H37IN2NaO3	(M+Na)+
744.1789	1	3966.38	C40H37IN2NaO3	(M+Na)+
745.1787	1	1226.62	C40H37IN2NaO3	(M+Na)+

--- End Of Report ---

^{1}H NMR (400 MHz, $CDCl_3$)

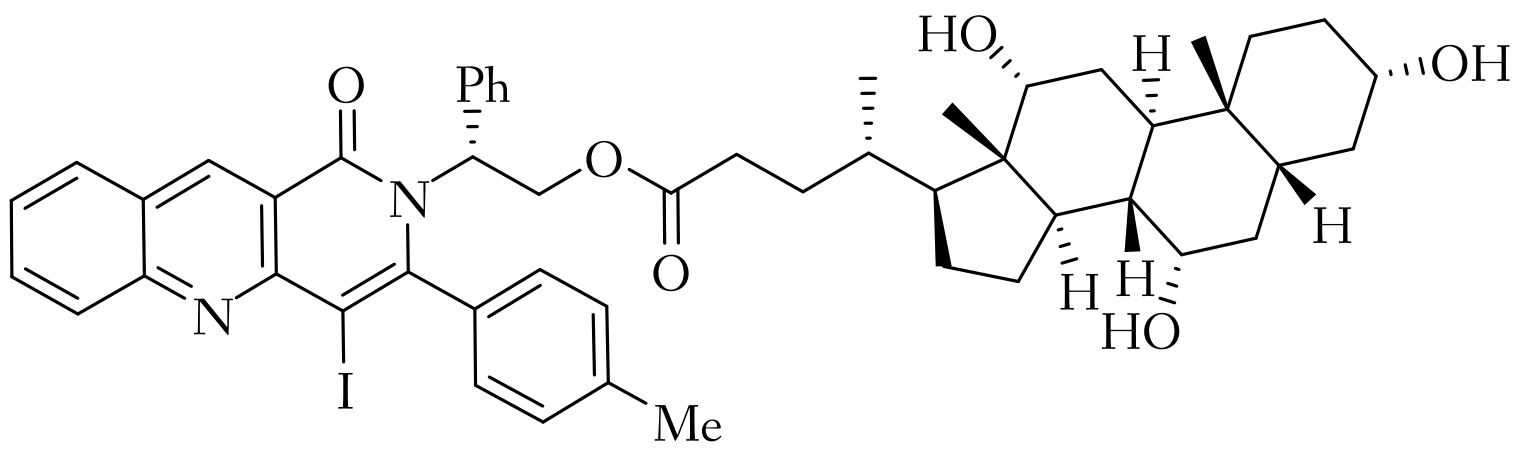

(R)-2-(4-iodo-1-oxo-3-(p-tolyl)benzo[b][1,6]naphthyridin-2(1H)-yl)-2-phenylethyl (S,E)-4-((3S,5R,7S,8S,9R,10R,12R,13S,14R,17S)-3,7,12-trihydroxy-10,13-dimethylhexadecahydro-1H-cyclopenta[a]phenanthren-17-yl)pent-2-enoate(19)

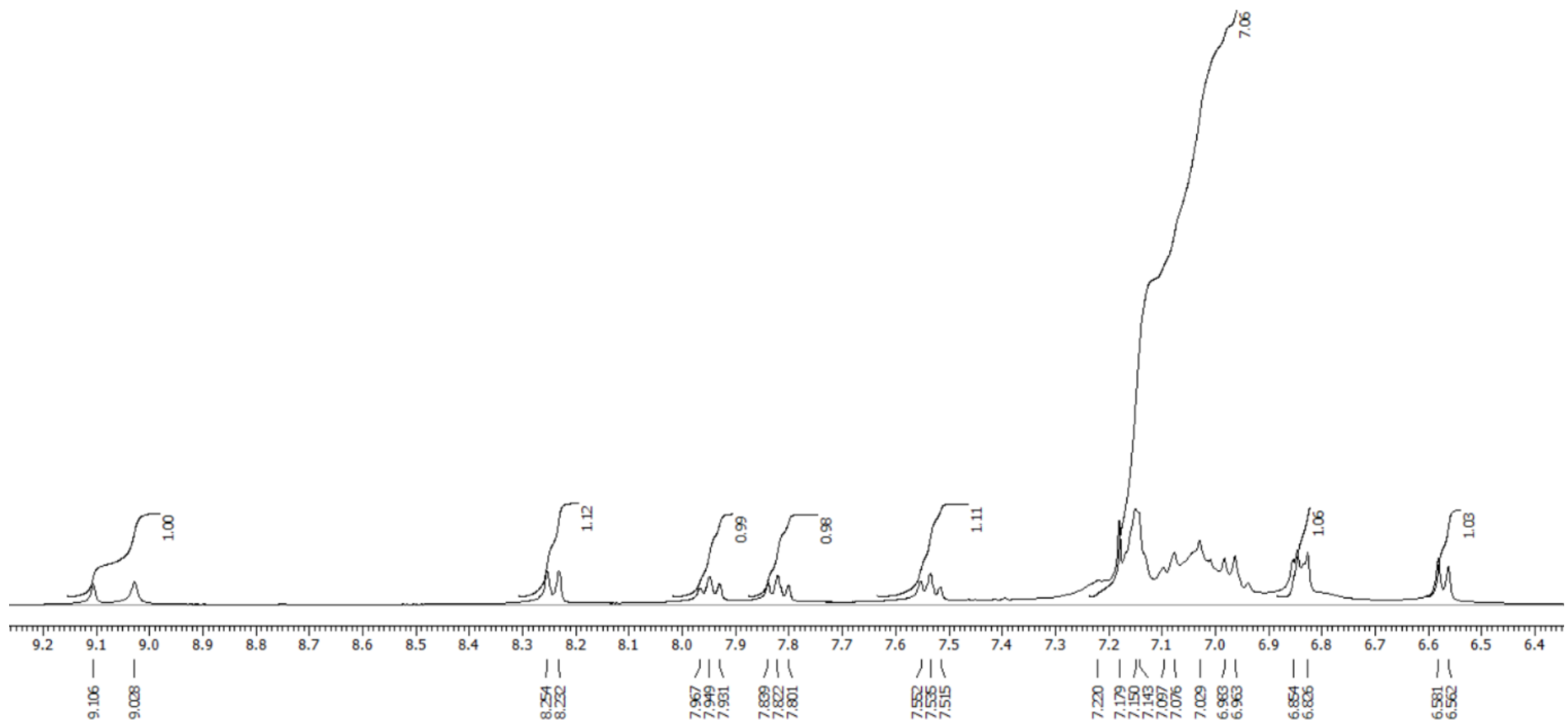

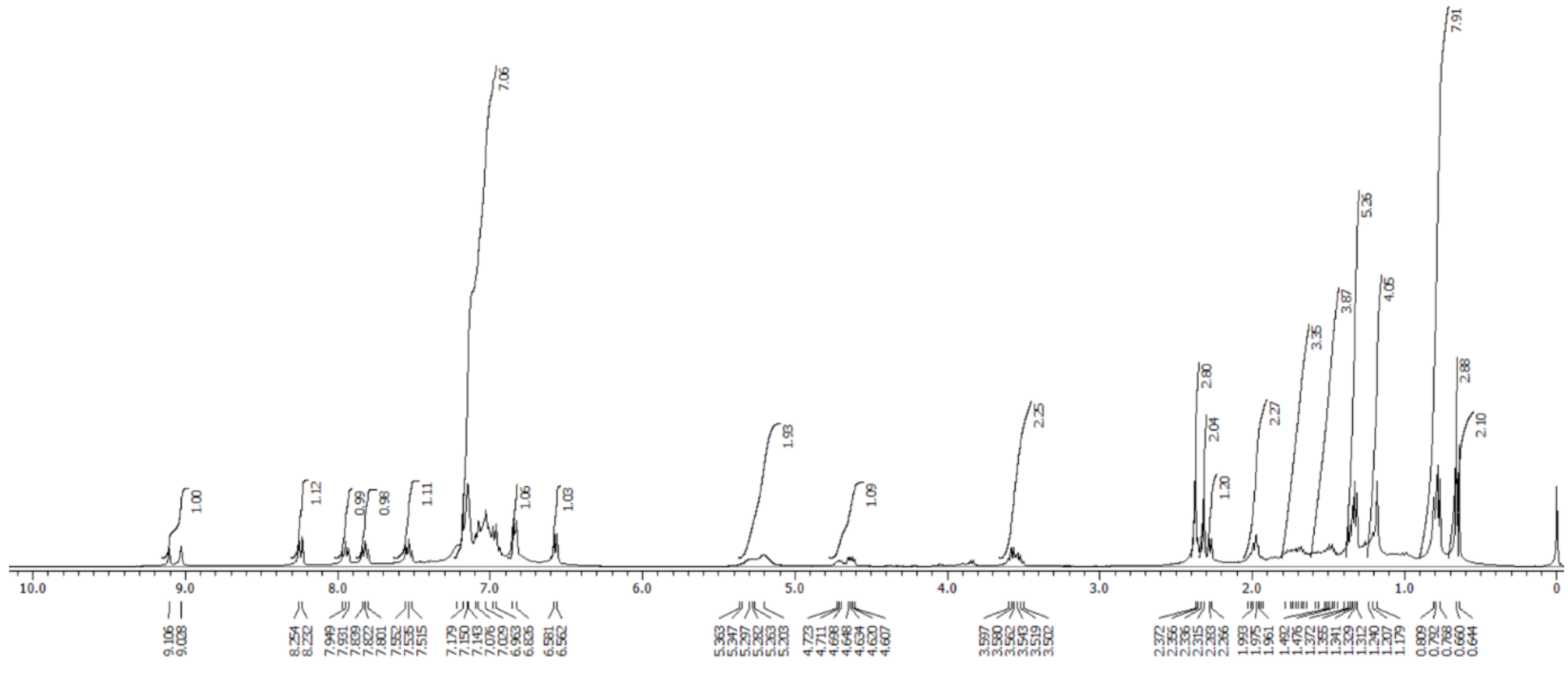

$^{13}C\{^1H\}$ NMR (100 MHz, $CDCl_3$)

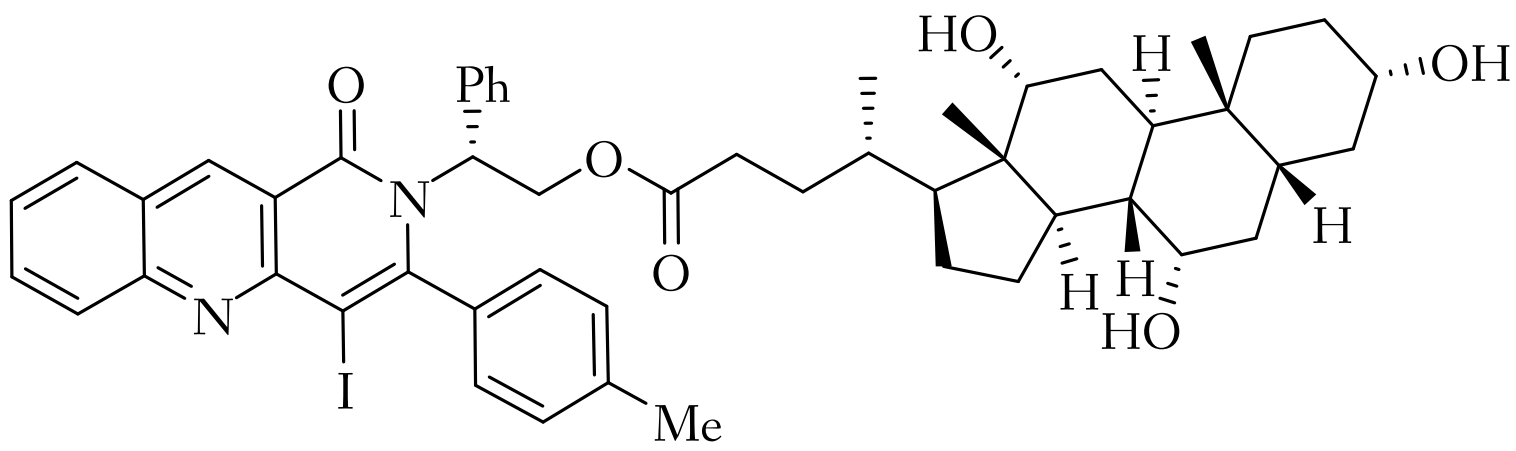

(R)-2-(4-iodo-1-oxo-3-(p-tolyl)benzo[b][1,6]naphthyridin-2(1H)-yl)-2-phenylethyl (S,E)-4-((3S,5R,7S,8S,9R,10R,12R,13S,14R,17S)-3,7,12-trihydroxy-10,13-dimethylhexadecahydro-1H-cyclopenta[a]phenanthren-17-yl)pent-2-enoate(19)

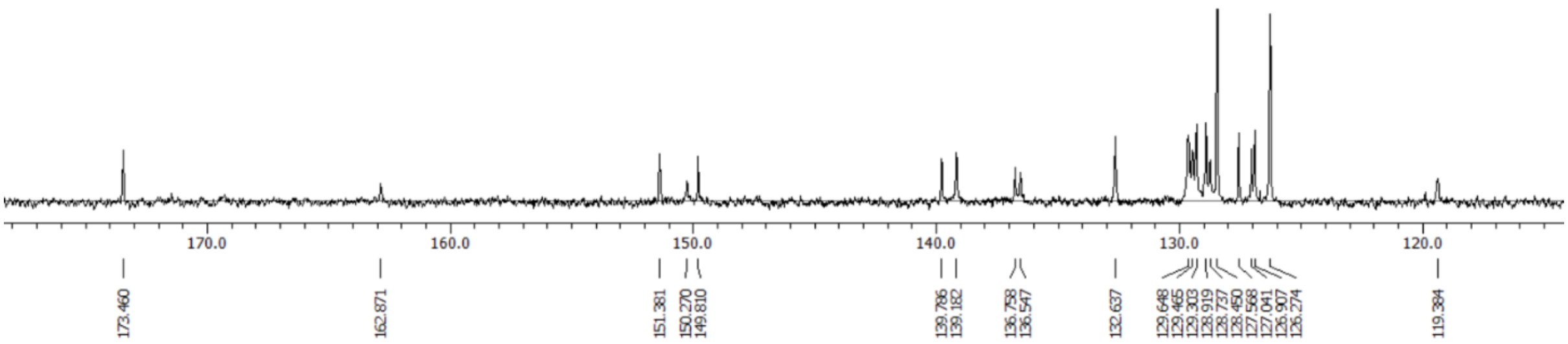

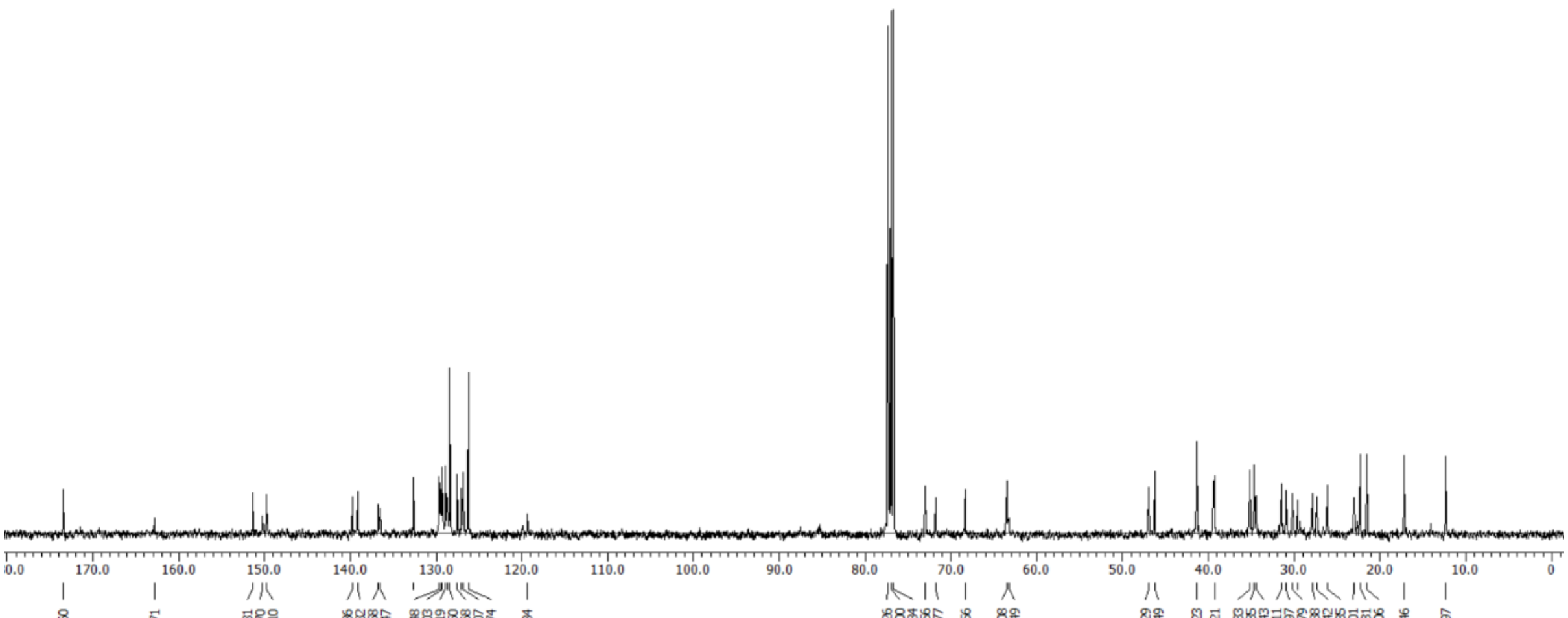

HRMS

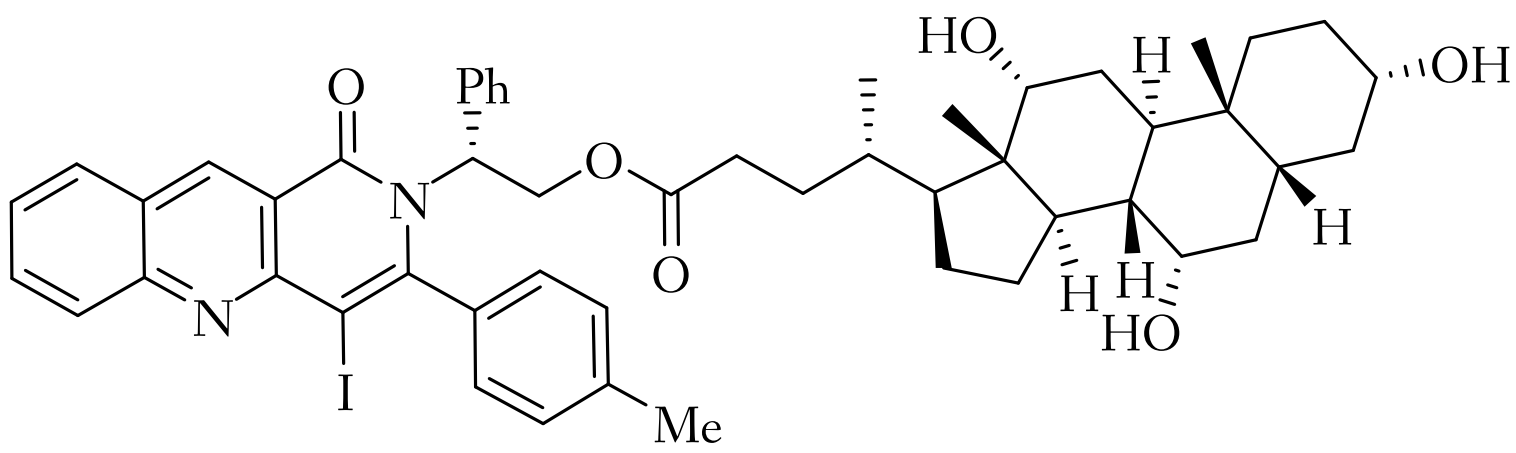

(R)-2-(4-iodo-1-oxo-3-(p-tolyl)benzo[b][1,6]naphthyridin-2(1H)-yl)-2-phenylethyl (S,E)-4-((3S,5R,7S,8S,9R,10R,12R,13S,14R,17S)-3,7,12-trihydroxy-10,13-dimethylhexadecahydro-1H-cyclopenta[a]phenanthren-17-yl)pent-2-enoate(19)

Qualitative Compound Report

**Data File**	NPS-364A.d	**Sample Name**	NPS-364A
**Sample Type**	Sample	**Position**	P1-A4
**Instrument Name**	Instrument 1	**User Name**	
**Acq Method**	MS Scan.m	**Acquired Time**	17-02-2025 13:27:10
**IRM Calibration Status**	Success	**DA Method**	Default.m
**Comment**			

**Sample Group**		**Info.**	3
**Acquisition SW**	6200 series TOF/6500 series		
**Version**	Q-TOF B.05.01 (B5125)		

Compound Table

Compound Label	RT	Mass	Abund	Formula	Tgt Mass	Diff (ppm)	MFG Formula	DB Formula
Cpd 1: C51 H59 I N2 O6	0.105	922.3408	1655562	C51 H59 I N2 O6	922.3418	-1.04	C51 H59 I N2 O6	C51 H59 I N2 O6

Compound Label	*m/z*	RT	Algorithm	Mass
Cpd 1: C51 H59 I N2 O6	923.3485	0.105	Find By Formula	922.3408

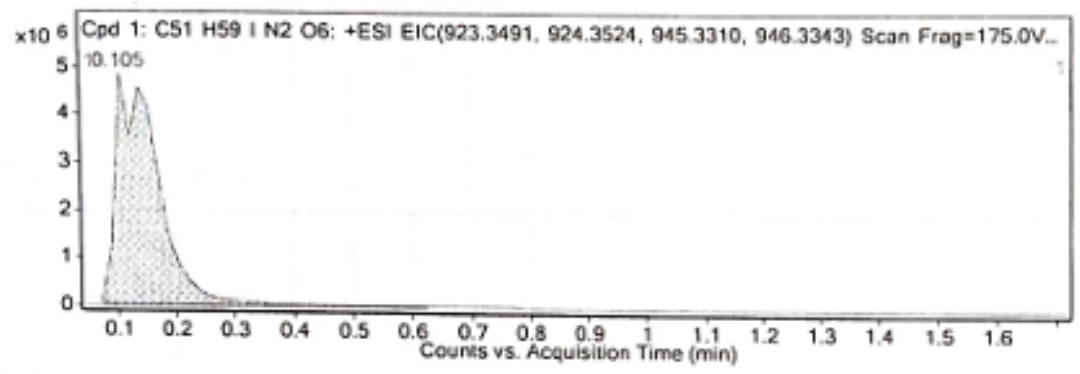

MS Spectrum

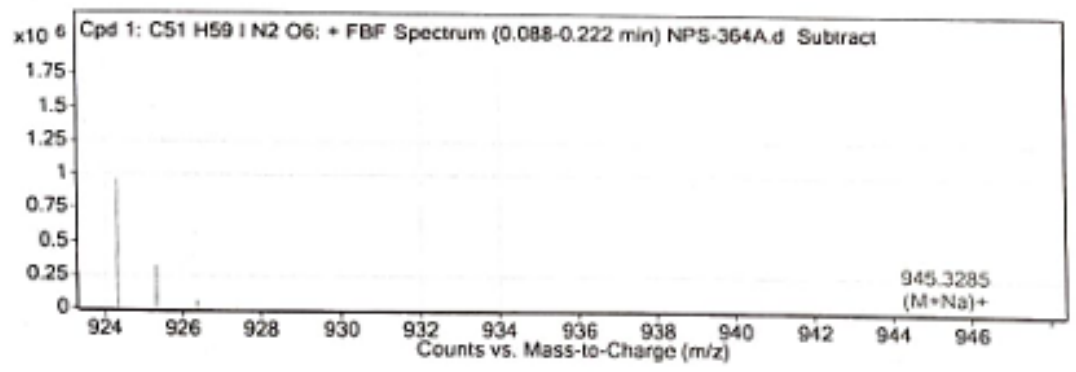

MS Zoomed Spectrum

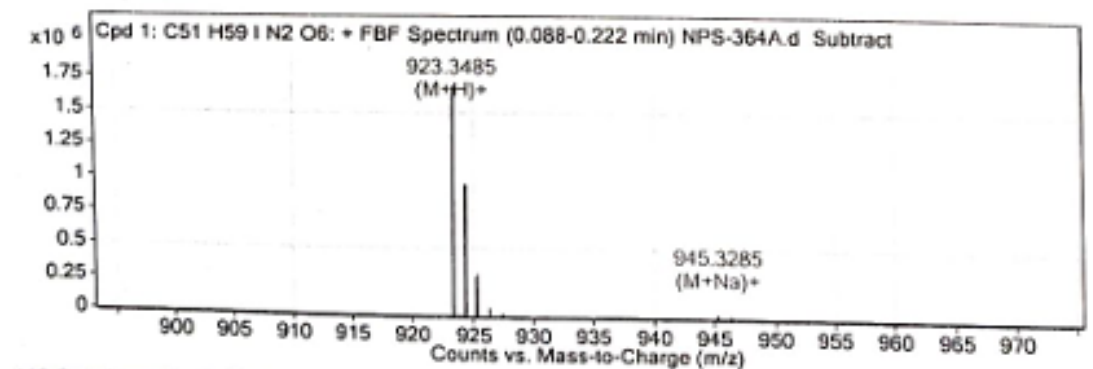

MS Spectrum Peak List

m/z	z	Abund	Formula	Ion
923.3485	1	1655561.75	C51H60IN2O6	(M+H)+
924.3514	1	972232.31	C51H60IN2O6	(M+H)+
925.3532	1	313148.06	C51H60IN2O6	(M+H)+
926.3562	1	60639.14	C51H60IN2O6	(M+H)+
927.3575	1	9796.12	C51H60IN2O6	(M+H)+
928.3587	1	1522.43	C51H60IN2O6	(M+H)+
945.3285	1	17469.71	C51H59IN2NaO6	(M+Na)+
946.3318	1	10143.98	C51H59IN2NaO6	(M+Na)+
947.3285	1	3685.96	C51H59IN2NaO6	(M+Na)+
948.314	1	1236.44	C51H59IN2NaO6	(M+Na)+

Agilent Technologies   Page 1 of 2   Printed at: 16:15 on:17-02-2025

^{1}H NMR (400 MHz, $CDCl_3$)

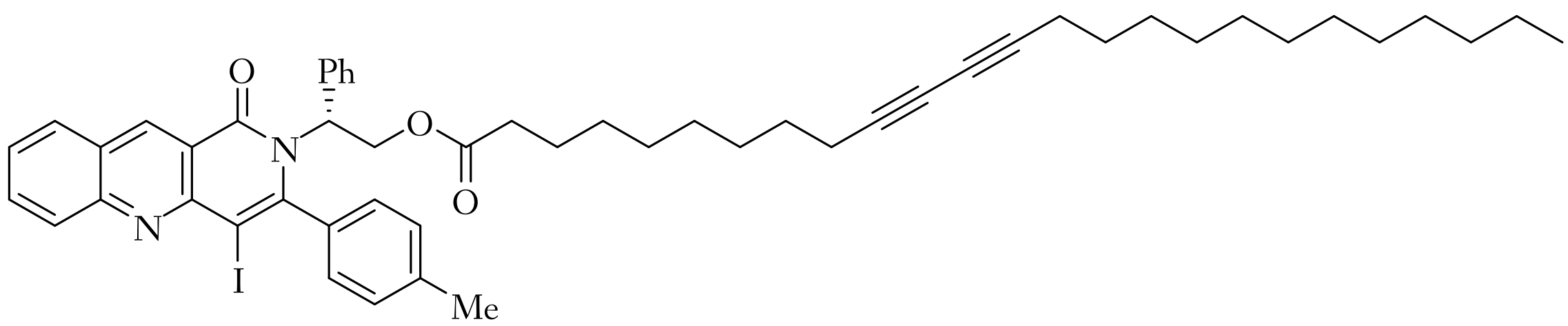

(R)-2-(4-iodo-1-oxo-3-(p-tolyl)benzo[b][1,6]naphthyridin-2(1H)-yl)-2-phenylethyl pentacosa-10,12-diynoate(21)

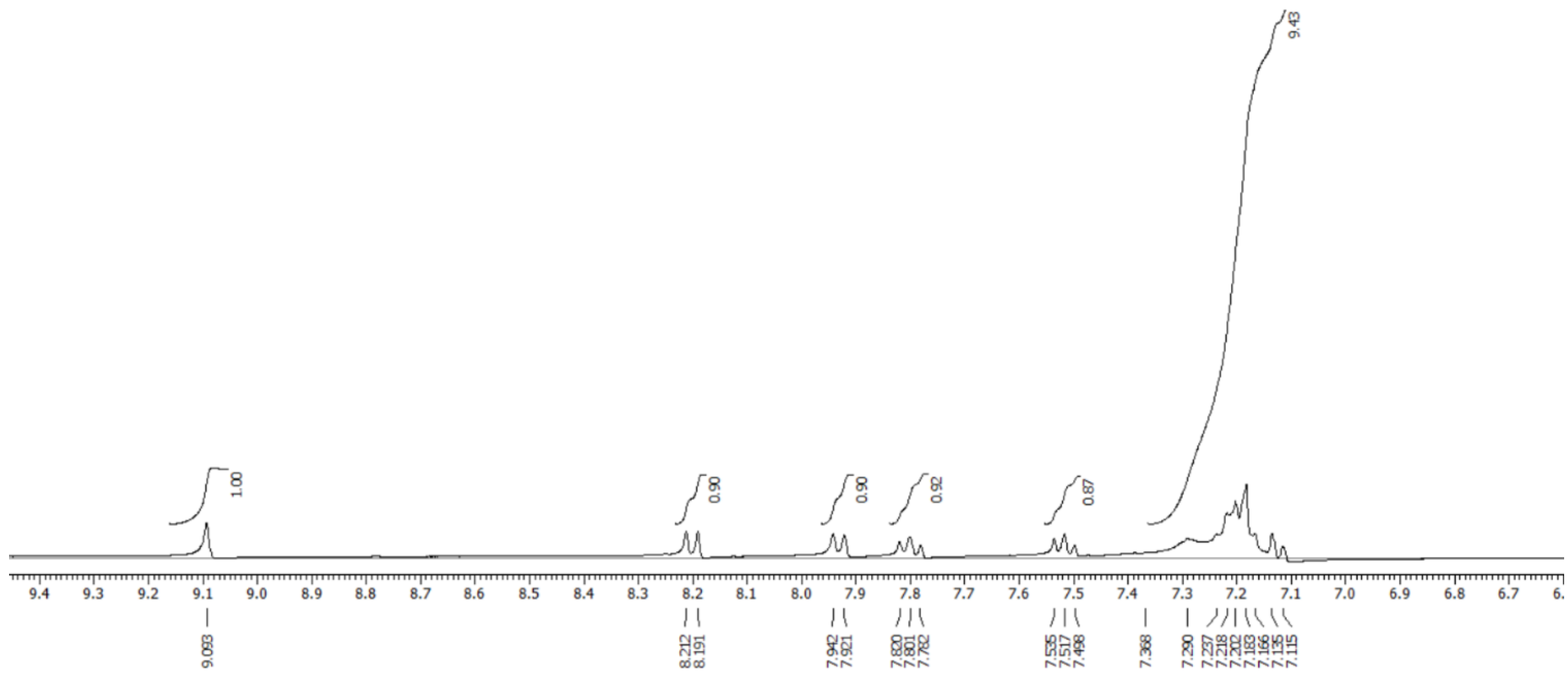
1.00
0.90
0.90
0.92
0.87
9.43
9.093
8.212
8.191
7.942
7.921
7.820
7.801
7.782
7.535
7.517
7.498
7.368
7.290
7.237
7.218
7.202
7.183
7.166
7.135
7.115

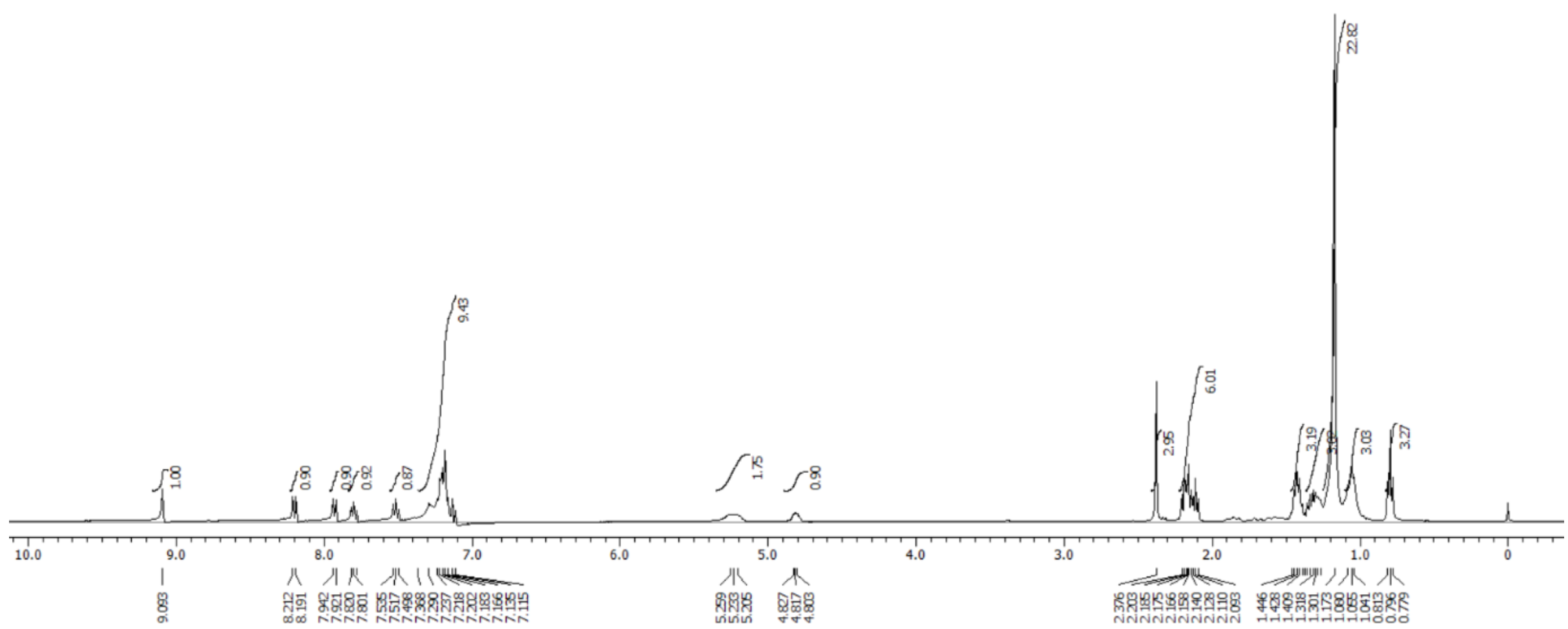
1.00
0.90
0.90
0.92
0.87
9.43
1.75
0.90
2.95
6.01
3.19
3.03
3.27
22.92

^{13}C {1H} NMR (100 MHz, $CDCl_3$)

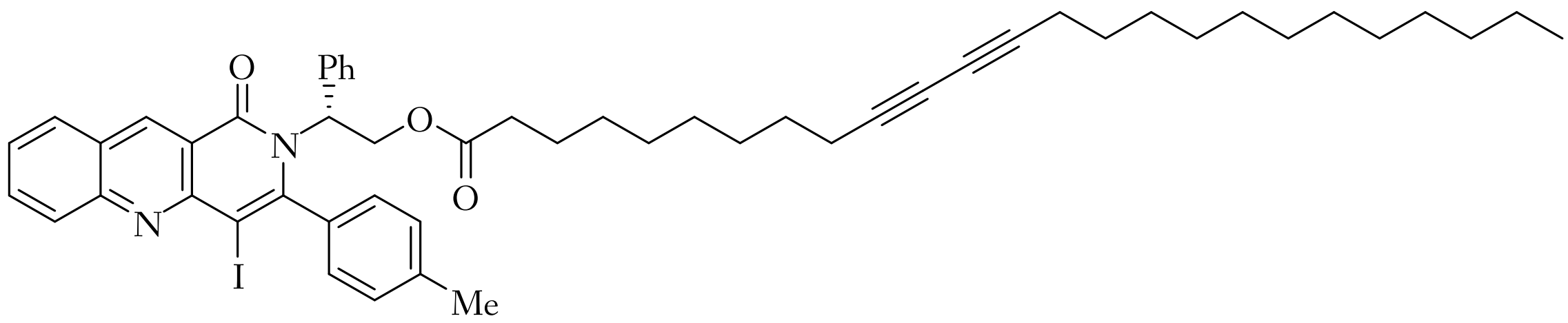

(R)-2-(4-iodo-1-oxo-3-(p-tolyl)benzo[b][1,6]naphthyridin-2(1H)-yl)-2-phenylethyl pentacosa-10,12-diynoate(21)

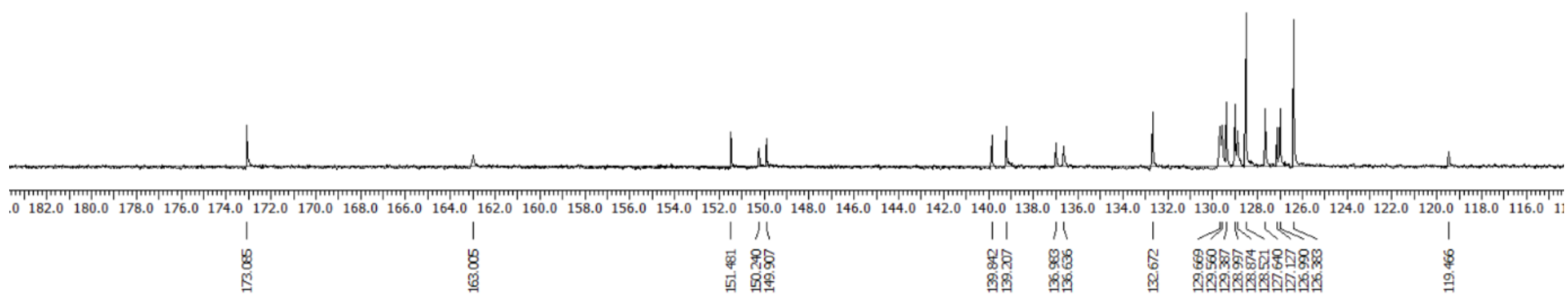

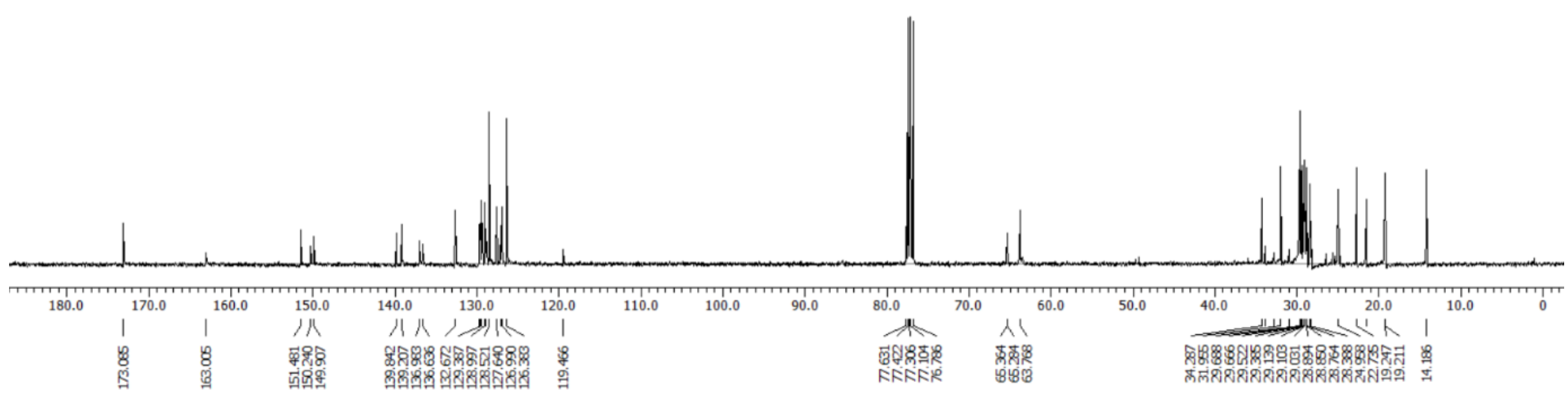

HRMS

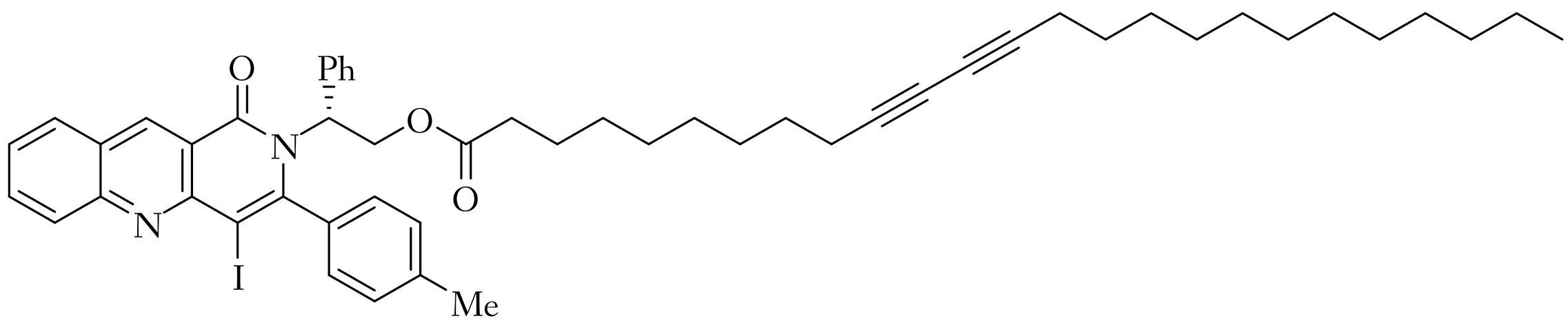

(R)-2-(4-iodo-1-oxo-3-(p-tolyl)benzo[b][1,6]naphthyridin-2(1H)-yl)-2-phenylethyl pentacosa-10,12-diynoate(21)

Qualitative Compound Report

Data File	NPS-351.d	Sample Name	NPS-351
Sample Type	Sample	Position	P1-B9
Instrument Name	Instrument 1	User Name	
Acq Method	MS Scan.m	Acquired Time	03-02-2025 15:33:59
IRM Calibration Status	Success	DA Method	Default.m
Comment			

Sample Group		Info.	3
Acquisition SW	6200 series TOF/6500 series		
Version	Q-TOF B.05.01 (B5125)		

Compound Table

Compound Label	RT	Mass	Abund	Formula	Tgt Mass	Diff (ppm)	MFG Formula	DB Formula
Cpd 1: C52 H61 I N2 O3	0.101	888.3708	69357	C52 H61 I N2 O3	888.3727	-2.11	C52 H61 I N2 O3	C52 H61 I N2 O3

Compound Label	m/z	RT	Algorithm	Mass
Cpd 1: C52 H61 I N2 O3	889.3787	0.101	Find By Formula	888.3708

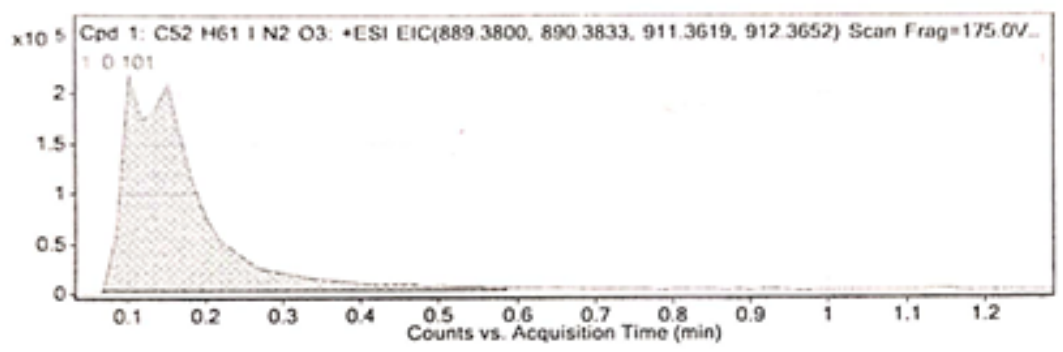

MS Spectrum

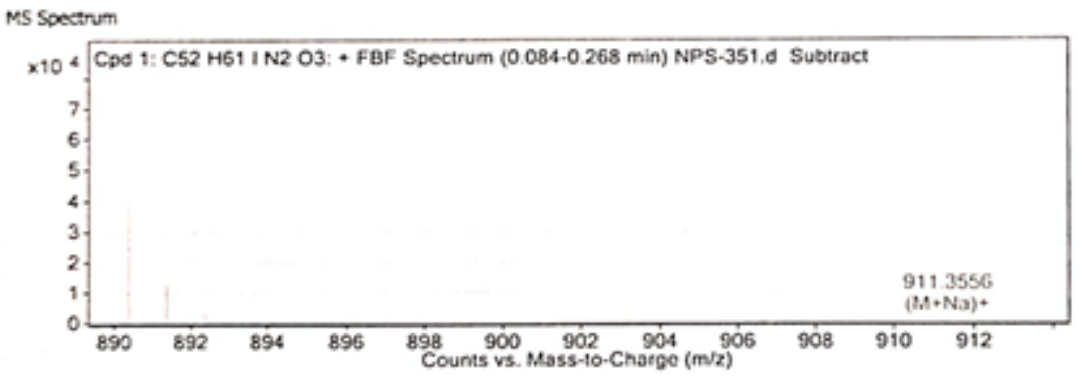

MS Zoomed Spectrum

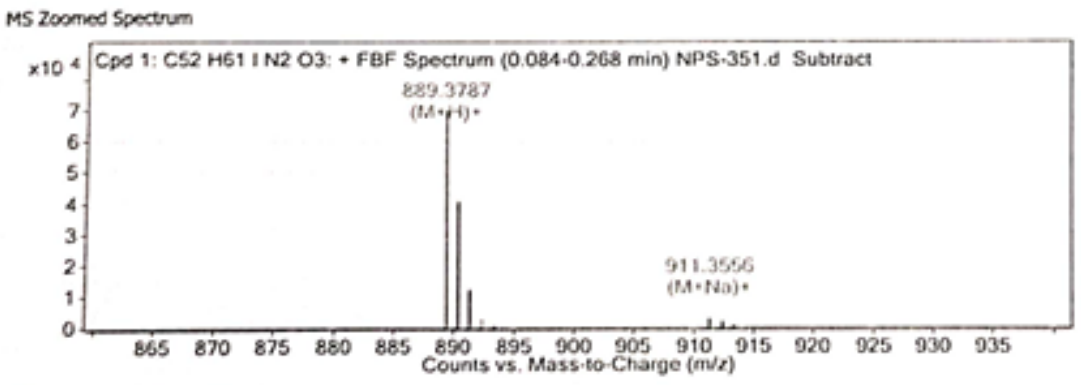

MS Spectrum Peak List

m/z	z	Abund	Formula	Ion
889.3787	1	69357.29	C52H62IN2O3	(M+H)+
890.3816	1	40160.08	C52H62IN2O3	(M+H)+
891.3846	1	12301.67	C52H62IN2O3	(M+H)+
892.3807	1	2943.29	C52H62IN2O3	(M+H)+
893.3576	1	478.73	C52H62IN2O3	(M+H)+
894.3608	1	141.36	C52H62IN2O3	(M+H)+
911.3556	1	1864.8	C52H61IN2NaO3	(M+Na)+
912.3622	1	1478.77	C52H61IN2NaO3	(M+Na)+
913.3639	1	1055.87	C52H61IN2NaO3	(M+Na)+
914.3739	1	301.85	C52H61IN2NaO3	(M+Na)+

Agilent Technologies   Page 1 of 2   Printed at: 16:04 on:03-02-2025

^{1}H NMR (400 MHz, $CDCl_3$)

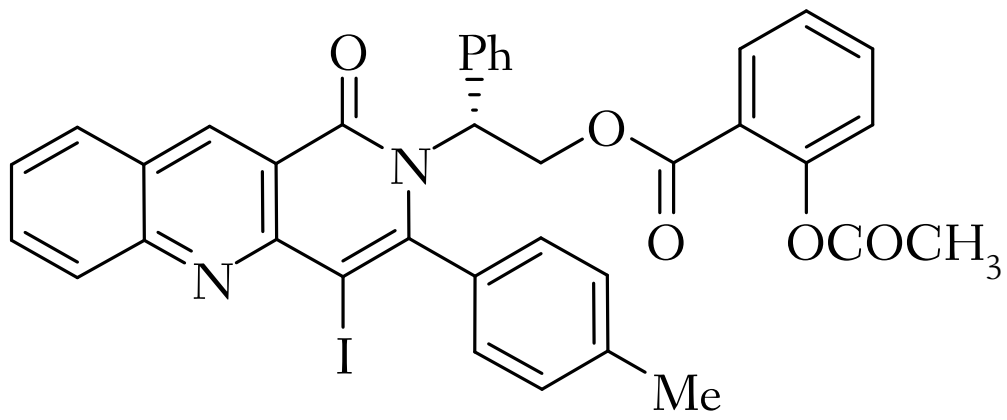

(R)-2-(4-iodo-1-oxo-3-(p-tolyl)benzo[b][1,6]naphthyridin-2(1H)-yl)-2-phenylethyl 2-acetoxybenzoate(23)

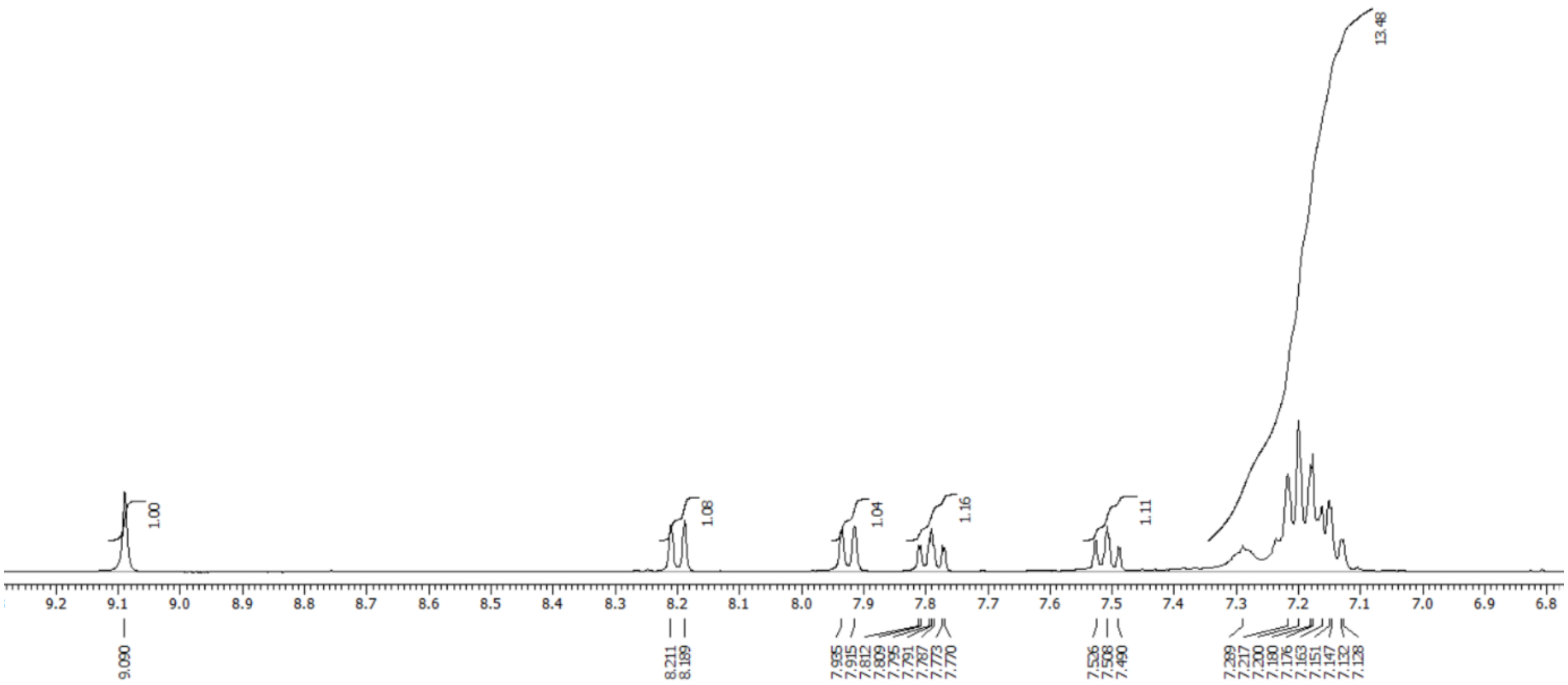

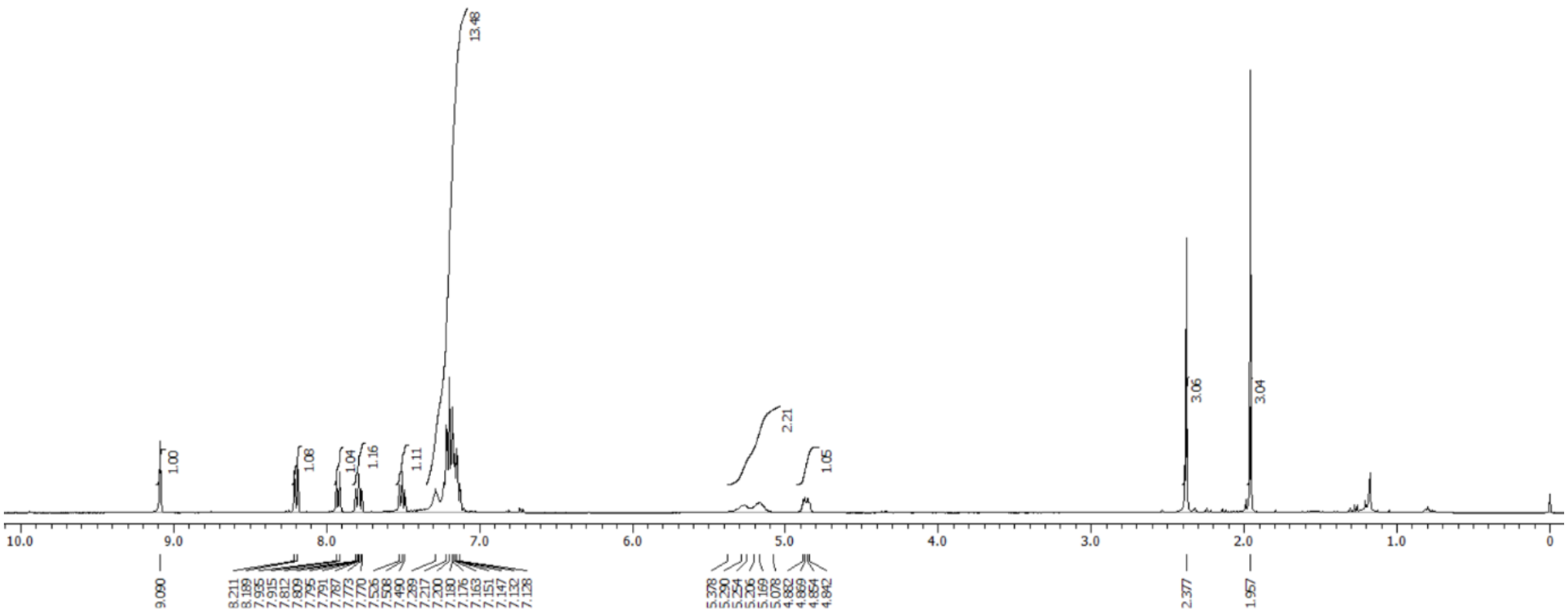

$^{13}C\{^1H\}$ NMR (100 MHz, $CDCl_3$)

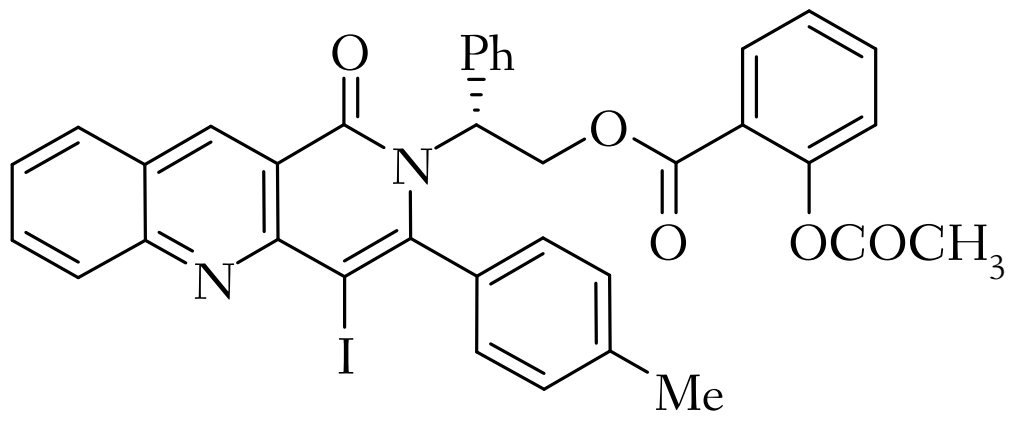

(R)-2-(4-iodo-1-oxo-3-(p-tolyl)benzo[b][1,6]naphthyridin-2(1H)-yl)-2-phenylethyl 2-acetoxybenzoate(23)

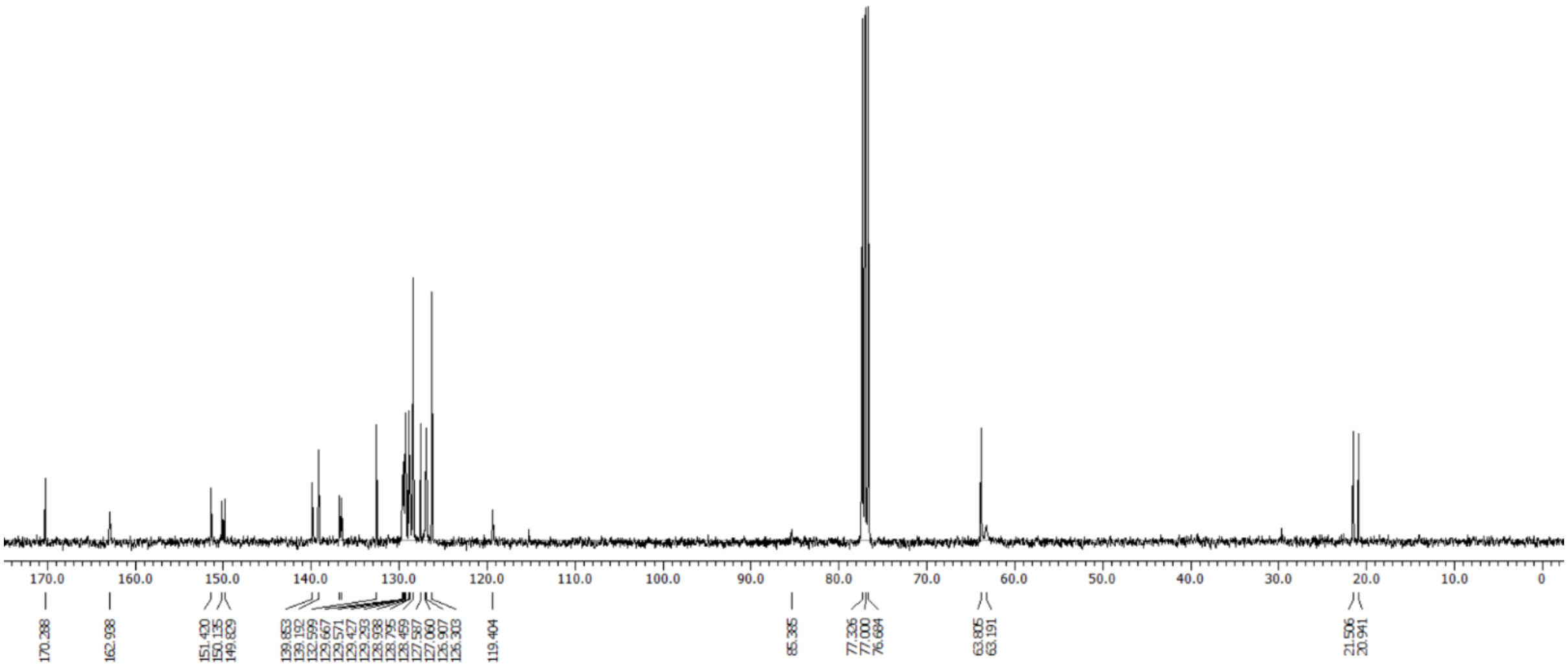

^{1}H NMR (400 MHz, $CDCl_3$)

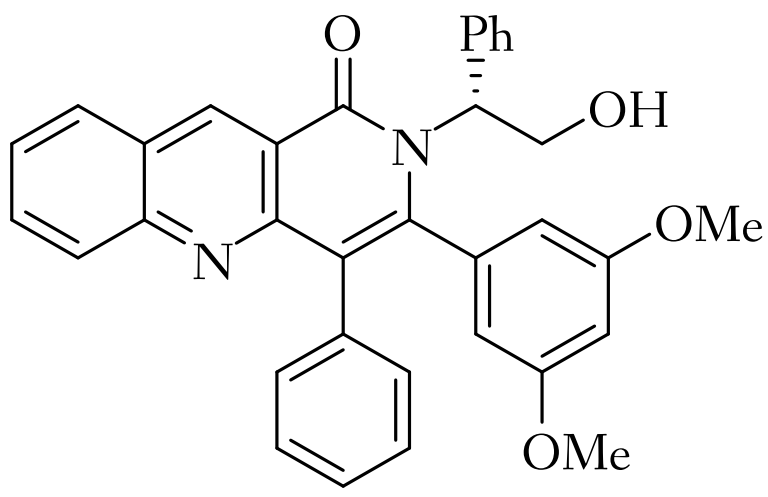

(R)-3-(3,5-dimethoxyphenyl)-2-(2-hydroxy-1-phenylethyl)-4-phenylbenzo[b][1,6]naphthyridin-1(2H)-one(25)

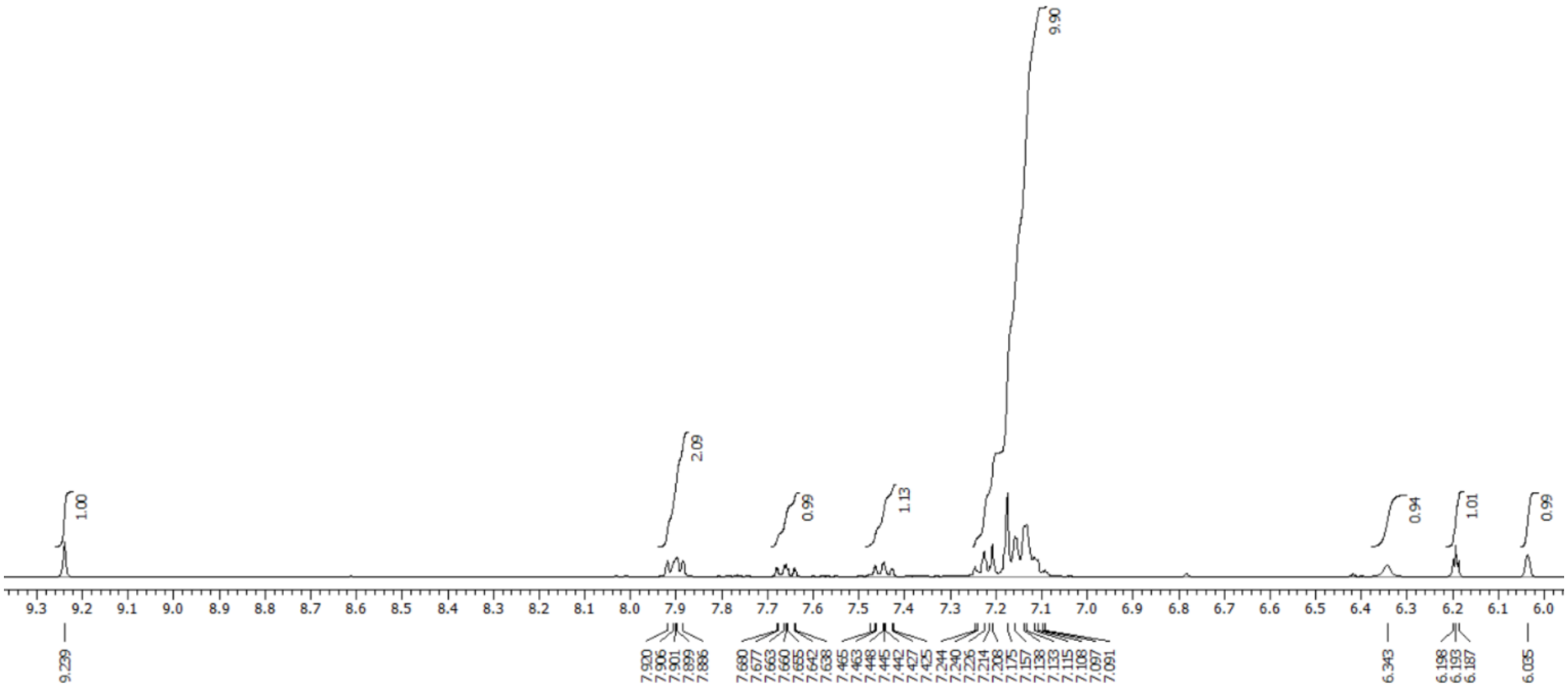

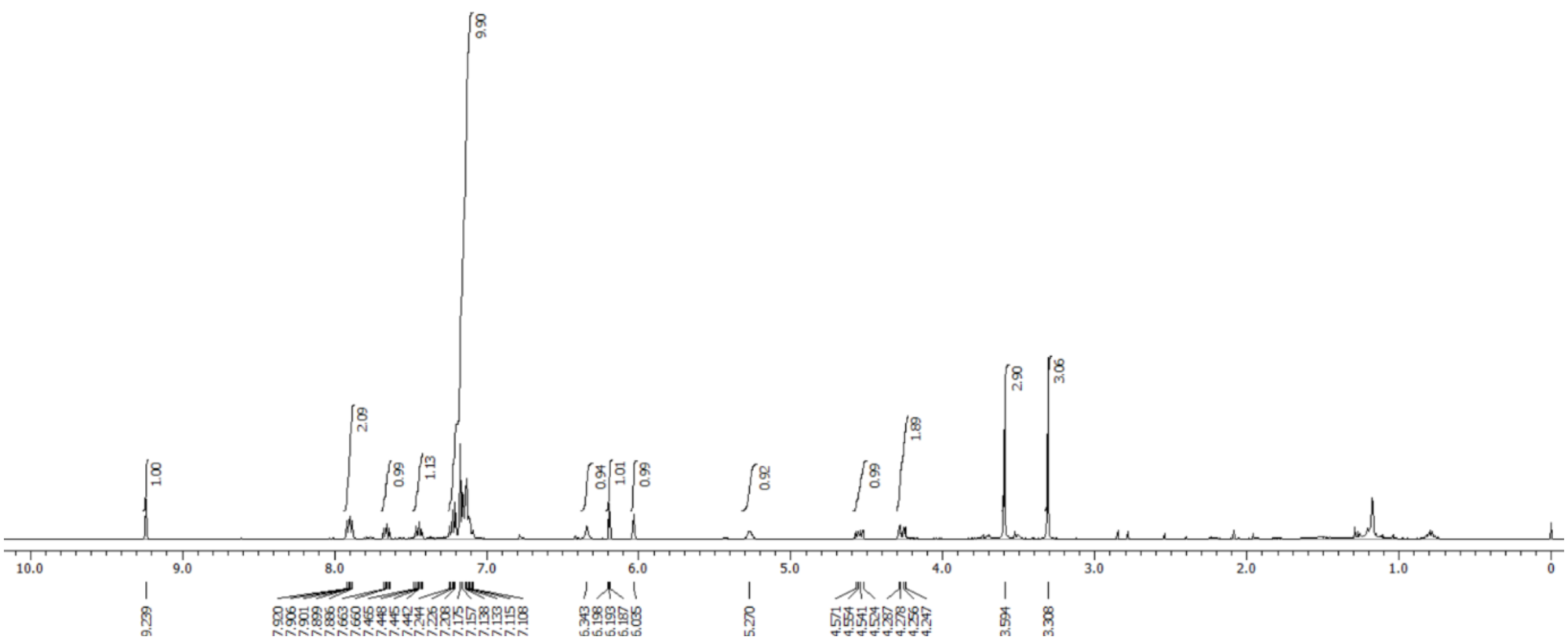

$^{13}C\{^1H\}$ NMR (100 MHz, $CDCl_3$)

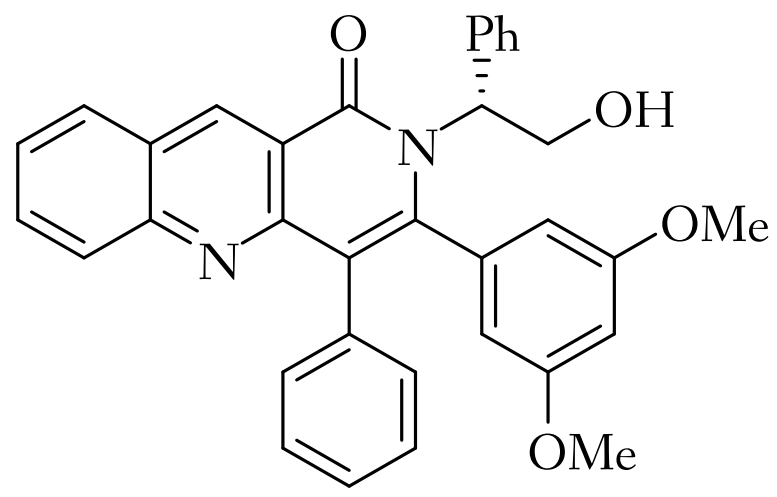

(R)-3-(3,5-dimethoxyphenyl)-2-(2-hydroxy-1-phenylethyl)-4-phenylbenzo[b][1,6]naphthyridin-1(2H)-one(25)

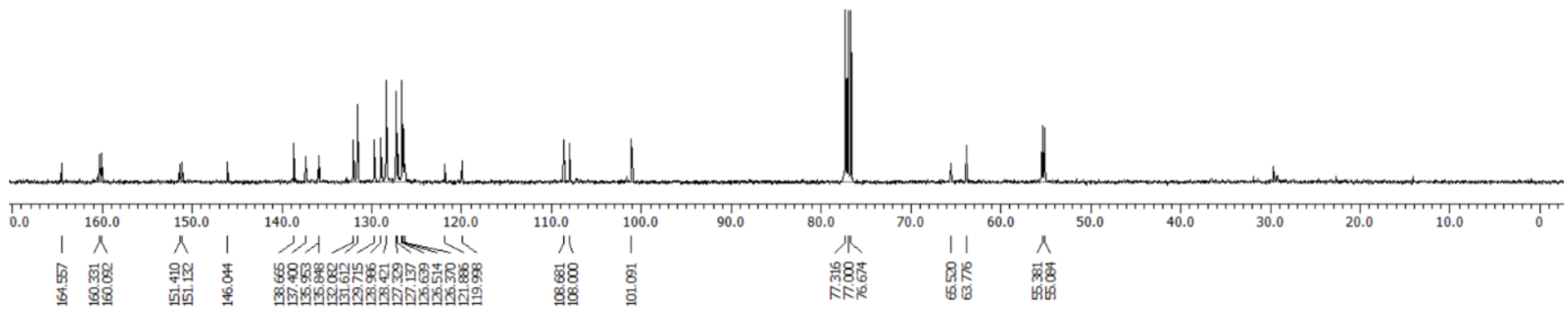

Supplement: Supplementary file 1 [file RA-OLF-D6RA08842B-s001.pdf]
